# Classification and characterization of human endogenous retroviruses; mosaic forms are common

**DOI:** 10.1186/s12977-015-0232-y

**Published:** 2016-01-22

**Authors:** Laura Vargiu, Patricia Rodriguez-Tomé, Göran O. Sperber, Marta Cadeddu, Nicole Grandi, Vidar Blikstad, Enzo Tramontano, Jonas Blomberg

**Affiliations:** Department of Life and Environmental Sciences, University of Cagliari, Cagliari, Italy; Center for Advanced Studies, Research and Development in Sardinia, CRS4, Pula, Italy; Physiology Unit, Department of Neuroscience, Uppsala University, Uppsala, Sweden; Department of Medical Sciences, Uppsala University Hospital, Dag Hammarskjölds Väg 17, Uppsala, 751 85 Sweden; Nurideas S.r.l., Cagliari, Italy

**Keywords:** Human endogenous retrovirus, Classification, Simage, Bioinformatics, RetroTector, Phylogeny, Recombination

## Abstract

**Background:**

Human endogenous retroviruses (HERVs) represent the inheritance of ancient germ-line cell infections by exogenous retroviruses and the subsequent transmission of the integrated proviruses to the descendants. ERVs have the same internal structure as exogenous retroviruses. While no replication-competent HERVs have been recognized, some retain up to three of four intact ORFs. HERVs have been classified before, with varying scope and depth, notably in the RepBase/RepeatMasker system. However, existing classifications are bewildering. There is a need for a systematic, unifying and simple classification. We strived for a classification which is traceable to previous classifications and which encompasses HERV variation within a limited number of clades.

**Results:**

The human genome assembly GRCh 37/hg19 was analyzed with RetroTector, which primarily detects relatively complete Class I and II proviruses. A total of 3173 HERV sequences were identified. The structure of and relations between these proviruses was resolved through a multi-step classification procedure that involved a novel type of similarity image analysis (“Simage”) which allowed discrimination of heterogeneous (noncanonical) from homogeneous (canonical) HERVs. Of the 3173 HERVs, 1214 were canonical and segregated into 39 canonical clades (groups), belonging to class I (Gamma- and Epsilon-like), II (Beta-like) and III (Spuma-like). The groups were chosen based on (1) sequence (nucleotide and Pol amino acid), similarity, (2) degree of fit to previously published clades, often from RepBase, and (3) taxonomic markers. The groups fell into 11 supergroups. The 1959 noncanonical HERVs contained 31 additional, less well-defined groups. Simage analysis revealed several types of mosaicism, notably recombination and secondary integration. By comparing flanking sequences, LTRs and completeness of gene structure, we deduced that some noncanonical HERVs proliferated after the recombination event. Groups were further divided into envelope subgroups (altogether 94) based on sequence similarity and characteristic “immunosuppressive domain” motifs. Intra and inter(super)group, as well as intraclass, recombination involving envelope genes (“*env* snatching”) was a common event. LTR divergence indicated that HERV-K(HML2) and HERVFC had the most recent integrations, HERVL and HUERSP3 the oldest.

**Conclusions:**

A comprehensive HERV classification and characterization approach was undertaken. It should be applicable for classification of all ERVs. Recombination was common among HERV ancestors.

**Electronic supplementary material:**

The online version of this article (doi:10.1186/s12977-015-0232-y) contains supplementary material, which is available to authorized users.

## Background

Endogenous retroviruses (ERVs) have a similar genetic organization as exogenous retroviruses, with two long terminal repeats (LTRs) encompassing the internal coding sequence of the four basic retroviral genes (*gag*, *pro*, *pol* and *env*), which thus are exposed to the vertebrate cellular environment [1]. ERVs have been found in all vertebrates, including humans [2–5]. In some cases, retroviruses can co-exist both as exogenous and endogenous forms in their host populations, e.g. the mouse mammary tumor virus (MMTV) or koala retrovirus (KoRV) [6, 7], however most of the endogenized viruses represent a “relic” of ancestral exogenous retroviral infections. This is apparently the case for human endogenous retroviruses (HERVs).

Many HERVs entered primate genomes over 30 million years ago [8, 9]. Since the first integration waves, most HERVs have been severely damaged in their original genetic structure by accumulation of mutations, insertions and deletions up to the total excision of the internal coding region through homologous recombination between the two flanking LTRs [10–12]. Solo LTRs are the most common HERV trace in the human genome. In a host population, a full proviral integration present in some individuals can coexist with a single LTR with the same flanking sequences in other individuals [13–15]. There are no known replication competent HERVs. However, some, especially the more recently integrated human species-specific HERVK(HML2), still retain some protein coding potential. Some retain the ability to produce virus-like particles [16, 17]. Nonetheless, the conservation of HERV within human DNA over time could be regarded as a balance between “beneficial and detrimental” effects in the host organism [8, 18]. In particular, HERVs and their LTRs can provide promoters (alternative, sometimes bidirectional), enhancers, repressors, poly-A signals and alternative splicing sites for human gene transcripts [19–21].

The pathogenicity of exogenous retroviruses spurred many efforts to find a correlation between HERVs and different human diseases such as cancer, multiple sclerosis and autoimmune diseases, see e.g. [22–24]. However, except for male sterility arising from HERV mediated deletion [25] there is so far no proof of HERV-induced disease [26].

A first important issue of HERV research deals with the different methodologies that have been applied for the identification and classification of the retroviral sequences. Wet-lab and bioinformatics/computational approaches were both used to detect and enumerate HERV sequences, both proviral and solo-LTRs. Generally, HERVs have been identified and classified according to sequence similarity, mainly using sequences in the polymerase (*pol*) gene, and comparing with their exogenous counterparts [4, 27–29]. This approach has led to a number of identified HERV groups (also improperly named as “families”), often ranging between 26 and 31. The copy number of sequences within each group varied from a few members (e.g. HERVFC) up to the large HERVH group with roughly 1000 members and an even greater number of solo-LTRs. A complete list of HERV groups and their copy number remains to be published.

A second important issue deals with the HERV nomenclature that it is still not standardized. Historically, HERV names are linked to the different approaches/methodologies applied for their identification leading to a puzzle of names sometimes difficult to interpret and translate. An up-to-date enumeration and classification of HERV present in the human genome, as well as the introduction of a definitive and standard HERV nomenclature [30, 31] are needed. Studies concerning possible pathophysiological roles of HERV sequences are also dependent on this.

It can be argued that ERV classification should be done at higher host taxonomic levels than in the human host, e.g. in primates. However, the necessity of merging the large body of previous HERV work, and the ongoing intense genetic investigation on humans, justifies a special treatment for HERVs, especially regarding HERV polymorphisms. Moreover, if the investigation is broadened to many different hosts it becomes impossible to handle HERVs in sufficient detail in a single publication. The issue of HERV characterization and phylogeny is large and calls for several publications.

The RepBase [32, 33] and RepeatMasker [34] systems are coordinated and comprehensive efforts to record and categorize all repeatable genetic elements. However, the approach is to identify repetitive sequences, and not to detect entire proviruses. Interpretation of a sequence as a provirus is central for studies on retroviral classification, phylogeny and function. Some of the functionality of, and data from, Repbase are now found in Dfam (http://www.dfam.org/).

RetroTector (ReTe) is a program package [35] implemented for the identification of endogenous retroviruses integrated in vertebrate genomes, including those of primates and humans. ReTe has some advantages, such as the possibility to identify full integrations (not only short sequence pieces), the attempted reconstruction of retroviral protein (termed “putein”), the estimation of open reading frame (ORF) and a preliminary retroviral genus classification. Moreover, ReTe detects proviruses a priori and is not dependent on repetition, giving the capacity to identify low-copy number retroviral sequences, like HERVFC, of which here two “canonical” elements are presented. However, ReTe is not optimized for a complete identification of some class III sequences, such as Spumaretrovirus-like and mammalian apparent LTR-retrotransposon elements (MaLR), as well as for single-LTR detection. What is referred to as “proviruses” may in some instances be processed pseudogenes, i.e. integrated DNA copies of retroviral mRNA.

Here we describe the identification of 3173 HERV proviral sequences in the human genome GRCh37/hg19 assembly using the ReTe software and the development of a classification pipeline. A new approach, called “Simage” (similarity image) analysis led to the classification of 1214 homogeneous, “canonical”, sequences into 39 HERV clades (here named groups), each represented by a consensus or a single sequence. The Simage of a canonical sequence had contributions from essentially one kind of HERV sequence (explained in greater detail in “Methods”). In contrast, the Simages showed that a high percentage of HERVs (1959, 61 %; segregating into 31 additional less well-defined groups), as reconstructed by ReTe, have a heterogeneous content. They were defined as “noncanonical” HERVs, arising from secondary integration of LTRs and other recombination events. We also considered that such proviruses could be artefactual, caused by ReTe joining retroviral fragments coincidentally located within the distance constraints of the program. A particular kind of recombination involved envelopes. We found evidence for frequent “*env* snatching” events.

## Results

### HERV identification and preliminary classification

When the human genome assembly GRCh37/hg19 was screened using ReTe [35] to identify the most intact HERV sequences 3173 HERV retroviral chains with a ReTe score ≥300 (average size 7 kb) were detected. The list of all 3173 reconstructed retroviral sequences together with the main parameters that contributed to their characterization is reported in the supplementary material (Additional file 1: Table S1), and in a publically available.dbf table (see “Methods”).

A preliminary HERV classification, inherent to ReTe, was based on Pol amino acid and nucleotide similarities [27, 29] of the detected HERVs compared to three limited retroviral reference sequence collections obtained from: (1) literature (RvRef; see “Methods”), (2) RepeatMasker/Repbase database (RMRef) and (3) an in-house generated set of 10 Human MMTV-like consensuses (HML; Blikstad et al. unpublished; [30, 36]). Thus, about 60 % of the 3173 HERVs could be initially classified either in class I (Gamma-like, shown as “C” by ReTe), class II (Beta-like, “B”) or class III (Spuma-like, “S”).

**Fig. 1:**
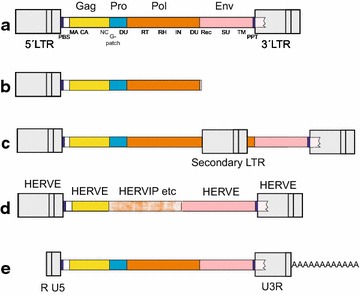
Some retroviral genetic structures encountered during this work. **a** Prototypical provirus, with genes and subgenes. Abbreviations are explained in the text, and/or in [35]. dUTPase occurred in either the protease or polymerase genes. **b** Partial, truncated, provirus. **c** Provirus with secondary integration, often an LTR in sense or antisense direction. **d** Recombinant provirus with contributions from different ERVs, in this case a Harlequin element. **e** Processed pseudogene, i.e. a reverse transcribed genomic retroviral mRNA. Processed pseudogenes were not distinguished from proviruses in the present work

For a more exhaustive classification of the 3173 HERVs, we first generated Clustal guide trees created with Pol amino acid and whole nucleotide sequences together with a broad panel of retroviral reference sequences included for taxonomic purposes (not shown). No Alpha-, Deltaretrovirus- or Lentivirus-like elements were detectable. A minority of the chains seemingly belonged to the large non-autonomous mammalian apparent LTR retrotransposon group (MaLR, class III). Although most LINEs, SINEs and other nonretroviral repeats were removed by ReTe after sweeping with “brooms” optimized for primate genomes [35, 37] before attempted provirus detection, a few aberrant representatives were still present after this procedure. At this stage we encountered chains which behaved in one way when analyzed by Pol amino acid sequence and in another way when analyzed by the chain DNA sequence. Likely explanations for this are mosaicism, repetitive nonretroviral elements remaining in spite of “sweeping” with the “brooms” [35], and outright ReTe mistakes when assembling closely situated defective proviruses. Figure 1 is an overview of the kinds of retroviral sequences which were encountered. For a reliable phylogenetic reconstruction and a definitive HERV classification, mosaic sequences and remaining nonretroviral repeats needed to be excluded. As described below, each of the remainder (“canonical” chains, see “Methods”) could be unequivocally assigned to one specific group. Recursively, these groups could also be used to classify many of the mosaic, “noncanonical”, chains.

### General observations on the dataset

The detected 3173 chains do not represent all HERVs. HERVs constitute 8 % of the human genome [38]. That includes many single LTRs (2 %) and defective MaLR elements (4 %). The 3173 chains reported here constitute 0.5 % of the whole genome, a quarter of the expected 2 %. This reflects the ReTe bias towards more or less complete proviruses. We are confident that our dataset still is of general interest. We tested different classifications until a consistent pattern with a minimal number of groups was evident.

ReTe uses a collection of generic, conserved, motifs. However, *env* and *gag* genes have relatively few generic motifs. If both are missed by ReTe, an entire provirus can be missed if it is defective. This seems to be the main reason for the low representation of HERV Class III proviruses (215 out of 3173 chains). Class III proviruses have an aberrant *gag*, may not have an *env*, and give a low ReTe score. Although a large number of chains scoring below the cutoff of 300 probably are correct this cutoff was necessary to reduce the number of artefacts [35].

An important aspect of proviral recognition is whether it is an independent integration or just part of a genomic rearrangement, like a duplication. We therefore initially estimated the frequency of common flanking sequences in the entire dataset. A few (227) chains had flanks which were >70 % similar to a flank of another chain (with respect to the BLAST score towards their own flanks), indicating that a minor portion (7 %) of the chains were the result of a duplication of a sequence encompassing both the provirus and its flanks (cf. Additional file 1: Table S1). The two HML10 chains on chromosome 6 [39] are an example of this.

### Use of Simages to detect proviral sequences with heterogeneous content

**Fig. 2:**
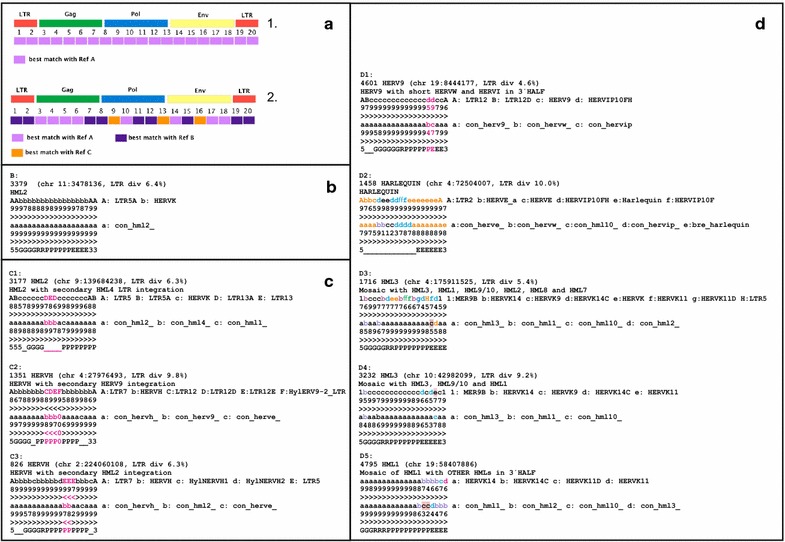
Simages. Panel **a** The principle. A proviral sequence is divided into twentieths, each of which is BLASTed against a reference sequence collection. *1* A homogeneous, canonical, provirus. *2* A heterogeneous, noncanonical, provirus. Panel **b** A canonical chain. The chain id (“rvnr” in Additional file 1: Table S1), HERV classification, the chromosomal position and LTR divergence (if both LTRs were recognized by ReTe) are shown in the uppermost row. The subsequent three rows depict the RepeatMasker nucleic acids with the highest degree of identity, the next three* rows* which of the 39 consensus sequences determined in this paper (Additional file 3: List S3) has the highest degree of identity, all per twentieth of the chain. The lowest row depicts the ReTe putein interpretation per twentieth. *5* means 5′LTR, *G* Gag, *R* Pro, *P* Pol, *E* Env and *3* 3′LTR. Panel **c** Three noncanonical chains containing secondary integrations which left a single LTR inside another retroviral chain. Annotation as in **b**. *Colour* is used here and in ensuing panels to distinguish components of mosaic chains. C1: HML4 LTR inside an HML2. LTR5 and HERVK refer to HML2. LTR13 is an HML4 LTR. C2: HERV9 LTR inside a HERVH. LTR12 and HylERV9-LTR are HERV9 LTR equivalents. A small *pol* piece most similar to HERVE is also present. C3: HML2 inside a HERVH. HylNERVH1 and HylNERVH2 are HERVH equivalents (see Additional file 2: List S2). LTR5 is an HML2 LTR. “0” depicts that no similarity was found with the respective query sequences. Panel **d** Noncanonical chains with signs of recombination. Annotation as in **b**. D1: HERV9 chain with a short piece similar to HERVIP at the end of *pol* and beginning of *env*. D2: a mosaic HERVE with HERVIP, HERVW and HML10 inside. ReTe recognized mainly one gene, *env*. As described in the text, this is a common pattern for chains labeled as “Harlequin”. D3: a complex HML3 chain where the RepeatMasker based Simage indicates contributions from six different HMLs. D4: An HML3 chain with short pieces of HML1, HML9/10 and HML8. D5: a complex chain which contains undetermined HML sequences in the end of *pol*, and whole of *env*. The differences between the consensus and RepeatMasker results in D3-5 indicate that the HML groups and HERVK families contain microheterogeneities, mainly in *env*, which sometimes can cause classification confusion. The HML10 consensus contains an HML9 like stretch in *pol* and an HML8 like stretch in *env*, which may explain some of the discrepancies between the RepeatMasker and Consensus Simages. HERVK14 = HML1, HERVK = HML2, LTR5 = HML2 LTR, HERVK9 = HML3, MER9 = HML3 LTR, HERVK14C = HML9, HERVK11D = HML7, HERVK11 = HML8

To resolve the complex genetic substructure of the identified HERV sequences and properly classify proviruses with heterogeneous content, we developed a novel methodology based on similarity image analysis (Fig. 2a). For this purpose, the retroviral target sequences (regardless of length) were sliced into twentieths. Each twentieth was BLASTed against several sequence collections (RVref, RMref, HML and Consensus). A detailed description is given in “Methods”.

The Simages can be considered as “magnifying glasses” that allow a look inside the proviral sequences. It condenses the distribution of similarity into a few computationally traceable characters, easily stored in tables and databases. Unlike current recombination detection tools, it can simultaneously look for similarities to large datasets, and depict degree of similarity with just one character. It is also a preliminary tool for distinguishing the source of heterogeneous content within HERVs. As shown in the Supplementary Material, we used Xenotropic Murine Leukemia Virus-Related Retrovirus (XMRV) as a test-case. It was previously shown that XMRV probably originated from recombination of two distinct Gamma-like murine ERVs, Pre-XMRV1 and Pre-XMRV2 [40, 41]. We performed a Simage analysis of XMRV and compared it to the Simplot analysis previously reported (Additional file 2: Figure S1). The PreXMRV1 and PreXMRV2 Simages precisely assigned each XMRV portion either to PreXMRV1 or PreXMRV2, validating the methodology.

The RMref consensus sequence collection is composed of retroviral sequence fragments, divided into those from LTRs and from internal sequences. It covers a wide panel of species-specific variants of retroviral sequences from different vertebrates. This naturally leads to an apparent greater heterogeneity of the RM Simages where closely related but differently named ERVs from different species sharing highly conserved portions sometimes occur in the same Simage. This may erroneously indicate a greater heterogeneity than they have (e.g. HERVH chain 467, RepSimage; AbbbcdbbbbbbccbccbbA; where A: LTR7, b: HERVH, c: HylNERVH1 and d: HylNERVH2; HylNERVH is the *Hylobates* [gibbon] HERVH version). To reduce the influence from such seeming heterogeneity, we introduced a “synonym list” (Additional file 2: List S2.5), combined with visual inspection of each chain, which allowed joining of results per twentieth in spite of seemingly different names on the hits.

Simage analysis revealed mosaic noncanonical sequences that contained twentieths derived from different HERV groups but with a backbone structure derived from either Class-I, Class-II, or Class-III. Typically, the backbone structure included one or two LTRs in 5′ and/or 3′ ends and internal hits belonging to the same group as the LTRs, according to Additional file 2: List S2.5. There were many instances where the backbone structure was vague. Although only portions of a full retroviral structure were often detected, the order of motifs and genes conformed to the general retroviral model inherent to ReTe. Additional internal LTRs could generally be attributed to a secondary “piggy-back” integration. The bias of ReTe for a specific succession of motifs could in principle lead to missed aberrant proviral structures. However, comparing ReTe interpretations by eye with those of independent retroviral detection methods, like RepeatMasker, among the 3173 proviral chains of hg19, and earlier work on the mouse [40] and bird genomes [42] did not reveal such aberrations. The proviral chains can be studied in detail in Additional file 1: Table S1, as well is in the.dbf table (see link in “Methods”).

The final results from the analyses of Simages and taxonomic markers of the 3173 HERV sequences (Table 1) showed that among them, 1214 (38 %) could be unambiguously assigned to a specific group (canonical sequences) while 1959 (62 %) could not be unequivocally classified to one group (noncanonical sequences). However, these noncanonical sequences were provisionally assigned to the group which was most commonly observed within the Simage. In unclear situations, the original retroviral backbone on top of which a probable recombination took place could often be deduced from the assignment of the LTRs.

**Table 1:** General HERV identification and preliminary classification in GRCh37/hg19 by ReTe

Probable genus	Type species	HERV genus	Nr of total sequences	Nr of clades
Gammaretrovirus and Epsilonretrovirus	Murine leukemia virus (MLV) Feline leukemia virus (FeLV) Walleye dermal sarcoma virus (WDSV)	Class I (gamma-like, epsilon-like)	2341	Canonical 27, noncanonical 25, total 52
Betaretrovirus	Mouse mammary tumor virus (MMTV) Mason-Pfizer monkey virus (MPMV) Jaagsiekte sheep retrovirus (JSRV)	Class II (beta-like)	598	Canonical 10, noncanonical 0, total 10
Spumaretrovirus	Simian foamy virus (SFV)	Class III (spuma-like), including MaLR (i.e. MST-MLT-THE)	216	Canonical 2, noncanonical 5, total 7
Errantivirus	Gypsy retrovirus	Uncertain_Errantilike	2	Canonical 0, noncanonical 1, total 1
		Unclassifiable	16	
		Total	3173	39 canonical clades 31 noncanonical clades

### Sources of chain mosaicism

The high number of noncanonical chains called for an explanation. The majority of the noncanonical chains contained heterogeneous contributions within the same ERV class, possibly due to *recombination* after cross-packaging of similar genomic RNAs. Certain groups had a higher proportion of noncanonical chains. For example, among Class I HERVE had 72 % (107/148) while HERVH had 48 % (500/1031). Among Class II, HML2 had 78 % (70/89) while HML8 had 41 % (24/58). A small number of cross-class mosaics were also recorded (Additional file 1: Table S1). Some of the noncanonical chains were also studied using BLAT and Genome Browser, which displays RepeatMasker results for genomic matches. Results generally matched well with the Simage analysis (data not shown).

Recombination as a source of mosaicism is further presented under “Envelope diversity”, “Evidence for repeated integrations of recombinant HERVs”, “Comments on specific groups” below, and under specific groups in Additional file 2: List S2.5.

Another cause of mosaicism was *secondary (“piggy back”) integration*. Many of the additional sequences which differed from a retroviral backbone were only LTRs (Figs. 1c,  2c and Additional file 1: Table S1). Examples are the noncanonical HERVH sequences (Fig. 2c2, c3) which harbour secondary LTR integrations, from other Class I and II retroviruses, respectively. The likelihood that an integration is secondary is especially high if the secondary sequence is antisense with respect to the receiving primary sequence, and provides an extra LTR. This is discussed in detail in Additional file 2: Section S2.

As shown in Additional file 1: Table S1, Simages from noncanonical HERVs demonstrated that a wide fraction of the mosaic sequences harbour MaLR (Class III; MST, MLT and THE fragments) on a Class I (n = 51) or II (n = 41) HERV backbone. Although MaLR is the most common retroviral component in the human genome [34, 38, 43], their expansion in vertebrate genomes was calculated to have occurred before 80–100 million years ago, MYA [44]. It is then surprising that we found them so often in chains where the backbone HERV mainly proliferated later than that (see the section on “LTR divergence”, below). It should therefore be investigated whether some MaLR integrations occurred later than 80 MYA, or if there are other mechanisms behind MaLR integrations. Besides recombination or secondary integration a possibility is that ReTe when trying to reconstruct a proviral chain found one of these prevalent retroviral fragments by accident, and included them. The MaLR fragments occurred mainly in the 3′ end, and were often in antisense to the rest of the chain (see below), which is compatible with this explanation [marked with “true” in field “possartifi” (n = 18) of Additional file 1: Table S1]. A more detailed discussion on possibly artificial inclusion of MaLR fragments in ReTe chain is given in Additional file 2: Section S2.2.

The homogeneous (canonical) HERV sequences identified by the Simages could be used both for phylogeny and consensus sequence calculation, avoiding misclassification caused by irrelevant incipient sequences in noncanonical chains.

### Distribution of taxonomic markers among the groups

**Fig. 3:**
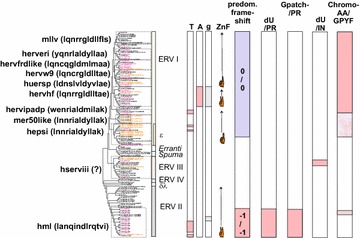
Mapping of taxonomic markers on an unrooted consensus maximum likelihood cladogram of the HERV groups and supergroups. At the *left*, HERV supergroups are shown with the first 13 amino acids of a representative ISD within parenthesis. HSERVIII have no known envelope proteins of their own, symbolized with a question mark. The occurrence of nucleotide bias (High T or A, or low g), predominant number of zinc fingers in Gag, predominant *gag*;*pro* and *pro*;*pol* frame shift strategy, occurrence of dUTPase and GPATCH domains together with the protease and occurrence of dUTPase and Chromo and/or GPY/F domains in the C terminus of the integrase, are shown. *Colour* codes for branch names: consensus sequences (con) are *magenta*, best representatives (bre) are in *brown*. The Chromo and/or GPY/F* reddish* fill was weaker for some groups because of inconsistent (HEPSI) or weak fit (HML6)

When Simage data allowed us to distinguish canonical from noncanonical sequences we could go on to study the frequency of taxonomic markers. None of these markers is absolute [42]. However, when combined with sequence similarity, the main grouping criterion used here, they give a clear indication of which class and group the chain belongs to. These features are described in Tables 2 and 3, and are detailed in Additional file 5: Table S5 and Additional files 2: List S2. However a few comments are given here. The distribution of these features is also described in Fig. 3.

**Table 2:** Taxonomic markers, zinc fingers in NC and frameshifts

HERV Class, and representative groups	Total Nr	Nr of zinc-finger motifs in NC			Frameshift Gag-Pro			Frameshift Pro-Pol		
		0	1	2	−1	0	+1	−1	0	+1
I (gamma- and epsilon-like, canonical and noncanonical)	2341	400	*1371*	304	380	*1128*	295	413	*669*	363
Canonical HERVE	41	6	19	0	6	28	3	9	10	17
Canonical HERVF(A-C)	18	2	1	10	3	10	4	5	4	5
II (beta-like, canonical and noncanonical)	598	213	49	*273*	*149*	111	75	*136*	*145*	76
Canonical HML2	19	4	1	11	6	2	5	10	1	2
III (spuma-like, canonical and noncanonical)	216	*193*	3	0	12	16	9	43	60	31
Canonical HERVL	86	78	1	0	6	7	1	19	25	18
Unc_Erranti-like^a^, noncanonical	2	0	0	2	?	?	?	?	?	?

Major variants are italicized

^a^These chains are incomplete, many markers cannot be identified

**Table 3:** Other taxonomic markers

HERV Class, and representative groups	Nr	dUTPase in Pro	dUTPase in Pol	GPatch in Pro	Chromodomain and/or GPY/F in C terminus of Pol	Nucleotide biases		
						A > 31 %	G < 19 %	T > 31 %
I (gamma- and epsilon-like, canonical and noncanonical)	2341	1	*1*	2	522	290	26	56
Canonical HERVE	41	0	0	0	21	1	0	0
Canonical HERVF(A-C)	18	0	0	0	5	0	0	0
II (beta-like, canonical and noncanonical)	598	395	0	110	16 (14 HML6)	179	11	25
Canonical HML2	19	14	0	11	0	15	0	0
III (spuma-like, canonical and noncanonical)	216	0	18	2	39	11	6	7
Canonical HERVL	86	10	1	1	23	0	0	0
Unc_Erranti-like, noncanonical	2	0	0	2	0	1	1	1

### PBS usage

PBS usage was long the mainstay of HERV classification [45]. However, this trait has proven to be relatively unstable [42]. Moreover, the allocation of a PBS to a specific tRNA can be equivocal. We wanted to check the PBS usage of HERVs in the light of our largely sequence based classification. Therefore all human tRNA sequences were downloaded from the Leipzig tRNA database (see “Methods”). Eighteen nucleotides from the 3′ ends, containing the PBS sequence were recorded. A comprehensive (BLAST) search, with up to two mismatches, in the 3173 HERV chains, yielded 1407 matches. ReTe identified 1406 elements with PBS score >0. Together, 1584 PBS motifs were identified. As explained in “Methods”, a few PBS motifs were probably mislabeled by ReTe (which uses published PBS sequences, indicating errors in the literature). The tryptophan (W; codon CCA) PBS differs only slightly or not at all from Arginine (R; ACG) PBS, cf. Additional file 6: Table S6. This affected mainly HERV9 and HERVW chains.

Additional file 1: Table S1 contains all PBS sequences detected by ReTe (with and without PBS score) and those matching a Leipzig sequence with up to two mismatches. It turned out that several major groups frequently used other tRNAs than earlier reported. For example, of 532 canonical HERVH chains 386 used H, 57 F and 19 K. Of 87 canonical HERVL chains 35 used L, 33 M and 2 S.

### Nucleotide bias

A well known example of nucleotide bias is HIV, where copackaging of a cellular post-transcriptionally active cytidine deaminase, APOBEC, gives a bias for “A” [46, 47]. For example, HIV-1 hxb2 (Genbank ID K03455.1) contains 35 % “A”. As shown in Table 3, Additional file 5: Table S5-1 (Excel sheet 1 of Additional file 5: Table S5), Additional file 1: Table S1 and Fig. 3, several of the HERV groups (HERVIP, HERVADP, HEPSI2, HEPSI3 and HML1-3 and HML10) show an “A” frequency of over 31 %, higher than in other HERV sequences, where it ranged between 23 and 30 % (average 28 %). It has been demonstrated that APOBEC can modify the genomes of several retrotransposons [48, 49]. Thus, several HERV sequences may have been influenced by this mechanism. Besides the bias for “A”, some HERVs (HERVH, HERVFB, HERVFC, LTR46, HML7 and HML8) had a bias against “G”, in accordance with our earlier observations [50]. A third bias, towards increased “T” frequency, occurred in LTR46, HERVFB, HERVFC and HERVH. The mechanisms behind the latter two biases are unknown.

AutoFrame search was another taxonomic tool. It allowed finding protein similarity with reference sequences which have not been formally translated into protein. Protein similarity searches can be made over longer evolutionary distances than those based on nucleotide similarity. As described in “Methods”, the AutoFrame mechanism depended on the presence of reading frames of ≥130 amino acids in the RepeatMasker library of 17500 LTR retrotransposons from a wide variety of hosts. That property is obviously biased towards recently replication competent members with long ORFs. This explains the sometimes unexpected hits in Additional file 1: Table S1, and Additional file 2: List S2, e.g. with Errantiviral (*gypsy*) and Pseudoviral (*copia*) elements from invertebrates. These hits often occurred at the end of Gag, over the highly conserved zinc fingers, and do not necessarily indicate a recent evolutionary relationship. Several hits covering longer sequence segments do however indicate a relationship worthy of further exploration. The AutoFrame mechanism allowed us to look for related retroviral sequences in a broad set of organisms covered by the RepeatMasker library.

### The “immunosuppressive domain” of the transmembrane protein

The paucity of conserved motifs, especially in the aminoterminal half, of Env necessitated a special effort for detection and characterization of envelopes. The so-called immunosuppressive domain (ISD, [51]) is a conserved feature which is often easily detectable (motifs TM2-TM4 in ReTe), and which is characteristic of the group. ERV Class I have especially characteristic ISDs. We used the ISD as an aid in the classification of envelopes.

### Other taxonomic markers

We studied the number of zinc fingers in Gag, translational frame shifts, dUTPase and Gpatch in *pro*, and dUTPase, chromodomain and the GPY/F motif in the C terminus of Pol. Proprietary programs were written for these purposes, as described in “Methods”. The results are shown in Tables 2, Additional file 1: S1 and Additional file 2: List S2.5. A graphical overview is given in Fig. 2.

### Consensus sequences and phylogenetic trees

Finally, the defined HERV groups were analyzed for their degree of heterogeneity through the generation of majority consensus sequences from their DNA sequences as well as their reconstructed Gag, Pro, Pol and Env puteins within each group (Additional file 3: List S3).

The “width” of the groups (represented by the number of members) was fine-tuned based on the properties of the consensus sequences of the group. We strived for at least 50 %, optimally 80 % [30], of both “intermember identity” (degree of identity within the group, WIGI) and “identity to consensus” (ITC) within the group (Additional file 5: Table S5-3). A third measure of consensus heterogeneity was the number of nucleotide or amino acid positions which were identical in more than 50 % of the members of the group. The proportion of positions which did not fulfill this criterion, was called “heterogeneity”. Calculation of consensus sequences allowed condensing HERV variability into a small sequence set which is useful for classification and phylogenetic inference. The consensuses will also be useful for identification of unknown retroviral sequences occurring in large scale sequencing efforts, e.g. aimed at pathogen discovery.

### Place of HERV groups in retroviral phylogeny

**Fig. 4:**
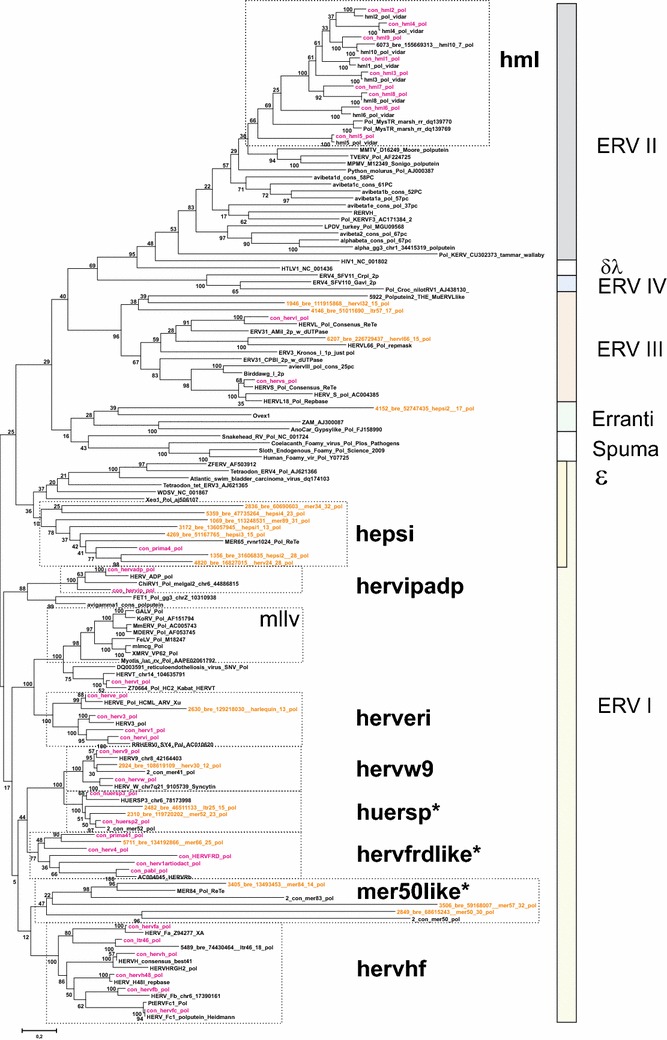
Unrooted phylogram of Pol consensus sequences (“con”, *magenta*) of canonical and best representatives (“bre”; *brown*) of some noncanonical proviruses together with reference Pols from GenBank (with Genbank id, *black*), and previous work by the authors (“2-con” were previous consensus sequences). Pol sequences were aligned with Muscle. A maximum likelihood tree was calculated. The *asterisks* mark the three supergroups which contain RepBase clades belonging to RepBase group MER4I

Maximum likelihood (ML; Fig. 4), and Neighbor joining (NJ) trees (not shown) generated with Pol-based consensus sequences together with a broad panel of both exogenous and endogenous reference sequences showed a consistent topology. A similar topology was seen in the nucleotide based tree, Additional file 2: Fig. S2.1.

We show the Pol consensus sequences of canonical and best representatives of some noncanonical proviruses, together with a wide variety of reference Pol sequences, in the unrooted phylogram of Fig. 4. In general, the great variety of retroviral sequences in the hg19 HERV collection often led to a weak bootstrap support in the most basal branches. Clustering of Pol at the amino acid level minimized this problem. The HERV groups clearly segregated into ERV class I, II and III. None clustered with the newly defined ERV class IV [52]. Except for one chain (rvnr 4152) none clustered with errantiviruses. Interestingly, avian (*Gallus gallus*), crocodylian (*Alligator mississippensis*) and turtle (*Chrysemis picta bellii*) ERV Pols (some of which included dUTPase) intermingled with the Class III HERV Pols, here given the supergroup name “HSERVIII” (cf. the AviERVIII group [42]). HSERVIII clustered with spumaretroviruses [53, 54] and close to epsilon-like elements, as noted before [50].

**Fig. 5:**
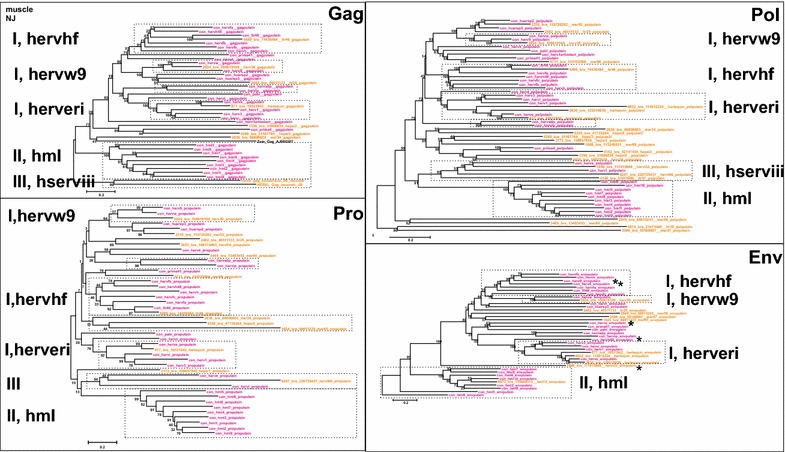
Unrooted phylograms of Gag, Pro, Pol and Env from the consensus sequences in Additional file 4: List S4, with fewer reference sequences than in Fig. 4. A maximum likelihood tree was calculated from Muscle alignments. The* asterisks *mark instances of possible Env recombination

The evolutionarily oldest relations seemed to be concentrated to the middle section, clustering around an errantiviral (Zam) Pol. A similar organization was seen in the Gag tree of Fig. 4. A group appearing close to the most basal branches of the Class I gamma-like group clustered with Pol and Gag of the exogenous epsilonretrovirus, walleye dermal sarcoma virus (WDSV). Both Simage and phylogenetic reconstruction using different genes (Figs. 4,  5), supported this relationship, which justified the classification of these sequences as separate HERV groups (here named “HEPSI”1-4) [50, 55]. The HEPSI supergroup is further discussed below, and in Additional file 2: List S2.5.2.2.8.

### Final HERV classification

The final number of proviral sequences and the HERV clade (group) assignments are reported in Table 4. Even if some noncanonical sequences were difficult to classify, 96 % of the 3173 proviral sequences identified in GRCh37/hg19 could be assigned into Class I (Gamma- and Epsilon-like), II (Beta-like) and III (Spuma-like, including the MaLR group), plus two uncertain, vaguely Errantilike, chains, whereas 16 remaining chains, mainly consisting of non-LTR retrotransposons, were not classified (Tables 1, 4). Both canonical and noncanonical chains were allotted a taxonomic number specific for a certain HERV group (“taxorder” in Additional file 1: Table S1). It was useful for generation of sorted lists like Additional files 2, 4 and 5: S2.5, S4 and S5.

**Table 4:** List of 39 canonical HERV clades found in GRCh37/hg19

Tax-order	HERV clade	Nr of HERV sequences^a^		Repbase identifiers^b^	Previously estimated nr of copies^c^
		Canonical	Non-canonical		
	Class I, Gamma-like				
10100	Supergroup MLLV*				
10110	HERVT	21	12	HERVS71/LTR6	80
10200	Supergroup HERVERI				
10210	HERVE	41	107	HERVE/LTR2	250
10230	HERV3	20	37	HERV3/LTR4	100
10240	HERV1	2	11	HERV1	NA
10250	HERVI	3	13	HERVI/LTR10	250 (together w. HERVIP)
10300	Supergroup HERVW9				
10310	HERVW	40	86	HERV17/LTR17	40
10320	HERV9	114	171	HERV9/LTR12	300
10400	Supergroup HERVIPADP				
10410	HERVIP	67	72	HERVIP10F/LTR10F	250 (together with HERVI)
10420	HERVADP	16	8	HERVP71A_1/LTR71	40
10600	Supergroup HERVHF				
10610	HERVH	531	500	HERVH/LTR7	1000
10620	HERVH48	16	8	HERVH48I/MER48	60
10630	HERVFA	8	7	HERVFH19/LTR19	45
10640	HERVFB	8	14	HERVFH21/LTR21A	30
10650	HERVFC	2	3	HERV46I/LTR46	6^d^
10660	LTR46	8	2	LTR46-in/LTR46	NA
10700	Supergroup HERVFRDlike				
10710	HERVFRD	1	10	ERV3-1-i/LTR58 MER50	NA
10720	PRIMA41	3	17	PRIMA41/MER41	40
10740	HERV1_artiodact	2	7	NA	NA
10750	PABL	2	8	PABL_BI/PABL_A, PABL_B	8
10760	HERV4	8	23	NA	NA
10800	Supergroup HEPSI				
10820	HEPSI2	2	4	NA	NA
10830	HEPSI3	1	5	NA	NA
10852	MER65	1	1	MER65/MER65C	NA
10882	PRIMA4	3	4	PRIMA4	NA
10900	Supergroup HUERSP				
10910	HUERSP1	1	3	HUERSP1/LTR8	200 (together with other HUERSP)
10920	HUERSP2	10	12	HUERSP2/LTR1_LTR28	See above
10930	HUERSP3	16	40	HUERSP3/LTR9	See above
	Class II Beta-like				
	Supergroup HML				
20010	HML1	9	45	HERVK14I/LTR14	70
20020	HML2	19	70	HERVK/LTR5	91^e^
20030	HML3	31	151	HERVK9I/MER9	150
20040	HML4	7	5	HERVK13I/LTR13	10
20050	HML5	27	69	HERVK22/LTR22	100
20060	HML6	17	48	HERVK31/LTR3	50
20070	HML7	9	5	HERVK11DI/MER11D	20
20080	HML8	34	24	HERVK11I/MER11A	60
20090	HML9	10	9	HERVK(14C)/LTR14C	25
20100	HML10	2	7	HERVKC4/LTR14	10
	Class III Spuma-like				
	Supergroup HSERVIII				
30100	HERVL	86	75	HERVL (HERVL/MLT2)	200
30200	HERVS	16	4	HERVS (HERV18/LTR18)	50

Only groups and supergroups with at least one canonical chain are included. Additional file 6: List S6 is more comprehensive

^a^Number of canonical and non-canonical HERV sequences identified in this study (see details in the main text)

^b^see Bannert and Kurth [8] and Mager and Medstrand [56]

^c^see Mager and Medstrand [56]

^d^see Bénit et al. [97]

^e^see Subramanian et al. [98]

A total of 39 canonical HERV groups are listed in Table 4, in which both the number of the canonical and noncanonical classified sequences per each group is reported in comparison with the previously estimated proviral copy numbers [56]. Some of the HERV groups presented here represent a merge of groups that have been previously indicated as separated groups. This is elaborated in Additional file 2: List S2.5. In order to compare our results with those previously reported, HERV groups identified here were, when possible, named according to established nomenclature (common names and/or RepBase identifiers).

Although a broad correlation between previous classification and enumeration attempts was observed, a strict comparison between the two sets of data (nomenclature and copy numbers) was not easy because of previous different strategies used for HERV identification and classification. For example, most of the copy numbers reported by [56] were estimated from BLAST searches of the human genome sequence available at the NCBI in August 2001. The agreement was especially clear for the more characterized HERVs, like HERVW, HERVH or HERVK(HMLx).

### Homogeneity of the chosen groups

During construction of HERV groups, we strived for at least 80 % Pol identity to the consensus sequence (“ITC”) [30]. As shown in Additional file 5: Table S5, the divergence from consensus was expressed in four ways, identity within the group [“WIGI”; varying between, for nucleic acids, 36 and 90 % (grand average 72 %) and for Pol putein amino acids, between 38 and 80 % (grand average 62 %), respectively], average divergence from consensus [“ITC”; range for nucleic acid consensus 24–95 % (grand average 78 %), and for Pol amino acid 59–90 % (grand average 75 %)], average portion of conserved sites [nucleic acid 0–0.72 (grand average 0.42), Pol amino acid 0.11–0.88 (grand average 0.40)] and frequency of gaps in the consensus sequence [nucleic acid 1–60 % (grand average 29 %), Pol amino acid 3–80 % (grand average 27 %)]. The two consensus HML10 sequences on chromosome 6 [39] were recently created by a large gene duplication. They gave artificially high levels of identity and were not included in these figures. Only one HERVFC consensus sequence yielded a Pol putein, hence an average could not be computed. We reached a Pol ITC of at least 80 % for 12 groups. Twenty-seven groups reached a Pol ITC of at least 70 %, and for a Pol ITC of at least 60 % only one group (HML1; 59 %) fell below. Comparable results were obtained for the other measures of group heterogeneity. In general, the groups were judged to be homogeneous enough to be handled as discrete retroviral entities and their consensuses useful for detection and classification.

### Envelope diversity

**Fig. 6:**
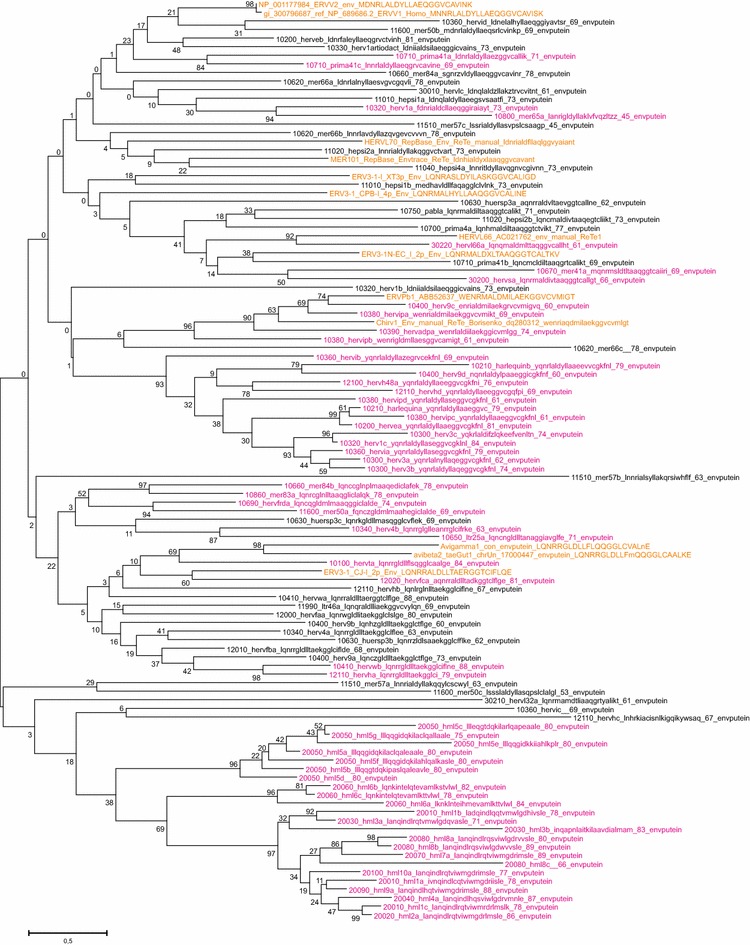
Retroviral envelopes encountered in hg19. Env subgroup consensuses (see Additional file 4: List S4) and reference envelope proteins were aligned by Muscle. A Maximum Likelihood tree was then produced. Branch names of the subgroup consensuses contain, in this order, taxorder nr, “con”, subgroup name, subgroup average percent identity to consensus for the envputein (if the subgroup had only one member, a 0 is shown), a 13 amino acids subdomain from the ISD (if identified), subgroup average percent identity to consensus for 23 ISD amino acids (Additional file 1: Table S1) and bootstrap value of the relation (percent of 100 bootstraps)

**Fig. 7:**
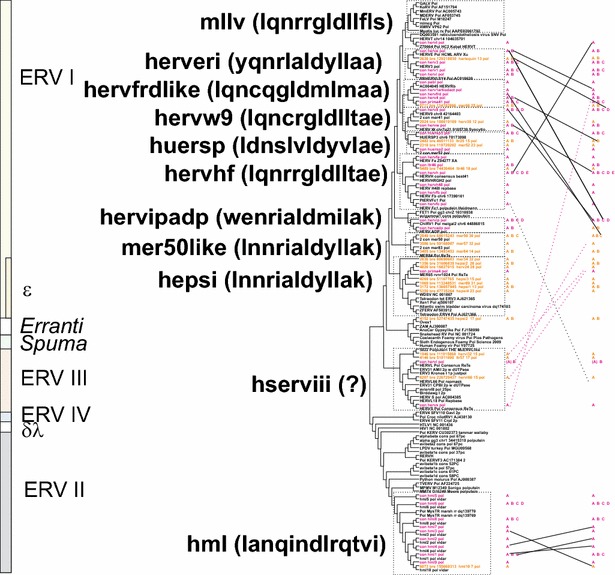
Retroviral envelopes with high similarity between Env subgroups. Envelope subgroups (A, B, C, etc) with high intersupergroup similarity are shown interconnected, superimposed on the cladogram of Fig. 3. Significant relations (branches with bootstrap >50) were obtained from neighbour joining (not shown) and maximum likelihood trees (same as in Fig. 6). To avoid cluttering, only intersupergroup relations were shown, except for the HML supergroup, where intergroup relations are presented. Relations shown indicate, but do not prove, an envelope transfer event

Envelope puteins were predicted for 944 chains (29 consensuses of 39 canonical groups; Additional file 1: Table S1). Figure 4 depicts the branch pattern for Gag, Pro, Pol and Env puteins of the 39 canonical groups and best representative chains for some noncanonical chains. The branch patterns were similar for Gag, Pro and Pol, but differed in the Env group consensus tree in some conspicuous cases. HERV9 Env clustered with HERVHF Envs. HERVS (a class III HERV) clustered with PRIMA41 (a class I HERV) Env. When Env puteins from the noncanonical HERVL32 and HERVL66 were analyzed, their Env also clustered with Class I Envs (Figs. 4,  6,  7, Additional files 2: Lists S2 and Additional file 4: S4). The Harlequin element Simages (see below) contained a prominent Env. This Env was highly similar to HERVE Env. Many of these elements were otherwise frequently deleted or defective in the other three major genes (Fig. 2d). Note that a heterogeneity was found in many of the Env sequences, leading to Env subgroups (see below).

As shown in the trees of Figs. 3 and 4, envelope consensuses sometimes clustered differently from the pattern in the trees constructed from the other three major proteins. Intra- and interclass rearrangements involving Env were noted for HERV9, HERV4, HERVS, HERVL32 and HERVH48. We therefore used the Autoframe hits for envelope puteins, ISD sequences deduced by a dedicated program (“henzyscore”, yielding “envhpoints”, see “Methods”), plus several Env putein trees (Additional file 2: Fig. S3a, b, and others not shown), to divide the envelopes into subgroups. We noted that some sequences (mainly HERVL) had evidence of being artefactual. An Env quality control program (“EnvQual”, yielding “envqpoints” see “Methods”) was therefore written. When a cutoff of 6 envqpoints was used (Additional file 2: Fig. S3), 286 of 944 Env puteins predicted by ReTe could be excluded as possible artefacts. New Env (n = 94) consensuses were calculated based on these subgroups (Additional file 4: List S4). Trees based on these consensuses revealed that there was an even greater heterogeneity at the Env subgroup level than brought out by the group Env consensuses (Figs. 6,  7). The FASTA names of Additional file 3: List S3 include especially clear intergroup similarities. Interclade Env similarities are further discussed in Additional file 2: Section S3.

### Evidence for repeated integrations of recombinant HERVs

Even defective recombinants can be packaged and reintegrated by a more complete retrovirus, so-called “midwife” elements [36, 57]. The following features were considered as evidence for a reintegration potential of a mosaic HERV. 1. Several chains with similar internal mosaic structure, all in sense, but different flanks (degree of flank identity <70 %). 2. Same LTR in 5′ and 3′ and absence of a third internal unrelated LTR. Six Harlequin, 4 HERV1, 3 HERV3, 1 HERV30, 26 HERV9, 20 HERVE, 3 HERVI, 10 HERVIP, 2 HERVW, 1 HML1, 17 HML2, 2 HML3 and 2 HUERSP3 (in total 97 chains) fulfilled these criteria. The Harlequin-related recombination candidates, which had contributions from HERVE, HERVI, HERVIP, HERV3, HERV30, HERV9, HERVW and LTR19, are discussed below.

It is also possible that a recombinant chain represents a retrovirus which was infectious at the time of integration. Additional criteria, on top of the above two, were used for accepting a recombinant chain as being of possible infectious origin: 3. Presence of all four major genes (*gag*, *pro*, *pol* and *env*, in this order), and 4. Not more than one unexplained twentieth (shown as a “0” in the Simage) per chain. Using these four criteria there remained, among class I HERVs; 22 HERV9 with HERVIP in 3′ half, 5 HERVIP with HERV3 and LTR19-int inside (both patterns are similar to the Harlequin mosaic), among class II 17 HML2 and 2 HML3 (Fig. 2d) with the mosaic patterns mentioned under “HML” in Additional file 2: List S2 Altogether 46 chains fulfilled these stringent criteria (marked “true” in field “possinfrec” of Additional file 1: Table S1). Thus, there is evidence that HERVE (the backbone of Harlequin), HERV9, HERVIP and HML2 were especially active in spawning infectious recombinant retroviruses.

### Comments on the chosen groups and definition of supergroups

Based on clustering in the protein and nucleic acid based trees, and taxonomic markers, the 39 canonical HERV groups could be placed in 11 HERV supergroups. Some noncanonical chains were also classified into the supergroups.

### Class I (Gammaretroviruslike) supergroups

#### MLLV* (Mouse leukemia virus like virus related supergroup, taxorder 10100)

HERVT is highly related to, but not formally part of, the MLLV supergroup. It was marked “MLLV*” in Additional file 1: Table S1 and Table 4. MLLV provisionally includes murine, feline and porcine gammaretroviruses [40]. HERVT is also similar to the avian reticuloendotheliosis virus. LTR divergence ranged between 8 and 13 %.

#### HERVERI (HERVE, HARLEQUIN, HERV3, HERVI, HERV1; taxorder 10200)

Recombination is especially common in this supergroup (Figs. 2c,  5, Additional files 2, 4: Fig. S3, Additional files 2 and 4: Lists S2 and S4).

The range of average LTR divergence was 5–24 %.

The HERVE group contains many ORFs (Tables 5 and 6). Although the distributions are wide and overlapping, Harlequin (range 2–49 %, average 8 %) may be younger than HERVE (noncanonical: range 2–49 %, average 16 %; canonical: range 2–41 %, average 11 %) (Figs. 1,  2c,  5).

**Table 5:** HERVs with the most intact reading frames, overall

Rvnr	Chr	Chainstart	Subgenes	Non-canon	Canon	Gag	Pro	Pol	Env
2025	5	156093754	5PMCNDPrRISTPp3	HML2		*0/0*	*0/0*	*0/0*	1/0
2410	7	4631501	5PCNDPrRISTPp3	HML2		1/0	*0/0*	*0/0*	*0/0*
2704	8	7364785	5PMCNDPrRISTPp3	HML2		*0/0*	0/1	*0/0*	*0/0*
3625	11	101565821	5PMCNDPrRISTPp3	HML2		1/0	*0/0*	*0/0*	*1/0*
6069	1	155605494	5PMCNDPrRISTPp3	HML2		1/0	*0/0*	*0/0*	*0/0*
4695	19	28137359	PMCNDPrRISTPp3	HML2		*0/0*	0/1	0/1	0/0
1260	3	185289375	5PMCNDPrRISTPp3	HML2		0/1	0/1	*0/0*	1/0
2409	7	4639990	5PMCNDPrRISTPp3	HML2		1/0	0/1	*0/0*	*0/0*
4434	17	26566203	5PMCNPrRISTPp3	HERVE		*0/0*	*0/0*	1/2	2/1
5600	X	97096726	5PMCNPrRIT3		HERVFC	1/0	*0/0*	1/1	*0/0*
1067	3	112752255	5PMCNDPrRISTPp3	HML2		*0/0*	1/1	0/1	0/1
2214	6	78436056	5PMCNDPrRISTPp3	HML2		*0/0*	*0/0*	0/3	*0/0*
3808	12	58730671	5PMCNDPrRISTPp3	HML2		*0/0*	*0/0*	0/3	*0/0*
6264	22	18926329	5PMCNDPrRISTPp3	HML2		*0/0*	1/0	1/1	*0/0*

The 15 most intact chains, sorted according to number of stops and shifts in *gag*, *pro* and *pol*. The criterion for inclusion was a maximum sum of shifts and stops in the three genes of 3. ORFs are marked in italics

Format: “Stops/shifts” are shown for the four major frames. Abbreviations in column Subgenes: *5* 5ʹLTR, *P* PBS, *M* MA, *C* CA, *N* NC, *D* DU, *Pr* Prot, *R* RT, *I* IN, *S* SU, *T* TM, *Pp* PPT, *3* 3ʹLTR. “Rvnr” is the chain identity number in Table S1, Additional file 1

**Table 6:** Chains with the most intact *env*

Rvnr	Chr	Chainstart	Subgenes	Noncanon	Canon	Gag	Pro	Pol	Env	Envgroup2
2862	8	74818435	5PMCNPrRITPp3		HERV9	5/4	*0/0*	6/9	*0/0*	HERV9_a
5600	X	97096726	5PMCNPrRIT3		HERVFC	1/0	*0/0*	1/1	*0/0*	HERVFC_a
738	2	166572669	5PMCNPrRISTPp3		HERVH	1/4	*0/0*	3/0	*0/0*	HERVH_a
4229	14	106663735	5PCNPrRIST3		HERVT	2/2	1/0	4/2	*0/0*	HERVT_a
2556	7	92107320	5PNPrRITPp3		HERVW	*0/0*	1/0	4/7	*0/0*	HERVW_a
2214	6	78436056	5PMCNDPrRISTPp3	HML2		*0/0*	*0/0*	0/3	*0/0*	HML2_a
2409	7	4639990	5PMCNDPrRISTPp3	HML2		1/0	0/1	*0/0*	*0/0*	HML2_a
2704	8	7364785	5PMCNDPrRISTPp3	HML2		*0/0*	0/1	*0/0*	*0/0*	HML2_a
3808	12	58730671	5PMCNDPrRISTPp3	HML2		*0/0*	*0/0*	0/3	*0/0*	HML2_a
6069	1	155605494	5PMCNDPrRISTPp3	HML2		1/0	*0/0*	*0/0*	*0/0*	HML2_a
6264	22	18926329	5PMCNDPrRISTPp3	HML2		*0/0*	1/0	1/1	*0/0*	HML2_a
2410	7	4631501	5PCNDPrRISTPp3	HML2		1/0	0/0	*0/0*	*0/0*	HML2_a
4695	19	28137359	PMCNDPrRISTPp3	HML2		*0/0*	0/1	0/1	*0/0*	HML2_a
5931	1	75842927	5PMCNDISTPp3		HML2	*0/0*	*0/0*	*0/0*	*0/0*	HML2_a
679	2	130727258	NRISTPp3	HML2		*0/0*	*0/0*	14/20	*0/0*	HML2_a
4648	19	20938119	5PCNPrRISTPp3		HERVE	3/1	*0/0*	4/2	1/0	HERVE_a
721	2	155723861	5PMCNPrRISTPp3		HERVH	5/3	1/1	6/3	1/0	HERVH_a
1220	3	166547460	5PMCNPrRSTPp3		HERVH	3/5	*0/0*	9/7	1/0	HERVH_a
2213	6	78376119	5PMCNPrRIST3		HERVH	1/8	*0/0*	3/2	0/1	HERVH_a
4639	19	20472849	5PCNPrRIT3		HERVT	3/3	3/1	11/2	0/1	HERVT_a
1067	3	112752255	5PMCNDPrRISTPp3	HML2		*0/0*	1/1	0/1	0/1	HML2_a
1260	3	185289375	5PMCNDPrRISTPp3	HML2		0/1	0/1	*0/0*	1/0	HML2_a
1793	5	30496057	5PMCNDPrRISTPp3	HML2		2/0	*0/0*	2/1	1/0	HML2_a
3625	11	101565821	5PMCNDPrRISTPp3	HML2		1/0	*0/0*	*0/0*	1/0	HML2_a
6079	1	160660602	5PMCNDPrRISTPp3	HML2		3/2	*0/0*	3/2	0/1	HML2_a
2025	5	156093754	5PMCNDPrRISTPp3	HML2		*0/0*	*0/0*	*0/0*	1/0	HML2_a
3340	10	101587714	PMCNDPrRISTPPT	HML2		0/2	0/1	1/1	0/1	HML2_a
2976	8	140482803	MCNPrRITPp3	HML2		*0/0*	*0/0*	*0/0*	0/1	HML2_a
5759	X	148763072	CNDPrRITPPT	HML5		*0/0*	2/1	8/2	0/1	HML5_a
691	2	136836895	CPrITPp3	HML6		*0/0*	*0/0*	8/3	0/1	HML6_c
2048	5	171824497	NPrRSTPPT		HML8	*0/0*	*0/0*	1/ 2	0/1	HML8_a
3898	12	105703025	RISTPPT	HML8		*0/0*	*0/0*	4/7	0/1	HML8_a
2521	7	64460314	5PMCNPrRIST3		HERV3	3/4	1/0	12/5	2/0	HERV3_b

The 33 most intact chains with respect to envelope genes, sorted according to number of shifts and stops in *env*. The criterion for inclusion was a maximum of 1 shift or stop in *env*. Because of its publication record (see e.g. Hervé et al. [99] and Fei et al. [100]) a HERV3 chain with 2 stops in *env* was also included. Format: “Stops/shifts” are shown for the four major frames. Abbreviations are as in Table 5. Envgroup2 is the envelope group classification shown in Additional file 1: Table S1.

*Harlequin* This remarkable group of recombinants requires a thorough description. As described by Kapitonov and Jurka [58], Harlequin had a complicated structure of LTR2-HERVE-MER57I-LTR8-MER4I-HERVI-HERVE-LTR2. They suggested that these recombinant forms were created by copackaging of different proviral RNAs and polymerase jumps between them. To better understand this mechanism we created Simages for all HERVs using the published Harlequin sequence as a query. The following results emerged: there were a large number of hits (539 chains). Most (406 chains) were noncanonical chains classified as HERVE, HERVI, HERVIP, HERV9, HERVW or Harlequin. The rest (133 chains) were canonical chains with no or a minor heterogeneity. The pattern of matches was complicated, from 1–3 twentieths matches per chain to one of HERVW, HERV9, HERVIP, HERV3, LTR19, and HERVI (162 chains), to 4–9 twentieth matches per chain (227 chains) involving HERV3, HERVI, HERVE in various combinations, to more extensive matches of 10–16 twentieth matches per chain (82 chains); containing HERVE, HERVIP and Harlequin itself. Finally, there were 68 chains where 17–20 twentieths best fitted with Harlequin itself. This indicated a complex series of recombination events, some ending up with Harlequin. This mosaicism makes the classification of HERVI, HERVIP and HERV3 especially difficult. Most (162) of these chains had an LTR2 (the HERVE LTR) in the 5′ or 3′ end. Ten had two LTR2. LTR10 (the HERVIP LTR) occurred in one terminal position in 23 chains, in 13 cases in two. Thus, HERVE and HERVIP backbones were most frequent in these Harlequin-related recombinants.

#### HERVW9 (HERV9, HERVW, HERV30, MER41,HERV35,LTR19; taxorder 10300)

HERV9 was related to MER41 and HERV30, more distantly to HERVW (Fig. 4). LTR divergence ranged from 9 to 31 %).

**Fig. 8:**
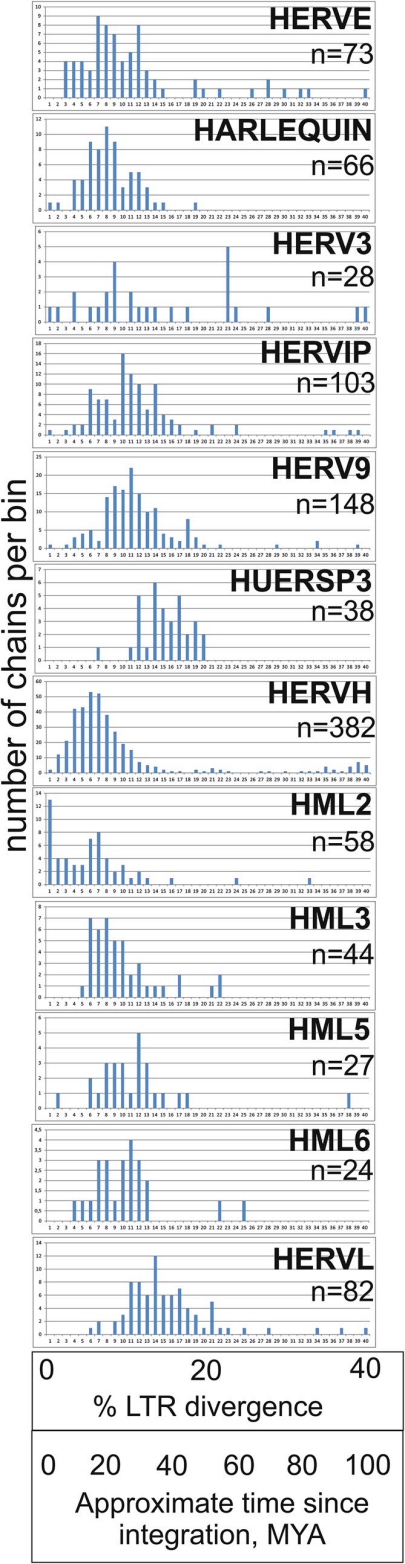
LTR divergence of frequent HERV groups. LTR divergence as calculated by ReTe is presented as a histogram divided into percent bins, from 0–1 to 39–40 %. A very approximate estimate of age since integration was calculated by multiplying percent divergence with 2.5. It is primarily intended to show the distribution of divergence of prominent HERV groups relative to that of other HERV groups

The HERVW integration on chromosome 7, band q21, is the origin of Syncytin-1 [59]. This *env* ORF is one of several ORFs in the supergroup (Tables 5 and 6). HERV9 chains commonly showed signs of recombination. Simages of 37 noncanonical HERV9 chains had a twentieth deriving from HERVI in the 3′ half. 30 of them also had a twentieth most similar to HERVW just before this HERVI. None shared flanks with the other 37. All 37 had at least one LTR12 (the HERV9 LTR) in 5′ or 3′ end. Sixteen had two LTR12. All had a discernible Gag putein, 34 had a Pro, 28 a Pol, and 17 an Env. The average LTR divergence of the 16 which had 2 LTR12s was 11.5 % (st. dev. 7.2 %, min 2.5 %, max 44.5 %). Although the limitations of determining integration time from LTR divergence should be considered, these recombinants could have integrated during a long time period, from 6 to 100 MYA (Fig. 8).

#### HERVIPADP (HERVIP, HERVADP; taxorder 10400)

The similarity to the avian gammaretrovirus ChiRv1 [42, 60] (Fig. 4) indicates that this is a relatively old group. However, the LTR divergence ranged from 10 to 19 %, compatible with integration 30–40 MYA, Fig. 8. The ERVPB1 envelope [61, 62] clustered with HERVIP Env from this supergroup (Figs. 6,  7).

#### MER50like (MER50, MER57,MER84; taxorder 10500)

MER83 is highly related to MER84 and was not classified as a separate group. The LTR divergence ranged from 17–32 %. Envelopes from ERVV1 and ERVV2 [61, 62] clustered with MER50 Env (Figs. 6,  7).

#### HERVHF (HERVH, HERVH48, HERVFA, HERVFB, HERVFC, LTR46; taxorder 10600)

HERVH is the largest HERV group (1093 chains). Among the two-zinc finger HERVs, HERVFA was most related to MER66 and LTR46, HERVFB most related to HERVH48 and HERVFC most related to ERV3-MacERV6 (rhesus), ERV3-1_CJal and ERV3-2_CJal (both from marmoset). Some of them are shown in Fig. 4. LTR19 is difficult to classify. It was provisionally placed in this supergroup. The LTR divergence distribution was wide (1–12 %), indicating both recent and ancient integrations. HERVFC had several ORFs (Tables 5 and 6) and the lowest LTR divergence (average 3 %, Additional file 5: Table S5-2) of the supergroup.

#### HERVFRDLIKE (HERVFRD, PABL, HERV1ARTIODACT, HERV4, PRIMA41, MER66, LTR39, PRIMLTR79; taxorder 10700)

HERVFRD is the origin of the envelope gene Syncytin-2 [59]. HERV4 is highly similar to HERVFRD.

The LTR divergence ranged from 13 to 36 % (Additional file 5: Table S5-2).

The HERV1_ARTIODACT group (introduced here) contains chains which scored highest in their RepSimage with RM entities ERV1-3_Ssc, ERV1-3_Bt-i, ERV1_cow, MER70-int and LTR35. Ssc here means *Sus scrofa* (pig) and Bt *Bos taurus* (cow). They probably derive from retroviruses which invaded both primates and artiodactyls.

#### HEPSI (HEPSI1-4, MER34, HERV24, PRIMA4, MER4, MER65, MER89; taxorder 10800)

The LTR divergence distribution ranged from 13 to 31 %, indicating a relatively high age.

The cladograms based on Pol amino acids and nucleotides of the whole chains showed the new HEPSI (Human Epsilon) groups HEPSI1-HEPSI4 as consensus sequences clustering with the exogenous Epsilonretrovirus Walleye dermal sarcoma virus (WDSV) (Fig. 4). The existence of epsilonlike sequences in primate genomes was earlier reported by Oja et al. [55] and by Tarlinton’ s group [63]. The epsilon-like sequences (“HEPSI”) border to some RepBase defined entities: PRIMA4 and HERV24 were related to HEPSI2. MER65, MER89 and MER34 were related to each other, and to the HEPSIs sensu stricto. HEPSI3 was most similar to HEPSI2 (cf. trees in Figs. 4, 5, 6), but also clustered with MER4 chains (termed MER4I in RepBase). The HEPSI groups are further discussed in Additional file 2: Section S5.2.2.8.

#### HUERSP (HUERSP1-3, MER52, LTR25; taxorder 10900)

It is one of the oldest groups (Fig. 8). The LTR divergence distribution was 18 to 32 %).

### Class II (Betaretrovirus-like)

#### HML supergroup (HML1-10; taxorder 20000)

The HML groups are presented elsewhere [36, 64]. Their putein and nucleic acid consensuses gave similar trees (Figs. 3, 4, 5, 6, 7, and Additional file 2: Fig S2). The origin of the HML groups is uncertain, but we found that Pol from the MysTr betaretroviruslike sequences of marsh rice rats clustered close to HML5 and HML6, the two oldest HML groups [65], LTR divergence (8–23 %, Additional file 5: Table S5-2). It is reasonable to assume that they had a common origin.

HML2 had at least two LTR divergence peaks, one intermediate (15 % divergence) and one low (1 % divergence), indicating at least two waves of expansion (Fig. 8). Some HML2 chains are the most intact of all HERVs. They feature prominently among HERVs with ORFs (Tables 5 and 6). Thirteen HML2 chains had a very low LTR divergence (<1 %, cf [15, 66, 67]).

### Class III HERV

#### HSERVIII supergroup (HERVL, HERVL32, HERVL66, HERVS, MaLR; taxorder 30000)

Their LTR divergence was intermediate to high (13–42 %). An ancient origin was also indicated by the AutoFrame hits. HERVL was most similar to ERV3-1 from hyrax, tenrec, armadillo, alligator and turtle (some shown in the trees of Figs. 4 and  6). HERVL66 was close to HERVL and to ERV3-1 from wallaby. HERVL32 was closest to LTR57 and ERV3-5 from horse.

Of special note is that HERVS here was found to have a Class I envelope related to the envelopes of PRIMA41, PABL and HERV1_ARTIODACT. The envelopes of HERVL32 and HERVL66 also clustered with Class I elements. Their Env sequences were similar to Env of HEPSI chains. In contrast to most HERVL, the HERVS, HERVL32 and HERVL66 chains did not have dUTPase sequences in the 3′ end of *pol*.

Both HERVL and HERVS were homogeneous groups. ReTe was not able to reconstruct their Gag, most likely due to a weakly matching major homology region (MHR), and absence of zinc fingers. A manual Gag reconstruction based on the HERVS and HERVL nucleotide consensuses was therefore made (cf. consensus sequence collection Additional file 3: List S3). As expected, nearly all HERVL had a dUTPase in the C terminus of Pol. ReTe erroneously placed this dUTPase in a predicted Env (cf. the field “dusimscore” in the main table, Additional file 1: Table S1, where a value >0 indicates presence of dUTPase in the Env putein). Interestingly, HERVS had a class I envelope clustering with the envelope from PRIMA41 (cf. [42]), see Figs. 4, 6  7. A HERVL which like HERVS, HERVL32 and HERVL66 lacked a dUTPase was HERVL chain 4244. It branched between HERVL and HERVL32 (Fig. 4) in the Pol tree. Its envelope clustered with MER50LIKE Envelopes (Figs. 6,  7). Although an extensive search was not done, in all instances when an ERV Class III envelope was detected in hg19, RepBase/RepeatMasker or the literature, it clustered with envelopes of a wide variety of Class I chains. However, the original *env* gene of HERVL, if it ever existed [68], remains unidentified.

MaLR (Mammalian apparent LTR-retrotransposon) containing chains were also found. ReTe recognized 31 chains mainly ascribed to MST, MLT or THE in RepeatMasker-based Simages. As described in the S2 list, 28 of them were judged as probably artificial, i.e. ReTe mistakes. Three remained unexplained. One (rvnr 5922) contained a MuERVL Pol-like putein, two (rvnr 4861 and 3058) vaguely Gag-like puteins. These MaLR containing chains are a very small proportion of all human MaLR [38]. A thorough analysis of MaLR is outside of the scope of the present paper.

### Other retrotransposons

#### Uncertain Errantivirus-like proviruses (taxorder 50000)

The two noncanonical sequences (rvnr 1114 and 5484) had retrovirus-like *gag* genes. They were part of two cellular zinc finger genes, ZNF9 and ZNF13 (described in Additional file 2: List S2). They are shown in the Gag tree of Fig. 5. AutoFrame gave errantiviral hits with zinc fingers of some proviruses, like rvnr 1114 and 5484. A few AutoFrame envelope hits were also with Erranti sequences from RM (see list S1). Moreover, a HEPSI2 sequence (rvnr 4152) clustered with the avian errantiviruslike sequence Ovex1 (Pol tree, Fig. 4). Thus there was scattered information regarding the existence of errantilike sequences in the human genome which could not be fully addressed in the present paper.

#### Unclassifiable chains (taxorder 60000)

Sixteen chains could not be classified due to inconclusive Simage patterns, and lack of sufficient taxonomic markers.

#### LTR divergence

As shown in Additional file 5: Table S5-2, the proposed HERV groups yielded widely differing LTR divergences. LTR divergence is not a universal indicator of age since integration [69]. The calculation, using two independently mutating LTRs, requires a clock-like steady rate of point mutation, roughly giving a 0.4 % LTR divergence per million years, see e.g. [70–72] since the integration. In a few instances, we use this simple way to indicate time since integration. However the calculation is only vaguely true. Several factors can influence the divergence. In the 1st million years post integration, gene conversion can diminish the degree of divergence. Indel events post integration can give artificially high divergences. Nevertheless, the structurally intact or relatively intact HML2 and HERVFC elements stand out as examples of probable evolutionarily recent integrations. Figure 8 shows the distribution of LTR divergences for the most abundant HERV groups. The peak of integrational activity seems to have been earliest for HERVL, followed by HUERSP3, HML5, HML6, HERVIP, HERV9, HERV3 (diffuse distribution with no real peak), HERVE, Harlequin, HML3, HERVH and HML2. HML2 had a bimodal distribution, with two peaks, at 20 % and 0–1 % divergence, respectively. The 0–1 % divergence bin contained 13 HML2, 2 HERVH, 1 HERV9, 1 HERVIP, 1 HERV3 and 1 Harlequin. It remains to investigate if all of these integrations really occurred during the last 2.5 million years. When we studied the age of these 19 integrations by searching their flanks in the Chimpanzee genome (separated at least 5 million years from humans) with BLAT in Genome Browser, only 10, all HML2, were not found (data not shown). Thus, a very low LTR divergence can be somewhat misleading as a measure of integrational age. It is striking that some highly degenerated HERVH, actually the majority of HERVH, have a discrepantly low LTR divergence. A possible explanation is that particles encoded by contemporaneous, more intact, “midwife” elements packaged RNAs from defective elements [57]. There are no apparent present-day HERV proviruses which tentatively can be ascribed this function.

#### Frequency of ORFs

ReTe interprets proviral structure and attempts a reconstruction of the original protein, in the form of a “putein”. In case a putein is reconstructed, ReTe estimates the number of shifts and stops for each of the four major genes. Two motif hits, similarity to at least one of the proteins in the template alignment for that protein, and presence of a stop-free stretch of at least 50 amino acids [35] within the estimated length of the gene, is required for starting a putein reconstruction. In our experience, the reconstructed puteins generally recapitulate most of the original protein sequence. This method is however not free from errors. If for example a relatively short unswept secondary nonretroviral repeat is present inside a chain, ReTe will attempt to translate it. Another (smaller) problem is whether the natural stop codon should be counted as a stop or not. We have observed that in many cases, putein reconstruction stops at the “correct” stop codon. Such stops are not counted. But if the program continues beyond the natural stop codon, that codon will be counted as a stop. A further problem is that the “correct” reading frame is sometimes hard to determine. For example, we have observed that in the 3′ ends of HML2 *gag* there might be alternative reading frames (JB, unpublished). Thus, it is reasonable to include near-ORFs of significant length, with 1 shift or 1 stop, in a survey of HERV ORFs (Tables 5 and 6). ORF-containing chains are also discussed in detail in Additional file 2: List S2.

#### Identification of HERVs found by ReTe which have been ascribed significant function

An HERVH at chromosome 8:13309237 which is part of the gene HHLA1, an important regulator of stem cell differentiation, and is strongly upregulated during early embryogenesis [73] is colocalized with the provirus with rvnr 2965. It is defective, lacking full *pro* and *env*. It has 3 stops and 3 shifts in *gag* and a highly mutated *pol* with 13 shifts and 16 stops. This is an example of a highly defective HERV with an important regulatory function.

Rvnr 2256, an HERVE element at 6: 89371970 which is relatively complete, has 4 shifts and 4 stops in *gag*, an open *pro*, 6 shifts and 7 stops in *pol* and 4 shifts and 2 stops in *env*. Yet it is able to encode a tumour antigen, represented by the peptide “ATFLGSLTWK”, immunity to which possibly may cause kidney cancer regression [74]. Likewise, rvnr 4362, a relatively complete HML6 element at 16: 30635509, with multiple stops in all four major genes, was reported to encode a malignant melanoma antigen “MLAVISCAV” from its envelope [75]. The sequence is “MLAVISCEV” in the envputein reconstructed by ReTe. The reason for this difference is unknown. Even highly degenerated HERVs may express pathophysiologically important proteins.

## Discussion

In spite of the great efforts made during the last 30 years, a comprehensive analysis, including classification, of the most intact HERV proviruses present in the human genome is still lacking. Moreover, the main established HERV databases [61, 76] are not maintained and updated. Hence we wanted to identify and characterize the HERV proviruses found in the GRCh37/hg19. It could be an important step to foster novel studies in the HERV field. We used a bioinformatics approach utilizing ReTe. ReTe retrieved 3173 HERVs integrated in one of the latest and most thoroughly made human genome assemblies.

HERV classification was achieved through a multistep procedure, including the novel principle of the Simage analysis. It led to a classification of 3045 (96 %) of the 3173 HERVs. As reported previously, Gamma-like sequences (Class I) were more common than Beta-like (Class II). Alpha-, Delta- or Lentivirus-like proviral sequences were not detected. However, the presence of Epsilon-like elements is notable and deserves a more detailed investigation.

We tried to combine previous HERV groups from literature and the comprehensive Repbase classification. RepBase (and RepeatMasker) is biased towards LTR classification, our system towards the inner proviral portions, primarily Pol. In many cases it was possible to merge the two systems. In other cases, like the complex MER4I group and HERVI/HERVIP distinction it could be problematic. In most cases, the high identities to HERV consensuses within the groups justify the chosen groups. As shown in Additional file 2: List S2, there exist RepBase HERV entities which were not detected in our ReTe-based search. Most of those are highly degenerate, giving a low chainscore of ReTe. It is likely that an even more comprehensive analysis, maybe including other primate genomes, could clarify the classification of such elements.

Our final HERV classification into 39 canonical groups partially overlaps with previously reported HERV groups [28, 56, 61, 76]. Possibly, some observed differences could be explained with the methodologies applied for both the identification and the classification of HERV sequences. Indeed, our current focus was to enumerate the members of each HERV group. We did not attempt to enumerate solo-LTRs. Moreover, the complex phylogenetic analysis, mainly based on Simage, allowed a better definition of “borderline” sequences between highly related groups e.g. HERV9 and HERV30, to introduce new HEPSI1-4 (human Epsilon) groups within the Class I HERVs (cf. [63]) and to identify short stretches of Errantivirus-like similarity within the Pol regions of some HERV proviruses (out of scope for this paper). Two Gag-containing chains, which encode zinc finger regulatory proteins, had a vague similarity to Errantiviruses (classified as “uncertain errantilike” [77].

Simage analysis also contributed to determine the presence of a high number of mosaic HERV structures, some of which may be “true recombinants”, with a level of detail not previously appreciated. In a minor portion of chains Simage analysis suggested ReTe artefacts, where dissimilar but proximal proviral fragments were artificially joined by the ReTe algorithm. Heterogeneity could occur because of imperfections in classification, making highly related sequences look unrelated. This situation is most likely to occur in highly conserved portions. The RepBase/RepeatMasker classification, used in the RMRef library, has overlaps between ERV clades. In order to recognize recombinants one must tackle this problem. Some of the “canonical” HERVs may be recombinants themselves. For example, some of the Harlequin chains behave as canonical, with a reiterated recombination pattern. Difficult issues are distinction of HERVI from HERV3 and HERV1, HERV9 from HERVW, the HUERSPs from MER52 and the so-called MER4 complex. This error was minimized by visual inspection of Simages. Features which then could be looked for were classification of 5′ and 3′ LTRs, and sense. An accidental joining of two unrelated fragments is unlikely to result in 5′ and 3′ LTRs belonging to the same HERV group. In the absence of a selection bias, a secondary integration would be expected to be in antisense orientation in 50 % of cases, and to provide an additional unrelated LTR.

The most extensive descriptions of HERV recombination events refers to the homologous recombination that is responsible for the solo-LTR formation [78–80] or for the documented intra-chromosomal recombination between two homologous HERV15 sequences (Repbase identifier for RRHERVI, here included in the HERVI group) located on chromosome Y (rvnr 5093 and 5106) that is responsible for male infertility due to the Azoospermia factor a (AZFa) microdeletion [25]. Nonetheless, an overall description and enumeration of “mosaicisms” occurring within HERV internal structures was not listed previously. Simages allowed both detection of mosaic forms that complicate sequence-based HERV classification and tracing the source of such heterogeneity. We present evidence that some of the noncanonical mosaic chains actually have been infectious recombinants capable of reintegration. Most of such putative recombinant forms seem to have occurred between related retroviruses, either belonging to Class I or Class II. A notable recombination seems to have given the Class III HERVS a Class I PRIMA41-like Env. A similar interclass recombination was earlier noted by us and others in avian ERVs, where the Avibeta2 clade (Class II) was found to have an Avigamma1 (Class I) envelope [42, 81]. HERVS Pol clusters with the AviERVIII consensus, an avian ERVL (termed GGERVL18 in Repbase), and PRIMA41 Pol with Avigamma1 (data not shown). The frequent similarities between envelopes belonging to different groups, supergroups and classes show that acquisition of new envelope (“*env* snatching”) is a widespread phenomenon among the retroviruses which became endogenized in the human lineage. Both acquisition and loss of envelope can lead to increased fitness. Acquisition of a new envelope can give access to new host cells. Loss of envelope may mean loss of extracellular replication and can enhance intragenomic spread [68].

Thus, HERVs show signs of the same recombination phenomena between replication competent retroviruses and ERVs as have been observed in mice [82] and cats [83–86]. These must have occurred in the distant past. Such recombination depends on many factors; access to cells with high expression levels, intactness of frames, number of cross-packaging and reverse transcription events, etc. The only prevalent extant human exogenous retroviruses, HIV and HTLV, are sufficiently dissimilar from HERVs to make this an unlikely scenario.

Some of the noncanonical mosaic chains may have had replicative potential. Although definite proof for such a phenomenon cannot be obtained from this bioinformatic study, the circumstantial evidence presented here indicates a widespread occurrence of such recombinants. Most of such putative recombinant forms seem to have occurred between related retroviruses, either belonging to Class I or Class II.

Among the Class I ERVs, the Harlequin mosaic pattern of HERVE-HERVW-HERVIP-HERVE stood out as being most frequent. However, Harlequin seems to be the tip of an iceberg of recombinant candidates with a smaller number of originating sequence donors. Among Class II ERVs, groups HML1, HML2 and HML3 were most frequently involved in probable recombinations. The HML groups are clearly separated at the nucleotide level, but sometimes overlap if studied at the protein level. This makes the distinction of recombinants complicated. However, the patterns of putative recombination are so consistent and clearly different from the canonical HMLs that we favour that they are the result of recombination. Retroviral recombination is most frequently caused by copackaging and template switching during reverse transcription. The particle harbouring the recombinant genome then must infect a germ line cell and get genetically fixed in order to be registered as a HERV.

Envelope subgroup diversity was especially pronounced in Class I HERVs, but occurred in all three classes. As described by [58] and in this paper, Harlequin proviruses are mosaics containing HERVE, ERV9/HERVW, HERVI and HERVIP portions. Env was obviously part of this diversity. Judging from Harlequin Simages, many of them have a rather intact HERVE Env. Hypothetically, a functional aspect of the large number of otherwise defective Harlequin and Harlequin-related proviruses could then be to provide envelopes of varying function, e.g. in *trans*. Regarding Class III ERVs, it is remarkable that, although an extensive search was not done, in all instances where a credible Env was detected, the Env was of Class I, indicating that “*env* snatching” is an especially common strategy among Class III ERVs.

## Conclusions

The study of HERVs represents an intriguing challenge. HERVs are fragmented, deteriorated, remnants of their exogenous retroviral ancestors. It is now clear that they also can become essential genetic components with many physiological functions. However, after 30 years of extensive research in this field, some basic questions regarding the HERV classification, structure and role in modulating human pathophysiology still remain. An advance in HERV knowledge must include a clear definition of the type, exact number and position of these retroviral sequences. We here attempted a detailed description of HERVs and their sometimes mosaic structure. The Simage technique proved to be useful for solving some mysteries of HERV classification which have plagued the field for a long time, highlighting the central role of recombination during retroviral evolution.

## Methods

### Human genome assembly (GRCh37/hg19)

The February 2009 assembly GRCh37/hg19, released by the Genome Reference Consortium [38], is the human reference sequence used to perform the HERV identification. The full haploid set (22 + X + Y) of chromosomes sequences was downloaded, as FASTA files (chr*.fa.gz), via the UCSC Genome browser (http://genome.ucsc.edu/) and the file storage was set up at the CRS4 Institute on an Intel based machine.

### Retroviral reference sequence collections

The different data sets of retroviral consensus and reference sequences, used to perform the HERV multistep classification procedure were obtained as follows:

1. An exhaustive data set of both exogenous and endogenous retroviral sequences (RvRef) was collected by Jonas Blomberg from literature with the principle of precedence for the first publication of the sequence. Briefly, the RVRef collection contains selected, essentially complete, proviruses from vertebrates found by running 40 different genome assemblies (partly described in, and given as supplementary material in [36, 40, 42]). It also contains 163 sequences collected from the HERV literature of the last 30 years. Some of these sequences are also part of ReTe´s preliminary classification system (found in the table forretrotector.txt). A few Errantiviruses and Pseudoviruses were also included;
2. A set of 9 HML (HML) consensus sequences, generated for the HERVK (HML1-HML9) group [64];
3. The entire Repbase Update [33], a database of repetitive DNA elements was downloaded from: http://www.girinst.org/repbase/update/index.html;
4. The “LTR” subset from the entire Repeatmasker (RMRef) collection of vertebrate repeats (release of May 2012) [34, 43] was downloaded from: http://www.repeatmasker.org.

### RetroTector

The human genome GRCh37/hg19 was examined for the presence of HERV proviral sequences using ReTe (version 1.01), a program package developed for the recognition of endogenous retroviral sequences in vertebrate genomes [35]. ReTe is mainly based on the principle of “fragment threading”, an algorithm that searches for the presence of conserved motif hits (from known exogenous and endogenous retroviral proteins) and from these attempts to reconstruct “chains” satisfying distance constraints, indicating proviral sequences. Further, it attempts to suggest putative retroviral protein sequences (“puteins”) and to estimate the original longest ORF (open reading frame) for each putein. A preliminary classification of the identified chains based on a ReTe viral genus assignment, and a chainscore that identifies the degreee of chain intactness are also given. The data generated during the analysis are stored in a MySQL database. They were further processed by Visual Foxpro programs written by JB. The results (MySQL and.dbf tables) could be visualized through a user-friendly interface and extracted, as Excel tables, for further investigations.

ReTe was set up at the CRS4 Institute on a computing cluster, an Intel based machine with 4 Xeon processors with 6 2.66 GHz cores, 256 Gb of RAM with an estimated execution time for the GRCh37/hg19 of 1–2 days.

Two files, hg19_HERV_master_20150608_for_publ.dbf, and hg19_HERV_master_20150608_for_publ.fpt, containing the entire dataset of 3173 chains were uploaded as a .zip file to Labarchives, BMC edition. An Xbase application like Visual Foxpro is required for reading the table. They can be reached via the link

https://mynotebook.labarchives.com/share_attachment/hg19_ReTe/MjMuNHw5NTI4MS8xOC00L1RyZWVOb2RlLzI0MzE2NDk0ODV8NTkuNA== and DOI 10.6070/H4QZ27ZT.

### Detection of taxonomic markers

#### PBS

For a comparative quality control of the HERV PBS sequences identified and scored by RetroTector (the first method), all human tRNA sequences were downloaded from the Leipzig tRNA database [87] at http://trna.bioinf.uni-leipzig.de/DataOutput/. The 3′ ends containing 18 nucleotides complementary to retroviral PBS motifs were stored. ReTe PBS sequences were matched, accepting only exact matches, against the Leipzig derived tRNA sequences (second method). The third method tested for matches between ReTe PBS motifs and Leipzig derived sequences, with up to two mismatches. Additional file 1: Table S1 contains all PBS sequences detected by ReTe (first method; fields PBsscore, PBSseqrete and PBStype), the exactly matching Leipzig sequences (second method; fields BestPBS and BestPBScod), and those matching a Leipzig sequence with 1 or 2 mismatches (third method; fields LikelyPSeq, LikelyPBS and LikelyPcod). A compilation of the results, in the form of a general hg19 PBS translation table which covers most of the encountered HERV PBS motifs is given in the supplementary material (Additional file 6: Table S6). It covers many HERV PBS motifs which were not encountered in the Leipzig database.

ReTe uses a heuristic algorithm where the predicted PBS sequence (18 nt, nearly always starting with “TGG”) is matched against a table of published retroviral PBS sequences (occurring in the Table motifs.txt, distributed with ReTe). It scores the closeness of fit where perfect match scores 200 and a fit with more than 4 mismatches scores 0. Yet, if the basic criterion of a TGG start is fulfilled, the closest PBS alternative is given. Thus, the type of PBS scoring 0 is uncertain. ReTe identified a PBS type in 2132 chains. Of these, 1401 had a PBS score >0. Leipzig tRNA database (URL) had 844 exact matches. Allowing two mismatches there were 562 additionally, i.e. 1406 totally. The concordance of PBS determination between ReTe PBS motifs scoring >0 and Leipzig perfect and imperfect hits with two mismatches, was 1108 of 1401 chains (79 %). When PBS motifs with perfect Leipzig matches were compared against ReTe matches scoring >0, nearly all (748 of 844 chains, 89 %) gave the same result. When PBS motifs with perfect Leipzig matches were compared against perfect (scoring 200) ReTe matches, nearly all (52 of 60 chains, 87 %) gave the same result.

Scrutinizing the discrepancies (cf. Additional file 6: List S6) revealed a few remarkable differences: 4 chains, all classified as HERV9, had the PBS “ttggcgaccacgaaggga”, labelled as “R”. In the Leipzig database a sequence shifted one nucleotide, “tggcgaccacgaagggac”, was labelled “W” (CCA). The PBS sequence used in ReTe was derived from the paper of LaMantia et al. describing the ERV9 provirus [88]. We suggest that this sequence “ttggcgaccacgaaggga” was mistakenly shifted one nucleotide. The canonical tryptophan PBS “tggcgaccacgaagggac” is more probable. Thus the PBS motifs of some HERV9 probably should be labelled “W” instead of “R”. This was not confined to the 4 mentioned high-scoring chains. A total of 59 chains with PBSscore >0, given the PBS “R” by ReTe, were partially identical to a Leipzig “W” (CCA) PBS, with 1–2 mismatches. On the other hand, two chains with a “W” ReTe PBS were partially identical to a Leipzig “R” PBS, with 1–2 mismatches. Thus, distinction of an “R” PBS from a “W” PBS can be problematic.

The distinction of some other PBS motifs was also difficult. In 13 cases, ReTe “T” for tggtgacccagatgggat, tggaggcccatctgggat, tgggggactacctggaat, tgggggcccacccaggat and tgggggcccacctgggat were just 1-2 mismaches away from Leipzig “R” (ACG), Leipzig “P” (AGG) or “C” (GCA). Three low-scoring ReTe “F” (tggtgccgcaactcggat x2 and tggtgccgtgactcggaa) were two mismatches apart from a Leipzig “H” (GTG). Two low-scoring ReTe “G” (tggtgcagtgactgggat) and “L” (tggtgccaggactcggat) were two mismatches apart from a Leipzig “H” (GTG). A ReTe “Q” (tggaggtcccagtgagaa) was two mismatches apart from a Leipzig “T” (TGT). Thus, in proportionally few cases PBS motifs were hard to unequivocally assign to a certain tRNA. In the course of working with avian ERV PBS motifs [42] we (JB) observed that bird tRNAs sometimes gave a better fit than mammalian tRNAs (unpublished). It is likely that ERV PBS motifs reflect the tRNA status during infective stage of the retrovirus. Most HERVs integrated 10–100 million years ago. One can therefore discuss which subset of tRNAs are most appropriate to use for PBS identification. In this paper we used the “Homo” subset of the Leipzig tRNA database. This provided a credible PBS identification in over half of the PBS sequences detected by ReTe. It is probable that a more thorough investigation, with tRNAs from other species, could achieve a higher identification coverage. However, it is out of scope for this paper.

#### Other markers

Nucleotide bias, number of zinc fingers in Gag, predominant frame shift strategy, dUTPase in protease, Gpatch in protease, dUTPase in integrase, and Chromodomain and GPYF motif in integrase were detected as described [35, 36, 40, 42, 50].

The number of zinc fingers in Gag were calculated from ReTe zinc finger motif hits.

Translational frame shifts were estimated from the reading frames recorded in ReTe for motif hits occurring near the *gag*/*pro* and *pro*/*pol* borders, respectively.

dUTPase in Pro was detected by ReTe using proper motifs.

Gpatch in Pro was detected by a program written by JB, using described features [93].

dUTPase in the C terminus of Pol and Env was detected by searching with BLASTP with a collection of dUTPase sequences in the 5′terminal half of all three forward reading frames for each chain.

GPY/F_Chromodomain motifs were detected by a program which used ReTe hits IN5 and IN6, then looking for further chromodomain [94] and GPY/F [95] features.

### Similarity image (Simage) analysis

In programs written by JB (unpublished) chain DNA identified by ReTe was divided into 20ths. Retroviral target sequences (regardless of length) were handled in two ways; either the target sequence with matches after BLASTing marked with upper case was sliced into twentieths, or the targets were sliced into tenths before BLASTing and upper case match marking, then halved to yield twentieths. In both cases, the proportion of upper case nucleotides (or amino acids) was recorded. Each target was BLASTed against the reference and consensus sequences collections (RvRef, RMRef, HML, Con1 and Con2) as queries. Each 20th was then BLASTed (BLASTN, with word length 7, i.e. relatively nonstringent conditions) onto a table of reference sequences (RvRef, RMRef, HML, con1 and con2), listing the highest scoring hit. A one-letter symbol was allotted to the sequence in the collection which gave this hit. The number of positions in a target twentieth that matched the search sequence was used to generate the Simage score with the maximum of similarity (all positions matched) set to 9. The other values (from 9 to 0) were calculated from to the number of matching positions relative to this maximum in the given twentieth. Simages allow a quick overview of the homogeneity of the sequence. HERV sequences for which more than ten twentieths derived from the same or a highly similar reference or consensus sequence and where less than four twentieths were “0” (absence of similarity to a reference sequence) were considered as canonical sequences. In cases where RvRef and RMRef indicated a different canonical reference sequence, preference was given to the RvRef sequences. This was because the RvRef sequences can be traced to numerous HERV publications. They are therefore important for maintenance of the collected knowledge on HERVs. However, the analysis with the RMRef system was performed simultaneously, so it was always possible to compare the two results. The same mechanism was used for proteins (used in Autoframe search, see below). In this paper, Simages were derived from BLASTing of nucleotide 20ths to the RepeatMasker library of May 2012, the retroviral reference sequences collected from literature, a collection of HML sequences provided by V Blikstad and two sets of hg19 consensus sequences (Con1 and Con2) derived from the present work. Con1 resulted from early work in this project. It contained 43 consensus nucleotide sequences (not shown) derived from “chaindna” (the ReTe representation of the proviral DNA) [35]. Con2 contained the final 39 consensus and 5 additional best representative nucleotide sequences derived from chaindnarm (chaindna which went through an additional round of repeat masking) established in this paper. The sense/anti-sense orientation of each twentieth, and the position of the twentieth within a ReTe recognized and translated gene (shown for Con2 only; 5′LTR-”5”, *gag*-”G”, *pro*-”R”, *pol*-”P”, *env*-”E”, 3′LTR-”3”) were also determined. The results are shown in Additional file 1: Table S1 in fields “Refsimage”, “RMsimage”, “HMLsimage”, “Con1simage”, “Con2simage” and “Con2simgtg”, respectively.

### Autoframe matching of ORFs

In this program, (written by JB), out of the RMRef library, DNA from 17500 LTR retrotransposons were translated in all three forward frames. All frames without stops for at least 130 amino acids (up to 15 frames per retrotransposon DNA) were BLASTed against the Gag, Pro, Pol and Env puteins found by ReTe. Results were shown as Simages (fields Gagsimage etc. in Additional file 1: Table S1). For each ReTe chain, the two highest scoring ORFs (Gagtwomost, Protwomost, Poltwomost and Envtwomost in Additional file 1: Table S1) were calculated. This program allowed the use of RMRef nucleotide sequences for protein matching. It was valuable because there are no easily available protein sequences for many retrotransposons. Protein matching is more sensitive than nucleotide matching, and thus could be used over a wide range of vertebrate retrotransposons for classification, phylogenetic inference and detection of protein aberrations, like the recombinatorial origin of envelope genes.

### Envelope subgrouping

Envelope subgrouping was first based on Autoframe hits and ISD heterogeneity

The Autoframe hits for Env puteins sometimes varied within a HERV group. This could be due to a variable defectiveness of the Env putein, or to variation in the original Env protein. An initial, automatic, classification was based on the Autoframe hits. The most common hit for the HERV group was named a, the next most common b, etc. ISD variants were detected by first retrieving TM2 hits (which contains hits from the immunosuppressive domain-ISD) from the chain field (in the “hg19_HERV_master_20150608_for_publ.dbf” table, see above). ISD was also detected using an algorithm (created by JB, with the name “henzyscore”) for detecting the domains SU-cysteine, SU-TM cleavage site, ISD, TM-cysteine and transmembrane, based on rules for retroviral envelope proteins primarily defined by Andrew Cunningham and Jamie Henzy [89–92]. An Env score based on this algorithm was stored in the field “envhpoints” of Additional file 1: Table S1. A 23-amino acid stretch containing ISD was stored in the field “isdextsh”, see Additional file 1: Table S1. Some ISDs were identified manually, and entered into the same field. ISDs were aligned by ClustalW. ISD variants with more than 5 amino acid mismatches to surrounding ISDs in the alignment were given sequential numbers; 1,2,3 etc.

During the work with envelope puteins, we noted that some contained homopolymeric (“KKKKK”, “FFFFF” and “YIYIYI”), long hydrophobic stretches and a low number of predicted N-glycosylation sites, abnormal for an envelope glycoprotein. A program for quality control of envelope puteins (“EnvQual”, yielding “envqpoints” in Additional file 1: Table S1) where these features are enumerated was therefore constructed. A cutoff of 6 envqpoints was used for excluding Env puteins which may have been artefactual (Additional file 2: Fig S3). In this way we created a provisional classification item, “envgroup1”, containing group name, Autoframe subgroup and ISD subgroup, e.g.”HERVH a 1”. Selected envputeins were used for calculation of initial env and ISD consensuses. These consensuses were exported to a FASTA file and aligned by Muscle. A maximum likelihood tree of Env puteins was then made. In this tree (not shown) it was noted that some Env subgroup consensus sequences clustered rather narrowly together. Others were widely separated from the main HERV group. Simplified new Env subgroup consensuses (labelled A, B, C, etc.) which additionally used the relationships found in the tree were therefore calculated. They are shown in Additional file 1: Table S1 as “envgroup2” (see also Additional file 4: List S4, Figs. 6, 7 and Tables 5 and 6).

### HERV classification

The MEGA software (version 5.2) [96] was used for sequence alignment and phylogenetic trees inference. Multiple alignments were performed using both Muscle and ClustalW with default settings. The neighbor-joining trees were based both on Pol amino acid and nucleotide sequences, and bootstrap analysis was carried out with 1000 replicates.

#### The final HERV classification was aided by Simage analysis and taxonomic markers

Simages where more than half of the best matching twentieths derived from the same reference sequence, and less than four twentieths were “0”, that means absence of similarity to a reference sequence or to a closely related reference sequence, were considered unambiguous (canonical) representatives of the most frequently matching reference sequence. In cases where both RvRef and RM indicated an unambiguous reference sequence, preference was given to the RvRef sequence. Simages were created by BLASTing, as described above. A final set of 39 HERV canonical consensus sequences, plus sequences from 26 groups, either canonical ones represented by a single chain, or “best representatives” from noncanonical chains, with the most intact Gag, Pro, Pol and Env ORFs within the group, was obtained. The consensus sequences were generated through ClustalW alignments of both whole nucleotide chains and puteins (Gag, Pro, Pol and Env) within each HERV classified group (clade) (Additional file 2: List S2.5). The degree of heterogeneity of the groups, that is the portion of positions not identical in more than 50 % of members (heterogeneity index), the portion of gaps in the alignment, and the average of both “intermember identity within the group” (WIGI) and “identity to consensus within the group” (ITC) were calculated (see Additional file 5: Table S5-3).

## Additional files

10.1186/s12977-015-0232-y Excel table generated from the master.dbf table. Field names that need an explanation, and are not explained in the main text, are; “Subgenes”: Presence of motif hits belonging to portions of LTRs and the four major genes (from ReTe); “Chainscore”: Weighted sum of motif hits calculated by ReTe, ranging from 300 to 2500; “Breaks”: ReTe detected two proviral portions seemingly belonging together but separated by a longer than normal distance, therefore disregarded the intervening sequence, its start and stop shown in this field; the PBS fields are described in the main text; “Gagscore” (as well as scores for the other three major genes) shows the degree of fit of the putein to the reference proteins in the best fitting genus-specific alignment included in ReTe; “Tperc”, “Aperc” etc.: percentage of each nucleotide in the chain (from ReTe); “Bestrefrv”: Best fitting nucleotide sequence out of a set of reference retroviral nucleic acid sequences, together with  % identity and total length of the reference sequence (from ReTe); “Polclass”: best fitting reference Pol amino acid sequence, with the score of the reference sequence to itself/score of the query sequence to the reference sequence (from ReTe); The five “idpc” fields show the  % identity to group consensus for dna and the four major proteins; the “Envidpc2” field shows  % identity to the Env subgroup2 consensus; “Repantist” shows antisense portions of a repeatmasker Simage; the five Simage field collections show nucleic acid Simages for repeatmasker (rep), HML (hml), reference sequence collection (ref), first (con1) and second [con2, this paper (Additional file 3: list S3), including best representative (bre) noncanonical or single canonical sequences] consensus collections, respectively. For each Simage are also shown a quantification (-simgst), a list (-simgls) explaining the letter symbols, and the sense relative to the chain sense (-simgse); The con2 set also contains the field “con2simgtg”, which depicts the presence of LTR (5 and 3), Gag (G), Pro (R), Pol (P) and Env (E) in each chain twentieth; The “twomost” fields show the two most frequent (with number of hits out of twenty) AutoFrame hits per the four major genes; The ensuing Simages show the distribution of AutoFrame hits per putein for each gene followed by a hit list and a quantification like for the nucleotide Simages; “Isd” is the “immunosuppressive domain” calculated from the envelope evaluation program henzyscore or identified manually; “Envhpoints” is the score from henzyscore; “Envgroup2” shows the envelope subgroup (like “HERVT_A”, in the main text often shown as “hervta”); “Envqscore” is the output from the envelope quality control program EnvQual.
10.1186/s12977-015-0232-y Supplementary figures, detailed discussions and detailed description of HERV groups.
10.1186/s12977-015-0232-y HERV group consensus and best representative sequences. Its contents are explained in the beginning of the list.
10.1186/s12977-015-0232-y HERV Env subgroup consensus sequences. The FASTA names contain 1. taxorder, 2. subgroup, 3. number of members in the subgroup, 4. 23-amino acid consensus immunosuppressive domain, 5. if present, the most highly related envelope protein from maximum likelihood (Fig. 6,  7) phylograms, and 6. the bootstrap value of the common branch of these envelope proteins.
10.1186/s12977-015-0232-y HERV group Excel table with 1. Nucleotide frequencies and marked biases, 2. LTR divergence and 3. Consensus statistics.
10.1186/s12977-015-0232-y Translation table for HERV PBS sequences, taken from ReTe and hg19. The derivation and structure of this table is described in Methods.
