# Supplementary material for: Classification and characterization of human endogenous retroviruses; mosaic forms are common
Source: Retrovirology. 2016 Jan 22;13:7. doi: 10.1186/s12977-015-0232-y (PMC4724089; doi:10.1186/s12977-015-0232-y)
Supplement: Supplementary file 2 — 10.1186/s12977-015-0232-y Supplementary figures, detailed discussions and detailed description of HERV groups. [file 12977_2015_232_MOESM2_ESM.docx]

**Supplementary list S2, Supplementary figures, detailed discussions, and details regarding the HERV groups.**

1. **Supplementary figures**

**Figure S1**

**
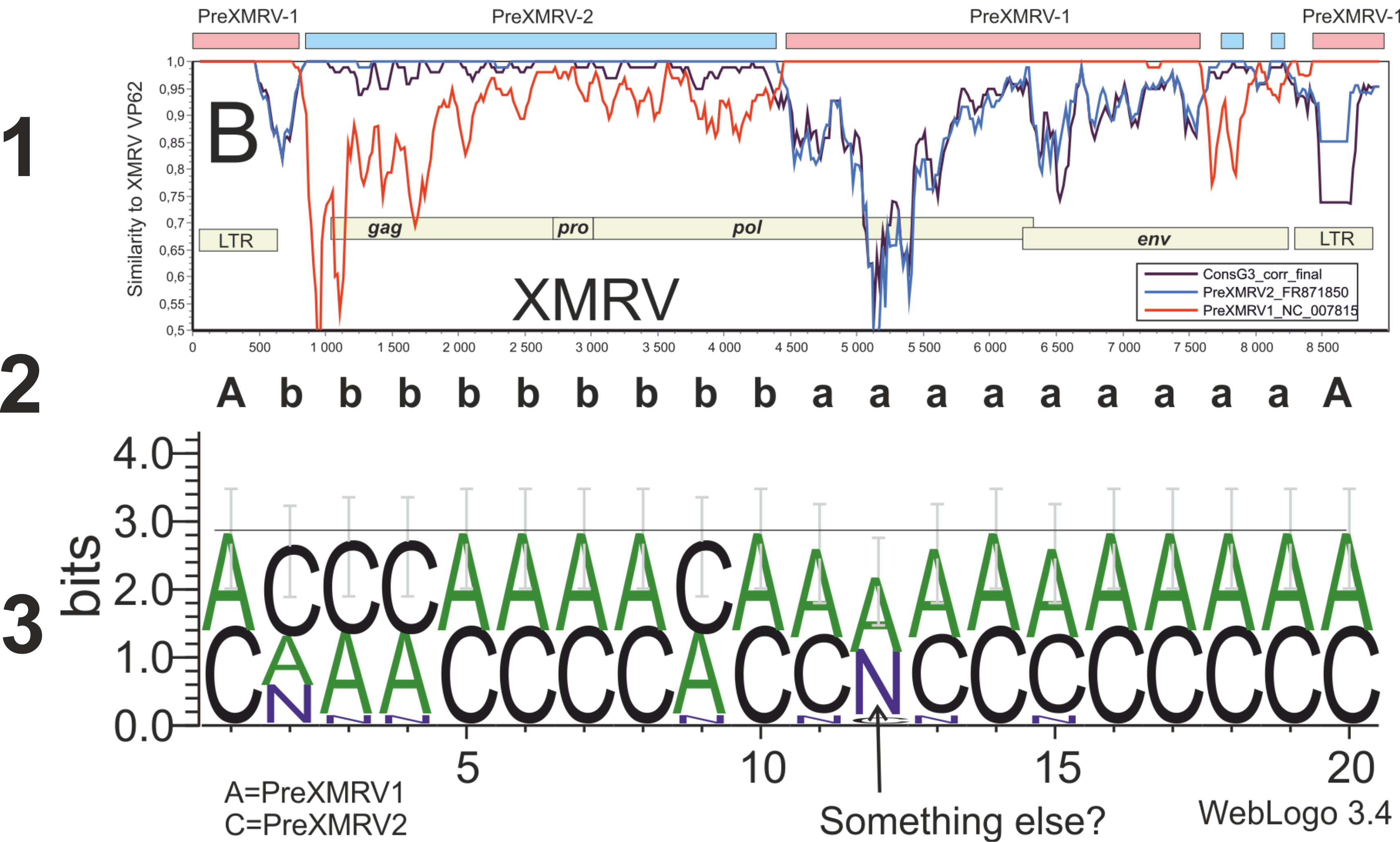
**

**Fig. S1.** Demonstration of the Simage principle. The Simage was here turned into a WebLogo. A Simplot XMRV analysis (1) is reported and compared with Simage analysis (2) and its WebLogo (3) representation. A: LTR-PreXMRV1; a: Pre-XMRV1; b: Pre-XMRV2. In two positions in the Simage, the score of PreXMRV1 and PreXMRV2 do not add up fully, implicating that a minor contribution from other sequences (“N”) may have occurred at these positions. The upper portion of this figure is identical to a portion of the figure in (1).

**Figure S2**

**
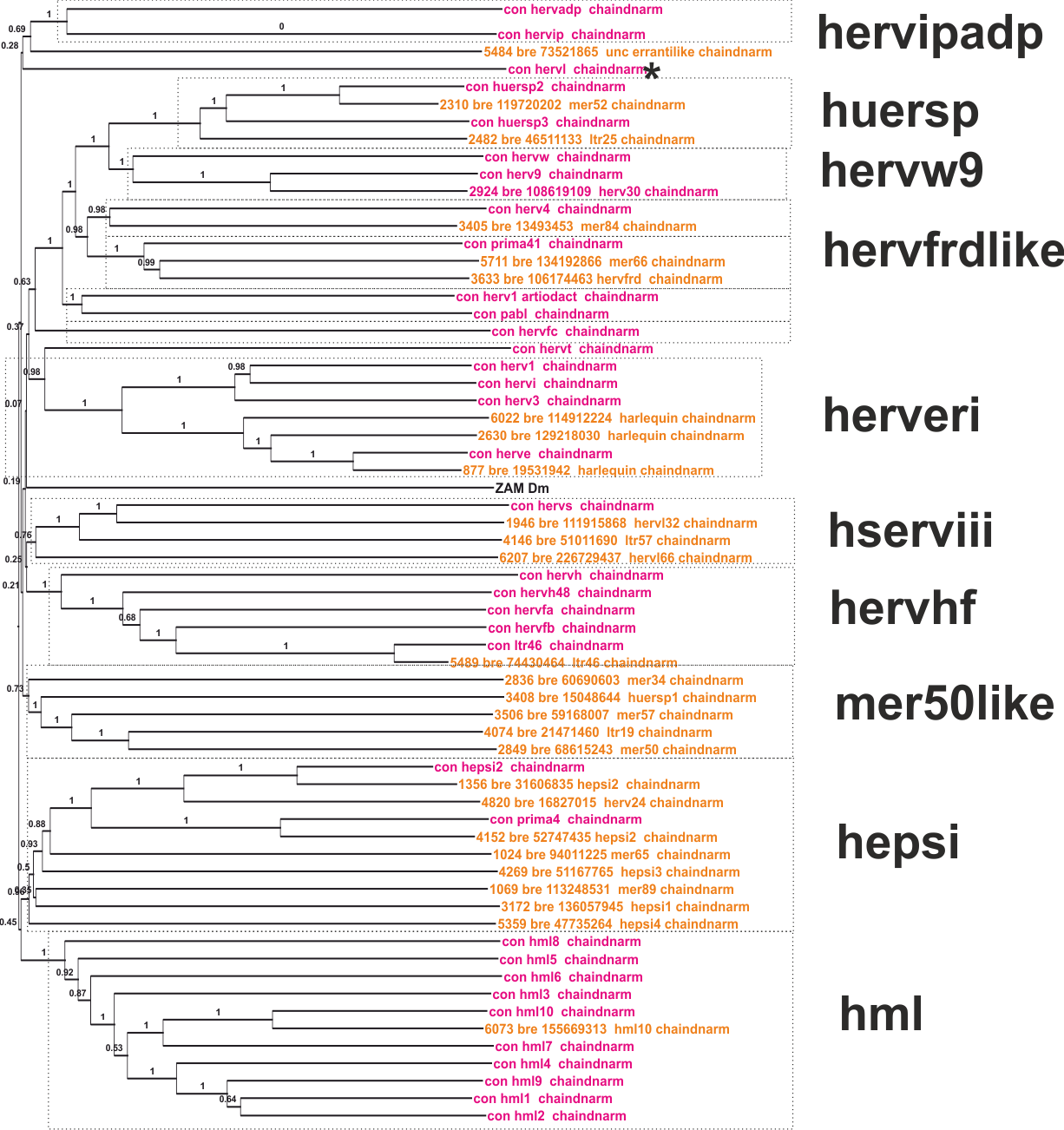
**

**Fig. S2.** Unrooted tree based on nucleic acid (chaindnarm, swept for repetitive DNA), together with reference retroviral sequences. Repeat-masked chainDNA was aligned using Multalin. A Maximum Likelihood tree was then created using Mega. Consensus (magenta) and best representative (brown) sequences were included.

**
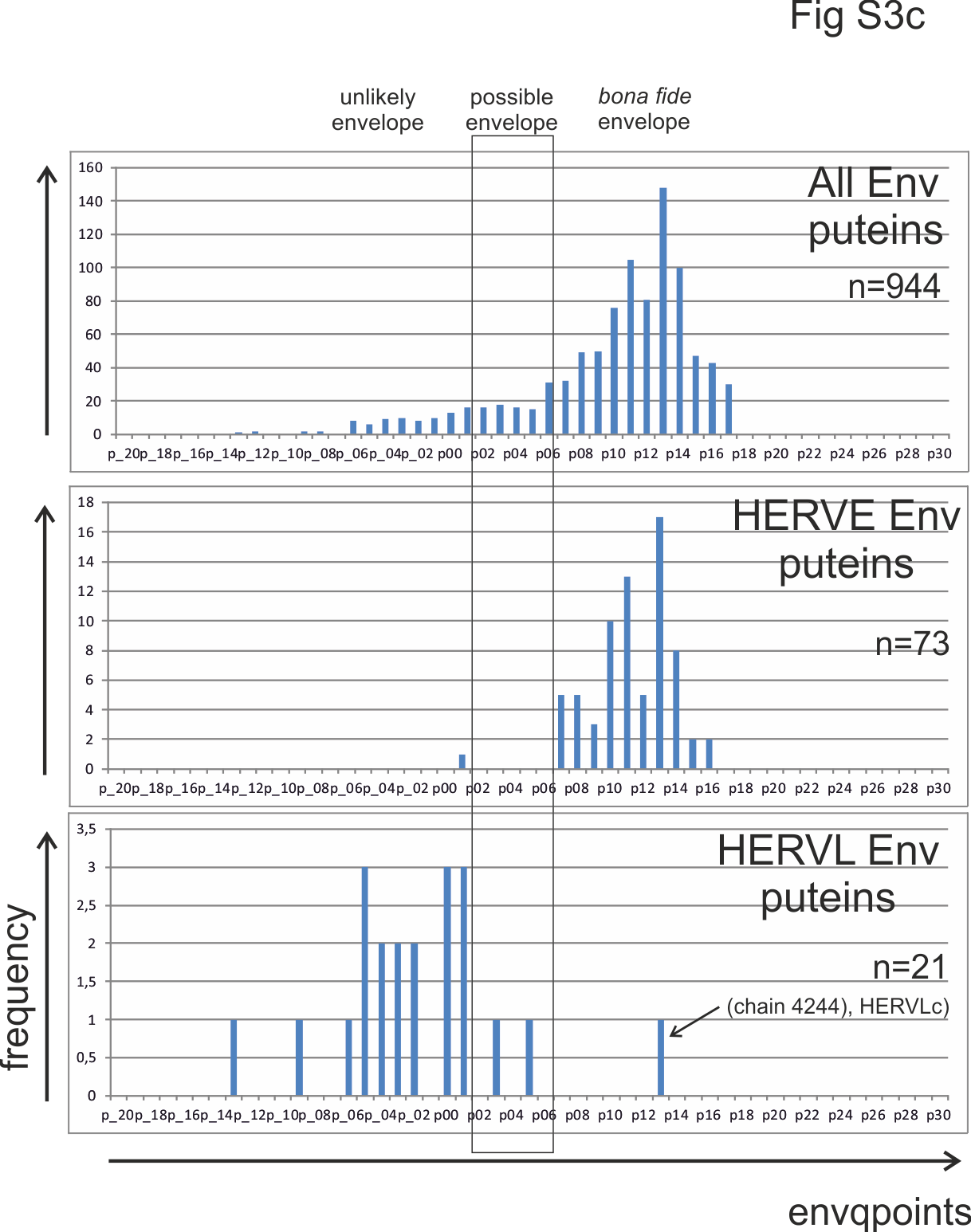
**

**Figure S3.** Results of the EnvQual program with all envelopes. Env subgroup consensuses were constructed only from envelopes scoring over 6 envqpoints. The artefactual nature of nearly all HERVL Env, and non-artefactual of HERVE Env, was supported.

1. **Discussion of secondary integrations in chains of hg19, and possibly artificial ReTe chains.**

The frequency of *secondary integration* per group was studied using the sense feature of the Simages. It is expected that a secondary integration will insert in antisense in 50% of cases. Antisense insertions were observed in 562 of the chains. Of these 231 were short pieces of Class III elements (52 MST, 145 MLT, 34 THE), whereas 224 were LTRs of other than class III origin. Antisense LTRs occurring in at least 5 chains were, using RM nomenclature, 5 LTR2 (HERVE), 26 LTR5 (HML2), 7 LTR6 (“HERVS71”, here HERVT), 34 LTR7 (HERVH), 16 LTR8 (HUERSP3), 7 LTR9 (HUERSP3), 10 LTR10 (“HERVI”, here HERVIP), 23 LTR12 (HERV9), 5 LTR13 (“HERVK13”, here HML4), 5 LTR14 (“HERVK14”, “HERV-K14” and “HERVKC4”, here HML1, 9, 10, respectively), 5 LTR15 (HERVI), 15 LTR16 (MER71A), 8 LTR33 (MER55) and 5 LTR48 (MER4I group). Thus, the most frequent antisense LTRs belonged to the most frequent proviruses (HML2, HERVH and HERV9), as reported here, and as expected if secondary integrations are random with respect to the combination of primary and secondary sequence.

A simage pattern which particularly suggests *artificial joining of proviral fragments by ReTe* is "LTRx..>(0)n<..LTRy" (n ranging between 1 and 17), or variants of it, where two phylogenetically unrelated fragments are joined via a sequence not recognized in the RMRef table, shown by the "0". It is our experience that a nucleotide search with RMRef detects most, but not all, retroviral sequences. It needed to be complemented with RVRef, HML and Consensus sequence nucleotide collections (with their Simages; plus a nonLTR search which uses the non-LTR portions of the RepeatMasker Library of 2012; not shown), as well as protein searches, like with AutoFrame. The similarity searches inherent to ReTe (shown in the BestRefRv [based on the entire nucleotide chain] and PolClass [based on Pol amino acids] fields of Table S1, also aided classification. If a "0" is present in a position in all Simages, the likelihood that the twentieth is nonretroviral is relatively strong. A possibility is that inclusion of such nonretroviral sequences may have arisen via the "broken chain" ReTe function, which is recorded in the "breaks" field. This pattern occurred in 118 chains. They were marked as potentially artificially joined in Table S1. Twelve of 118 (10%) possibly artificial chains had breaks, while 376 of all 3173 (12%) chains had breaks. Thus, the broken chain function is not likely to be a major factor behind the possibly artificial chains.

1. **Discussion regarding cross-clade relationships of envelope proteins**

Among Class I, HERVT Env subgroup A (HERVT_a) was highly related to Avigamma1 and Avibeta2 (Fig. 6-7), as expected from its relation to the MLV-like ERVs (MLLV, (1)), HERVE subgroup A (HERVE_a) was highly similar to HERVIPc, Harlequin_b highly related to HERV9_d, HERV3_b to HERVI_a, HERV1_c to HERV3_c and HERV1ARTIODACT_a to HERVE_b, indicating frequent intraclass I *env* recombination. HERVADP_a Env turned out to be highly related to Env of Chicken retrovirus 1 (Chirv1) (2). HERVIP_a Env was related to ERVPb1 Env (3), HERVFC_a Env was related to the primate Class III ERV3-1-CJ of RepBase, PABL_a Env to horse Class III ERV3-1N-EC of RepBase.

Among Class II ERVs HML1_a Env was related to HML4_a and HML9_a, HML1_c to HML2_a, HML3_a to HML10_a and HML4_a to HML7_a. Although the probable HML5 ISD (starting with "LLLQ") was aberrant, the HML5 Env puteins clustered together next to the other HML Envs and had high EnvQual scores. The other phylogenetically old HML group, HML6 (4), also had a different ISD than the other HML Envs, starting with "LKNKLN" or "LQNKIN" instead of "LANQIN" or "IVNQIN" of the other HML (HML1-4 and HML7-10) Envs.

Among Class III ERVs HERVL_c Env (from chain 4244) had a recognizable ISD ("LDNQLALDZLLAKZTRVCVITNT ") Class I Env, related to the Class I envelopes from MER101, ERVV1 and ERVV2 (5). HERVS had an envelope related to PRIMA41. HERVL32 and HERVL66 Env were similar to PRIMA4_a and HEPSI2_b, respectively. Although not part of our hg19 dataset, HERVL70 Env (deduced by us from the RepBase sequence) was found to be related to that of MER101 and HEPSI1a (Fig. 5-7).

In a search for Class III ERV envelopes outside of hg19, four envelope containing reading frames were found in RepBase (from Turtle; "ERV3-1_CPB-I_4p_Env" [clustering relatively close to MER41 Env], Frog; "ERV3-1-I_XT3p_Env" [clustering relatively close to Hepsi1 Env], Horse; "ERV3-1N-EC_I_2p"_Env [clustering with Prima41 Env, like for HERVS Env] and Primate; "ERV3-1_CJ-I_2p_Env" [clustering with HERVFC Env], Fig. 7). They had Class-I like envelopes, with ISDs starting with "LQNR..". Thus, all Class III ERVs with *env* studied in this work had a Class I *env*.

**4. Discussion of retroviral ORFs found in hg19.**

The following HERV chains ORFs or near-ORFs (where c=canonical; nc=noncanonical):

*gag* (average predicted length 352aa), with sum of shifts+stops<2, length >100aa : HERVE (2c, 1nc), HERV9 (3c 2nc), HERVFC (1c 1nc), HERVIP (1nc), HERVT (1nc), HML2 (1c 15nc), HML3 (1nc), HML4 (1nc).

*pro* was frequently open (possibly because of its shortness; average predicted length 91aa).

In total 966 chains fulfilled a shift+stop<2 criterion with an over >70aa putein. Clades which frequently had such Pro ORFs were HERVH (191c 157nc), HERVIP (28c 13nc), HML3 (9c 35nc) and HML2 (7c 26nc).

*pol* is the longest gene. It was predicted to encode an average of 821 aa.

Thr following chains fulfilled the criterion of shifts+stops<3, and length of >700aa HML2 (12nc), HML4 (1nc), HERV9 (1c), HERVFC (1c).

*env* was predicted to encode an average of 178 aa. This was probably articifially short because ReTe can have problems finding full length Env. Chains with intact or almost intact *env* genes are shown in Table 4b. When shifts+stops<3 and >200aa were required, 42 Env puteins were found. Of them, 18 were from HML2 (1c 17nc), HERVH (7c), HERVT (2c), HERVE (1c 1nc), HERVW (1c 1nc), HML1 (1nc), HML5 (1nc), HML6 (1c), HML7 (2c), HML8 (1nc), HERV3, HERVFC (1c), HERVIP (1nc), PABL (1c). Some of these envelopes were earlier described, some not. For example, the large survey of De Parseval et al (6) did not mention the HERVE envelopes described here. The following concordances were observed: Syncytin-1 (*envW* of (6)), the envelope protein of HERVW on chromosome 7 (rvnr 2556, 0 shifts and 0 stops). Syncytin-2 (*envFRD* of (6)), the envelope protein of HERVFRD on chromosome 6 (rvnr 2073) is detected by ReTe, but was allotted three frame shifts and no stops by ReTe, therefore not included . The envelope of HERV3, rvnr 2521 on chromosome 7 (rvnr 2521, 0 shifts 2 stops), is a much studied provirus and envelope protein (NP_001007254), and corresponds to *envR* of (6). In spite of a premature stop codon (7, 8), the HERV3 envelope is expressed in certain tissues (9), illustrating that truncated HERV proteins can be expressed and possibly have function(s).The envelope of PABL (approx. equal to HERVRB), on chromosome 3, (rvnr 875, 1 shift 1 stop), is identical to *envR(b)* of (6), (locus ERB1_HUMAN).

The envelope gene of HERVT on chromosome 19 (rvnr 4639) is probably identical to *envT* of (6). Likewise, the ReTe-detected envelope genes of HERVFC (rvnr 4639), several HERVH, and several HML were also detected by (6). There is probably much more to discover about functional HERV proteins.

**5. The HERV groups**

**5.1. Justification for the chosen 39 canonical HERV groups, and remarks regarding some non-canonical ones:**

The groups were based on: 1) HERV groups from literature, with preference given to the first published group, and 2) the Oct 2014 version of RepBase Update and the RepeatMasker collection of May 2012. The clades which gave the greatest coverage, and the greatest homogeneity judged by RMRef and RVRef based simages, were chosen. Each clade was represented by a DNA consensus sequence. A goal was that the consensus should have an average identity to the clade member sequences of at least 80% (see also the consensus sequence compilation S2, and table S8). It was however not possible to achieve this in all cases. ERVs which endogenized a long time ago (like the HERVIPADP (10)) have mutated to an extent that this classification criterion (11) could not be fulfilled. During work with this paper, some clades were joined into another group if their average identity allowed it, other groups were split because the average identity became too low. A few such non-final clades are mentioned in the table below to allow tracing of the classification process.

**5.2. The chosen HERV groups and their correspondence to RepeatMasker/RepBase nomenclature**

**5.2.1. Legend for the terms used:**

Chaingenus: the main retroviral genus, as determined by ReTe, is given in upper case letter, lower case letters indicate other chaingenus determined by ReTe within the same clade. It is based on a weighted mean of motif usage when the ReTe chain is built.;

PBS (primer binding site): upper case letters show the most used PBS used within a clade, lower case letters or upper case in bracket indicate other PBS found to be used by some sequences of the same clade. Some PBS sequences (E,F,H,K,L,S,T) are strongly connected with a certain HERV group. Others are more promiscuous. nd=not determined;

Znf (zinc finger motif in Gag): numbers indicate the principal Znf motif used within a clade, secondary numbers used by a minority of sequences in the same clade are shown within brackets. A subset of HERV Class I have only one zinc finger;

Frameshifts: predicted frameshifts (translational strategy) between the respective putein ORFs boundaries, Gag-Pro and Pro-Pol, are given. Major frameshift patterns are for Class I: 0;0 and for Class II -1,-1. ;

DU (dUTPase domain in Pro): The symbol + or – indicates presence or absence of the dUTPase domain within a clade, respectively. Most Class II HERVs have this motif.;

G-Patch (C-terminal Protease motif; see main text): The symbol + or – indicates presence or absence of the G-Patch motif within a clade, respectively. Most Class II ERVs have G-Patch.;

GPY/F_Chromodomain (C-terminal Polymerase motif; see main text): The symbol + or – indicates presence or absence of the domain within a clade, respectively. Most Class I HERVs have this motif.;

ISD: "Immunosuppressive" domain. It is based on hits with the motif TM2, and a program which detects cysteine rich portions of SU and TM, the probable SU/TM cleavage site, the ISD and the hydrophobic stretch of TM; see main text and materials and methods. Although originating from Snyderman and Cianciolo (12-14), the motif and its functions, in XRVs and ERVs, have mainly been explored by Heidmann´s group, see e.g. (15). ISD consensus sequences distinguish between HERV supergroups, like HERVERI, HERVW9, HERVIPADP and HMLs (see main text). ISDs were both identified manually, and by the EnvQual program. ISD consensuses were first calculated manually, then automated after alignment. Both consensuses are shown to illustrate the degree of certainty.

**5.2.2. Class I (gamma- and epsilonretrovirus-like) elements**

**5.2.2.1. MLLV* supergroup, taxorder 10100**

**HERVT (nc12 c21), taxorder 10110:**

The group was described as S71, SSAV1, CRTK1, CRTK6 by Leib-Mösch and colleagues (16-18). Members of the same clade were named Hs5 by Levy et al (19), and HC2 by Kabat et al (20).

Its LTR is LTR6. HERVT is equivalent to HERVS71 In RepBase.

Taxonomic markers:

Chaingenus: C, cd; PBS: T; Znf: 1; Frameshifts: 0 1 -1; 0 1 -1; Gpatch-; DU-; GPY/F_chromodomain+

AutoFrame hits:

Gag (23 found): HERVS71

Pro (13 found): MULV-INT, HERVS71, BAEV, MURRS-INT

Pol (30 found): HERVS71, CFERV1, BAEV,

Env (13 found): HERVS71

ISD: LQNRzGLDLLFLSQGGLCtALG, LQNhzGLDLLLLSQGRLC, LQNpRGLDLLFLSQGGLCAALG, LQNRRGLDLLFiSQGGLCtALE, fkNhqGLDLLFpSQGeLCAALG, LQNcRGLDLLFLSzGGLCAALE, LQNcRGLDLLFLSQGGLCAALG, LQNRzGLDLLFLSQGeLCAALG, LQNcRcLDLLFLSQGGLCAALG, LQNRzGLyLLFvSQGGLCtALG, LQNRRGLDLLFLSQGGLCAALG, LQNRzGLDLLFLSQGGLCAALG, LQNcRGLDLLFLSQrGLCtALG

Manual ISD Consensus; LQNcrGLDLLFlSQGgLCaALg

One Env subgroup;

Hervt_a_lqnrrgldllflsqgglcaalge

**5.2.2.2. HERVERI supergroup, taxorder 10200**

**HERVE (107nc 41c), taxorder 10210:**

The term was introduced by Martin´s group (21) (clone name 4-1; other names ERVA, NP-2), and exists in RepBase.

Its LTRs are LTR2.

Taxonomic markers:

Chaingenus: C; PBS: E, h; Znf: 1, (2); Frameshifts: 0 (-1 1); 1 -1 0; Gpatch-; DU-; GPY/F_chromodomain+

Autoframe hits:

Gag (117 found): HERV3, HERVE_A

Pro (111 found): ERV1-2-I_BT, HERV3, HERVE_A, HERVE, HERVS71, MMERGH-INT

Pol (132 found): BAEV, CARLTR1-INT, CFERV1, HERV3, HERVE_A, HERVE, HERVIP10FH

Env (69 found): HERVE_A, HERVE, MER70-INT

Manual ISD; YQNRLALDYLLA and variants of it, LdNRfALeYLLA (MER70-INT)

Two Env subgroups;

Herve_a_yqnrlaldyllaaeggvcgkfnl

Herve_b_ldnrfaleyllaeqgrvctvinh

**HARLEQUIN (68nc), taxorder 10220:**

The term derives from RepBase. It was introduced by Kapitonov and Jurka 1998.

This is a recombinant mainly containing HERVE, HERVIP10 and HERV9 information. It uses LTR2, the HERVE LTR. Its DFAM identity code is DF0000017.

As described in the main text, there are many intermediate recombinant forms highly related to HARLEQUIN. The chosen 68 chains have a consistent internal structure, which is evidence for a recombinant with a high replicative potential. HARLEQUIN is here treated as a noncanonical HERV clade.

Taxonomic markers:

Chaingenus: C; PBS: E; Frameshifts: 0,1; 0; Znf 1; Gpatch-; DU-; GPY/F_chromodomain+

AutoFrame hits:

Gag (1 found): HERVE

Pro (1 found): MMERGLN-INT

Pol (17 found): HERVE_A, HERVE, HERVIP10FH, RTVL-IB-INT

Env (63 found): ERV3-1_CHO, HERVE, HERVE_A

ISD; YQNRLvLDhLLA, YQNRLAfDYLLA, YrNRLALDYLLA, YQNRLALDYLLA and many variants.

Manual ISD Consensus: YQNRLALDYLLA

Two envelope subgroups were found,

Harlequin_a_yqnrlaldyllaaeggvcrkfnl

Harlequin_b_yqnrlaldyllaaeevvcgkfnl

**HERV3 (37nc 20c), taxorder 10230:**

The term was introduced by O´Connell in O´Brien´s group (22), and exists in RepBase.

Its LTRs are LTR4, LTR76 or LTR61. Internal sequence HERV3i. Partial overlap with HERV1, RHERVI, HERV15 and HERVE.

Taxonomic markers:

Chaingenus: C; PBS: R, P, w, e; Znf: 1; Frameshifts: 0,-1 1;0-1 1; Gpatch-; DU-; GPY/F_chromodomain+

AutoFrame hits:

Gag (41 found): HERV3, HERV1-I

Pro (34 found): HERV3

Pol (45 found): HERV3, HERV1-I, BAEV, CARLTR1-INT

Env (30 found): HERV1-I, HERV3

ISD; YQNRLALDYLLA, YQksLALnYLLA, YQNRLAinYLLA, YQNRLALDhLLA, YQNsLALDYLLA, YzNRLALnYLLA, hQNRLsLnYFLv.

Manual ISD Consensus YQNRLALnYLLA

Three Env subgroups;

Herv3_a_yqnrlalnyllaqeggvcgkfnl

Herv3_b_yqnrlaldyllaqeggvcgkfnl

Herv3_c_yqkrlaldifzlqkeefvenltn

**HERV1 (11nc 2c), taxorder 10240:**

The term was introduced by O´Brien (23), and exists in RepBase.

Taxonomic markers:

Chaingenus: C; PBS: P,L,r,t; Znf: 1; Frameshifts: 0,1-1;0-1 1; Gpatch-; DU-; GPY/F_chromodomain+

Class I. LTR is LTR35A. Internal sequence is HERV1. Partial overlap with HERV3 and HERV15.

AutoFrame hits:

Gag (10 found): HERV1-I, HERV3, PABL_B

Pro (10 found): HERV1-I, HERV3, PRIMA4-INT

Pol (11 found): HERV1-I, CARERV4, HERV3, BAEV

Env (9 found): HERV1-I, MER70-INT

ISD; YQNRLALDYLLA, YQNRLALDHLLA, fdNRiALDcLLA (MER70-INT), LdNiiALDsiLAEQGGICvAiN (MER70-INT), cQNRLALDYLLA, YQNRLvLnYvLA.

Manual ISD Consensus YqNRlALDylLAeqggicvain

Three Env subgroups;

Herv1_a_fdnrialdcllaeqggiraiayt

Herv1_b_ldniialdsilaeqggicvains

Herv1_c_yqnrlaldyllaseggvcgklnl

**HERVI (13 nc, 3 c), taxorder 10250:**

These sequences were first described by (24).

LTR is LTR15. It here contains the RepBase and literature terms HERV15, RHERVI, RRHERV-I (25) , RTVL-I (24) and Rtvli-int. It is highly related to HERV1 and HERV3, belonging to the group HERV-ERI.

Taxonomic markers:

Chaingenus: C, cd; PBS: I, P, E; Znf: 1; Frameshifts: 1 0 -1; 1 -1 0; Gpatch-; DU-; GPY/F_chromodomain+

AutoFrame hits:

Gag (9 found): HERV3

Pro (7 found): HERV3

Pol (8 found): HERV3, HERV1-i, BAEV, CFERV1

Env (9 found): HERV1-i, HERV3, MACERVK2 (this chain may artificially have joined an HML6 with a HERVI)

ISD; YQNkLtLDYLLv, YhNRLALDYLLA, YQNRLALDYLLA, YzNRLALDYHLA, hQNRLALDYLLA

Manual ISD Consensus: yQNRLaLDYLLa

Four Env subgroups;

Hervi_a_yqnrlaldyllaseggvcgkfnl

Hervi_b_yqnrlaldyllazegrvcekfnl

Hervi_c <no ISD detected>

Hervi_d_ldnelalhyllaeqggiyavtsr

**5.2.2.3. HERVW9 supergroup, taxorder 10300:**

**HERVW (nc86 c40), taxorder 10310:**

The term was introduced by Blond et al (26).

Its LTR is LTR17. Equivalent to HERV17 in Repbase.

The HERVW clade merges with HERV9. They do however cluster separately in Pol and chaindna based trees. Taxonomic markers:

Chaingenus: C, a, b; PBS: W, p, r, i; Znf: 1; Frameshifts: 0 1 -1; 0 -1 1; Gpatch-; DU-; GPY/F_chromodomain+

Autoframe hits:

Gag (96 found): CFERV1, ERV3-2_CJA, HERVIP10F, MER52-int, ERV1-1, HERV9, GYPSY-18_DPU (short piece of zinc finger; RaCFQCglqGHfKkDCPgRN), MER84-INT, HERVP71A, LTR25-INT, GYPSY-97_AA, BEL-1-is-i (zinc fingers CFQCgLQGHfkKDCpnrnkppprpCTSCqGnHckAhCPrgRrS), HERVFC2, MER34-INT, MACNERV5, CFERV2, HERVH48, ETNERV3 (Class II element), GYPSY-2_ANO (Long stretch, KLSDNPdGyidVLQGLeQcfyrTztDImlLLDqtLTtKERsatitaaREfgnlwylsqVnDrmTTeerEqfstgQEaVpsVDPhWDakSeHGDwcRrHlltcVQeLRKtrrKtmNysMmsTitQEkEKnPtAFLEtLREAlrKHtslShDsiEGQLiLKdkFItQsaaDIRtKLQKsAlgpeqnLEtLLnLAtsVFyNKD)

Pro (96 found): HERV9, MER34-INT, HYLERV9, MER52-INT, ERV22_MD, GGLTR11-INT, HERV4-I,

Pol ( 89 found): HYLERV9, MER52-INT, HERV9, LTR77-INT_TS (*Tarsius syrichta*; long, RDLTVWTsHDVnsILTAKGdLWLSDKYQALLLErpvLrLhtCATLNPAkFLPDNeeKmEHNCQQaIaQTYAtzrDLlEvPLtDPDlnLYTDGSSFaEKGLQKvGYAVVSdNgILES), PABL-B-INT, CARLTR1-INT,

Env (9 found): BAEV, HERV9

ISD: LQNRRALDLLTAERGGTCLFLG, LQNRRdLDLLTtKRGGTCLFLG, LQNqRvLDLLTtERGGICLFLG, LQNRRALDLLTAERGGTCLFLG, fQNzRALDLLTsERGGICLFLK, LQNRRALDLLTAERGGTCLFLG, LQNRRGLDLLTAEKGGLCIFLN

Manual ISD Consensus; LQNRRaLDLLTaERGGtCLFLg

Two Env subgroups;

Hervw_a_lqnrraldlltaerggtclflge

Hervw_b_lqnrrgldlltaekgglciflne

**HERV9 (171nc 114c ), 10320:**

The term derives from LaMantia (27) under the initial name pHE1, and exists in RepBase.

its LTRs are LTR12, Internal portions are either termed PTR5 or HERV9-int.

Taxonomic markers:

Chaingenus: C, cb, cd; PBS: W, R, k, p, c; Znf: 1, (2); Frameshifts: 0,-1 1;0 1 -1; Gpatch-; DU-; GPY/F_chromodomain+

AutoFrame hits:

Gag (266 found): HERV9, ERV1-1_SSC-I, LTR25-INT, RSV-INT (ERVK!), HERV9N, MDOERV3, ERV6_MD, GYPSY-87_AA-I, MER84-INT, COPIA-21_PIT, HYLNERVH2, HERVP71A, MER52-INT, HERV4-I, ERV1-I_EC, CFERV1, ERV1-1-I_BT, LTR25-INT, BTERVF2-I, GYPSY-12-DEL, HERVH, HERV-FC2, HYLERV9, HERVH48,

Pro (209 found): HERV9, ERV1N-2_SSC-I, HYLERV9, ERV1-2-)_BT, MER52-INT, ERV2_TSY-I, HERV3, ERV1-8_AMI, HERV9N, HERVK9 (HERV9-HML3 recomb), RNLTR10-INT, MER34-INT, HERVH48,

Pol (211 found): HERV9NC, HYLERV9, MER52-INT, CARLTR1-INT, CFERVF2, MER84-INT, HERVI,HERV4-I

Env (99 found): HERV9, BTERVF2-I, HYLERV9-2_LTR, HERVIP10F, HERVE,

ISD; LQNcqGLDLLTAEKGGLCtFLG, LQNczdLDLLTAEKGGLCtFLG, LQRhzGLDLLiAEKGGLCtFLG , etc.

weNRiALDmiLA (HERVIP10F), nQNRLALDYLpA (HERVE),

Manual ISD Consensuses; LQNcqGLDLLtAEKGGLCtFLG, weNRiALDmiLA (HERVIP10F), and nQNRLALDYLpA (HERVE)

Four Env subgroups;

herv9_a_lqnczgldlltaekgglctflge

herv9_b_lqnhzgldlltaekgglctflge

herv9_c_enrialdmilaekgrvcvmigvq

herv9_d_nqnrlaldylpaaeggicgkfnf

**HERV30 (6nc), taxorder 10330:**

The term comes from RepBase.

Its LTR is LTR30. The entire provirus is named HERV30. It is highly related to HERV9, HERVW, LTR19 and MER52.

Taxonomic markers:

Chaingenus: C; PBS: R; Znf: 1; Frameshifts: -1 0;1 0; Gpatch-; DU-; GPY/F_chromodomain+

AutoFrame hits:

Gag (5 found): MER52-INT, HERV9, BTERVF2-I

Pro (3 found): HERV9

Pol (1 found): HERV9

Env (1 found): BTERVF2-I

Manual ISD; LQNRRALvLLTAEKGGTCLFLG

One Env subgroup;

Herv30_a_lqnrralvlltaekggtclflge

**MER41 (11nc), taxorder 10340:**

The term comes from RepBase.

Its LTR is MER41. Its internal structure is MER41-INT. Belongs to MER4I group.

Taxonomic markers:

Chaingenus: C, cb, cs; PBS: W; Znf: 1, (2); Frameshifts: 0 -1; 1; Gpatch-; DU-; GPY/F_chromodomain(+)

AutoFrame hits:

Gag (2 found): MER84-INT, HERV9

Pro (0 found):

Pol (2 found): CARLTR1-INT, HERV9

Env (2 found): LTR25-INT

Two Env subgroups;

Mer41_a_mqnrmsldtltaaqggtcaiiri

Mer41_b_mqnrmsldtltaaqggtcaiiri

**LTR19(6nc), taxorder 10350:**

The term is from RepBase.

Its LTRs are LTR19a (Like for HERVFa, a contradiction). It is also called HERV19i in RepBase. It is part of the protean MER4I group (see above). LTR19 is intermediate to the HERVHF and HERVW9 supergroups, here placed in HERVHF.

Taxonomic markers:

Chaingenus: C, cd, cs; PBS: F, r; Znf: (1); Frameshifts: ?; ?; Gpatch-; DU-; GPY/F_chromodomain-?

AutoFrame hits:

No Gag, Pro, Pol or Env puteins.

**HERV35 (1nc), taxorder 10360:**

Its LTR is LTR35. Its internal sequence is HERV35I. Although here placed in HERVW9, it is also highly related to MER4I and LTR19-INT.

Taxonomic markers:

Chaingenus: C; PBS: P; Znf: ?; Frameshifts: ?;?; Gpatch-; DU-; GPY/F_chromodomain-

AutoFrame hits:

Gag, Pro, Pol: 0 found.

Env (1 found): LTR25-INT

**5.2.2.4 HERVIPADP supergroup, taxorder 10400:**

**HERVIP (72 nc, 67 c), taxorder 10410:**

The term comes from Seyfarth et al (28) where it was called HERV-IP-T47D or ERV-FTD.

Its LTR is LTR10. It is often called HERVIP10F in RepBase. It is related to the bird ERV, Chirv1 (2), and more distantly to HERVADP.

Taxonomic markers:

Chaingenus: C, cd, cs; PBS: P, I, k, c; Znf: 1; Frameshifts: 0 -1 1; 0 -1 1; Gpatch-; DU-; GPY/F_chromodomain+

AutoFrame hits:

Gag (91 found): HERVIP10, HERVP71A, TGUERV4-i

Pro (55 found): ERV3-1_CHO, HERVIP10F, HERVI, GGLTR11-int, HYLERV9 (sec. integr.)

Pol (112 found): HERVIP10F, HERVIP10FH, HERVIP10B3, HERVI, RTVLI-B-INT

Env (62 found): HERVIP10F, HERVIP10B3, HERVE (due to recombination), MER70-int (different Env in chain 2017, also deviating in Pro), HERV1-i,

ISD: weNRiALDmvLA, weNRiALDmiLA weNRmALDviLA, gkNRiALDviLA, wgNRiALDmLLA, weNRmALDmiLA, zeNtiALDmiLA, weNRiALDikLA, weNRmALDtiLA, weNRiALDtmLA, weNRLALDmiLA, seNRiALDmiLA, weNRmALDmmLA, reNRiALDmiLA, wkNRiALDmiLAEKGGVCimie, YQNRLALDYLLA (HERVE recomb), wkNRmALDmiLA, weNRtALDmiLAKRGGVCImiK, LhNRitLaYLLA (MER70 envelope in chain 2017), weNRiALniiLA, zeNRiALDniLA, gkNRiALDtiLA, heNRiALDmLLa, zeNRiALDmiLA, weNRiALDiiLA, gkNRiALDmiLA, geNRiALDmiLA, rkNRitLDmiLAEKGGVCIiiK

Manual ISD Consensus; weNRiALdmiLAekggvciiik

Four Env subgroups;

Hervip_a_wenrialdmilaekggvcvmikt

Hervip_b_wenrigldmllaesggvcamigt

Hervip_c_yqnrlaldyllaaeggvcgkfnl

Hervip_d_yqnrlaldyllaseggvcgkfnl

**HERVADP (8nc 16c), taxorder 10420:**

The term was introduced by Lyn et al (29). ADP refers to the ADP-ribosyl transferase locus, where a provirus was found. It does not exist in Repbase.

LTRs are LTR71. Its closest clade in RepBase is HERVP71A.

Taxonomic markers:

Chaingenus: C, cd; PBS: F, h; Znf: 1, (2); Frameshifts 0 -1 1; 1 -1; Gpatch-; DU-; GPY/F_chromodomain+

AutoFrame hits:

Gag (19 found): HERVI, HERVP71A,

Pro (7 found): ERV3-1_CHO-I, HERVP71A

Pol (15 found): GGLTR7-INT, HERVI, HERVP71A, RTVLIB-INT

Env (9 found): ERV3-1_CHO-I, HERVP71A

Manual ISD; weNRLALDmiLA and variants of it

One Env subgroup: Hervadp_a_wenrlaldiilaekggicvmlgg

**5.2.2.5. The MER50LIKE supergroup, taxorder 1500:**

**MER50 (14nc), taxorder 10510:**

The term is from RepBase.

Its LTR is MER50. Internal sequence is MER50-int or MER83-int. Part of the MER4i group.

Taxonomic markers:

Chaingenus: C, cd, gc; PBS: H; Znf: 2; Frameshifts: 1; ?; Gpatch-; DU-; GPY/F_chromodomain+

Autoframe hits:

Gag (3 found): GYPSY-15_PIT-i (Two zinc fingers, KPsspsRLPQNtlPgnChwCGKpGHWkAmClnGisgkkfrtaCHlCHKlGYwkRgClEgzRvprtE), MACERV5A, MGRL3-i (gypsy, zinc finger; GnChQCRKPGHwNaNCPNGineRPT)

Pro (1 found): CFERVF1

Pol (0 found)

Env (3 found): MER84-INT (next best is MER83-INT), LTR25-INT, ERV2B-CPO

ISD: fQNczGLDmLmAAhEGICLALd, mdNRLALDYLLA (MER84like), LQsszgLGmLiTvQQGICLALp Approximate manual ISD Consensus; LQNRLGLDmLLAAQQGICLAL

Three Env subgroups;

mer50_a_fqnczgldmlmaahegiclalde

mer50_b_mdnrlaldyllaeqsrlcvinkp

mer50_c_lqsszglgmlitvqqgiclalpl

**MER57 (14nc), taxorder 10520:**

The term comes from RepBase.

Its LTRs are MER57. Its internal structure is MER57-INT. It belongs to the MER4I group.

Taxonomic markers:

Chaingenus: C, cd, cb, cs; PBS: L; Znf: 0?, 1, 2; Frameshifts: 1; ?; Gpatch-; DU-; GPY/F_chromodomain-

AutoFrame hits:

Gag (1 found): RODERV21

Pro (0 found):

Pol (0 found):

Env (5 found): MER70-INT, GGLTR7-INT

ISD; LnNRiALDYLLA, LnNRiALsYLLA, LnNRtALDylla

Manual ISD Consensus: LNNRiALdYLLA

Two Env subgroups;

Mer57_a_lnnrialdyllakqqylcscwyl

Mer57_b_lnnrialsyllakqrsiwhflf

**MER84 (7nc), taxorder 10530:**

Its LTR is MER84. The internal sequence is MER84-INT. It is related to MER83, HERW/HERV17 and HUERSP3. It belongs to the MER4I group. MER83 was joined with MER84. Its LTR is MER83.

Taxonomic markers:

Chaingenus: C, cd; PBS: k, r?; Znf: 2, (1); Frameshifts: -1 0; 0 -1; Gpatch-; DU-; GPY/F_chromodomain+

AutoFrame hits:

Gag (5 found): MER84-INT, CFERVF1

Pro (3 found): LTR46-INT, HERV4-I

Pol (2 found): THE1-INT (This provirus had a THE insertion), CFERVF2

Env (2 found): HERV9, MER84-INT

Manual ISD; sgNRzvLDYLLA (MER84-INT), LQNccGLnpLmAAQEdICLAFE (HERV9)

Three Env subgroups;

Mer84_a_sgnrzvldyllaeqggvcavinr

Mer84_b_lqnccglnplmaaqediclafek

Mer84_c_lqnrcglnlltaaqgliclalqk

**5.2.2.6. HERVHF supergroup, taxorder 10600:**

**HERVH (500nc 531c), taxorder 10610:**

The term RTVL-H was introduced by Mager and Henthorn (30), later called HERV-H. It exists in Repbase. HylNERVH (Hylobates, i.e. Gibbon, HERVH) of Repbase/RepeatMasker is closely related.

LTRs are LTR7, a few times also MER72 (in chains also related to HERVH48).

Taxonomic markers:

Chaingenus: C, cd; PBS: H, f, k; Znf: 1 (972), 2 (14), 0 (71); Frameshifts: -1 0 1; 0 -1 1; Gpatch-; DU-; GPY/F_chromodomain+

AutoFrame hits:

Gag (828 found): MACNERV6A, HERVH, HERVH48, LTR19-INT, HYLNERVH1, CFERV, MACNERV5, HERVFC1, GYPSY25_OD-I, GYPSY-2_DTA-I, GYPSY2_PP-I, HERV4-I, GYPSY-15_PIT, GYPSY-7_BG-I, ETNERV3 (A damaged ERVK.), SC3-INT (COPIA; Severely damaged.), GYPSY-13_CSA (Severely damaged), GYPSY-15-I_DYA, COPIA-9_GM-I, COPIA-21_GM-I (Damaged), LTR46-INT, COPIA1-I_DPSE, ERV1-1_STR, MDOERV2, GYPSY-15_DR, ERV1-I_EC, RODERV21-I, HERVIP10F

Pro (363 found): BTERVF1-I, BTERVF2-I, CFERVF1, CFERVF2, ERV1-1-I_DR, ERV1-I_EC, ERV2-4_TSY-I, ERV22_MD, ERV25_MD, ERV3-2_CJA, ERV4_DR-I, HERV3, HERVE, HERVH48, HERVS71, HYLNERVH1, LTR46-INT, MACERV6, MACERV5, MURRS-INT, MURVY-INT, RODERV21

Pol (793 found): CARERV4, CFERV1F, HERVH48, HERVH, HYLNERVH1, HYLNERVH2

Env (97 found): BTERVF1-I, CFERVF2, HERV9, HERVE, HERVH48, HERVH, HERVS71, HYLNERVH2, LTR19-INT, MACERV1, PTERV2A, RLTR4_MM-INT, RNLTR17-INT, RNNICER3-INT, TGU_REP1

ISD; LQNRRGLDLLTAEKGGLCIFLN (HERV9,BTERVF1), YQNRLALDYLLA (small HERV3 portion), YQNgpALDYLLA (TGU_REP1).

Manual ISD Consensuses; LQNRRGLDLLTAEKGGLCIFLN and YQNRLALDYLLA.

Hervh_a lqnrrgldlltaekggavcmcvl

Hervh_b lqnlrglnlltaekgglciflne

Hervh_c lnhrkiacisnlkigqikywsaq

Hervh_d yqnrlaldyllaeeggvcgqfpi

**HERVH48 (8nc 16c), taxorder 10620:**

The term is from RepBase.

Its LTRs are MER48, LTR21A and MER72. HERVH48 is highly related to HERVH and HERVFb.

Taxonomic markers:

Chaingenus: C, cd; PBS: F, h; Znf: 1, (2); Frameshifts: -1 0 1; 1 -1; Gpatch-; DU-; GPY/F_chromodomain+

AutoFrame hits:

Gag (17 found): HERVH48, LTR19-INT, LTR46-INT

Pro (14 found): HERVH48, ERV25_MD-I, ERV3-2_CJA-I

Pol (19 found): CFERVF1, HERVH48, LTR46-INT

Env (6 found): TGUERV7K, HERV3, HERVH48

Manual ISD; YQNmLALDYLLA(TGUERV7K), YQNRLALDYLLA (HERVH48)

One Env subgroup;

Hervh48_a_yqnrlaldyllaeeggvcgkfni

**HERVFA (7nc 8c), taxorder 10630:**

The term came from Kjellman, Sjögren and Widegren (31). It occurs in RepBase.

Its LTRs are LTR19A and LTR19B. It is also called HERVFH19 in RepBase.

Taxonomic markers:

Chaingenus: C, cd; PBS: F; Znf: 2, (1); Frameshifts: 0 1 -1; 1 0 -1; Gpatch-; DU-; GPY/F_chromodomain+

Autoframe hits:

Gag (10 found): LTR46-INT, HERV-FC2, LTR19-INT, CFERVF1, CFERVF2, MER84-INT

Pro (6 found): CFERVF1, RODERV21, BAEV, BTERVF1

Pol (8 found): CFERVF1

Env (7 found): HERV9, BTERVF1-I

ISD; LQNRqeLDviTtERGdLCLsFg, LQNRqGLnLiTAEnwsLCLsLG, LQNRwGLnLimvEKGGLCLSLg, LQNRwGLDLimAEKRdLCLsLG, LQNkwGLnLiTAEKGGLCLsLG, LQNRRGLDLLtTEKGGsCLsLG, LQNRLGLDLTmAEKGGLCLsLS.

Manual ISD Consensus; LQNRwgLdlimaErgglCLslg

One Env subgroup;

Hervfa_a_lqnrwgldlitaekgglclslge

**HERVFB (14nc 8c), taxorder 10640:**

The term came from Kjellman, Sjögren and Widegren (31). It occurs in RepBase.

LTRs are LTR21B/LTR21C. HERVFb is synonymous with HERVFH21 in RepBase.

The LTRs LTR21A, MER72 and MER48 belong to HERVH48 which is highly related to HERVFb

Taxonomic markers:

Chaingenus: C, cd; PBS: F; Znf: 2, (1); Frameshifts: -1 0 1; -1 1 0; Gpatch-; DU-; GPY/F_chromodomain+

Autoframe hits:

Gag (18 found): CFERVF1, HERVH48, CFERVF2, RODERV21, LTR19-INT

Pro (7 found): CFERVF1, MACNERV5, HERVH48, MACNERV5

Pol (15 found): CFERVF1, CFERVF2, GGLTR7-INT, CARERV4

Env (15 found): HERV9

ISD; LkNRRGLDLLTAdKGGLCIFLd, LQNRRGLnmLTAEKrGLCIFLd, LQsRRGLDLLTAEKGGICvFLd, LQNczGLDLLTAKKGGLCIFLG, LQNhRdLDLLTAEKGGLCLFLd, LQNRRGLgLLTAEKGGLCIFLd, LQNczGLDLLTAEKGGLrIFLG.

Manual ISD Consensus; LQNrRGLdLLTAEKGGLCIFLd.

One Env subgroup;

Hervfb_a_lqnrrgldlltaekgglciflde

**HERVFC (3nc 2c), taxorder 10640:**

The term came from Kjellman, Sjögren and Widegren (31), with later work from Heidmann (32). It occurs in RepBase.

Its LTR is LTR46. LTR46-int also has this LTR. There is no other term in RepBase.

Taxonomic markers:

Chaingenus: C, cd, g; PBS: F; Znf: 2; Frameshifts: 0; 0 1; Gpatch-; DU-; GPY/F_chromodomain+

Autoframe hits:

Gag (4 found): HERV-FC1, HERV-FC2, MACERV6

Pro (2 found): HERV-FC2

Pol (3 found): HERV-FC1, HERV-FC2, MACERV5A

Env (2 found): HERV-FC1, HERV-FC2

ISD; mQNRRALDLLTAdKGGTCmFLG, aQNRRALDLLTAEKGGTCLFLQ.

Manual ISD Consensus aQNRRALDLLTAeKGGTClFLq .

One Env subgroup;

Hervfc_a_aqnrraldlltadkggtclflge

**LTR46 (2nc 8c), taxorder 10660:**

The term is from RepBase. It clusters with HERVH and its relatives. It has like most of them two zinc fingers in Gag.

Taxonomic markers:

Chaingenus: C, CD; PBS: F; Znf: 2; Frameshifts: -1 0 1; 0 1 -1; Gpatch-; DU-; GPY/F_chromodomain+

AutoFrame hits:

Gag (8 found): LTR46-INT

Pro (3 found): CFERVF2, LTR46-INT, CFERVF1

Pol (10 found): LTR46-INT

Env (3 found): LTR46-INT, HERV9

ISD not manually detected.

Two Env subgroups;

Ltr46_a_lqnqraldlliaekggvcvylqn

Ltr46_b_lqnwrvldllttekggtciylqe

**5.2.2.7. Supergroup HERVFRDLIKE, taxorder 10700:**

**HERVFRD (10 nc, 1 c), taxorder 10710:**

The term came from Leib-Mösch [(33); GenBank Id U27240]. It does not occur in RepBase.

Its LTR is LTR58. HERVFRD is a synonym of ERV3-1-i in RepBase but it is also related to MER50.

Taxonomic markers:

Chaingenus: C, cd; PBS: H; Znf: 2, (1); Frameshifts: 0 -1; -1 1 0; Gpatch-; DU-; GPY/F_chromodomain(+)

Autoframe hits:

Gag (9 found): RODERV21, MACERV5A, ERV1-3_SSC.

Pro (6 found): MURVY-INT, HERV4-i, MMERGLN-I, ERV1-2_BT

Pol (4 found): CARLTR1-INT

Env (6 found): HERV9, BTERVF1-i.

ISD; LQNcRRLDtLTAAQGGIfLALh, LQNRRGLDmLTAAQGGICLALd, lqnyqeldkltaaqretclalg, LQNcqGLDmLmAAQGGICLALd, skNhqGLDrLmAAQGGICLASd (this Env was most similar to BTERVF1-i), LQNfqGLnmLmaAQREICLALd.

Manual ISD Consensus; LQNyqgLdmLmAAQggicLAld.

One Env subgroup;

Hervfrd_a_lqncqgldmlmaaqggiclalde

**PRIMA41 (17nc 3c), taxorder 10720:**

The term derives from RepBase.

Its LTR is MER41C, PRIMA41-int is the internal sequence.

Taxonomic markers:

Chaingenus: C; PBS: r, m, k?; Znf: 1; Frameshifts: 0 -1 1; 0 1 -1; Gpatch-; DU-; GPY/F_chromodomain+

Autoframe hits:

Gag (15 found): MER84-INT, HERV4, ERV1-I, MACNERV5, ERV1-3_SSC, PABL-B-INT

Pro (3 found): ERV1-1, BAEV, MURVY-INT, TGUERV1-I, HERV3

Pol (10 found): BAEV, CARLTR1-INT, CFERV2, CFERV1

Env (4 found): PRIMA41, HERVL66, CARLTR1-INT, HERVE, ERV1-8_AMI-I

ISD; LQNcmcLDiLTAAQGRTCALiK (HERVL66), LdNRLALDYLLA (HERVE), LNnRLALDYLLa (ERV1-8_AMI-I) Manual ISD Consensus: LQNRLALDYLLA

Three Env subgroups;

prima41_a_ldnrlaldyllaezggvcallik

prima41_b_lqncmcldiltaaqgrtcalikt

prima41_c_lnnrlaldyllaeqgrvcavine

**MER66 (13nc), taxorder 10730:**

Its LTR is MER66. Its internal structure is MER66-INT. It is related to LTR41 and to HERV19.

Taxonomic markers:

Chaingenus: C, cd; PBS: R; Znf: 1; Frameshifts: 0; 1 0 -1; Gpatch-; DU-; GPY/F_chromodomain+

AutoFrame hits:

Gag (8 found): ERV1-I_EC, MER84-INT, ERV1_MIM, ERV1-1_STR-I, RLTR4_MM

Pro (3 found): HERVE, HERV3, ERV1-1-I_XT

Pol (5 found): CFERVF2, CARLTR1_INT

Env (4 found): MER84-INT

ISD; ikNRLvLDYLLA, LdNRLALnYLLA, LnNRLAvDYLLA

Manual ISD Consensus: LdNRLALDYLLA

Three Env subgroups;

Mer66_a_ldnrlalnyllaesvgvcgqvli

Mer66_b_lnnrlavdyllazqvgevcvvvn

Mer66_c <no ISD detected>

**HERV1_artiodact (7nc 2c), taxorder 10740:**

The term is introduced in this paper. It contains chains with high similarity to the RM entities "ERV1-3_SSc-i"," ERV1-3_Bt-i", "ERV1_cow", "MER70-int" and "LTR35", where SSc means Sus scrofa (pig) and Bt means Bos taurus.

Taxonomic markers:

Chaingenus: C; PBS:R; Znf: 1; Frameshifts: 0,1-1;0-1 1; Gpatch-, DU-; GPY/F_chromodomain+

AutoFrame hits:

Gag (6 found): ERV1-3_SSC-I, MURRS4-INT, PABL-B-INT

Pro (2 found): HERV3, RLTR4_MM-INT

Pol (3 found): ERV1-3_SSC-I, CFERV1, CARLTR1-INT

Env (1 found): MER70-INT (According to RM annotation, this is an ERVL, a class III RV)

Manual ISD; LdQRLALDYLLA (this is reminiscent of HERVERI ISDs)

One Env subgroup;

Herv1artiodact _a_ldniialdsilaeqggicvains

**PABL (8nc 2c), taxorder 10750:**

The term was introduced by Fukagawa et al (34), later by Blusch et al (35). PABL is short for "PseudoAutosomal Boundary-Like". It exists in Repbase (and DFAM).

Taxonomic markers:

Chaingenus: C; PBS: R; Znf: 1; Frameshifts: 0 -1 1; -1 1 0; Gpatch-; DU-; GPY/F_chromodomain+

AutoFrame hits:

Gag (7 found): PABL_B-INT

Pro (5 found): HERV3, GYPSY-7_DVIR, TGUERV1-I, ERV1-3_SSC-I

Pol (7 found): PABL_B-INT

Env (4 found): PABL_B-INT

ISD; LQNRmGLDiLTAAQGGshALiK, LQNRmALDiiTtAQGGTCtLtK, LzNRmALDiLTAAQGGTCALiK

Manual ISD Consensus: LQNRMALDILTaAQGGtcaLiK

One Env subgroup;

Pabl_a_lqnrmaldiltaaqggtcalikt

**HERV4 (23nc 8c), taxorder 10760:**

The term exists in the Repeatmasker library of 2012, but not in RepBase. It probably derives from O´Brien but we have not found a good literature reference for that.

Its LTRs are MER51A/MER51B or MER61D. Its internal sequence is also referred to as MER51-I. Its closest relations are PRIMA41, MER84, MER66, HERVFRD, HUERSP2 and HUERSP3, in Gag, Pro and Pol. Its envelope is however related to those of HERVH and HERV9. It is also related to HERVRB (a term initially used in our classification, later abandoned, see below).

Taxonomic markers:

Chaingenus: C; PBS: R; Znf: 1; Frameshifts: 0,1 -1;-1 1 0; Gpatch-; DU-; GPY/F_chromodomain+

AutoFrame hits:

Gag (15 found): HERV4-I

Pro (7 found): HERV4-I

Pol (12 found): HERV4-I, GGLTR7-INT

Env (6 found): HERV9, BAEV, ERV1N-2_SSC-I

ISD; lQNRsepDLLaaEkGcLClfLE, LQNRRGLDtLTAEKGGLCLSvE, LQNnRRLDfLaaekggicLFLK, ieSLAvVaLzNRRGL.

Manual ISD Consensus; LQNrrglDlLAAEKGGlCLflE

Two Env subgroups;

Herv4_a_lqnrrgldlltaekgglclflee

Herv4_b_lqnrrglglleanrrglcifrke

**LTR39 (1nc), taxorder 10770:**

LTR39 is the LTR. It belongs to the MER4I group.

Taxonomic markers:

Chaingenus: C; PBS: R?; Znf: ?; Frameshifts: ?; ?; Gpatch-; DU-; GPY/F_chromodomain-?

AutoFrame hits: No puteins.

**5.2.2.8. The HEPSI supergroup, taxorder 10800:**

These terms are introduced in this paper. However, the LTRs of the HEPSI groups are highly related to LTRs Prima4_LTR (with internal seq Prima4), and MER101. Internal sequences are most similar to Prima4, ERV24_prim, ERV24B and PRIMAX-int, sometimes also to ERV1-1-EC (EC=Equus caballus). Env is often labelled MER70 by AutoFrame. However, according to RepBase MER70 is an ERVL-like, ERV Class III element. MER70a LTRs are similar to MER54 and MER52. MER101 is weakly related to MER65C and MER87, and belongs to the MER4I group. It is a contradiction in the sense that many members of that group are typical gammaretrovirus-like (Class I). As shown in this paper, HEPSI groups are related to both Class I (Epsilonretroviruses, Gammaretroviruses) and somewhat more distantly to Class III (HERVL and HERVS) proviruses. They branch off at the base of the HERV Pol tree (Fig 3a, main text). In this tree, they also cluster with MER89, MER66 and MER34 (Class I). The latter two are members of the MER4I group. We suggest that the classification problems for the MER4I group stems from recombination. MER4 relations are complicated. These proviruses are highly defective and often did not yield a Pol putein. The MER4 group of RepBase contains chains here classified as Class I, either HERVFRDlike, MER50like or HEPSI; but also as Class III. In RepBase MER21 and MER31 are both classified as belonging to Class I and Class III. Their detailed classification requires work with a larger number of proviruses than the present collection.

As mentioned in the main text, Tarlinton´s group distinguished three primate Epsilon-like ERV groups, PE1, PE2 and PE3. By comparison of chromosomal location, we established that out of 59 HEPSI supergroup members, 22 proviruses were the same as identified by Tarlinton. PE1 proviruses included PRIMA4, HEPSI2, HEPSI3, HEPSI4 and MER4; PE2 HEPSI1; and PE3 MER34; proviruses (data not shown).

In RepeatMasker (RM) based Simages, the dominating hits for the HEPSI chains were MER57 (here classified as MER50like), PRIMA4 and PRIMAX. Using the AutoFrame mechanism, it was shown that where a hit was found, HEPSI1-4 Gag was most similar to PRIMA4 and ERV24 Gag, their Pros to PRIMA4 and ERV6, and their Pols to CARERV4 and ERV1-2i_XT. The Envs of HEPSI1 were most similar to MER70 and ERVIP10B3, of HEPSI2 to PRIMA4 and MER70, of HEPSI3 to MER70 and of HEPSI4 to MER89. This was also seen in the tree of Env subgroups (Fig. 6-7).

A special case was chain 4152, here classified as "HEPSI2". In the grand Pol tree (Fig. 3a), it clustered with Ovex1, an avian provirus (36) which in its turn is errantiviruslike. The Repeatmasker Simage of chain 4152 was rather homogeneously PRIMA4, a HEPSI2like entity. This illustrates the intermediate position of the "HEPSI" supergroup (Fig. 2 and 3a of the main text) and that more work is needed to dissect the relations within it and its relations to other groups.

**HEPSI1 (13nc), taxorder 10810:**

The term is introduced in this paper.

Taxonomic markers:

Chaingenus:C; PBS :L, (W); Znf: 1; Frameshifts: 0,1; 0; Gpatch-; DU-; GPYF/_chromodomain+

AutoFrame hits:

Gag (9 found): PRIMA4-INT, ERV24_PRIM

Pro (1 found): PRIMA4-INT

Pol (10 found): CARERV4, ERV1-2-I_XT

Env (3 found): MER70-INT, HERVIP10B3

ISD; LdNqiALDhLLA (MER70-INT)

Two envelope subgroups,

Hepsi1_a_ldnqlaldyllaeegsvsaatfi

Hepsi1_b_medhavldllfaqagglclvlnk

**HEPSI2 (4nc 2c), taxorder 10820:**

The term is introduced in this paper

Taxonomic markers:

Chaingenus: C; PBS: nd; Znf: 1; Frameshifts: 0;0; Gpatch-; DU-; GPY/F_chromodomain+

AutoFrame hits:

Gag (1 found): ERV24_PRIM

Pro (2 found): ERV24_PRIM, PRIMA4-INT

Pol (5 found): CARERV4

Env (2 found): PRIMA4-INT, MER70-INT

Manual ISD; LQNcmALDivTAAQEGTCLiiK (PRIMA4-INT), LnNRiALDYLLA (MER70-INT), thus two kinds of envelope.

Two env subgroups:

Hepsi2_a_lnnrialdyllakqggvctvart

Hepsi2_b_lqncmaldivtaaqegtcliikt

**HEPSI3 (5nc 1c), taxorder 10830:**

The term is introduced in this paper.

Taxonomic markers:

Chaingenus: C, cs; PBS:L, k; Znf: 1; Frameshifts: 0;-1; Gpatch-; DU-; GPY/F_chromodomain+

AutoFrame hits:

Gag (1 found): PRIMA4-INT

Pro (2 found): ERV6_MD, ERV24_PRIM

Pol (5 found): CARERV4

Env (2 found): MER70-INT

ISD; LnNRiALncLLA, LdNciALDYLLG.

Manual ISD Consensus LnNrIALdyLLa.

One Env subgroup, Hepsi3_a_lnncialncllgkqwcvcgvahv

**HEPSI4 (2nc), taxorder 10840:**

The term is introduced in this paper. A small group of severely damaged elements.

Taxonomic markers:

Chaingenus: C; PBS: ?; Znf: 1; Frameshifts: 0,1;0; Gpatch-; DU-; GPYF_chromodomain-

AutoFrame hits:

Gag (1 found): ERV24_PRIM

Pro (1 found): ERV24_PRIM

Pol (1 found): CARERV4

Env (1 found): MER89_INT

Manual ISD; LnNRitLDYLLA

One Env subgroup;

Hepsi4_a_lnnritldyllavqgnvcgivnn

**MER65 (1nc 1c), 10852:**

The term derives from RepBase.

Its LTR is MER65C. The internal sequence is highly similar to ERV1-6 EC-I (*Equus caballus*).

Taxonomic markers:

Chaingenus: C; PBS: L; Znf: 1; Frameshifts: 0; 0; Gpatch-; DU-; GPY/F_chromodomain(+)

AutoFrame hits:

Gag (0), Pro(0), Pol (1 found): CARERV4

Env (1 found): MER70-INT

Manual ISD; LaNRiGLDYLLA

One Env subgroup;

Mer65_a_lanrigldyllaklvfvqzltzz

**MER21 (1nc), taxorder 10852:**

The term is from RepBase.

Its LTR is MER21, similar to MER34, MER39, LTR29, LTR48 and LTR49. Its internal sequence is MER21-INT. It is classified as Class III by RepBase, here as Class I. Its classification status should be further investigated.

Taxonomic markers:

Chaingenus: C; PBS: L; Znf: ?; Frameshifts: ?; ?; Gpatch-; DU-; GPY/F_chromodomain-

AutoFrame hits:

No Gag, Pro, Pol or Env puteins.

**MER34 (2nc), taxorder 10860:**

The term is from RepBase.

Its LTR is MER34. Similar to MER39 and LTR29. Its internal sequence is MER34-INT. Member of the MER4I group.

Taxonomic markers:

Chaingenus: C; PBS: Q?; Znf: 1; Frameshifts: ?; 0 1; Gpatch-; DU-; GPY/F_chromodomain-?

AutoFrame hits:

Gag (2 found): MER34-INT

Pro (2 found): ERV1-1-I_DR

Pol (2 found): MER34-INT

Env (0 found)

**MER61 (4nc), taxorder 10870:**

The term derives from RepBase.

Its LTR is MER61C. Its internal sequence is MER61-int. It belongs to the MER4I group.

Taxonomic markers:

Chaingenus: CB, S, B; PBS: K; Znf: 0?; Frameshifts: 1; -1 0; Gpatch-; DU-; GPY/F_chromodomain-

AutoFrame hits:

Gag (0 found)

Pro (0 found)

Pol (1 found): No Autoframe hits

Env (1 found): MER52-INT

One Env subgroup was attempted, but it did not fulfill EnvQual criteria;

**HERV24 (1nc), taxorder 10872:**

The term comes from RepBase, where it is called ERV24-PRIM.

The LTR is LTR24_PRIM. HERV24 is related to the HEPSI clades of this paper.

Taxonomic markers:

Chaingenus: C; PBS: W; Znf: ?; Frameshifts: ?;?; Gpatch-; DU-; GPY/F_chromodomain-

AutoFrame hits:

Gag, Pro, Env (0 found):

Pol (1 found): CARERV4

**MER4 (13nc), taxorder 10880:**

The term comes from RepBase. See "The MER4I group".

The LTR is MER4. The internal sequence is MER4-INT.

Taxonomic markers:

Chaingenus: C, sc, cd; PBS: W, r, h; Znf: 1; Frameshifts: -1 ; -1; Gpatch-; DU-; GPY/F_chromodomain-

AutoFrame hits:

Gag (3 found): MER52-INT, MACNERV6A, LTR25-INT

Pro (3 found): MER52-INT, ERV1-2-I_BT, CARERV1

Pol (1 found): CARERV4

Env (0 found)

Definition of two Env groups, Mer4-a and Mer4_b, was attempted by manual reconstruction, but none of them fulfilled the Env criteria in the EnvQual program.

**MER4i group, here placed among the HEPSIs:**

This is a RepBase/Repeatmasker term for a group of related gammaretroviruslike sequences. In the RepeatMasker collection of May 2012 it covered:

HUERSP2, HUERSP3, HUERSP3b, LOR1, LTR8, LTR9, LTR19, LTR23, LTR24, LTR25, LTR26 (especially similar to LTR8 and LOR1), LTR27, LTR28, LTR29, LTR34, LTR35, LTR36, LTR37, LTR38, LTR39, LTR43, LTR44, LTR45, LTR48, LTR49, LTR51, LTR56, LTR59, LTR60, LTR68, MER4, MER21, MER31, MER34, MER39, MER41, MER49, MER50, MER51, MER52, MER57, MER61, MER65, MER66, MER67, MER72, MER83, MER84, MER87, MER92, MER101 and PABL.

Although not listed as such in RepBase, PRIMLTR79 and LTR31 should also belong to the MER4I group.

**MER89 (1nc), taxorder 10881:**

The term comes from RepBase.

Its LTR is MER89. Its internal portion is MER89-I. It belongs to the MER4I group.

Taxonomic markers:

Chaingenus: C; PBS: ?; Znf: ?; Frameshifts: ?; ?; Gpatch?; DU?; GPY/F_chromodomain+

AutoFrame hits: No Gag, Pro or Env puteins. Pol(0 hits)

**PRIMA4 (4nc 3c), taxorder 10882:**

The term derives from RepBase.

Its LTR is PRIMA4-LTR, PRIMA4-int is the internal sequence. Highly related to the HEPSI groups.

Taxonomic markers:

Chaingenus: C, b; PBS: (W); Znf: 2, (1); Frameshifts: 0 -1; -1 0 1; Gpatch-; DU-; GPY/F_chromodomain+

AutoFrame hits:

Gag (4 found): PRIMA4-INT

Pro (2 found): PRIMA4-INT

Pol (6 found): PRIMA4-INT, CARERV4

Env (3 found): PRIMA4-INT

Manual ISD; LQNhmAsDiLTAAQrGaCtIiK

One Env subgroup;

Prima4_a_lqnhmaliltaaqggtctvikt

**LTR38 (1nc), taxorder 10883:**

The term comes from RepBase.

Its LTR is LTR38. Its internal portion is not defined in RepBase. The LTR is related to LTR36 and MER87. It belongs to the MER4I group.

Taxonomic markers:

Chaingenus: C; PBS: I; Znf: ?; Frameshifts: ?; ?; Gpatch-; DU-; GPY/F_chromodomain?

AutoFrame hits: No Gag, Pro, Pol or Env puteins.

**MER31 (1nc), taxorder 10884:**

The term is from RepBase.

The LTR is MER31. Similar to MER67. Its internal sequence is MER31I. It belongs to the MER4I group.

It is classified as Class III by RepBase, here as Class I. Its classification status should be further investigated.

Taxonomic markers:

Chaingenus: C; PBS: L; Znf: ?; Frameshifts: ?; ?; Gpatch?; DU?; GPY/F_chromodomain?

AutoFrame hits:

Gag, Pro, Pol, Env: No puteins

**5.2.2.9. Supergroup HUERSP, taxorder 10900;**

**HUERSP1 (3nc 1c), taxorder 10910:**

Term was introduced by Harada (37).

Its LTRs are LTR8, LTR35, LTR73, LTR19b and LTR19c. Referred to as HUERSP1, HERV35I and LTR25-INT in RepBase (in the latter case it uses LTR19b and LTR19c). Alternative names are HERV-P and HURRS-P.

Taxonomic markers:

Chaingenus: C; PBS: P, w; Znf: ?; Frameshifts: ?; ?; Gpatch-; DU-; GPY/F_chromodomain-?

AutoFrame hits:

No Gag, Pro, Pol or Env puteins were predicted.

**HUERSP2 (nc12 c10), taxorder 10920:**

Term was introduced by by Harada (37).

Its LTRs are LTR1b and LTR28. Referred to as HUERSP2 or MER52-int in RepBase.

Taxonomic markers:

Chaingenus: C, cs; PBS: P; Znf: 1; Frameshifts: 0 1 -1; -1 0 1; Gpatch-; DU-; GPY/F_chromodomain+

AutoFrame hits:

Gag (16 found): MER52-INT, GYPSY-10_DEU (MHR-NC portion; HtSLypdSvKrZlTLKDkCitqAApnIRrKLqEqALEpSITLEnLlkvAtSvFyNRnReaqekegeNRKEaeAvmATTqahKPNNpzgaPVnCYrCGkpGHFS), GYPSY-60_CQ (MHR-NC portion; PLKytklsmIDQGfDENptaFLeRLgEafvKHtslspDsVEgzLilKGkfitqAApDIRrKLqkQalgPdsTLenlLKVatSVfydrdrkaqErdrKYRKetealMatrQaHZVQnSqGTpvnCYKCGKPwHFkkNCPgsmRKppR), LTR25-INT

Pro (6 found): MER52-INT, MDOERV-3 (Whole of Pro putein)

Pol (15 found): MER52-INT

Env (2 found): LTR1A2, MER84-INT

Manual ISD; LdNsLvLDYvLA (MER84)

One Env subgroup;

huersp2_a_ldnslvldyvlaeqegictvtnt

**HUERSP3 (nc40 c16), taxorder 10930:**

The term was introduced by Harada (37).

Its LTRs are LTR9A-D and MER61A-F. Referred to as HUERSP3 or MER83 in RepBase.

Taxonomic markers:

Chaingenus: C, cd, cg; PBS: P; Znf: 1; Frameshifts: 0 1 -1; 0 -1 1; Gpatch-; DU-; GPY/F_chromodomain+

Autoframe hits:

Gag (41 found): LTR25-INT, MER52-INT

Pro (18 found):MDOERV3, MER52-INT, ERV24_PRIM

Pol (32 found): MER52-INT, LTR77-INT

Env (12 found): MER83A-INT, LTR19-INT, HERV9 (sec. integr. x3),

IDS: QNRwRvmDvLTAEvGGTCAlLN (MER83), aQNRzALDvLTAEvGGaCsvLN (MER83), aQNRRGLDvLTAEvGGTCALiN (MER83), LQNzRGLDLLmASQGGICvFLk (HERV9, sec integr), mQNRqALDiLmA(MER83), LzNRkGLDLLTAEKGGLCLFLE(HERV9), LQNRRzLDLsaAEKGGLCfFLK (HERV9).

Manual ISD Consensus: aqnrRaLDVLtAEVGGtCallN (MER83); LQNRrGLDLltAEKGGLClFLe (HERV9)

Three Env subgroups;

Huersp3_a_aqnrraldvltaevggtcallne

Huersp3_b_lqnrrzldlsaaekgglcfflke

Huersp3_c_lqnrkgldllmasqgglcvflek

**MER52 (nc24), taxorder 10940:**

The term is from RepBase.

Its LTRs are primarily LTR27D, LTR27E and MER52. However, LTRs belonging to other MER4I elements also occur. MER52 shares fragments with LTR20, LTR25, LTR27, LTR28 and MER61 repeats. LTR25-int seems to be related. It overlaps with HUERSP2.

Taxonomic markers:

Chaingenus: C; PBS: P; Znf: 1, (2); Frameshifts: -1 0 1; -1 0 1; Gpatch-; DU-; GPY/F_chromodomain+

AutoFrame hits:

Gag (16 found): MER52-INT, LTR25-INT

Pro (10 found): MER52-INT

Pol (20 found): LTR77-INT, MER52-INT

Env (0 found)

**LTR25 (3nc), taxorder 10950:**

The term is from RepBase.

Its LTR is LTR25. Its internal sequence is LTR25-INT. It is related to LTR19, HUERSP3, MER52 and LTR27. LTR25 belongs to the MER4 group.

Taxonomic markers:

Chaingenus: C, cb; PBS: P; Znf: 1; Frameshifts: (1); (-1); Gpatch-; DU-; GPY/F_chromodomain+

AutoFrame hits:

Gag (2 found): LTR25-INT, MER52-INT

Pro (0 found):

Pol (1 found): MER52-INT

Env(2 found): BAEV, LTR25-INT

Manual ISD; LQNcRGLDLLTAnQGGICvFLK

Two Env subgroups;

Ltr25_a_lqncngldlltanaggiavglfe

Ltr25_b_lqncrgldlltanqggicvflke

**5.2.3. Class II, betaretrovirus-like**

**5.2.3.1 Supergroup HML (Human MMTV Like), taxorder 20000**

**General remarks regarding HML proviruses:**

Certain combinations of HML components were common among the noncanonical HML chains (where the figures refer to one of the 10 HML groups):

HML2 chains (n=17): 2-3-4-7-8-9, 2-3-4-7-9, 2-3-7-8-9 (7 instances), 2-3-7-9-8 (2 instances), 2-1-7-8-3-9, 2-3-1-9-8, 2-1-3-8-9, 2-1-3-9-8-7, 2-3-1-8-7-9, 2-1-8-7 and 2-3-1-8-7-9 .

HML3 chains (n=2): 3-1-9-2-8-7 and 3-9-1 (Fig 1D).

**HML1 (46nc 9c), taxorder 20010:**

The term derives from Medstrand et al (38), and Andersson et al (39). In the latter paper, the term HERV-K(HML1), or simply HML1, is suggested.

Its LTR is LTR14A,B. Equivalent to HERVK14 in RepBase.

Taxonomic markers:

Chaingenus: B, c; PBS: K; Znf: 2, (1); Frameshifts: -1 0 1; -1 1 0; Gpatch+; DU+; GPY/F_chromodomain-

Autoframe hits:

Gag (40 found): HERVK, HERVK9, GYPSY-12_TCA-INT (long; QlZIMHLIhcFlsTptssppasDhqzIVWaSRaRgLhslhtSvGPlvpLyAlLShLFDSaglHsphSLELglitpTFlapnmRqrtpqEGtLeCVKEdasseDtRgrlkEaqQEszaLGrtrvtmaqSEsKhSaQDVLysfLhtImLMQcQtaIrplTgkvqLAEcIKacDgIgGNlhKANLlaqamaglkvGKnmphfLGhCFNCgqfGHrkkECRkgnqkakttiNQqksPsvCpqCkKGnHwanqChskfskdgQPlsgNgKRglpQApQqtkaypaqpVplqtY, rvnr 1112), LTR25-INT (recomb w HERVWlike seq, rvnr 1236 and 1795), COPIA-1_GDE (recomb w HERVWlike seq, rvnr 3037; SGgsGkgkawAnQmpNRACFqCglqGHFKKDClNRnKppP), GYPSY-16_ANO-I (recomb w HERVWlike seq; rvnr 3038; PpnPkaKpPLRcIlrIrSRvhePFSvSDLkqiKiDlgklSDnPdgytdVLQGLGqSfdlTWrDtmlLLdqtvTSnERsAavtalrvwrSLvsQVndrlTTeEreqfptGqQavPgVDPhWDTesEygdwcsGHL), PLATERVK2A

Pro (41 found): HERVK, HERVK9, CFERV1 (recomb w HERVWlike se, rvnr 1236), MACERVK2, HYLERV9 (recomb w HERVWlike seq, rvnr 1795, 3038), HERVL66 (ERVL!), ERVIIA_ME (ERVK)

Pol (26 found): HERVK, HERVK11D, HERVK13, HERVK9 (recomb?)

Env (10 found): HERVK11, HERVK, HERVK9

ISD; RQTVIzMrDhIvSLKskiQmzC, cQTVIWMGDhIlSLETRiQmRC, hQTVIWMGvRIMSLEnRiQIQC, cQTVtzMGDRIiSLETRiQmQC, pQTVIWMGDcIiSLETRiQmQC, RQTVIWMrDRLMSLKyLFQLQC, LqQTVmWLGDhI (recomb? rvnr 6164), NLLKSIKTLGGsVIS

ISD Consensus: rQTVIWmgdriiSLeriQmQC

hml1a_ivnqindlcqtviwmgdriisle

hml1b_ladqindlqqtvmwlgdhivsle

hml1c_lanqindlrqtviwmrdrlmslk

**HML2 (70nc 19c), taxorder 20020:**

The first report (HERV-K) was from Ono et al (ref). The HML2 term derives from Medstrand et al (38), and Andersson et al (39). In the latter paper, the term HERV-K(HML2) or simply, HML2, is suggested.

Its LTR is LTR5 or LTR5Hs. It is equivalent to HERVK in Repbase and DFAM (DF0000188).

Taxonomic markers:

Chaingenus: B, c; PBS: K; Znf: 2, (1); Frameshifts: -1 1 0; -1 0 1; Gpatch+; DU+; GPY/F_chromodomain-

Autoframe hits:

Gag (60 found): HERVK, HERVK9, ERV2X1-I_ML, GYPSY-16_DTI-I (Incomplete Gag, Zn fingers KCYNCDQIGHLkKNCpvSnKkEpPDlcpRcKQgKhwasqChSkFdrN),

Pro (56 found): HERVK, TGUERVK9-I (defective element, damaged by sec integr), HERVK9,

Pol (55 found): HERVK, HERVK11D

Env (56 found): HERVK

ISD: RQTVIWMGDRLMSLEHRFQLQC or slight variants of it.

hml2a_lanqindlrqtviwmgdrlmsle

**HML3 (151nc 31c), taxorder 20030:**

The term derives from Medstrand et al (38), and Andersson et al (39). In the latter paper, the term HERV-K(HML3) or simply, HML3, is suggested.

Its LTR is MER9, MER9A, MER9B. Equivalent to HERVK9 in RepBase.

Taxonomic markers:

Chaingenus: B, c; PBS: K; Znf: 2, (1); Frameshifts: -1 1 0; -1 0 1; Gpatch+; DU+; GPY/F_chromodomain-

AutoFrame hits:

Gag (121 found): HERVK9, ERV2-3-I_BT (damaged Gag), ERVB4-6-I_RN (damaged Gag), HERVK13 (damaged Gag), ATCOPIA36-i (Zn fingers)

Pro (163 found): HERVK9, ERV2-5_PCA-I, TGUERVK9-I (recomb?), ERV2-3-I, MACNERVK1

Pol (119 found): HERVK9, HERVK13 (defect in Pol),HERVK11D (defect in Pol) ,

Env (10 found): HERVK, HERVK9, MER70-INT (ERVL!; large defect in Env, either secondary integration or artefactual chain)

Three Env subgroups;

hml3_a_lanqindlrqtvmwlgdqvasle

hml3_b_INQAPNLAITKILAAVDIALMAMMAMAEG

hml3_c <no ISD detected>

**HML4 (5nc 7c), taxorder 20040:**

The term derives from Medstrand et al (38), and Andersson et al (39). In the latter paper, the term HERV-K(HML4) or simply, HML4, is suggested.

Its LTR is LTR13,LTR13A and LTR13_ . Equivalent to HERVK13 in RepBase.

Taxonomic markers:

Chaingenus: B; PBS: K; Znf: 2; Frameshifts: -1 0 1; -1 0 1; Gpatch+; DU+; GPY/F_chromodomain-

AutoFrame hits:

Gag (8 found): HERVK

Pro (6 found): HERVK, HERVK9

Pol (8 found): HERVK13, HERVK

Env (9 found): HERVK, HERVK11D

ISD; cQrVIzLGnRVMNLEHRMQLQC, LhQSVIWLGDRm, LRQSVtWLGDRV, lhQSVIWLGDtV, LcQSVIWLGvRV, LRQSVmWLrDRV, hQSVIWLGDRVtNLEHcMQLQC, LcQSVtWLGDRV

ISD Consensus: lrQSVIWLGDRvNLEHrMQLQC

One Env subgroup;

hml4_a_lanqindlhqsviwlgdrvmnle

**HML5 (69nc 27c), taxorder 20050:**

The term derives from Medstrand et al (38), and Andersson et al (39). In the latter paper, the term HERV-K(HML5) or simply, HML5, is suggested.

Its LTR is LTR22A,LTR22B and LTR22C. Equivalent to HERVK22 in RepBase.

Taxonomic markers:

Chaingenus: B,; PBS: I, K, p; Znf: 2, (1); Frameshifts: -1 0 1; -1 0 1; Gpatch-; DU+; GPY/F_chromodomain-

Autoframe hits:

Gag (32 found): HERVK, IAPEZ-INT, ERV2-1_CHO, ERV2X1-I, MMTV-INT, ERV2X1A-I_ML, ERV2-3-I_BT. ERV2-3-I_TBEL, HERVH48 (Secondary integr), GGERVK10 (damaged Gag), ERV2-2_PVA-I, MMTV_INT, HERVK11D, RNERVK22, PLATERVK1

Pro (28 found): ERV2-2-I_BT, ERV2-3-I_BT, ERV1_MD, ERV41_MD, IAPEY3-INT, GIBRV1

Pol (8 found): All Pols are damaged, only short matches were found. IAPEY-INT, HERVH (Secondary integr), ERVB2-1-I_MM, HYLERV9-5_LTR (four chains with same pattern, with HERV9 in 3´third. Rvnr 5049, 5231, 5242, 5280)

Env (0)

ISD was initially not detected, later a highly conserved ISD-like sequence was manually discovered.

Seven Env subgroups;

hml5_a_LLLQQGIDQKILACLQALEAALE

hml5_b_LLLQQGTDQKIPASLQALEAVLE

hml5_c_LLLeQGtDQkiLArLQaPeAALe

hml5_d <no ISD detected>

hml5_e_LLLQQGIDKKIIAHLKPLR

hml5_f_LLLQQGIDQKILAHLQALKASLE

hml5_g_LLLQQGIDQKILACLQALLAALE

**HML6 (48nc 17c), taxorder 20060:**

The term derives from Medstrand et al (38), and Andersson et al (39). In the latter paper, the term HERV-K(HML6) or simply, HML6, is suggested.

Its LTR is LTR3A,LTR3B and LTR3B_. Equivalent to HERVK3 in RepBase.

Taxonomic markers:

Chaingenus: B, c; PBS: K; Znf: 2; Frameshifts: 0 -1 1; -1 0 1; Gpatch+; DU+; GPY/F_chromodomain+

(The GPYF variant in HML6 is GPV)

Autoframe hits:

Gag (40 found): ERV2X1-I_ML, HERVK9, ETNERV, MYSERV_RN, MACERVK1, HERVK9, ERV2-4_TSY-I, PLATERVK1, ERV1_MD, GIBRV1, ETNERV3, ERV2-1N_EC, HERVK, ERV2-3-I_BT, HERVI, ERV2-1_CHO, IAPLTR3, MURERV4

Pro (38 found): TGUERVK8-I, RNERVK22, GGERVK1, PLATERVK1, TGUERVK5-I, MMTV-INT, ERV2-2-I_BT, MURERV4-19, IAPEY_INT, IAPEY3_INT, ERV2-3-I_BT, ERVB2-1-I_MM, RNERV14, TGUERVK6-I, HERVK9, ERV2-1-I_BT, ERVLB4 (!), PLATERVK1, MYSERV6, ERV2X1A-I_ML, ETNERV, GIBRV1, SRV-M-INT

Pol (2 found): MMTV-INT (both damaged)

Env (33 found): HERVK, MACERVK1, HERVK11, ERV147_MD, ERV1_MD. There was evidence for several kinds of hml6 envelope.

ISD was discovered manually, later incorporated into the Env detection algorithm.

Three Env subgroups:

hml6_a_lknklnteihmevamlkttvlwl

hml6_b_lqnkintelqtevamlkstvlwl

hml6_c_lqnkintelqtevamlkttvlwl

**HML7 (5 nc 9c), taxorder 2070:**

The term derives from Medstrand et al (38), and Andersson et al (39). In the latter paper, the term HERV-K(HML7) or simply, HML7, is suggested.

Its LTR is MER11D. Equivalent to HERVK11D in RepBase.

Taxonomic markers:

Chaingenus: B, c; PBS: ?; Znf: 2, (1); Frameshifts: 0; -1; Gpatch-; DU+; GPY/F_chromodomain-

AutoFrame hits:

Gag (3 found): HERVK11D

Pro (3 found): HERVK11D

Pol (12 found): HERVK11D, HERVK,

Env (9 found): HERVK11D

ISD; RQTVIWmADRiISLEHRlQmQC, RQTVmzVGDRIMSLEHRlQmQC, RQTVtWMGDkIMSLeHrLQMQC, RQTVIgMGDRIMSLEHRspMQC, LQqTVIWMGDRI, RQTiIWMrDRIMSLeHrLQMQC, RQTVtWMGDRIMSLEHRLQmqC, RQTiIWMGDRIkSLEHglQMQC, LRQTVIWMGDRI, RQTVIWMGDRiitlehrlqmqc

Manual ISD Consensus: lRQtviWMgDRImSLeHrlqmqC

One Env subgroup;

hml7_a_lanqindlrqtviwmgdrimsle

**HML8 (24nc 34c), taxorder 20080:**

The term derives from Medstrand et al (38), and Andersson et al (39). In the latter paper, the term HERV-K(HML8), or simply, HML8, is suggested.

Its LTR is MER11A,MER11B and MER11C. Equivalent to HERVK11 in RepBase.

Taxonomic markers:

Chaingenus: B; PBS: K, r?; Znf: 2, (1); Frameshifts: 0; -1; Gpatch+; DU+; GPY/F_chromodomain-

AutoFrame hits:

Gag (31 found): HERVK11, ERVB4-6-I (defective Gag)

Pro (32 found): HERVK11, IAPEY3-INT

Pol (32 found): HERVK11, HERVK11D, HERVK13, HERVK9, MMTV-INT

Env (38 found) : HERVK11, HERVK

ISD; LRQSVIWLGDRV, LRQSVIWLGDQV, LRhSVIWLGDRv, LqKSVIWLGDRV, LRQSVIWLGDwV, LRQSVIWLzDQV, LRQSVIWLGDRv, RwSVIzLGDwVvSLKnQMQkQC, RQSVIcrGDwViSLKyhMQmQC, LRQSVsWLGDwV, RQSgIWLGDwLMSLEHhmqmqC, LRQSiLWLGDQL, LRQSVIWLaDRV, RzSVIWLGDRLMSpEHgMKmQC, qQTVtWLGDzaASLgyRMQLkC

Manual ISD consensus: lrqSViWLgdrvSLkhhmqmqC

Three Env subgroups;

hml8_a_lanqindlrqsviwlgdrvvsle

hml8_b_lanqindlrqsviwlgdwvvsle

hml8_c <ISD was not detected>

**HML9 (9nc 10c), taxorder 20090:**

The term derives from Blikstad et al (40, 41). The term HERV-K(HML9), or simply HML9, is suggested.

Its LTR is LTR14C. Equivalent to HERVK14C in RepBase.

Taxonomic markers:

Chaingenus: B; PBS: K, e; Znf: 2, (1); Frameshifts: -1 1; 0 -1 1; Gpatch+; DU+; GPY/F_chromodomain-

AutoFrame hits:

Gag (12 found): HERVK, ERV41_MD (defective)

Pro (14 found): HERVK, TGUERVK8-I, ERV2-5_EE, HERVL66 (!), ERV2-3-I_BT, MACNERVK1, ERV2-2-I_BT

Pol (5 found): HERVK

Env (8 found): HERVK

ISD; cQTVIWMGDRIMNLEHRIQmqC, LRQTVIWMGERI, cQTVIWMGDwIMNLEHRIQmqC, LhQTVIWMGDQI, hQTVpzMGDzIiNLEHRiQIQC, hQTVIWMGDQIMSLQHRIQkqC, hQTVIWMGDRIMSLEHRIQmqC, sQTVIziGDRIMSLEHRlQmQC

Manual ISD Consensus: LhQTVIWMGDrIMsLEHRIQmqC

One Env subgroup;

hml9_a_lanqindlhqtviwmgdrimsle

**HML10 (7nc 2c), taxorder 21000 :**

The term derives from Blikstad et al (40, 41). The term HERV-K(HML10), or simply HML10, was suggested.

Its LTR is LTR14. Equivalent to HERVKC4 in RepBase. HML10 is not homogeneous. The two canonical chains from chromosome 6 cluster with the noncanonical HML10 in nucleotide alignments. In Pol alignments, the HML10 members cluster in two separate groups.

Taxonomic markers:

Chaingenus: B; PBS: ?; Znf: 1?; Frameshifts: ?; ?; Gpatch-; DU-; GPY/F_chromodomain-

AutoFrame hits:

Gag (1 found): HERVK

Pro (1 found): HERVK

Pol (9 found): HERVK11D, HERVK

Env (9 found): HERVK

ISD; RQTVIzMEDhIMSLEHRlQIQC, cQTVIWMGDwIMSLEHRiqKQC, cQTVRWMGDRIMNLEHRiqMQg, RQTVIWMrDRIiSLEHRLQmqC, sQTVIziGDRIMSLEHRlQmQC

Manual ISD Consensus: rQTVIWMgDrIMSLEHRlqmQC

One Env subgroup;

hml10_a_lanqindlrqtviwmgdrimsle

**5.2.4. Class III, Spumavirus-like**

**Supergroup HSERVIII, taxorder 30000**

**HERVL (75nc 86c), taxorder 30100:**

The term is from Cordonnier et al in Heidmann´s group (42). It occurs in RepBase.

Its LTRs are MLT2A1, MLT2A2 and MLT2B.

Taxonomic markers:

Chaingenus: S, cs, bs, ce, cb, bd; PBS: L, k, w; Znf: 0, (1); Frameshifts: -1 0 1; 0 -1 1; Gpatch-; DU+;

38 out of 163 have a low-scoring GPY/F_chromodomain motif in Pol (often with GQRF instead of GP(YF)), 17 out of 163 have a dUTPase in the C terminus of Pol, erroneously placed in an Env putein by Rete (see below).

Autoframe hits:

Gag (1 found): ERVL-B4

Pro (54 found): ERVL-B4

Pol (53 found): ERVL-B4, MERVL-2A

Env (17 found; see below!): TGULTR1-I, ERVL-B4, ERV3-1_PCA, MER84-INT

ISD; LdNqLALDzLLA (MER84-INT; rvnr 4244)

A special note on HERVL Env: ReTe predicted Env in 17 HERVLs. However, T Heidmann and his group, together with JB, showed at the Missilac conference in June 2014 that these were probably misinterpreted by ReTe. These Env puteins are instead composed of a large portion of IN in Pol which contains dUTPase + an additional sequence from the post-dUTPase sequence interpreted as part of the putein by ReTe. The relatively intact reading from of dUTPase, plus a sequence encoding hydrophobic amino acids just preceeding the PPT, simulating the TM5 motif, mislead ReTe.

However, an envputein which does not include dUTPase, was predicted for chain 4244, a noncanonical HERVL:

isPStsSSLSHLRilKLGKKLYSdFFLYHLVLLVLNIqGSLGHqHDSQifPYYiASAnnLSECYITTGTPrLGaqGpVCTFGKvvSTLECCHGNLTTYTCCClPEKFSAYGLhGSRSzNQLLLzSSTHLRKzKILLTLLcTPSSsNiSSSnKKYKFHLNSRGPsVIVRRLPLPLGSQLLHGLNFrIPTRVCASPKYVVICGSPHhhLPnSSSAATfSSpLLvvdhPAVDNEIHiGQCTTGVMeYSeiTIhNTTChYsRPKMSLGmLlARglAVSWLtr*EWGKAlyVhEDTLInTTQTKNLASNTrKvIQNiQpSfNrLANVVLdNqLALDzLLAKztRVCvitNTtCCTwVKpSMEIEaKIkQifKqeeRPhsfVLTkssskdiWSaiKSvLFNhTLFLLgLLVMILfLIFLTPvFrLRMRLEAIKLQrVITQgyeQLgLQP*rdnqIYlRiaRERfCssnLSSDNthKhqEvApkvgPLpLsSrqe

Underlined are probable TMstart, ISD, C-rich area post ISD, and hydrophobic transmembrane stretch, respectively. Their presence indicates a Class I ERV. Italics indicate the portion which is similar to the Class I Gorilla ENVV2 (Genbank ID KC010510). Thus this HERVL seems to have an ERV Class I envelope, like HERVS, HERVL32 and HERVL66.

Another (canonical) HERVL chain (rvnr 775) has both a dUTPase (italics) and a TGULTR1L1-i (a zebra finch ERVL)-like (underlined) start of the predicted Envputein:

iaSDqdiHsvakeggqWAQtHGIhWsYHVPHHpeAtGmIEwwnailksklhhqlgdstlqgwskvlqkavfvlnqhliygtvspiarihrsrkqgvevevapltitpsdppakfllpvpm*TLhSAdLEVLvpzggmLPpGDTTrIlsnzKLRLPPGYFGLLlPLSQQAKKrVTVLAGVIDpDyZdEIsLLLHNkGKEEnaWNtaDPLgcLLvL*pcpvikingklqqpnpgrttndlpsgmtsglttddfwnevlghstrkkphyllrcllkakgiqngeznkvvinnsydhvtshrnedcnwvlsisssfvknmfvhvytctkkissfyflsslscdirfidftsafkyczlfnsigvgdwcisgctkescimldvimtlslsffedcmisgdvyrikltrgglvmvntvcplegykvlilgvsvrvlpkeinfelvgwhrqthpzsgwaqsnhllaqleykqaakcekkpvlafqptsfshvgcflperqtpsssvlelglvllaprpvdgllwdlzscelilnkliyiltciythiynyvyihniyiyiyiyiplvlslwrsltntgsylidegstlmnqsppssptsnaitlgiristykfwgytkthfiayazfzhkktffsckqrciltyfdeivqhittlllkrsmtslvlyzifhlivfrlfvfstislyhdalncvg

Matches are shown with upper case. ReTe artefactually started the Env putein at the 3´end of pol. The sequence tailing the dUTPase of rvnr 775 is of uncertain significance.

The chain 3636, a canonical HERVL, has the predicted envputein:

lprlpsmdsrnalsimifhtalpltkelalwlkkc~~gsgtvfpiarihgsrnqgvelevapltitpgdplakflfpvpai~~LrSAGLEVLvpeggmLPpGDTTrIqLnWKLRLPPGHFGLLlPLSQzAKKaVTVLAGVtDpDyQDaISLLLHNGGKEKYaWNtGDPLgrLLVL~~pcpvikvngklqqpnslanpgrttndpdpsgmkvcatppgkkpqpaevlaedkrntewvve~~gvintiydhvtscrkedcnwheyfllpllqtclcmytlvkeissfyflsplscktrfieftsafkyltlcnsiqigdw~~cisgctkdsciklgiimtllvfmisgdvygfkltrgtlmmantgcqld~~wiegykvlilgvsarvlpkrltfesvg

The stretches with overstrike are highly similar to the C terminus of Pol of MuERVL.

The AviERVIII of galGal3 (36) has a short relatively open stretch at the position of Env.

It is similar to the C terminus of MuERVL Pol (GenBank ID Y12713).

DTYSGYGFAFPARNASAKTTIHGLTECLIYRHGIPHSIAS*~~DQGTHFTAREVRQWAHDHGIHWSYHVPHHPEAAGLIERWNGLLKMQLQHQLGGNSLEGWGRVLQKAVYALNQRSIYGTVSPIARIHGSRNQGV~~EKGIVPLTITHSDPLGKFLLPVPITLGSAGLEVLAPEAGVLLPGATTNIPLNWKLRLPPGHFGLLMPLNQQAKKGITVLGGVIDPDYHGEIGLSLHNGGKQHYVWSVGDPLGRLLVLPCPVIKVNGKLQQPNPSRMTKDADPSGMKVWVNPPGKEPRPAEVL*AEGEGNTEWVVEEGSYKYQLRLRNQLQKRGL

Italics show the similarity to AviERVIII_GalGal3_EnvTrace. Underlined is the identity to MuERVL dUTPase. Overstricken is the similarity to the C terminal end of WDSV Pol. Thus, also AviERVIII has a C-terminal dUTPase portion of Pol. The presence of a dUTPase in the C terminus of pol seems to be an ancient feature of some class III ERVs.

In conclusion, the most likely explanation for predicted envelopes of the majority of HERVL is an artefactual premature start of ReTe reconstruction inside pol, including dUTPase. One noncanonical HERVL, whose internal structure is complicated, has a Class I env. The ReTe predicted sequence after the dUTPase is of uncertain significance.

Three Env groups;

Hervl_a (artefact)

Hervl_b (artefact)

Hervl_c ldnqlaldzllakztrvcvitnt (rvnr 4244)

**HERVS (4nc 16c), taxorder 30200:**

Term is from Tristem (43). It does not occur in RepBase.

Its LTR is LTR18. HERVS is equivalent to HERVL18. It is related to HERVL32 and HERVL66. It is similar to AviervIII and Birddawg I (Fig. 6-7). Like this group of bird viruses (36), it is an interclass recombinant, and has a Class I (gammaretrovirus-like) ERV envelope. The envelope is most similar to that of PRIMA41 (Fig. 6-7), as described in this paper. It shares similar envelopes with two other Class III ERVs, HERVL32 and HERVL66 (see the AutoFrame hits).

Taxonomic markers:

Chaingenus: C, S, sc, cd, cb; PBS: S; Znf: 0; Frameshifts: 1 -1 0; -1 0 1; Gpatch- (1 weakly scoring); DU-; GPY/F_chromodomain-. Chains have neither dUTPase in C terminus of Pol, nor erroneously placed in Env.

AutoFrame hits:

Gag (0 found):

Pro (10 found): GGERVL18, CARERVR1

Pol (10 found): GGERV10­­_RT

Env (11 found): TGULTRK2D, PRIMA4-INT, HERVL66

Env is in 6 cases most similar to HERVL66 and in 4 cases most similar to PRIMA4 Envs.

ISD; LQNzmALnivTAAzGGTCAlLG, LQNqmALDiiTtARGGTCslLG (TGULTRK2D), LrNRmvLDivTAAzGGTCAlLG (PRIMA41), LQNzmAfDiiTAAQGGTCAlLG (PRIMA41),

LQNwmALDivTAdQGGTCALtg (HERVL66), wQNRmALgiiTAAQGGTCvFLG (HERVL66)

Manual ISD Consensus: LQNrMALDIITAAQGGTCALLG

One Env subgroup;

Hervs_a_lqnrmaldivtaaqggtcallgt

**HERVL66 (1nc), taxorder 30210:**

The term is from RepBase. The provirus is also called MER66.

Its LTR is similar to that of GGERV10. HERVL66 is highly similar to HERVS.

HERVL66 envelopes occur in HERVS. Prima41-int can also have HERVL66 envelopes.

Taxonomic markers:

Chaingenus: C, B; PBS: T?; Znf: 0?, (1); Frameshifts: ?; 0; Gpatch-; DU-; GPY/F_chromodomain-

AutoFrame hits:

Gag (0 found)

Pro (1 found): ERV3-1-I_XT

Pol (0 found):

Env (1 found): HERVL66

ISD; LQNqmALDmLTtAQGGVCALLh (This is reminiscent of a gammaretroviral TM sequence.)

One Env subgroup;

hervl66_a_lqnqmaldmlttaqggvcallht

**HERVL32 (1nc), taxorder 30220:**

The term is from RepBase. HERVL32 is similar to, but separate from, HERVS.

AutoFrame hits:

Gag, Pro, Pol 0 found.

Taxonomic markers:

Chaingenus: CD; PBS: T?; Znf: 0?; Frameshifts: ?; ?; Gpatch-; DU-; GPY/F_chromodomain-

AutoFrame hits:

Env (1 found): PABL-B

ISD not found initially, however manual inspection later identified an ISD.

One Env subgroup;

HERVL32_a_LQNRmAmDtLiAAQGRTyALiKT

**LTR57 (1nc), taxorder 30230:**

The term is from RepBase.

Its LTRs are LTR47A, LTR47B and LTR57. Its internal portion is LTR57-INT or HERV57I. It is related to both HERVS and MER4I. Do not confuse with MER57.

Taxonomic markers:

Chaingenus: C; PBS: L; Znf: 0?; Frameshifts: ?; ?; Gpatch-; DU-; GPY/F_chromodomain-.

Neither dUTPase in C terminus of Pol, nor erroneously placed in Env.

AutoFrame hits:

Gag, Pro and Env: no puteins

Pol (1 found): LTR57-INT

**MST, MLT and THE (MaLR sequences), taxorders 30300, 30400 and 30500, respectively:**

We mention these chains for the sake of completeness only. Twenty-nine chains were judged to be MaLR. However, 9 of them were classified as possible Rete artefacts. Either, the simage twentieths were mainly in antisense, or the chain was concluded by an unrelated element. Thus, on one hand there was evidence for ReTe artefacts, on the other many of the 29 chains had a rather large number of weakly scoring motif hits, involving Gag, Pro, Pol and Env. 8 of the 30 had zinc finger hits, most with bona fide zinc finger sequences.. A few chains had features tentatively suggestive of internal MaLR sequence: rvnrs 4861 and 3058 had Gagputeins with weak similarities over the MHR region of retrotransposons in the Gypsy database. Rvnr 5922 had a weakly MuERVL-like Pol putein. It clusters with HERVL32 and HERVS. A further exploration of the MaLR elements is outside of the scope of this paper.

Two Env groups detected;

msta_ (probable artefact)

thea_ (probable aretefact)

**5.2.5. Other retrotransposons**

**Uncertain Errantilike (2nc)**

The term is introduced here.

These two low-scoring chains were based on several weak motif hits in Gag and Pol but both had strong NC (zinc finger) hits.

Chain 5484 (chr X; chain score 415): The chain starts with a predicted 5´LTR followed by a PBS (tggaactattaaatcagt) most similar to the Glycine tRNA of Cereba, *H vulgare*. The NC1 hit was most similar to a zinc finger from Peabody, *P sativum*, the NC2 hit was most similar to a zinc finger from Cer1, *C elegans*. The chain then included weak hits in protease PR3, in integrase IN3, a typical PPT and a predicted 3´LTR 71% identical to the predicted 5´LTR. A Gag putein was reconstructed (gagscore 0.90 [max 1.00], with two shifts and 9 stops, classified as gypsylike by ReTe). The evidence that the chain is retroviral is relatively strong. A search at Genbank showed that the putein, and the DNA sequence of the whole chain, is part of the gene encoding CCHC zinc finger protein 13 of humans (ZNF 13; NP_976048) it is also known as ZNF 45 and KOX5. Acording to RefSeq annotation (NM_203303), this gene appears to represent an intronless retrocopy of a related multi-exon gene located on chromosome 3, i.e. where chain 1114 and the CNBP gene reside.

Chain 1114 (chr 3; chain score 310) starts with a weakly predicted Gag start by neural network, two strong NC hits and weak RT2 and IN5 hits. NC1 was similar to a zinc finger from Gypsy element MarY1 from *T matsutake* , NC2 was most similar to a zinc finger from Gypsy element Peabody from *P sativum*. A Gag putein was reconstructed (gagscore 0.97 [max 1.00], with six shifts and two stops, classified as gypsylike by ReTe). The evidence that the chain is retroviral is moderate. A search at Genbank showed that the putein, and the DNA sequence of the whole chain, is part of the gene encoding CCHC zinc finger protein 9 of humans (ZNF 9; AY329622), also named "Cellular Nucleic acid Binding Protein", CNBP (44-49) (RefSeq NM_001127194) .

In protein and nucleic acid alignments, ZNF 9/CNBP and ZFN13 proteins were highly similar to each other (data not shown). It is thus likely that both have a retroviral origin. Previous observation of zinc finger containing genes of possible retroviral origin are (50).

Taxonomic markers:

Chaingenus: G; PBS: ? (tggaactattaaatcagt); Znf: 2; Frameshifts: ?; ?; Gpatch- (1 weakly scoring); DU-; GPY/F_chromodomain-. Thus, they neither had dUTPase in the C terminus of Pol, nor erroneously placed in and Env.

Autoframe results:

Gag (2 found): GYPSY6_NVI-I (5484; NVI=*Nasonia vitripennis*), GYPSY_DG-I (1114; DG=*Drosophila grimshawii*). Both hits were nearly exclusively based on the two zinc fingers. A BLASTP search at low stringency in Genbank revealed relatively weak similarities to several retroviral gag proteins (Visna and lentivirus of small ruminants), but not to Gypsy proteins. The *Errantivirus* representation is not strong in Genbank. However, a BLASTP search at the Gypsy database, version 2.0, gave a similar result (Visna and lentivirus of small ruminants).

Pro, Pol, Env (0)

In conclusion, it is likely that both chains are of retroviral origin, but the clade is uncertain. They were provisionally labelled as "Uncertain Errantilike".

**5.2.6. Clades which are not represented in the present classification**

**HERVP71 (0):**

Class I, Its LTRs are LTR71A, LTR71B. Internal sequence HERVP71. It is highly similar to HERVADP (see above).

No members

**HERVRB (0):**

Class I. The term was introduced by Tristem (51) (Genbank id AC004045). Approximate equivalents are MER51A, MER41B, PABL_A, PABL_B (LTRs), HERV4-1-i and PABL_B-int. For this paper, it was first included as a canonical group, but was later split into HERV4, PABL and ERV1_ARTIODACT in order to maintain homogeneity within the groups.

No members

**HERVV and HERVPB (0)**

The terms were introduced by Villesen (3, 52).

Class I. They were not detected by RetroTector. They contain little else but the *env* gene.

**LTR77 (0):**

A RepBase term.

Class I, related to LTR20

**MER55 (0):**

A RepBase term.

Class I, but also ERVL-related. Its LTRs are LTR33. Internal sequences are MER55.

**MER71A (0):**

A RepBase term.

Class III, HERVL-like, its LTR is LTR16A

**MER71B (0):**

A RepBase term.

Class III. Its LTR is MER16D or MER16B

**HERVL16 (0)**:

A RepBase term.

Class III. Internal sequence is HERVL16.

Literature references for this List

1. Elfaitouri A, Shao X, Mattsson Ulfstedt J, Muradrasoli S, Bölin Wiener A, Golbob S, et al. Murine gammaretrovirus group G3 was not found in Swedish patients with myalgic encephalomyelitis/chronic fatigue syndrome and fibromyalgia. PloS one. 2011;6:e24602.

2. Borysenko L, Stepanets V, Rynditch AV. Molecular characterization of full-length MLV-related endogenous retrovirus ChiRV1 from the chicken, Gallus gallus. Virology. 2008;376(1):199-204.

3. Aagaard L, Villesen P, Kjeldbjerg AL, Pedersen FS. The approximately 30-million-year-old ERVPb1 envelope gene is evolutionarily conserved among hominoids and Old World monkeys. Genomics. 2005;86(6):685-91.

4. Lavie L, Medstrand P, Schempp W, Meese E, Mayer J. Human endogenous retrovirus family HERV-K(HML-5): status, evolution, and reconstruction of an ancient betaretrovirus in the human genome. Journal of virology. 2004;78(16):8788-98.

5. Villesen P, Aagaard L, Wiuf C, Pedersen FS. Identification of endogenous retroviral reading frames in the human genome. Retrovirology. 2004;1:32.

6. de Parseval N, Lazar V, Casella JF, Benit L, Heidmann T. Survey of human genes of retroviral origin: identification and transcriptome of the genes with coding capacity for complete envelope proteins. Journal of virology. 2003;77(19):10414-22.

7. de Parseval N, Heidmann T. Physiological knockout of the envelope gene of the single-copy ERV-3 human endogenous retrovirus in a fraction of the Caucasian population. Journal of virology. 1998;72(4):3442-5.

8. Herve CA, Forrest G, Lower R, Griffiths DJ, Venables PJ. Conservation and loss of the ERV3 open reading frame in primates. Genomics. 2004;83(5):940-3.

9. Fei C, Atterby C, Edqvist PH, Ponten F, Zhang WW, Larsson E, et al. Detection of the human endogenous retrovirus ERV3-encoded Env-protein in human tissues using antibody-based proteomics. J R Soc Med. 2014;107(1):22-9.

10. Martin J, Herniou E, Cook J, Waugh O'Neill R, Tristem M. Human endogenous retrovirus type I-related viruses have an apparently widespread distribution within vertebrates. Journal of virology. 1997;71(1):437-43.

11. Blomberg J, Benachenhou F, Blikstad V, Sperber GO, Mayer J. Classification and nomenclature of endogenous retroviral sequences (ERVs): problems and recommendations. Gene. 2009;448:115-23.

12. Snyderman R, Cianciolo GJ. Immunosuppressive activity of the retroviral envelope protein P 15E and its possible relationship to neoplasia. Immunology today. 1984;5(8):240-4.

13. Nelson M, Nelson DS, Cianciolo GJ, Snyderman R. Effects of CKS-17, a synthetic retroviral envelope peptide, on cell-mediated immunity in vivo: immunosuppression, immunogenicity, and relation to immunosuppressive tumor products. Cancer immunology, immunotherapy : CII. 1989;30(2):113-8.

14. Cianciolo GJ, Lostrom ME, Tam M, Snyderman R. Murine malignant cells synthesize a 19,000-dalton protein that is physicochemically and antigenically related to the immunosuppressive retroviral protein, P15E. The Journal of experimental medicine. 1983;158(3):885-900.

15. Mangeney M, de Parseval N, Thomas G, Heidmann T. The full-length envelope of an HERV-H human endogenous retrovirus has immunosuppressive properties. The Journal of general virology. 2001;82(Pt 10):2515-8.

16. Leib-Mosch C, Brack R, Werner T, Erfle V, Hehlmann R. Isolation of an SSAV-related endogenous sequence from human DNA. Virology. 1986;155(2):666-77.

17. Brack-Werner R, Barton DE, Werner T, Foellmer BE, Leib-Mosch C, Francke U, et al. Human SSAV-related endogenous retroviral element: LTR-like sequence and chromosomal localization to 18q21. Genomics. 1989;4(1):68-75.

18. Werner T, Brack-Werner R, Leib-Mosch C, Backhaus H, Erfle V, Hehlmann R. S71 is a phylogenetically distinct human endogenous retroviral element with structural and sequence homology to simian sarcoma virus (SSV). Virology. 1990;174(1):225-38.

19. Levy LS, Lobelle-Rich PA, Elder JH, Payne S, Montelaro RC. An unusual retrovirus-like sequence identified in human DNA. The Journal of general virology. 1990;71 ( Pt 7):1613-8.

20. Kabat P, Tristem M, Opavsky R, Pastorek J. Human endogenous retrovirus HC2 is a new member of the S71 retroviral subgroup with a full-length pol gene. Virology. 1996;226(1):83-94.

21. Repaske R, Steele PE, O'Neill RR, Rabson AB, Martin MA. Nucleotide sequence of a full-length human endogenous retroviral segment. Journal of virology. 1985;54(3):764-72.

22. O'Connell C, O'Brien S, Nash WG, Cohen M. ERV3, a full-length human endogenous provirus: chromosomal localization and evolutionary relationships. Virology. 1984;138(2):225-35.

23. O'Brien SJ, Bonner TI, Cohen M, O'Connell C, Nash WG. Mapping of an endogenous retroviral sequence to human chromosome 18. Nature. 1983;303(5912):74-7.

24. Maeda N, Kim HS. Three independent insertions of retrovirus-like sequences in the haptoglobin gene cluster of primates. Genomics. 1990;8(4):671-83.

25. Kannan P, Buettner R, Pratt DR, Tainsky MA. Identification of a retinoic acid-inducible endogenous retroviral transcript in the human teratocarcinoma-derived cell line PA-1. Journal of virology. 1991;65(11):6343-8.

26. Blond JL, Beseme F, Duret L, Bouton O, Bedin F, Perron H, et al. Molecular characterization and placental expression of HERV-W, a new human endogenous retrovirus family. Journal of virology. 1999;73(2):1175-85.

27. La Mantia G, Maglione D, Pengue G, Di Cristofano A, Simeone A, Lanfrancone L, et al. Identification and characterization of novel human endogenous retroviral sequences prefentially expressed in undifferentiated embryonal carcinoma cells. Nucleic acids research. 1991;19(7):1513-20.

28. Seifarth W, Baust C, Schon U, Reichert A, Hehlmann R, Leib-Mosch C. HERV-IP-T47D, a novel type C-related human endogenous retroviral sequence derived from T47D particles. AIDS research and human retroviruses. 2000;16(5):471-80.

29. Lyn D, Deaven LL, Istock NL, Smulson M. The polymorphic ADP-ribosyltransferase (NAD+) pseudogene 1 in humans interrupts an endogenous pol-like element on 13q34. Genomics. 1993;18(2):206-11.

30. Mager DL, Henthorn PS. Identification of a retrovirus-like repetitive element in human DNA. Proceedings of the National Academy of Sciences of the United States of America. 1984;81(23):7510-4.

31. Widegren B, Kjellman C, Aminoff S, Sahlford LG, Sjogren HO. The structure and phylogeny of a new family of human endogenous retroviruses. The Journal of general virology. 1996;77 ( Pt 8):1631-41.

32. Benit L, Calteau A, Heidmann T. Characterization of the low-copy HERV-Fc family: evidence for recent integrations in primates of elements with coding envelope genes. Virology. 2003;312(1):159-68.

33. Seifarth W, Skladny H, Krieg-Schneider F, Reichert A, Hehlmann R, Leib-Mosch C. Retrovirus-like particles released from the human breast cancer cell line T47-D display type B- and C-related endogenous retroviral sequences. Journal of virology. 1995;69(10):6408-16.

34. Fukagawa T, Sugaya K, Matsumoto K, Okumura K, Ando A, Inoko H, et al. A boundary of long-range G + C% mosaic domains in the human MHC locus: pseudoautosomal boundary-like sequence exists near the boundary. Genomics. 1995;25(1):184-91.

35. Blusch JH, Brack-Werner R, Werner T. A pseudoautosomal boundary-like element adjacent to the SSAV1 locus at 18q21. DNA sequence : the journal of DNA sequencing and mapping. 1999;10(2):115-9.

36. Bolisetty M, Blomberg J, Benachenhou F, Sperber G, Beemon K. Unexpected diversity and expression of avian endogenous retroviruses. mBio. 2012;3(5):e00344-12.

37. Harada F, Tsukada N, Kato N. Isolation of three kinds of human endogenous retrovirus-like sequences using tRNA(Pro) as a probe. Nucleic acids research. 1987;15(22):9153-62.

38. Medstrand P, Blomberg J. Characterization of novel reverse transcriptase encoding human endogenous retroviral sequences similar to type A and type B retroviruses: differential transcription in normal human tissues. Journal of virology. 1993;67(11):6778-87.

39. Andersson ML, Lindeskog M, Medstrand P, Westley B, May F, Blomberg J. Diversity of human endogenous retrovirus class II-like sequences. The Journal of general virology. 1999;80 ( Pt 1):255-60.

40. Blikstad V, Benachenhou F, Sperber GO, Blomberg J. Evolution of human endogenous retroviral sequences: a conceptual account. Cellular and molecular life sciences : CMLS. 2008;65(21):3348-65.

41. Blomberg J, Benachenhou F, Blikstad V, Sperber G, Mayer J. Classification and nomenclature of endogenous retroviral sequences (ERVs): problems and recommendations. Gene. 2009;448(2):115-23.

42. Cordonnier A, Casella JF, Heidmann T. Isolation of novel human endogenous retrovirus-like elements with foamy virus-related pol sequence. Journal of virology. 1995;69(9):5890-7.

43. Tristem M. Identification and characterization of novel human endogenous retrovirus families by phylogenetic screening of the human genome mapping project database. Journal of virology. 2000;74(8):3715-30.

44. Abe Y, Chen W, Huang W, Nishino M, Li YP. CNBP regulates forebrain formation at organogenesis stage in chick embryos. Developmental biology. 2006;295(1):116-27.

45. Lombardo VA, Armas P, Weiner AM, Calcaterra NB. In vitro embryonic developmental phosphorylation of the cellular nucleic acid binding protein by cAMP-dependent protein kinase, and its relevance for biochemical activities. The FEBS journal. 2007;274(2):485-97.

46. McGrath CF, Buckman JS, Gagliardi TD, Bosche WJ, Coren LV, Gorelick RJ. Human cellular nucleic acid-binding protein Zn2+ fingers support replication of human immunodeficiency virus type 1 when they are substituted in the nucleocapsid protein. Journal of virology. 2003;77(15):8524-31.

47. Rajavashisth TB, Taylor AK, Andalibi A, Svenson KL, Lusis AJ. Identification of a zinc finger protein that binds to the sterol regulatory element. Science. 1989;245(4918):640-3.

48. Sato SM, Sargent TD. Localized and inducible expression of Xenopus-posterior (Xpo), a novel gene active in early frog embryos, encoding a protein with a 'CCHC' finger domain. Development. 1991;112(3):747-53.

49. Taylor FM, Martindale DW. Retroviral-type zinc fingers and glycine-rich repeats in a protein encoded by cnjB, a Tetrahymena gene active during meiosis. Nucleic acids research. 1993;21(19):4610-4.

50. Shigemoto K, Brennan J, Walls E, Watson CJ, Stott D, Rigby PW, et al. Identification and characterisation of a developmentally regulated mammalian gene that utilises -1 programmed ribosomal frameshifting. Nucleic acids research. 2001;29(19):4079-88.

51. Tristem M. Identification and characterization of novel human endogenous retrovirus families by phylogenetic screening of the human genome mapping project database. Journal of virology. 2000;74:3715-30.

52. Villesen P, Aagaard L, Wiuf C, Pedersen FS. Identification of endogenous retroviral reading frames in the human genome. Retrovirology. 2004;1:32.
