# Supplementary material for: Classification and characterization of human endogenous retroviruses; mosaic forms are common
Source: Retrovirology. 2016 Jan 22;13:7. doi: 10.1186/s12977-015-0232-y (PMC4724089; doi:10.1186/s12977-015-0232-y)
Supplement: Supplementary file 3 — 10.1186/s12977-015-0232-y HERV group consensus and best representative sequences. Its contents are explained in the beginning of the list. [file 12977_2015_232_MOESM3_ESM.docx]

Supplementary list S3;

**Nucleotide and protein sequences from consensuses ("con") and best single chain representatives ("bre") in FASTA format.**

The FASTA names contain: Clade name, taxonomic ordering number, chainid (=rvnr; consensuses have numbers >100000), type of sequence, and retroviral gene (chaindna or putein). Chaindnarm= repeat masked chaindna.

**HERVT**

>hervt_10110_100018_con_chaindnarm

tgaaatgcctaaccttgtttttactctaactcattactttgaattttgtcctgcttgtctctttaatcacctatccttgctttaaaatgcctaaccttgtttttataaataactttgtactcctagactttccttttcttttaatcacttagccttgtttctacctgaattgactttctattagctaagagagctagacagactttatcttagctttttcactgtcAgtcccttcataaaggActtaactatgtgcaagtatgActcttagcacatctaagaatgcAgtTaactcAtaAgatactgtgGtgagcAatacccacAGccCCtaggaatttgtccgattGataatgcccaaagCCCctgcgTCTaTcACtTTgTgatAgtcTTaAAGCCCtTGcACCTgGAACTGTTTAtTTTtCCTGTAACcATTTatgTaaccattaacCTTTTAACTTTTTtGcCtacTTTaCTTCTGTAAAAaaTTgTTTtAaCTAgaCcCCccccccaTCCCCTTtCTAAACcaAaGTATAAAAGaAAATCtAGCCCCTTCtTCGGGGCCgAGAGAAtTTTgAGcgtTAGCCaTCTCTttGGtCGCCaGCTaAATAAAgggActCTtAATTcgTCTCAAAGTGTGGCaTTTTcTCTAtaACTCGCTcgGgTACAACATTTttGGAGGCCCCAGcGAGAtattaaCgCCACcGGGtGAGAGCCGGaCTCgCTCCgGGCTCCCCCgGAaaGACGGCcGGCTTgTAGGGGgGGcGCCACCTGAagaaAgAaTTTtCAgGGtCCCcgAaagGtgACtGTCTTCCaGAGGaGAgCGGATCGACTACcGtGTGtGTGCCCacaAAAATTCaACttCTGAGTCCTCAgcTTCTGacCCCGgGgTcAGGTAaGTCAGATtTGACTTcgtttgcaatgggaggcgacgggcgtagacgcggaatctctgtcttcagtTTCTgattctggggtacagtaggtcggattctgctgtgtttcccgaaaaagggtaacggtcagtctaattgtgctgagtcctcagcttctgaccccggggtcaggtaggtcggatgtgacttcgtttccggtgagaggggagcggcacaaacgaagacatatatgactcagtttctatgcgcgggaaaatgtttttagattgctacttctgtttctgTaaGAGGGaAGcGGCCCTGAtgAGGGcgTCCctCTTTtGACTCaGcCCaTtaCTCtAGGAcgCTaGagGgtaGAGcCcTGGTTTtCTGtTAGGCaccTcTGTgTCTcttTcTagGggaagcagccctggGAAGtggccctgacaggGacCCTCcctctttttgacTcaGtccacatcCcAGgAtGCTggaGgactgaGtCttGgTtTCTggCagaCcctctatcactcttggTCactctcTCTCTCTCTCTCtctctctctctcttttTCTATCTCTcaTCCTTCTCTTGTTCAGGTtTCttGGAGAaTcTccgtgaAAgAAAAaAaaAAAAaaacTGTTATAAACTCTGTGTGAATGaGTGAGTGAATGtGtgattgtgGAGgaCAAGGGCTTGCGCTTGtacTTCCAGTTTGTAGCTCCAtGGCGAAAGCTACGGAGTTCGAGTGGGCCCTCACCTGCGGTTCCgTGGtGACCTCATAAGGCTTAAGGCAGCATCgGGCATAGCTCGATCcGAGCCGGGGGTTTATACCgGCCTGCCAATGCTAAGAGGAGCCcAAGTCCCCTCAgGGGGAGCGGCCAGGCGGGCATCTGACTGATCCCATCACGGGACCCCCTCCCCTTGTCTGTCTAaaAAAaaaaaaaaaaaaaaaggaaaaAACTGTCATAAtTGTTTAcATgCCCTAgggtcaaattgtttgttttatgttttattgttctgttCAGTGTCTATTGTCTTGTTTAGTggtTGTCaAAGTTTtgtATGTCAGGtCgTCGATAtTGCCCAAGACGtCTGGGtAAaAACTTCTTCAAGGTCCTtAGTgCTGATTTTtTaTCACAGGAGGTtAAATTTCTCATCAgTCATTTaGGCTGGCCAcCaCAGTCCTGtCTTTTCTGtCAGAAACAAaTCAGGTGTTGTTAcGGGAAaGAGTGTGgAAAACATTcgCCTatgtgggaaatatttgcccGaTTGGGATTTCTGGCACCATaAAGGTTGgCtGGtATTTAGATTGcCATACCCCACGtCCaAgTGAcTGGACCaCCTCtaaatTAaACcAGTGGTGGaTTCAAAAtAGCCACCCTGCAGAcCTCCTTGCTCACCTtTTcTGTCATtCTGTAACTTTTCCtGTGCCCTTAAATAgGaCACTGTGtAGAGAAACCTACGCCCgTACTGCTTTACTtCgTtTagaggtctcaggcgtgtctctttgggctgtcaggcccaggggcgaggcacgcctccaccagtttacaaaagaaacaaGAcTCTTAtTCTgTggcaggggctccctgacctgtgggcaaggactggagggtgacgacgccggatcccctcccccggcacccccTCCTCTGTGGCTccctcaggtgcccgACTCTCCcACCTTAagttgtcaaagcccctgacttcccagagcaccagcgcatccgcacccagctcagaatgcaagggcccagcctgctgcagtgaacttggtcccaggccaggcttctgagctgggcttgcagcgtctgacagggcctggaggcagggcgggggaccgcagagtggccctggtgacccagagctgcccggcatggctcctcctagcctagAAaGATCCGAGTGGtCCcTTTtCCtCCTCATCCCtgtCCCtTACCCCGCACATCTCgTTTTCCtcctcctcgtccctgcccccctaccccgtacatctctttttccaGTGtcACAGCAAGTtCAGCGTCTCCAAGACTTGGCTCTGCTCTCaCTCCTTAAACCCTTAAAAGAAAAgGCtgAGTTTGAACTtTTTGCCTTTgAGTCaTGGAGACACCAAAAaTATTTAGGcTATAGGTCaAGAAGAAGAaGgaGagggagAaCaCCTAGaTCcaACTGgCCcAGgAgacCTCaGgCtgGCctcTagTCCtCcTCCCTcaATCTtAAAGCTacAGcAgacagaccttATGTGGCAAGaAGTATTAGCTgTtGTtGTTTTtCTgCTgttctTTCTGGTtATgtTAaTTCTGTTCTTCCgATACTcCAGCCCCCCAGGgaaTGAaTTTCTCTGTCCATGCTGGGTTTAATATcTCTGCTCAAgACCTTgTTAAATTGCCTtCaaataaaaaaaAaAATagGAAtaataaatgggaaACtCTTCtTCCCaGCCTtgTAAAGaTTGGAGCCCTCTCCAATGTATGcTGCAatgAATTTtTCTCTaGGtTTCTCAGAGGATTATGGAGTCCGCCTTAAAAAAGGCAAaCTCCaGACACTCTGTGAAGTAGAATGGCCAAAGTTTGGAgtCGGGTGGCCCCCtGAAGGGTCAtTGAAtCTCACAAtTGTTCAgGCTGTGTGGCgGGTtGTTaCTGgAACTCCCaGcCACCCtGATCAGTTTCCCTACATTGATCAATGGCTAAGTTTGGTCaGGAgCCCTCCTCCATGGCTCCGcTCATGctaaaactctgtaGCCATTCATAATtCtACCTCCAAGGTCCTtcTgAGCCAGACCgCAcatTTTcGCCtCgACCCTCAGCccgtTCgGCTCCtCCTGTAcTGCCTCCCTCTGAAGAAGAGGAgAGTtTtCCTCACCCAGTTCCACCGCCTTAcAACCatCCTGCTCCCctAGAaTCtTCCCtTGTCTCCTCGACtACaTCCCCTGTGGGCtTCtCCaCCcATTGCCTCcCaATTatGgCCgtGGCaggcAGGAGGAaGTAGCCCCtCTcCTcCCACTGAGAGAGGCACAAgTCCCTCcGGGTGATGAGCGCTCAGCtCCcTTCTTAGTTTATGTCCCTTTtTCTACTTCTGACtTGTAtAAtTGGAAaaCTCATAATCCtCCCTTCTCTGAAAAGCCCCAGGtTTTGACCTCACTGATGGAGTCCGTgCTCCGGACTCACCgGCCCACCTGGGATGAcTGtCAaCAgCTCCTTTTAACCCTTTTCACCTCTGAAGAGAGGGAaCGTATCCGAAGAGAgGCCAGAAAGTAcTTCCTtACATCAGCCagTaGaCCgGAgGAGGAAGCctAGAGACCTCCTTGAGGAggagaaatctaaaGGTcTTTCCCTCTACCCgGCCTAAtTGGGACCCAAATTCCTCAAGTgGAAaGAGAGCTTTgGAtGATTTTCACCGGTATCTCCTtGCAgGTATTAAagGAGCcgCTCgGAAACCCATaAAcTTGTCTAAGACaACTGAAGTTGTCCAGGGGCCTGATGAGTCACCAGGAGCGTTTTTAGAAcGCCTCCAGGAGGCtTATCGGAcTTACACCCCTTTTtgtagaaatgactccccattaGACCCGGCgGCtaagtCCcGAAAATAGCCGTGCTCTTAATTTGGCATTTGTGGCTCAGGCAGCCCCtGATATTAAAAGAAAAgcTcCAAAAACTGGAaGGATTTGCTGGaATGAATATCAGTCAGCTTTTAGAAATAGCCCAaAAAGTTTTTGACAATCgAGAGTTTGAAAAACAAAAACAaGCAACaCAGGCAGCTGAAAAGGCtGCTGATAAAGCATccAAAAGACAAGCAAAAATCTTAGTGGCaGCtATCCAaGAAGccAaaAaggAAaGGCCCCCATcaCAGAaaAaTggCCAgGGaacCccgGGTcCccaCcagAAAgGcaAgAaAggtGAacaGGctcCcCTcagaaAaAaAcCAATgtgCtTATTGcAAgCAgActGGaCAcTGGAAaAaGGAaTGCCCatTAatacCAgAaGaAAAAtCAGAAaAgAAAAAggTcCTCACCCTcCCtgcagcgGAGGAgtCTGATGAtTGAcgGGgCcaGGGCTCCcTctctcTtGGCcCcccgggAgCCCATgGTgActGctAcAgTagGggggcCAgCctgtAcgcTTCCTAataGAcACcgggGcggaacAcTcaGtAcTGcAgAcaCCctTggGcAGTGTCTctAATAaaAaaaatagtggctgtacaaggggctActgGAGctATtCAaGAatATCCTGTCACaCACTCacgAGAaGTgAgtTTgGGACAGAAAAGAGTaACcCACTCaTTtCTtgTgGTcCCAGaGTGcCCttTtCccCTatcCTtgGAcgAGACCTgCTcCAtAAGtTacAGGCcTCtATcTCcTTcTCaGCcCAaCAaGCTcAcCTcAtgtTaGGagAcaCaacgcCCCCtACTGCcCaaCTcctGcTAActAcCCcTCTgTcaGAgGAATAtCTttTagTTTCACCaTCAcAAccaCtGGAgaAtAAaActAATcCTCTccTatTGGattTACAGACtCTcTTTCCctgaGTcTgGgCcGAgtCAAaCCccCcaGGACTgGCtAAgCAcCATCtgCCaGtgGTtGTAGAACTCcTGgCCACtgCCttgCcagTCcAGgtaAAaCAaTAtCCtaTGAGTCAGcaGGCTAgagAGGGgATcAAtCCCcAtATTCAgtgACTgTTACaAGCTGGcAtACTcACACCATtGtcAGTCcgCCTGGAAtACtCCATTtTTGCcgGTCCAgAAACCtgGAAcAAATGAtTACcgGCCaGTACagGAcTTaagGGAAGTTAAcAaatggaCaGTtACtgTCCAtCCaACtgTcCCTAAcCCTTATAcTtTACTCAGCCTGCTcCcaCCAGAACAtACAgtaTacACTGTCCTTGACtTaAAgGATGctTTcTTTGCTaTTCCTCTGGcCCCcAAaAgCCaaCCtATcTTTGctTTTGAATGGACAGATCCtgGcTcaGGAgACACcaCCCAAtTgACcTGGACTcagTTaCCtCAagGTTTTAAAAAtTCccCcAccCTTTTTgggGAaGCccTcCAaCAgGATCTTaTACCaTTccgAGCCAGTcacCctAActGtAaCTCTTCTtCAGTAtgTaGAtGAccTtTTAtTaGCTACTGAAACTActGACAGcTGCCTGCAACAtActAggGACCTaCTtTACCTCCTTCAGGAaCTcgGGTAtcggGTCTcagccaAgAAGGcCCAgCTTTGTCtTCCcAGagTgTCCTACcTgGGGTAtGAGAtAAAcaaaGGaaAAAGGGCACTCACcAGTGCtcggAAgGAAgCcATCCTgcgAATCCCCaCTCCCACCACcAAgAGACAGGTAcaTGAaTTcCTGGGgGCcaTGGGATAcTGTcgcCTaTGGATATtgGGGTTtgcgGAgATTGCcAAGCcCcTGTAcACtgCTACAGGAgGaAATGGCCCACTAATTTGGACtGACACaGAaGAACAGGCTTTtCAAAAcCTgAAaAAGGCATTAACTGAaGCCCCTGCTtTAGCCCTagtCCCAAATATCTCAAAgCCaTTTCACCTtTTTGTcCATGAAAGCCAGGGAGTTGCTAAAGGGGTgCcTtACTCAGACTTTAGGACCcTGGAGATGCCCAGTGGCCTATTTaTCTAAGAGACTGGAtCCTGTGGCCTCTGGATGGCCAAGTTGTCTGcGAGCCaTAGCGGCTACAGCAAGCCTaGTCCAAGAGGCTGATAAATTaACTCTAGGCCAAAATTTAACCCTTACAGCTCCTCATGCCGTAGAGACTTTgCTACGAAGTGCTTCTGGCAAATGGATGTCAAATGCTCGCATCTTaCAGTATCAGAGTTTACTGTTAGATCAGCCTCGTTTGACTTTCTCTCCCACAAGGTGTTTAAATCCAGCTACtTTACTCCCTGATCCAGACTTCACTACACCTGTCCATGACTGcCAAGAACTGTTAGAAACTAccGAAACTGGCCGACCTGATCTtCAAGATGTGCCCCTAAAgaAGGCAGATGCCACCGTGTTTACAGACaGTAGCAGCTTCCTTGAGCAGGGAGTACgAAAGGCTGGTGCAGCcGTTACCACGGAGACAGATGTgtTGTGGGCcCAGGCTtTACCAGCaaaCACCTCAGCACAGAAGGCTGAATTGATcGCCCTCACTCAGGCTCTCCGATGGGGTAAGGAcAAACGTATTAACATTTACACTGACAGCAGGTATGCTTTTGCTACTGTGCATGTACATGGAGCCATCTAcCAAGAGCGCGGGCTACTCACCTCAGCAGGAAAgAttATCAAaAACAAAGAAGAAATTtTAGCCCTGCTTGAAGCtgTtTGGCTCCCTCAGCAgGTGGCTGTAATTCACTGCAAAGGACATCAAAaAGAAgACACGGCCATTGCCCGTGGTAACCAaAGAGCaGATTCtGCAGCTCaGGAgGCAGCacGGCTTtCAGTCAcGCCTtTAAaCCTGCTGCCcGCAGTgTCCTTTCCACAGCCaGActTGCCtGACaAcCCcGaATACTCAaCAGAAGAAGtaAAAAACTGGCTTCAGATCTTcAaGCCAaTAAAAATCAGGAAGGTTGGTGGATTCTTCCTGACTCcAGAATCTTCATACCCCGAgCTCTTgGgGAAACTTTAATCAGTCatCTaCATTCTACCACCCATTTagGAGGAGCAAAACTgGCCCAGCTCCTaaGGAGCCATTTcAAGATCCCCCACCTTCAgAgCtTAACAgATCAAGCAGCTCTCCAGTGcACAGCtTGTGCcCAGGTAAATGCCAAGCAAGGTCCTAAACCCAGCCCAGGCCACTGtCTCTGaGGAAACTCaCCAGGAGAAAAGTGGGAAATTGACTTTACAGAAGTAAAACCACACCGGGCTGGGTAcAAATACCTTCTaGTACTAGTAGACACCTTcTCcGGATGGACTGAGGCATTTGCCACCAAAAACGAAACTGCCAcCACGGTAGTTAAGTTTTTACTCAATGAAATCATCCCTCGACATGGGCTGCCTGCTGCCATAGGGTCTGATAATGGACCaGCCTTCACCTCGTCCATAGCTCAGTCaGTCAGTcagtAAGGCATTAAACATTCAATGGAAGCTCCATTGTGCCTATCGACCCCAGAGCTCTGGGCAGGTAGAACGCATGAACCgCACCCTAAAAAatACTCTTACAAAATTAATCTTAGAAACacgGTGAAAATTGGGTAAGtCTCCTTCCTTTAGCCCTaCTTAGAGTAAGaTGCACCCCTTACCaGGCTGGGTTtTCACCTTTTGAAATCATGTATGGgAGGGCtCCGCCTATCTTGCCTAAaGCTAAGgGATaCCCATTTAGCAGAAATATCACAAGCTAATTTATTACAGTACCTgCAGTCTCTCCAACAGGTACAAGAtATCATCCAGCCACTTGTCCGaGGAGCcCATCCCAATCCAGTTCCTGACCAGAtgGGGCCCTGCCACTCtTTCCAGCCAGGTGACCTGGTGTtTGTTAAAAAGTTCCAGAAAGAAGGACTCACTCCTGCTTGGAAAGGACCTCAtACTGTCATCCTCACCACGCCAACaGCTCTGAAgGTGGAcGGCATTCCTGCTTGGATTCATCACTCCCGCATCAAAAAGGCCAACAaAGCCCAGCAAGAAACATGGGTCCCCAAGCCTGGGtCAGGCCCCTTtAAAACTGCGCCTAAGTCaaGTGAAGCCATTAGATTAATTCTTTTTATTTACCTCTcTTGTTTGTTTctGCCTGTTATGTCCTCTGCgCCTTCCTACTCCTTTCTCCTCACtTCTTTCACaACAGGACGTGTATTTGCAAACACTACTTGGAAGGCaGGaACCTCCAAGGAAGTCTCTTTTGCAGTTGATTTATGTGtTCTGTTCCCAGAGCCTGCCCATACCCACGAAGAGCAAcaCAATCTGCCgGTCATAGGAGCAGGAAgTGTCGACCTTGCTGCAGGATTTGGACACTCCgGGAGCCAaACTGGATGTGGAAGCTCCAAAGGTGCAGAAAAAaGGACTCCAAAATGTTGACTTTTACCTCTGTCCTGGAAATCACCCTGACtCTtctAGtTGTCGAGATACTTACCAGTTTTTttCTGCCCTGACTGGACATGTGTAACTTTAGCCACCTACTCTGGGGGgATCAACCaGATCTTCAACTCTTTCCATAACTCGTGCTTCCCATCCTAAACTaTGTACTAgAAAAAATTGTAATCCTCTTAaCTATAACTGTCCATAACCCTAATTCAGCTCAATGGTATTATGGCATGTCATGGGGATTAAGACTTTATATCCCAGGATTTGATGTTGGAACTATGTTCACCATCCAAAAaAAAAaaaaTCCTGGTCTCATGGAGCCCtCCTAAGCCAATCGGGCCTTTAACTGATCTAGGTGACCCTATGTTCCAAAAACACCCaGACAAaGTTGATTTAACTGTTCCtCCACCATTCtTAGTTCCTAAACCcCAGCTgCAAaGACAaCAtCTCCAACCCAGCCTGATGTCcATtCTAGgtGGaGTACATCAcCTCCTtAAtCTCACCCAGCCTAAACTAGaacCCCAAGATTGTTGGTTtaATGcCTAAAttaGCCAAACCCCCTTATTATGcTaGGATTAGGAGTAGAAGCCggACgCTgtatatTAAAaTTGgCTCTCTaTCTTGTCATACAtGACCCCaTGCCCTCACACTAGGAGATGTGTCTGGAAAcGCaataTTCcTGTCTAATTAGcACTGGaGTATAACTTATCTGCTTCTCCcTTTCAGGCTACcTGTAATgCAGTCcCTAacCTTACTTCCTTAAGCACtgttCTCAGTCTCCTACCAGGCACCTAACAATACCTGGTTgGCCTGCACTTCAGGTCTCACTCgCTGCATCAATGGaACTGAACCAGGACCTCTCtTGTGTGTGTTAGTTgCATGTgCTTCCCCAGGTATttaACGTGTACAaGTGGGCCAGAAGGACAACTTCTCATgCgCTCCCCCTGAATTACATcCCAGGTTTCaCCGAGCtGCCCCACTCCTaGTaCCCCTccTGGCTGGCCTTAGCATAGCTGGGTCAGCAGCCATTGGCATGGCTGCCCTGGTTCAaGGAGAAACTGGACTAATGTCCCTGTCTCAACAAGTAGATGCTGATTTAAGCAATCTCCAgTCAGCCATAaATATACTACATaCCCAGGTAGAGTCTCTaGCTGAAGTAGTTCTTCAAAAaCcGCcGAGGCTTAGATCTGCTATTcCTCTCTCAAGGccAGGaTTATGtgCAGCTCTAGGAGAaAGTgTaGTTGCTTgCTAtGCCAATCAgcTCTGGAGTCATctcAAAAGATAgCtCTCCAAAAAGTTCGAGAAAATCTAGATAGAcGCCAaCAaGAACGAGAAAATAACACcCCCTttctcattcctggagtaccatGGTAtCAAAGCATGTTtAACTGGAAtCCaTGGCTAACTACTcTAATCACTGGGTTAGCTGGAccaCCCcTcCTCcTCCTaTTgTTAgGtTTAaTCTTtGGGCCTTGTATaTTAAATTGGTTtCTTAAtTTTgTAAAgCAacgCATAGCTTCTGTCAAaCTTATGTATCTtAaAACcCAATATAACcCCCTTGTTaaTAACTGAgGAATCAAtGATTTGATTCCCCaAAAACAcaAGTGGGgAAtgtgAtaccctaccttgTTttattttcaataacttctctcttttagttgagagatacggacacccttaatcatctaaccttgactcaCtctcacttaaccccttccctagctcagaaaacctgataaActcgactTgtgCaagctgattacaagcccatctaagaatgcaaTacccagataagatttacacggAGttcatctgtgtacgCagttcctaagaAtatcaaaggtcaagaattaaCagatacgctactgaaaccaccaatgtaagaagtccccaggactctgcttttatcactttataacaccataaacccttgcatctgtaactgtttatttttctgtaactatttatattaacaattttaattctatccttctgctaccaatgcttttatagtaatcacttctcttctattctgcttagactattttgAccattataaatatttaaatttctagcactttcttaggactgaaaaattttttcaaattagagccctctctccatCccaaacactaaaaataaaatttaattaggctcagaatttggggtttatagaatattgagcatgagccaactgttaggcggacagcctaataaagaactcataatttatattaaaatatgggatatataataagatacttggaaacaacaggggaccccaagcaatgtgcaatgcagttctccgggtttatgggtgtataagaatttacaccctggccaaacaagttatagatagttgcttaacatgtaaaaaaagctaataagcaggatctaaggaaattgcgccttggtggaagaaatccagcactgagaccatttcaaagtgttcagatcaattacactgaaatgcctccaattggtagttatacttattagtaataatagaccattttacccactgggtagaggca

>hervt_10110_100018_con_gagputein

PLQCMLQNFSLGFSEDYGVrLKKGKLqTLCEVEWPKFGvGWPPEGSLNLTIVQAVWrVVtgTPgHPDQFPYIDQWLSLVRsPPPWLcSCAIHNSTSKVLLSQTaFlPqPSAgSAPPVLPPSEEEESLPHPVPPPYNpPAPleSSLVSSTTSPVGSPPIASqLwPwQEEVAPLLPLREAQvPlGDErSAPFLVYVPFSTSDLYNWKtHNPPFSEKPQvLTSLMESVLwTHrPTWDDCQQLLLTLFTSEERErIqREArKYFLTSAgRPEEEArDLLEEVFPSTrPNWDPNSSgGrRALDDFHwYLLAgIKgAaqKPiNLSKTTEVVQGPDESPGaFLEcLQEAYqTYTPFDPAAPENSrALNLAFVAQAAPDIKRKLQKLEGFAGMNISQLLEIAQKVFDNrEFEKQKQAtQAAEkAADKAsKRQAKILvaAIQEakKEgPpsQstgQGtpgphqKgqKgerapLqknQCAYCKQtGHWKKECPLlPEEKpEkKKvLTLPaaEEsDD

>hervt_10110_100018_con_proputein

wgQGSltLGPqePMVTATVGGQPVrFLIDTGaEHSVLQTPLGSVSNKKVAVQGATGAIQEYPVTHSrEVSLGQKRVTHSFLVVPECPFPLLGzDLLHKLQASISFSaQqahlmlgdtmpptarlllttplseeyllvspsqllgnsanpllldlqtlfp

>hervt_10110_100018_con_polputein

fpylapllasisfsaqqahltlgdttpptaqlllttplseeyllvspsqppenntnpllldlqtlfpzvwaesnppglakhhpPVVvELLATalpvQvKQYPmSQqArEGINPHIQzLLQAGILTPCQStWNTPFLPVQKPGTNDYqPVQDLrEVNKwtVTvHPTVPNPYTLLSLLpPEHTvYTVLDLKDaFFAIPLaPKSQPIFaFEWTDPgsGDTTQLTWTqLPQGFKNSPtLFgEALQQDLiPFrAShpnctLLQYvDDLLlaTETtDSCLQHTrDLLYLLQELryrvSAKKaQLCLpRVSYLGYeInkgkRALTSAqKEaILqlleiaqkvIPtPtTKRQVhEFLGAvGyccLwIlgFaeIaKpLYtaTggNGpLvWTDtEEQAFqNLKKALTEAPALALPNISKpFHLFvHEsQGVAKGVLTQTLGPwrcPVAYLSKRLDPVASgWPSCLzAiaATASLVQEaDKLTLGQNLTLTAPHAvETLLrSASGKwMSNARILQYQSLLLDQPrLTFSPTRCLNPATLLPDPDfTTPVHDCQELLETtETGrPDLQDVPLKeADATVFTDgSSFLEQGVqKAGAAvTtETDvLWAQALPAgTSAQkAELvALTQALrWGKDKRINIYTDSRYAFATVHVHGAIYQErgLLTSAGKiiKNKEEILALLEAvwlPQQVAVIHCKGHQkEdTAIArGNQrADSAAqeAArLpVmPLtLLPAVSFPQPDLpdhPeySpEEeKlasdlqasKNQkgWwILPDSRIFyPqaLgetLisrLHSTTHLgGvKLAQLLrShFKIPhLQdLtnQaaLqCtACAQVNAKQGPKPSpgHcLwgnsPGEKWEIDFTEvKPHrAgYKYLLVLVDTFSGwTEAFATKNETAtTVVKFLLNEIIPrhGLPAAIGSDNGPAFTSSIAsQSVSKALNIQWKLHCAYrPQSSGQVEhMNhTLKsTLTKLILETGENWVsLLPLALLRVRCTPYqAGFSPFEIMYGRAPPILPKLRDTHLAEISQANLLQYLQSLQQVQDIIqPLVrGAHPNPVPDQmGPCHSFQPGDLVfVKKFQKEGLTPAwKGPHTVILTTPtALKVDGIPAWIHHSrIKKANkAQqETWVPKPgpGPLKLcLSqvK

>hervt_10110_100018_con_envputein

mgpqawvrplktapklgeairlilfiylsclflpvmssapsysflltsfttgrvfanttwragtskevsfavdlcalfpepaiphesqttlpvigagvvdLaagfghsgsqtgcgaskgaeKglqnvdfylcpgnhpnascrdtyqffcpdwtcvtLatylGgstrsstlsisrashpklctkrncnpLtITvHnPnsAQWYYGMSWGLRLyIpGFDVGTMFTIQKKILVpWSPPKPIGPLTDLGDPMFQKHPDkVDLTVPpPFLVPKPQLQrQHLQPSLMSILgGVHHLLNLTQPKLAQDCWLCLKAKPPYYVGLGVEATLKigpLSCHTcPhALTLGDVSGNASCLISTGYNLSASPFQATCNQSLLTSLSTSVSYQAPNNTWLACTSGLTRCINGTEPGPLLCVLVHVLPQVYVYSGPEGQLLIAPPELHPRFrRAAPLLVPLLAGLSIAGSAAIGMAALVQGETGLMSLSQQVDADLSNLQSAIDILHsQVESLAEVVLQNrRGLDLLFLSQGGLCAALGESCCFYANQSGVIKDTLQKVrENLDRrQQERENNTPpWYQSMFNWNPWLTTLITGLAGPLLlLLLgLvFGPCILNWFLNFvKQrIASVKLMYLkTQYNPLViTEESmI

**HERVE**

>herve_10210_100008_con_chaindnarm

tggatgggactgcctcttgccacagattattttgctgtcagggatttcaaagagcaaaagggactttgggtaggctggctgcactccatgtttcaggtggtccttttctcattgtgtgggctgaggttgtctgcactttgcaggaggctattgggtcctctgacaggaatcattgaacactgcttcaactccagcacaaggcagcttgttctctcagctgagtcttggatttttccgtgctttcttcaggaatccacgtgccttaatatttcatccaccaaaataaactgaaaaaattaaagattatttaaacctaactgctatggaacagcatttaaaatacaataaattttttaaacaaagaaaaaaaagaatctgcccaaaaacaacacaaggattaaaagaaagtacaattccagagactaataaacaaaaaagattataaaatatgctcaaaggtttcaaaaaaatgaaacctacaataatcatctacttacatcatttaacctaataccattctgtctctgaatgtataataatttattttttctttcaatccaacaaccaaaaagtaaaacgcttaaaattgaggcgagcaacagacagagcagccccattgcctaagcccggaagttaaagaataaacccatgacctaatagcttatgtatatctatagagtacagaatattgtatggaaaagctttgtgaaaatacctgtcctgttctgttacgctctcattaccagtgcatgtacccgcccccagtcacatacgcgcccctgcttgctcaaatcaatcacgaccctttcaagtggactccatttagagttgtaagcccttaaaagggacaggaattgtttcttcggggagctcggctcttgaagacgcgaGtcttgccaatgctcccagctgaaTaaagccccttccttctttaacccttcgGtgTctgaGgggttttgtctgcggcttgtCctgctacattTctTggTtccCtGaccggGaagcgagGTgatTgatgGAccggtcgAggcagccccttAgGcggcTTAggCcTGCCCTGtggAgcaTcccTgcgggggacTccggccAgcttgAGcgacgcggATCctgaGAGcgCTcccgggTAggcAatTgCCccgGTggaAcgccTcgtcagAgcaGtgcgtgGCAggCcCCcgtgGAggATcAacgCAgtggctgaACAccgggAaGGAActgGcacTTggAGTCtgGAcatcTGaAActtGGTAaGaCtgGTctTtGgAAcTtgCCCcACTCcaTtTgAGTGGAAGcaTggcCTgAtCAcCCAcgGtgTGcCtgTactgGcActTtgGTttttgtttTgattcttgacttgacttgaattgcttgatactttGgttttggtttttgacctggcttggatttttagagtactcggatgtttttttcgattctggtttggtggaaactgtaatattgtttgtgtttctgttttaccctttctttgttttgagatgaaaactgaaaaagtgtgtgtggtctagtttgagagtgacgttttgactcagtatttgttctaccgtttgcgtgtggtggtgagattgttgttttgtctcgagagaaAcatggGtcagacacAaAGtAAGcaaccACtCcacTaGgAaCTatgTTgAaaaATtTcaagaaagGatTtaatgGagACtaTgGagttaaggtagcTAtgAcaccAggaaAaCTTAgaACtTtgTgTGAaaTAGAtTggCCagCaTTAGAagTgGGttgGCcATCAGAAGGAAGCcTgGACAGgTCccTtGTTTCtAAGGTaTggCACAaggTAACtggtAAgccaGgacACccaGAcaCAgTTtCcATAcATAGAcaCtTGGTTACagCTggTttTagaccccccCAcaGTGGtTAAgAGGaCAGaaaagCAGcAGcAgTacTAGTaGCAAAGgGAcAGatAGctAAGGAAggatcccgCTCCACCcgCcgAggGAAaTCAgCtCCTaAAGTccTGTccgACCCAaCaTCAGAaGAttcATgGCAggAgaTgGcACcAgtggtcccCcagtggtgccccCcccTtAcCAaGgagagaGGCtccCcacatcctgAGcCCACaGcgcttgcgcctCcacaAgacaaacatAtCCCTAGgggggactCCACCCAGAGTAGAcAAgAgAGGAaGTGAagcagCtcgGGAGAAgACcCCTCCCTTGGcAGCtcgtTTaagaCCcAAaACtGGgAtaCAAATGCCCCTGAGAGAGCAgcggTATACTGGtGaTAGATGAGGAtGGaCAcaTGGTGGgagttcgtgtatcaacccttcacctctgccgatctcttcaacttggAaAGGcgTGctTTTGTGTAcCAgCCcTTCACCTCTGCcgAcCTcCTCAAcTGGAAAAAcAATACcCCATCcTATACtGAAAAGCCtCAAGCTcTAATTGAtTTGCTCCAAACTATTATCCAGACcCAtAAacCCcACtTGGGCTGATTGCCACCAGtTGCTCATGTaCCTCTacTTAACACaGATGAAAGGcGgAGaGTgCTCCAaGCAGCAACTAAGTGGCTaGAgGAaCATGtaCCAGCTGATTAcCAAAAcCCCCAAGAgTATgTAAGgAtcCAgtTaCCAGGAACaGACCCCCAgTGGGACCCAAATGAAAGAgagGaaTATGCAaAGGCTAAacgctcGgTACAGGGAaGCtcTctTgGAAGGaTTAAAGAgggtcccGGaGCtCAgAAGGCCACAAAtGTtAAcAAgGTCTCTGAGGTCATTCAGGGAAAAGAAGAAAGTCCAGCACAATTCTAcGAGAGAgaaCTGTtcaccGTGAGGCCTATCGTATGTATACTCaCCTTTGATCCcGATAGCCCTGAAAAaTCAGCGCATGATTAACATGGCTTTAatgttcacctGTTAGaggacTCAAAGCGCAGAAGACATTAGAAGAAAACTGCAGAAACAgaaatGGCTGGGTTTGCAGGGATGAATACaTCACaAGTTAtTAGAAATAGCttcagaAACCAGGTGTTTGTAAAaaagtgtcCAGGGATGCAGTAAGCCGtAAgGAAAACCGCAAAGAGAAagaaTGAAcGTCAGGCCCGGCGAAACGCCGACCTGcTtgttAGCTGCAGCAATtAGAGGGGTCCCCCcacatcaacCAAAGaGGCAAGGGAAGGGGGGgCCCCcaaaccaaGGGAAAgAaACTCAGCCTGGCTGTtggaCAgAGCTTGCAGCGTAACCAGTGTGCTTaATTGTAAAGAAATAGaaaaagcagcaagcaGACATTGGAAGAACAAATGCCCTCAcGCTaAAAgGAAAACAAGGTGACTCAGAGCAGGAGGCCCCAGACAAGGAgGAAGGGGCCCTGCTCAAcCTGGCAGAAGGGTTATTGGACTGAGGGGGACCGGGCTCAAGtGCCCCCAAAGAGCCtATGGTCAGgATGACAGTtgGGGGGTAAAGACATTGAtTTTCTTGTaGATACCGGTGCTGAACATTCgGTAGTAACCaCCCCGGTtCGCCCCCTTATCCAAAAAGACTATTGAcATaATCGGAGCtgggCACgGGGGTTTCAGCAAAGCAAGCTTTCTGCatgTTGCCcCGGACtTGtACTGaTAGGAgGGACATAAAGTGATTCATCAGTTTTTGTACATGCCTGACTGTCCCTTGCCCTTGTTGGGAtAGGGACTTGCTTAGCAAGCTGAGAGCCtACTATCTCTTTTACAgAGCAcGGCTCTTTgCTGCTAAAGTTACCcGGAACgGGAGTCATTATGaCCCTTACGGTCCCCCGAGAGGAGGAATGGAGACTTTTCTTaAaCTGAGCCaGGCCAAGAGATAAGACCAGCTCTGGCTAAGCGGTGGCCAAGAGTATGGGCAGAAGACAACCCTCCAGGgTTGGCaGTCAACCAAGCCCCcGTACTcATAGAAGTTAAGCCTGGGGgCCCAGCCgGTtAGGCAAAAACAGTACCCGGTCCCCAGAGAAGCTCTTgAAaGGTATCCAGGTCCATCTCAAGCACCTAAGAACTTTTGGAATTATAGTTCCTTGTCAGTCTCCATGGAACACTCCCCTCCTGCCTGTTCCCAAGCCAGGGACCAAGGACTACaGGCCGGTACAGGATTTGCGCTTGGTTAATCAAGCTACAGTGACTTTACATCCAACAGTACCTAACCCGTACACATTGTTGGGattGTTGCTGCCAGCTGAGGACAGCTGGTTCACCTGCTTGGACCTGAAAGATGCTTTCTTTAaGCATCAGATTAGCCCCTGAGAGCCAGAAGCTGTTTGCCTTTCAGTGtttgacttccggatctgGGAAGAaagaTCCGGAGTCAGGTGTCACTACTCAGagtagtgTACACTTGGACCCGGCTTCCCCAAGGGTTCAAGAACTtCCCCCACCATCTTCGGGaGAGGCaTTGGCTCGAGACCTCCAGAAGTTTCCCACCAGAGACCTAGGCTGCGTGTTGCTCCAGTACGTTGATGACCTTCTGCTGGGACACCCCACGGCAGTCGGGTGcGCCAAGGGAACAGATGCCCTACTCCGGCACCTGGAGGACTGTGGGTATAAGGTGTCCAAGAAGAAAaGCTCAGATCTGCaGACAGCAGGTACGTTACTTGGGATTTACTATCCGACAGGGGtcgGAGCGCAGCCTGGGATCAGgaaaaaaaaaaAAAGAAAGCAGGTCATTTGCAATCTACCGGAGCCTAAGACCAGAAGGCAGGTGAGAGAATTCTTAGGAGCTGTGGGGgTTTTGCAGACTGTGGATCCCAAACTTTGCAGTATTAGCCAAGCCTTTGgTATGAGGTCACAAAggtgGGGGGGGGGGACcgGGAACCTctatgagcctcTTTGAATGGGGATCCCAACcAACAGCAAGCCTTTCATGAGTTAAAGGAAgAAACTTATGTCAGCCCCAGCCCTGGGGCTACCtGATCTGACAAAGCCTTTTACATTGTATGTGTCAGAGAGAGAgaAAAGATGGCAGTTGGgAGTTTTAACCCAAACTGTGGGGCCCTGGCCGAGGCCgGTGGCCTACCTCTCTAAACAACTAGACGGGGTTTCTAAAGGATGGCCCCCcaTGTTTGAGGGCCTTGGCAGCAACTGCCaatggacggatcCTGCTAGtACAtaaAGAAGCAGATAAGCTGcaACTCTTGGGagcCAAAACCgtacTGAACATActtAAGGCCCCCCacATGCTGTGGTGACTTTAATGAATACTAAAGGACATCATTGGCTAACGAATGCTAGACTcACCAAGTACCAAAGTTTGCTCTGTGAAAATCCCCGTATAACCATTGAAGTTTGTAcaagtttACACCCTGAACCCCGCCACCTTGCTCCCGGTATCAGAGAGCCCTGTCGAGCATGACTGTGTAGAAGTGTTGGACTCAGTTTACTCTAGCAGACCTGACCTCCGGGACCAGCCTTGGGCATCAGTAGACTGGGAGCTATACGTGGATGGGAGCAGCTTCATCAACCCACAAGGAGAGAGATGTGCAGGATATGCAGTGatgcagtaGTAAaCCCTGGACACTGTTGTTtGAAGCCAGATCGTTGCCCCAGGGCACTTCAGCCCAGAAAGCTGAACTCATTGCTTTAATTCGGGCCTTAGAACTCAGTGAAGGTAAGACTGTAAACATTTACACTGACTCTCGGTATGCCTTTTTAACCCTTCAAGTGCATGGAGCATTATATAAAGAAAAGGGCCTAcctaTTGAACTCTGGGGGAAAAGACATAAAATATCAACAAGAAATCTTGCAATTATTAGAAGCAGTATGGAAACCCCACAAGGTGGCAGTTATGCATTGCAGAGGACACCAGCGAGCTTCCACCTTGGTGGgTTTGGGGAATTCCCGcGCTGACTCAGAGGCTCGAAAAGCAGCATCTaCCCCCTTCCGGGCATCAGTCACAGCcaCCCCCTGCTCCCTCAAGCACCTGATCTTGTACCTACctacTTATTCTAAtgaAGAAGAAAAGGACTTTCTCtCAGGCAGAGGGAaGGACAAGTGATaGAGGAAaGGATGGATtCGGTTACCAGATGGGggAGAGTAGCTGTGCCACAGCTGCTAGGAGCTGCAGTTGTACTGGCTGTGCATGAAACCACCCATCTAGGTCAGGAGTCACTTcGAAAAGTTGTTAGGCTGGTATTTCTACATCTCACATTTtGTCAGCCCTTGCCAgAAACaGTGACGCAGCGGTGTGTTACCTGCCGACAGCAtAATGCGAGGCAAGGTCCAGCcGTTCCGCCCcGGCATACAAGCTTATGGAGCAGCCCCCTTTGAAGATCTggatCCAGGTaGACTTCACAGAGATGCCAAAGTGTGGAGGTAACAAGTATTTACTAGTTCTTGTGTGTACCTACTCTGGGTGGGgtggtggtggtggtgTGGAGGCTTATCCAACACGAACTGAGAAAGCTCGTGAAGTAACCCGTGTGCTTCTTCGAGATCTtATTCCTAGATTTGGACTGCCCTTACGGATCGGggCTCAGATAACGGGCCgGCGTTTGTGGCTGACTTGGTACAGAAGACAGCAAAGGTATTGGGGATCACATGGAAACTtggaGCATGgCCGCCTACCGGCCTCAGtggaaaAGTTCCGGAAAGGgTGGAGaCGGATGAATCGGACTATCAAAAATAGTTTAGGGAAAgTaTGTCAgGAtcAACAGGATTAggcttagggaaagtgtgtcaagAAaTGGaTACAgGCtcTcgCCTATgGTATTgTTTAAaATtAGaTGgtACccCTTCTAAaAgAACAGGATAtTCCCCTTATGAAATAtTaTATCATAGgCataatatacgcCcCCTCCcATAcTgcggGGACTtCCAGttaaaaGCACTCCccgAGAGtTAGGTGAAATTGAgTTACAGcgAcAGCagTACAGGCtTTAGGaAAAATTACAccAAacAATcTCAgCcTGGGTAAATGAGAGgtgcCCtgTtAGCTTATTCTCCCCAGTTCACCctTTcTCcCCcAGGTGATCgaGTGTGGATCAAGgAcTGGAAcGTAGCccCtTTGcGgCCAcGGTGGAAAGGACCcCAGAccgTCaTCcTGAcCACtCCCACcGCtgTgAAGGTAGAaGGAATCCCagCCTGGATcCAcCACAGCCatgTaAAaCCTgcCtctcAGcacCTGAaACCTGGGAGGcaAgACcaAGcccaGACAAcCCagctacTGCAaAaatGTGACtcTGctgcAagAaGAcgACAAGCCCTGCTCCAGTCACACCcgGAAGCTGACTgGTCcAcgCAcgGCcgAAGCATGAGGAaacTCATcgTGggAcTcATTTTcCTtAaAaTtTGGACTTgTatacAGTAAggaCTTCaACTGatcTTcCttccaCAcActGaGGaCTgttccCAgaagaTGtataCatCAggttActgAggTAGGgCAacAagTTAAAACAaTCTTTcTtGTTctAtAgttAtTAtgaaTgtataggaActttaAAAGgaACttGttTgTATAATgcCACtCAGTAcAaatctttAgGTATGtAgctattttgaatgcCcaggAAatGAccaaCCtgaTgtgtgttataacCcatctgAgCCccctatgaccacagttTTTgAaataAgatTaAgaActGgccactggtgaggactcataggtGatAcaaGtaaagTAaTAactAgAACAgAAGAAAaaaggagTcCCcAaacaaataAtcTTaAaatttGAtgCtTGTgcagccATtAAtAgtaacaaGttaggAAatagaataggAtgtggcTcTcTaaatTggGaaaaAAaggagccatatatatagtagAaaataagtatatttgtcatgaattaggagtgtgtggaaataatgaatgtagttactggtCttgtgTcAtTtaggctaCtTggaataaAAAgaatgAAaaggAccctgtttaccTtcagaaagGaaaagctcaaccccTcctgcactagtggtcactGtAacccattAgaactAataattaccAatCccctagAtccccattgGaaaAaaggaGAacatgtaaccctagGgatcgatgggacaggactagaattcctcaagtaaatattttaaTtaaaggagaggtccacaagcgctctcccaaaccagtgtTtcaaAcctttTatgAtgaactaaAatcTgccagtaccagaacttCcaaaaagaagAcaaaaaatttgtttctccaattagcagaaaatgtagctcattcccTcaatgttacttctTgttatgtatgtggaggaaccactatgggagaccaatggccttgggaagcccgagaattggtgcctactgatccagttcctgatataattccagttcaaaagacccaacctagcaacttctgggtcctaaaaacctcaattattggacaatactgcatagctagagaaggaaaagacttcatcatccctgtaggaaggcttagttgtctaggacaaaagctgtataacagcacaacaaagacagtcacttggtggggtctaaaccacactgaaaagaatccatttagtaaatttccaaagttgcaaactgtttgggcccatgcaccagaatctcaccaggactggacagctcccactggactatactggatatgtgggcatagagcctacactaagttacctgacacaatggggcaggtagttgtgttattggcactattaagccatcttttttcttactgcccataaaaacaggtgatgagctcctaggcttccctgtctatgcttcccgagaaaagagaagcatagctataggaaattggaaagatgatgagtggccccctgaaagaatcatacagtattatgggcctgccacatgggcacaagacggctcatggggataccgaacccccatctacatgctcaaagccggatcatacggttacaagctgtcttagaaataattactaatgaaactggcagagctttgactgttttttagcccggcaagaaacccaaatgagaaatgctatctatcaaaatagattggccttagactacttgctagcagctgaaggaggagtctgtggaaaatttaacttaaccaattgctgtctacaaatagatgatcaaggacaagtagttgaaaacatagttagagacatgacaaagctggcacatgtgcctgtgcaggtttggcatggatttgatcctggagccatgtttggaaaatggtttcgtttccagctataggaggatttaaaaccctcattgtaggtgtattgctagtgacaggaataggaggatttaaaactcttataataggagttataatagtaataggaacctgcttactgctcccttgtttgctacctgtacttcttcaaataataaaaatgcttcattactaccttatttcaccaaaaaacttcagcacaagtgtattatatgaatcactatcgatctgtctaacaggaagacctaaatagtgagaatgaaagtgagaactcccactatttcctggagtgagattctcaaagtggggaaactacagtagtccctaggagatcaccccgcaaaaaatgaaattgtcttatactttgtatcttcctcaaaaagaaagaagaagtaaaaactaaaaggcagaaaacctgaaatcgataggcagacaacccagaaccacaccctggcctgaccaaaattagcctggtagttaaagattaacccctgacgctaatcacttatgttatctatagattacagacattgtatagaagagctatctgaaactcttattcctgttccttacttatcttattacagttgcatgcagtacacaccttagaattcccagcttttacaatggatcacaaccttttcatggggaaacccttatagtaataaaccattaaaaggaacatcaagttcgcattaaaaaagctctgtctttggaaacgtgagttctgatgagtctattctgcaaatcaagctgctacttcattaattcattgtctaaggggttttttctgcaaattgtcctgctaaaggttcttcagaccccctcccttccctacacatcaagctcagggatttgcctccacccaggactggcaaattagctttactcaacgtgccccaagtcaggaaactaaaataactcttggtctacgtagacactttcactggataggtagaggcctttcccacagtgtctaagaaggccaccacggccatttcttcccttctgtcacacataattcctcagtttggccttcccacctctatacagtctgatagcagaccggcctttattaatcaagtcagccaagcattttttcaggctcttagtattcagtgaaagctttatatcccttacagtcctcaatcttcaggaaaggtagaacagactaatggtcttttaaaaacacaccttaccaagctcagccaccaacttaaaaaggactggacaatacttttaccactttcccttctcagaattcaggcctgtcctcagaatgctacaaggtacagcccatttgttgcgggaagtcagggacccca

>herve_10210_100008_con_gagputein

fcdiesniilfplgsweqytwwcplsaiyvvlslpppwnvikdhltrgctlpamlgvialsssvnirskimgqtqskptplgtmLknfkkgfnGdyGvtmtpGKLRTLCEIDWPaLEVGWPSEGSLDRSLVSKVwHkVTgKsghpDQFPYIDtWLQLvLdPPqWLrGqQaAaVLvAKgQiaKEgsrSTrrgKsaPkVLsdPtsEdswQEmapslvipvvpspyqggrlPtpeptvlappqdkhiPRPpRVdKrGgEasGETPPLAArLRPKTGIQMPLREQrYTGIDEDGHmVERscrvFvYQPFTSADLLNWKNNTPSYTEKPQALIDLLQTIIQTHNPTWADCHQLLMFLFNTDEggRRRVLQAATKWLEEHaPADYQNPQEYvRTQLPGTDPQWDPNEREDMQRqgLNRYREALLEGLKRGAQKATNVNKVSEVIQGKEESPAQFYERLCEAYrMYTPFDPDSPEsNQrMINMALVSQSAEDIRRKLQKQAGFAGMNTSQLLEIANQVFVNemRDAVSrkEnrKEneerQARrNaDLLAaAIRrGvPPKrQGKGGPGKeTQsGcqsLQrNQCAYCKEIGHWKNKCPQLKrKQGDSEQEAPDKdEGALlNLAEGLLD

>herve_10210_100008_con_proputein

GGPGSsaPKEPmVRMTVGGKDIDFLVDTGAEHSVVTtPVaPLSKKTIDIIGATGVSAKQAFCLPRTCTVGgGHKVIHQFLYMPDCPLPLLGRDLLSKLRATISFTeHGSLlLKLPgTGVIMtLtvpreeewrlfltepgqeirpalakqwprvwaednlpgwastkpayl

>herve_10210_100008_con_polputein

aavlgvwgypvlwdgialvsltkasllqlklpgtgvimaltvpreeewrlfltepGqeirpalAKrwPrVwaEDNPpglavnQAPvLIEVKPGAQPVRQgKQyPVPREALEGgvfsIQVHLKHLRtFGIIVPCQSPWNTPLLPVPKPGTKDYRpVQDLRLVNQATzVTLHPTVPNPYTLLGLLPaAEDSWFTCLDLKDAFFSIRLAPESQKLFAFQffqWEDPESGVTTQYTWTgLPQGFKNSPTIFGEALARDLQKFPtRDLGCVLLQYVDDLLLGHPTAVGCAKGTDALLrHLEDCGYKVSKKKAQICRQQVrYLGFTIrqqGErSLGSERKQVICNLPEPKTRRQVREFLGAVGFCRLWIPNFAVLAKPLYeVTKggggadtEpFEWGSQQQQAFHELKEKLMSAPALGLPDLTKPFTLYVSEREKMAVGVLTQTVGPWPRPVAYLSKQLDGVSKGWPPCLRALAATALLvQEADKLTLGQNLNIKAPHAVVTLMNTKGHHWLtNARLTKYQSLLCENPrITIEVCNTLNPATLLPVSESPVEHDCVEVLDSVYSSRPDLrDQPWASVDWELYVDGSSFiNPQGERCAGYAVVTLDTVvEARSLPQGTSAQKAELIALIrALELSEGKTVNIYTDSrYAFLTLQVHGALYKEKGLLNSGGKDIKYQQEILQLLEAVWKPHKVAVMHCRGHQRASTLVgLGNSrADSEArKAAStPfrASVTAPLLPQAPDLVPTYSKEEKDFLQaEGGQvieEGWIrpLPDGRVAVPQLLGAAVVLAVHETTHLGQESLEKLLGwYFYISHLSALAKTVaQqCVTCRQHNARQGPAVPPGIQAYGaAAPFEDLQVDFTEMPKCGGNKYLLVLVCTYSGWVEAYPTrTEKArEVTrVLLRDLIPRFGLPLRIGSDNGPAFVADLVQKTAKVLGITWKLHAAYrPQSSGKVErMNRTIKNSLgKvcQETGLlkqhhkgKwiQalpmVLFkIRCTpsKrtGYspyeilyhrppPilrGlpGTpaeLGEIeLQrqLQALgKITQtISAwvnErcpvsLfSPVhPFSpgDrVWIKDWnvApsrLsPlWKGPQTviLtTPTAvKVEGIPAWIHHShiKPaapETwEarpspDNpckvtlkkttSplfvtgvsygvggiflfttpailevizvsppldirnnitggceqplrylezyhrlpsrilrtis

>herve_10210_100008_con_envputein

ALLQSHPEADWSTHgrSMRKlIvGLIFLKfWTCtVraSTDlpqTeDCSQCIHQVTEVGQQVKTiFLFYSYYEClGTLKGTCLYNaTQYKVCSPgNDqPDVCYnPSEPPmtTVFEIRLRTgdwwgflgDTSKiItRTEEKgvPKqItLkFDACAaINSnlyKLgiGCGSLnkwErsYrvENKYvCHElGlCgkeCgYWSCVIzATWKKNEKDPVyLQKGKanPSCtsGhCNPLELiITNPLDPhWKKGErVTLGIDGtGLDPqVNILirGEVhKcSPKPVFQTFYDELNlPvPElPkKTKNLFLQLAENVAhSLNVTSCYVCGGTTmGDqWPWEArELVPTDPvPDiIPvQKahpsNFWVLKTSIIGQYCIAREGKdFTiPVGkLnCLGQKLYNSTTKTVTWWGlNHTEKNPFSKFpKLQTvWAHPESHQDWTAPaGLYWICGHRAYtKLPDQWAGSCVIGTIKPSFFLLPIKTGELLGFPVYASrEKRSIAIGNWKDDEWPPERIIQYYGPATWAQDGSWGYrTPIYMLNwIIRLQAVLEIITNETGRALTvLAwQETQMRNAIYQNRLALDYLLAAEGGVCGKFNLTNCCLqIDDQGQVVENIVRDMTKLAHVPvQVWHgFDPGSlFGKWFPAiGGFKTLIiGViiVIGTCLLLPClLPlLlQMIKsFvaTLVHQKtSAQVYYmNHYRSvsQeD

**HARLEQUIN**

>harlequin_10210_877_bre_chaindnarm

tgcagcccccagtcacatactccctgcttgctcaatcaatcccgaccctctcacatggacacccttagagttgtaagcccttaaaagggacagtaattgctcactcggggagctcggtttttggagatgtgagtctgcagatgctcccagttgaataaagctctttccttctacaattcggtgtctgagcggttcttgtctgcagctcatcctgctacatttcgtggtttcctgaccaggaagcaggtgattaaggacggacagttgagtcagccccttagtcagcttaggcctgccctgtggagcatccctgcgggggactccggccagcttgagcgatgcagatcctgagagcactctcgggtaggcatttgccctggtggaacgcctcgtcagagcggtggacagcaggcccttgtggatgatcagtgcagtggctgaacaccgggaaggaactggcacttggagtccggacatctgaaacttggtaagactggtctttggaacttgcccactccatttgagtggaagcatggcctgatcacccacgacgtgcctgtactggcactttggtttttgtttttggcttgacttggattgcttgatactttggttttggttttgacctggcttggatttgatactctgattttggttgtttctggtttggtgtaaactgtaaaagtgtgtgtgtgccctctttacctgttctttgttttgtggtgtgagtgtggtgtgagtgtggtgttttgtctggaggaaacatgggtgaggcacaaagtgagcccatcccactaggaactatgttgaaaaatttcaaaacaggatttaagggagactatggagttatgacaccaagaaaacttagaactttgtgtgagatagactggccagcattacaggtacgttggccatcagaaggaagcctggacaggtcccttctctcaaaagtgtggcacaaggtaacttgtaagccagggcacccagatcaattcccgtatcaattcccgtatatacatatagattcttggttacagttagttttagacccctcacagtggttaagaggacaggcagcagcagtactagtagcaaagggacagttagttaaggaaggttctcgctccacccaccgagggaattcagcaccaaaagtcctgtctgtcccaacaccagaagaatcatggcaggaattggtaccaggaataccccctccttattgagaggaagggctccccactccagaacccacagcacctccacctccaccagatagccacactcctagaccacccagcatagacaaaagaggaagtaaagctgaaggagaaactcctcccttggcagcttgcttacggcctaagactggaatccaaatgcccctgagagaacagcaatattactggggtaaatgaggatggacacatggtggaaaggcgtgcctttgtatcaacctttcacctctgctgacctcctcaattggaaaaataatactccaccttataccaaaaagcctcaagctttaattgacttgctccaaaccgttatacagactcataatcctacttaggctgattgccaccagctgctcatgtacctctttaatacaaataaaaggcaaagggtgctccaggcggcaactaagtagctagagtagcatgtcccagccgattaccaaaacccccaagaatatataaggattcagctgcaaggaacagacccccaatgggacctgaacgagggaccagacacagggaggctaagatggtatcctaaggcattgataaaaggtctaaagaaaggggctcaaaaggttacaaatttaaataaggtctctgaggtcatccaagaaaaaggggagagtccagcacaattctatgaaagactgtgtgaggcttaccgtatgtacactccttttgatccaaatagccctgaaaatcagcacatgattaacatggccttagttagtcaaagcacagaagataacaggaaaaaattgcaaaaacaggcagggtttgtgggtatgaatacctcgccattactggaaatagccaatcaagtgtttgtttgtgaacaaagatgcaacaagccgctgagaaagccgaacaccaggccaggcggaaacgccaacttactggccgcggccattaagggaattcccccaaaagggaaagaaaaggagggttctgggaagaatacccagtctaatcgcccacactttcaatgtaaccaatgctcctattgtaaggaaataggacattagaaaaataagtgtccccaactgaaggaaaaacaaggtgattcagaacaaaagaccttagataaagatgagagagctttgttcaatctggctgaagggctattagactgaaggggactgggctgaagcgcccccaaggagcctgccgtcaggattacaattggaggcaaggacattaagtttttggtcaatactggtgctgaacattcagtagtgaccaccccggtcacccccttacccgagaaaaccattgatataatcggagcaacagaagtttccactaagcaggctttctgtctaccacggacctgctgggtggtgggacataaaatagttcaccagttcttgtacatgcctgactgtcccttgcctttgctgggaagagacttgcttagcaagctgagagccaccatctcctttacaagacagggctctttacagctaaagttactgggaacaggagttatcatggcccttacggtcccccaggaaaaagaatagagactttttctaaccgagccaggccaagagataaaaccagtctagctaagcgatggccccaaatatgggcagaggataatcctctgggactggcagtcaatcaagcacccgtactcacagaagttaagcctggggcccagccaattagacaaaagcagtatccggttcccagagaagctcttgagggaatccaggttcatctcaggcgcttaaaagcacctataatttagaactatagttccttgccagtctccatagaacacctacctcctgcctgtccctaagccagggacaaaggactaccggccagtgcaggacttgcgcttggtcagccaagctacagtgactctgcacccaacagttcctaaccgttacacattgttagggctactgcctgctaaggacagttggtttacctgtctggacttaaaagatgccttctttagcatcagactagctcctgagagccagaagctgtttgcctttcagtaggaagatccggagtcaggtgtgactactcagtacacttggacccggcttccccaagggttcaagaactcccctactatcttcagggaggccctggctcaagacctgcaaaagtttcctgctaaagacctaggctgcatcttgcttttgtacatgaacgaccttctgctgggacactccttggcagtcgggtgcgcaaaaagtacggatgtcctacttcagcacctggaggactgtaggtagaaagtgtccaagaagaaagctcagatctgcagacagcaggtatgctacctgggattcactattcggaaaggggagcgcagcctggagtcaggaagaaagcaggtcatctgcagcctactggaacctaaaaccagaaggcaagtaagggaattcctacgagctgtaaggttttacagattatggattccaaactttgcagtactagcccaacctttgtacggggctacaaaggggggcgaccggtaacttttaaatgggagcctctacaagagctagtcttttgtaagttaaaggaaaaacttacgttggcctgtgccctaggactaccagatttgacaaagccctttacactctatgtgtcaaaaagaaaaaaaatggcagttacagttttaaccgactgtggggccctggccaagaccagtggcctatctctcaaaacaactagatggggtttccaaaggccggccaccatgtctaagggccctggcagcaacggccctgttagcacaagaagcagataaactaacccttgggcaaaagctgaatataaaagccccccatgctgtggtaaccttgatgaataccaaaggacatcattggctaacaaatgctaaattaaccaagtaccaaagcttgctttgtgaaaatccccagataacaattgaagtctgtaacaacctaaatcccaccaccctgttcccattatcagaaaggctggttgagcataactgtgtagaggtgttgggctcagtctattctagcagacctgaccttcaggaccagccatgggcatctcagtagactgggagttatatgtagatgggagcagcttcatcaacccacaagatgaaagatgtgcaggatatgtggtggtaactttggatgctgtcattgaagccaaaccattgccacagggcacttcagcccagaaggctgagctcattgctttaactcagattctagaactcagtaaaagtaagactgtaaacatctacactgactctcgatatgcctttctaattctcaagtgcatggagcattatacaagaaaaagggcctgttaaaccctgggggaaaggacataaaatatcaacaagaaattctacaattattagaggcagtgtaaaaacctcaaaaggtggcagtcatgcactacaggggacaccagcgagcctccacctcagtggccctaggaaactctcgagctaattcagaagctcgaaaagcagcatctaccccttactgggcatcggtagcagccccattactccctcaaacacctgacctggtacctacctattctaaggaagaaaaagactttttccatgcagaaagggggcaagtaataaaaggaggatagatcagactgccagataggaaggtagctgtgccacagtttctgggagccacaatcgtattggccgtgcacaaaactactcatctaaggtcaagagtcacttaaaaagttgttaggccagtacttctacatctcacatttgccagcccttgccaaagcagtagcacaacagtgcgttacttgccaacagcacaatgtgaggcaaagccccactgttccacctggcacacaagcttatggagcagctccttttgaggatcttcaggtaaatttcacagaaatgccaaaatgtggaggtaacaagtatttgctggttcttgtgtgtacttactctaggtaggtggaggctgatccaacacaaactaaaaaggcctatgaagtaacccgtgtgcttctctgagatcttattcctaggtttagactgcccttacgaatcggctcaggtaacaggtttgtgagcgacttggtacagaaaacagcaaaggcattaggaatcacttggaagctacatgccgcctaccgacctcagagttccggaaaggtggagtgaatgaatcggactatcaaaaatagttttaaaaaagtatgtcagaaaacaggattaaaatagatacaggcccttcctatggtattgtttaaaattaaatgcactccttctaagaaaacagaatactccccttatgaaatactgtatcataggcctcctcctatactacagagacttccaggcactccccgagagttaggtgaaattgaattacagtgacagctacaggctttaagaaaaattacacaaacaatctcaacttaggtaaataagaggtgtcccatcagcttattctccccagttcatcctttctttccaggtgatcgcgtgtggatcaaggactggaacgtagcccctttacagccaccgtggaaaggacctcaaaccatcatcctgaccacccccacggctgtaaaggtagaaggaatcccagcctggatccacaacagccatgtgaaacctacagccactgaaaactaggagacaaaaccgagcctggacaagccctgcaaagtgactctgaggaggacgacaagccctgctccagtcacatccagaagctgactggtctacgcacggctgaagcatgaggagaatcatcgtaaaactcattttccttataatttggacttgtatagtaaaaacttccactgattttccccgcatgaaagactactcttagtgtatacatcaggttactgaggtaagacaagaaagtaaaacaatctttctgttctatagttactatgaatgcctagaaactttaaaaggagcatgtttatataatgacactcagtacaaggtatgtagcccaggaaacgaccagccagatgtgtgttatgacccctctgggcctcccatgtccacagtttttaaaataagattaagaactaaagactggtgaggactcataaataatacaagtaaagtattagccagaacaaaagaaagggggtgcccaaatgcataatcttgaaatttgatgcctgtgctgtcattaatagcaataagttagaaaggaaatgtgactctcttaattaaaaaaaagactatatgaccaaaagtaagtaaatttgtcataaattaggactatataaatataaatgtaaatactggtcttgtgtcatttaggccatttgaataaaaaataaaaatgatccagtccaccttcaaaaaagaaaaaaatggcccttcctgtactaagggacaatgtaactccttagagctagtaataaccaatccccttgatcctcagtgaaaaaaaggggaacgtgtgaccttaggaatcgatggggccagactggatccttgagtaaatatcttagtttcaggaaaagtttacaaacgctctcctgagccagtgttttaaactttctatgatgaactaaatgtgccagtaccagaaattccaaaaaaaacccaagaaatttgtttttgcaattagccgagcatgtagcccagtttctcaatgtcacttcatgttatgtatgtggagaaactgtaacaggagatcaacagccataggaagcatgaaaattagtacctacagacccagttcctgatgaattcccggctcagaaaaatcaccctgacaatttctagtcctaaaagcctcaattattggacagtattgcatagctagaaaaaaaaaatccactcaccctgtaggatgacttagttgtctaggacagaaactgtatagtggtaacacgaaaacagtcactgggtggagttcaaatcacacaaaaaggaatccatttagtaaattcccaaagttgcaaaccgtgtggacctacctggagtcccaccaggactggacagcccccactggattatactggatatgtgggcatagagcttatgcgaaattacctgaccagtgggcaggtagttgtgttattggcactcttaaaccatctttcttcctactgcccataaaaacaggcaaactcctagcttccctgtctatgcttcccacaaaaagagaagcatagttataaaaaattaaaaaaaaaatagccccctgaaaaaaaatcatacaatatcataggcctgctacttagacacaagacagctcgtggggataccagactcccatttacaggatcaatcaaatcatacggttacaagctgtcttagaaataatcactaataaaaccggcagagccttgactattctgctctggcaagaaactcagatgagaaatactatctatcaaaatagattagctctctactacttgctagcagctgaaggaggggtctgtaggaaatttaaccttactaattgctgtctacacatagatgatcaaggacaagtagttgaagacatagttaaaaatatgacaaaactgacacatgtgcctgtgcaagtgtggcatggatttaatcctaaggccatgtttaaaaaatggctcccagcgctaggagaatttaaaacttttataataagagttataatagtaataagaacctgcttattgcttccttgtttactacctgtacttcttcaaatgataaaaagcttcactgctaccttagttcacaaaaatgctttggcacaagtgtactgtatgaattactatcgatctgtcttgcaaaaagacataggtagtaaaaataaaagtgaaaactcccactaatgagtgaggttctcaaagggaggggaataaagagggagaccacccctcatattgtcttatgcccaatttctgcctccaaagaaagaataagtaaaaactaaaaggcagaaatgaaatccacaggcagacagcccggcgccacaccctggggcctggtagttaaagactgacccctgacctaatgggttacgttatctatagattatagacattgtatggaaaagcactgtggaaatccctgtcctgttctcttccattctaattaccggtgcaggtgcatgcagcccccagtcacgtactgcctgcttgcttgctcaatcgatcacgaccctctcacatggacacccttagagttgtaagcctttaaaagggacaagaattgctcactcggggagctcggtttttggagacgtgagtctgccgatgctccca

>harlequin_10220_877_bre_gagputein

MGeAQSePIPLGTMLKNFKtGFKGDYGVMTPRKLRTLCEIDWPaLqVRWPSEGSLDRSLLSKVWHKVTcKPGHPDQFPYqfpyIhIdSWLQLVLDPSQWLRGQAAAVLVAKGQLVKEGSrSTHRgNSAPKVLSVPTPEESWQELVPGiPPPYzeeGlPTPEptApPPPPdSHTpRPpSiDkrGSkAEgeTPPLAAclRPktGiQmPlrEqQyywgmrmDTwwkGVPlYqPFTSADLLNWKNNTPPYTKKPQALIDLLQTVIQTHNPTzADCHQLLMYLFNTNKRQRVLQAATKzLEzHVPADYQNPQEyIRIQLqGTDPQWDLNEgPdTGRLRwYpkALIkGLKKGAQKVtNLNKVSEVIQEKgESPAQFYERLCEAYRMYTPFDPnSPENQHMINMALVSqSteDnRKKLQKQAGFVgmNTSPLlEiAnQvfVCEQRcNKpLRKPNTRPggNAnllAAAIKGiPPkGKekeGSgKNTQsNRPhfQcNQCsYCKEIGHZKnkCPqlKeKQGDseQkTLDKDeraLfnLaeGlld

>harlequin_10220_877_bre_proputein

rGLGzSaPKEPaVRiTIGGKDIkFLVnTGAEHSVVTtPVtPLpeKTIDIIGATeVStKQAFCLPRTCwvvGHKIvHQFLYMPDCPLPLLGRDLLSKLRATISFTrQGSLqLKLlGTGVIMaLt

>harlequin_10220_877_bre_polputein

PpGkRieTfsNraRPrdKTSLaKrwPQiWAEdnplGLAVnQAPVLtEvKpGAqPIrqKQYPvPrEAlEGIqvHlRRLkapiIzniVPCQSPzNTyLLPVpKPGTKDYRPVQDLRlVsqatVtlHPTVPNrYtLLGLlPakdsWfTcLDLKDAFFsiRLaPESQkLFAFqzeDPEsGvTtQyTWTRLPQGFKNSPTiFrEALaqDLqkFpAkDLgcILLlYmnDLLLghSLavgCaKsTdvLLQhLeDcRzkVSkKKAQICrQQVcYLGftirKGeRsLEsgRKqVicSllePKTrRQVREFLrAvrFyRLWIPnFAvLAqPLYgaTKGrPvtFkWEplQeLvFcklKeKLtLAcALGLPDLTKPFTLYVskrkKmAVtVLTTvGPWpRPVAYLSKqLDgVSkGrPPCLRAlAAtALLaqeADKLTLGQkLnIkAPHAvVtLmNtKghhWLTNAklTkYQSLLcEnpQitIeVcnnLNPtTLfPLSErLVeHnCvEvLgsvyssRPDLqDQPwasVDwelYvDGSSFINpQdeRcAGyVVvTldaVIeAKpLPqGTSAQKAELIALTQiLeLsKsKtvNIYTDSRYAFnsqVHGAlYKkkGLLnpgGKDIKyqqEILqLLEAvzkPqkVAvmHyrGHQRaStSVAlGNsRAnsEARKAASTPywaSvaapLLPqTpdLvpTYsKeeKdfFHaergqViKGgzirlPdRKVavPqflgaTivLavHKtTHLGqEsLKkLLgqYFYIshLpALaKaVaqQCvtCQqhNVrqSptVpPGTqAyGaaPfEdlqVnFTEmpKcGGNKYLLVlVcTySrzVEAdPTqTKkAyeVTrvLLzdliPRFrlPlriGSgNrFVSdlVQkTAkaLGItWKLHaAYRPQSSGkVEzMNRTIKnsfkKvcqkTGlKziqaLPmVLfkikcTPskkTeysPYEILYhrPPPILqrlPGTpReLgeieLQzqLQALrkItQTIstzvnKrcpisLfsPVHPFfPGDrVWikdwnvaPLqPpWKGPqTiiLTTPTAVKVeGIpAWIHnSHVKPTaTETRRQNRawTSpak

>harlequin_10220_877_bre_envputein

ALLQSHPEADWSTHGzSMrriIvklIFLIiWTCiVktSTDFPrmKDySzCIHQVTEVrQeSKTiFLFYSYYecletlKgaCLynDTqykvCSpGnDqPDVCYDPSGPPmSTVFKiRlRTkDwzGLinnTSKISQnKRKgvPKcIiLkFDACAvINSnKLerkCDSLNzkkdYmTKvsKFViNzdyinINvnTGLVSfrpFezKIKmiQsTfkKeKnGPSCtkGqCNSLELvITNPLDPqzKKGERVTLGIDGarLdPzVnILvsGkVyKrSPePVFzTFYdELNvPVPEiPKKPrNLFLQLAEhVAqfLNVTSCYVCGeTvtGDQqPzEAzKLVPTDPvPDefPAQKnhPQFLVLKASIIGQYCIARkKkSTHPVGzLsCLGQKLYsgnTKTVTGWSSNHTKrNPFSKFPKLQTvwTYLESHQDWtAPTGLYWICGHRAYakLPdqWAGSCVIGTLKPSFFLLPIKTGkLLFPVYASHKKRsIVIkNzKkNsPlkkIIQYhrPATztQDsSWGYqTPIYriNqIIRLQAVLEIITNkTGRALTiLLwQETQMRNtIYQNRLALyYLLAAEGGVCrKFNLTNCCLhIDDQGQVVeDIVKnMTKLtHVPVQVWHGFNPkamFkKwLPALGeFKTfIIrVIIvIrTCLLLPCLLPvLLQMIKSFtAtLVHKnaLAQVYcMnYYRSVLQkD

>harlequin_10220_2630_bre_chaindnarm

tgtctcctgtctccaaagaaaaggaggaaactgaaaaataacagactgattgggcgccactggccaggcctgtaagttaaagattaacccccatcctaatcgcttgtgctatctgtagaccacagacaatgagaccacagacaatggtacagagaaatgcttgccttgcttacccccacctagtcaggtatcccacgcttgctcaatctatcacaaccctgtcacatggaccccttagagttgtaagcccttaaaagggccaggaactcttcttcagggagtttggttcttgagacgtaagtctgccgatgctcccggccaaataaagccacttcccacttccttctttaacccagtgtctgaggggttttgtccacggcttgtcctgctacatttcttggttccctgacagggaagcgaggtggttgaaggttggccgaggcagcccctaggcggcttaggcctgccctgttggagcatccctgttgggggaatccagccagcttgagcgaagcagatcctgagagcgctcctgggtagacacttgccccagtggaacgcctagtcagagcggtgcctggcaggcccccgtggaggatcaatgcagtggctgaacaccaggaaggaattggcacttggagtctggacatctggaatacggtaggaccggtcttgggaacttgcccactgcatttcagtggaagcgtggcctgcctttattggcactttggttttgcttttgattttgacttggtttgattgcttgatgaatgagcatgcctttatcggcactttggttttggtattgattctgatttggtgtgaattgcttgacgagtgagttaccttttactctttgccctttttctttcccttcttgtggcaagagtgttgttttgtctcggagaggaaaatgggtaaaacacagagtaagcccaccccgttagggactatgttaaaaaaaattttccaaaaaggttttaatggagatgatggggttactgtgaccccaggaaaacttaggaccttgtgtgagatagattggccagcattagaagtgggttggccatcagaaggaagcctagataggtcccttgtttcaaaggtatggcacaaggtagctggtaagccaggatacccagaccagtttccgtacatagacctttggttacagctggttttaaaccccccacagtggttaagaggacaggcggcagttgtactagtggcaaagagagggagaatttatgtaaaaagaatgttatatggtaaattcttgtcctgaaataaattaacttcttgtttaaagaaagggatgtttgcaataagtcagaaagttgaggcatgtcgaagaattgtctgtgaaagtcgtgaaagaaaaaaaatgtgctataaaaagcaacttatgcaagaaatgctgtataatttcaaagtaattaggcctcctgaatgtaaaactattgaagaaacagtttatatgcaaggtgtataaggaaagtaaaatatacttttaataaaaggattataaggaggcataagaatgtggatttttacctacattaaaaggttaaaaaaattttgttttgaaggtttaagcaagttttgaaacgttaattgtaaaggaaattctgtgtgtaaacatattggctgaagttaaaggggtatcatccagtttttctgtgactggacattaaaataaaaacacaatgggtttttcttgaagcattaacctgctctttaacaaaaattataaaaggttaaaaagagtctataaaaatcttaccttatggtcagacattaaaaattgaataaatatatctacaaagttttattgaaactaagtttaacattaataacacactaatataaaggtgaaatttagcttatctggtataaaaatcatacaggaagcattgtcaaataaaaaatggtgtttgtctttctttggtctaaaaactaataaaaataggtgttaaaggaaatttctcagtaagaaggcaccaaggactataaagtccactgctgatgtccccacatttacaacaaaaggtcaatttcttcgaaattatatacttggtttatcttccactttactttccttcaaaactaaaagtctttcagcacacgtaccacccctagaatttccagtaaaccagcaccagcctgaagatcacgttctcatcaaagggtggaaagaaggaaaactcgagccagcctgggaaggaccctaccttgtgctgctaaccactgagattgctgttcgtacagccaaaaatggatagactcatcacaaccaagtcaagaaagtaccaccccctccagagttgtgggccatagtcccaagggaaaatcctaccaaactgaagctaagagaaatttaactctttcatctattctattacttctgtccttgctctgttgctgaccatctagttattaacaaaactaaatcaatttcacctcaaactattacatttaatgcttgccttgttataccctgtggggacttgccaaatcaaagacagctctctacttcagaaaagtaactctgcccctcctgactctcctcagactgggcattagtaaattgggaccatttaatctggggagattttgataaagaccccagagtcaaccaggagtcttgtcccctgatgtagagtttttatgccatagttggtccaacattctgtgggccactaaagagcaaggatggactgccccaaccggtttttgtaatttcctaaaaccatacattcattttactagaggatcatagaagttaaagacaaaacaaactttagcaattaagacaggataacaagatgcaaatgcttggttggaatagatcaaatattccatctgcatgttaaacaaaagcaattgttatgcttgtgcacatggcagaccagagacccagattgtcccctttccactaaagtggtcctccagtcgaccaggcgtggactgcatggtatctcttttccaggattctacagcctggagtaataagtcgtgccaagctctctctctgctatgtcctgaagttcagcaccctgtgggtcagcccctgagggccacccagcttctgtctcccaacactaagttcacttcatgtctctcatgacagggaggaaacttagcattccttggagacctgaagggatgcagtgagcttaagaattttcaaaagcttatcagtcagtcagcccttgttcgtccccaagcggatatgcggtggtatcgtagtggacctttattggacactctgccgagtaactggagtggcacttgtgctttagtccaattggctatccctttcaccctggcatttcatcaaacagagggaggaaaaataagacatcgtagagcgagagaagccccttataggtcttttgactctcacatctatttagatgcaattggagtcccatggggaatatcagataagtttaaagcctgaaatcaaatagttgcaggatttgagtcaatattttggtgggtgacagttaataaaaatgtagattggataaactacatctattacaaccaacagcaacgaatttttcatgagtttaaaaaaaaaaaaaaaaaaactcatgtcggctccagccctggggctacctgacctgacaaaaccttttacactatatgtgtcagagagagaaaaaatagcggttggagttttgacccagactgtggggccctggctgaggccagtggcctacctctctaaacaactagatgggatttctaagggttggcccccatgtttgagggccttagcagaaacagccctgctagcacaagaagcagataagctaactcttgggcaaaacctaaacataaaggccccccatgctgtggtaccactaatgaacgtctaaggacatcattggctaacaaatgctagactaactaggtaccaaagcttgctctgtgagaatctccacataaccattgaagtttgcaacaccccaaaccccaccactttgctcctggtatcagagagcccagttgaacataactgtgtagaggtgttggactcagtttattctagcaggcccgacctccgagaccatctttggacatcagtagactgggagctgtacgtggacgggagcagcttcatcaacccacaaggagagaggtgtgcgggatatgcggtggtaatcctggatgctgtcattgaagccaaatcattgccccagggcacttcagcccagaaggccgaactcattgctttaatttgggccttagagctgagtgaaggtaagactgtaaacatttacactgactctcggtatgccttcttaaccctctaagtacatggtgcattattcaaggaaaaaggcctgttgaactctgggggaaaagacataaagtatcaacaagagatcctgcaattattagaggcactgtggaagccccaaaaggtggcagtcatgcactgcagaggacaccagcgagcttccacctcgattgccttagggaactacagaggagacttagaagctcgaaaagcaatatccaccccctgccgggcatcagtcacagcccccctgctccctcaagcacctgaccttgtacctacttctgtacctacttattctaaagaagagaaggactttctccagacagaaggagggcaggtgatagaagagggctggatccggttatcagacggaagaatagccatgccactgctgctaggagccgcagttgtactggctgtgcatgagaccacccacctaggccaagagtcacttgaaaatttgttaggccagtacttctatgtctcacatctgtcagcccttgccaaaacggtggcgtagcagtgtgtcacctgctgacagcacaatgctaggcaaggtccaaccgtcctgtctggcatacaagcttatggagaagccccctttgaagatctcaatatagacttcaccaagatgcccaaatgtggcagtaacaagtatttgctagttccagtgtgtacatactctgggtgggtggaggcctatccaacacgaaccaagaaagctcgtgttcttctccgagatctcatcgctaggtttggactgcccttatggatcggctcagaaaatgggccggcgtttgtggctgacttggtacagaagacagcaaaggtgatgagtgtggatcaaagactggaacgtagcccccttgcagccacggtggaaaggaccccagactgtcatcttgaccactcccacagctgtgaaggtagagggaatcccagcctgggtccaccacagctgcgtaaaacctgcagcaccggagacctggtaggtgagaccaagcctagacaacccctgcaaagtgactctgaagaagtcgacaagccctgctccagtcacacccggaagccgactggtccactcatggccgaagcatgaggaaactcactgcaggactcattttccttaaattttggacttgtacagtaaggacttcaactgaccttcctcagactgaggactgttcccagtgtatacattaagtcacttaggtaggacaaaaggttgctgcagtcctattattttatagttattatgagtgtactgggactctaaaaggaacttgtttgtataatgctactctatacaaggtatgtagcccagtaagtgaccagcctgatgtgtcctataacccatctgagccccctatgactaccgtttttgaaataagattgaggactggcagccgggaaaaagctgatatgagcaaaataataactagaacagaagagaaaggagttcccaaacaaattatcttaaaatttgaccctgtgtggcaatcaacagtgacttgtgtggaaatagaataagatgtggctctctagattgggaaaggggctatatagcagaaaataagtatgtttgtcatgaattgggactgaatgtagtgatgaatgtagttactgatcctgtgtcatttaggccacctggaaaaaaagatgagaagggaaggaccctgtctgccttcaaaaaggaaggagtaactcttcctgcactagtggtcactgtaacccattagaattgctaattaccaatccccttgatccctgttggaaaacaggagagtatgtaactctaggaattgatggaagtggactggattcccgagtaaatatttcagtccaaggggaggtccacaggcgctctcccaaaccagtgtttcagaccttttatgatgagctgaatctgccagcacccgagcttccaaaaaagatgaagaacttgtttctcctgttggcagaaaatatagctcattccctcaatgttacttcctgttatgtatgtggggggaccactataggatacggatggccttgggaagcctgagagttggtgcctcctgatccagttcctgacacaattccagtccagaaggcccaaactagcaacttctgggtcttaaaaacttccattattggacaatattgcatagctagaggaggaaaaacttcactgtccctgtaggaaagctcagttgcctaggacaaaagctgtataacagcacagcaaagacaatcatctggtggggcttaaacccatactgaaaagaacccattcagtaagtttcctaagttgcaaactttttaggcccattcagaatttcatcgggagtggatggctcccactggactgtattggatatgtgggcacagagcctaccctgtccattggtcaggtagttgtgtcattggtaccattaagccatcctttttcctgttacctggaaagacaggtgagcttctaggtttcccttcctatacctcccgagaaaaaagaagcatagccataggtaattggaaagatgatgagtgtccccctgaaagaatcatacagtactatgggtctgccacatgggcacaagacagctcgtggggataccgaacccccaactacatgcttaactggatcatatggttacaggctattttagaaataatcactaacgaaactggcagagctttgactattttagcccggcaggaaacccaaacgagaaatgcgatctatcagaatagattagccttagactatttgctggcagctgagggagaagtctgtggaaaattcaacttgaccaattgctgtctgcaaatagacgattaaggacaagtagtcgaaaatatagttagagacatagcaaagtgggcacatgtgcccatgcaggtttggcatgggtttgatcctggatccctatttggaaagtggtttccagctctaggaggatttaaaactcttacaataggtatagtagtagtgttaggaacctgcctagtgctcccttgtttgccacccatagttccccaactactaagaagttttgttgccaccttggttcatcagaaaacttcagcacaaatgtattacatgaatcactattgatctgtctcgcaggaagacctggatagtgaggatgagagtgagaactcccactagtgagtgaggttctcaaaggggggaatgaggagagaggccatttctcttactgcctcctgtctccaaagaaaaggaggaagtaaaaactgaaaaataacagactgatcggtgccactaggcaggcctgtaggttaaagattaacccccatcctaatcgcttgtgctatctatagatcacagacaatggaatagagaaatacttgccttgcttaccccaacttagtcacataccccatgcttgctcagtctatcacgacactgtcacgtggaccccttagaattatgagcccttaaaagggccaggaactctttcttcagggagctcagttcttaagacacaagtctgccaacactcccggccaaataaagcca

>harlequin_10220_2630_bre_polputein

eilirPqsQPgVLspdvEfLchSwSnIlWatKeQgWTAptGfcNflkPyIhftrGSeVkdKtnfSnzdRitRcKcLvGIdQifhLhVKKsNcYacahgRpEtQIVPfpLkwSSsrPgvdcmvSlfqdsTaWSnKscqAlsllCpevqHPvgQPLrAtqllsPNTkfTscLszQGgNlafLgDlkgcSeLknFQkLIsQsaLVrPqaDmrwyRsgpLLdTLpSNwSgtcALvQlaipfTLafhqtEGgKiRhrrarEApyRsfdshIyLdaIgVPwgisdkFkAznqivaGFeSifwwvtvnKnvdwInyIyynqQQriFhEfkkKKkkLmSAPALGLPDLTKPFTLYVserEKiAVGVLTQTvGPWlRPVAYLSKqLDgiSkGWPPCLRAlAetALLaqeADKLTLGQNLnIkAPHAVvpLmnvzghhWLTNARlTrYQSLLcEnlHitIeVcnTpNPtTLLLvSEsPVeHnCvEvLdsvyssRPDLrDhLwtsVDWelYvDGSSFINpqGERcAGyAVVilDaVIeAKSLPqGTSAQKAELIALiwALeLsEGKtvNIYTDSRYAFlTlzVHGAlfKekGLLnSgGKDIKyqqEILqLLEALwkPqKVAvmHCrGHQRaStSiAlGNyRgDlEARKAiSTpcRaSvTaPLLpqApdlvPtsvPTysKEEkDfLQTEggQviEeGWiRlsdGRIaMPlLLgaavvLavHetTHLGqEsLenLLgqYFYvshLSALaKtVazQCvtCzQhNArqGPtVlsGiqayGeapfEdlnIDFTKmPkcGsNKYLLVpVcTySGWVEAYPTrTKkArVLLrdliaRFGlPlwiGSeNGPAFVAdlVQkTAkvmsVDqrLnvAPlqPRWKGPqTViLTTPTAVKVeGIpAWVHhScVKPAaPeTwzvrPSlDnPcKVTLKkSTS

>harlequin_10220_2630_bre_envputein

ALLQSHPEADWSTHGRSMrKLTaGlIFLKFWTCTVRtSTDLpQTEDCSQCIHzVTzVGQkvaaVLLFYSYYECtGTLKGTCLYNATLYkVCSPvSDQPDVSYNPSEPPmTTvFEIRlRTGSRekADmSKIITRTEEKgvPKQIiLkFDpvAINSdLcGNrIRCGSLdWERgYIaeNKYVCHELGlnVvmNvVTDPVSfRpPgKKdeGKDPVcLQKGrsNsSCtSGHCNPLELlITNPLDPcWKTGEYVTLGIDGsGLdsrvNIsVQGEVhRrSPKPVFQTFYdELNLPAPELPKKmKNLFLLlAENIAHSLNVTSCYVCGGTTIGYgWPWEAzELVPpDPvPDTIPVQKAQTSNFWVLKTSIIGQYCIARRKnFTvPVGKLsCLGQKLYNSTaKTIIWWGLNHTEKNPFSKFPKLQTfzAHSEfHreWMAPTGLYWICGHRAYPvhWSGSCVIGTIKPSFFLLPgKTGElLGFPsYTSREKRsIaIGNWKDdEcPPERIIQYYGSATWAQDsSWGYRTPnYMLNwIIwLQAILEIITNETGRALTiLARQETQTRNAIYQNRLALDYLLAAEGeVCGKFNLTNCCLQIDDzGQVVENIVRDIaKwAHVPmQVWHGFdPgSLFGKWFPALGGFKTLTiGIVVvLGTCLvLpcLpPIVPQLLrSFVaTLVHQKTSAQmYYMNHYzSvSQED

>harlequin_10220_6022_bre_chaindnarm

ttgctgctattaagggaggagaccacccctcatattgtcttatgcccaatttctgcctccaaataaaaaagaagtaaaaactaaaaggcagacagcccagcgctgcgccctgggcctggtagttaaaaatcaacccctgacttaactgcttgtgttatctatagattccagacattgtatggaaaagcatcgtgaaaatccctgtcctgttctgctccattctgattaccagtgcatgcagcccccagtcatgtaccccctgcttgctcaattgatcacgaccctctcatatggacccccttagagttgtaagcccttaaaatggacaggaattgctcactggaggagctcagttttttgagacataagtctgctgatgcttccggccaaataaagcactttccttcctcaacttggtgtctgagggttttgtctgcggctcgtcctgctacatttcttggtgccccgaccaggaagtgaggtgattagcggacggttgaggcaaccccttaggtggcttaggcctgccctgtggagcatccctgtggggcactctggccagctttagcgacacaaatcctgagagcactcccgggtaggcaattgccctggtggaatgccttgccagagcagtgcacggtaggcccccatggaggatcaacgcagtggctgaacacccagaaggaattggcgcttggactccagacatctggaacacggtaagactggtcttggaacttgcccacttggaaacatggcctgatcacccatggcgtgcctttatcagcgacttggttttggttttgattttgactagattcacactgctttggtttagattttggtactggcttttggatttgaactgttttggctttgatttcagttctgacttggctcaaattgcttgatgaatgagtaactccttatccatgctttggttttagtgtgaattgcttggtgagtgagtggagtgagtgactttttgcccctttttcccttccctctttgtggtaagagcgttattctgtctcctgagagaggaaaatgtgtaaaacacaaagtaagcctaccccattagaaacgatgttaaagaatttcaagaaaggattcaatggggactatggaattgctatgacacctggaaagcttaaggctttgtgtgagatagactggctggcattagaggtgggatcaccatcagaaggaagcctagacaggtccctagtttcaaaggtatggcccaaagtaactggtaaatcaggaccagatcagtttccacacatagatacttggttgcagctggttttagaccccccacagtgcttaagaggacaggcagcagcagtgctagtggcaaagggacagacagccaagaagaaagagtacctccactcccgctggtccctcccctagaggaagaggaaggagagaggagaacagcagcataagcagctggcaaaggcaaggaaagaccagcagagagaaaaagagagagagagagacagacagacagagaggagagaaagagaggggaagagagagagagcgaaaagagagagagaggaaagagagaggaaaagatgcaaagagagaaaagaagagagagagaaagagacaggcaaagagggagtcagagagaaagagagaggcagagagagaggaagagacagagacaaaaagaaaaagagagagagacagaaagtcaaagagagaaagaaagaaagagagagagagatatacaagtagttaagaaaaaaacagtgtacctgattcctttaaaagccaaggtaaatttagaacctataattgataactaaaggtcttctctgtgaccatataacactccaataccactttgttgtcagtgtaaacaagggcgtagcgtggaagcactgaggccactgacaacctgtagccttcctatcaaaattccttaacccggtaacctgcagatggcccaaacacattcaatctgtagcggcaatggatttgctaatagaaaaaagtagaaaaataacttttaaaggaaacctcactgtaagcacacctaaccagttcagaactatcctaagttaaaaaaaaaaaaaaaaaaagagagagagaaaaaggtgggggagaatttatgtaaaaagaatgttatatagtaaactcttgtcctaaaataaattaactggttgtttaaagaaagggatgtttgcaataagtcagaaagttgagacatgttgaagaattgtctgtgaaagtagtgaaagaagaaaaaaaaagtgtgttataaaaaaggaatttttgcaagaagtgttgtataattgaaaagtaattaggcctcctgaatgtaaaactattgaagaaacactttatgtgcaaggtgtataagaaaagtaaaatatacttttagtaaaaggattataaggaggcataaaaatgtggatttttacctacattaaaaggttaaaaaaatttttttgttttgaaggtttaagcaagttttaaaatgttaattgtaaaggaaattctgtgtgtaaacatattggctaaagttaaaggggcatcatccagtttttctgtgaactggacattaaagtaaaaacaaaatgtgtttttcttaaagcactaacctgctctttaacaaaaattataaaaggttaaaaagagtctataaaaatcttaccttatggtcagacattaaaaattgaataaatatgtctacaaagttttattaaaactaagtttaacattaataacacactaatataaaggtgaaatttagcttatctggtttgaaaatcatacagaaagcattgtcaaatataaaatgttgtttggctttctttggtctaaaaactaataaaaataggtgctaaaggaaatttcccagtaagaaggcaccaagaactataaagtctgctgctgatgtccccacatttaaaacaaaaggtcaatttcttagaaattatatacttggtttatcttccactttcctttccctcaaaactaaaagtcttttagcacatgtaccacccctagaatttccggtaaaccagcaccagcctgaagattacattctcatcaaagggtagaaagaagaaaaactcaagccagcctgggaaggaccctaccttgtgctgctaaccactgagactgctgttcatacagcagaaaggggatggactcatcacacctgagtcaagaaagtgccaccccctccagagtcgtgggccatagtcccaggagaaaacccaaccaaactaaagctaagaaaaatttaactctttcatctattatattatactttcttctttcctcgctctattgctgaccatctagttattaacacaaccaagtcaattttgcctcaaactattgcatttaatgcttgccttgttataccctgtggggacttgccaagtcaaagacagctctctacttcagaaaagtacctctgtccctcctgactctcctcagactgggcattagtaaattgggaccatttaatctgaggagattttgataaagaccccagtgtcaaccaggagtcttgccccctgatgtagagcttttatgccgtagttggtccaacgttctgtggaccactaaaaagcaaggatggactgccccaaccggtttttgtaatttcctaaaaccatacattcattttactagaggatcatagaagttaaagacttaaaacaaactttggcaattaagacaggataccaagatgcaaatgcctggttggaatggatcaaatattccgtctgcacattaaacaaaagcaattgttatgcttgtgcacgtggcaggccagaggcccagattgtctgctttccactaatgtggtcctccagttgaccagacatgggctgcatggtagctcttttccaggattctatagcctggagtaataagtcatgccaagctctctctctgctatatcctgaagttcagcaccctgcgggtcagcccccaagggccatccagcttccgtctcccaacactaagttcacttcatgtctctcatgacagggaggaaacttagcattccttggagacctgaaaggatgcagtgaacttaagaactttcaagagctcaccaataagtcagcccttgttcatccccgagcggatgtgtggtggtattgtggtggacatttactggacactctgccgaataactggagtggcacttgtgctttagtccaattggctatccctttcaccctggcatttcatcaaccagagggagggcaggtgatagaaaaagggtggatctggttatcggatggaagaatagcaatgccacaactgctaggagccgcagtcgtactagctgtgcatgagaccacccatctaggccaagagtctcttaaaaagttgttaggccggtacttctacatctcacatctgtcagcccttgccaaaacagtggtgcagcagtgtgtcacctgccagcagcacaatgctaggcaaggtccaaccgtcccacctggcatataggcttatggagcagccccctttgaagatccccaagtagacttcactgagatgcccaactgtggagatctcatccctaggtttggactgcccttacaaatcggcttggacaacaggtcagcatttgtggctgacctggtacagaagacagcaaaggtgattgggtgtggatcaaggattggaatgtagccccccttgcagccatggtggaaaggaccccagaccgttgtattgaccactcccacagctgtgaaagtagaaggaatcccagcctggatccaccacagccatgtgaaacccgcagcacctgagacctgggaggcgagaccaagcccagacaatccttgcaaagtgactctgaagaagacaacaagccctgctccagtcacacctggaagctgactggtccacgcacagctgaagtatgaggaaactcacagtgggacttattttccttaaattttggacttgtacagtaaggacttcaactgaccttcctcaaactggggactgttcccagtgtatacatcagctcactgaggtatggcaaaaagttaaaactgtctttttgttctatagttattataaatgtgctggaactctaaaagggacttgtttgtataatgtcacccagtacaaggtatgtagcccaaaaagtgaccagcctgatgtgtgttgtaacccatctgagccctctatggtaacagtttttgaaataagattaagaacggtcacctggtggggtctaaaccatactaagaagcatccatttagtaagtttcctaagttgcaaactgtttgggcccatccagagtctcactgggactggccagctccagctggactatactggatatgcaggcatagagcctacaccaagctacctgaccaatgggcaggtcgttgtgtcattagcaccattaagccatcctttttcctactgcttgtaaaaacaggtgagctcctaggcttccctgtctatgcttcccgagaaaagagaagcatagccataggtgattggaaaaatgatgagtggccccctgaaagaatcatacagtactatgggcctgccacatgggcacaagacagctcgtagggatactgaacccccatctacatgctcaactagattatacggttaacaagctgtcttagaataatcactaatgagactggcagagctttgactgttttagcccggcaagaaaccctgatgagaaatgctatttatcagaatagattagccctagactacttgctagcagctgaaagaggagtttgtgggaaatttaaccttaccaattgctgtctacacatagatgatcaaaggcaagtagctgaagacatagagatatgacaaaactagcacatgtgcctgtgcaagtgtggcatggatttgatcttggggccatgtttggaaaatggttcccagcgctaggaggatttaaaacccttataataggaattataatagtaataggaacctgcttactactcccttgtttgctacctgtacgccttcaaatgattaaaaagcttcatcactaccttagttcactaaaatgcttcagcacaagtgtactatatgaatcactatcaatctgtcttgcaagaagacatgggtagtgaggatgaaaatgagaactcctactaatgagtaaagttctcaaaggggggaataaggaaggagaccacccctcatatagtcttatgcccaatttctgcttccaaagaaaaaagaagtaaaaactaaaagacacaaatgaaatccacaggca

>harlequin_10220_6022_bre_polputein

likiGakGfPskkaprtIksaadvPtfKTKgQflrnyIlGlSStfLslktkSllahvppLeFPvnQhqpEdyilikGzkeekLkpAwegPyLVlLtTeTAVYSRkGMDsshLsQeSaTPsrVvGHsPrRKpnQTkakkNLTLSSiiLyFLLSSLyADhlvInTTKSiLpqtiAFNAcLVipcgDLPSQrQLsTSeKYLCLlTLLRLgisKLGPfNlRrfzzRpqcqpGvLpPdvELLcrswsnvLWTTKKQgwTAPtGfcnflKPYIhFtRGieVkdLKqtlAiKTGPrCKcLVGMdQIfrLhIKQKQLLcLcTwQArgPdcLLSTnVvLqLTrHGLhGALfqDSiawsnKscqAlsllypEVqhPAGqppRAIQLpspNTkfTSclszqggnlAflGdLkgcsElKNfqELtnKsALVHPRaDVwwycGghLlDTLpnnwSgTcaLVqLAiPfTlaFHQPEggQviEKGWiwlsdGRIaMPQllGaavvLavHetTHLGqEsLKkLLgRYFYIshLSALaKtVvqQCvtCqQhNArqGpTVpPGizAyGaaPfEdpqVDFTEmPncGdLiprfGLpLqIglDnrSafVAdlvqKTAKVIgcgsRiGMzPpLqPwWKGPqTVvLTTPTAVKVeGIpAWIHhSHVKPAaPeTweaRPSpDnPcKVTLKkTTS

>harlequin_10210_6022_bre_envputein

ALLQSHLEADWSTHszSMrKLTvGlIFLKFWTCTVRtSTDLPqTGDCSQCIHQlTEVwQKvKTVfLFYSYYKCAGTLKGTCLYNVTQYkVCSPkSDQPDVcCNPSEPSmVTvFEIRLRTvTwwGLNhTKKHPFSKFpKLQTVWAHPESHWDWPAPAGLYWICRHRAyTKLPDQwAGRCVISTiKPSfFLLLVKTGeLLGfPVYAsREKRsIaIGDWKndEWPPERIIQYYGPATWAQDSszGYzTPIYMLNzIIRLtscLrIITNETGRALTVLARQETLmRNAIYQNRLALDYLLaAERGVCGKFNLTNCCLhIDDQrQVaEdIEMTKLAHVPVQVWHGFdLGamFGKWFPALGGFKTLIiGIiiVIGTCLLLPCLLPvrLqmIkKLhhylSSLNASAQVYYMNHYQSVLQeDmgSeDeNENSyz

**HERV3**

>herv3_10230_100004_con_chaindnarm

tgaaatataataaattgataagtaatgagctagattaagtaaaaaagagaaaactcgaataaataaaataggaagtgaaagagaaaacattgccactcatccacagaattgaaaaagaatcctacaaggctactatgaacaattatatggcaaaaaattagataatctagatgaaataacttcttagaaacacacaaccttccaagatgaaattataaagaaatagaaaatctgactgtacccataactagcaagaatattaaatgagtaattgaaaacctcccaacaaaggaaagtccagggccagttgccttcactggtgatgaacatttaaagaagtaatgccaatccttcttaatttcttccccccaaaattaggtgaataggacactttcacattcattttatgagtagcatgctgcagtttattctttatagagaatttacattgaccatttctgatgtaatgtgatgaccatctcatcacattcttatatcccaaatcccccaaggcttttaatgacatttggaataatgttccaacccttcaccttagcttgaatgaatctacatgatttcaatcctgattatttctcttgcactaaaaaaaataaatcaaaaaaaaattctactagaactttgataacttaatctatcaagcaatgtagtgaagaccatcgaaacgtttcaatgagaagatatgaaaaacctaagaactttttatcaagttccgtccttatattaaatccttcttcaatgaaagttaaagtaactctaataccttttctagcttctcattcctctattgagtaaacaattaataacaaatgtctttttttattatttcattgatttatcttaaaactcacttaagaaagaaaaggttatgtaacttatcaagttataaaacctattatgttatgattaaaagtctattttctgattctgtaaaaatgatttccttactttcagattatgtaatattaaatagacaaacccctgttagttgcatgtataaaagtcaagccctgtctttgtttggggctcagtgtttaaaatgttaatctgttaaatcagggtgcacacaataaaattcttcttttccacccattggtctctactattccttaattcctgcaacatttttctgggagctcatccaggattggagacaacaggtatgctgtttcttttgcctgtcagactggagctccaggccaggagagacctgtgaccccaggtgcaccatcagaagaacttcaacctgaaggagagatcagatgtcctgtgaccctgtgcacctccctgaaagcacaatggaacttaaggggctacaggacaattccagggacagcatgctacagaaccatggtaaggtttggggcccaaggcaggacccatcccataaggatggaatgggagcctgatcacctcccagggatgtatctagtaatccaactcaggacacaagattggctctctaattcagatgatcaaacaaacctacaccccaaataacctctgaagaggaactgagagtggaaactggtcaagaaaggtgaaatgaaagagagaagcttgctaacccaattaaaaagaaaatgggagtgtgaaagcatgtgcaagagtatgggcgatgtatgtggggctgcaagtctcttagcatagaccatatgtgccgagtgaagtgtgggaccaaccaggattaatggcaaatgtcctctgggactaccacatatggcttaggcaggcaccacacaatttagtgattgtggtggtctgggtaattaggggttatacaaaccatccattaaagctaagcagcatctgaaatactcccgcaggagaggtggtctaatcagtctgaagcaaaagtaagagagaaagaatgtattacactataactgggaggaaatgggagggaagtcgtcaaaacccaccctattagaatgcatgttaaagaactttaagaaatgttacacagggaattatggtatcaaGttgaccccccaGagGttaAgaactctttgtgaaaTagaataGcCctcTTTtagtgTtGgatggccagccgAaggaacaatagATaggggaataattggccaTGtatttaaggtggtgactggggtCagaGgacAGccagggcatCCagagacccaaTttcctTatatTGACtgtatggctaAAtaTaatccaaactcaaCcagcatggctacAGccctgcctggtagcttActGcAAaacgcTtgttgCtcaagctgaGcCtaatcccagtgaAaGaaaaatcAgCtTcaccagcAgctacgGAGAcAAAgggaAAGcCAcAggAAgggcaagaaaAACtaGtTTTgcaGgAaCcaCcaGAgGAgAcAGAaAtcCcTCCTCCcTATacccCAaTctaCCCCcgcTTTaCCaAGGCcaatGGCCcctgaGGAGtCAaatTCAGATggTgacAcaCcCcagGtttCACCccAAAgGGAgaaAtcaGAGCCcctgcccCAGGAgGTCAaGGAggaaagtCAggAtaAtCAAGcaaGCtgcCTCtacTcaTGGcCatgCccggGtTaTGCAgATGCCtcTCagGGAgActtGgGGAccCcccTCTAtTaTGATgAAcAtGGcCAtatccAAGGgGgGGcAAtggACCtTcATcTAcCAGCCTtTttCAAcCACTGATCtCCTAAaCTGGAAaCACcatactcaTAttCcCTCCTACAtggAGAAGcCcCagGCCcTcATAGATCTgATgCAGTCCATtTTcCaGACACAtAATcCAActTGgCCAGATtGcAaaCAgCTcCTcCTgAtgCTGTTtAAcACcgAgGAGcaccgaAGgaTgaCcCAggCAGCaaccagtgggttacCTCcaCTGGCtAGAAgcCaaTGtaCCaGaAGGcACAtTtAATaggcatgtcCAGgCATAcgtCTCAgGgCCAgTTCCCAGAAGCAgAccCcCACTGGGACCCAaATGATgcaaCCcAgtTtcAGcAactgcagaggttctgagaggcactcctacaaggtttgagagaaggcaggacaaaggcactcaatacgggaaagatttcggaggtgcttcagagagcagatgaaagccctagccTaggtaccgagaggcattcctgcaaggtttgagagaaggcaggaaaaaggcactcattatgggaaagattttggaggtgcttcagagagcagatgaatcctgagGCAGAggTACtgAgAagCACTctTGCAAaGGCTAAaGGAAGGtttaGaAAAaAGGcAATCAATATAGGaAAAATCTCAGAAGTGCTTCAgGgAActGatgAgAGCCCTAGCcAGTTTTATgAGAgACTcTgTgcAGGCATTCtgGCTTTAcACtCCGTTTGAcCCTaAGGCtgcTgAaAAtCAGtacATgGTAAAtaCAGcaTTTGTAGGttcctACAAGCttCCAGGGgGACATaAgacGgAAacTaaGCAAAAGCTAGAgGgTTTTgCAGGCATGAATGCCACCCAGCTatTTTAGAAgTGGCCACcAAGGtTGTATGTTAACcgTGACCAGGAgGCAaAaAggGAgGCTGATCaGAGaCTcAgGAAAAAgGCaatgaactCgATCTGCTAGtgGCAGCCCTCATgGAAAGgGAAGCTAtCATCACcAGtGGATGCAGAtggcacagaaagagaaactggcatcaccaggggtataGACtcaGAtGcgGAAGgGgCCAagctaGGCAGAGacCtAAAAGTCAgccaAGacTAGaTAGataggGATCAATGTtCAcaaTGCAAAAAgAgGGGatCaCTGGAAAaATaAgTGTCCAGAGGGcAaTtaaAAgAAAATacaACcctccgAGGCtAcaaAActatcAgaCCaCCaGCCAAgGgCcTGCcACACCcTGAGGGAgCacgCAGGcACCGACtTaATCaGGCTGGCAGgGACtGAAGaATATGAAGACTAGGcCAGACcGGGCTCCaTCTCCtTAgGcCCCCAGGAGtgCCCATGGTCACAtTaGAAGTaGGGaGgcCAACaGATGGACTTTATGGTgaACACtaGgGCTgAACACTCaGTAGTGcaACacAaCCCATAGGGCCACTATCcAAaaATTATACAACTATTGTtGGgGCTACAGGgGtCcCAGAAAaGAGGCCatttTGCtaGcCaAGGAGGTGTGTCATAGGGGGAcAAGAAGTCCAaCATGAATTCCTaggggTACCTCCCAAATTGCCCAGtTCCccTGCtgGGAAGAGAcCTtgaCTCCAaAAACTGCaAGCACAGATTgCTTTTaGGGCCACAcGGagATATGACTTTAAGCCTGACTCACCCAAAGGcCATGGTGTTAAggCCCTtAccaTcCcgCAGGCTGAGGAATGgAGAcTATAcAcAAAAaatgcccagccaccccaactgcaaaactagaggtagcaaaagtggcttgccacccgagataaAGaCACCAGAACtGtctcaTatGtcAGGGcttctatgTaAAtGAAgtggtacTGTaTaGGCTACTTAtggggagTAAAATTtcTGGAGTcATgGGtctCTgAagATAACCCACcTGGgCTGGCtaaagagtgTGtAAATCAGGCACTAGTGGTAGTAgAgctacggggctactacaTAAAACCAGGAGCAggtgccccacaactcagtagagttACtCCaGTTCaGGTTCAcCAATACCcAgaTTcCCCcAGAAGaCATAcGGGGCATTcAcAAACACTTAaAGTGGcgaCtCTtcAAACAtgGaATCtTAGtCcaATagtctagtGccAGTCACCcTGGAACAcTCCACTttTgCCagtACAAAAacCAggaaatgggagggAgtCTGgTAAATctAaACCaGTGCctctgtgAGAACTTAtaTGcaaTAAACttaagaaaggttatgcaggggaagattatagaattaagttCAgGCtACtGTaACCATCCACCCAGTGGTACCAAACtctCtGTAtAcTtTAATGGGACTCATTCTaGCAAGTGCTGCCTGGTTTACttgaCTAGACtTagaaAAAgATgttTTCTTCTaTCTttGCcTGGCACCAaTTAGtcAGCCCATCTTTgGCATTTCAATGGGAtgatTgAgTCACAGgCaCAgGGgaacAgcTCACCTGGACtAGACTCCCACAAgGATTCAAAAACTCTCCcACAATCTTTGGaGAAaCAcTGGCctCAGACcTCAAGGCCTACACCccACCAAATgACAACTgCgCCTTGCTaCAaTAcATaaAcGAcCTtCTccTAGCAGCCgcaagagaagCCAACCCaAGAGGACtGcTACCAAGGAAcCCAAGACtTCCTCCACCTCcTttATgGAAAgCTgGtTATAAtGTATCCAGGAAGAAgGCtCAAATTTGCCAgGAAccAGaGTCAAATATTaaTAGGCTTCAtAGTaAGCCAAgGGGAAcAccAGCTcAGCagTGAgCaAAAGCaGtCtgTTTGTGCACTCcCAACTaCCAACCACcCaGcgtCAAATaaGAgaATTCTTgGGgtatGCAGGGtTCTGCCgtATCTGGATCCCAAATTTCTCAcTtATtGCcAaGCCCTTATATaAAGcCACAAAgggGGGgaGAAAaGGAACCCCTCCTCTGgGAGGCTGaCCAGGAaaaGAAGGcgTTTAAACAAATCAAAGAAGCCtTAACtCAGGCCCcAGCCtTAGGACTGCCAGATATAAcTAAGCCTTTCtTTTCTATATGTCCATGAATaAAAgGGAATGGCTATAGGaGTCCTGACTCAAGTcaTAGgATCATGGCATCGCCcGGTGaCaTAgCTTATCCAaACAAtTgGACTCcGTGGCaCTAGGaTaGCCcTCCTTGCCTTAGGGCACTAGCTGCCACcaCCCTAtTGGCACAAgAAGcTaaCAAACTgACTCTaGGgCAaCAgCTGACCATCCaGGtaTACCACACTCAGTTATAACTTTAATGGATCAGAGAGgGCAcCATTGGTTATCAAATCCAAGAATGAtCTCAGTACCAGGGGCTCCTATGcGAAAATCCccACATAACTTTgGAAACAGTAAACACCCTTAACCCaGCTACCTTgCTCCCaattgAgTcGAaACCgGGAGcCCCCCTTCATgACTgTGTGGAAAcAGTAGATGAgGTATTCTCAAGCCaGggAGACCTTACAGACCAACCCCTCaGGGACCCAGActgTGtTGAATACTTcAcaGATgGgAgCAGtttcATACTGgAAGGGGTCCGtgagGCtGGGTATGCaGTGGTAACATTgGACTCAGTGGTAGAGGCTcaCAGccTcTGCCcACcgGAACATCAGCCCAAAAGGCAGAGCTAATAGCCCTAACAAGAGCtCTtTTGCTGGCAAAAGACAAAAAGttccacatctcaGCCAATGTTTATACtGAtTCCAAagaGTATGCtTTTGCCACAtTGCATGTTCAgTGGAGCTATATATAAAGAAAGagaaaaaaaAGGACTCTTAACTGCtGgGGGccaaaAAAGAAATAAAGTACAtcAAGAAaAAATTCTACAGCTCTTAGATGCTGTATGGGCcgccCCAAAGAAgGTAGCTGTTATGCACTGCAgagGGGCACCAAAAGgCAGGAACACTAGAGGCCAgcccaAAGGAAACAGAAAGGCAGACAGGGAgGCAAAAgGctGGCAGCtAATGACTACtCCaCATttTtttttttttttaAAtcAaAGGAAGCCaTAGCTaTGcCTCTCCTCCCAcgcGAgCcTCCCCTCccAGAGGagTCaagtCAAGTTActCTCCAAATGAGAaAcaagaaGGCCTGtGTTTGagaCCCAAGAAaaaggactCTGGAAAATAtgaatcATTgAAgGAGGATGGTGGaAAATTCTCcGATgGcGAGACTAGCCATCCCTGAAATGgTGGCCCCcAAATTTGTaagagAAAAaCAATTCCATCAAGGAACtCAcAgaaTGGGAAAAAtGGCACTAGAAACACTACTgGGACACcgggcacctCATTTCTATGTGCCcaACGGCTCACTGCCATCACcCAAGCTGTTTGCAAACAATGTCTAACTTGTcGCCCAGAACAACCCATGACAAGGGCCCACTtGGCCCCcgGGAATTCAGGAAATgagaaGGAGCCACACCCTGTGAAAACCTgCTTATgGACTTCACtGAGCTGCCCCaAGCAGGGagGGCTATaCAGTACATGCTaGTGCTTgTCTaGCACCTTTTCAGGATGgGTTGAGGCTTTCCCCACCcgAACAGAGAAAGCAtGAGAAGTgACTAAAGTACTGTTAAGAGACATTATCCCCAGATTTGGACTGCCTCTAACTCTAGGGTCAatGACAATGGACtGGCATTTGTAGCTGAAATAGTTCAaGAACTAACAtGgCTGTTAAAAATAAAATGGAAaTTACACACaGCCTACCAGCCACAGAGCTCAGGAAAAGTGGAgtgCATGAACcaGAtggaCACTCAAaCAGCTACTgAAgAAAaTATTGCCAaGAAACtCATCTaAGATgGGATCAgGTCcTGCCcATGGTCCTCCTCTgAGTCAGGTGCACCCCCACCAAACAAACTGGGTATTcgCCCTaTGAaATcTTGTTCgGcCaGCCACCCCcAaATCATAgGTCAAATTAAaGGTGAtaattaaggtaaCTCCgtGAACTAGGGGAAtTAACtTTaAGAAGGCAAATGCAGGCTTTAGGGATAaGCCATGCAAGatGTCCATGGCTGGGTAtGGGAAAGAATGCCCATtAAGCCTaACAGACCCAGCACACCCCTTTAAACCTGGGGACTCTGTTTaGGTcAAaAAATagAATCCAACCACTcTAGGACCCATATGGGATGGGCCCCATACTGTAATCTTGTCCACTCCCACTGCTGTTAAAGTTGCAGGAATTGTGCCTTGGATCCACCcatACAGTCaGCTaAAACcaGCAGCCCAAGACAAGTGGACCAGCCAaCAGGACCCAGACCATCCaAcCcGGCTgATCCTAtGacGaGACCaAGTTGCCatTgaAGAtaACaACAGCCCTGCTCTGGTCACTCCGGAAGCTGACCAGTCTATgCACcGGCTGAAGCTTGAGGAGaCAACAgcCCtggctCTatagtCACcCtgGAAGCTGACTAGTcTAcGCACGGCtGAAGCTtGAGtaatcaTcaaggAAgtAAATGTgGtTAgAAATCTTAAGtCtAaTAaTTTTCCTTgTAaTAtTAatTgTTTTaCTaTTagTTCTGTCaCTgTGcTCAACCTcCTcCcCttcctccctggtataagGtaAagACCTCTTtTGTCCTTGCTgggTATgAatATGCTAcaCtTTActTTGtTgtTatTcCTCCccattataatactccccTTAtctgTgttAGAAgAagaacCCAtAGAAGggTGCCCcCACTGcACtCATAtcggcaaCtACaagttaaTGGTCAGGGAcctgtGCACtATAAcCtAttaAACCCTgTTaTAtCATACTTATTATgAGTGTACAGGGACCCaCCTAGGAACTTGTACTCACtgaAAcCAGAccACCTACTCAaTCTGgcaTGACCaCAGGAAATGGCcAGCCTTATATaTGTTATaACCCtAaattcttaCCTgagtaggACcTgGTTTaAAATtCATgccagGtCAAaaGAAGGaaaCCTtcTAaaCCaAAcCaAaggtctCtcCCTCctAcagGGGggttaTaTCCtTgTacTTtaATgtaTGCCAgttAacATccatgggcTcacctTttcCCgTAatctCtagttataaagagtAcTAtaatAgattctatAaaaAtAtAtgttagccttatacaacctgccagtacaagaattaggggtatgccCcctGtTTgcTccaCCaatTtCCCAgcaAtAgaTTGcTGGaACTGCAcAActcaGTcCacTaACcaagtacaacaaCAgCAActaatgcaaAtCaAAcTtacagtaAAaaCAAactgTaaAAcAaacacTTGCaAgcTagaAAaTTTCAgtaCttTAAAtCttaaTcTcttagaatgggcTAcAcgTaTAtaGgCtaCAgTatgataccatgacAtTAcaAgTCtgtgGtcAaAaAaCagactcAaaAgtattCtaTtTAaAtAttATaaAgAaaactCaGacagtttCaacccAacAaTTctgAGTtTTtAAgTCATTCTaTaAgcATaTcAACCAGaAgtTgccTGAaCCctCTCCcTTAGCCAgAAaCCTATTtggttgaagtatatggaagcctattcagttgatgtataCTCAACTGGCTGAAAACATaGCcAGCAGCCTAcacaTcTCCTCATGcTATGTTTGTGGagGGACcAACATGGGAgACCAATGGCCaTGGGAAGCAAgAgAGcTAATGCCcCAAGATAACTTtACTCTgACTGtCtCTTCCCCcgAACCtatgctCACAAGcccGAGCaTCTGGcTCTTAAAaAaCcTCtATTATcaGaAgATtcTGcATTGCTcGcTGGgGAAAAgCcTTTACAGaCCCAGTAGGAtaGAattAACtTGCCTAGgACAacAATATTACAATgAAACAgTAgGAAAAACTTTATGgtGgGGcAaAaaaAATaATTCCAAAtcaCCcCAtCCAaGCCCaTTCTCCtGtTTCcCTTCTtTAAAcCACtCTTgGTACCAACatacttagcaagtatttggaagtatttgcaagtatcTTgAAGCTCCAAATACCtGgCAgGcaCCCTCTGgcCTcTACTGGATCTGTGGGCCAtgGGCATATtGgCaatTGCcaGCTAAATGGtCAGGGGCCTGTGTaCTtGgaACAATTAGgCtaTCcTtCTTcCTAaTCcCcCTaaaaCAgGGAGAAaCtTTAgGGTAcCCtGTCTATgATgAAactAaAAgaagAaAaAAAAgaaAtAtAcccAtacaaAAtatattaaaaaagatttaaaaaatTggaAagaCAatgAaTGGCCCCCTgAAAgaatAaTcCAAtActATGGccCAGCcACCTGGgCAgAAaATGGaataTggGGATACtGCACtCCTATTTACATgcTcaACCaCAtCATAAGGTTaCAGGCAGTgCTtgAAATcATtACtAATgAcACtGCAAaggCctTAaATcTgCtgGCCcAgCAAgCCACAAAAATGAgaAAtGCtAtttATCaaAAtAGAcTggCtTTAgACTAgCcTCCTAGcCCAGGAAGGAgGgGTATGtgGAAAGTTCAATCTAACTAAtTGcTGCCTaGAaattgatgacaatggaAAagtcATtaaaaAtAtAAggattCTgCaAaAATccAAAAATTaGCCCATgTTcCAGTcCAgaCTTggAAAGGgTgGTCTCCAGaTTCcCTCTTtGGaGGCTgGTTTTCATcctTtGaAGgaTTtAAgAccTTAgTAagAaTAGTtCTaGCCATACTAGGAggcTGCcTaaTaCTcccTTGTCTcTTACCccTCCTTGTTAaAAacATtCAAtCAgCcATAGAGgCtcTtGTAaccAgaCAaACtACtACtCAaCTAATGGCTCTaacTAAAtATCAACCctTGccatAatAaggAActactcccccttgaagaataaaaattaagtaataGTGATgCTtTCTATTAAaCtTcaTTtAtAAaAAGCATcAAAgggGGgAAtgaaacagaaaatgTtAAAaaaaAAaaaaattaaatgtaaaatgaaagtTcactcactgcaaagactcAgtcaaaggCCAGgctgtggtagGgctaggattgaaaaatcagcaaaaAaagtacctgtaaagcaagagcccaaagagcagaagtgaAaaaaacatattaaatcacacctgcttcccagCaatggtaatataAcccaataccattcaccccactcttctttgaagtgctctcacaaatacttttataactacttctggaagtcgacaaaaaTtttgtatgttcctgttttcccatccatatattataaaagttaatgattcatttatgagttataacacctgtcacgttattttttttaataactctcttcattctgctcctataagcttgCttgcctatccttcagatttcatgccattaacttgtcactgtatgattaacagcttgccaacccttttcagttgtatgtatgaaagtcaagccctatctttgttcagggctcagtctttaaatgttaatctgctgagtcagcgtccgcccaactaaaatcctcctgttccacccattggtctctcctgtcccttaattcccacaagagtgggagctttgtcctttgtttcagggatgagcaaacatatatatacacacattatatatacacacacaactgttatatatacttcgagacataaatatgcatacatacatagccacacatttatctgccatttcctctctttacttattttaccttagattgttttttccatacctgcacacataaatctactttagtcctactaattagtaaataacaacgtattgcatgggattgtcttaatgtattcagcaagtctattgattttaggttgtttccaatgtcgctactgaaaacaatccaacagcaagtatttatatataagtcttcttctgcatatgggtcagtataaactttagcatacttattagtaattcactgcttacaaattcataagaacttctgcatatatgtttctaatctttagcttttaaaaaatcagttatttcctaatgtcttttaagagatgtaattatttgattgctttcaacttataaaagagaaggctttaaacttcttgttaatactcacacttaaccttgagtggacatcactctacttctatttattgcactgaaacaagaggaaattttagataacaatgtcatgtctatatcgtgactgttctttttttacagttgtaacagagttctttataagagcatacaatgatctgttttcaaggataaagctgttcttttctgagaataaactcccagatttcaatgtaaatattattatataagagaatgggaaatatgtttttagaattttttttatagttctggagaactgtcattcaaattcccctgagtcattccaaaacttgctgaatttatgatctgaggagttcttcaaatttcagcatttcttaccagattctttttactgtaagtataagatcaactgagacttttgtctgtcttctactcctgggttcttgctctttgattttgtatcccctaggggactaatttcctcttctttacactgagattatgctcctttgctgcccttaatctaccctgtttgcactttcttcttcctttaaaaaatatctactctttttggtaatgattttataatataatttttattttgtggaattttttgatgttaccagggacttactatgtgccagagacttatgataaatagcagagtgatttgttcctatattaatttaggtaggagaaaatgtagacttagtgaagttgaagatctttgtccagtgtccacatggcctctcttttaacca

>herv3_10230_100004_con_gagputein

plqcmGgnsSkgiplefmlknfkzgltgnygvkltpqrlrtLCEiewPSFsVgwpaegTidretIGhVfkVVTgVrgQPGHPDqFPYidswlniiqtqPAwLQpcLaaYCKtLVAqAePKvKekSASpAAtEkKgKpqEgQEKlvLqePPEEtEIPPPYtPIsPlLPRpmaheEsnSDgdtPwaSPQrekSEplPQEVKEEcQnnQvScLcsGhalvlQMPlkEtwGplYydeqGhtqGGkwTFIYQPFSTTDlLNWKHHtPSYmEKPQALIDLMQSIFQTHNPTwPDCKQLLLmLFNTeEhrRvTqAALhWLEAhAPEGTlNgcvQAYaQgQFPEAdPHWDPnDaTQfqhlQRYqEALLQGLREGRKKAINIgKISEVLQgtDESPSqFYeRLCEAFwLYTPFDPeAtENQcmVNaAFVGQAQGDIRzKLQKLeGFAGMNATQLiEVATKVYVNcDQeAKkEADwRLRKKADLLaAALmEREtsItRGCGcGHRccGRGQaGQRPESQlRLDRDQCahCKKkGHwKneCPEGneensegigtkrppakgcltlkepdtdLirlagtkgyedeggpgmff

>herv3_10230_100004_con_proputein

qrtqnyqtasqgpphhegarhqlnwagrdrkigrlcktglhlpspqepMvTvEVGgQlMDFMVdTgaeHSVVTqPIGPLSKhyTTIVGATrVPEkRPfCWPRRCVIgGqEVQHEFLYLPNCPVPLLGRDLLQKLqAQIaFGPQGDiTLsLThPKAMVLTLTIlQAEEWRLytkkspelgvnelyrlltkipgv

>herv3_10230_100004_con_polputein

gaslvligalvlazrvztkqspelshkpglekvftllgkifgvwannnplslavlavnqalvivklkpgatPvqVhQyPlsqeaiaGisqhlkwllqhgnlsivvqcqSPWNtPLLpvQkpesgeyrmvyipVQnLhavnlagynpvgezalvcglvavlqaTVTiHpVvPNpYtLmglIpASAawFTcLDLKdaFFcLcLaPiSqpiFaFqwddsvtgtgeQlTWTrLPQgFKNspTIFgealasDlKAyTpPNdnctLLqYidDLLLaAPTpedcyqGtQDlLhLLwkagyivsqKkaqiCqesVkYLgFiVSqgEhqLsseqKqavcALpTpTTqcQigeFlGaAgfCcIWiPNfSliAkpLYegtKrGErePLLwEAdQeeKaFKQIKEALTQapAlGLPDITKPFFlyVHEzKGmAIGVLTQVigSwhrPVaYLSkQLDSvALgwPPCLrALAaTaLLaQeAdKLTLGQQLTIqvPhSViTLMDQRgHhwLSNPRmTQYQGLlCeNPhITLEtvNTLNpATLlPveiePGvpLHdcVetVDeVFSSqgDLtdQPaLrDPdVeyFtDGSsFILeGVrqAGYAVVTLDSVvEAqplPTgTSAQKAELIALTRALlLaKdKkwvNVYTDScfslvKYaFATLHVHGAIYKErGlLTAgGqgpKEIKyKEkILQLLDaAVwaPKKVAvMHCrGHQkaGtLEAKGNRKaDREAKqAAMTTpHfkkEALAmpLLPepPLpevpsYsPNEkAwFaQesGKYIeggwwkfSdgrlAIPemvaPKFvKqfhqgnphgkktaleTLLghhFyvprLtaitqavceqcLtCaqnnpmqgptlPPGiqenGahPcenLLmdFTElPqagGyqYmLVliCTFSGwVEAFPTrTEKareVTKvLlRdiIpRFgLpLTLGSdNgpaFvAeIvQeLtrLLKIKwKLHtAYqpQSSgKVEhMNqTLkqllKkycqiethlrwdqvLpmvLlqVrctPTkqtgYsPyEILFgwPPPIIgQIKGdLrELGlneLTLRRQMQALgIAMQeVHGwVwERmpISlTdPaHPFkPGDSVzVkkwNPTTLGPIWDGPHTviLSTpTAVkVAGIVPWIHHSvqLKpAAQDKWTSqglqDpDHltwLILrrdqfalrndeallwsfrkltslfsakteataal

>herv3_10230_100004_con_envputein

llgismllmnllllllllllllggleggpiegclhcthttrsgsaitrtllyhtycectgtrlGtcthnqtTysicdpgngqpyvcydpgslpgtwieihtgskeggllnqtkvflpgslvislylcvcqavaagyvlschknvcpplafvagipacscWdCatwstsqqslrpvmlTkipakPdCKtsTCnpVNlTILkPnLpIWTTGlkaplgiavsgygqgtgkkvyLYIIKKtqtdssatQQFrVFKSFyEHiNQKLPEPpPLAkNLFAQLAENIASSLhiSSCYVCgGTNMGDQWPwEAkELMPQDNFTLTvsSPEPTpTSsSvWLLKTSIIgRlCIAhWGKaFTDPVGELTCLGQQYYNETLGKTLWwGkkNnSKSPHPsPFScFPSLNHSWYQLEAPNTWQAPSgLYWICGPwAYrQLPAKWtGACVLGTIRPSFFLIPLkQGeaLGYPVYDETKRRNKRgitigigidigwwkednelpPERIIQYYGPATWAEDGmwGYcTPIYMLNHIIRLQAVLEIITNdTArALNLLAQQATKMRNAIYQNRLALDYLLAQEGGVCGKFNLTNCCLEIDDNGKVIeeITAKIQKLAHVPVQTWKGWSPDSLFGGWFSSLgGFKTLVGIVLAILGgCLILPCLLPLLVKnIQSAIEALVaRQTTTQLMALTKYQPLpneElivmLpLhEELnndSDAFYnpklskg

**HERV1**

>herv1_10240_100002_con_chaindnarm

tgttttttttTGtttaGtaAGCCTTATCTGTaCTCACCAGTTTCACATTCCTTGAGGCTCAGtaAGTTCCTGCtttACCTCCCTAGCaCAGCTGCAAAGTTACAAGGTTGATAaGCATATGTTACAGAAAgAtaGTTTCCCAAGGATaTaGAACATGTAGTATAgATAAATGTAAAAGACTGATCAACTGCCTTTGTTCTCaCTTgTGTAAGTAgaCTTCaTGaATCACATAGCTCtCaGCCACTGACTGCTTAAAAGGTGGCTGCaTTCTTTgTCCaaGGCTCAGACTTTCCTGGACGCTAGTCCTACTGAGCCAGGTGATCaCCTTAATAAAGGCTTTCCTGAACTCTgtTtaGTCTCTCCCGTCTCTGATTGTCCCAtAACATTTCTGGGGGCtTGTCtaGGATTGGAGATGGCAGaTTTCTaTaTCCTTTGCCTGTGGGCTAaAGCCaCAGGAtGCaGGAGATTTGaGAaCtTTGGtaCCACTGGGtAAGACTTAGCCCAGAAGGAGAACaGCTCtCtaTGTaTTGGAGCCTttCCCTGACAGtGCAAACaGAACCGACTtAGGAGTTGCAGGACAGTCACAGGAGCAGtGtGCaGGCAGACTACTGAACCatgaTAAGGTTGGGCCCTGGAAAAGCCCATCCCATAAGGACAGAAGGGGAGCTTGATtACCTtCCagGGAACaAtCACTTATtCAACCCAGAGTGGCTGGGGGtaGCAGGAGTGGaCTGCCAATTTGGATGAAtCtCaTGTCCCCACtaACAAGtaAAAGTGGTTCACTGGATCTGGAaaCAGAAACTGGGAGTGTtTtGgtggGTGtGTGTGAACCTACttGGGACATaAaaAAGGCTTgTTTCATCtaATgAGttggggtagGAGTGatttgtgtAaGTGTGTGAAaGaGaaaGtCTaataagtatgggaCAtGAGAGaggttgtttatCCaATGaaGagttggggaGGaGaGataTGtGaAaGTGtatgaaagaggtGaTCTgGGAGaaGCaaAtaggggagtgatgtggggagttgagaTCTTAGtGCAGACTGTGTgCttCaAGGtaAGtGTGGGAtaAGCCAGACCTAGGTtACTGtaTaAGaCtGACAaGATTaaCTTCATggtAGCTTCACAGCAGTAGTTGGCTGTGACCTGGCCAAGCAGtaTCCaAACCTCCtGTAATAGGACCCaGTCTGGTGaATCCaAGAGTGAAAGTGAGAGTGAAAGTGtaCCatGAGGGAGGaAATaGGAGGAAAAGtATtGAAGCCAACTCaATTAGAAtaTATaTTGAAGAACTTtAAGAAAGaCTtTtATGGTGATTATGGAATGAAGTTAACAaCGtAGaGGCTAAGAACaCTTTGTGAAATTGACTGgCTCTCCTTtAAtaTAGGGaGGgtGGCCAAGGGAACCaTAGATaGGGAAATAATTGGCtGAGTaTTTaaGGTGGTCACTGGGaTtGGAGAACAGCCtGGaCAaCCTGATCAGTTtCCATACAtAGACTCCTaGCTGAGCaTGATTtAAACCCATCtGAAATGGCTACAGGCCTGTTTTGAAAATTATTgTAAGACTCTaGTGGCTtGTACAAAACAAGGAACCATAGAAAAGACCCAaAAAatgtAGtCCCAGGAGAAAgAatAGCAAGGAAAACAGGAAAAACCTGTCCTACAGGCCCCACtAGAAGAGCTAGAGACTCCAtCCCCTTATGTTCCaATTTAtCtatCTCTGaCAAGaCTTAGGaggAAGaCACTCCAGCAGCTGtCTgtGGAGGGTCAGACTCAGAGaAGAGTACCCttCAAACTTCACtAtaTAGGGAAGAGCCAGGGtCACTGCCtGATAAaTCAAAGGAGGAAaTCCAGtATGAggttGGCCATCTCCGGTCAGGACaCGgCCGAGataTGCAGATGCCatTCAGAGAAaCTAGGgGACAGATCTATTTAGATGCACAAAATGAGGTTCAAGGAGGAGAAtGGCTTTATGTTTATCAGCCCTTCTCTACTACAGAtATTTTCAACTGGAAaAGCATACTCtCTCCTATAtaGAAAAACCCCAGGCTCTTATCGACCTaATGCAGTCCATCTTCTTAACTCACAACCCAACCTGGGCTGACTGCAAaCAGtTCCTTCTGTCACTGTTCAATACAGAAaAACACtGCAGAGTAATACAAGtGGCTtATCAGaGaCTAGAAAaaAATGCtCCAGTAGGtACAGGAGATGTCAGAaAGTaTGCTCaGCAGGCTTTGCCAATAGAAACTGACCCAGGCTGGGACCCAAATCAGGCCtAAGatCTGCTGAAgTTGCTGAGATACCAAGaGGCTCTaATACAAGGAATAAAaaCTGaAGGGAAaAAGGCAACAAACACTGGAAAGGTTTCAGAaGTCtATCAGAAACCAGATGAAAGtaCCAGTGAGTTCTATGaGAaGCTTaGagAGGCTTACCGGCaCTACACACCTTTTGACCCAGAAGtGGCAGGGAATCaGTGCATGGTTAATGCGGCATTTGTGAGTCAGGCACAAAATGACATTAAGtGAAAGTTGtAGAAGCTGGAGGGGTTTGAAGtTATGAACATTTCCCAGCTTATCCAaGTGGCAACtaaaaTGTTTGTAAATCaAGATGAAGAAGtAAAGtaGGAGGCCAAGCATAGAGTGAAGGAAAAGGCAGAGTTCTTaGCTGCAGCCCTGaTTGAAAGAGAGGCTGGATTTGCAAGAGGAtaTGGAtaTGGtCaTaGATGCAGTtaTGGTAaAGGACAAGCTAGGgtaggCCAGGTgAGGAGACCAaGACAGGTCAGGAAGGTtaGCCTAGACTAGAGAGAaATCAATGTGCGAGATGCAAGCAAAGAgGGCATTGGAAAGATGAATGTCCAGAGAAAGAAAAGaATAAAGGCaACAAgCAGGGACAGAATGGCTGGACAAGGCCTCCTTCtgCTGCTGGGCAaGGtaTAGTAGGATgtGAtaTGGATCTAATTGGGCTGGCAGGaGtCAatGaCTACCTTGAAGACTGAGACAGACtGGGCTaCATCTCATTAGGCCCTGAGGAGCCCATGGTCTCAATGGTAGTAGGGGGCtGAAAAAtAGACtTTATGGTAGACACAGGTGCTGAACACTCaGtTGTGACTCAAACAATTaGGttGTTATCAAAAgaCTatGCTAATATTATTGGGGCCACAGGTATCACAGAAAAAACAttaTTTTTCAaATCAAAGAGAtGTATaATTGGAaACCAAGAAGTCCAACACAAGTTCTTATACTTGCCaAACTGCCtaGtGCCgTTGTTGaGGAGAGAtTTGtTGCAGAAGaTGCAGGCTCAAATCTCCTTCACACtaAatagAGATATgACCTTGAGCCTAGGGCAAAAaAAGGCTATGGTACTaACTCTTACAGTACCCAAtGCAGAGGAGTGGAGACTTTATGAAaGTAGTTGtCaGGAGTGTGGAAAGGAGTACAGCatagTGAGAAAGAAAAACTGTTCACAGACTTACTCCtTAAGTTACCAGGAGTCTGGGtGGAGGACAATCCCCCaGggttagagTAAATCAAGCACCTGTCATAGTAGAGCTACTAtGAGGAAtCTaCCCaGTGtGGATgCATCAGTATtCCATTCCCATAGAGGCTaACCAAGGGATTGCAAAaCACTTAAAATGaCTCCtTGAATTTGGAATAAtAGAgAtATGTGTCTCCtCAtGGAATACTCCCCTAtTGCtaGTGTTAAAACCTTCTGGtaACTACCAGCCTGTACAAGATTTAAGGaCAGTCAACaAGGTAGCtagTAtACTGCATGCTATTGTGCCTAACCtGTACACTaTGCTTGGACAAATACCTGCTAGTGCTGCTTGGTTCAtATGCTTGGACATTAAAGATGtaTTCTTCTGCATCttaTTAGCCCCTaTAAGCCaAGACATTTTTGgtTatatgTTGaatattaaAGatgtaTtttagTatAtCTGaTTAGaCttTataAaggaatAgAtTTttgCaattaatTaaaaaCtatCtaAgTATattTttatTaGatTTttttaaAaaTTTAgtaaAaACtgggtCtCAACTATCTTTGaAGAAGCACTAGCCTCAGACTTAAAGGCTTTCACACCACCAAGtaATCGCTgTGTCTTACTGCAATATAtAGATGATCTGCTGTaAGCtaaACCCACAAgAAAatAATGTATCCAAGGAACAGAGagTCTCCTTaaAGTGCtGTGGGAAGCTGGCTATAAAGTaTCTAAGaAAAAGGCACAGATCTGTGGCtaAGGAGTTtaGTATCTTGGaTTTTAttTCTCCCAAGGaCaGTGTGAGCTTGGACAGGAGtGAAAAGAGATTGTCTGTAGCATTCCTCAGCCAGACAgAAGGtaGCAAGTGtaGGAATTCCTAGGGGCAGCTGgTTTTTGCCAaaTATGGATTCCTaACTACTgGCTCCTAGCAAAGCCTTTATATGAGGCAACCAAAGtaGGaGAAAAaGAGCCCCTCCTTTGGGGAAAAGAACAGGACATGGCTTTCAAGGAAATCAAGAAAGCTTtAAtCtaGGCCCCAGCACTAGGACTGCCAGACATGACAAAGCCCTTTTACtTGTATGTTCATGAAAGAAAAgaaAtGGCTACAGGAGTCTaAGTGCaAAtaCTAGaaTCaTaGTATtaGCCTaTAGCaTATTTGTCCAAGCAACTaGACTTGGTGGCTATGGGATtGCCACCCTGTTTCAAGGtgCTGGCtGCCACTGCCTTGTTAGCCaAAGATGCTAACAAGCTCACATTTGGaCAGaAGtTGATAATTCatGTGCCCCACACAaTTGTCACCTTGATGGAACAGAGAGGACATtGTTGaCTCTCTAACCgTAGGATGCTAAGATACCAAGGACTCCTATGTGAGAATCCATAaTCACCTTAGAGaCTGTGAATACCCTAAATCtatCCACACTGTTACgAATAGAATGTGCAGAACATGGAAAGttCCTGTTATGTGCCCCAGGGTACCACTGCTGTGTAGAAACAGTGGATGAAGTaTTTTCAAGCtaGGAAGACTTAAAGGATCAACCCTTAAaaaACCCAGATGTTGAATATTTTACTaATGGAAGCAGCTTCATATCTGAGGGTATCAaAAAGGCCAGATaTGCCaTAGTCACATTAAGCTCaGTaGCTGAAGCCtaCCCCCTACCaGTAGGAACCTCAaCGCAGAAGGtAGAACtAATAGCTCTCACtAAaAGCAaTATTTCTAGtGAAGGGAAaGTCAGTGAATATCTATaCTGACTCAAaATATGCTTTTGCaACCTTGCATGCTCATGGaGCtATTTACaAaGAAAGAGGACTGTTAACTACTGAAGGAAAAGAAATAAAGAACAAAAAGaAAATAgAGCAGCTCCTAGAGGCTaTaTGGaCTCCAAAGGAAGTAGCAGTCATCCAtTGTAAAGGACAtCAAACAGGAaGAAGTGATAAGGCTACAGGAAACAGaAAAGCAGACAAGGAAGCAAaAAAGaCTGCAATGACAGAAAAAaACAAAtAAAGAAGAGACTTATGCCATGCCCTTATTAGAGCtTCgCCTTGCAGAtaCTCCTAAtTACTtGTCCAAtaAGAAGGCaTgGTTtaTGCAGGAAAACGGGAGTTATCAGAAAGaAGGCTGGTGGAAGTTTTCAGATGGGAgaCTTaCCATTCCAGAAGCCAtTGCCCCCtaATTTATAAAGCAGTTTCACCAAGGAACACACATGGGGAAGACAGaTTTAaAGATTCTCaTAGGGCaGTATTTCaaTGTGCCAtGCCTAACTGCCATgACTCGAGCtaTCTaCGAGCAGTGTGTTACTTGTGCCCAGAATAACCCAAGGCAAGGaCCTACTTGGCCtCaAGGAATTCAGGAAACAGGagCAGTGCCATGTGaAAACCTAtTTGTAGACTTTACCGAACTGtCTCAGGCTGGaGGCTATCGtTACAtGTTAGTGTTTGTCTGCACCTTTTCAaGGTGaGTtGAatCATTCCCCAtCAGGACAGAAAAGGCAtGAGAAGTAAgCAGGATATTATTAAAAGACATTATTCCtAGATTTGGACtGCCTCTAACCTTAGGCTCAGAaAATGGACCAGaTTTTGTGGCAGaAGTAGTACAGCAACTAACTCAGAtGTTAAAAATTAAATGGAAACTGCATaCAGCCTAaCatCCACAAAaaTCTaGAAAgGTTGaAAGGATGAACTGGACaTGAAACAACTGTTGAAGAAGTTTTGCCAAGAGACTCATTTAAGGTGGGACCAGGTATTGCCCATGGTCCTTCTCtGAGTCAGGTGTACCCCTACAAAaTTAACtaGGTaTtCACCTATGAGATAGTGtATGGCCCACCACCCCCACTtATATCTCAatTAAAAGGAGATTtAAAGGAAATTGGAGAACtGACCCTAaGAAGAtAAATGCAGGtATTAGGTGAGGTAATGCAAGAAGTACAAaGGTGaGTAAGAGAAAGAATACCTGTTAGCCTTACAGATGCAATACATtttTTTCAACgTGGGGACTttGTATGGGTTAAATGCTGGAATCCTACCACCCTtGGGCCCTTATGaGATGGCCCCCATATTGTGAtCATGTCTACCCCTACTGCTGTTAAAGTTGCAGGTATTACACCttagatAtCACAGttaACTaAAACCTGCAGCCTCAGTTCAAGACaaaTGGACGAGTCAGCAAGATCCAGATCATCCAACTCaACTGATCTTGCAGAGGAACCAAGGCGCAGtAGGAAAAGACaACtaCCCTGCTaCGACCACACCAGAaGCTGGTCtGTCAAtGCACaGCTGaAGCTTAAGGAAaCaTCAAGCtCTGCTCTAGTCACACAaCTGGAAGgTGACTAGTCTACGCAaGGCTGAAGCTTAAGGAAAaaTCAAGtCCTGCTCTAGTCACACAACtatAAGCTGACTAGtCTATGCACGGCCaAAGCCTGAGGAAGCCAGCACTAGATAAGTAAATGTaGAtTGAATTTGCAAaTGTAGTTAtACTATTGCTTATACTGATTGtCTTGCTGTCATGCTATCTTTGCAAtTGCTAtCAAACTTGTTGCCCaGGAGGATGCCtGTGCATAGTATAAACTTaATCAgACTAATGACAATAATGTTAACAGGCATGGGAGGAAAtCAAGATAAtTGTCAtaTTGTATGATAGAAagTTGGTCTGGTAAAGGTATAACTAAAACCCTGTTATATCAAACTTATTATGAGTGTAataagtttgtaaAGGAACTCCCtTGGGGACATGTGTTTATAATCAAACCAGCTACTCCaTCTaTaACaCaGGAAATAGGCAACCTCAAGTATGTTAtAATtCAGGCCTCCTaCCCTaTGaCTTCTGGTTTtAAATTAAAATAGGGAAACCTTTATTACCtTCATATGCCAATCCTAAAGAtGTTAGAACTGGGAAACTCaTAAGCAAAAtaCAGGTATTCtCTTATTtACATAAAGGATCTaTTTCTATATATTTTGATGCCTGTCAGGCTGCACACCTCAGCAACCTAAACAATCTAGGAGTAGTCTGCAAGAACTTAGGACAAGAAAGAgTCAGtAGCAAGgCTGCTAAGATCATAACAGGAGAACtAGAAGAAGAATGTCCTGATtGTAACATTCAAaGGACCACAaATgAGTTCAGCCAATGCCTTTATGCAGGaAGAGTAGCTCTGCTAACCAGCCAAGAAGCAAAGATTGGTTGTGCaACTAAAAtATGCAACCCCCTaAATCTGACCATATTAAaGCCAAACATGCCTTTTTGGACTAAAGaACACCAAGGAGAGCtAAtCTtTGCTCGAGAAGaAGtAAATCTaGGTaTTCaaTTAATCATTATTAAAAAGACaCAGtGAGCTaAAGCTCAAGTCAGTCCAATGTCACAGTTCAGATTTtTCAAATCCTTCAaTAAACATTTTAACCCCAaGGAGCCAAAAGTTCAGATTCCACCAATGTtAGCtaAGAACCTAtTCACTCAGCTAGCTGAAAGTATTGCTACTAATCTtaGAGTCAtCTCATGTTATaTATGTGGAGGTACCAGTaTAGGTGACCAAtGGCtCTGGGAGGCTAGAaAAaTAATaCCAtAAGATAACTTTACCATACtaGAATTTGTTACAAaGTTCAATtgAAACCCAaGtGTCTGGCTaTTAAAAACCCCTATCATTaGAAGATACTGCATAGCACaATGGGGAAAAGACTTTCAAACtCAAGTAGGAGATAaaACTTGTTTAGaaatatgattgtttagGTCAGCAaTATTTTGAAtAGTCTAAGAACAAGACACAGTGGAGAAGCTTTATAGACAATTtCTCTgtACCAtATTTTAACtCTCTCTtTCAGTTTCCAGtaCTAAATCAGTCaTaGTATCAACTAGAtaCTCCAAATGTTTaGAAAGCACCGGCAGaACTAtATTGGAtCTaTGGGACAAAAGCCTATtAACTATTGCCaGAGAAGTGGACtGGAGCCTGTaTtTTAaaGACAATAAGGCCGTCCTTCTTCTTGCTCCCACTGAAACAAGGGGAAGATTTaAGTTACCtaGTCTATAATGAAGAAaGaAaaaggaCAaAAGAAAtGTCTTTAtTCAGATAAGTACTGTAGAAAAgATAAaCACAAACATAAAGAAGGACATTGAGATAGGaAGCTGGAAaGATAAaGAaTGGCtTCCTGAAAaAATTATCAAATACTATGGGCCAGCTACATGGGCtCAAGAtaGATCATGGGGCTACCaCACCCCTATTTACATGTTAAACCaAATTATAAGATTGCAAGCAGTACtAGAAaTCATAGTCAATGAAACAgCCtaAGCCTTGGATCTGTTAGCtaTACAGGCtACTCAAATGAGAGATGCTAtATAtCAAAATAGGCTAGCATTAGACtACCTCCTAGCCTCAGAAGGAGGAGTtTGTGGCAaGtTTAATTTGACCAACTGTTGCTTACAAATCtaTaATAAtaGAAGAGCTGTTAtGGAAATTACTGCTAGAATaaGGAAGTTAGCCCATGTCCCAGTCCAGACTTGGTCttGGTGGAGCCCAAACTCACaTTTTaGAGGaTGGTTCTCATGgTTtGaAGGCTTTAAAACCTTGATAATTGtTTTTaTAaCTATAGTAGGAaGATGTCTAATACTGCCTTGTCTTTTACCTCTCCTCAaCAGAAGCATCCAGTCTACTATtaAAGCAATAGTGGACgGAAaAACTACCACCAAAATAATGGCATTACAAAAATACCAACCaGTCCCCCAGGAAGAATAtGaGCCTACACAGGAAGAGATAAAtGAtTGTGGTGCTCTTTATtAATCTACATTTATGGtGAGCACCAAAGGtGGGGGgATGAAGAAGGAATTCATGAATTTTACAAGTAtAATCAAAGACaCAAGAAATTTTtACTTTTTCCtTCAAAAGCTAAaTGTAGTtTAGaaCCtaCaCCCtatCGTAGTCtAAGTTAGAGAAgAATACTAACTGCCTGTTTTTCCTTCTGtGCTCAGCAAGCCTTATCTGTACTCACCaGTTTCAtATTCCTTGAaGCTCAGCGAGTTCCTGCTTCACCTCaCtAGCaCAGCTGCAAAGTTACAAGGTTGATAaGCaTATGTTAtAGAAACAtAGTTTCCCAAGGATGTaGAACATGTAGTATAgATAAaTGTAAAAGACTGATCAatgtttgttta

>herv1_10240_100002_con_gagputein

rllcqadrisfiasqqswlzpgqavseppvigpslvnprvkvrvkvhreggngrksieansirmyieelqerllgdygmklttzwlrtlceidwlsfnrvagqgnlrlgnnwlsvsgghwdrrtagapzsvsihrllaehdlnpsematglfzklfktlvactkqgtiektqktsprrnssgkqekpvlqappeeletpppyvpiylsltrlrktlqqlspegqtqrrvppklhyvgksqghpdkskeeiqdevghlrsgrgrdmqMPvREpRgQIYLDAQNEVQGGEaLcvsallsttdiFnleqHTlSYmEKPQALIDLMQSIFLTHNPTWADCKQlLLSLFNTEeHcRVIQaAyQrLEsNAPVGTGDVRqcAqQALPIETDPGWDPNQAqgLLnLLRYQgALIQGIKagGKKATNTGKVSEVyQKPDESpSEFYgkLcqAYRlYTPFDPEaAGNqCMVNAAFVSQAQNDIKzklqKLEgggVmnisqliavaggnvFVNqDEEaKwEAKHRVKEKAEFLAAALvEREAGFARGcGcGrgCScGkGQARarPGqETkTGQEGwPRLERnQCARCKQRgHWKDECPEKEKnKGynQGQNGWTRPPSaAGQGvVGsDvDLIGLAGasgYLED

>herv1_10240_100002_con_proputein

DRlGsISLGPEEPMVSMVVGGrKiDlMVDTGAEHSaVTQTIgvLSKlyANIIGATGITEKTlFFtSKRgiIGnQEVQHKFLYLPNClgavvgRDLLQKlQAQISFTlsaDiplslgqkkamvltltvpnaeewrlyecscrecgkeysiaekeklftdlllklpgvwaednppvnqapvivell

>herv1_10240_100002_con_polputein

slgqkkamvltltvpkaeewrlyessvrsverstaekeklftdllpklpgvwvednppglavnqapvivellrGicPVwiHQYpIPIEAnQGIAKHLKwLlEFGIidiCVSpwNTPLLlVLKPSGnYQPVQDLRaVNqVAaiLHAIVPNlYTvLGQIPASAAWFiCLDIKDaFFclllapvpRdifaiczgpsqytwsrlsqgldfplDlKtpqlsfflflcfVlFCielapiSvaiagvqWgiSaytwlcLlgsfkspasasrvagitgtchharlifcnfsrdgvpTIFgEALASDLKAFTPPSnrlVLLQYiDDLLlakPtqkzcvsgtqSLLqVlWEAGYKVSKeKAQICGqGVwYLGFYvSQGqCELGQErKEIVCSIPQPDtRwQVwEFLGAAgFCqlWIPnYwLLAKPLYEATKgGEKEPLLWGKEQDMAFKEIKKAliwAPALGLPDMTKPFYLYVHERKgmATGVlVpmLaSwYwPvAYLSKQLDLVAMGlPPCFKaLAATALLAeDANKLTFGQqLIIqVPHTvVTLMEQRGHcwLSNpRMLRYQGLLveniyITLEaVNTLNlaTLLpIECAEHGKlLLCAPGYHCCVETVDEVFSSwEDLKDQPLnnPDVEYFTnGSSFISEGIkKARcAvVTLSSVAEAcPLPVGTSaQKaElIALTkAlFLaKGkSVNIYaDSkYAFATLHAHGAIYdERGLLTTEGKEIKNKKeIqQLLEAvWaPKEVAVIHCKGqQTGgSDKATGNsKADKEAkKaAMTEtTnKEETYAMPLLElpLADaPNYlSNeKAwFvQENGSYQKgGWWKFSDGtLaIPEAiAPqFIKQFHQGTHMGKTaLeILvGqYFiVPcLTAiTRAvcEQCVTCAQNNPRQGPTWPpGIQETGaVPCvNLlVDFTELpQAGGYRYmLVFVCTFSgcVEaFPiRTEKArEVsRILLKDIIPRFGlPLTLGSdNGPaFVAaVVQQLTQmLKIKWKLHaAyrPQnSrnVvRMNWTlttvleVLPRDSFKVGPGIAHGPSlSQVYPYKltwvfpYEIVyGPPPPLISQvKGDlKEIGElTLgRqMQaLGEVMQEVQgwVRERIPVSLTDAIHlFQpGDfVWVKCWNPTTLGPLwDGPHIViMSTPTAVKVAGITldPpQlTeTCSLSSRpvDESAR

>herv1_10240_100002_con_envputein

MHGqSLRKPALDvnvwtICKCSYTIAYTDCLAVMLSLQLLpNLLPgRMPVHSINLItLMTIMLTGMGGNQDNCHhCMIEaWSGKGITKTLLYQTYYECnkctGTPLGTCVYNQTSYSvcnpgignlqvviisgllPmgfgFeiKIGKPLLPSYANPKDVRTGKLvSKmQVFpYlHKGSvSIYFDACQAAHLSNLNNLGVVCKNLGQERvSSKaAKIITGElEEECPDgNIQwTTnqFSQCLYAGRVALLTSQEAKIGCATKiCNPLNLTILkPNMPFWTKgHqgeaifAREgaNLGvlLIIIKKTQrAeAQVSPMSQFRFlKSFsKHFNPkEPKVQIPPMlAeNLlTQLAESIATNLgViSCYvCGGTSvGDQwlWEAReliPqDNFTIlEFVTkfnanPsvlalKtPiiwkiliapmGKdlstpvgrydlfrnmTCLGQQYFEeSKNKTQWRSFIDNfSvpiFnlllsvpaansvvvsldapnvleapgeltltlgtKallllaeevtgalvlktikavllLaPletggdlsflvpslegkktkRNVFiQISTVEnIsTNIKKDIEIGSWKDnEWlPEkIIKYYGPATWAQDgSWGYrTPIYMLNqIIRLQAVlEvIVNETaqALDLLAlQATQMRDAiYQNRLALDyLLASEGGVCGklNLTNCCLQIcnNgRAVmEITARiwKLAHVPVQTWSgWSPNSlFgGWFSwlgGFKTLIIgFvaIVGgCLILPCLLPLLiRSIQSTieAIVDgtTTTKIMALQKYQPVPQEEYvPTQEEINDCGALY

**HERV1_ARTIODACT**

>herv1_artiodact_10740_100003_con_chaindnarm

tgtttaaatgttttattaaagaatttaggaatggtttggaaattaaatataaaaaggttatggagtgttattaggaaggaatgttaaagtgatttgagatggttgtgtggagaaaggtggtattataagataatattgttaaaaaaaaaaaaaataaaaaattatagataaaagatatgtatgatatattagatggtttgagagtgatatttattatgtaattaattggtaataaattttggtttttagggagtagtagagagtttgatttatgttttgtgtagaaagtgaaataaaaatggttgggaaatttgttggaggttaatttttattaatggggaaaaaaagggtgtgattgtaaagattttgatgagtagaggagatgatatgtggggagatttttaggagtgggaggttgtgagttagtgaaaagagaaattttaggtgatgggggatgaaattggtagaatgaatggattttttaggggtttaagtaatgagaaatgttttattgtattgtggtattttttttttttttattttttgtttattttttttttttttttgtttttttttgtttattttattaattaaggtagtgaatagaattataaggatggattgaaatggtgattggttggtggtaggaattgttggattgatttggtttataattttttatttCTtCTaaGtaTtAGtaTGaTaAAaaATtaaTTagAAAttgaTtTTTtgCtCttAaAaaTaataGGaaAaaaaatTtgAACAGgattttTaaaaCtatgACCAaaaCAaGGCaAaTatCtaCTtAGAtaaaTGgCagATGGaaAtattGaAgttAtaagggTGgATaTaGCTttaTTtaCAatggaTttagaACtGTgtAAAGtAatAAatTGGaAaAttTtCaAagaAtCCTtATAaatatAttAtttTtaaaaaaTaaCTGAttTTAtaGatAAatTaTgTTaaaCaaaatTtAGCattttatTAtaTGGgaaGATGtTaTGGTCatTtTtaaatAttgTtgtACtaCaGAtaAGAaAaAaCaaaTgaTaaAtAAGGtaAAtGAaaaGagtaGaTGtCtagataaAgCAAaCaCaaAataTaCTgTCAattaagtAAGGGgAGaTgtaGtCatgaaCCaaaataaGATCCCAAtgaaaagGTGGaagtagaAtGtaaAaaAtgCTaACTaGAaCAtTagtaaACaTGCaTtaCTGaaaaaAttAaGaAatGtaTaGtaAAAgCaGaaaACtaaaAgaaaGTtaaAGaAATCACCCAaaaAaaaaAtGAGaaCCCatCaGaaTTCtTgaAataaaTtaatgAaGtCtTtaAtAaATAtACtaaTatAGaCatgaAGAatCatGaGAagtgAaGGattTGataaaCATGaaTTTtATaaCaCAGagaaatCttGATaTtAaAaGGAAAtaCAGaAatggAGtCAGaaattagaCaCTagtaatGACCtTttTtgAGAaGGaTttTAatatTTTCAAtAagAtaATTttCaatAAaAGAtACAataagAGGAaGCTGAaaAaGaaAaaagCaaagAtAGtATagtTataGataCaaTtAtgaAaCatCCAagTCAGaataAgaatAaaAGttggaGaaaaACTgagaaCTGattaAAtaTaaGTGGCtaTtGaaaGaAAtAtGaaCATTAgAaGagtGAgtgCCCCTaaAAaaGGGAtTgCaaaaTgCAgaAAAAtaAGaTGgttagtaaGgAAtggagtAaagCCCaTtGgCAAaTgttaGAagAGAGaTaaatgtatagCAataGAtAGGGattgaACtCataggCagCtCtAAaTgCagtAgAttCaATTaaaATtaTgCCaaCAGaagtTCaGgttACaataGAtagTGGgaaatAaaaaaAtTGAtTTtttTtTGtaACaGtGGaGtgACtTAtTCaGTtCTtAaCAgCCtAtaaAgaagaagtaCAaagaTGACTGtAtgTgTaAtaGGAaTagACaGataGaaTAaaatAAgAAAAtTTaCTtaCagtCTTtataatGagaaTtGGtaaAgattTAaTTGaCACaCAggTTtCTgTaTaTgCCaaaATgtCCtAtaTCtaTaatTGGGaaaaGACtTatTAtgtaAGtaAAATagtGAGtgACtaTtaaCCTaGAAaAAaaaagAGTtagtGCAGgTtggAGGTaaaaatagAatGTGaaaTgCAgaTGCTaGCaCaCTTAaattGAgAaGAAtaGAGAAataaAaCaTTtCtgaaGAaaTCaAaaAtaaAgTaAaaAgattCatatgaGAtGaGCtAAaAATGtaaaaCTaGTAaAaAtAGAaaTAAAagatGGaagTaaaaTACtTgTGGAAaaAaaAtaTCtttttAAAAagtAAGaCatTGAAaGaATTCAGCCatTattaCAaAaaTTtCTagAaaaTaGaTtAATTaAaCCtTGCaagTtCCCtTatAACACtatgATtCTgCCTaTaAaAAAGCCTatttatttAataaAGAAaaaaatataaaaaaTATTGGTTTatttaAGACTTaaatGataTTAAtaAaataaTCaaagatATtCAtCtaAtttTgCCaAAtCtaTACACtTTatTaACtgatATaCtTAaaaatgtTaAGTttTTTTaCaGTGtTAtACTtAAAaGAtGCtTTTTTtTGtaTgtaCtaaTttAagaAaAatgCCAaaataTaTTTgCtTTTGAATGGAGAaatCtaGAgAtagAaaaAaCCataCAATACaatTGGaCaGTttTtCCtCaaGGaTttAaaaGatCACCtAttaTTTTGGGaaatATaTGGCaAAaGAtgTgAGatagaTAaaatTAtataAtGGAatTtTatTaCAATAtaTaGATGACTTGtTaAaaGCCAGttggAaatatgAtaAaTtagatTttAAaACCaTtaaAattCTaAAttaCCTgaCaaaGaGtaGATAtAAaGTgTattCAaaAGaCtCAaATtTaTaaAtAAAaaaaaaagTATCTtGGaTTCaatttaAaagaAGGaaaaaagAtTCTGatttgtGataGaAAAaAGGTAATaaCtGtCATTaaaGgtCCtaaaAAtAaaAaGaCAGCTgaaAGGaTTCTTGGGaATGGtgAGCTTCTGttGtATtTGgATTCCaAATTaTGaagTaAtaGaTAAaCtCtTaTaTGAaaCCtTAAAaaGagaAaAaaCAGAGtCCTTttaCTGGaaaAataAATGCCAaCAaGtaTTTGAaatCaTaAAAaaaaAgTaataTCAaGCtCTaaCttTtGGaCTaCCaaatttagaAAAaCaaTTTAagtTgTATaTtaaTGaaAaACaAGGaAtAGgatTGGGAGTgtTaATCtAaAaaaTAgagaGAtaTGtTCagtaaCttaTatCttATTTTTCAAAACAattaGAtCaAataataaaGGGaTGtCCTTgCTGttTgatGGCAaTaaCaGaCACTtgCtAatattTaGtaAAGCataaAAGtTtAttaTGGGatAaCaaaTaaaaaTtaTtatatCttAtgAtGTtCaaACTtTGtTaGAagaaAAtaGAgataAtTGtCTCACTtggtgGtGGGGaaAaTGAaCAAACAttAagCtaTCtTaTTAGAtaaCtCAaACATtaCtTTGaaaATaagtgaaaCtCTGAAtCCagCaaCtTtagTgCtaAgCatAaataaTGagtGattATGACggTGCataGAGAtgaTaaAagAGataTatTCCAGCaGGCttaACTTaTgAGATCAGattaTaaataatCtatAttgaGAattatAtACTGATGGgAatAtCTTtaTGGAaaAaaaAaaataaAtaGCaGGaTATaCatTaGTaAgtaTGaataAGataAtAGAaGgTaAaaCCtttCCtaCCaaaaCCTtaGCaCAaAAAGCaGAACTGATtGCttTtAaCAGaGgCtTTAgAaCTGtGagAAGGaaaGtGaaTAaaTGTaTACACTGACTCaAAaTATGggTTttTtGtagTGtaTGgtCATGtaGatATaTGGAAaGAaAGaaGaaTtTTAAtAtCaaAaAATaaggaaATtAaAtATaaaGaggaAaTCCTagttCTgtTaGAAGtaGTAtaattaCCtagCtAgaTaaCaaaCATttaCTGCaatGGaCACCAaAaAGAtaatTtCCaaaTtAtCaAaGaAAAtaAaagaGatGAtAaGGCtaCAAAataGgCTGgTTaAGaAaCaCAgAATGttTtGtgCTTaAatATagACttagaCCtAttaaatTTgaaAattatttAtaatAatCAaaAtGAAGaatTAGtgtgAgtaGGaAaataaTaATGgAtaatTCtaAgaTGtTtaaAAaAtTaatAtTCCtGGAAtggtCTaatCaGTaaAaaCtTAtTgtgagtttaAAaCATCTgCATGAaagaAtaCAtTATGGGagatATGttTtAttgAAtaTTGTatAgtaAaAtaaTAattGaaaAtataTTGAaaAAaagTaaAaaAaaTaACaTttGgatataaattaTGTtataAaAATAATCCtCAaACCtAaCaaaaTCCTCCttTaAgttAAagtGGTaCaatACAatGGaattTatCtattaaAAGAtTaGCAagTAGATTTtACTCAAATGCCCaaggCaAaTgtAaaaTaCAaatatCTtCTGGTGTTTGTaaAtACtTTtatagtaTgagTtaAaGCaTatCCtAtTaAtaCaGAAGGAGtaataGAAaTatatAAAtCTTTatgAAAAtAAAtattaaTAATTtCtaGATTtGaACTtCCaaaaTttaTaCAtAGtaAtAATtggCCtTatTTTaTttCaaaaaTTaagagtttaatgtaggaatagattaaagaagtgattggaaatataggtaaagttgaaaagatgaatatatttaagtgatttagaaaagtttgtaagagataagtgggttagatgtagttgattttagagttgatggttggggagtaagattaattttgagatgattatgagaggagttttaatgattgttagatggaaattaaattaaaaattataatatataattaatttaggaaggttaaaaggaatttagaatatgggaaaaaattgagttataaagatggggaaatttatggattaataggaaaaagttttaagatgggaaggaggatttggaggaaaaattggtaaatggaaggatttattggtgataaaaagtaaggtaagaggtgtataatgaggattttgtattagtgggtaatatgattaaaaatttttttaaatgttttagaatgagaaagatttatatgtgaatatggatgattaaatatgttaagagagagttattggagtaatgtggttagagatttgagtagtaatttttaaatatgaattgttttaaattatttgttgtttgtagtttggtaaaatttttattgtgatatagttatgtttttatttgtttattatgtagggggagtggtgggtgggtttgaaaagggaaaaaatttattattttggattgagtttgggtaataatttgtaatgtggatatgaatatagaggtaggggtgttttatttaaaagtgataagtttaaaaggaggtatgggaataaagtaatgattatggtaataagtgtattttgtgaatgtataaatatggtgtaaatgttagtatgaaagggtgggaaaaggggaagttgaaatgtatttgaattaaggtttttatagttttttttttaaaaaaagtaataaagtggataggggagttgttttttaatgaaaaagtatatttgaaagtagtagtatgatttttgatgtaaattgaataggatatgtgtttttgtggaaaagaatttggaaagtatataaaatttaatgtattatataagatgttgtgttataatgtataaaatgattaggtatttgagaatggaataaagttataaagaaataaaattaaaaggtgaaggtttaagtaattttggatgagttggaatagtatgtttagagaattgggggtggtttgatatgtgaggaatttgaggaagttaaagaaattaaaatgtaaagtagagggaaaatttaaaagaattaggtttagattagtaatgttgtttagaagagattggtggatattttagtgaaaagggggagaatgaagaataaaaatgttgttttatgtaaaattagggttagttgaatgaagttaaaagatttaaaaggtatggatgaaatttaaaaaatttagaattgggattataaaggatattatatgagtgaagaatttttatttttggataggtggtattttattgtattattttgggttgtatttttaattttggtaaagtttgtattttaggttaatagtttaagtaagtaatgatggaaggatttaaatatattagaaggatttgttgaggttatagaaagtagtgagagattaataagattaggatggaagaaatgttagaggaagtagttagaagatgagattttggtttttttttaagaataaggaggggaaaggtgaaatttagggggaattgagatagaataggaatgggatgaattttaatatgttttttttttttttttgtagtgataaaaattaagttattgtggaattattgtaaaaggtttagtataaagaaatagatttttgtgttatgatgtttaaatgttttattaaagaattaggaatggtttggaaataaatataaaaaggttaagagtgtattgggaaggaatgtaga

>herv1_artiodact_10740_100003_con_gagputein

MgtvtPppmvataAavlavavsagsaLlyEaaDggasaapppqynkgvtkVfvaflamglglcqGKfgtfskniptciiaygyLglpIgfvwlylaFSlsgvllWsfwnpvazrepsendzsyrlllckpsallglclVsmayaPpssqyavvivkgneetwclhkantngtlnppdAVlAmnPnWDpNekveaggasLsrlvlcltsGmtkkgvaKavnldeVqaITQganEsPavFLnhvtqalkkYTsagmsgpEgsallaMtFIpQStlDvkgKprsweqspialgmpffRwlvllSitgfsitetgqrrlmncLaKIaWlsAafaktppsiyGDptepsksdnelKlgglgglnfagYcREgpwkRltPpctgIqwlaggggqLgplasasggtslwlwq

>herv1_artiodact_10740_100003_con_polputein

erelsprglKqTdktpdeknaklvyieislGvgilWKqylplKpealngIQPvlqkFLqlglIkPCsfPcNTlILPvkKPhfsilrektphSgeYWFaqDlsavNeavipIHlmvPNlYTLLTaIlksasfFsVLylKdaFvCvllypegQmlFaFEWRgldmqtafQYcWpVLPlGfkcaPiilgdaLAndvRclsLwnGvLLQYvDDLLiASwtgqdllfnTilvLNfLamsgYKVspKaQIclqnitYLGFllipGariLaggwKqVIaallaPetkRQLkGFLGMaSFCwIwIPNfgvmaKlLcEaLKgldsEplpWtteCQqaFdalkteLgsALpLGLPglpKpFiLYvcgklGigLGVLIyklprivswlvayFSKQlDpvaqGwPsCLmAvaaTallvsaasfliGlpvisymlvPgsvlvllqqtggswLvgGqltKHqalLLDtpnIaLsItvaLnPapgvpatdpvglhdClEmldqffSSglnLsDQmiivlyaElyTDGsiFvEngthmAGYavVslveviEgyalPpqsfAQKAELiAligALqLgqGewvnVYTDSKYgFLavcgHaaIWnERglLiaeNspIkypvpvLaLLEaVslPthvaiiyCpGHQkDdflvingnnavDkAkAaWaaepsNalglmIdltlLllkPlYsqqdlglacnwGvapagsnllipgTllewlisvlpdaaqfgtivlwnivmggliqsgLcsttIgafasEllfqnspgdpfqvipvilspltlvlrkGtctmgcaclklagdFTQMPpalGncklLLVFVnTFigwveAfPiksEGavEvvKaLpKzilIIprFgLPcflqSnNgPsFvaavTqsvSqalnilwtvpswepsswgkvekmiiilklilqnlsqetlSWvtvLPvALLcvtvAPwgaLrLNpfEiIYeRpFlTtdlLLpGilpzkllyilNlgqVqqavselGniILpvSttvglillwlspgaaVllaaGvefflienpLaqwqalslvinaPisvcycsvtlgilpppmvrltsstp

**HERV4**

>herv4_10760_100005_con_chaindnarm

tggaggcagggaacataaagccaatccacactgacttcagggaccttaacatcaaccttcgattgaactcttagaacttaacaaaaaggaaaaccccactttccatacttaaattacaaattttccaattacatatcttaaacaactaccaatatcctatacataatcagtgggaaatacttttgataaaataagtgacttcactttaatcacttcatagttccattgatctgaaaaattagagcatcaatacttcattaacattgtaagaaacttaacagagtctattaaattgggctattcgaaagtaatatattttagtaaaaaaacctagagataatttcaaatggcgattttgagcaatttgtcgattattcttctaatctgctaagaatctgaacactttctccttgtaaaatctatatttttcagcaaaaattggaaagcaAaccatGaGgtattaaggctacagTttatgatttatatatcttTttcTcTctcTCttTtcctTtTcCaACttgggAccttcggtggacAgCgtctaaaCAtggaggtcCAaCTGctaAggTtTctgGcCggGgCcaCtctgtgGTgAaAcaggtttccaTGaaagGtaTctaaccaccaccacccggctcggggaataacctgattcccataacctgGttTtggTgagggaccctattcctttctcccttttacTGaGTtcttttctTTttcagtcttTcaGtggctgtgctattcCTagtAGcccctgggtAatTGAgggcatagtcactGgccgGGaCcaCTctccagTGttacttgcAAggCgaAggaatGaAtggGgataGcTggCcTgcccagAaGgggaAAggaCTaTtttatatcTtctatctTatAgtctCTgAtccCtAcatgTGaCagacagatgatTggcaGtggcaGcttaTcCAgggcgaatgTcaCAcacatttCagataaCtTaaaccttctttcaTtaTaCaaaataaattcttcacttcacaaacttgatatgttcaagacAagttGggggTTctgctatgTtttagatggtctgtgagattcgtgaattAcaAggtgtggATtcaTggaagatcatctattataatTtgctacaatctTgcctaaattTatagagTtaaagaaTTgtgaTTTaagtgggaTaggaaaaCCcaAcaagggatgtAattgtaaaagtggaagcaTtctAaAatggctgaggtcttcatCcagGgacaaaaagGaataacaaGttcaaatttggccatcAagggtgAAagcagaaagtggtgCcagtaccCatctaaggtcagagatatctgaCAgactAAgtcaggggatctAaagtggGgaacCccctagggaccCcatttaggacCcAAaacttttcCtctagtacAccCCgggtataaAaTaTGGGtaaccaaataaaCttTgctaAggatctcttCCaGAtcaCTaTggGgaaAactCtCCATCTATgataCACCtGATTCacctcTtGGCtACcaatcctccaccgattggaatcaatttgaccctgacaaTCTaaAaaggaaaCattTGATTcttcgttgcaatattacatggcaccctcagtatgagctgcacagtcaggaacaatgggcagTcaatgGtatCCTtaAttATtagaCACtATcCTGCAATTAGaCCTATTTTGtAaAAGgCAGGGcAAATGGTCAGAAATCCCATATgTAttCAGGtCTTCATGGCccTATAccAAAAcccaacaatctgtaaaactccaggacctgtcccccaaaggaaagtCCtAAggcaGaactAGATATTaTAGATGAtCCccTTTTtACAAGGGcCACCTGTCTCtcAGGGggAACaGcAACCaCCCCCATATAGCCCCTTGCCAAGTGCTCCTGAGGCTaAAACCgCAgcttcaaaaACaaGGAACCCaTACTAAGTCCCCCTCACActtggAGttGAACACCaTATTtaACTcTCCcTCTAgCCCTaCTACTcCTTAaGGAAaGTAGCAaGAGCtGAGGGGCaAcTtctAGTGCAGGaCtCCTTCTCcATAACTgATAtaCAACAATGTAAgGAAAAGCtaGGAAGCTAttCTGAGAAtCCTaGGAAaTTTGCAGATGGgTTCCaaactttgactttagcctttgattTcTcAtGgAGaGatgttcaAtTtATTCTAGCAAccTGTTGCACCCCtTtgGAAaAGGAACGAATCTTTGAGGCCaCCCaCTagGAAGtaGACAATTTATTtGCCtgAAACCcTcAGGGCAATCACCTGGGCCCAGACACagTCCCCACTACTGAtCCTAATTGgGACTAtaACACcCCtGTGGGAATGAACAACTGGGCTAAATTTCTTGAGGCcCTttCCTTGgAGGAATGAaAAAGGGAATAACTgAAGGCAGTAAATTATGATaaagTaAGGGAGaTTACACAaGGCAAGGAGGAAAATCCAGCCATGTTTTATGGCAGGCTGgaGGAAGgaaatatgccaatctggacacttcCTCTTTAaAAgcaaaatattaatgcaaggaggaaaatccagccatcttttatggtaggctggggaaaggAATATaCtAATCTGGACCCTTCCTCTCCcaAAGGTAAaaTATTAATGGCACAaCATTTtATTAGCCAATCCACaCCAGACAtTAGatgcAAGCTCCAAAAGCTACAgtATGGgGCCACAAACTAAtCAAAATCAgCTTCtTGATACTGCCTTtATGGTGTATAACAAtCGTGATCTGGAGGAAGGAAAAAgggAACAGAGTAAAGAAAAAcCagCAaGCCAAAATTATGgtAGCCATCAtTGGcgATGCCCTGAATGCCCAAAGAGcaTCCAAGGGAAACCCAAAGGGCCAtAAaGATaATGCCAGCAAGGGCTCTTGcTTCAAgTGCAAGaAAaCTGGGcATTGGaCAAAGGACTGtActAAGCCcCcgCcagaaCCCtGCcaaaAaTGTgAagGCaCCAGTTATGAcCCcTGGCACTGGAGataTTGaCTGCCTtcaCTCCCACCAAGGAGCTcaGTCAGGCAAAACTCtcAGCAGTGCaaAAGGAGGAATcAGATGAAGACTGAAGgGGCCCaGGGtCTTcCTcACtAcCCCTGTcCAGGAACATCatacATTACTACTGAGGAGCCCTGaGTAACTCTGGACaTCATGGGCAaacaaattcagtttctttcttttctagCCCAAATTCAGTTTCTTTTTGATACAgGGaCAAATTActtTGtcCTTACTGCTtATgCAGGAAAACtTTCctCccGGTCcATGAGTGTTATgGaAATgGAAGGaAAGtCACAAACAAGATTcTTTAcTCCTCCTtTgatTTGtCAATTTGAGAAAcaaaTCTTCCAACAGGAATTTCTAGtAGTACCAAGCTGCCCAGTCCCCtTGTTGGGAAGaGATATTATGGTTAAAATAGGGgCACTaCTACAATTTAAGcaatatCACCcaGtaAAATTGCTAatagtcAGtAATaCAGACAATGtCCCAGACCAcAttAATAAACAGGTTAACCCACTgGCATGGtATACTGtcGGAAACCgGGgAaGGCTAAAACAGCAGTGCcagtCAAAATACAGCTTAAAGACCCCAgCtaTTTTtCCAATCaAAAACAATACCCAATTAAGtTGGAAGCAAGAAaAGGCCTAGCacccATAGTTGAGgTATTACTTACCCaTGGaCTCTtaAAACTCTGtaATTCTCCCTGCAAtACCCCCATCTTACCCgTTCTAAAGcCTtggGgggAATACCaGtTAGTACAGGaCCTCAGAaTAATTAATGAGGCTGTtATCCCTGTCCACCCATTaGTGGCAGATCCATATACCCTCCTGgCTCAaGTaCCAtttaGGGATgCAAAATGGtTCTCAGTcCTAGACtTAtaagaTgCTTTcTTCTCTATTCCTCTGGCCCCAGaGTCCCAAtAcctTTTTtcCcTTtGAATaGaAAAATCCTAATACCAGAtAAAAAcaAtAataCACTTGGACAGTGCTCCCTCAgGGaTTTtGgGATAGCCtCCAtTTCTTTGCCcaAgCCTTAGAGaGGGATCTGAGGGATcTGcAActGGAGAATGGGAGTATACTcCAGTATGTGGAtgAacttCTTGTgTGTAGCCCAACTCAGGAaGCTTCTgaCcAAAATACTATAAAAACtTTgAATTTccTGGCTGACAGGggATACAAAGTGTCCAtgAAAAGAAGGCtCAGATTAccCTCcAATGGGTCCAaTATTTAgGGTATGTCTTAAcACCcaGctAACCcgGCAAATATCCataAAACaAGTGCAAGCTATATgtGGtTtGggtcCCctatgcccgcaacCCAcCCcgagCcGCagCtcaCCtCcAagcagcagctttgttctTTTTTGGGAATGGCCttAGGTTTTgcaGaATATGgGtACCAAATTTTGGGcTCATaGCAAAGCCCCTaTATGAAGCAACAttAGGtGGCCTGAAAATGAgCTAATGGAATGGAcCCCAGAaATGAGGGAAGgggcCCTttgCCAAgCtAAAACAGGctcTTACCCaGgCTCCaGcTcTTGGgatCCcaGaCCTAACTtggaAgcccttctccttgtatatAGCAGAGAAgAagGGCATAGCTGTGGGAGTGCTAGCcCCAGAAaTTAGGATCAGAACcCAGACCAACCACCTACTTTTCAAAcgAAGTTGGATgGAGaTGGtCTCaGGATGGCCAAGTctTGCCTcGcgGGCAATAGtagCAGCCACTGCTATGTTAGTGGAGGtactAAGCCACcgcccTAAAATCACCCTGGGcCAACCACTGGAAGTtcTAAccCccCATCAGGTAAAGTCAGTCTTAGAGATAAAAGGACACATCTGGATGATGaGGGGAAAGGTTAACCAAATACCAGGCCATGCTCCTAGACAATCCAGATGTAACCCTTAAAACCTGTAACACTTTGAAaTCCAGCTTCATTGCTgCCCAtAGGCCCAATAACTaATCATTCCTGtGAGactCAGGTCATTGCAcACACATATGTTcAGCCgGCCTGATTTAAAAGATCAGCCTacactgtcCTCCCAGATTCTGcAGGATGACTGgTTCACAGAtaGCtgAGTAGTTTTGTGTCAAATGgggagcactgagcggataGGGAACActAAGCtaGATATGCAATtcAGTAAATCACAACACaATTATTGAAgCCCAGCCACTGCCCCCTGtGCACATCAGCACAAAAGGCTGAAATCATTGCTCTTaCtCGAGCgATTAATGTTGGGACAAgGgaAAAAGCTTAACATaagCTATACAGATTCTAAATATGgCATTCCTTGTGGTTCATGCTaCATGCTaCAATCTGGAAAGAaagagaaagaGGACTACTAACTAGCAAACACTCCCCtATAAAGCATGGGCCTGAAATTCTTCAGCTATTGGAAGCAATACACCTGCCAAAGGCCaTAGCTATAATtCATTGTAGGGGGCATCAAAGGGACTTAACCCCTATAGCACAAGGGAACAGAAAGGCTGATAGAGAAGCtCAAAGCtGCAGCCCTCAGgGacccTGCAATCCCAACAGcaATCCTAGCACTGcTTCCaTTTCTATGATTCCCCAGTaGAACCTaAATAtACAcgtgatatAGGAAGAACAGTTAAgTAAAGGAGCAAGGgGGACaaaaaaaaaaaaaaAAAAACAAGGATCCTGGTGGTATATGGGATCAAAAATATATatCTCCCTCAAACAGCCCAATGGAGAaTTATAAAAaCCCTGCATGACTaaacaccctgcatgaatCTTTCCATATGGGGAGAGATGCcACTCTGGCCATGgTAAACAaGCTCTTtATTGGGCCTAACTTAGCTTCgGTGGTTAAGCAGGTCTGTCAAGCCTGCTCACTGTGTGCACTTAACAAaCCAGGAAACAAAATGCCTCCTCTAATAGAACCAGTCCAGAGGAGAGGAACTTAtCCAGGGGAAGACTGGCAATTAGACTTCACCCATATGCCAGCTTGCAGAGGATACAAGTTTTTGTTAGTGCTAATAGATAtaTTTACttacTGGTTgGGTCAAAGCTTAcCCTaCtAGAACAGAGAAGGCTAATGAaGTTATAAAcacGtTTCTCTTggggtggggggAAAAGAAATAATCCCCtGGTaTTGGGTTACCTCAGAGtCTCCAAAGTGAtAAtGGcctATCCTTTcaacaATCTCCCAAATcacaAACTCAAGGccaGGTTGCTAcacaAGGCTCTTGGAATCAAAgcTACTATTTACATTCAGCATGGAGGCCTggtgctccaCAATCCTCTGGGAAAGTAGAAAGGGgcccCTAACCtgAAACTCTAAAATgAGCgTTAtttcccttGCTAAGCaTATGTCAGGAAACAgaatttTCAGcgtggccAAACTTGGGTCAGCTTACTGCCCATAGCCCTtTTAAGGATcCGTAATAgtctcctgCCCCTAGAGCAAAAATTAATATAAGCCCATActggctgaaTaAAATGTTATAcagaaagaattgaGAAGGCCATTctaattTTAActAaTgAtTTAATTgaACTGATCCAGAAACAGCcaGTTTAGTAAAATACCTAGTTAACCTAGGACAATTTCAGCtcaAGGCTTTACAAAAGTTcTGGAAcTCAAAGGCTCCaCCATACTgGGAACTAACgCAGCAACCCtctgggaaagAAAATCAGGCCAGGAtctGATAAGGTACTTGTTAAgctatgccaggaAACAtTGGAAGGAGGGATCAgCCTGCTCAACAAtTaCAACCCAAATGGAAGGGACTgTTTTCAGTGgTACTGGCCAtaCCTTCTgtggTCAAAGgcTAcatAGGATTAGATagtTGGATACATCTTTCAAGGATCAAgCCaGcaATACCaGAtttAGccCcaGACCaGGtAACCTGAAGtaaTtCCATCAGcCAcTAcAcctGTttgGAACctgtgGacttcccagacCtGaAgttaaCCtGtaaTTaaAaGacAGgcCAAAAGATAAGTaAATGCCTACCAACTtTCcTTGGTGtcTtTGtTGCATAgTTACTGTAGGCTgGATAATAGtAGCCataTTTttatttTTtTtGcagtTTaatTGCCTTCTTcCAAAtggaTaGAAaTCAcTTCCTTTGTAaTaAtTAAgCAGAATgTttTAattaCATTtcTaTAACAaatATTCCTGAcAGCATAgGtATCCACcCCCactacccgaagacctaAAGTTCCCATTaAAtcttttAACctAAtTcaTTTcctcTCaCaTAgaGAttAtCaaggttCaGattATcctGgaACaAGGttTcCAGCctaaTccaagtGaAGgcacCacCCCatggCCaTCaaGaAactAccctaTcTccacTaaatagataAGggtgagAgtTctaagAtCaccaAcaGgTAaggACtACacctCaAGtcaggatgAAGCAgTtacaGaAgaaagAcCATCAgtCactCtgtcTcCCATaaagATttatGGggATCatGtctCTCaagGaaaAaATGAggcAggagAatagggtctggAGGcAGGGAaCCTaAggTtaaTgcAcgaTGACTTcctaGAacTcAAttaattacaaaaacgacAAcacTtACatTgacaggaTaagcagtAaAagttCtccaGtcctatcaaattaAccTttcttggattgcatctcttcaaaaacCtaTctGgagcccaacatcgAttgacaaaGaaCaaagactccatgtGcacttTgtAttaAaAgaagggaaaattctggtcaatccctcttggttaatttatgcaaaaAatttGatgttTcctCAagggtgttacaccttaggcattAaccCatcccctgAtcatgTgagtTaTGCAattactcAgAcTgcATtatttggtaaacCTgcattgttttcAgGtgaacaacaccTAgttaatgGgatttAAagtatagagagTcTaagtActcaaaaagatctagtaacagtcgcggTtGggctaCtagtTgtGtttacattttcacTtgggaagattgTtttAatttcaAgaaAtCtagatgccttttacttccccactaTGaaactacCttTattTGgTTtgtggtGaatattaagCAtaacTatcTtCAaaatgctaAgAaccTagctacaagttgaagTCaagAcaCTttaCaAGgttcttaCcagcttttaattgGagccatcttaagaaGgactTtAacaatttttgAaAgaaaaaacaaAacacaacatcatatgttctaaaccatAgagaacAaatTttatcatgaaaaacaagGaGccttTttcccatgtGgaaatgGttgatagataTgtctgccaatcagcTgtactgagaacatgggtAgagttgatcAtataGcTctggaaaTcagtataaatctaaataacaagTctctcattatatCtctaactacaaccgccaggcgcaaatgaaccatccaattgataCcctttTTtgtaagagttaggaaaAactgcacaagtaGaaacaggatttagcaggctTgcaactttgctatcctattatgaatatgcttgTccaaGgAgctttgagaaagtttggaagacattgtccaaagcattgtcatacttacaaaatcatatagactcattagcaatggacgttttacaaaataaaaggagactggatttcctaactgctgaaaaaggtggcatatgtctttccataaaagaagaatgctgggaatgctgtttttatatcaaccaatcaggattagtaagagattaagtctgacaattagctgagtgggcctctaaaataagacaacagctgtccgagtcatggggctactggtcaagggcactaagttgggctgtcatggctacttcccctggctggcacattattaacaattatgcttgaattgatttttggaccatgtttgttaaatcttttaaccagatataatttttcttacctagaggccattaagcttcaaatgatcatgtagcaggacttcctgttccaggaaaaaatgccagccccggccaacaagaagtcatcctgtctccactagacagaacagggtgagagttccatggtccccaatagatagggacagagccccaaacaacatgaagcagttacagaagaaagactgtcattcctatacctcctgtaaagatttgatattaagtctctaaggaggaaatgaggcaggagaataagatctggaaagagggagcctaaaacaaatctgtgaatggccacgagaaatttggccaagagtataattccacttcaacatccaatggacaaagggcaagaacttcctttacaagcctggggtgtgtgtgtgtgtgtgtgtgccctgagtgttaaaagtgcatatgaggcaggagaatagggcctggaggcagggaacctaaagacttcccagaactcgatcaaactgaaacactttagctatgacaagaaatatcctctttatttacatggtaaatgactttgtaactttacttcgtcctcttcatttacatagagtgtacaccaagtaaataactttgtaacttcagtttaacctcctcattctagccatacaccacataaccaatggaaacctctagagggtatttaaaccccagaaaattctgtaaccgggcccttgagccgcttgcttgggccccctcccaccctgtggagtgagctttca

>herv4_10760_100005_con_gagputein

lglplalssilfqtligngplvltqnsSgrtPrIkfGSplgylLksrPlWapdNLKrsHLILlCnTvwlfYeLPslEqlaVNGsLNYDtIlqlpllcKRqGqmVeiPictgLhglipnPlilgilaglagkgkapggaryyRzpPFlkGalsLRvnsnhPhItPcQvllrlKPlgsTgdltkSPsHsevnTifsslsiaLllLREVAGAEGPvLVQAPFSitdIQQCKEKLGSYSEnPrKFADGFQTLTLAFdLSWRDvQFILAtcvtPpEKenifeAahwEvdNlFpeNPQGNHLGPDTvPTTDPNWDYNTPmGMNNWAKFLEALLGGMRKGITKAVNYDKVREVTQGKEENPAMFYGRLEEAFkKYtNLDPSSPeGKILMAQHFISQSTPDIRcKLQKLQMGPQTNQNQLLDTafMVYNiRDLEEGKREQSKEKqQAKIMAAIIGDALNAQRASKGNPKGHKDNAsKGSCFKCKKpGHWAKDcTKPpPgPgqqceaasydPwhwrcwlPplppgssvrqnlisssakrGlphcpv

>herv4_10760_100005_con_proputein

RGPGSFLSTLYRNTVVTTEEPZVTLDIMSTQIQFLFDTGPNYTVLTAHAGKLSPGSTSVREIEGKSQTRFFTPLSIZEIFQQKFLEVPSCPVPLLGRDIMVKIRALLQLKCHPVKLLEVKNTDNAPDHTNKgvnplawhtgkpgkaktavpvkiqlkdpnyfsnqkqypiklearrglapivevlftpgllklcgss

>herv4_10760_100005_con_polputein

tmvinrltplawitgklgkaktavpvKIQLKdPSyFPNqKQYPIkLEARKGLAPIVEvLLThGLLKpCNSPCNTPILPVLKsGeyqLVQdLRIINEAVIPVHPLVADPYTLLaQVPgDaKWFSVLDLkdaFFSIPLAPaSQYLFaFEgeNPNTReKqQYTWiVLPQGFwDSPHFFAqALERDLRDLQLENGSVLQYVDDLLVCSPTqevSDQNTIKTLNFlADRGYKVSKKKAQITLQWVQYLGYVLTpgtRQISmeqVQAIcglaslpptlplpPTpsaqlcsFLGMArFcRIwvPnFGLiAKPLYEATRgPENELMEWTpeMREAFaKLKQALtqAPALGIPDLTKpsLYvAEKkGIAVGVLaQKLGSElRPTtYFSKKLDgVAlGWPSCLlAIaApAMLVEEATKITLGQPLEVLtPHQVKSVLEIKGHIWMmGERLTKYQAMLLDNPDVTLKTCNTLNPASLLPtGPITnHSCEQVIahTYVSrPDLKDQPLPDSEDDcFTDgSSFVSNgEHzAGYAiVNHNTIIEaQPLPPGTSAQKAEIIALTRALMLGqgKKLNIYTDSKYAFLVVHAHAAIwKERGLLTSKHSPIKHGPEILQLLEAIHLPKAIAIIHCRGHQRdLTPIAQGNRKADREAKAAALsVQSQQILALlPFYddSPVEPeYTlqeeqlIKgKGgKnKDPGGIwsgKYISLpQsPQWRIIKPLhDSFhmgRDATLAmvNrLFIGPNLASVVKQVCQACSLCALNnPGNKMPPLIEPVQRRGTYpGEDWQLDFTHMPACRGyKFLLVLIDiyTFTGwVKAYPTRTEKANEVIKFLLKEIIPwFGLPQSLQSDnGlSFISQITQGVAKALGIKYYLHSAWRPQSSGKVErANQTLKZALAKLCQETSETWVSLLPIALLRIRNTPRAKINISPYKMLYrRPFLTNDLITDPETASLVKYLVNLGQFQQALQKFGTQRlPilGTNQQPKIRPGDKVLVKTWKEGSPaQQLQPKWKGlFSVVLAtPSvVKVLGLDSWIHLSRIKSvIPEAldqepfigpgTsssvqpLklllwtt

>herv4_10760_100005_con_envputein

rqlvnsgyllrtlikhkdckyhqstfgkallassisiiiqicchpnpnpilglzkndlshlnhkgripfklmnlddlydskvtkltKVTmlGztvnLLqsyqsnlsgvtspqkplfGaismttnlnfrapFVLKnasilvgpfvlkeakillSHQssYTLGINlSHDcvlcavlgtaifrKpvmvsgKlfLvKGTLKDsQSKYzvlskyCQGRpTSCVHIFpWEDCsNpeTaSCLLVlqYENTSgWLLVDTKHNYLHzgeNlTagaTqDTSQGpFQPLigatLaRtLttWgseNnKLTHmFTIENnFcLEKqgaFFlCgTSSyLCLPanWTeisTLvYLAPrsIClaltslSLIpltapaaqmntitlalllvgLGITAgVgTGVsRLATfLSYYQrLSKDLSdSLEnIaQSIVTlqNQIDSLAvvaLQNnRgLDfLaAEKGGlCLfveEECwgfllllleeccFYvNQSGLaRDagZqLAdgASeIrQQLSeSWGfWSRALsgfaSwlLplagilliialvlivGpCLLNLLTkfifScLEAIKLQMIMaggfypgndaspgqqeviLpalsRagzefhgpqvdrdsapqheavteerlsvplppvniilslsrgneage

**HERVI**

>herv4_10250_100014_con_chaindnarm

TGaaaAgatGaTTtTaGTaTGAGTGAAGGTATAGAAGAtAAATtAAGTtatAAAAATGTTTtTtTTGAAAAGTAAGAAATGATGTAATGCATGTCTCAACTGAATAACTGTCTTTGTTTCTCACTTCTGTAATATGCTTCCCtCTGCACAGATCTCCCCCaACCCCACAAAATGCTTAAAAGGTAACTTGACTCTTTGTTCAGGGCTCAGTCCTTTGGATGTTAATCTGACTGGGCTGGTGCACCTAAATAATTAATAATAAATCCTCCTCAACCaCTTGGTCTCTCTGATTCCTTAATaATCCCGCAACATTTCTGGTGGCCCAGACaGGGATTGGAGATGACAGATTTACTGTCTCCTTTGCCTGTGGGACTAGAGCCCTGGGGCCAGGGGAGACCCAGCATCCAAGGTGCACCACAGGGGAGCTTCAaCTGGATGGAGACCAGCTCTCCCTGCATCCTGGCACCCTGCCTGGCAGTGCAATGGAACTGGGGATGGGGCTGCAGGATGATACCAGCACTTCAGGAACTGCGGTAAGGAGAAAGTGCCCAAGGCAGGAAAGCCCATCCCATAGGGAtGAAGGGGAGCTTGATCACCTCCCGGGGACTGACCACTAATCCAACCCAGAGTGGCTGGGGGTGGCAGGAGTGGCCTGCCAATTTGGATGAACCTCATGTCCCCCTAACAAGTAAAAGTGGTTACTGGTGGAGAAAATGGGCTGATAGAGCGGCAAGTGCAGCAAGGAAGAGCTTGCTGGCAGGGTGGCAAGAGTGGCTTGaACCCCAACTGGGAGTGTGTGGGTGTGTGTGGACCTACCCAGGACATGAGAGAGGCTCaTTTtATtgATGAGGAGTTGGGGTAGGAgTGGTGTGTaTGTGTGTGAATGTGGaAGTAATAGaTACCCAGGACATGAGAGAGGCTaGTTTCATCCAGTGAGGAGTCCTGGGGCAGGGGAGGTGTGTGAAAGTGTGTGAAAGAGACGGTCTCAGGAGAGGCCAATGCGGGGAGTGATGTGtGGAGGCACAGATCCCTTAGCATGGGCTGTGTGCTCTGAGGTGAGTGTGGGGGAAATCAGACCTAGGACATTGCaTATGGCTGATAGGACCAGCTCCATGGCTGCAGCAGGCTGTGAGAGGGGAAGGCATGTTCCTGGCTAAGCAaTGTCCAAAACTCCCATAATAGGACCCGGTCTGGTGGACCTGAGAGTaAAAGTaAaAGTGAAAGTGCACCGCAAGGGAGGAAATGAGAGGAAAAGCATTGAAACCAACTCCTTTGGAGTGCATGATAAAGAATTTTAAAAAAGGATTTAGAGGTGATTATGGGATGAAACTGGaTGCTCAAAAGTTAAGGACATACTGTGAGATAGATTGGCCTGCTTTCAATGTGGGGTGGCCCTCTGAAGGTACAATAGACAGGGAATTAATTGGCCATGTGTTTAAGGTGGTCACTGGAGTTGaAGGACAACCAGGATACCCAGACCAGTTTCCCTATATAGACTCTTGGCTCAATGTGGCACAAACTCACCCCAAGTGGCTACAGCCCTGCCTAGAGGGATATTGCAAGGCATTAGTGGCTCAGGCAGCCCAACCAAAGGAAGCAGAGGAACCTAAAGCCCCTAGaGTCTCCCAGGAAAAGGAATCCTCAAAGCCTCAGCTGAAACCAGTTCTTCAGGCTCCACCTGAGGAAAGGGAATGTCCACCCCCATATGTGCCAGTCTACCCATCTTTGGCCAGAATAAGGCAGGAGGCAGAGTCAGGAGCATCCACAGAGTCGGGCTCAGAGGAAAGTGAGGCTCAGTCTCCCCaCACCCAGAGGAACAGAAGCCCCTaTTAGAaAAAAACAAGGAAGATGGACAGGGCAAGGCAGCTGGGTGCCTCCACTCAGGCTGACCACGGGCTTTGCAGATGCCACTTCGAGAGACCAGGACACAAGTTTATGATGACCAGGGGCAGATACAAGGTGGCCCTAGGCTTTATGTTTATCAGCCTTTCTCCACTACTGATCTCTTAAATTGGAAACAGCATAaCCCCTCCTATACAGAAAAGCCTCAGGCTCTCATTGATTTGGTGAATTCTATTATTATAACACATAACCTGACCTGGCCGGATTGTCAACAACTTTTGCTAACTTTATTTAATACAGAGGAGCGTAGGAGAGTTAATCAGGCAGCTCTCAGCTGGTTAGAAGGGGAAGCCCCAGAGGCCACCCCTAACCCATGCCAGTTCaCCaTGGAGCGATACCCAAATGAGGACCCTAACTGGGACCCAAATGAGGCAAGGGACATGGAACGGCTGCAGCTATATAGAAAGGCACTCCTGAATGGGATAAAAGCAGGAGGAAGGAAGGCAATGAATATaAGTAAAATATCAGAAGTGCGCCAAAAGCCTGATGAAAGCCCAAGTGCATTCTATGAAAGGCTTTGTGAGGCATATAGGCTGTACACTCCAATTATTCCAGAGGCTCCTGAAAACCAAAATATGATAAATATGACCTTTGTCAGGCAAGCTCAGGGAGACATAAGACAAAAGCTTCAGAAGCTGGAAGGCTTTGCAGGGAAAAATATTAGTAAACTCCTGGAAATAGCAAACAAAGTATAaATAAACTGGGAAGAAGAGGCAGAGAGAAAGGAAGAAAGAAAAACaAGAAATAGAAACAAAGAGACAGtTCAATTTatAGCTGCTGCACTAGCAGAAAGTAACCCTGGATTTGCTAGAGGGCGTGGCCAAGGCAGAGGCCAAGGAAGAGGGCAGACAAGACCGGGAGAGGAAAGCCAGTCCCaGTTGGACAGGAACCAATGTGCAAGGTGCAGGCAAATaGGCCACTGGAAAGATGAGTGCCCCGAAAAGGAAAAGGATGAAGATGATGGTCAATGGTCTAACACCCGAGTGCGGCGTTCGGTTGCTAGTAATGGTGCTTCAAAGGCAGATCCTGATCTGATCGGCTTAGCAGGGGCCAAGAATTTAGAGGACTaAGAAGACCGGGCTCCATCCTTTTAGGCCCTGGGGAGCCTATGGTCTCTATGGAAGTAGGGGGCCGATTAATGAATTTTTTGGTCAATACTGGTGCTGATTTCTCTGTGGTAACTCACCCAATTAGCCCCCCCCCAAAGAACTGTGCTACTATCGTAGGGGCTACTGGGGCCAAAGAAAAGAGACCTTTTTGCAAATCCAGGAGATGTGTTATTGGGGGACAAGAAGTGCAGCATGAGTTTCTATATATGCCAAATTGTCCAGTGCCCTTGTTGGGGAGAGACTTACTCCAGAAACTGCAGGCACAAATTTCCTTTACACCTAAAGGGAATATGACCCTGGAGaTAGGGAAGCCAAAGGCAATGGTATTGACTCTAACTGTCCCAAAAGCTGAGGAATGGCGGCTCTATGAACTGTGTACCAGGAGGCTGCCGGAGCTGGACCTACACAATATGTAGGGAATGCTTTTCAAGGTACCAGGTGTATGGGCTGAGGACAACCCCCCTGGACTTGCTGTAAACAGACCCCCAGTGGTAGTAGAGCTTAACCCTCATGCTGCCCTGGTATGAGTCCGTCAATACCCACTACCCAGAGAGGCAATTGAAGGCATAACAAAACATtTAAATCAGCTCTATGAACAAGaGATTATAGTAAAATGCAAGTCCTCTTGGAATACTCCTCTGCTGCCTGTGCaCAAGCCAAATGGTGAATACAGGCCAGTGCAGGACCTCCAGGGGGTAAACAAGGCCACTGTCACTATCCATGCCATAGTACCCAACCCATACACAATGTTGGGACAGATTCCTGCTGAGGCCGCGTGGTTCACGTGTCTGGACTTAAAGGATGCTTTCTTTGCTTGAGGCTTGCTCCCCAAAGTCAGCCTATATTTGCCTTCCAGTGGGGGCAATCGCAGTATACCTGGACAAGGCTGCCACAAGGGTTTAAGAATTCTCCCACTATCTTCGAGGAGGCTTTGGCTACCGACCTTGAGGCTTTTGCGCCATCTAGTGACAATTCTGTGCTATTACAATACATTGATGATTTGCTATTCGCTGCCCCCAaGAGGGAGGAATGCCTCCAAGGAACAGAGAGGCTTCTTCACCTGCTGtGTGAAGCTGGTTACAaAGTGTCCAAGGACAAGGCAAAAGTCTGTTTTCGGGAGGTTGaATATCTAGGATTCATGGTATCCCAAGGCCAGTGCAGGCTTGGAAGTGCATGCAAGGAGGCTGTATGTGCATTGCCCACCtCAGTTACAAGGCAGCAGGTCAGGGAATTTCTAGGTGCAGTGGGATTtTGCCGAATCTGGATTCCAAACTTCTCCCTTATAGCAAGGCCCTTATATGAGGCTACCAAAGGAAAGGAAAGAGAGCCCCTCCTATGGGAAAAGGAACAAGAAAAGGCCTTCAAGGATATAAAGGAAGCTCTCATCCAGGCCCCAGCACTAGGGTTGCCAGATGTAAAAAAAAtCCTTCTTTTTGTAtGTGGATGAACGAAAGGGAATGGCAGTCGGAGTCTTAACTCAGTTGTTGGGCTCTTGGCATCAGCTGGTAGCATACTTATCCAAaAGACTGGACTTGGTGGCCTTAGGTTGGCCCCACTGCCTCAGGGCATTGGCAGCTACTGCAATCCTTATAGAAGATGCCAACAAGCTAGCCCTAGGTCAGAAGTTAATAaTTaGGGTGCCACACGCTGTAGTCACCTTAATGGAGCAAAGAGGACATCGTTGaCTGTCCAACTCTAGAATGCTAAAGTATCAAGGGCTTCTGTGTGAgAATCTCCAGATAACACTACAGACTGTAAATACCTTGAACCCAGCTACCTGCTGCCTGTGGAGGAACCTGATTGGAAGaATGGTGGGTTGCCTCACTGCTGGCAGGACCTTCCCCACTGTTGCATAAATACGGTGGACAAAGTGTTCTaGAGCTGGGAAGATCTCAGAGATACCCCCTTGGAGAGCCCAGATGTTGAATACTTCACTGATGGTAGCAGTTTCATAACAGATGGGGTGTGATATGCAGGGTATGCAGTAGTGACCCAACACTCGGTGGTTGAGGCTCAAGCCTTACCTTCTGGGACTTCTGCTCAGAAGGCTGAATTAATAGCATTAACCAGAGCACTGTTATTGGCCAAGAGAAAAAAGTAAACATATATACTGATCAAGATATGCTTTTGCAACCCTGCATGCCCATGGGGCAATATACAAAGAGAGAGGACTTTTGACTACTGAAGGAAAAGAaATAaAAAATAAAGAAGAAATTTtGCAATTATTAGAAGCCATATGGGCTCCAGAGAAaGTGGCTGTCATTCATTGCAAAGGACACCAAATTGGGAAAAGCTATGAGGCACAGGGCAACAGAAAGGCAGACCGAGAGGCTaGGCGGGCAGCAATGAGCAAGGTTTTACCTGAAGAAAaAACTCTAGCAATGCCTCTCCTTATAGAGCCCCCTTTGCTaGAGGTACCCAATTACTCTTCAATTGAAAAAGCTTGGTTTGGTCAGGAAACAGGAAAATATATTAAAGGTGGATGGTGGCTGTTCTCTGACAGGAGGCTAGCCATCCCAGAGACAATAGCCCCAAGGTTTGTGAAGCAGATCCATCAAGGAACACACATTGGAAGGACAGCCCTAGAGACTTTGATAGGTCAGCATTTCTATGTGCCACGGCTCTCTGCCATCACCCGTGCTGTTTGTGAACAATGTCTATCCTGTGaACAGAATAATCCAAAACAAGGACCTACTCGACCtCCAGGAATTCAGGAAATGGGAGCTGTGCCTTGTGAGAACCTGCTTGTAGACTTTACTGAGTTACCTCaAGCAGGAGGTTACTGGTATATGCTAGTGTTTGTTTaCACCTTCTCGGGGTGGGTTGAGGCCTTCCCCACCAGGACTGAAAAGGCAaGAGAGGTGACAAAGGTGCTACTAAAAGACATCATATCAAGATTTGGGTTGCCTTTAACCCTAGGATCAGACAATGGTCCTGCATTTGTGGCAGAAGTAGTACAACAGCTGACTCAACTTTTAAAGATCAAATGGAAACTGCACACAGCCTACTGACCACAGAGTTCAGGGAAGGTAGAACGGATGAACTGGACACTCAAACAGCTACTAAAAAAGTTTTGCCAGGAAACTCATTACGATGGGATCAGGTCTTGCCCATGGTCCTCCTCCAGGTCAGGTGTACACCTACAAAACAAACTGGGTATTTGCCCTATGAAATATTGTTCGGAAGGCCACCCCCAATCATTAATCAAATTAGAGGGaATTTAAAGGAGTTAGGAGAGTTAACCCTTAGAAGACAGATGCAGGCTTTAGGAGTGGCAATGCAGGAGGTGCAAAGCTGGGTAAGAGAAAGGATACCTATAAGTCTAACAGACCaAGTGCATCCACATAAGCCGGGGGACTCTGTTGGGTTAAAAGGTGGAATCCAACAACCCTGGGGCCCTTATGGGATGGGCCCCATATTGTAATCATGTCTACTCCCACTGCTtTTAAAGTTGCAGGTGTCACACCTTGGATTCACCATAGCCGGCTGAAACCAGTGGCAGCAGTGACTCCtGATGATGACCAaTGGATTAGCCAACAAGACCCAGATCACCCCACCTGAATGGTCCTACGGCGAAACCCAACCACCAGTAAGAAGGACAACTGCCCTGCTCTGACCACACTGGAGaCTGGTCAGTCTACaCATGGCTGAAGCTTGAGGATCCTGCAAGCTCTGCTCTAGTCACATCCCAGAAGCTGACTAGTCaATGCACAGCTGAAGCTAAGAGGACCATCTCaGGATAAGTAAATGTGGATACAATTTATAACCCTAGTTATAATTCTGTTAATACTGATTGTTCTGTTGTTATGTTATTACTGCAAATGTGaAATGTCTATGCCCAGAGGAAGGTTTGCCATGCaCATGTGTAGTGTAAGCATGTTTCTATTACATACACTGATGTTGTTACCATTTCTGCCTATACTAAAAGGGGAGAAATCTCTAGAAGGATGCCCACACTGTGTACATACTACCTGGGTAAaGAATACCATAGTTAAAACTCTACTGTACCATACCTACTATGAATGTACAGGAACCAAGTTAGGAACCTGCACATACAACCAGACCACCTATTCAGTCTGTGACCCAGGAAATAATCAGCTATATGTATGTTATGACCCTAAGCTCTTACCtTATGAATTCTGGTTTGAGGTACATATTAAATCAGAGaGAGAAAAAGAAAAAGAGCTTATAaCTTGAACCAAAGAAGCCCCTCCCTCCTATAAAGGGCCTATTTCCTTGTACTTTGATGCCTGCCATGCTGCATATGTTCATAATCCTAAAAAAACAGAAGCAGTCTGCAATGGTTTAACACAAGAGAGGCTTAGCAGaAGCAGCCCTAAACATCTGTATGGAGAACCACAAATCAGATGCCCAGACTGTAACATTCCaTGGTCTATGCTAACACAGCTCCAACACTTATATTCAGGAAGGACTGCTCTGCTAAGTAGTATGTCAACCAAACCAAATTGTAAGACAAGGACATGCAATCCTTTAAATTTTACTaTTtTAAAGCCAGAGCTACCTTTTTGGTCTACAGGACAGACAGCACTATTATGAGTTaATAGACAAGGAGCAGGCCTTGGAGTTCCACTACTAATTGTCAAAAAGACTAGAAGGACTCAAATGCATCCAACCCTGCAATTCCGGGTCCATAAGTCATTCTATAAGCATTTTaATAGaAGTGaAGTTTCCTATAAtaAAAaATTATTTaCTCAACTAaTaAAAAATAGTGGAGCTTAGtAATTTCCTCATGCTAtGTaTGTGaAaaAACTAATATGGGGGACtAGTGGCaATGGGAGGCAAAGaAATTAATGCCACAAGATAACTTCACTTTGCCTAACCCTGCCAGTGAACCAACAGCCTCAGCCAGTGTTTGGTTGTTAAAAACCTCCATAATTGGAAAGTACTGTATCGCCTGATGGGGAAAGGCTTTCACAGAGGCAGTAGGAAAAACAACCTGCCTAGGGCAACAGTATTATGATGAGACTAAAAACAAAACTTATGGAGAAACaCCCAGAAtGACTCCTACTTACCAGATCCAAATCCTTTCTCTTGATTCTCTACCCTAAGCCACTCTTGGCATCAGCTAaAGGCTCCAAATGCTTGGAAAGCACCCTCTGGCCTATATTGGATCTGTGGAGCATGGGCATATCGGCAACTGCTGGCTAAATGGACAGGGGCATGTGTGTTAGAAACAATCAAGCCATCCTTCTTTTTAATTCCTCTAAAGCAAGGGGAACTCTTAGGGTATCCAGTTTATGATAAAAATAAAAGAAaAACTAGAAAAAGCATAATCACAAAAATAGACACAAATGTCAAAAAaGATGTAGACATAGGAGACTGGAAAGATAATGAATGGCCTaGAAAGAATCACTAAATATTATGGGCCAGCTACCTGGGCGCAAGATGGGTCATGGGGGTACCACACCCCAATCTATATGCTCAACCGCATCATAAGGTTGCAGGCAGTCCTAGAAATTATAACCAATGAAATGTCAAGGGCACTAGATTTATTGACAATACAAGCAACACAAATGAGAAATGCTATATATCAaAATAGATTGGCTTTAGATTACCTCTTAGCCTCAGAAGGAGGAGTATGTGGAAAATTTAATTTAACCAACTGTTGCCTAGAAATTGATGATAATGGCCAAGCTGTCATGGAAATCACAGCTAaAATGCaCAAGTTGGCCCATGTTCCAGTTCAGACTTGGTCTGGATGGTCCCTGGATTCCTTGTTTGGAGGATGGTTCTCAACtTTTGGAGGATTCAAAACCCTCATTGGTGGGTTTTTGTTTATTCTTGGCATCTGCCTCATCCTCCCTTGCCTTTTACCCCTGTTTATTAGGAGTATTCAGTCAACTATAGAGGCAGTAGTAACCCGAaACACTACCaTGCaGTTGATGGCATTAACCAAATATCAGCCACTGCCAGTAAAAGAAGCAGCTCAGCTCCGtGAAAAGGTGGCAAATAGTGGTGCTTTCTATTAACACCTTTGTTATAAAAAGCACCAAAtGtGGGAAATGGAAGAGGAATTAAAAGAAATTAAAGaATGTGTAAGCAAAAACTCAGTTGTATGTAAGAAAACCCAaTTCCCCCTGAGGAAGAGAAAGAGCTGGAGTCCTTTAAAAATTAACTGCCTGTTTTTCTGTGGCTAGTGAGCCTTATCTCTCCCTTTCCCAGGCATTGTGAAGACCCTGTTTCTCTAGCTGTGCAGCTGCAAGGTCACTAGACAGATAAACTCAAGTCGTAAAACATGTTTTTTCTTGAAAAGTAAGAAATGATGTAATGCATGTCTCAATTGAATAATGTTTTGTTTTTTTTGTAATATGTTaTGAAGATTtaAAAAAATGtTAAAAGGTAaTTGATTGTTAgaGTAGTTtTTTGaaTGTTAATTGATGGGtaGTaaTAAATAATaAAATAATTTTAATattTTTTGATTTaAATTATtaaaA

>herv4_10250_100014_con_gagputein

MgGKalKlTPLECMIKNFKKGFRGDYGMKLVAQKLRTYCEIDWPAFNVGWPSEGTIDRELIGHVFKVVTGVgGQPGYPDQFPYIDSWLNVaQpHPKWLQPCLEGYCKgLVAwAaQPKEAEEPKaPlVSpEKESlKPqLKPVLRaPPEEREClPPYVPVYPSLARIRQEAESGASgESgsEESEAQSPlPREtEAPviKtQKmgtgqgaggaplgapqgLQMPLREtRTQVYDmQGQIQGGPRLYVYQPFSTTDLLNWKQHaPlYTEKPQALIDLVNSIIITHNLTWPDCQlLLLsLFNTEEcRRVNQAALSWLEGEAPEATPNPCQFavERYPNEDPNWDPNEARDMEwLqLYRKALLNGIKAGGRKAlNiSKISEVRQKPDESPSAFYERLCEAYRLYTPIIPEAPENQiMINMTFVRQAQGDIRQKLQKLEGFAGKNISKLLEIANKVYvNWEEEAERKEERKTRNRNKdTaQFvAaaLaEStPGFARGegQGRGQGRGQTRPGEESQSpLDRNQCaRCRQIGHWKDECPdKaKDgDDGQwllvmVlc

>herv4_10250_100014_con_proputein

VASsGaSKADPDLIGLAGAeNlEDsdtagsllLGlGEPMVSMEVGGllMDFLVNTGADFSVVTHPISPPTKNCATIVGATGAKEKRPFcKSRRCVIGGQEVQHEFLYMPNCPVPLLGRDLLQKLQAQISFtlmGi

>herv4_10250_100014_con_polputein

TlEvGKpKAMVLiLavliAEEWqLsELCaRkllELDLHsmwGMLFKVPGVWAEDNPPGLAaNRPPVVVELNPHAALVZVRQYPLPREAIEGITKHLNQLYEQgIIVKCKSSWNTPLLPVlKPNGEYRPVQDLQgVNKATVTIHAIVPNPYTMLGqIPAEAAWFTCLDLKDAFFCLRLaPQSQPIFAFQWGQSQYTWTRLPQGFKNSPvIFidALATNLEAIAPFSDNSVvLQYIDDLLFAAPmREECLqGTERLLHLLgEAGYiVSKDKAKVCFlEVgYLGFMVSQGQCRLGSACKEAvCALPTvVTRQQVREFLGAVGFCRIWIPNFSLIARPLfEATeGKEREPLLWEKEQEKAFKDIKEALIQAPaLGLPDVKKpFFLYVDEqKGMaVGVLTQLLGSWHQLVAYLSKRLDLVALGWpHCLRALAATAILIEDANKLALGQKLIvgVPHAVVTLMEQRGHRwLSNSRMLKYQGLLCdNlwvTldvNTLNPATLLPVEEPDlKnGGLPHCWQDLPHCCINmVDqVFlSWEDLRDTPLESPDVEYFTDGSSFITDGViYAGYAVVTQHSVVEAQALPSGTSAQKAELIALTRALLLAKgKKvNIYTDSRYAFATLHAHGAIYKdRGLLTTEGKdIKNKEEIlQLLEaIWAPEKVAVIHCKGHQlRKSYEAQGNRKADREAgwAAMSKVLPEEiTlAMPLlvEPPLLEVPNYSSVnKaWFGQKTgKYIKGGWWLFSDRRLAIPETIAPRFVKQIHQGTHIGRTALETLIGQHFYVPwLSAITRAVCEQCLSCaQNNPKQGPTRPPgIQEMGAVPCENLLVDFTELPlAGGYWYMLVFVtTFSGWVEAFPTRTEKAqEVTKVLLKDIIlRFGLPLTLGSDNGPAFVAEVVQQLTQLLKIKWKLHTaYZPqSSgKVdwMNaTLKQLlKNvLpggSgWDHVLPMVLLQVRCTPTKQTGYLlYEILFGRPPPIINQIRGnLKELGELTLRRQMQALGVAMQEVQSWgRERIPISLTDvVHPHKPGDSVWVKRgNpTTLGPLWDGPHIvIMSTPTAvKVagVTPwIHHSqlKPVAAVTPnnDqwISQQDpDHPa

>herv4_10250_100014_con_envputein

MFLlHTLMLLPFLPILeGEKSLdGCpHCVHTTwVtNiIVKTLLcHTYYECTgTKLGTCTYNQTTYSVCDPGNNQLYvCYDPeLLPYEFWFEVHIKSEgeKeglllaptKpaPPigPISLYFnaCHAAYVpilKKTEgVssGLTQeRLSSSPKHlYgepQIRCpDCNIpWSmliQLQHLYSGRTALLsSMSNKansKTRTcNPLNFTvLKPELpFwpTGQTALLfVHRlgAGLGvPLLIVKKTRWTQMHPTlQFwaHKSFYKHFnQsVtlPPSiKnLFaQLaeNIAGSLgISSCcVCggTNMGDqllWEAKeLMPQDNFTLPNPASEPTASASVWLLKTSIIGKYCIAcWGKAFTEAVGKTTCLGQQYYDETKNKTLRRNaQtDSYLPDPNPFSZFSTLSHSWHQLqAPNAWKAPSGLYWICGAWAYRQLLAKWTGACVLETIKPSlFLIPLKQGELLGYPVYDKNKgRTRKSIITKIDTNVKKDVDIGtWKiNnglpwpITKYYGPATwAQDGSWGYHTPIYMLNRIIRLQAVLEIITNEMSRALDLLaIQATQMRNAIYQNRLALDYLLASEGGVCGKFNLTNCCLEIDDNGQAVMEITAtMsKLAHVPVQTWSGWSLDSLFGGWFSTFGGFKTLIGGFLFILGICLILPCLLPLFIRSIQSTIEAVVTRyTTvQLMALTKYQPLPvKEaAQLcEKVANSGAFY

**HERVIP**

>herv4_10410_100015_con_chaindnarm

tggcatctaaatgacaaacaagatttcatttttagtaaaccagaaaaaacactgtacatcttttattgtaaaactgcaaagacaattttttaaaaaaacaaaattctacttgatttgtaaaaatttccttattttttaaatgtctgaattattaaatactgttgttcaatttattaacatatttctttaacatttaatgttctatgactatttaaaaatacatttcaggaaaattttaaattctatatgtagacattatttataatacctcaaaactggaaaggagttaaatgtccacagcagaaaatggttaaataagctgcagtatataaaaagggcagtttaagtaaataaaataaaaattaaaaggcagtttacaagatggtatttataattccgtaaaacaaaaaaaggctggaagtttattttaaaaactatgtgaaaaaatagtaatgatgatcgataatgatattatagttgatttttattttctcctttatacaatttggtattttccgccttattgcaatgagtatctattacccttaaaaatcaggaaaaaataagaaaactttaatcttgtaaaaacaaatggatataaatccatcctaagatgaccatgtaagtactaaatgaattactctttaaaaaaaatagactaaaacagatatttaaaatccaacataacataagattaagtaagttaagttttgagttcctcattaaagaataaatatttcactatgttcagcttttttgttttttgttctccgttttaaagtttaacttcctctttctttatgcttccttgttcttagtttcataaaacaacctcctcctagcctctacctcctttttaattcttatttttctttcctaacagtattcaatttcttggtctgtcctcatccttaacgcacacatccctccttatttcctccttcttttcccttatttcccctcataaacccacatcttatcttcctttcccatcctactcactcacccccccccttttcCttatttggcaaagcTtTtaacaatAGcCtacaaagtcaggAATcgGgaTtAGcTTAGAtTGTGccatgcccctttgGTCcaACCCcctAGCCAATaGGGgAAgGACACAGaAGcAGGgActagcTGcgTagtaaaattgTAGGgATAAaAACcccCTTcccCTCCtTTGTTcagGTGTGggatCTCTtGCcaTtGtcacagctGcaAGcatGCACCCTTCTgcAGAAGTAAATtttGCCTTGCTGAGaaatgaaaataaAactTTtTgtccagtgctgatttgttgAGTGCTggTTtTtcTTtGtggCACtagggtgaGAgcAtTTaTTTCcAACAAaTttTGGGGGCtCaTCcgGGAtTCCCATTCTCCTCTGGGGAaGGGgtCTCcggTCAtCgtTCTCaTGAGGAGAcgCaTCCCaCTGCCTCgTTGcgGTGGCCTCAgaGGGTgAGGgATCaaGACCCAcCCtttccttaGTGTGAtGAATAAACCCGGaCTCTCAGCAAtgtgGgaagggctaagcattatggttgtgtggagtgaaaaaaAAcAGgCctgCAacttaaggggaaaggatcctcacATAagcaCcgcgGcGACatacCAGGTAAccTcTGTGCACAGACCAAGGTgAAGAAAagaatcgtcaCaGGgGctaggcGACAAAGTATTTCCTTGGTGGTcGGGgAtATtcTGGAGGTTGAAAGgTGTGTGtacacctcagaatgatcacaagcactactgcttgaagtgctgcttgtgtagatggtAtTaaGtgctaatgctgtacggaGTgAGtgggcCctATctgtGGttccatggtcacctcatatggcttaggacagagccattagaatccttgaataggGgtTtatattgAcCcaccagcaatgCTAAgAgGgAcCtgaaatatTCccgcaagGGaAgtggCcagagaaggacaaagcaaaaggatgaaattccttctgatagcccctaagaggggtgtaagaatcctcaaaaaggtGcaagaAaacTccAgtaggaaggaatctcgaatatgagaaaatagagccataataaatggcaagaaaaaggcaagcaAgagagctccaacaggtggccaacttaGGgaagaaAgggcaAGaAAgctctaataaaagggattggaacgtgttaataataagaaccctgtgtcccaggaagaaaaaactctaacatgagggattgAgcCtaAcaAagcccCAagAtgGGaAAtacCcCcaAGcAAGAcAGggAgtAaaAAaGAtaAaGatgGtAAtAAAgAtagcttgCCCcctgATAGtCCCCTaGGtCTCATGtTAAAATAcTGGAAgGATAATGAaAGgACTAcctAACATAAggAAAAAgaataCAACAAATGATAAAATATtttTgcTGTTTtaATTTGGACtcAaGaaCCcATCCTCAAACCCTCAatTCTTCTGGCCAaAaGTTTGGgTCgAATGAGGATttaATgTGTCAACTtcTAATtcAATATGTtAgcATGAtAAAAgTCCaGtttTCtCAAGAaGAAaTAGAcTATGCtCTtTGTTtGGAGgCAgGGACCTGTCCTCCTttttCCCtTAAAgacaAcccagctgcattttatgaaatAgGGAaaAAcCcaAtctagcAcctCacaatGAaaAgTcActcGatcagccagctcccAtacCtaAagactctaaCCAacaCacaATGGGATCCcCTgAgACCcAtCTTCCCCcatgCtcAgTacCcCcctcactaacaacCcttCcCCtCagGCAGCtgCtgccacCcCAGatCctgccgcagatccctcctcccattCcAgATCCtctttCccCTaCtCAcgtTaTtCCTCCTCcCTTAtAAcCCTGACTCTTGGGAaTtAtcaTCCCAtgAgCCTGcTCcacCtccCAgCCtAacaAgTACcctTCCCTAAAAGGACgggcTcCAgCGTGAGATAGAacCAATGTAAAAAgGATATTCAaAACTTCCCtTTtCCCTtccCtACatCTAAgGAGTCAGCcCCAACTCTCTTCCCCTTAAgAGAGGTgCCaCaAGGAgggggaGGgGGagtgggccATTGGCTTTGTAAATGCTCCCctcTTAACCAGTTCAaGAAGTCcgAAATTTaAAAaaaaAAGGAaCTtAAGCCAtcgagtcacTaTTAGATGACCCTTATGGAGTgGCAGATCAAATTggtGAtCAATTttTAGGACCtCAGTTATAcatggcttatataagttatatacttatatACTTGGGCtGAGTTAATGTCCATCCTAGGCATCCTCTgcgtcctctTTTCAGGGGAAGAAAGAAGCATGATCtgatcCgtAGGGCTGCTATGGtAGTTTGGGAACGTGAACACCctcctCTCCtGGTCAAAACGcaTTCCTgCaGCgGAcCAaAAAtacTTCCCcGCCCAAGcaagACCCcCagTGGGACAATAACAAtGCAGCTCACcgagGAAAAtATGCAAGAcCTaAGGGAaATGATAATAAAAGGAATTcGGGAATCAGTACCCCgAACcCAAAATCTTTCTAAAGCATTTGATAttgtttTACAACAacgGaaAAAGATGAAGGggCCTATGaAATTCtTAGACAgGACTgAAGGAgCAAATGAGAAAATATGCAGGCCTAgATTTAGAAGAtCCCCTTGGaCAAGGAATGTTAAAGCTcCAttttgtcactaaaagttgtccagataTTTGTtACTAAaAGTTGGCCAGAcATTtCAAAAAAaaagTTACAAAAaatTAGAAAATTGGaAAgACcataccgtCCcaTAAgtGAaCTTCaatgagaagtgaacctcaagtgaagtgacctcaagtgaaccTcAGaGAaGCTCAAAAAGTaTaTGTgAggAGaGAtGAaGAAAAgCAaAAAcAAAAaaccAAAActtAtgtTatccACCTTCCAACAaacaGatGGCtCCAAAcCcAtatacttctaaAcAgaGCTtcCaGgtgggCCAGaAACTatAAAgGGtCcAAAcaaaccttctttatgcatatagAGgaCCcAAgcCtcCaaaatctagAGGaccaAGGcCCTCattTaagcttctaaacaaagcttccagggggccatgaaaaatgagaagaCcAggCCctatgttatgcttctaaacaaagcttccagggggccagaaacTAAAgaatgtccagaccctcgagtATgtttctaaacaaagcttccaggGgcagaaactataaatagcAAagtgaatttatgctaaaacAAAgAAtCCcatgagAActgtgagaggGaGgAAGGACcagaccctcgtttatgcttctaaacaaagcttccaggAAgATAgaTGcTaCAAATGgtccgtccagaccgtTGGAAgAaCAGGCCACTTCAAGAGaGAATGTCCcgAATTAgAAAagGAaAAAGAAGcCcTTCCACTCATGACtTTTGAGGAAGAATAGtagGGGgGTCAGGGGCTCTGTCTCTTTTtATCTTGAGTCCCACCcaccAgGAGCCCTTGATAAATTTAgGAgGTGGGACCtAAACATGAgcTTATcACCTTTTTAaGTcgATTCAGGaGCtGCTcGCTCCTCTGTTTGTTtCCCtcccCCAtCaTAATaTtgCCTGCTCtTCAGAAGAACTTTTaGTCTCtGGGGTAAAAGGAGAAGGATTTAAAGCAAAAATtTTcAGAAAAcACAGAAGTtAgATAccAAgATcgaTCAGCTaattcATATTCAaTTTTTGTTAATcCCTGAAGCAGGAACTAATtTATTAGGAAGgGAtTTAAaTGTTAAAaTTAGGCATAGGcCTaCAAgTcaGcCCAAaAGGATTCCTCACcTCATctcccTAAACCTACTCctACCACTGCAGATGAAAAATAcATTcATCCTgATGTcTGGTCaAaaGAAGGAAAccGAGGgAAACTcCAAGTcCCTCCAATcCAtATCAAGcTAAAAAcCCcgcCtGGGGAAgTAGTAAGGAGAAAgCAATACCCtAtTTCCCCTAGAaGGCAGGATAGGgTTAAAAcCCTATAATTGAAgGtCTtATTAAaGATGGGCTTCcctattcccttggaagccaggataggtttaaaaccaggTTGAaCCCTGTATGTCcCCTTATAACACCCCAATcctgtatgtccccttataacACTGCaatattgcCtGTcAAGAAATCAGATGGGTCATACCGgCTtAGTaCAAGACCTtAGAGCTATTAACCAAATAGTCCAGACtACcCACCCtcgTTGTCCCcAAtCCTTACACCATTtCTCAGcAAAATTCCATATAAtCATCAATGggGTTTACAGTAATAGATTTAAAgGATGCcTTTTGGGCATGtCCCTTGGCTGAGGAtAGCcGAGAcATATTTGCTTTTGAGTGGGAaGAtCCCCATTCaGGGgtGGAAACAACAATATCGATGGACAGTCTTgCCCCAAGGGTTCACAGAcTCCCCtAACCTtTtTTGGTCAAATTtTAGAaACAAGTacTAGAAAAAGTTtccaTCCCcAAAACAaATATGCCTGCTCCAgTAcGTGGATGAtaTtCTtATATCTGGTaGAaGaTATAGAgAAaGTAgCTGaCTTCTCtACACATATTCTtAACCATCTGCAaTttGAGGGGtTAtGaGTCTCAAAAgGaAAaGCTTCAgTATGTAGAaCCTGAAGTTAAATATTTgAGGcCACTTAtATAAGTgCAGGcgcAAgcGAAGAATAGGGCCTGAAcGAGTTGAaGGAATcGTgTCCtTACCCTTGCCTCAAACTtAAACAAGAACTCAGgAAATTttattacaggattcaggaaattTTTAGGgTTAGTTGGATAcTGCCgCTTATGGATTGACTCATATGCaCTAaAcAgTAAACTtcTATATCAAAAACTTaCCCAGGagAAGCCTGACCacctaaccTCTCCTgTGGcACTTCTGAgGAAgTCcAtCAGGTTGAaGAGcTaAAAgAaataGCTcATAACTGCCCCTGTTtTAGCCTTACCtTCCCTAGAAAAGCCATTtCACCTTTTTGTTAATGTAAAtAAtGGcGGTAGCTTTAGGAGTGCTtACCCAAGAACAcGGAGGCCACCgGCAGCCCatgGTgGCCTTCCTaTCcAAAAGTcTTAGACCCAGTaACcTGTGGATGGCCTcAATGcATcCAATCCATtGCaGCTACaGCatTatTAgttgAAGAAAGCAGaAAgtTAACCTTTGGaGGAAAaTTgAcaGTAAGCACaCCcCAcCAAGTTAGAACTATcTTAAAtCAAAAAGCAGGaAGGTGGCTtACTGACTCAAGAATtTTgaaatactgaaattgaatcttgaaaaattAAAagtaTATGAgGCTATtCTgTTAGAAAAAaaaaaGATGAtTTaAaaaaaaaaaaaaggatggtttaaCATTAACCACTGATAATTCACTtAACCCAGCAGGTTTCcTAACAGGGgATCatgCAAAtCTAAAaaaaaaaataaaacaaacaaacaacaacaacaacaaaacatccaagccacaaataAAGAcatcttGAgCACAcATGTTTAGATTTAATTGAtTACCATACAAAaGTcaGACCAGAccTAagGGAGAAACttaCCCCTTCAAAACaGGacGgCACTTATTTATAGATGGtTCCTCCCaGGTaATTGAAGGAAAAAGACACAATaGGGTATTCAGTAATTGATGGAGAAACTCTTGAtgtatAGAAATAGAGTCAGGAAGATTGCCtAATgacAATTGGTCTGCCCAAACaTGTGAatgaaCTGTTTgcccaGCACTcAaagCCAAGCCTTAAAaTACTTgCAaAACCAGGAAGGAACTATcTATACTGATTCaTAAGTATGCCTTTGGAGTaGCtCATACaTTTGGAAAAATTTGGACTatgtGAACGAGGTCTcATTAAgccTAGTAAAGGCCAAGAcacCTtGTcCACAAaGAacTAAaagTCACCCAAGTATTAAATAATCTtaCAGTTGCCAGAAGAAATAggctctgcaagacccatggGCTATTGTCCATGTCCCAGGcACACCaaattgaacaacaaAGAAAAgTCTTTCTTTTGAAAGTCGAGGAAATAACCTaGCAGATCAaATAGCCAAAataaacaaagCAaGCTGCCgTTTCTTCTgAAAaagCaCCTATTTTTCACTTAACTCCTTGCCTaTCCTCCTCCTACTGCAATCCCCATcTTCTCTcCCaCTGAAAAAGAAAAAtataaataaataaataaaaaagaaaaaagaaaaagagaagtTAATAAAAATAGGAGCTAAAGAagaAAcTCAGAAGGAAAaATGGaTATTACaCAGAcCAAAGaGAAATGTTaTCCAAACCCCTCATGAGGGAAATCTTGTCcCAaCTaCATCAAGGGACcCACTGGGGaCCCCAAGCTATGTGTGATGttCAGTTCtaTCAGAGTTTATGGGTGTATAGGAATTTATACCCTgGCcAAACAAGTTACAGATAGTTtGCtTAgTATGTAAaAAAAaaaaacaaaaaaaagaagaCTAATAAaCAaACtctaTAAAAAAAtcACCCCTtggactgGGGGAAGaAATCCAGGgaTTAAGgCCATTCCAAAGTgTCCAAATTGATTACACTGAAATaGCCCCCAATAGGTCatCTAAAATATTTAttTAGTaATAGTAGAtCACCTtacACTCACTGGGTAGAcAGCTATtCCCTTTTCAAaTGCAACaGCCAATAATGTAGTTAAgGCATTAATTGAAAATATaaTACCCAGGTTTGGACTAATgAGAAAATATTGAtTCAGACAATGGAACCCATaTTCACTGCACAtaTcATTaAAaaaAAGCTAgCCCAAGttAcTAGAcATaAaAaTGGGAATAcCATACcCCcTGGCAttaaataaccttcagttaccagaaaagaaaaaaatacCCAcCtTCATCAGGaAGAGTAGAAAGAATGAATCAgACTCTaaaaaatcaaccaggccaAAaAACCACcTAACCAAATTAGTctTAGAgACtcgagactcggTTgCCATGGACtAAATGccttCCcatTttttGCCTTgtTaAGAatccaAaCcaaactacccctcgaaaagatgttggcctaagacatacatacacacacaaaaacagatctcctccttcctccccgcctctggctgccggcaggacctttctctcgctgctactgggaccccgtgtcatagcccaggctgagcacgatgccccctcaaaagggaggtgatggaattaaaccacccccaatcattggaagatttggaacctcactgaaaattggtattgttggattgccaaatgttgggaaatctactttcttcaatgtattaaccaatagtcaggcttcagcagaaaacttcccattctgcactattgatcctaatgagagcagagtacctgtgccagatgaaaggtttgactttctttgccaatatcacaaaccagcaagcaaaattcctgcctttctaaatgtagtggatattgctggccttgtgaaaggagctcacaatgggcagggcctggggaatgcttttttatctcattttagtgcttgtgatggcatctttcatctaacacgtgcttttgaagatgatgatatcacacatgttgaagaaagtgtagatcctattcgagatatagaaataatacatgaagagcttcagcttaaagatgaggaatgactgggcccattatagataaactagaaaaggtggctgtgagaggaggagataaaaaactaaaacccaaatatgatataatgtgcaaagtaaaatcctgggttatagatcaaaagaacctgttcgcttctatcatgattggaatgacaaagagattgaagtgttgaataaacacttatttttgacttcaaaaccaatggtctacttggttaatctttctgaaaaagactacattagaaagaaaaacaaatggctgataaaaattaaagagtgggtggacaagtatgacccaggtgccttggtcattccttttagtggggccttggaactcaagttgcaagaattgagtgctgaggagagacagcagtatctggaagcgaacatgacacaaagtgctttgccaaagatcattaaggctgggtttgcagcactccaactaaaatactttttcactgcaggcccagatgaagtgcgtgcacggaccatcaggaaagggactaaggctcctcaggctgcaggaaagattcacacagattttgaaaagggattcattatggctgaagtaatgaaatatgaagattttaaagaggaaggttctgaaaatgcagtcaaggctgctggaaagtacagacaacaaggcagaaattatattgttgaagatggagatattatcttcttcaaatttaacacaccttaacaactgaagaagaaataaaatttagttactgctcagataaacatacaacttccaaaaggcatctgattttttaaaaattaaaatttctgaaaaccaatgggacaaataaagttggggagatgggaatctttgacaaacaaattatttttgttttaaaattaaaatactgtgtaccctctccccccaatgaaatgcaagttcactaaatgtgaacacctttgcttttcatgtgattaagaccctactccaaattatagaagcttttcaagaaccatgttactctcatgatacttcattaatctccatcatgtatgccaagcctaacacatttgacagtgagaacaatgtggcttgctcctttttgaatctacagataatgcatgttttatagtactccagatgtctacactcaataaaacatttgacaaaaccaaataaaaaaaaaagaatccaaactgccCCtcggAaagAtattgGccttTCtCCTTATGAAATGCTcTATGGaTTGCCTTATTTACACTCCtACTGCTGACATTCCTACaTTTGtAAACAAAcatAGATCAgTTcCTcAAAAAtTATATAtaCTTGGTCTaTatCtTCtACTTTCTCTTCcCTTAagAcCtAAAGGcCTctTAGCACcccAggCaCCACCCCTgGAgTTcCCAGTaCATCAaCAtCAGCCtGGacagtGAtCACgTCCTcataCATCAAAAGcTGGAAAGAaGgaAAtgCTcGttAaCCagCCTGGGAAGGACCtTAcCTAtatccaGTGCTCCTtatctaatactcctAACtACTGAaACtGCAGaaaatcTCCgcACaGCaGAAAAAGGATGGACtCAtCACACCcGAGTCAAaAAAGCacctCCaCCccCTCCAGAagTCATGGgCcaTcaTcCCAGGGccAAcaCCCtACCAAAcTAAaGCTAAagaaAAgAGtTTAAttcTCtTttatctattctatTacTtTTTtTTcTTTCCTctttCtattGCTggtCAtCTgttGTTATtAATGgTAACTcAgGTCAAaCTCaCCTCAAACtagcATTACaTTTGATGCTTGTCTTGTTATcACCcTGTGGAGATcTgCAAAGTCAAAGgCAGCTcTCcaCTTCAGAAAAGTATCTCTGTCCcTcCTggatatcctcAgAttagcCAattatgAaTctgGAtCcTTaAatctTggcagttgtcaatgaagaccccagtgtcaactggtggggagtcttgccatggaacacttatacatAgcagttaTGccaTAgTTGGtacactAtgTTcTgTGGACcACcaAAgAcagcacaAGGcTGGaACTtCctCAAcaaGttaGTTGTtaAaatcCCTAAAACCaTACATtCAtTTTACTAAagagaacataccaCcCCCcCTAAtTGTCAgttaaACCAGTGtAAcCCAGtatcccctTACAgaTttCTATTactAtCCCcAaaagttcctcccCtTcactaaaCcataacCCTtCTTTAAGccGcTTgtttTATGGtATaGGAgGCtGAtgTtattCAGGaAcAGACCCTATAGGATccTTctcttTGAAatgtgcTtcatcaTtaatccCctaccgcCTaCacCttCCtCaccctCTtCcccgagaaatctatcctccccAAaactcTCTcccAAtcgaatcaAACctatTgTTCctcctccACCtccagAATGACAAAACCAAGGTAaGCtATTGTAGAAGTtAAAGAtTTtAAAaCAAAaaaaCTtTAGCAATTGAGACAGGATATCAAGATGCAAATGCCTGGtTGGAATGGATTAAATATTCCaTCCGCACTTaTAAggACAAAAGCaATTGTTAcGCTTGTGCgCAcGGCAaGGCCAGcAGGCCCAGATTGTCCCCTTTCCACTtGGATGGTCCTCCAGcCaACCAGGCATGgGtgtCTGTATGGTAGCTCTcTTCCAaGAtCCCACAGCCTGGGGTAAtAAaTCATGCCAAGtCTCTCTCTCTGCTATTcCCtGAAGTtCcctgaagtccAACACCCTGCGGGTCAGCCCCCGAGGGCCATCCAGCtTCCATCTCCcgAtGCCAATTTTtACcTCGTGTCTtctCTCACgACAAGGGGAAAACTTGGCATTCCTataTGGAGACCTAAAGGGATGCAGTGAGCTTAcAGCCtTTCtgaaaaagaactttCAAGAGCTTACCAATCAGTCTGCCCTTtaTtCATCCCCGAGCAtctgGATGTATGGgacctagtctatccctgagTGGTATTGTcGGTGGACCaTTACTGGACACTCTGCCAAGTaAACTGGAGTGGCAagtaCTTGTGCTCTAgTCCAATTGGCtATCtcCCTTTCACCCTGGCATTTCATCAACCAGAaAAAccagaggatcacaaaggAAAAACAaAACATCgTAAAACAAGAGAaGCCCctCTcATGGGTCtTTTGACTCTCAcGTTTATATAGATGCtATTGGAGTCCCAcGAGGAGTACCAGATaAATTTAAaGCCCgAAATtcCAAATAGtCTGCAGGAttcaaaTTTGAATCtATATTatTTtTGGTGGtTAACTacatatctaTaAATAAAAATGTAGATTGGATaagtagattgggtAAATTACATCTATTAcAATCAAtgatgCAatGATTTATTAATTACACTAGAGATGCTATTAAAGGAATAGCTagctGAACAATTAGGgCCtactACtAGCCAAATGGCCTGGGAAAATAGAATAGgCCCTAGACATGATATTAGCaGAAAAAaaGGTGGAGTTTGTGTCATGATTagAACCCAATGTTGTACcTTttTATCCctagggggCtAAtAAcACtGCCCCtGATGGAACcATAACaacAAAAGCcTTACAAGGtCTTACcgCttccTTATCCAATaaatGAATTAGCcAAAAATTCTGGAaTAAATGACCCCTTtactaatccttcACAAGaTTgATaGAAAAaTGGTTtGGTAAATGGAAAGGAgaTcATGgCCTCAATtCTTACTTCTCTTGCAATtGacTAATAGGTGTACTTATTCTTGTtGGaTGCTGTATCATACCaTGCATCCGTGGgcTAGTaCAAAGGCTTATAGAAACAGCtCTTACTcaccAAAACCTCCCTTAacTCTaccgctCCTCCACCTTATTCAGATAAGCTTTTcCTTTTttttttaAGAaGAtCcAAGtAGAACAACAAAGaaaaagCCAAGACATGTTagtAAAaAAGTTTGAAGAGGAAGAAcTATAAAAAcaaaaTaAAAaGGGGGAAATTGTgagaaAtAataaatttttgtaAaTTcCTCTtcaatgTCAAAtatctggtTtaagttgcttaattttatttgttaAtcTccTttaagtacTgttcTtTcttctgaaggacaattttttctTactactcccctccctacctttcttctcttttctcCttttttgattctttattcctatattacctgccccaatattcttcctccctatccataccagtttttatcacttcctagctcacaattccttcccgtccctccttttagaaactgatctcatgaggtaacttcccccctcgttcCtaacacctactctccctttaccccttcttacccttatgctccctccttatttattcaaaatgtttaaccccgttaccatctaattatTAGccAAtcgGgattaGcttAggaAtTGTgtggtCcaaccccagcCaatgGggaaaggacAcAgaagcacgggttctgtgcccgtgatagataaaaacaaatccccttctctcctttgtttggtgttcttctcattgtcactgcctgaggtgcaagaagcatacccccttctcaaaaagtaaatttgccttgctgagaaatcttttttgtctgagtgctggttttccttaggaactcaaacatttttttccaaacataagtatatccaaccaatcgtaatagagacatttgatctaattatacaatttctgattgtatttgagacaataaggcttctgataattttggagattgtgacattggaataaaggaaaatgtacaggactcatgaaaagctgaaatgctcacaaatgtcaagcaaaacaagagttaactaaacggactaaacttaagaaactgaagcaaatctttttgacttttccttggaatattgttaatctttgttttgtttttcagagtcaaggaaacttattttgaactatttatggcctttaataattgagtaaggtatactcttatgaagaaaatttggaacatgtttgtttctctctctgcctgttttctgtaaaatttggaaactatttgtgagtattcttatggcaatacagttgtttacatcagtgcaataatatttttttttttttttttggcaacaggacacaattggaaaaggtcattattttaccaaggctttgactggaagggtatgcttccctttaaggagtcaatctcaacttgcagctgataaaagcccaatgggagactggcctcataccctcatctacacagtccctgtacagggtttctaacctgtggtcagtaaagaattccaccttatagcaggtctaggagctccaaatttatcttgggaccctaagaggagagaatcacccaactcacaggtgtttgaggatacaatcccatggttgtgctcagatttaaaagttcttttctgagattccttgtggaacagaattccatcaaagccaatccaaaaggcctatgtagaaataattattcttgctgcactttatgca

>herv4_10410_100015_con_gagputein

mgnvssktgklkvesdeipsdsplglmlkywkdnertkykrkqqmikyfcfiwvrepilkpslfwpkfgsneericqllMGNTpSKtGskkdeidytlrkkkdsnkdIPpDSPLGLMLKYWKdNERTKHKKKQQMIKYCCFIWTqePILKPSiFWPKFGSnEDvmCQLLIqYVNDKsPVSQEEldyALCWrqgpvglalLfPLKttrekPnlasqneksdeltpmpkdsqststWDPLdHLPplstppppppqnlpsPqAAaaapdPsPdpspahvipPPlYNPdSwelsshepvpsqpcppKYpSLKGLQrEiEQCKvKDIQNFPFPSTsgKESAPTLFPLrevPqgvgaggggggaIgFVNAPLTSSEVqnLKKkELKPqatLLDDPYGVADQIgDQFLGPQLYTWaELMSILGILFSGEERSdMIhRAAMvvWErEHppitpPgQNVPtadQKFPAQDtPqWDNNNaAHqENMQDLREMIIKGIrESVPqTQNLSKAFDIQQqEKDEGPMeFLDRLKEQMRKYAGLdLEDPLGQGMLKLHFVTkSWPdisfzktlsDIsKKLQKIeNWkdrpisELLRlepqvkzpqvllrEAQKVYvvRrdEeqkkQKqKtKlMlstfqqgapnphtskqssqgarnyKGpkpspqlkgpkppskgprpsltrPyKeygGakpkNPrTerEeGQdRCyKCGRtGHFKRECPikelkkEkealpLmtfeEeegvqglclfyleshqeplinlev

>herv4_10410_100015_con_proputein

llifegggqglclfyleshQePLINLeVGPKhElITFLVDSGaarSSVcfpPsniacSSEELLVSGVKGEGFKAKILEeTEVrYQdqSAHIQFLLIPEAGTNLLGRDLMLKLGIGLQVsPrGFLTSLNLLTTaDEKYIhPnVWskEgNrgklqvppihiklktpgevvrrkqypipperrigl

>herv4_10410_100015_con_polputein

lvvsgvkgegiklkilketevrckncsanveflliseagtnllgrnlmlklgiglrigsegfltslnllttaeekyihpdvwaregnrgklqvppihiklktpgevvrRKqyPIPLEGRIGLKPiIEgLIKDGLLEPCMSPYNTPILpPVKKsDGSYrLVQDLrAINQIVQTTHPvVPNPYTILSKIPYnHQWFTVIDLKDAFWACPLAEDSzDIFAFEWEDPHSGzKQQYrWTVLPQGFTDSPNsLFGQILEQVLEKVsiPkqICLLQYVDDlLISGeDIEKVadFSTHILNHLqfEGLwVSKgKLQyVEPEVKYLGHLIsSaGKzRIGPErVEGIVSLPLPqTKQELRkKFLGLVGYChLWIDSYALksKlLYqKLtQeKPDpLLWTSEEvsdQVEELKhlLITAPVLALPSLEKPFHLFVNVnnplqGVALGVLTQEHGGhrhqQPVAFLSKVLDPVTCGWPqCIQSIAATAlLveepESRKLTFGGKLTVSTPHQVRTILNQKAGRWLTDSglslvlllRILKYEAILLEKDDLTltltLTTDNSLNpagflggPAGFLTGnPNLektpcprpKRpEHtCLDLIdYhtKVrPDLgETPFkTGrgHLFIDGSSQVIEGKkRHNGYSViDGETLeEieESGrLPNgNWSAQTCeELFiALsQALKyLQNQEGTIYTDSKYAFGVAHTFGKIWTERGLINSKGQDLVHKlltasflavvlELItQVLnNLQLPEEIAIVHvpghVPGliHQKsLSFeSrliGNNLADQIAKgQAAvSSetpiFHLTPcLPPptssptaiPIFSstEKEKLIKIGakencaakEnSEGKtaWvLPDQREMLSKPlLMREiLSqLHQGgTHWGPQAMCDAVLrVYGCIGIYTLAKQVTDSCLvCKKTnttpkknKQtlkKlPLGGRnPGLRPFQSvQiDYTEMPPIGhLKYLLVIVDHLTHWVEAIPFSsATANaNVVKALiENIaiPRFGLIENIDSDNGTHFTAHiIKKLaQvLDIkWEYHtPWHPpSSGRVgERMNpQTLKNHLTKLvLETrLPtWTKCLPIALLRIqTAPrKDiGLSPtitgfffvYEMLYGLPYLhSTaDIPtFETKDQFLknYiLgylSSTfSsLktkgLLAQtPpLefpvhqhqpgDHvLIKswKEgKLePaWEGPYlyLVLLTllvgvTETaVrTaekgwphhwthhtzvptrvkkappppimglcsraksnqakvktslltplpeswaivpgeskgvkliclfllllatsnPtklklkrstsssslyffsfqhkvilssvinaslalslp

>herv4_10410_100015_con_envputein

mghsprgkpyqtkakksltlfhlmyssfffpssiadakvilillsispqtlallallllpcgdlasqkqlslsekylcpvwlssvlsllnldelilgdfdkdpphnqscslrndvellchswsnvlwttkeqgwtaptsvcnflkpyihftkgsappncqynqcNPvqiiIliPtSssPslSqfpsLSrFYgmGAevsgtdPIGsFemhFitpspptpspkpsqsggttwppppSklShNeTiVpPpsNDKtKVAIVEVKDLKQTLAiETGYQDANAWLEWIKYSVrTLNKSnCYACAHsRPEAQIVPFPLGWSSSqPGMsCMVALFQdpTAWGNeSCqaLSLLFPtlrvaeVQHPAGQPPRAIQlPSpdtnFTSCLSqQGENLAFLGDLKGCSELKpFQELTnQSALvHPzAdVWWYCGGPLLDTLPSNWSGTCALvQLAIPFTLAFHQPekgKtqhhKaReaPhGSFDSHVYiDAIGVPRGVPDeFKArNQIAAGFESiLFWWvTiNKNVDWINYIYYNQQrFINYTRDAIKGIAEQLGPTSqqmAWENRIALDMILAEKGGVCVMIgTQCCTFiipaNNTAPdGTITKALQGLTallsNELAKNSGiNDPFTgLmEKWFGKWKGlmsSIlTSLAIVIGVLILVGCCIIPCIcGLVQRLIETaLTKTSLssPPPYSDKLFLLEnQaEQQSQdMLKKFEEeavrelqKlrgeegvknefzislqrigmsvcsilcllllnltfsslpfslaltlplllfalvlpsvsasvlipfligkp

**HERVADP**

>hervadp_10420_100007_con_chaindnarm

tgctttagtataagtttaagcaggcttcatggaaataaaggaataaagaagctgagaaaaaattttaaatggtccatttttaaggcataataaatctaagtactggcagccagcctgcaaatgtaaatttcaaaacgcatggctcatgcacctAgaaagtcataataagtgaacagaaTgtAgaggtcaggggtcagcccataaAaggataaaaGAagtatctgtagtttcatTatTgggaaaTcgAAacttAaGcagggaAAggggactagggtAtAacCTtataagggggAtaatgaaACtaaTAGgcaacaTctaggAagATTGTAacCCCaTAGtAcTcaaccaATGAgGAtaatAcTGGGggAgGGACtTGcatgCtAGGAgATAAATtaCcTgctgTaAagCtGccccgGGtgTGCcTGCCTACCAGACACCcgATCTTGCAAGtAccgCCATTAAAAGTcTtgaactatcCTTCcgcTgTTcttcgTgTCTCataAGTCCATTCtTTGgGTTTGGAtgGGTGAATgTGTgatTTCTCACAAAgtctTGGGggcccatctgGGATCTCctTGTGCCTGtgTgGAGTGGGgtgatcaAcTCcaGccaAGAGgggAGAtacgTCCCACccaATTTaagGTGGCCcaCTCTgTCcgGGcaTcctgGcTCcCtacAGAggCcatAgAcAAAcCtgAGActgTTATTcaggAgGcAgcaGAggcaacAcAgGGAgAActtgAAGCAGgCACtgtttgaGcAAcCAGGcaAcCTcgTGCAtgAGtCcaagGtAgGAaAATTggACTaTaagTaCTgCCTTgGTggGTTGGgCATTTTCaGAGGTcgAGTGgttcacTgTGtgTgACTGAGAtgTATCcTcAGAtAtGAAGCaAgTGCaGAGTCCCAATcCatggTTCtgTTCTCCcgcgagGgaAAtggccagAGAtaGacaAAgcaATtcttgaagtgatttttGGGTGTGCAAGAAACCTCcAGTcggggaagtGgGgGTTGAtGTACACaGGGAAgaAaGCTCAGACAcAgaGACTGACcaAAAATGGGAAACAGAAATTCTAgGCCTAGGGgACAAagGAAAGAGGGAGCCAAaGAgACtCcCTCTGACATTCCCCcggATAGTCCttTGGGgAGAATGcTgcAGGtTTGgAGGgACAACcCttgAAcCAGgGAcAAgGAAAAgCaAAAGATaAtAAAGtaTTGCTgTTTtATCTGGCCcAAAGAcCCcATTcaTAAgCCtTcggTCTTTttTGGCCTAAGTTTGGCTCAGatgAggATTGGGTGTGCCAAGCTTTAATTCTCTATGTgAATgATAAAACcCCAtCCTCACAAGAaGAGaTAgGTTAtgcTcTCTGcTGGATCAAGgAATTAGccCCccCATgTTcCCccTCAAAGAAGaAGAAAAagAgCcTAgTAAAaagccCttacCCAgtgaaaagcCCTGGGAcCCCCTAtCATgCtTgCCcCacccccTCCATAcaTCTCACAAAATAggGgACAGGAAgATaCAggaagaaacAGGGGCAgcAGGAGGGTtAGAGGAAGAaAgAcCTGgAGaCCATGgggGAgctaAACCAACTGCTCCTTTAAATCCTTATCCaAATTTaAGAAAAgAATTAGAACAgTGTAAGAgGGATATTGAGAACTTCCCTaTcCCtTCCAcACAGCAGgcaTCTAgCaaacaTGTTCCCtCTTAGGGAAGttCCcATgGGACAGGGAGAgaTTGGcTTTGTAAaTGCtCCTcTtACAAGTACTGAagTtAGgAATTTcAAgAAGGAAATGAaacCACTcCTAgAAGATcCcCTcgGTTTAGCAGAcCAgCTGGAcCAATTCcTAGGACcCAGcTTTTAcACcTGGGCTGAAATgATGTCTATCATGAATATCCTgTTcACaGGAgAAGAAAGGGGAATgATTAGgAGaGcggCCATgACCAtcTGGGAgAGgCAaCAcCCTCCcaGGCAAGgAGTCTTGCCAGCtgAACAAAAATTTCCAAATGTcaATCCcaAATGgGATAATAATGATCCCAGGgAccagGCCCAAATGCAgGACCTcAgGGAACTAATAATTAAAgGGATCAAAGAGTCCACTCCTAGGACACAAAATGTCTcAaAgGCATTcgAGATTCAACAAGAAcaaAAAGAggaAaACTCCCTCTGCATTCCTgCAGAGGCTCAGAGATCAgATgAGAAAAtAaaaaaaaataaaaaagaaaacCTCcaGATTAGATccaGAGGACCCAGTAGGGCAAGGCCTTTTgAAGGTTAACTTTGTAACTAAgAGCTGGCCTGAcATTACaAAAAAAaaTTACAAAAGATTgATGGATGGAATGAgAAACcaAttgAGGAaTTAcTGAGGGAAgCTcAGAaGGTCTTTgTAaGgAGAGAgGAAGAgAaaCAgAAAcAAAAaGcaAAAATCATgGTTTCcACTGTggAaGAgGTAGTcAgAAAaAggtTAgATCAAgAtcCccCTtgaAGGAGAcaAGgGAAtaatAGaTttcgaCAaAgAGaaAGaAGgGAAATaCAgGGAAAAgcTCCTAAgACTaTtgAGTGGATGTTACaAGTgTGGaAAgCCAGGACATTTTAAgAgaGAaTgtCctgAATGGAAAAAataagcccaagagaAaGAAAAGGTGATcCCCCTtATGACtaTTGATGAAGAcTAGgcgGGgTCAGgGGtTCCTTcTGAGTAGGTCCCACCtaccAGGAAcCcTTGATAAAtTTGAAGGTGGGACCTgAgGggGAAGAAGTAACATTTTTGGTtGATAcTGGgGtgGcTTGCTCCTCCCTAATTCACCAACCAAGGGGtACAGAActcTcTAAGgAAAaAaTtGAcAGTaTCaggggTaAAAgGGGAGgGATTTCAGGTTCTaATATTCAAGAAAATGtTAATTAGaTTggGAtCAgAACAaAtTaAagagTCAcTCTTATATGTTCCtgAAGCAGgAACTAACcTCCTggGTcgAgACcTGAtTGTGAGAtTGGGTTTAGGATTAGGAATAGAGGAaGGAcAAaTAAAAGTaATgATGtcgaGGCCTCCTAACAgAGGAGGagGAAAGAAAtctcaAttTAATCCCCTTGTGTGGgTTAGgaAAaGGCAACAGGGgAGgGTTAAAAATCACACCCTTACAaATTGAACTAAAACAACCAGGAGAAcTAGTTTGCAGAAAACAATAtagaaaaaacagaTCCCATtTCTATTgAAGGGAGAAAAaagGTCTCCAACgGGTAATAGAGGGATTaATtAAAGATGGACTATTaGAACCCTGCATGTCACCcATACAAtACTCCAATTCTCCCAGTtctcaCAAaAAgCcTGAgcTgGGTcgTATAGATTgGTGCAAGATCTAAGGGCTATAAATCAAATTGTTCAGACcTACCACCCTGTGGTGCCTAACCCCTACACCCTgtttctttttctttttttttcctctgtactccCCTTAGTAAGATAgCCCTATGAACATAAGTGGTTCAGTGTGGTGGATCTaAAAGATGCATTCTGGGCATGTCCCcTAGACTtTAGGAgTAGGGAgacacacCCTCTTTGcCTTTGAATgGGAAAATCCtATaAACTGGGAGAAAAtCAACAcGTAcCgCTGGACTGTGCTGCCACAAGGtTTCAcgGAAGCCCCAAACttATTTggTCAAGtCTTAGAAAAgGTcCTGGAGGAATTCCAAcCTTCCAGGGGAACCCAGTTGTTACAATAccgTAGATaATCTTTTAATTTCTGGGGAGAAGAGGgCcAAGGTATCAGAAACCACcatAAgcTTgcttAaTTTCCTAGGaGAAAGGGGATTgTaAGTCTCTAAgAACAAAtTGCAgTTTGTAGAAAAAagAAGTTAAATATTTAGGACACCTGATTAGTGAAGGgAAGtagAGAATAAACCCAGAaAGAaTATcgGgAATAGTGGgtTCTgCCtTTgCCTAAgACAAAGAGAGAACTCcaaAAAATTTTtAGGTTTAACTGgcTAcTGTAGGTTATGGATTgACTCATATGCTcAAAAGAcAAAGAtTcTGTATCTCAAgtTACTAGAAgAGGAACCtAAtCCCTtGggtaacttaatggggttacccttctagtaaccCAATGGTCCcCAgAgGAAATTCgaggtAGGCAaTgaAaGAgCTAAAGCAGGCCCTCATtACAGCCCcGGtCCTGGCcCTCCCATCTTTAGAgAAAcCATTCCATCTGTtTGggtttccctccccactccccccaccccattccatctatttgTaACAGTAGAcCAGGGcgtgGCCCTTGGGGTGcTCACTCAAAcCTGGGGagGGAAGAGGcAAcCtGTTGCTTTTGtCTCCAAGCTTCTtGATCCTGTCTCTaagGGGTGGCCcAAATGTGTGCAAGcAgTAGCTGCCACAGCCCTGCTcaGGTAGAgGAGAGTcGAAAGCTAACCTTTGGTGGGGCCCTAATAGTAAGCAtatCCCCACAcCAGGTCAGGAATATATTAAATCaaAAAAGCcGGGAGATgGTTtgAAcagATTCtCAGAtTCTAAAATATGAAGCCATATTAcTAGgAAAAAAttttaaTgATTTGGTtgTAACAACAaATACTTGCCTGAATCCAGCCAGTTTCcTATGGAAAGGAGAGGAGAAcAAAGaaGaCATCAGACCAtAACTGcTTAGATATCATAGAATAcCAAACCAAAattaGTTAGACCAgACCTTAGgGAAGCTCCACTAcCATGATGGGATAAGGCTGTTTGTGGATGGGTCgTCCtgAGtatgtTGAtAGAtGgCAAagAaAcAtAcaaATGgTTATGCTGTCAtTGATGaAtAAtAAACAcTcCTTtATGTGAGAAAGGTAGATTAgCCTAATGGCTGGTCAGCCCAAACCTGTgAATTAtATGCTCTTAACCAGGCCCTAAAgCTCCTtGAAGGCCAAGAAGGCACTATATATACTGATTCTAAATATGCCTAaTGGgGTGGTACACACtTTTGGAAAAATCTGGACAGAGCaGGGCCTAATAAATAGCAGGGGAAAAGAATTGGTACATGGgGAACTGGTCAAACAGGTTTTAGAAAGCCTCCTGCTTCCAGCAGAGGTAGCtaatCATAGTTCATGTAAATGGTCATCAgAAAGGGAACACTATAGAAGCTGtAGGAAACAGGCTTGCAGATGAAGCTGCTAAGCAAGCCTCCCTGGAGGAAGAAaTTAGACTATTTAGCCTgATCCCAGAtATCCCTAAGGTAGTATTAAGgCCCCAgTTTACccccaataccCAaAGAGGAGAAGGAAGAAtTAGaCAgGATAGGGGTCACTCAAACTGAAtaGATGGGAaATGtGGTaCTTCCTGATGGGAGAGAagctAATGATAAGtAAACCCCTAATGAGAGAACTAATGTCTATATTACACAAAGGGtAGTCAcTGGGaggttaGACCCCAGGCTCTGTGTgATGCAATACTtAGGAATTAgattTGGGTGTgtATAGGGAgaagggaaaagctcTTTATACCCTTGgCTAAtgttACAAGTATGTGGAAGTTGTGTtAACTTGTCAAAGgATAtcctgAACAAAAAGGTaATTAGAAAACAGGCCACgGGAGGAAGACCTCCcGGACTAAGACCATTTCAAAGttcCATTCAAGTAGgATTTCACAGAAATGCCCAAtaAaTAGGAAGACTAAAGTcgATTTACTGGTaATcGTAGATCACCTTTCTGGCTGGGTGGAAGCCTTTCCCCTaaTCCAACAGCCAtCTgCCgGGAATGTGGTCAAAAgggagggatttcaggttcTAATATTAgAACAGAaaTTgTACCTAGATTTGGCCTGGTGGAAAATgaggtgtgactcttATTGATTCAGACAATGGGAGCCACTTTACCTggCAAGGGTGTTAAGGtGGAATTATGGttAAGGTTTACAAATTAGATGGGATTATCACACCCCcTTGGCATCCCCCTTCCTCTGGAAAGGTAGAAAGAATGAATCAAACTCTCAAAAttaAGCATATCACCAAACTAATCTTAGAAACTAAAcccttATGCCTTGGACCAAAaTGTCTCCCAATtgAGCACTCCTTAGGATTAGGACAaGCCCCAtccccgctAGAAAAGACTTGGGATTGTCtCCCCTAtgAGTTATTATATGGaCTCCCATATTTGGGCAGaGCTACAGATCTTCCTACTATGGAAtACtgaagaccaCAAGGAtCAATTtTTAAaGAAATTATATACTGGCCcATATCCTCCACCCTGTCATCCCTTAGGTTAAAAGGACTTCTGACTCAAACTCTgCCTCgTTGAGTTCAcAGTTCACCACTTCCAGgaCCTGGtGACTTGGTGCTGATTAAGACTTGGAAAGAAGACAAGaCTCCACCCAAGCTGGGAAGGTCCCTATCAAGTGCTCCTGACCACTGAGAcaGCCaTGggtCAAACAGCTGAAtGGGtccgGGTGGACTCACaacctTATACTCGAGTCAAGGGACTGcaatatGTAAAAGAtcttttGACCCTGGAAGGGAgGGGAAccgaggtatcAAAGACCAGTGGAAAGTGCATatGGGTCACCTAtgctacgggaggacctcaggcacaggtggttccatttcccctaggatgggatactgatcctgaagaagtggattgcacgttggctctataccaggacaaggatgcatggggaaacaagacttataagagtctgtcactgctctttcccacattgcagaggttaactttaAGGAACCCTTAAAGTTAAgtCTCTgAGAAAAAattgcagttTCTAaAAAGAAAACATGGGCatttTGGacaCCCCATTTCTGGAAGTTAATATGGcaTaGGATgaGcTActATACcAaGaGCaAAAGggcgtcaAAATGgagaatatggaAACTGGtAGGGGACTCCTccggttcaaCTACCCAATgCAgGTTggTAATTAActcaTGTAACCAAgAcggtAgcatatgtgactgaagctaaGCACCCCAaACTATAAaATTTGATGCCTGatcCCAtgcAGTCTTACCTTGTGGGAATTtTAGAAAATCAGAGACAGCTCTCaCAaGCAgtcATAAATATCTtTGCCCTGAACCAGataatacagGttacAgTAgggCAtCaCCCTGCCCCAGCTGGGATGATGTaTGGcctTGGACtACCCActcATTTCAGGGTTGGAcAGTAgcAACATGgGGTtgGGTAACTCCaAgCTGcttGAGACtgtCCTTAAAGAATAAAcTACATcTGTCCAAGGGagCTCCctGCCAaATAACTGCCAGtaAATTTAGAATGCAATCCTggtggggccctaATACTCATcACCATtAACAATCCAGCaaTAtTCTAGACCAAGAAgCCAAaAGgtggttaaTAGcaTCTtGGGTATATGGgTTAGGGattagggGCAGACATCACtAGGGAAAGAtccaccccCCCCcTAGGGTGATaacagatacTTGTTCgaaTCAAACTAATCacctatggaaaggagagAAgAACTCAcagACCTCacacCctAtTTGCCTGGctaGACTACcaacTCCAACCtagaCCAGACCCTAATAAAccCTTTAgTCCACCAAATAATgACCCTAAAAGaGTAAAAATAATTgAGtgGTAAAgGATTTAAGGCAAACCTTAGAAATTGAGACAGttgGGTAcGGGGATGTGAATGCCTGGGTcaAATGGGTCAAATTTTCgGTACAAGCCCTCAACAAGAGTAACTGCTgtaattgctATGCATGTGCTGCgGGAcGACCTCAGGtCACAGGTGGTTCCATTTCCCCTAGGATGGGATACCAAgttttgacggtctgctctttgtatagtagtccatcctagccatattaacttccagaaatggggccagcacatgttttctttttagatttttcttagagttaaTCCTGAAGGAATGCATTGCATGTTGGCTCTATACCAGGACAAGGATGCATGGGGgAAATgAGACTTGTAAaGAGTCTGTCATTGtctgtcattgCTCTTTCCCACATTGCAGAGGTCAGATCCCAGAGCAATCCCCTCATTCTCTATAGGGAATATGAACCACTCCTCTTGCCTCTCTAGGCAGGGGGCAGAGgaggTTCAATAAGCCCATGGGAGAACTCTCgAaCTTGTACCCACATCCTAAACATCACTGGTtGAGTCAGGCAATGGCAATTACTCAGCTCTCCATATAaCCCCGGGCTGATGTCTGGTGGTATTGTGGGAAAAGGAACCTCCaTtAACCTGTTagttACCaTCCAATTGGACtGGGACTTGTGCTTTAGTCCAATTGGCCATTCCCTTCACCCTGGCATTCCATAAGATACCtGAAAATACACATGGCCACCgAAACCGGAGAGATTTAACAAATTCTTTTGATCCCAATATATATGTTGACTCAATAGGAGTCCCTAGGgggggGGGGTGCCTAATAAATTTAAGGCCCgAAACCAAATAGCTGCTGGGTTTGAGTCAGCACTCTTCTGGTGGTCAACTATTAATAAAAATGTGGATTGGATTAACTACATCTATTATAATCAACAGAGATTCATCAATTATACTCgGGACaCCCTCAAAaGGGGTGGCTAGCCAGTTAGATGCCACCAGCCAAATGGCCTGGGAAAACAGGCTTGCACTAGACATAATACTAGCAGAAAAAGGGGGtGTATGTGTTATGCTGGGTGtGGAAATGTTGtACTTTCATatTCCCAACAATACTGgggaccaattgtCCCCAGATGGGACCATCACAAAAGcactaCTTTACAAGGACTaACAACTCTAGCCAAtAAACTGGCAGAAtAATGCTGGAATTgATgACCCATTTACGGGTTGGCTAGAAGGTTGGTTTGGAAAATGGAAAGGcATGGTAGCTcTCAATCCTTACATCTCTCAaTAATTGTGGCAGGAGTCTTAACAGCAGTGGGATGTTtGTATTATCCCcTGTGTGAGGGGACTAGCACAGAGATTAATTgAAACAGCTATTAATAAACAAATGCCCATaACTTAtCCAGCAAAATtgAACCTGCTACTATTAGAgAACCAAATTAAACTCACTCTCCTATaAGGAAGAAAGTAAACAACTTCTAGAGtGATTCAAGGACCAAAAGGcGTTTAGATGAAAATGAGACCAAAGGAAGTAAAcccTAGAAAAGAGGAGGGAATTTGTGAGAAAATATTTTAAtgATGGTCCATTTTCAAGGCAtgcctcgagtagtgATAAATCTAAGTAaaCTGGCAGgacaaaatataCCAGCCTGCgGATGTaACAAACtaCATGGCTCATcggGCACCTAGAAAGTCAtGATAAGTGAACatgcgtttagtctAGAATGTAGAtttactaacctGGaacAGGGGTCAGCCCATAAAAGGGAAGAAAGTTTCgttgctaccaTtATTGGGAAATCaAAAcTTAAGcaGccagGGAAgGGGaCtaGGgTATAACCTTATAAGGGGgATAagaaaatgcagtgacaaccccaaaaATgAAACTTAaaactctcctacccGGtgAtcaataggTaacaaCCaGGAAGATTGTAACCCCATAGTACTCaACCAATGAGGAACTgGGGGAGGGAcTTGCaTGCTAGGAGATAAATTACcTGcTGTtAACTGCcCCaGGTGTGCCTGCCTACCAgACACCCgATCTTGCAAGACcGcCATTAAAAgTCTtgcTTctgCTgTtCttcgTGtcTctaAgTcCATtcTTtgGgtTtgGatgggtgAgtgtgtttctcacataattagagtttaaattaaatgatgatacaaagatacatttgtctgacctttaaaacattgttcttttagtccaagttattttttaagagtataaatgaccctccctgagacccgcccaccccca

>hervadp_10420_100007_con_gagputein

gwrqhrqksgmaapqasatamggkliglsvlggwagivggcvpviglvlpagawaesqsavlfscegsvqrwaknlggaqnlpvvgveytggsaqtqtdqkwetnirgLGqkkegeketLpLiFPqsvLgrmlqglggqPltrdKeKaKdiKylcFiwpKdPihkpsVFWPKFGSDEnWVCQALILYVNDKTppSSQEEiGYaLCWIKELaPmFPlKEEEKEpSKepsPSEKPWDPLsaclPPpYiSQNRgQEdQGAaGGLEEERPGDHGGaKPTAPLNPYPNLRKELEQCKRDIENFPIPStQQaSSMFPLREVPMGQGEiGFVNAPLTStEVRNFKKEMKPLLEDPLGLAdQLDQFLGpSFYTWAEMMSImNILFTGEERGMIRRaAMTIWERQHPPrQGVLPAeQKFPNVnPeWDNNDPRDqAQMQDLRELIIKGIKESTPRTQNVSKAFEIQQEpKEETPSAFLQRLRDQMRKcYSRLDpEDPVGQGLLKVNFVTKSWPDITKKLQKIdGWNEKPIEELLREAQKVFVRREEEKQKQKAKImVSTVEEVVRKrLdQdPplRRqgNdRfqHRERREiqGKaPKTmSgCYKCGKPGHFKRECPEWKKeEKVIPlMTiDEDsg

>hervadp_10420_100007_con_proputein

epLINLKVGPEGnEvTFLVDTGAAcSSLIHQPRgTELSKEKLtVSGvKREGFQVPIfkKmLirlGakqteaSLlyVPKAgTNLLGgnLIVgLGLgLGIgEGQIKViMGLLtEEEERKlnplvgikkatgegkl

>hervadp_10420_100007_con_polputein

nlkvgpegeevtflvdtgvacsslihqprgtevsknkltvsvkgegfqvlifkkmvirlgpeqieesllyvpeagtnllgqdlivrlglglgieegqikvmmgllteeeerkinplvwvregnrgglkItpLQIeLKQPgEvvcRkqkqYPIsteGRKgLQpVIegLIKDGLLEPCMSPYnptpilpvkKpdgsyRLVQDLRaINQIVQThHPVVPNPYtLLsKIPYEHKWFsVVDLKDaFWAcpLDlrsrdLFafewENpITGRKQQYhwTVLPQGFmEAPNlFgQiLEKVLEEFQPSRGTQLLQYVDnLLISGErRaeVSeTTisLLNFLGERGLqVSKNKLQFVEKEVKYLGHLISEGKqRINPERISGIVGLPLPKTKRELqKFLGLTGYCRLWIdSYaqKtkIlYlklleeeppnplqwspeEIQAvKELKQALItAPVLaLpsLEKpFHLfVTVDQGvaLGVLTQTwGGKRQPVAFVSKLLdPVSqGWPeCVQAvAATaLLVEeSqKLTFGGALIVStPHQVRNILNqKagrwLtDSqIlKYEAILLEKddlvvTTdtCLNpASFLwKGEenketSDhNCLDIIEYQTKVrpdLrEAPLHDgiRLFVDgssqvidgkrhnkmanntigyAvidgNKhsLCEKGRLPNGWsAQTCELyaLNQALkLLegQEGTIYTDSKYAyGVvHTFgKIWTEqGLINSRGKeLVhGELvKQvLESLLlPAEVAIVhVNGHQKGNTIEAvGNrLADeAAKQASLeEEIRLFSLIPdIPKvVLrPQFtkeekEeLgkiGvTQTEDGkWVLPdGREmiskpimreLmSILHKGSHWGPQAlCdAILrNYgCIGIYTLaKqVCGSCVTCQRINKKViRKQatGGRPPgLRPFQSIQVDFTEMPKvGRLKYLLVIVDHLSGWVEAFPLpTATagNVVKIILeQIvPRFGLVENIDSDNGSHFTSRVLRGIMeGLQIRWDYHTPWHPPSSgGKVERMNQTLKKHITKLILETKMPWTKCLPIALlLRIRTAtaPRKDLGLSPYELLYGLPYLGRATDLPTMETedKDQFLRNYILAISSTLSSLRLKGLLTQTLPLEFtvHHFQPGdLVLIKTWKEDKLHPSWEGPYQVLLTTetAmQTAEzGwThyTqVKgLvketlEggvskgmplgwstnpllllgrekrpvkvygsleeplkltlrktgrelggapllikltkggg

>hervadp_10420_100007_con_envputein

fglsamrmgwawpswtpvKglvkltkgsppnncqNLecsPiLItidspavLnQEPKVASrswVYGLGADitGKdPlGvFVLKLIKNSTSHLPGTTPTPdPNKhFSPPNNDPKRVKIIEVKDLRQTLEIETGdvnawvvnawveWVKFsVQALNKSNcYTCAvGgPQAQVVPFPLGWDTnPEgMCCMLALYQDKDAWGNETCKSLSLLFPTLQRSDPRAIPSFStGNMNHSSCLSRQGAEFNKPMGELSTCTHILNITgESGNGNYSALHIPpgmvwGyCGKRNLcNLLPSNWTGTCALVQLaIaFTLAFHKIPENTHGHqNwRDLTNSFDpNIyVDSIGVPrGVPNeFKAqNQIaAGFeSaLFWWSTINKNVDWINYIYYNQQRFISYTRDaLKGVASQLDATSQMAWENRLaLDIILAEKGGICVMLGGKcCTFIPsNTAPDGTITKALqGlTTLANKLAeNAGIndPFMGWlEgwFgKwKgmsfNpYISHNCGRSLNsSGiliyPCVrGLAQrLIetAInKQMPMtYQqNnlLLlEtKLNsLsyeeesnf

**HERV9**

>herv9_10320_100006_con_chaindnarm

tggctgaggagtgcgggcacatagcacaggactggaaggcagctcgacctgcagtcatggtttgggatccaatgagtgaagacagctgggctcctgagtctggtggggatgtggagaacctttatgtctagctcagggattgtaaatacaccaataggcattttgtatctagctcaaggattgtaaacacaccaatcagcaccttgtgtctagctcagggattgtgaatacaccaatcaacactctgtatctagctattttggtggggacttggagaacctttgtgtctagctcagggattgtaaatgcaccaatcagcaccctgtaaaaacttactaattagctcactgatttaaaacggaccaatcagcactctgtaaaaatggaccaatcagctctctgtaaaagtggAcCAATCaGcAgGAtgTGggtgGGGCCAgAtAAggGAATAAAAGCaGGCcacCcgAGCCAGCAgtgGCAACtaccgcTcgGGTCaaCCCTTCCAcaCTGTGGAAGcTTTGTTCTTTcgCTCTTcaCAATAAATCTTGCTgCTGCTCACTCTTTGGGTCcaCaCTaCCTTTATGAGCTggTAACACTCaCcgcgaaggtcttcagcttaaactaaaaagaaaggaagaccacgaacctaaagggaggaattaacaatctgcAgcttgcCactcctgaagtctaaattcctaaaccttataaacactctttgtaaaatactgtacaatcctgtgttctttgcaacgctctgacaaggtcattataaaatgcaccaatcagcgcactgtaaaaatgccagcaagACcAcGgctctaaaatcAaCCcAccaggaggaaagaacAaaaaccagctgggaGgAAtaAacacaaActccaGacacgCcaCCTTtAAGAGCTGTAACACTCACtGCgAAGGTCTGCAGCTTCACTCCTGAAGCCAaGtgAGACCACgAACCCACCAGAAGGAAgAAAcTCCggACACaTCTcacaataaaagAACATCaGAAGGaACAAACTCtGGAtcCACaCCATCTTTAAGAACTgaGTAACACTCACCGCGAGGGTCCttagggaatatactcaaaacaaatctaattgttaaaaaaaacaaaatgggaacacaccaactttaagaactgtgacactcaccacaaatatccGCGGCTTCATTCTTGAAGTCtAGCGAGACCAAGAACCCACcggtaggaagaaactccagacaaatctaaacgtctgaAgGAACCAATTCcGGACACATTatctTTGGcGACCacGAcactcaccgtgaGGGACtaTcgCCTcatcgccaaggtcggtgagactatcacccaacgccaagcggtgagaccattgcctatcgccgagcggtgagactatcgccTATCGCCAAGCgGTGAtctggGTACCATCGGACCCCTTTCgCTTGCTATcccTCTGTCCTATTTTTCCTTAGAATTtCGGGGGCTAAATACCGGGCACCTGTCAGCCAGTTAAAAGCGACcaatgtgacTAGCGCGGCCGCcGGACTAAAGACACGGGTGTCAGGCTTTCTGGGAAAGGGCTttCTCTAACAACCCCCGgACTCTTCGGAGTTGGGAGCGTTGGTTTGCCTGGAAaCCAGCTTCCGCTTTTCCTGTACTTCTGGGCTGAtactGCCGAGGGTCGAtCAGAGAGGAAAGCCATTCAGCTcCCgGGGgTCCCGACAACAAGTTGGTTGACCCTGCGGCCATGAGCaGAACTCTCAAAGTCATGTCGCCCAAGCGAGACTCtcGCCCATCTATCCTAgtTCTATCCTGACCCTTGCCTCCTGGGTCCTAATGCCTGTCAcGACAAAtCTTCCTCTCGCCTCTCTTCTCCGccctttacaAGGCTAGTCCTGCTTCTAAAAACcaaCACTCCCTGTattagaaCTCTGGTGCTTTTCTAGTTTCTCCTATAAGAATGATTTtcatgatgccccggccatgagtggaatgctcaaaagcatgttgctcaagcaagactcacccatctatcctatctattctgacccttgccccctgggtcctaacacctgccaaacaaacttcctctcacctcttttctctgaggttggacccgtttctaaaaattgctacctgtctctggtgcttttctggaatttaggcaggtgttaccatCTAGTATAAACTcCAGGcaACTCTatacGTTaCCTTCTTTAGGCACCCGGGCTCACCAATCAGAAAGAcctaaattttaCATAATTTTTGCCCAAAGCCCCATCgtgggTAGGGGGGAtatctaCTATCTGGAATaccgtctatcctTTTAGGccATCCCTCCTCAGACTAGCAtGGCCTAACcAAAAGCTATTCCTGAAGCTAtcatgttgtGGATATGGGGAGCCTCAGAAAtgttgaattgTTGTAgactccaaaattggggggtataTCCTTCCTATTCATATgatgagAAGTGAGGACAgtttAAAGGCGTCACTCTTCCAAcggaagataCCCTGGAGagggagATCCCTTCCCTCCCTCAGGGTATGGCCCTCCACTTCATTTTTGGGGgCATAACATCTTTATAGGACAgGGGTAAGGTCCCAATACTAgACAGGAGAAaacgactgtaacaggTGCTTAGGACTCTtAACAGtatttcagaaatgcttcagtaagggtcattaaatctgatttttttgagtcctctttatggtttaggaggacaggcaaggtacagGTTTTgcGAGAATGCGTCgGTAAGGggaGCCAcatcCaTAAATCCGATTTTTCtcggTcGGTCCTCTTTGTGGTCTAGGAGGACAGGCAAGGGTGCAGgaacagGTTTTcGAGAATGCATCgGTgaAAGGGCCACaaTAAATCtgatccGACCTTCCTtgCGGTCCTCCTTGTGGTCTAGGAGGAAAACTAGTagGgcaaaggTTTCTGCTGCTGCgTCGGTGAGCccaacttttctgatcaggagggtccatggaccattgcaggttcttgggcaagaggctgctgctgctgcatcggttagcGCAACTATaTCCGATCAGCAGGGTCCAGGGACcgTTGcGGGTTCTTGGGcAgGgGtTGTTTCTgCTGCTGcgTcgGTGAGcaCAACTATTCtgATCAGCAGGGTCCAGGGACCGTTGcGGGTTCTTGGGCAGGGGGAGgagtccaggaAAcaAAACAAACCAAAACCGTGGGCGGTTTTtGTCTTTCaatttcAGATGGGAAACACTCAGGCATCAACAGGCTCACCCTTGagAAATGCATCCTAAGCCATTGGGACCAATTTGACCCACAAACCCTGgAAAAAGAGGCGGCTCATtgcagccatTTTTTTttttCTGCACTATGGCCTGGCCCCAATATTCTCTCTCTGATGGGGAAAAATGGCCacctgagggaagtataaattacaatactACCTGAGGGAAggaaggcacactgccccagaGTATAAAgttctctgggccagaagcccccaaccagatgatccaacaacaggaatgagggtgcctggggcaagtgccagctcatgccgtcaccctcactgagccccgggtaagattaaccactgagggccaggaaattgacttcctcctggacactggcgtgaccttctccgttttaatctcctgccctggacggctgtccccaaggtccgttacTTACAATACTATCCTGggacagcctgtaaccaggtatgtctcctacctcctCAGagCTTGACCTTTTCTGTAAGAGGGAAGGCAAATGGAGTGAAATACCTTATGgtatTCCAAGCTTTCTTTTtCATTGAAGGAGAATACACAAcaCTATGCAAAGCTTGCAATTTACATCCCACAGGAGGACCTCTCAGCTTACCCCCATATCCTAGCCTCCCTATAtaGCTCCCCTTCCTATTAATGATAAGCCTCCTCTAATCTCCCCctGCCCAGAAGGgcaaagAAATAAGCAAAGAAATCTCCAAAGGtACCACAAAAACcccaacCCCcccccGGGCTATCgGTTATGTCCCCTTCAAGCTGTaAtGGGGGAGGGGAATTTGGCCCAACCCGGGTACATGTCCCCTTCTCCCTCTCTGATTTAAAGCAGATCAAGGCAGACCTGGatGGAAGTTTTCAGATGATCCTGATAGGTACATAGATGTacCCTACAGGGTCTAGGGCAAACCTTCGAtCTCACTTGGAGAGATGTCATGCTATTGTTAGATCAAACCCTGGCCTTTAATGAtaaaaAAAGAATGcGGCTTTAGCTGCAGCCCGAGAGTTTGGAGATACCTGGTATCatcTTAGTCAAGTAAATaGATAGAATGACAGCtGAAcGAAAGGGACAAATTCCCTcACtGGTCAGCAAGCCATCCCCAGTATGGATCCCCACTGGGAaCCTTGACTCAGATCATGGGGACTGGAGTCGTAAACATCTGTTGACCTGTGTTCTAGAAGGACTAAGGAGAATTAGGAAAAAGCCCATGAATTATTCAATGATGTCCACCATAACTCAGGGAAAGGAAGAAAAaTCCTTCTGCCTTCCTCGAGccGGCTAtGGGAGGCCTTAAGAAAATAataTACTCCCCTGTCACCCGACTCACTCGAGGGTCAATTGATTCTAAAAGATAAGTTTATTACCCAATCAGCccagccGCAGATAaTCAGGAGAAAagcaccaGCTCCcAAAAGCaAGCCCTGGGCCCTGAACAAAATCTGGAGGCATTATTAAACCTGGCAACCTCGGTGTTCTATAATAGGGACCaaccAAGAGGAACAGGCCCAAAAGGAAAAGCGAGATCAGAGAAAGGCCaCAGCtcatggccCTTAGTCATGGCCCTCAGACAAACAAACCTTGGTGGTTCAGAGAGGACAGAAAAaTGGAGCAGGCCAATCACCaccCGGTAGGGCTTGTTATCAGTGTGGTTTACAAGGACACTTTAAAAAAaagatcatcGATTGTCCAATGAGAAACAAGCTGCCCCcCTCgTCCATGTCCACTATGCCGAGGCAATCACTGGAAGGTGCACTGCCCCAGAGGACAAcatccAGGTTCTCTGGGCCAGAAGCCCCCAACCAGATGATCCAACAACAGGACTGAGGGTGCCCGGGGCAAGCGCCAGCTCAaTGTCATCACCCccTCACTGAGCCCtCGGGTAtGTTTAACCATTGAGGGCCAGGAAATTGACTTCCTCCTGGACACTGGgCACGGCCTTCTCAGTGTTAATCTCCTGTCCTGGACGACTGTCCTCAAGGTCCGTTACCATCCGAGGAATCCTGGGACAGCCTtGTAACCAGGTATTTCTCCCACCTCCTCAGTTGTAATTGGGAGACTTTGCTCTTTTCACATGCCTTTCTTtGTTATGCCTGAAAGTCCCACACtcacCCTTATTAGGGAGGGATATATTAGCCAAAGCTGGAGCTATTATCTACATGAATATGGGGAACtAAGTTACCCATTTGTTGTCCcCCTACTTGAGtGAGGGAATCAACCCTGAAGTCTGGGCATTGGAAGGACAATTTGGAAGGGCAAAAAAaTGCCCAccacCCCAGTCCAAATCAGGCTAAAAGACCCCACCACTTTTCCTTATCAAAGGCAATATCCCTTAAGGCCTGAAGCTCATAAAGGATTACAGGATATTGTTtAAACATTTAAAAGCTCAAtcgaGGCTTAGTAAGGAAATGCAGgCAGTCCCTGCAACACCCCAATTCTAGGAGTACAAAAACCgAAatGGTCAGTGGAGACTAGTGCAAGATCTTAGACTCATagCAATGAGGCAGTAATcattatataTCCTCTATATCCAGTTGTACCCAACCCCTATACCCTGCTCTCTCAAATACCAGAGGAAGCAGAATGGTTtCACTGTTCTGGACCTCAAGGATGCCTTCTTCTGTATTCCCCTGCACTCTttgctttGACTCCCAGTTTCTCTTTGCCTTTGAGGATCCCACAaGACCACAgaggaCaTCttagcttactcccatatcctagcctccctatagctccccttcctattagtgataagcctccttatgtggacgtCCAACTTACaTGGACgGTCTTGCCCCAAGGGTTTtAGGGATAGCCCTCATCTGTTTGGTCAGGCACTggcccaatatctaggccaattctcaagtccaggcactGGCCCAAGATCTAGGCCACTTCTCAAGTCCAGGCACTCTGGTCCTTCAGTATGTGGATGATTTACTTTTGGCTACCAGTTCGGAAGCCTCATGCCAGCAGGCTACTCTAGATCTCTTGAACTTTCTAGCTAATCAAGGGTACAAGGTGTCTAGGTCGAAGGCCCAGCTTTGCCTACAGCAGGTCAAATATCTAGGCCTAATCTTAGCCAGAGGGAgCCAGGGCCCTCAGCAAGGAATGAATACAGCCTATACTGGCTTATCCTCaCCCTAAGACATTAAAACAGTTGtGGGGGgTTCCTTGGAATCACCGGCTTTTGCCGACTATGGATCCCCgGATACAGCGAGATAGCCAGGCCCCTCTATACTCtaaTAATCAAGGActattGACCCAGAGGGCAAATAaCTCATCTAGTAGAATGGGAACCAGAGGgCAGAAACAGCCTTCAAAACCTTAAAaaGCAGtaccactaGCCCTAGTtACAAGCTCgagcccctcccccctacccccacccccaagctcCAGCTTTAAGCCTTCCCACAGGACAAAACTTCTCTTTATACaTCACAGAGAGAGaaCAGGGATAGCTCTTGGgAGTCCTTACTCAGACTCGTGGGACAACCCCACAACCAGTGGCAtaggaaccccaTAaCCTAAGTAAGGAAATTGATGTAGTAGCAAAAGGCTGGCCTCACTGTTTAtGGGTAGTTGCgGCaGTGGCCATCTTAGTGTCAGAGGCTATCAAAATAATACAAGGAAAGGATCTCACTGTCTGGACTACTCATGATGTAAATGGCATACTAGGTGCCAAAGGAAGTTTATGGgtttatggCTATCAGACAACcGCCTACTTAGATACCAGGCgCTtagatactcctacttagatACTCCTTGAGGGACtGaccaGTGCTTCAAATACGcAcGTGTGTGGCCCTCAACCCTGCCACTTTTCTCCCAGAGGATGGGGAAaCCAATCGAGCATGACTGCCAACAAATTATAGTCCAGACTTATGCCACCCGAGATGATCTCTTAGAAGTCCCCTTAGCTAtATCCTGACCTTAACCTATATAtataCCGATGGAAGTTCATTTGTGGAGAATGGGATACGAAGGGCAGGTTATGCCATAGgTTAGTGATGTAACcGTACTTGAAAGTAAGCCTCTTCCCCCcaAGGGACCAGtGCCCAGTTAGCAGAACTAGTGGCACTTACCCGAGCCTTAGAACTGGGAAAGGGAAAAAGAATAAATGTGTATACAGATAGCAAGTATGCTTATCTAATCCTACATGCCCcATGCTGCAATATGGAAAGAAAGGGAGTTCCTAACCTCTGGGGaGAACCCCCATTAAATACCACAAGGAAATTATGGAGTTATTGCACGCAGTGCAAAAACCCAAGGAGGTGGCAgtcaGTCTTACACTGCCAAAGCCtaaggagaaaaATCAaAAAGGgTGAAGGAgaaaaggcatgaaaagaaaaggaaaGAAAAGGCAGAAGGAgcatAACCGTCaGGCAGATGCTGAGGCCAAAATTGCTGCCAGGcgGAACCTCCCATTAGAAATACCTAtGGAAGGACCCTTGGTATGGAACAAcCCCCCTCCAAGAGATTAAGCCCCAGTATTCCCcaACTGAAACAGAATGGGGACTTTCAcGGGGGgcactttCATAGTTTTCTCtcCCCTCgGGGTGGTTAAtGACAGAAGAAGGAAAGGTACTtAaaaaaggttTACCcgAAGCCAGCCAGTGGAAAATACTTAAAACCCTCCACCAAACTTTTCATATGGGTATTGAAAACACTCATCAAATGGCCAAATCCCTATTTACAGGGCCAAATCTCCTCcAGACCATCcatgACAgGTAGTCAAAGCCTGTGAGGggTGTGCCAAAGGAATAATCCCTTGGTCCATCaTAAGGCCCCTcTGGGGGgAACAAAGAATAGGTCACTATCCtGGAGtcccaacattaacattAGGACTGGCAGTTAGACTTCACCCATATGCCTtttgAAGTCAAAGGGATTTCAATACTaTGTTGGgatccagTCTGTGTTGATACCTTTACAAATTGGATAttctcGAAGCTTTCCCCTGCAAGACAGAcGAAGGCTCAGGAAGTGATTAAAGTCCTAATTCATGAAATAATTCCTAGATTTGGGCTTCCCCcAAAGCTTACAgAGTGACAATGGTCcggGaatggtcaggCTTTTAAAGCCAcgATAgccatgataACTCAGGGAATTTtCCAGGGcgCTAGGGATacaACAATATCACCTTCACTtcaccttcattGcgCCTGGAGGCCACAATCCTCAGGGAAGGTCgAGAAGGCAAATGAAACACacTCAAGAGGCACTTAAGGAAACTAACACAAGAAACTggaaactCATCTCCCATGGCCTActctttactcTtTTGCCCATGGCCTTGTTGAGAATCcGAAATTCTCCTCAaaatttgatcCAAAATcaaagGGGGCTCAGTCCATATGAAATGCTGTATGGAcGACcTTTTCTCACAAATGACCTCCTACTTGATCAGGAAAtGGCCAACTTGGTCAAAGATATAAtaaCTTCTTTGGCAAAATATCAAcaAAACCTTAAAAAcctaatgtcaaataaaaaacggtatccTACCTGAAGGATGTCACAGAGAAAAGGGgAAcAGAGTagagtTGTTTCAACCaGGcacccAGATCTAGTGaTTGGTCAAATCtCTCCCCTCTACCTCCCCaggATCTATGGACtCTTTaaGTGGGAAGGACCATACTcagTtAATCCTCTCTACcCCatcCACTGCAGTTAAGGTGGCAGGAGTGGAAtccTCTTGGATTCAaCCACACCcGAtGTTAAACTTTGGACAtCCCCTGAGGAACCTgCgGGACCaTCAGCTCAGGAGTCCCAAGATCAGCCAGACCAGCCTCctGATACACCTGcgAACcaTTGGAGGACTTGCATCTCCTATTTCAGAAGGAAAcATCaCCAGACTAAAAAGGCTCtaCTACcaCTGttATCCTGAGGAAAAAcaaCCCTTCCTcCTTAAAAaaaaAGATAAGTGAAAACCtACATaATctttatcattacttttaattacatCtCtcCttgCcCctttaaTggAAtcCtTTtaCtgttttaacacctctccttgcccctttaatggTCaTCatttactgtttcatcacaTtATTaAGCAGCATACTAACCATACTCTTTGcaatcaAGGACTATATACTGTAGCTCCTGCTgGGATgAAAATcCTAATCACATCAAcCTtcttTCTatcaaTCtTcCTTtcctttttcttatcCcTtCTGACAGcAATTTACTCCTACCTtTAACTcaGACTgGaTAAaaTGAtCTCaTcTtCcaGaGcACCctCTTtAccTTCCtAtTTactCTTTGCcTatcTATccctCCtGCttcCTtGGatACCtCatacAatCaCCccacTCCCcttCcActAGctcCTaAtTAccctggccagtCCtCTaCaagaCtcTCaACTtaAccCACtcTctgttaaAcCAGTcCAAtCCTTCCCTggCaAAtgACTGTtggCTTTgTaTCtCtcTAtCaaCCTCtgcTTAcGTTGCCAcTCCcaTTCCtgCAaAAAaaatCtgggtctttttttACCAACTTAaCctAcCaCcCtcgttTatgAagGAAAAgACCCtTtCcgaaCTTCTAaATATgCAATcAtccTAGCcaACTtCcCCATctCTGATAGGACCAAgAatACCCTAACAGGAcgtgCAAtcCAACTTTTAcGTTCTTAcATTtcCAAcCTCACcTATTcACAcaAGCAATgAAAAGCCCATACAtGGcCCTGTAACtAcGAATACcATCTTAACgtcttggTTTCCAAGCCCCTTTATGCATCCAAtGCAACcTgTTtttaATCAgGCCTGCCcCTGGGGCAcCTACTAacccCCCATCAGTGTAATTACACCCTAcAACTTCAAGCCCCAaCTGATCATAgtAACTTccgAGTcACcCAaACAGCTCctgATTCAGAtgGCTTgTCcgcTTCTCAGggcCcccaAAAATCATCACcTCCTCCCTgCTTaACAAACaGTCcagGTTTTgTAATGGCAAACATACTCCCTGCATGAcCATTCACCCCtgGgactctggaccCCCTGCAGCAgcgCCCCCACCcAcTAgTGAATGccTTCTCATtCCCcTCTTTCAATcACTCTCTctaacgAATgGTTCCTAGTAGATACaAAAcaGtTTTTTgcTCCAATGGGAAAATAGAACAcAgGGAgCCACTCAGTTTgCTCCCAACACCCcTTttCCAGCcaCTCACTgGAGcTACCTTGGCAAGTACTCTAGGAGTATGGGAAAATGAAAACAACAAAcTCACACACCTttgtcagttTTtAACATAcAcAAtcaaCCAGTTCTGTCTACcaaCCAGCCAAGGtATATTCTTctTATGTGGAAcaTCaACCTATATcTGCCTCCCCACTAACTGGACAGGCACCTGCACCTTAGTCTTtCTAAGTCCCAACATTaACATTGCCCCAGGAAATCAGACCcTATCAGTaCCCCTCAAAGCTCAAGTCcaTCAGcaCAGaGCCATACAACTAATACCcccTACTTAtAGGGTTgAGGAATGGCtACTgGCTACAGGAACCaGAATAGCcAGTTTATCTACTTCAtTATCCTACTACCaacaACACACTCTCAAAGGATTTcTCAGACAGTTTGCAAGAAATAACaAAATCTATCCTTACTCTACAATCCCAAATAGACTCTTTGGCAGCAGcattTGACTCTCCAAAACcgCcgAGGCCTAGACCTCCTCACTGCTGAGAAAGGAGGACTCTGCAcCTTCTTAGGGGAAGAGTGTTGTTTTTACACTAACCAgTCAGGGATAGTAcgAGAtgCttCCcGGcaaataaaggaaaagtcttctgaaatcagacatgctgcctagcaTTTACAGGAAAAGGCTTCTGAAATCAGACAAcgCCTTTCAAAcTCTTATACCAAcCTCTGGAGTTGGGCAACATGGCTTCTCCCCTTTCTAGGTCCcgTGgCAGCCATCTTGCTaTTACTcgCCTcacacTtGGGCCCTGTATTTTTAACCTcCTTGTCAAAtTTtGTTTCCTCtAGgAaTcgAGGCCATCAAGCTACAGATGGTCTTcACAAATGGAACCCCgtttgtaagttgttAAATGAGcTCAACTaACAACTTCTACcgAGGACCCCTGGACcgACCcaCTGGcccaaaCCCTTtcaCTGGCCTAgAGAGTTCCCCTCTGGAGGACACTACAACTgCAGGGCCCCTTCTTcaCCCCTATCCAGCAGGAAGTAGCTAGAGcgGTCATtggCCcAATTCCCAACAGCAGTTGaGGGTGTCCTGTTTgtttAGAGGGGGGATTGAGAGGTGAagccagctgggCttcccttgggtcgattggggacttggagaacttttctgtcttgctataaagtattgtaaatacactaaacagccctctgtgtctagctaaatgatttgtaaatgcaccaatcagtgctttgtaaaagctggagctagaatgcaccaatcagtgctctgtgtctagctagaggtttgtaaaagcacccaatcagcactctgtaaaacgcaaccaatcaggagattctactggtaaatagaccaatcagcaatctgtaaaatgggccaatctgcagggcgagagaggggacaaataagggaacaaaaaacggcaaccagactgagcaccccagccagcagctgcaatcggctccccgtccccttccacactgtgggaagctttgttcttttgctcttcacaataaatcttgcTgctgctcactcccacatggcgaaagctttgggtccgtgctacctttaagagctgagtaacactcactgtgaaggtctgcagcttcactcctgaagtcagcgagaccacgaacccactgggaggaatcaacaactccgggtccgatcgacttaccaactgttgaactgactgcgagggtatggggcgggactgctgaggagatccaccaacaggcacggagcgggatccactaggtgaagccagctgggctcctgagtctggtggggacttggagaacttttatgtctagctagaggattatatatgcaccaatcagcactctgtgtctagctcaaggtttgtaaatgcaccaatcagcactctgtatctaaatgatctggtgaggacttggagaacctttatatctatctaagggattgtaaatgcaccaatcagcacgctgtgtgtagctacccaggttagtgaatggaagaaggctgtgtcagcactctgtgtctagctgctctggtggggactttgagaacttttatgtctagctcagggattgtaaatacaccaatgatcactctttgagttcgaagtttgtaaatgaactaaaaaacacactgtaaatatctgcagcttcattcctgaagccagcgagactatgaatctagctggaggaatgaagcacaagggagccagcattggcaacccactcgggtccccttccacactgtggaagctttgctcttttgctctttgcaataaatcttgctactgctcactctttgggtccacgctgcttttatgagctgtaacactcactgtgaagatctgcagcttcactcctgagcccagcaagaccacgagcccaccaggagtaatgaacaactccagatgcactatcttaagagctgtaacactcaccgcgaaggtctgcagcttcactcctgagccagcgagaccacgaacccaccagaaggaagaaactctgaacaca

>herv9_10320_100006_con_gagputein

nklvdpaamsrtlkvmspkqdsliypiapdgcllgpsacttlsilssspllvpllkpsallwsflssssrvslallllpgsgaflvffirifpsinsrlglsalgssglsgarlpflppfrlwgllsgilgsllslagmgslrnpmgilrnsilpihimrsedkrchssnpgdpfppsgygpplhfwgitslqdrgkvpiltgeclglltlffenavvgrlnliflgplcglgggarvqvffnasvratksdlppsslwssrrrktsvaaasvsttipissvqtvlrswgrgcfaaaasvsttisisgvggplgslgggekkqtktcgvfffqMGNTQASTGSPLKCILSHWDQFDPQTLKKrwrlLiFFCTmAWPQYSLSDGEKWPPEGSINYNTILQLDLFCKREGKWSEIPYVQAFFSLKENTQLCKACNLHPTGGPLSLPPYPqSLPIAPLPINDKPPLISPAQKgkEISKEISKGPQkppPGYrLCPLQAVGGGEFGPTwVHVPFSLSDLKQIKADLGKFSDDPDRrYIDgVLQGLGQTFDLTWRDVMLLLDQTLAFNEKNaALAAAzEFGdDTWYyLSQVNDRMTAEERDKFPTGQQAIPSMDPHWDLDSDHGDWShKHLLTCVLEGLRRIRKKPMNYSMMSTITQGKEENPSAFLEwLwEALRKYTPLSPdSLEGQLILKDKFITQSAtdatDdIRRKLQKQALGPEQNLEALLNLATSVFYNRDQEEQAQKEKrDQRKAAALVMALRQTNLGGSERTENGAGQSlPGRACYQCGLQGHFKkkiKDCPmRNKLPPhPCPLCqGNHWKVHCPRgqrfsgpeapnqmiqqqdlgcpgqapahvitltepqvcltiegqeidflldtgmaf

>herv9_10320_100006_con_proputein

gagpcprgqrfpgpeapnqmiqqqdzGapqqqdlgCpGQaPAHVITLTEPwVCLTIEGQEIDFLLDTGaAFSVLISCPGrLSSRSVTIrgsGILGQPVTRYFSHLLSCNWETLLFSHAFLVMPESPtTPLLGRDILAKAGAIIYMNMGNKLPICCPLLEEGINPEVWALEGQFgraKnahpvQIrLkdpttfpyqrqyplrpeahkglqdivkhlkaqglvrkcsspcntfil

>herv9_10320_100006_con_polputein

tvlsavtiqgilgqpvtryfshllscnwetllfshaflvmpesptpllgrdilakagaiiymnmgnklpiccplleeginpevwalegqfgraknahpvqirlkdpttfpyqrqYPLRPEAHKGLQDIVKHLKAQGLVRKCSSPCNTPILGVQKPNGQWRLVQDLRLINEAVipipLYPVVPNPYTLLSQIPEEAEWFTVLDLKDAFFCIPLHSdDSQFLFAFEDPTDHTSQLTWTVLPQGFRDSPHLFGQALAQDLGHFSSPGTLVLQYVDDLLLATSSEASCQQATLDLLNFLANQGYKVSRSKAQLCLQQVKYLGLILARGTRALSKEZIQPILAYPhPKTLKQLwGFLGITGFCrLWIPrYSEIARPLYTLIKlkETQRANTHLVEWEPEAETAFKTLKqlkqasQALVQAPALSLPTGQNFSLYvTERAGIALGVLTQTrGTTPQPVAygpvyLSKEIDVVAKGWPHCLrVVAAVAvLVSEAIKIIQGKDLTVWTTHDVNGILGAKGSLWllgLSDNcLLRYQALLLEGgpVLQIcctCvALNPATFLPEDGEPIEHDCsitQQIIVQTYAtzDDLLEVPLANPDLNLYTaDGSSFVENGIzRAGYAIVSdscfDVTvLESKPLPPGTSAQLAlELVALTrALELGKGlKRINVYTDSKYAYLILHAHAAIWKEREgfFLTSGGTPIKYHKEIMELLHnsaVQKPKEVAViLHCQsigpsHQKGegeilllkaEgnhqadAEAKiaarqnlpleipmegplvrplvwnnplqeikpQySptEtEwglsrghetirlprhsflpsgwltteEgqtgikerqkqKvlipeasqwKilKtlhqtfhmgienthqmakSlftgpnllqtiqqvVKaCevcQrnnpLvhhkaplgeqrnwtgtcighypgedwqldfthmpkskgfqyllvcvdtftnwieafpccktekaqevikvliheiiprfglpqslqsdngpafkaglatitqgisralgiqyhlvisralgityhlhcawrpqSsgkveKaneTLkrhlrkLtqeThlpwpgltlLpmallriznspislimlvlhkmGlspyemlygrpFlTndllldqemanlvkdkitslakyqqnlknnlpegchrekgtelfqpgdlvlvkslpstspsmdslwegpysvilstptavkvagveswihhtnvkfwtppeelagpslqspkisqtqldtpcepwrtcisyfrrkhprlkrllplilkknpfllkkdkztptgpllcpypagsslsshqpnsqqqlgcpvzrgdgpfpggvvvspagllilvgvlsivaal

>herv9_10320_100006_con_envputein

alllylmisssnmisssrapSLpSYLLFaylsLlLPWipashtitpPLPLaPnyLYKTLNLTHSLLNQSNPSLANDCWLciSLStsayvatPipaKngwvFtnLTyhPhYegKdPFqLLNmQSLAnFPISDRTKNTLTGcAIQLLrSYISNLTYYTSNEKPIhgPVTtNTILTFQaPLCiqcnlLsgsLPLGhLlPhqcnytlqLqaptdhsnpfsvtqTalfrwlvsfSgpPkIiTSSLLNKqSRFCNGKHTPCMTIHPWTPCSSaPTTSECLLIPSFNHSLeWFLVDTKrFFLQWENRTQGATQFAPNTPFQPLTGATLASTLGVWENENNkfklTHLFNIHNQFCLPpSQGIFFLCGTSTYICLPTNWTGTCTLVFLSPNINIAPGNQTLSVPLKAQVhQhRAIQLIPLLIGLGMATATGTRIASLSTSLSYYhhTLSKDFSDSLQEITKSILTLQSQIDSLAAVTLQNczGLDLLTAEKGGLCTFLGEECCFYTNQSGIVzDAtwHLQEKASEIRQcLSNSYTNLWSWATWLLPFLGPVAAILLLLAFGPCIFNLLVKFVSSRIEAIKLQMVLQMEPQMSSTNNFYrGPLDzPtGPfTGLkSSPLEDTTTAGPLLhPYPAGSSraviiqiptaigvsclegglrgeagwasgvggdlelfcvalat

**HERVW**

>hervw_10310_100019_con_chaindnarm

tggggtgatcctgagagacaggactagctggatttcctaggccgactaagaatccctaagcctagctgggaaaggtgaccacatccacctttaaacacgggacttgcaacttagctcacacccaaccaatcaggtagtaaagagagctcactaaaatgctaattaggcaaaaacaggaggtaaagaaatagccaaatcatctatcgcctgagagcacagcgggagggacaatgattgggatataaacccaggcattcgagccggcaatggcaaccccctttgggtcccctccctttgtatgggagctctgttttcactctatttcactctattaaatcttgcaactgcacactcttctggtccatgtttgttacggctcgagctgagctttcgctcaccatccaccactgctgtttgccactgtcgcagacctgccactgacttccatccctccggatccggcagggtgtctgctgtgctcctgatccagcgaggcgcccattgctgctcccgattgggctaaaggcttgccattgttcctgcacggctaagtgcccagggttcgtcctaatcgagctgaacactagtcactgggttccacggttctcttccatgacccacggcttctaatagagctataacactcaccacatggcccaagattccattccttggaatccgtgaggccaagaaccccaggtcagagaacacgaggcttgccaccatcttggaagtggcctgccaccatcttggaagcggcccaccaccatcttgggagctctgggagcaaggacccccgacagtaacattttggtgaccacgaagggacctccaaagcggtgagtaatattggaccactttcgcttgctattctgtcctatccttccttagaattggaggaaaataccgggcacctgtcggccagttaaaaacgattagcatggccgccggacttaagactcaggtgtgaggctatctggggaagggctttctaacaacccccaacccttctgggttggggacgttggtctgcctggagccagcttccactttcaattttcttggggaagccaagggctgactagaggcagaaagctgtcgtcccgaactcccggcattagccggttgagatcatggcgcagccagaagtctctattcaacaagtcgcccatgtgtgcgcccctgacctttccttctgacccatacctcctgggtcccaaccacgactttcttgaaagtgtagccccaaaattctccttacctctgaatctacttcctctgatccctgcctcctaggtactaatggttcagactttcatttaacctctagcaagttgtatctccaaagggatctaaggaagctctacgctgcgtccttaggcacctaggctataaacccagggagtcttatccctggtgtccctcctgatttaggtatacagctctcgacatgggcagttatgtgggacccattccccaccacccttgccagggcatactacctatcaacttttattttaccgaatataacatggtcaccaatgaactaatcattcaggaagacacttactagagcacctaattggtagacgggttttcccaagtttgtaatggggaaaaaaaatgagatggtcctcaaagatgtaaacacaagtcattgactaagagagagagagagagagagagagagagagagagagagagagagagagagagagagagagagagagagaggaaatagaaaactgagagagaaagagacctaaagcaaagaaagacagagagacaagagatagagaaagagacaaagagaagaaaaaaagatgtaagagagaggaaaaagagggaagtaagagaaagaaagaaaaagaaaagaaatagaaaaaaaaaaagagtgtgccctattcctttaaaagccagggtaaatttaaaacctataattgataattgaaggtcttctccatgaccctataacactccaatactgctacaagtaaactttgttgtcagtgtaaataagggcgtagcccgaaagcactgaggccactgacaacccatagctttcctatcaaaaatccttaacccagtaacccacggatggaccaaatgcattcaatcggtagcggcaactgctttgctaacagaagaaagtagaaaaataacttttagaggaaacctcattgtgagcacacctcaccagttcagaattattctaagtcaaaaaaaaaaaaacaaagaaagcaaaacttcaggtagcttactaactcaaaaatcttaaagtatggggctattctgttagaaaagaggtaatttaactccaaccactgataattcccttaacccagcagatttcctaacaggggatttaaatcttaattaccatacaaaggtccgaccagacctaggaggaactcccttcaggacaggatgatagatggttcctcccaggtgattaaggaaaaaaaaccacaatgggtattcagtaattgatagggagactcttgtggaagcagagttagaaaaattgcctaataattggtctcctcaaatgtgcgagctgtttgcactcagccaagccttaaagtacttacagaatcaaaaaaatgaagactctatctcaatcctgactcaaaaggttacctacaacctctctgaaatgaatttgcataagaactgttgtttatgtgaaatgcatcttgatgggacaactactgggatgatatgaaatactcaggaatccagcccagctataggactcactcctgagcacaaaggcaatgttgggcacgctggtaaaggaccactagaatccagcagcctggacccctttctttgtggtcaagaaaggcgggaaaaggggtgcaggactgctacatgggtaagcataactaatccgataagcagaggtccatgggtggttatgcaccctggaaaagaataagcattaggaccctagaggacgctctaggactaatgctcatcagaaaatgactaggggtcctggcatcccaaccctatattcttttttcagatgggaAacgttccccccccccccaaggCAAAaatgcccctAagAtgTAttctggagaattgggaccaatttgaccctcagacgctaagaaagaaatgacttatattcttctgcagtaccgcctggccacgatatcctcttcaagggggagaaacctggcctcctgagggaagtataaattataacaccatcttacagctagacctcttttgtagaaaagaaggcaaatggagtgaagtgccatatgtacaaactttcttttcattaagagacaactcgcaattatgtaaaaagtgtgatttatgccctacaggaagccctcagagttctacctccctaccccagcgtccccccgactccttccccaactaataaggacccccccttcaacccaaatggtccaaaaggagatagacaaaggGGTAAAcAAtTgAACCaAAGAGTGCCAaTATTCCCcgATTATGcCCCCTCCAAGcAGTGGGaGGAGGAGAATTCGGCCCAGCCAGAGTGCATGTACCTTTTTCTCTCTCAGACTTAAAGCAAATTAAAATAGACCTAGGTAAATTCTCAGATAACCCTGATGGCTATATTGATGTTTTACAAGGGTTAGGACAATCCTTTGATCTGACATGGAGAGATATAATGTTACTGCTAAATCAGACACTAACCCCAAATGAGAGAAGTGCCgCCATAACTGCAGCCcCGAGAGTTTGGCGATCTCTGGTATCTCAGTCAGaaGTCAATGATAGGATGACAACAGAGGAAAGAGAAtgATTCCCCACAGatgccatGCCAGCAGGCAGTTCCCAGTGTAGACCCTCAcTGGGACACAGAATCAGAACATGGAGATTGGTGCCGCAGACATTTGCTAACTTGCGTGCTAGAAGGACTAAGGAAAACTAGGAAGAAGCgCTATGAATTATTctCAATGATGTCCACTATAACACAGGGAAAGGAAGAAAATCCTACTGCCTTTCTGGAGAGACTAAGGGAGGCATTGAGGAAGCATACCTCTCTctGTCAaCCTGACTCTATTGAAGGCCAACTAATCTTAAAGGATAAgtaaGTTTATCACTCAaGTCAGCTGCAGACATTAGaAAAAAAaCTTCActtccAAAGTCtGCCTTAGGCCCGGAGCAAAACTTAGAAACCCTATTGAACTTGGCAACCTCGGTTTTTTtATAATAGAGATCAGGAGGAGCAGGCgGAAcGGGACAAACGGGATaAAAAAAAaaaaataAGGCCACCGCTTTAaaaaaGTCATGGCCCTCAGGCAAGCGGACTTTGGAGGCTCTGGAAAAGGGAAAAGCTGGGCAAATCGAATGCCTAATaAGGGCTTGCTTCCAGTGcGGgTCTACAAGGACACTTTAAAAAAaGATTGTCCAAGTAGAAATAAGCCGCCCCCcTCGTCCATGCCCCTTATGTCAAGGGAATCcACTGGAAGGCCCACTGCCCCAGGGGATGAAGGTCCTCTGAGTCAGAAGCCACTAACCAGATgGATCCAGCAGCAGGACTGAGGGTGCCCGGGGCAAGCGCCAGCttCCATGCCAaTCACCCTCACAGAGCCCCtgGGTATGCTTGACCATTGAGGGCCAGGtAGGTTAACTGTCTCCTGGACACTGGCGCGGaaatctcagtaacCCTTCTCAGTCTTACTCTCCTGTCCCGGACAACTGTCCTCCAGATCTGTCACTATCCGAGGGGgTCCTAGGACAGCCAGTCACTAGATACTTCTCCCAGCCACTAAGTTGTGACTGGGGAACTTTACTCTTTTCACATGCTTTTCTAATTATGCCTGAAAGCCCCACTCCCTTGcTTAGGGAGAGACATTCTAGCAAAAGCAGGGGCCATTcATACACCTGAACATAGGAGAAGGAACACCCcgTTTGTTGTCCCCTGCTTGAGGAAGGAATTAATCCTGAAGTCTGGGCAACAGAAGGACAATATGGAtGAGCAAAGAATGCCCGTCCTGTTCAAGTTAAACTAaaccctAAGGATTCCGCCTCCTTTCCCTACCAAAGGCAGTACCCCCTTAGACCCgAGGCCCAACAAGGACTCCAAAAGATTGTTAAGGACCTAAAAGCCCAAGGCCTAGTAAAACCATGCAATAGCCCCTGCAATACTCCAATTTTAGGAGTACAGAAACCCAACGGACAGTGGAGGTTAGTGCAAGATCTCAGGATTATCAATGAGGCtGTTGTCCCTCTATACCCAGCTGTACCTAACCCTTATACTCTctGCTTTCCCAAATACCAGAGGAAGCAGAGTGGTTTACAGTCCTGGACCTTAAGGATGCCTTTTTCTGCATCCCTGTACATCCTGACTCTCAATTCTTGTTTGCCTTTGAAGATCCTTCgAACCCAtctgagttgaaACGTCTCAACTCACCTGGACTGTTTTACCCCAcAGGGTTCAGGGATAaaGCCCCCATCTATTTGGCCAGGCActcaagactaTTAGCCCAAGACTTGAGCCAGTTCTCATACCTGGACACTCTTGTCCTTCGGTACGTGGATGATTTagACTTTTAGCCaCCCGTTCAGAAACCTTGTGCCATCAAGCCACCCAAGCGagaatgtataaattttttatttaccattaaaatgCTCTTAAATTTCCTCGCtACCTGTGGCTACAAGGTTTCCAAACCAAAGGCTCAGCTCTGCTCACAGCAGGTTAAATACTTAGGGCTAAAATTATCCAAAGGCACCAGGGCCCTCAGTGAGGAATGTATCCAGCCTATACTGGCTTATCCTCATCCCAAAACCCTAAAGCAACTAAGAGGGTTCCTTGGCATAACAGGTTTCTGCCGAATATGGATctTCCCAGGTACGGCGAAATAGCCAGaCCATTATATACACTAATTAAGGAAACTCAGAAAGCCAATACCCATTTAGTAAGATGGACACCTGAAGCAGAAGCGGCTTTCCAGGCCCTAAAGAAGGCCCTAACCCAAGCCCCAGTGTTAAggaGCTTGCCAACGGGGCAAGACTTTTtCTTTATATGTCACAGAAAAAAaaaaaacaaaaaacaaacaaaaaCAGGAATAGCTCTAGGAGTCCTTACACAGGTCCgAGGGAcGAGCTTGCAACCCGTGGCATACCTGAGTAAGGAAATTGATGTAGTGGCAAAGGGTTGGCCTCATTGTTTACGGGTAGTGGCgGCAGTAGCAGTCTTAGTATCTGAAGCAGTTAAAATAATACAGGGAAGAGATCTTACTGTGTGGACATCTCATGATGTGAAaTGGCATACTCACTGCTAAAGGAGACTTGTGGCTGTCAGACAACCgTTTACTTAAATATCAGGCTCTATTACTTGAAGGGCCAGTGCTGCgACTGCgCACTTGTGCAACTCTTAACCCAGCCACATTTCTTCCAGACAATGAAGAAAAGATAGAACATAACTGTCAACAAGTAATTGCTCAAACCTAcGCCaCTCGAGGGGgACCTTtTAGAGGTTCCCTTGACTGATCCCGACCTCAACTTGTATACTGATGGAAGTTCCTTTGTAGAAAAAgGaactcaaaaaggagggtaaagaggtgatagataataaagaaatattaaaaaataaccccacaccacgagaaattgttcatcaatgatagcaaaaataatacacattagtacaataataaaaagaggagaaaaaagaaaaaatatataaaaaaactaaaaatattattctaaactactccacccctacaacgcaataaaaagaaaaggaaaaaaaatcatatctgaggaaacaactcaaaaaaaacaaaaagaaaataaaagaatattattctttgcagaacaaaaaaaagaagagatagtaatactacactggtgatatcaaaagaaaggaaagggaaaaaaaaaagaaaagcacagcaaagataataaagaaaaaagaagcgcagcaaggcactcccctataaaagaaattataaaagaaccaataatagggtgaaatcctccccgaaaaaaaaccccacaacactacaaagaagaaaaaatagggagaatcacaagaagagataatttcctcctctccggatggctactaactaaagaagaaaaaataatactgctaacagctaaccaatggaaattacttaaaaaccttcatcaaacctttcacttaggcattgatagcaccaataagatagccaaatcattatttactggatcaggccttatcaaaactatcaagcagatagtcagggcctgtgaagtgtgccaaagaaataatcccctgccttatcgctaagctccttcaggagaacaaagaacaggcaattacccaagagaagactggcaactagattttacccacatgccaaaatcacagggatttcagtatctactagtatgggtagatactCTTcgAAAAGcGGGGTATGCAGTGGTCAGTGATAATGGAATACTTGAAAGTAATCCCCTCACTCCAGGAACTAGTGCTCAGCTGGCAGAACTAATAGCCCTCACTcGGGCACTAGAATTAGGAGAAGGAAAAAGGGTAAATATATATAtaCAGACTCTgAAGTATGCTTACCTAGTCCTCCATGCCCAtGCAGCAATATGGAGAGAAAGGGAATTCCTAACTTCcGAGGGAACACCTATCAAACATCAGGAAGCCATTAGGAGATccaccTATTATTGGCTGTACAGAAACCTAAAGAGGTGGCAGTCTTACACTGCcgGGGTCATCAGAAAggaaaggaaagggacaaaaagaaatactgtatggaaggccttacaaaccaaataccttttggccaaccgatagacaactaacttagttgcagaagtcacctacttagctaaaaatcaacaattactaaaaaaataagaaggaatctatgcctgaaaagaaagaaaagaattattccacctttgagagatggtattagccaagtggaaaggaaaggGAAATAGAAGGGAACCgCCAAGCgGATATTGAAGCCAAtcAAGAGCcgCAAGGCAGGACCCTCCATTAGAAATGCTTATAGAAGGgtcttggatacatcacacttgaatcaACCCCTAGTATGGGGTAATCCCCTCcgGGAAACCAAGCCCCAGTACTCAGcAGaAGAAATAGAATGGGGAACCTCAcgAGGACATAGTTTCCTCCCCTCAGGATGGCTAGCCACcgAAGAAGGAAAAATACTTTTGCCTGCAGCTAACCAATgGAAATTACTTAAAACCCTTCACCAAACCTTTCACTTtcactgcAGGCATTGcATAGCACtgtaCCATCAGATGctccccttGCCAAATtATTATTTACTGGACCAGGCCTTTTCAAAAtattgatgccCTATCAtatAGCAGATAGTCAGGGCCTGTgAAGTGcaccttcacTGCCAAAGAAATAATgCCCCTGCACtgcaggccatacatttcaatccctgtatctttaacctccttgttaagtttgtctcttcaagaattaaagctgtaaaactacaaatagttcttcaaatggagcctcagattcagtccatgaataagatctactgtggtagcgctggacttgcctactagccaatagtccaatgttaaaaacatggaaggcaccccctccccaggaaatatcaactgcctaacccctactatgcaccatattcagtaggaagcacttaagagccgtcgtaggccaacctccccaacaacactaggtttttcctgttgaacagggggcactgagagacaggactaactggatttcctcaggccgattatagaatccctaagcctagctgggaaaggtgacagcacctacctttaaacaagggacttgcaacttagctcacacccaaccaatcattcagtaaagagacgctcactaaaatgctaattaggcaaaaacagaaggtaaagaaatagccaattcaatctatcgccttagagcacagcgggagggataacaatggggatataaacccagtcattccagccggcaagtgtaaccccctttaggtctcctccctttctatgggagctctgcttttactctattgaatggtattaaatcttgcaactgcatgctcttctggtccatgtttgtcctagcatctcgagctgagctttacgctatccatacaccactgctgtttccaaccgtcgcagcccaaccgtacccattcttccttttgctgacttcgaaccctcccgatctggaatggtgtctgctatgctcctgatccagagaggcacccattgctactacagattatgctaaaggcttgccattgttcctgcatggctaagtgcctaggttcatcctaatcgagctgaacactagtcactgggttccatagttctcttccatgacccagctttagacttgctaaccgctgaaagagggggatggcttctaatagagctataacactcaccgcatggcccaagattcgcattccttggaatctatgaggccaagaaacccaggtcagagaacaagaggcttgcagccatcttggaaggaatccaacataaagttgagaggcttcaaaaacaatcgaccctggtgcctcctcaaccaatggatgccctggattctccccttcttaggacctctagcagctataatattgttactcctctttggaccctgtatctttaacctccttgttaagtttgtctcttccagaatcgaagctgtaaagctacaaatggttcttcaaatggagccccagatgcagtccatgactaaaatctaccgtggacccctggaccagcctgctagcccatgctccaatgttaatgacatcaaaggcacccctcctgaggaaatctcaactgcacaacccctactatgccccaattcagcaggaagcagttagagtagtcatcggccaacctccccaaaagcacttgggttttcctgttgagaggggggactgagaaacaggactagctggatttactaggctgactaagaatccctaagcctagctgggaaggtgactacatccacctttaaacatggggcttgcaacttagctcacacctgaccaatcaggtagtaaagagagctcactaaaatgctaattaggcaaaaacaggaggtaaagaaatagccaatcatctattgcctgagagcacagtgggagggacaatgatcgggatataaacccaggcattcgagccggcaacggctaccctctttgggtcccctccctttgtatgggagctctgttttcactctattaaattttgtaaatgcacacaattctggtccatatttgttacggctctagctgagctttcgctcgccatccaccactgctgtttgccgccaacgcagacccgccgctgacttccatccctctggatccggcagggtgtccgctgtgctcctgatccagcgaggcacccatgcacggcgaagtgcctgggttcatcctaatcgagctgaataccagtcagtgggttccacggttctcttccatgacccatggcttctaatagagctataacactcaccgcatggcccaagattccattccttggaatctgtgaggccaagaaccccaggtcagagaacacgaggcttgcca

>hervw_10310_100019_con_gagputein

mgmgnvppkakmplrciLenwdqfdpseqtLrkkzliffcsttawpryplqggEtwpPegSinynTiLQldlfcrkeGkwsevPyvqtFfsLrdNsqlCKkcdLCPTGSPQSLpPyPsvPptdggiPsPtNkdPpSTqtVQKEIDKGVNNEPkSAnIPrLCPLQAVGGgkmilgEFGPARVHVPFSLSDLKQIKIDLGKFSDNPDGYIDVLQGLGQSsFDLTWRDIMLLLNQTLTPNERSAAITAArEFGDLWYLSQVNDRMTTEEREzFPTGQQAVPSVDPHWDTESEHGDWCrRHLLTCVLEGLRKTRKKPmNYSMMSTITQGKEENPTAFLERLREALRKHTSLSPDSIEGQLILKDKFITQSAADIRKKLQKSaLGPEQNLETLLNLATSVFYNRDQEEQaEwDKrDKKkkKATALVMALRQaDFGGSGKGKsWANrMPNRACFQCGLQGHFKKDCPsRnKpPPrPCPLCQGNHWKAHCPRGersseseatnqmiqqqd

>hervw_10310_100019_con_proputein

GCpGQaPAHAITLTEPqVCLTIEGQEVNCLLDTGaAFSVLLSsCPGQLSSRSVTIrGVLGQPVTRYFSQPLSCDWGTLLFSHAFLIMPESPTPLLGRDILAKAGAIIHLNIGEGTPVCCPLLEEGINPEVwategqygqaknarpvqvklkdsasfpinpqvplrpeaqqglqkiakd

>hervw_10310_100019_con_polputein

plleeginpevwategqygzAKNAhPVQVKLKDSaSFPYQRQYPLRPeAQQGLQKIVKDLKAQGLVKPCNSPCNTPILGVQKPNGQWRLVQDLRIINEAVVPLYPAVPNPYTLLSQIPEEAEWFTVLDLKDAFFCIPVHPDSQFLFAFEDPSNPTSQLTWTVLPQGFRDSPHLFGQALAQDLSQFSYLDTLVLwYvDDLLLAArSETLCHQATqQALLNFLATCGYKVSKPKAQLCSQQVKYLGLKLSKGTRALSEECIQPILAYPHPKTLKQLRGFLGITGFCrIWIPRYGEIARPLYTLIKETQKANTHLVRWTPEAEAAFQALKKALTQAPVLSLPTGQDFSLYVTEKTGIALGVLTQVqGtSLQPVAYLSKEIDVVAKGgWPHCLwVVAAVAVLVSEAVKIIQGRDLTVWTSHDVNGILTAKGDLWLSDNhLLKYQALLLEGPVLrLrTCATLNPATFLPDNEEKIEHNCQQVIAQTYAaRGDLLEVPLTDPDLNLYTDGSSFVEKGhmpyncLqKAGYAVVSDNGILESNPLTPGTSAQLAELIALTrALELGmsEGKRVNIYTDSKYAYLVLHAHAAIWREREFLTSEGTPIKHQEAIRRLLLAVQKPKEVAVLHCwGHQKGKERwmgEIEGNrQADIEAKRpAARQDPPLEMLIEGgPLVWGNPLqETKPQYSaeEcIEwGTSqGHSFLPSGWLATEEGKILLPAANiQwKLLKTLHQTFHLGIDSTHQMAKLLFTGPGLFKTIKQIVRACEVCQRNNPlpyrqapsgeqrtghypgedwqldfthmpksqgfqyllvwvdtftgwaeafpcrtekaqevikalvheiiprfglpzglqsdnlaglhcRPYIsiPvSltsginlhLlsvSspiSaVKvLqivLQMePQMQSMTKIyhgPLDqpaSpasmVndikaplplrkSqlhmPyyaPiqqeavravissgzemanlvaditsysclqpPQQhlvFllrggterqdzlglpkelfhLdflgrLripkPSlagkvttSweghgacnlahtlqvrsagpigszveswihhelTKmlirqkqevkkganasysfgappepledlacllfkpnleesnrevsllppacflglpvetgcnrtslldflgdlripplagghvhdrlglsaaiilaglsgwvvsslakllildrakgaklglvlialglgaimkiillgvvggllgrsaslriatllaaakipfgggflasiqleevgrvatigphsivvpgggrglswrtswggpvvtiiailicl

>hervw_10310_100019_con_envputein

MALPYHIFLFTVLLPPFTLTAPpPChCmTSSSPYQeFLWRMwlPgNIDAPSYRSLSKGtPaFTAHTHMPHNCYNSATLCMHANTHYWTGKMINPSCPGGLGATVCWTYFThTGMSDGGGVQDQAREKHVKEVISQLTgVHsTlSPyKGLdLSKLHETLRTHTRLVSLFNTTLTGLHEVSAQNPTNCWMCLPLHFRPYISIPVPEQWNNFSTEINTTSVLVGPLVSNLEITHTSNLTCVKFSNTIDTTNSQCIRWVTPPTqIVCLPSGIFFVCGTSAYhCLNGSSeSMCFLSFLVPPMTIYTEQDLYNyVIPKPrNKRVPILPFVIGAGVLgGLGTGIGGITTSTQFYYKLSQELNGDMEwVAnSLVTLQDQLNSLAAVVLQNRRaLDLLTaERGGTCLFLGEECCYYVNQSGIvTEKVKeIqDRIQhRaEELqNTGPWGLlSQWMlWILPFLGPLAAIILLLLFGPCIFNLLVKFVSSRIEAVKLQMvLQMEPQMQSMTKIYRGPLDqPASPcSnVNDIKgTPPEEISTAQPLLcPsSAGSS

**HERV30**

>herv30_10330_2924_bre_chaindnarm

tgaagtaaaaggaatgttaactgcctgttttcctgtgaccagcagaccttatctatgctcccaattccaattctttgtaaacatactttgtaaagtcctgtaagatcctgtctcctttgccatgctgctgcaagttcataaaatagataaaacctaagttgcaattctggttttcctcaagatctaagacatgtcataaatgattaattgtctttgtttctcactctggtaacatcttcccaccacacgtatttccctccttaaagagtttaaaagtcaattgtataatctaactctggctacctgtttgggaccccttccatgctgtggaaactttgtacttccactctgctcaataaggcctacagccttttctctctatcggtccgtgtctctatcacttgccatggtcatcgccactccaattctttggcatggctaggcaagaaccttaagcattacattttggtgagccagccaggagactccaggaaatgcatctagattgtcacatggtgagtatgattggacctctttcgcttgctattatgtcctatccttccttagaattaggaggctaaatactgggcacctgtaggccacttaaaggcaattagcatggccgccagactgaagacatgggtgtcaggctgtctggaaaagggctgtctaacaatccctgacccttcaaggctgggagcattggttggcctggaaccagttccaattcttttgctttccacggtggtcttgaagtacacctgggagtgctcagcagccttcttagtctcccagatatcctggttgagacaatgaccccgccagaggctccccctgcacgggttactgagcgtgagacagccacatcttctgactccttcttcctgggtcctaatgtccaccagctagacttctttcctcatcttgcaatcaaggtgattcctgctaggcaggatcaagattccctatttagaagtcttaaattcttggggtgatgcccagaagatccctgttcatggtgccctccggggtttaggcagatgtcaccatttgatggccacttggaagggcgagtccaccaccatagtgtatggtcccccgcatcaggacaatttaaagacaggtctgtaattttcatgtggatagtaaaagccttagggcatttcatccattgctcccagatagactttcaccttccttgggacctctcaggtacaatctgtggtgcataggcacgggtcttagagacattgaattgttgtttcaaccattcaataattggtattggaaggaagaaaatatagtcagttgggacaaaggatactggtaccaccttgaaatgagggcttactcttttgatggcaagtggggacagaaggctaaagtacagcagctgttctcttggccctggcctagaggacctccaccaccccctttaagcttactaagcctcctgttgctaattcagagatttctccttgaaggacagttttacggccagggccacgtaaattgggccttagcatgcaagcatcagtggtgcccctgacccaggccttgccatcctggaacaggcaggacacattggcagaaggaccacaataaatccaacagtccttgtgccctatttagtggtcaatgactgcatggcaggggcaagggaagtttccattctgccagtaagcatggttgaatctggtagatggagagctcaggaaaagcggccatgagatttgagcataattggacctgactcttaggggacgccctaagggaaaacaaggcacagaactaaccagggctgtgggcatccttgtgtttaaaatttcagattggcaccacaccttcaaaaccagacactcccttaagatgtattctgaataactgggacaaattcgaccctgaaaacttaaaaaagaagtggctgattttcttctgtaccactgcctggccacagtattccttacaaaacagagaaacttggccccctgagggaagtattaattataacgcccttctacaactagatcttttctgtaaacaggaaggtaaatggagtgaagtcccttatgtacaggctttctttgcccttcttgaccatactgtcctgcgccaagcctgaaagctttgcccaaatgacagaggcccacaattgtttccatattcaggacctctctttcagccctactcttctcccttgctgactctcctccatccggccccactaaagtgttaaaggcacactggaaagagaacgtaaactccatgagctaggcacccaaactatgtcccttacaagcagtaggaggagaatttgtgcccacccgtgtacatgtctctttctcactctcatatttaaaacacataaagtcagatttagggaaattcttggatgatcctgataactatatagatgtcctgcaaggattagggcggttctttgatctaacatggagagatatcatgttatttcttaattagaccttaagtcctactgaaaaagaagcagctttaacagcagcttgtcaatgtggggatctgtggtaccttaaccaggtaaacgatcgaatggccctggaggagagggaaaaattccccacagggcagcaggcagtccccactgtagaccctcactgggatactgactcagatcatggagattggagccacaggcatttgctaacttgcattttggaagggttgaggaagactaagaaaaagcctatgaactactcaatgctatccacaattatgcagggaaaaaaggaaaacccctctgcttttctagaaaggctaaaggaggatctaagaaagcacactgccctaactcgaggttctgtagaaagccaattttatttaaaggataaatttatcacccaatcagtggctgacattagaggaaaactccaaaagtctgccttaggcccagaacaaaatttggaggcattattcaacctggcaacctcagtgttctttaacagggaccaagaggaacaggccgaaagggaaaagcgagataagagaaaggctgcagccttagtcatggtcctcagacaggcagaccttggtggctcagagggaatcaaaagaggaacaggccaattgcctagtagggcttgttatcagtgcagtttgcaaggacactttaagaaagattgtccaacaagaaacaaaccaccccctcatccatgtccaatatgtcaaggcaatcactggaaggtgcactgccccagaggatgaaggccctctggaccagaagcacccaaccagatgattcagcaacaggactgaaggtgcccggggtaagcgccagctcatgccatcaccctcaagagccccaggtaagtttgaccactgatggccaagaagtggacttcctcctggacactggtgtggccttctcagttttaaccttctgccctggatgactgtcctcaaagtctgttactatccaaggaatcttaggacagcctgtaaccaggtatttctctcaccttttcagctgcaattgggagacgttgctcttttcacatgcctttcttgttatgccgaaagtcccacacccttgttagggagggacatattagctcaagctggggctattatctacatgaatatggggaaaaattacccatttgttgtcccctacttgaagaaggaatcaaccctgaattgtcgttttggaagaacaattcggaagggcaaaaaaatgcccatccagttcaaatcaggctaaaagaccccaccacttttccttagcaaaggcaatatcccttaaggcctgaagctcataaagaattacaggatattgttagacatttaaaagctcaagtcttggtaagaaaatgcagcagtctttgcaacaccccaatcctaggaatacaaaaaccaaatggtcagtggagattagtgcaagacatcagaatcatcaatgaggcagtaattcctttatatcctgctgtacccaacccctatacactgctctctgagatatcagaggaagcagaatggttcactgttctggacctcaaagatgccttcttctgcattcccctgcacgctgactcccagttcctctttgactttgaggatcctacagaccacatgttccagcttacatggacggtcttgccccaagggtttagagatagccctcatctgtttggtcaggcattggcccaagacctaggccaattctcaagtccaggcactctagtcctccaatacatgggtgatgtatttctggctatcagtttggaagcctcatgtcagcaggctactctagatctcttaaactttctagctaatcgagagtacaaagtgtctaggacaaaggcccaggtctgtctacaacaagttaaatatctaggcctagtccttgccaaaggaactagagcccttagcaaagagctattcagcctatactgtcgtatcctcaccccaaaacattgaaacagttgtggggattccttggaatcactggcttttgctgactgtggattcctggatacagtgaaatggtgagaccactctatacgctgataaaggagacttagaaggcaaatacccatctagtagaatgggagtcagaggtggaaacagccttcaaaactttaaagcaggccctggtacaagcttcagccctgagccttcccacaggacaaaatttatccttttatgtcaccgagagagcaggaatagctcttggagttcttactcagactcgtgggacagccccacaaccagtggcatacctaagtaaggaagttgatatagtagccaaaggctggcctcactgtttatgagtggttgcagcagtagtcatcttagtgtcagaggctattaaaataatacaaggaaaggatctcactgtctggactactcatgatttaagtggcatattaaatgctaaaggaagtttatggctctcagataactacctacttaaataccagcactactccttgaggaaccagtatttcaaatacgcacgtgtgcagccctcaaccctgccacttttctcccagaggatgaggaaccaattgagcatgactgccaacaaattatagctcagccttatgccacccaaaaagatcttttagaagtacccttaactaaccctgaccttaacctgtactccgatggaagttcatttgtagaaaatggggtacgaaaggcaggctatgccatagttagtgatgcagcagtacttgaaagtaagcctctttccccagggaccagtgctcagttagcagaaccaatggcgcttacccgagccttagaactgggagaagggaaaagaataaatgtgtacacagatagcaagtatgcttatctagtcctacaggcatatgctacaatatggaaagaaagggagttcctaacctctggaggaacacccattaagtaccacagagaaatcacggagttattgcacacagtgcaaaaacctaaggaggtggcagtcttatactgccgaggacattagaaaggtgaaggagaagaagcagaaggaaaccgccaagcagacacttaggacaaaattgctgccaggtaggactttccttcagaaatgcccatggaagggcccctggtatggagcaaccccctccaggaggttaagccccagtatttcccaactgaaacagaaagggactttcacaaggacatcgttttctcccctcggagtggctaacaacagaggaaggaaaggtgctcatacccgaagccagccagtggaaaatacttagaatcctccaccaaatttttcatatgggtattgaaagaacccataagatggccacatccctatttacagggccaaacctcctcaaaaccatctggcaagtagtcaaagcctgtgaagtgtgccaaaagaataaccccttggcctactgtaaggcctctccaggaggacaaagaacaggacattatcctggagaggactggcagttagattttacccatatggcaaagtcaaaaggatgttaatacttattggtctgtgttggtacctttacaaattgggtggaagccttaccttgtagaacagagaaggcccaagaagtggttaaagtcttagttcatgaaataattcctagactttgacttccccaaagcttacaaagtgacaacggtccagcttttaaagctataataactcaaggaatttccaaggcactaggaatacaatatcaccttcactgtgcctggaggccacaatcctcagtgaaagccaaaaagacgaatgaaacactcaagaggcatttgagaaagctagcacaagaaactcatctcccatggcccactctcttgcccattgccttattaagaattgaaaaatcccctcacagaatggggctcagtccgtatgaaatgctgtatggacagacttttctcacaaatgacttcctgctcaatcaggaaatggccaatttagtcaaagatagaacttctctggcaaaatatcaacaaaaccttaaaactttaccgaaaagttgtgacagggaaaaaggaacagtgttgtttccaccaggagatctagtattggtcaagtctctcccctctacctctccatctatagatcccttatgggagggactatactcagtaatcctctctacccctactacagttaaagtggcaggactggaatcctggattcaccacacctgagttaaaccttagacacctcctgaggaacttagaggatcatcatctcaggagtcacaaggtcagccagaccagcctcgatacacctgtcagccactagaggacctgcaactcctatttcggaaggaaacatctcagaccagaaaaactcctgcagttaatcctgaagaggaacttctccctacataaaagaggataagtaaaaaaacctacatgatctttgacatctctccttgctctctttaatggaatccttctactgttttgttacatttttaagcagtatactaactatactctttgcagtaggattatacactgtagctccagctgggacaaaaatcttaatcacatcaacctttcttctattgtccttccttctaacagcaatttactcctttttccctcctctttcctatgaccattccacctacaacaggtcatgactcctcttaggcttcctgccatcctccggcttcctgccatcctcttcatactcatgcccctttctccaactaccacgcaacccccatgtcagtgtgcctcccttggaggagtcagctggcattctctcagaaactcttggggattaggtagccccttccgagcacccgcatcttttgccacatatacttacatgagaaaagaatgttataaaactgcttctctctgctctcacaaaggccatacacatcaccaaggaaaaatgatccgaggtgactaccctgagaaatggggggccaatgcttgttggacatgttatacccatataggtatgtctgacagaagaggcatccaagatgaggctaaagaatggcatactcaacaagtaattaaaaacttagtccagctctcctgtactcccagtccatacaagaaggtagacctttccaggctacaagaaacacttaactctcattcttgtctctggaggctgtttaacggtatctctttcaggaatactagaggcctttcctagtaatccaaccaactgttggatgtgtctccccttgtgtattcaaccatatgtcccagtccctgtccccagagagtggaacttatccgccccattcctaaacaccaccaaattaatcggtcccatagtcaccgatttaccagtcacacaggcctcaaatctcacatgcataaacttcagcatgactctcaataagtgcacctccaatgtcagtcctgggtatcagtaacctcaggtttcacctgtctaacttcaggcatctttttcatctttgataacacagcctatcaatgcctaaatggcactccaaaagaactatgctttccctcctttctagcgcctcccatgtccatatatactgaacaagagttacaaagtctccttacaccccaatcctgccacacacaagcccttgttgtcccttttattgtgggagctggaacactgggcagtcttggaactggaattggaggcataacctcctccacctaattctattataaatgatcatgagaattaaatgatgtcatggaacaagttgccaactccctagtggcccttcaatgccgacttaattctctagcttcagtagtcctccaaaaccgaagagccctagtcctattaacagctgaaaaaggaggaacctgcctcttcttaggagaagaatgttgctatttcattaaccattcaggaatcattattgaaaaggtcaaaaaaaaataacagaatggatataaagtaggaaaaaggagcttgaacactcaggaccctggaatatgtttaaccaatggataccttggctcttcccctttctaggccctgggacagccatcctactattactcacctttgggccttgatttttaacctccttgtcaaatttctttcctccaggatcgaggccatcaagctacaactggtcttacaaatggaacctcaaatgagctcaacccatggcttctactgaggacccctggattgacccgctggtccctcatctagcctagaaagttcccctctggaggacaccacaattgcagggccccttctttgcccctaaccagcaggaagtagccagaaccaccactgcccagttcccaacagcagttggggtgtctgtttagaggggggactgagaggagaggctagctgggcttcctgggttgagtaggggcttagaaagctgtgaaactcactcatttcctacatcaggacttgcttcagtcctggatgaataatattgaagatatatgcttaaactattcctaacatcaggatttgtgcatgtgttgtcttccccaagaaagctataaacaatgaaaattttgctgtaagtttccctgtgccctgtctgcctctctccctttcctctaccccaaaactaaagtaaaaggaatgttaactgcccatttttctgtgaccagaagaccttatctatgctcccaattccaattccttataaacatactttgtaaagtcctgtaagatcctgtctcctttgccatgctgctgcaaggtcataaaatagataaaaccgaagttgcaattccagttttcctcaagatctaagacatgtca

>herv30_10330_2924_bre_gagputein

PLRCILnNWDKfDPEnLKKKwLIFFCTTAWPQYsLQNRETWPPEGSINYNaLLQLDLFCkQEGKWSEVPYVQAFFALLDiLSCAKPESfAQmTeAHncFHIQDLSFSPTLLPczLSSIRPhzSVKGTLERERkLHELGTQTCPLQAVGGEFVPTRVHVsFSLSyLKhIKSDLGKFlDDPDnYIDVLQGLGRfFDLTWRDIMLfLNzTLsPtEKEAALTAAcqcGDLWYLNQVNDRMALEEREkfPTGQQAVPtVDPHWDTdSDHGDWsHRHLLTCILEGLRKTkKKPMNYSMLSTIMQGKkENPSAFLERLKEdLRKHTALTrGsVESQfyLKDKFITQSvADIRGKLQKSaLGPEQNLEALFNLATSVffNRdQEEqaErEKRDKRKaAalVMVlrQAdLGGSEGIkRGTGqLPSRACYqCslQGHfKKDCPtRNKPPPhPCPIcQGNhWkvHCPrg

>herv30_10330_2924_bre_proputein

DDsatGLkvpGvSaSschhPqEPqVSLTtdGQeVDFLlDTGvAfSVLTfcPGzLSsKsVTIQGilGAcNQvFLSpfQlQLGdValFtcLscYaEsPtPLLGRDiLaQAgaiIymnmGKnyPfV

>herv30_10330_2924_bre_polputein

PLLEEginPELSfWKNnsEGqknAHPVqIrLKdpTtfpzqRQYPlrPEAhkElqdIvRhLkaQVLVrkCSslcNTPiLgiqKPnGqwRlVQDiRiINeaVIplyPaVPNPYtLLSeiSeeaeWfTVLDLKDAFFCipLHadSQfLFdFEDPtdhMfQLTWTvLPQGFrdSPhLFgqALaqDLgqFssPgtLVLQYmgDVflAiSLEasCQQaTLdLLNfLanreYkVSrtKAQvCLQQVKYLGLvLaKGtRaLsKAiqpILSyPhPKTlkQLwgFLGitGFCzLWIPGysEMvrPLYtLiKEtzkanThLveWesEvetAFktlKQALvqAsALsLPtgqnLsFYVtERAGiAlGVLTQTrGtaPqPVAYLSKevDiVAkGWPhCLzvVAAVvILVseAiKiiqGkdLTVwTtHdLSgIlNaKgslWLSdNyllkYALLLeEpVfqirTcAaLNPATfLPEdEEPieHDCqqIiAqpyATQkDlLEvPLtnPDlnLYsDGSSFveNGvRKAGyAIVsDaaVlesKpLsPGTSAQlAEpmALTrALeLgEGKrINVYTDSkYAylVlqayAtIwKeRefLTSgGtpIKyhrEIteLLhtvqkPKeVAvlyCrGHRkVKekkqkETAKQtLRTkLLPgrTfLqkcPwKgPWYgaTpsrrlSpsIsqLkQKGTfTRTrflPsEwLTtEeGKvLIpeaSqwkilRiLHQifHmGieRthKMAtSlfTGPNLLkTiwQVvkaCevCQkNnplAycKasPGgQRtGHyPGedWqlDFThmaKskGczYLLVcVgTFtnWVEAlPcrTEkAqeVVKvLvhEIiPRlzlPQsLqSDNGPAFkAiitQGiskaLGIqyhLHCAwRPQSSVkakktNeTlKrhLrKLAqEThlpWpTLLPiALLRieksPhRmGLsPYEmLYGqtfLtNdFllnqEMAnlvKDRTSLAkyQQnLkTlPKscdREKGTVlfPPGDlVLVkslpsTSpSidPlWeGlYsViLsTPTtVKVaGlesWIHhtzVKPzTppeeLRgSSSqesqgqpdQpRyTcQPLedLqLlfRketS

>herv30_10330_2924_bre_envputein

LLLfPSSfLzpFHLQQvMTPLRLPAiLrLPAILfILMpLSPTTTQPPCQcASLGGVSwHSLRNSWgLGSPFRAPASFAtYTYMRKeCYkTASLCShKGHTHHqGKmIRGDYPeKWGANAcwTCyTHIgmSDRrGIqDEAkEwHTQQViKNLVqlScTPSPYkKVDLPGYKKHLTLILvSGgCLTVSLSGILEafPSNPTNCWmCLPLcIqPYVpVPVPReWNlSAPfLNTTkLiGpIVtDLpvTqaSNLTcINfSmTLNKHLQCQSWVSVTsGftCLtSGIFFIfdNTAYqCLNgTpKELCFpSFLaPPMsIYTEQELqSLlTPqScHTQAlVvPFiVGAGTLGSLGTGIGGITSSTzFYYKzSzELNDvMEqVAnSLVaLQcrLNSLAsVVLQNRRALvLLTAEKGGTCLFLGEECCYfINHSGIiIEKVKKKzQNgyKVgKrSLNTGPWNmFnQWiPWLfPFLGPGtAILLLLTFGLIFNllvKfLSSrIEAiKLQlVLQMEPQMsSThgfYzgPLDzPAgPsSSlessPlEdttiAgPLLcPzpAGSs

**LTR19**

>ltr19_10350_4074_bre_chaindnarm

tggattacagactgcaactggttaggttaaatattaaaagctaaaaaaaaagccggtgcctatacaaaggccgtcatgtaacaaaagcccatcaagagttttgattaggcctttcctggcaaaacttaaagcatgacaaaataacgaaggaattcttaccaggacccatttaggattaaacaagttttattgagggtctgaagaaactccccaggcctccacaaacaagtttactgggcatctgaaggaactccccaaacctctgtgatttagcagaagacaagataagggtaaccaccccagcatctagacccatttagattaagcttactgaggctccaggagtaggtcttccggactcagaccttagttatagatgaaaagaagttaatcacttatgtctttagatgaatgcacgcttacacgtagacatatagcttagaaggtatataagctctgaaaaactttgtaattttgagttggtctggtgataatttccaggccttctccctgtaaccgatgacagaaataaaaactctctttctgcccagttcatcggcatctcattattgggccacgagaaatagcagcgtgaccctctgtttggtctgggaacaaaatttggagagccagccaggagataaggatagtgctgccttcaatggccggcagcttgcgacgagacagtcttcaggaggatcccagcagctgtggggtgaggtttcccccggggaccctccagagggctggtccatgtgcaaatctgcacatccctttcactactggcgaagaacagaggtcaggagcggagaggctcaaagggtgagttaactggatcatatgccaggagcctattgttttcctatctgggcttgtgaagccatttgtccggtaccaccaagggaagcaatagggctcgttcatacgcctgctttgcattttggtcgagatcaggttttgagttagttttgagtctcttttgcctgactgcactcccccttgaaatctgtctccacttgtttgtgtgtctgtcttgttccttttgataccatgtaaacttgaaaacgggaagtattgggtccattcctgctgggaggcctctgggaaggagaaaggtttttttagaagctcaatagttgagagtcagcttaattaaaagctaccatccaaaatgtgtatgcgtatatatgtgtgtgtgcatgtttgtatttaaaaggccttcatgtttttgtttatttctcacctaggaccttgtttttttgagcaaaagttttttcttcacagttgactgaatcctgttttcttcattaatggctagtacaacagaacctactctgagatttttaagataaatgtaatttagatgctagaaatgtctttgtttgaaaaaaaaattaagtgcactgtaaaagcatcaaatggtctagcctcataataattctccccttttgaaaacccaggattcagtgtggggtctgcccagagctcaaaggtccagttaaaaaataggaaaaggaaggcttatgaatttataaaatatacttctattggcatgactaatacgtgtaggtatttatgtcttgtgtacaccatgtttcactactgaaaaatataaaagagttgtaatttactgactcaaagaaaaaataaaagcacttacaaactttaacagaaaaaaggaaagactagtcaagtgctttttcaagtttatgtgacttaagtaaaatccttaataaataagctagctttaaaaattattggtaaagtaatattagaaatcttaagagttgccagcatacatttttgtttgcatttattgatcaagcaatttcatacttatctctgccaaatactataaagtgtcaaaatttggcatggaggctacaaaactataacccagcccaaaacagaatgatctttgcttgtgtagtttttaagaataaaacattaatattggtttaatgaagatagctacatcttgaattatttagtaaaatactgtaacttctgatcttgtggccttaggcagtctagtccacagacatgaagaaagtttgttctgggaaaggactgttatcgtctttgtttcaaagctaaactataaactaagttaataaacaagaatagcttgtagattagacgcaaaatggagtcaggtcagatcattttcactgtctcagttataattttgcaatggcggcttcaaaactttaaatgatgactatcgtagttttcataaataatccaggtaaacgattaaaataattagataaatgcaattggataaatacttgtaaataacttgtcaaaatttagaatctaaaattatattaaacaacagatatctcattatttgggtattttccaataacaatatattgtaggaaaacattctttctaaagattgtgtcttttttaaagggtaactaatttttgtctaattcaaagcttatttaaagattatatacaaaacaaggtaaaagaaaccagggaataaaagaaatataaagaaagctataaaaataaatagagttttaaagattattggtgaaataaaaatattttcaaaaatgtaagcctttggtctaaattatgcaggtcaaatattacgtttgcgaaatgctttagtccataaactgcttctttgacttaaaaattgttcaatttattttggcgggttaaattctagataaggcctggggacatgtaaaattagccatgccccctagctgtgcaaaaaggtattaaagaaaagagaatttatataagaaaggatctggatcttgtatggtacattcttgtcctaaagtaaaataactgcttgtttaaagagagggatgtttaggacaagtcagaaagtcaagacatgtcagagattgtgtaagtcatgaaagttttttgttttgtgtgaaagatttatgaaagggaatttatgcaagaaatgttgtacaatttaaaggtgattaggcctcctaaatgctttataaaatgctactatggctcttagctgtacaacttgcctgctttacacctaggtaaggcctgggacacatggagttagatgctggaataagtcagaccttatctgcacttctgtctaggtcctaggctccacacctagtacacagttaaaatcccagacttaccaaggttttcaccaaaagtaaaggttgctaagagttaacggtgtaacatgtattttaagactactgaagaaacagtttacatgccaggtgtgtaaggaaagtaaaatatacttttggtaaaagattataaggatgcatgagattgtggatttctgcctagattaaaaggttaaaggattgttttaaattggatacaataaagataaaggtttaagcaagttgtggaaggttaattgtagaggaaattctgtgtgtaaaatattggctaaagttaaaagggaatcatccagttttttctgtaaatcgagcattgaaataaaagcacaatgggattctcttagagcactaacttgctctttaacaaaaattgtaaagggttataaaaagtctataaaaatcttaccttgtggtgaaacattaaaattgggtaaacatgtctataaggatttattaagaattgggtttaacatccatagtacactaatgtaaaggtgaagtttggcttatttggtatgaaaattatacaggaagcattgtcaaatatgaaatggtgtttggctttctttgggttatatttgtgtaaatatgttattggtatgtgttccaaagttatgggagactcctataattctgatatatcttagtgtatgttatcagtaataattataattgttatgttaaattattgtgtgccacagaggtaacagattttcttgtcagttgtgtcgtcaattgtgtctttaactatggctaccctaaacatttttgttatctgtaagcaattgttgtcttgttttcatcctcttttgaaggtggttttataatcagctataaagctctaacaagctgtactaggtgctcttgattgcagatttctgataacttttgagattgtgacatcagaatagaggaaaacgttcaggactcttgaagagctaaaattttcattaatatcaagcaggataggaattaactgcatgaactgaactaagaggagactggagtgattttgttgacattttgcttagaatattgctaatcttttgttttgcttttcaaagtcgaagaaacttttcttttgagctattgacagcttttaacaatttagtatactcccatgaacaaaatttagagcatacttttttctctctacttgattttctccagaatttggaaactgtctgtgagtattcttaagttatggcaatagagttattcgcataagtgtagtaagaatctgttttcttttgtaacaggccacaattggaaaaacgttatttttaccaaggctttgactggaatgatgcgctttcctttaaggaatcaaacctgacttatgaacccaataaagcccttgggaaactgccctcatattttgtgtacacagtccctgtacagggtttctgacctgtagtaagtaaaaaatgtcactttctgacagggcaggaaccctaaggtatcttggaacctcaagaggagaggaattcacccaactcataggtatttgatggtacaaatccatggctgggtttggctttaaaaaggtcttatgtcagattccttctatggaacaaagttccatgaaagccagttcaaaaggcctatgtgaaaaataattattcttgctgcactttatgcaaataatctggccaagtataataaagctaaccagtcctatcatgatttgtcttttaataaaaatgggaaactggagaaagaaaaattgtttcaaaactatagtacacctgttgttaaattctagtgttgcctggtgtttttcagtttttattattttctacagtttggattaaattctaatttttctgactacaagtctgcagataatgttttcaattttttccttttgcttttcctttgtccccatttttcctaactgaaaatcattgaaacctaagctgtgctttcttaaagccctgtgaacggaagactagacaacttacatttcagaagaaaacagcagcaacctatttatatgtattgctgttgcatactattatgtttcatcgggcactgcctctaatccccccaaacagagagcgctactgggaacaaatcgacctcttccaccccagtgctgcgttcagtgccccataacgatgaccctctctcagcaggaagtagccagaaagattacaatgccccatctccctacgattctcatgataaataaatatacaagcatgatagaaatcatgcacaaattgacagtggggattgtagcaggccaggcttcactaatgcaggcctccatcacaactgtttcagtactgactgggtagttaagttaaatattaaaagccagtgcccttatacaaaggctgtaatgtaacaaaagcccatcaagagttttgcttaggcctttcttgggccttaaagcatgacaaaataacgaaggaattcttaacaggacccatttaggattaaacaggttttattggatggctgaagaaatttcccaggcctccacaaacaagtttattggaggtctgaaggaactccccaaacctctgtgatttagcaggagacaagataagggtaatcatcccagcatctagacccatttagattaagtaaacttactgaagctccagaggtaggttttccggactcagaccttagttatagattaaaagaagttaatcacttatgtctttagacgaatgcatgcttacacgtagacatatagcttagaaggtatataagctctgaaaaactttgtaattttgagttggtctggtgataatttccaggccttctccctgtaaccgatgacagaaataaaaactctctttctgcccagttcatcggcatctcgttattgggccacaagaaatagcaggccaaccctcagtttggtccaggaacactatagtgttgctttcacatacattatcattaagaatgacaccaaaccaggtgcttatttaaagaaaggaggatagaataaaaataaaataacgaaaggaggggatgaccccctgtgtatagaatgctaaataccacacagctacacactcccatttgttgcttctagttctagtcaaagactacttgaaactcctagaaagcaataatttccttagacaatccacaatccctgggggcttggaatgtaaatggatgaacaaatgcctgaaggtacctgataaactcaatatttcataacctcaacaggtagacattatctccacagtaagtccagggaaactggcattcagaaaagttaaataatttgatcacagagctagctgggtttcaagcccatgtttccgtgattctaagaaaacttcaatgtatagattctcttctccctcattaccttttccttaccacatcctcccatctccccagcctatatgttaggatcccagaagaaagcagatagtacattagatgattgaggtgatattaataaagggagtatttaggaaggtgtgggtttgaacaaagcaacaaatcatggggcaacaccctgggactgaataaaaacaatgctattacctaggctgaagagaagggagcagttgtcagaaactagacagggccatccaacacgagctgtggcctttggtggagggatgcagccaacttgcagggacccaacaaggaggaaccaggggaaaaaatttactctcctactctttgatctcctgccaaacccaaacagaagccagaggacaaaaatctctgttggtgcattgcagccaggtaagcctcatggggcacagagcataatacccagatgtaaaggggcaagcaggtg

>ltr19_10350_4074_bre_polputein

PkVkvaRvngvtCiLrLLkkqftcqvcKESKIyfWzKiirmhEivdfcLdzkVkgLfzIgynkdkglSKlwKvncrGnsvcKiLakVKesssffckssieikaqWdslralTcsLtkiVKgyKksiKilPcgetLklgKhVykDllrigfNihSTLvkvkfglfgmKiiqeaLSNmkwcLafFglylcKyvIGmcskvmgdsynsdiSzcmLsviiiivmLnycVpqrzQIfLsvVssivsLtmATLnifvicKqLLscfhpLlkVvlzsAIkLzQaVLGaLDcRfLitfEiVtSezrKTfRtLeELkfslissrIGincMteLrgdwsdfvdIllrillifcfAFQsRrnfsfeLLTAfnnLvysheqnLehtfFsLlDflQnletsVsilKLWqzsyshkcSKnLfsfVtGhnwKnVIfTKALTGdaLsfkesnLTyePnkALgKlPsyfvyTvpvQgfzpvvskkcHFlTgqePzgiLePqEErnsPnHRyLmvqiHGwvwLzkgLmsDsfYGtkFhEsQfKrPmzKiiIlAALyAnnlAKiIkLTspimicLliKmGnwrkKNcfkTiVhlllnSsvAwcfsVfiIfQfGLnSnfsDyKsaDnvfnffllLFLcphfStenHznlScAflKpceRktrQlTfqKKtAATylyvllLhTImfhraLPLIPPnREryweqIDLppqccVQcPItmTlsqQEvARKiTmphlPTiLMinKYTsmIeimHKLTvGiSrpgfTnAgLhhNcfStdwvvkLnIksqcpytKaVmTKAhqefclGlSwAlKhDKitkelnRthlGlnrfYWmaeEIsqasTnKfiGGlKeLpkplzFSrrqdkgssqHldpfrlsKLtEaPevGfPdsdLSyrlkeVnhlclzTnAcLhVDizLrrYiSSeklvilswSgdnfqafsLzpmTeiKtlfLpsssAsrYwAtRNsrPtlsLvqEhYsvAftyIiiknDTkPgAyLkkggznKnkItkggdDPLcieaKyhTaThsHllllVLVkdylkllesnnflRQstiPggLEckWmnkcLkVpdkLniszpqqVDiIstVSpgKlAseklNnLItElagfqAhvsvilrklqciDsllPhylfLttSsHlPsLyvRIRRkqivh

**HUERSP1**

>huersp1_10910_3408_bre_chaindnarm

tgatttcatctccaacctaaccaattagcactcctcacttcatgacccctacccaccaaattatccttaaaaacttcagtccccgagttttccaggagactgatttgaataataataaaacttcagtctcccacacaacaagctctgtgtgaataagtctttctttattgcaattcccctgtcttgataaatcagctgtatctaggcagtgggcaaggtgaacccattgggcagttacacatttgggggctcatctgggattgcccttgtggctacctgttaatggttcaatagccccctctggcaaccaatccagaggccagccaagtgaccatctagttctcctggacaggggctgactctactctagtaccatctctaccggtagggcactgctgatctaatgtgcatggatttaattgcaataaagaaatagtcctggggacatgtcctatcccataactgtagccccatagtggagtgtctgtctgtagccacatggtggtgggtctgtctgtatccccactgcagagtgtctgtctgtagttccaccacagagtgtctgtaactgtagtcctatcacaggatgtctgtctatagccccatcatggagtgtctgtaactgtagccgatttggtgagtattctaggaactgccaacacctcctttcttatccaaattttttgagctccttcggggatctctgttggctctttctaattagtaggaagaatcgtgattcaggagatttctcctcagtcagaaagatttgggggagatttctcagttggagaataggcggctagtttggaaggaatactcttggaattcttggttagggatcttggtttggaagaccttttgtctgtcttgtatttttgtgtgtgtgtgtttgtatatgtggaggggatctctaaaggaattgctgacagaagtccagcaggcctaacttggagaacccaagttctggtcacatttggtgagtcctgaagaaagctcaacaggcctgacttgggatgactgtctgctcttcatcttgcccagagaacacctccaaaattcccagtcagaggtcatccctccccaccttgagtggatcaaaggtgacaaggaccaaggggagaaagtttgagtcttgccatgttgatattgggtgctgaccgaggtaactagtgtccgtttgttatgtatattttcctcagctgggaaggaaaatgttaatttggtaactcatgcaacctattgggcagtatcttgcaaaattgaaaagttttgtctatgattccataaaacataatggataattttcttttttgacatgatctgccccccacagctgtggcagagggagcagggtcatcaaaagccactccattcttctggaagctgcagagaaagggaacccagaaacatggtgtgccagcaaaaagggtaagaatttcttaccagccaggcttctggcctctgtgtgtgtgtgtgtgtgtgtgtgtgtgtgtgtgtatacagtaaatgtcatcactgtttgtctcctctgcaagggtttgattaatagaaaaaagaatttgtgagactactcttaggctgtagtgaatctggtgtactttgtggcaggaatttgtctttctgtgtcattctgtcattgagagatgagtatcacaggatagaatgtgggcttaagaaccctatacacctgcttttcacaccagcctagcaggcttgttggttacaaactttgctgcaggtccctgaaaccagtaatggatgaagtttctctctcttgttttatgtccttgagagtttaaccttgtggccatgtggggatactttctcttggtctctgccatctagaggagaggaattttggggttcatatcatagttggccctaaaagcagcagttaaaagcctttgcaagctcgaaattggctgctgtagcctccttctggaaaaagcaatggaaactgctcagtgctgcagctcagtggctaaggctttgtcttttgacaatggtagcccaagttcaattcttggcttaaggaatgagtccattctggtttgttatttgtgttcttttttttgtcatgtattgattcatttcccccctgagtggactgcttctgattttctgtcttgattttttctttctctgaactacctttagggagattctaaatcttggaaaaaagaacaaaaggaagaaaggaaggaaggaaggaaggaaggacagaagaaaggatggaaggaagaaaggaagaaagaaagaaagaaaaagaaagaaagaaaaagaaggaaagaaagaaagaaagagagaaagaaagaaagaaaagaaaagaaagaaagaaagagaagaaactgcttatcatctctgagatagcttatgcatccatggttaagtcataaccttagttaagacttactaattttgtatgggaggttacctgtggtagaattcaaaagccagaaatattggctgtctgtcctggctagagtcacatataagagataaaaaataaattatttaaagaactctatggttaaaaccagcttaactaaaaatgaatattcaagatatatatttatatatgtatatatactatacatatatatttaaaaggcttttatgacttttctctacttggatcttgtttttttttttcctttttttgagaaaaatttcttgtttgtttttcttctcagtctactgaactatttccccagtatgtcttcttgacaccttgatgcccacatgagaggacctaaggtaatttctgagagcctgggactccttgggaaaaacagagaaggtgccacagggcatattttgggagaaatctctgttttcctcatggaaccccaagaagtgtaagagacagtttcctctcaaaatctaaggctctgctctattttgcatcacattacccgacatttttgactttaggaggcatcagaaattactttagtagaggatagatatgtaaagaaatttatggatctgaagatgtatttatggtaaggaaggttataaagaaaagaaatttaacatgagaaaggatcttttacagtaaattccagtcctaaagtagaatgacgaattattgaagaaagaggaaaatataggacaagtcagaaagtccacgtatgtcatagatggcctgtatgagttgtgagataaggttcataaaatgaaaaaaaaaactcacaactgctacatcttcttctgtctctgtatttataagtgttgtgtgtttgatctttatataaaggagctctaattaattggcttaaagaaaatgaaagtacttaaatattttgtcagacaaatagaaactctaatgccttctagttcatgtgactttagtaatctttgggaaataaaaacagttttaaagattattagtaaaataaaaatgtcttcaaaatttagacatttggtctaaattaagtcagagattagatttgttaaatactttaatgtcataaactgtttctttgacttttgaaaactgtgtaatttacatgctttagagtcaatagattctaattaaggcctggggacatgtggagctagccatgtctcctagctgtgctggaaacagtcagactttatctgcagttctgttgtgcatcctagactctgcacctatctaattaaaattgcttacactaaaaagaaaaattatgtgcttttgatacaaagtcataggaatatctttttttcaaaaaactaattttgtctaatttagaaaatttaagaattatattttcttgtaagaaagaaaataaaattgaaagcttaagcaagttatagaaggtttatgaaaaattaactttataaagaattctgccagtaggcaagttgactaaaattaaaagggtatttattatttagttcttccataaattaaatattaaaacaaatgcacactgacatagggccagaatcttggcccatatgtcaaagtaacaggatttttttgaacattgatttgttctttaacaaaaatattataaagttataaaaagtttatgaaaatcttactttatgaacaaactagttaaaactgaatagatttataaaatttaattaaaaaccagctttagcattaaagatacactaatacaaaaataaaatttgggtttctcttttgaaaaaaaattttatagaatattgagagacaataaaagatttttgtttgccttttgagtaaactacaaaaacgggagcggggagggagaagagatagatttcactggcttcatgctgcctttattgggtcttgtttagaaagctgattctctcttctatcagcaagtatggatttttgccttttaaaatttttttgagttaccatttcagctaaatgaataacctgtgatcatattttgtgatatccagtgttttaaacctttaatatttggcaagccttccaagatcatattctaaatttaaaaaaaggtccccaaaagtccaaaagagacatattcagcttacttgatgtattaaaatcatacaggaaacactgtcaaatttaaaatggtgtttaactttctttgagttatattcatataaatatgttaatatgtgttacgaaattaataataagattcctaaaatgctgacatgtctcagtaaatgttattagtcataatttttttaaagttctggggtacatgtgcagggtgtgtaggtttgttacataggcaaatatgtgtcaccatagtttgctgcacctgtcaacccatcatctcactattaagcccagtatgcattagctatttttcctaatactatccctcccttcacctccccctaacaggccccagagtgtgttgctctcctccctgcagccatgtgttctcactgttcagctcccacttgcaagggagaacatgtggtgtttggttttctgttcctgtgttagtttgctgagaataatggcttccagctccatccatgtccctgcaaaggacatgatctcatttctttttacagctgcatactattccatggtatatatgtacctcattttctttatccagtctatcattgatggccatttgggttgattctctgtctttgctattatgaatagtgctgcaatgaacatacacatgcatgttatctttataatagaatgatgtatattcctttgcgtatatgctcagtaacgggattgctgtgccaaatgatatttctggttctagatctttgaggaattgccactctgtcctccataatggttgaactaatttacattcccaccaacagtgtaaaagtgtttctatttctctgcaacctctacagcatctgttgttatttgacttttcaataattgccattctgactggtatgagatggtatctcattgtaataaagacataccccaaactgagtaatttataaaggaaagagatttaattcactcacaacccagcatggctggagaggcctgaggaaatttacaattatggaagaaggcacctcttcacaaggtagcaggagagagaatgagcaccaagtgaacagagaagccccttataaaaccagcagatctcatgagaactccctcactattatgagaacagtaaggtagaaacagccctcatgtttcaattatctccacctgatcattcccttgacctatagggattattacaattcaaggtgagatttgggggggcacagagaaacaaaccatatcattctgcccctcagccctcccaaatcttatgttatcacatttcaaaacaaaatcatgcccttccaacagtcccccaaaatcttaactcattcaagtattcacccaaaagtccaagtccaaagtctcatctgagacaaggcacatcccttctgcctatgagcctataaaatcaaaagcaaattagttacttcctagatacaatgggggtacaggcattggataaatacacccattccaaatggaaggaattggccacaacaaaggggctacaggccccatgcaagtccgaaatccagtggggcaatcattaaaccttaaagtttcaaaatgatctcatttgactccatgtcttacatccaggtcacactgatgcaagaggtgggctcccacagccttgggaagctccacctctgtggctttgcagagtatagcccacctcctggctgctttcatgggctggcattgagtgtggcttttccaggcctatacatccatgcatttccttatatcctctgaaatctatgtggaggttttcaaacttcaattcttgctttatgtgtccccacaggaccaacaccacatgaaatttgccaaggcttgggccttgcaccctctgaagcaatgccctgagctatactttagccaattttggctatggctggagcagctggaaaacaggacaccaagtactgaggtttcacacagcagggggcccttgacccagcccagagaaccatgtttctctgctaggcctccgggcctgtgatgggaggggctgccataaaggtgtctgacatgtcctggaggcatttcctccattgttttggcaattagcatttggcttctcaatacttacaccaatttctgcatcctgcttgaatttcttctcagaaaataggtttttcttttctactgcatcatcaggttgcaaattttccaaacttttatgctctgtcacctcatgaaagctttgctgcatagacatttcttccaccagataccctaaattatgtctctcaagtccaaagtatcacagttctgtagggcaggggcaaaaagtaaccagtttctttgttaaagcatagcaagagtcacctttgctccagttcccaagaagttcctaatctccatctgagaccagctcaggctggaattcattgtccatccatattactatcagcattttggtcaaaaccattcaacaagtctctaggaagttccaaacattcctacattttcctgtcttcttctgaaccctctaaactgttacaacctctgcctgttacccacttccaaagtcactttcacatttttgcatatctttacagcagtgccccactcctggtagcaatttgctgtatcagaccattctcacaccactaataaagacatacccaagactgggtaacttataaaggaaaaaggtttaattgactcacagtccagcatggctggggaggcgtcaggaaatttacaatcatgatggaaggcacctcttcatagggaagcaggagagtatgagcaccaagcaaaggggaaaacccctttcaaaaccatcagatcttctgagaattccctcactatcatgagaacagcatggcagaaactgcctccatgattcaattatctccacctggtcccgcccttgacacatagggattattacaattcaaggcgagtatggaagtgtgttttccaacttggttatgttcaccccatgtctttcaggtaactcaatcagtcataagttcgatctttttacataatctcatatttctcagaggttttgctcctccttctctttcttttttctctaatcttgtctgcctgccttatttcagcaggataatcttcaagttctgatattctttcttttgcttggtctattcagctattgatacttgtgtttgtattgtgaagttctcatcttgtgttttccagctccatcaggtcatttatgtttctctctaaactggttattctggtgaacaactcctgtaatgttttatcttggttcttagcttctttgaaattagttagaacatacttctttagctcagcaaagttcatttttgcccacttctgaagcctacttctgtcaattcatccatctcagcctcagcccagatctgtgcccttgctgaagaagtgttgacataatttggaggagaagaagcactctggctttttgagttttcatgtttttgcattgattcttcctcaactttgtgggtttatctaccttcaatctttaaagtttctgagctttggctggtgagtttgtggggccttttacattgatgttgttgttgttgctgctttctgtttatttttcttttagcagttaggcctctcttctatagggctgctgtggtttcctggggttccactccagactttattcacttgagtccctcctgtacctggtggtatcaccagtggaggctgcaacacagcaaagatggttgcctgctccttcttctgggaactctgtcccagaggggcactgacctgatgccaactagaggcttttgtatgaggtgtctggagacccctgttgggagttctcacccagtcaggggaaatgggatcagggacctgcttaaataggtagtctggctgccccttggcagagcaggtgtgctgtgcttggggggatctccctcttccagactgtcttccagactgtcctgagtctccagagcccacaggcagaaaagactgagaatggtgattcgtggataccactgccacctctccccctaatggctccatcagattttgatagcccctcccccaattacaattcttatgttaaattgttgtgtgtcacagagatgaccagatttcttttttaattgcatttttaactatggtcgtcctgagaattttgtcacccacagataattgttgtcttgttttgattcttttcaaaaggtggtttataatctgatagaaaactctgatgggtactcttgaatacaggtctctgataactttagaaattgtgctattggaatagagaaaaaaaacttctaagactctcatggagagctgatatgttaaacattgctaatccttttatttttcagaattaagagaacttttttccttttgagctatttacaacttttagcgattgagtaaagtatactcttgtaaacaaagctcagagcatatcattttctctcttacctgatttctccagaatttagaaactatttgtgagtatactcaacttatggcagtatagttattttcataagtgtaataagaatctgttttcttttgtaacagaacacaattggagatgccagttattttaccaaagatttgactggaatggtgtgcttttttttttttcttaggaatcaaacttgacttgtagagccaataaaaccccattgggcccattgggctaactggcctcatacctgtacagggttcatggcccaaggtaggtaaagaatgtcactttcttacagacccaggagcccccacgttatcctgagacctcaagacaagaagaatttacccaactaaactaggtattcgatgtcacaaatttgcaactaggctcaaggctttaaagagtgtaatctggtgtaacctgatattccttatggaacaaactttcagcaaagccaattttaaaaagtttaagattaattctacaatttgtatggaaaataattattcctaatgcactttatgcaaataatcaggccaagcataataaaactaagatttattttgcaagcaaacttgtcctactatgatttgtgtttaataaaagtggggactggagagagaaaaaatgtgtttcaaaaaactatagtatatctgttgttagttgttcttgaatttttattattttctatagtttgaactaaatcctgaattctttgtgggctgcaagtccccaaactaacactctcagatttttgtttcacttttctgacttggactcaatgaaatggctactaccttttacctgaggccctgaaaactgaagcttattctttgtaatacaggcaataaaaacgtgtcagattgccaccaccttcctcctctataacttaagaggcttagcccatttgcaatgaacctcctgatacataagatagctgttgagctgaactgatctagtctcaggactaggcaactgacaaaaaaagatttggggcagtacatttaaattcgctctgtcctgtctaccccaatctgtctaacaacctctgacccaaatctctctctgttagtaaccccatgtcgaatttgttctccaagctattcacctggatcccccagagtttaagatcaattctccaaaacttctgaagctaaggctttcagtctttatcctgggactcattatttaccttatagttcactgttctttgctaaaactatagatgagaatactaacgcccttgtcatgcaagctttgaaaccttaatgaggcacccaagagtatgtgcaaacagctgcaaagcagttccactcctcttaccttggggtcaacatctatccccctgtcagcaggaaaaggttagagcagttttcatttttcccaacttcgtagcccacacttgaagaataaggtgttataaatccaaagggagggattgaaaccaccttcgcaaaaatcataactgagaaaattattacagtgaaagagatctgacctaaccaactccatcttgctttaacctccaggctgtcctaggaatatgccagactaactttgggaggaatttagattataaattagctttgaaacaaagatgattaacagccctttcccgaaacaaaccgcttcctgcctgggaactagactgcctttccaggactaacaaattagccacaacattagaaattatggtttaggacttttgcagctggaggctgcaagattctaagcctaccaaattgctcctggcaataacatcactattgtaaaacctcagatcagtgtttgtgatattttgcagaccctgcattctgatgcacagctgacactgcccagacctgtaatctggctcaaacagttctgcaaccctacccaggaatagaagacagcaagaaaaactcactccaacttgaccgatcagcactccccacttcgtgagcccctacccaccaaattatccttaaaaactttagtccccgagtttttcaggagactgatttgattaataataaaacttctgtatcctgcacagctggctctgtgtgagtaagtctttctctattgcaattaccctttcttgataaatcggctctgtctaggcagcaagcaaggtgaacctattgggcagttaca

>huersp1_10910_3408_bre_polputein

AhmrGPkvisesLgLLEkqrRcHrayfGrnLcFPhgTprsVRdsfLsKSkAllyfAshYPtfLtlggIRnyfsrGzIcKeiygsedvfmVrKviKkRnLhEKgsfTVNSSPkVeDeLLkkEenigqvRkstyvIdGLyeLzdKvhkmkkktHncyIffcLciykccVfDLyikELzLiGlKKmkvLKYfVrQIETlmpsSscDfsnlWEiKTVLKIisKikmsskFrhLvzIksEirfvKyFNvincfFDfzKLcnlhALesIdsnzgLgtcGAshVszLcWKqsdficssvVhprlcTyLikIAyTKKkNyVlLiqShEyLffkkliLSNlenlriifsckkenkIeSLskLzkVyEKLtLzrILpvGkLTkLkGyLlfsSSINzilkqmhtdIGpEsWPicqSnRifLnIdLffnKniiKLzkVYeSYFmnklVktEzIYKizLkTSfsIKDtLIqKznLgfsfekKfyRilRdNkRflfAfzVnykngSgegEeIdfTgFmlplLgLVrKLilsSisKYGfLPFKiFLSyHFSzMnnLzSYFvIssvLnLniWqAFqdHILnLkkGPQkSkrdiFSlLDvLksyrKHCqizngvzlSlsyihiNmlIcvTKLiiRfLkczhVsVNvisHnfFkvLGymcrVcrFvTzAnmcHhSLlHLsthhLTIkPsmhzlffliLslPsPpPnRPqSVLlSSLqPcvlTVqLPlArenmwcLVfcscVsllrImAssSiHVPAkDmISflftAayySmVymyLiffIqsiiDghlg

**HUERSP2**

>huersp2_10920_100030_con_chaindnarm

tgaaacccacagccgaaagtgagaacatctatccctgtttgcctgatatctcccgattggttctttctgaataaagtctttttaccaattgaatattgccagcttgacagcagtgcttcagagaagagtctggacggagatggctagactacaggggaagaatatcttcctgccccatccccctttcagctccccttcccgctgaaagctgctttcatcagcaataaaatgctccgagttaaggaacttactaatttttcatgcaacctcattcctcctagacaacagataagaacacgggtgtaggtgtaaaaggctgtcacagtgatcattgactgagctgttaacacacaagcaatctgacgacaggaaagatgaaacagcactcagtggaagaagctgtttagagcttccaggctcttGagtagatccagtctagatgctgcgccagattcacatggagttctgcacctgctaatagCcaaaagcagtcgctaaaattcctgcacacactcacttacactctctgtcctatgaggggttgagagctgcaggccaagtaAatgagGcacccatattgcaagcccgttgaaggagtaacagggaaatagctgtattaccactGgaCccaaataagagtcctcaaaagGGcagagtaAaagttcatgcTgcaacatatggcctttcagggtttacttcccaagagtgattatacgcAgActTtccccataggtcagatcctatactcaaactttccgttgatttaagcaaaaaatcaagttgcagaGgaCAggatGcaActctaCCacctatctctttctttcaagtgaaaggaatgttggCttTgtttCccttCacagagGtctagccatcGggtgGgaccaGaAtAaaGtcctggggcaacTgAAGgcaTctgGccaatgcCaCTccttggTgtTgccagaAGGcCcCtaaaCTGgAttccaTccctgAtaacctataaatgttttGcCcAAgaccCCcaGatttttCtatggtaTcTtTcctTttcTTCTttcAtgGTTtGAAATGgCTCtTATCtCTtCcTTTATaaTGtTAAGgGTTTTGCTgCAaACTGCaGAAATGtTAcTAagTAgAaTgAgCAtTTGGCcCAGcCAtCAgatatacaatatagtaaaactgaaagaaaaattGCAATTCAGAaCAATgtgATttCcGttTgTTCtTAGAaGTgCCAtCCCcACgCCcaacCCCAACAGCtcCAGactCatgCaGcaCgTaGtcgtacCtcctttcataCCCtCccCTCCTcGCTgggGCACcTGGGCATgTCCACaGcATGCacatGtcGtgCCcAgcacctacgtatacaGgGCaGGAaAaAgCTgTggCTGCaaCCaaGgcCcCAgggtAgccttggggacaCAGggacCCtaTtcagCcagctGaccAGcAcTTcCaACtTaCcgaactCTgttacCacAgaaCcAaAGAggcTtTaaTCcgctggactaCtaAGGagtccAgctcAGTccAAAcaaaaggaaGGaTGCagTaattaaaggaaCCCaTTTgCAgtGAccGCaaggGGctcTtcCCcCtcTTAAGctgtttttttttttTtTtCTttCtTTTTCaAtgtGAcAGGGTTcttttcatcCtaCCTCaaCACTcTGcTTaTgaTagggaaGcgAataGAGgagTgacCcCaaCTggCtgaTAACTGCAAaTTtGacAaggcccaTctggGaCTTAATCTAAAtgaAtcCAtGCAcCCcccTGAaACAccTTTTTatcccaaactcacagtCccAAACTcgATTccAAgCTTcagGtTGAAGCCCTAGAAaggAAAACCaAgaTCTGAGGGATCCAAagccAgaaGCAAcAGGCACAGTAtAAAtGGGCAGGACtaATtCCTGCtgAtTAAGCCcCtcaCtgtTCATGGAaGGAGGCCATGCatttaTCCATGGCATAgATgAaGCCCAGGgaAACCCAaAGGTTGCtGaCAGtAgGGgggAtGgAGgGCATAGGTgAGtGcagAtAATTCCTAacTCTCTAGGCCCTCCCTGCTTCATGGGTacagGCcGCAttgGcACCcATGGaTGGtGtCtAtCtaAGgtTGCcGGGcCTaGGGGATAAAAaaaaaaaGAtgGAAGAgAAAaggAGGAaGcCTtCTTTCTCTcttCatCaCACCccaAGTttTTtCTGaaaGaAGGAAaGGAAaTGAGGGAcACCtaTtTCtCTgTCTTTtatAATGGGtaAcCAGCTatcttcAtCAcCccCAGtCTatACTCCTCTGGAGTtgctGtATCCTGAAtCACTGGGACTGCTTTGACgCCTCAGACTCTGGAGgAAAAAaaaaTGCCTCATAGCCCTtTGCACAAAGGTTTGGCcaAATTATGAtcTgcAGGaAGGACTGGCtTGGCCTCAGGAAGGAACCATTcATTTCAATAaCCATCttGCAGTTGGAcCTTTTCTGTAAATGTGAGGgtgaCAgaTgGTCTGAGgCtcCATATaTGCAGGCTTTgCTaTaCCTTGCAAGgtAAtCcAGACCTTTGCtGACAGTGTAGGATTGaTCCAGcCCTcCTGtTTGCCATCTCAGGaaAgGCTGCAAggGgcaAATCCCAgGGAACTAaAGAaACaAAtCCCAGAGgCaCCtCcAGCAGgGGAGCCAGCTCCCTCaaGCCCTGTTtCTCCgGGTCCACCCCaTCCTCCctATCCAGcTTtTCTcTCTCACTTGCCCCCTCCTAGAAATCCTCACtCTAgACAAGCCCCAGTCTCAcTccTGCCCcTcCAACAGATGCCtGGTGAATaTGaCCCCAGTAAGgTCCAGGTtCCCTTCTCTCTACAGGACTTAAgGCAAATTAAGGgaGgAtCtTgGCAAGTTTTCAGATGACCCTGACAGgTATATAGAGGCTTTCCAGAATTTAACCCAAGTATTTGAACTCTCCTGGAAAGAcaTTATGTTACTTTTGAATCAaACCCTaACTaaCACTGAGAAGCAGgCCgCTCTgCAAGTgaCAGAttttGagATTTGGGGATGAGCTTTGTATCACATATAGTgTCAGGGAAGGgGttGAACtTTATCCAAtTGGAAGAGAAGCAGTACCAtTGgATGACCCTAaATGGGATCCgCAATGAtgAaATGGGAGAaTgGAAGAGGAgACACTTTcAtGGTGTGCATAATGGAGGGCTTACgTAGGACTAGAACTAAGCCTCTCAATTATACCAAgCTATCCATGAtaGACCAGGGATTTGATGAGAATCCtACTGCCTTCCTGGgAAAGGCTAAGAGaGGCCTTGGTAAAGCACACCTCTCTATCTcCTGATTCaGTCaAGGGACAACTAATCCTAAAgGATAAtATTTATTACTCAGGcaGCCCCTGATATcAGGAGGAAGCTGCAgAAACaGGCCcTgGGACCAGATAGTACtTTAccGAGAACCTCCTGAAAtagtactttaGTGGCCACCTtGGTCTTTTAtAATAGgGATAGGGAGGtcgCagaAGAgAGAGAGaggAaACACAgGAAAtAGGCAGAGGCTTtAATgGccaCCATGcAtAGCcCAcaAAACcCCAGaATCCCtaaGaTGcAccTGtTAACTGCTACAgATGTgGcAAGgacccAGGgCAtTTTAagaAGGATTgcCtAGgCAGCaTGAGGAAGCCACCatGAtCCTGTCCAATCTGtAATGGGGACCACTGGAaGGTaGACTGTCCCtttCAGAGatGCaGGTCACCAGgTCCAGAGCCAaTCTCCcAAATGaaTCCAGCAtgACTGATGggTCCTgGagCTCCTcTCcCTGGCTCcAgTGGTCCAgACCAcCATTACCATCCAGGAGCCCcAGgatttatAaTTCTGGAAaTTGAAGGGAGGAAAGtgGAcctCCTTtTGGAcACtgGGGctggtCTCTtaaGTTcTCctCTCCAaTCtGGGCCCcctcTCCTCTCTTAgCAcaACaGtGAgGgGtGTCTCAGGAAaGactTTAACCCaaTaTTTTTcCcaAcCCtTTAGTTGTAGTTGGGGActttgGACCTCTTGggTTcACCCATGcCTTTCTAATTATGCCTGAAAGCCCaaAAcTCCTCTGTTGGGtaAgGGATATTcTaGCccaTaggtgcctccacATgGGAACCACCATCCTGATGGCcCCaGGACAAACTCTTTGTCTCCatcaaagcCCTAGTGGAGACtAATaTTAACCCAGAAGTaattTTGGGCAACTCAAGGGAAAATTGGCaaAGCCACAACcGCCATctACCAGTCTGGaTcCAcCaaggTTAAgGATCcCaCCTCctTtCttaaggCTAACCAGAaACAATATCCCCtaAAAtgcagatCCAGAAgTTAGgAAAGGatctttCTAGAAGCCAattCATTGATctgAACTTtcctgggAGGaTGCAGGGCCTCCTttgtatCAAACCCTGCAACAGCCCTTgTAATACCCCAATATTGGGGGTACAAAAACCCAACAGGGAATGGAGACTaGTtCAGGACCTCcACCTTGTTAATGAGGCTGTGGTTCCAATTCACCtCAGTGGTTtCCAAaTCtaTATACCCTGCTgtAaCTCAAATACCTGAGGGAACTAAATGGTTCACAGTCCTGGACCTAAAGaGATGccggtatgcTTtTTCTGCATACCATTACACcCcAACTCCCAaTATTTGTTTGCATTTgActGGATCCCTCTAACCAAACCACCCAGcTAgACCTGGAcAGTGTTACCTCAGGGATTCtGAGACAGCCCCCACtTGTTTGGGCAGGCAtTGTCAAGAGAcCTCTCTGAGTTCCTTTATCCTCAGGTTAAAGTTTTACAATATGTAGATGACATaagTCTCCTcTGTGCTCcAACTGAGGAAATCTCTCAGGAGGGCAGTAAGGCTCTTCTTAATTTTCTGGCTAACAGAGGATATAAGGTTTCAAAAtcTatgacTAAaGCTCAGCTCTGgactcTCAGaACTTCAGTGAtaatAGTACCTAGcaGTCTGGTCTTGTCAGAGGGGACCcaAGGGCAtTGGGCAAAGAAAGGATTAAGCCCATCTCCTCCTTTCCCCTCcgaCCAAAACcCTCAAGCAACTgAGGGGATTCTTAgGCATTACAGgATTCTgCAGACTATgGATACCTgGGTACgGtGAaATAGcTCATCCCTTATaTCACCTAAgTAaaGGAaACTCAGgCAGCTAAaaCTCACTgtCTAATTTGGGAACCAGaGaCTAaAAgGGCCTTTGACCAatTaAAACAAgCCTTGCTTgAGGcAcCAGCCCTTagTCTcCCCATAGGGAAGActTttAATCTTTATGTATcAgAAaaaggAAgGaAATGGCCcTGGGAGTtCTAACacagGcCcgaGGTcCAGCCCAGCAgCctgTAGGCTACCTaAGtAAgGAgCTTGATTTgGTAGCtAaAgGATGGCtagCCTGCCTCTGGGcAGttGCAGCagTAGcCTTGcTGGTACCAgAGGCTACTAAGTTAACCAtGGGgAATAACtTAACTgTTTatAtcCCtAcaTAATGTGGCAGGACTgCTGTCTtCTAAGGgGAgTCTCTGGCTAAtggAcAACcACCTCCTcAaaTAtCAAGCTCTGcTATTAGAGGaaTCTGCAGTCCatctAaTTAAGAACcTGTCCcTcCCTAAACcCAGCCAcCTTCCTCCCAgAGgAagCtggGgAgctTGaacatgaCTgcaAaCAgatagtaGtacaAaCCtATGtggCcAgAGaGtAtcTCAAagAAACcCCCTTAGAgAACCCAGAcTGgAtTCtcTTTAtGgACagAAgTTcTTTTtGTAGAACAAGgGATacATAAaGCAcgGGTATGCAATAgtcAccCTGAATGAtaTTgtTgAGAGtatgcCTCTCttCTcgGGcAcAAgTGcTCAAcTAGctgAaCTAATTgCCCTCAcaaGgGtGCTcaaATTAAGCaAaGGgAAAgcaGTtAACaTTTatacTGAtTCTAAgTAtgcTTTCCTagTCCTCcATGCCCATGcCAcTATCTgGAAAGagAgaaactTCcTCAcagcTAaTgggtctcCcaTTAaATacCATCaggAaAttaatagAcTatTAtcctCAGttTtCCTtcCAtGGGAAGTGgCAGTAAtACatTGtAAAGGCCaCatattcaagCaAAAAgGgAtGgATGAAaTAGctaAgGgAAATAagagcatctTgGCAGACCAAGCAGCTAAATcaGcAGcaAgaGgGCCaCAGaTtTCTGAtCCAcTTGAaGgctcaCTgATCTGaGAggGCcCcaTaAgagAAATAAAACCTCAATATTtTCCTGTaGAAATAGAATgGGCCAcCTcTcAGGGATAcatCtTTcgaAGtCctCAgGaTGGCTAcAATTggAgGAtAGcAAGCTTCAtCTaCttcAGCTtCcAaCCaAtGGAAAGTTCTTAAAAtCcTTCAcCAaGccTTccACctAgGTAAGGAtAAAAcCtATcAaatggccCagAgattgtTctcaggtaaAaAtCTGcTAaAAatgGTtaAAcAggtCaTTAATgCTTGtgAGACTTGCCTTaAAAAtAATCCccTtAATtGAtagCTTcTtCCCcCaGGAAcCCAAagaAtaGGAggCTAcCcaGGGGAAgACTGGcAttcagGaTGgATTtCACCCAtATGcCAAAGacAAGGGgCATcCAgTAtCTCctagTaTGGatAGagttTACCTTCAcTAACTgGGtAGAaGcatTTCcaTGTtaaacAgAGAAAGcCTcTgAGGTGATAAAAGTACTAATtAATgAgaTAAtTCCtcacTTTGGACTtcCtaagTACCTCCAGAGtgATgtgataATaGccCCTaGTTCAaGGtaGCtGTCACcCAGGGaGtTCTCAAAgGCACTAGGCATAcAgTACCATCTTCAtTGTGctTGGAGACcAcAATcCTCaGGAAAGgTagAAAAGacaAATgatAtTATCAAAAGGcAccTCaGgAAACTGTCTctcAgGAGACTcaTctCCCCTGgAtTACccTTCTtcacATaGCcccTacTacgtgttaGAAAtaCtCCTtcaAAGCTGgGTTTaAgTcCCTTtgAAATGATgTATGGAtgGccTTTTCtCAcCAATgATTTCTTGCTAGACcAAgAAaccTCTGaTTTGATTAAaCataTaActTcTTTGGcCCATTTcCAACAgGAACTgaAAcaActGTtgGagGCcCAatcccaTGaACtAGgGCCAccTCTaTTCAACcCAGggGACtTagTaCTGgTAAAGGtaCTTCCTTCcCtTTCtccctcTaTAgGCCcagaTTGGgAgGgAccTTAcActgTACTTCTttctacTccTaCtgCAgTgAAagTCACTGGAATAGAtTcTTGgATTCAtTAtACtCgAgTAAAGGCCTGgGAAactgacagAgTtaCCTctgTtgAcCCAGaagAGCaCcCaAAGtACCagTGTGAAGAaATcGGaGAcCTCAAGCTAAAAAtcacAAAAgATaAGTgTcAataaTTaaccTTCcATGGATATcCTCttTatAgTCTtgCCtATGcTTGcTGTtaTTaCCTTtaTTCTgTtCttcAccATgaGgtacAtttTTGctttcaaactctTtAtcaTgaATatCacTtgttaTaTCTataatCtTaaaCAgtaattTcTtTcCTattatttaatttccctagcaCctATAcaagAtttatgtacacttAaccagGTaAAACttgTtTgtttacaacatTgtttTaaagatcATtAaactcctaaattTtaggcaactatagccTcagacaatgattttttTttgctaGtgactcTtagaaaaAcattcatatggaatCtatctacagTTatCccCattgtaAcActtatTgtttcctaacaagAGctaAgccaacTCaaaaTgattATtcaAATgGcagtTaGatgaatgtCTtccttttaaggagGgccctgAtatgGaCatcagagAGagAtcttactGgcataatcccaaaacaatgctgccaatgagCatgaagcagatacgagcagtCagtgtCCCtatgtgAgaacaagttAtaccTaccTtttcacaggaaggaTtgctatTgaaaggacacagacaaattcCtaggcagcCaattctgtgccCataaaaAaaCctgaCcTtaaagcCaaaGAcaagatgaagggtgaaaagagagctgctagttccaaGaaaAgtagataaacagaggaagaacttcctTgatGcagtaaattcaAtaaaattctggtttttccaagcccAaccataGaggaAtaagaacacactcccccattctgtttccagtaaagccagagactcaggcactcagagagcaacctactttttgtccccttctccacagaaGtctagCtagttgacggCcaaacTcaagtggaaagttattcTgtcACTCaaTaaaatTttcaGCtttccTcactttctagTGagaGCctacttCAttcctctTaatcaccgacaagaAtttAgaatccactaaattgTgggTGcgAccaaatagcTctaacAtgctgtacaagaactcaagtagtggtccataagactgtcatactgaccctatgctgaggtattaacacttaagggattcatagagctagcaaagctaaaagagttctgtgtaagacactgttactagagcttcaaaaaatattgaaaaccatcttggtcacactttaggcctcaaattcagagatgatgtaggcaacgctcagtggagacatcccataatggaagtggacagaagtccaggaagaatgacctgactctgcagtgccaccagagttgctataaagttctgaaatatattatgtttcttcataaaagttaagttctacagtcagcatgaaagtcagaaactacattgcggtttttttgttttgttgagaaacatgttttaaaccaaccctcaaccaacccatactttggagaaatacctacagaaactaagattggtgggaggcccagcagcgtctcacctcccatctcagactcagtccaggacacaggctcagggggcagaaggagggctg

>huersp2_10920_100030_con_gagputein

mgnqasvngaglflaggappswkelhGipkaqgtqgllgvggmeaflfanpsfigecrlglhgWglplvarlrgdtwgreKelvaflsiiwlvfalkeggnMgnayfpvfimGnpfsspppaYTPLECILNHWDCFDPQTLEeKCLIaLCTkVWpnYdlqegwpGLReEPFIsItilqWdlfvnvrgdgLRphmcraFftLqviqtfadsvrlvqalLlAiSGkAArGnPRtletqtpeaPpAGEPAPSgPvspGpPpLPipllSLTCPlLEIlTldqpPvSlLPlqRmlvElaPsKvQVPFSLqDLKQIKGDLGKFSDDPDRYIEAFQNLTQVFELSWKDvMLLLNQTLtnTEKQAALQVaERFGDELCITYSvREGvelYPiGREAVPldDPKWDPsDemGEwKRkhFQVCilEGLcrTRTKPLNYTKLSMIDQGFDENPTAFLERLREALVKHTSLSPDSVEGQLILKDKFITQAAPDIRRKLQKQALGPDSTLENLLKVATSVFYNRDREAqERERrHRKeaEaLMaTMqAHKPQNSQGAPVNCYRCGKPGHFrKDcpGSMRKPpqpvplvvgplegglspgglvtrsrtnlpngaaglmgpgaplpsssgpdhhyhpgapgnsgn

>huersp2_10920_100030_con_proputein

rdaghqvqsqspkwsiktddswgllfpvpvvqttittqepqvfleiegrkvdllldtgvgllVllSslgafsSLsTIMkgvSGRsLTqYfpQPlSCSWGDllFTyAFLIMPEstiPLLGRDILacigTTILMAPGQTlCLPLVETnINsEvWAiqgkig

>huersp2_10920_100030_con_polputein

pnqglgpgiaagqgtgklgkptpaipapllggpavaamgmplfmaggsilsplllpcgvgavwvtvsllpgqatfgpgVwvhLkdptSfpnqkqynqtfipLKPEVRKGLEAiIDNLkmqgLLKPCNSPcNTPILGVQKPtGewRLVQDLhLvNEAVVPIHPVvpNpYTLLaQIPEGTKWFTVLDLKDaFFCiPLHPnSQYLFAFEDPSNQTTQLTWtVLPQGFzDSPhLFGQALSrDLSEFLYPQVKVLQYVDDILLCAPtEEISqEGSKALLNFLANRGyKVSKSKAQLcQTSVKYLGLVLSEGTRALGkERIKpISSFPLpKTLKQLRGFLGITGFCRLWIPGYgEIAhPLyhLIKETQAAKTHsLIWEPEakkAFDQLKQALLeApALsLPIGKmfnLYVserKeMALGVLTqAggpAQQPVGYLSKELDLVAKgWpAcLwAvaAVaLLVPeATKLTmGNNlTvYiPhNVAGLLSSKGSLWLmdNcLLkYQALLLEgSaVQLRtCPSLNPATFLPeEagElEhdckqiVvQTYvaRedLKETpLeNPdWiLFmdrssFVEQgihKaGYAIvTLnDtieSapLsSGTsAQLAELIALtRvLkLSKGKaVNIytdSkyaFLvLhAhatIwKernfltaNgSpikyhqkInrLLssVFLPwEVaVIHCKGHqKgmdEIaeGNrLADQAAKSAarGPQiSDPLEipLiwegpvreikpqyspvEiegalSqgyifpplgwlqledgklhlpAssqWKVLKiLHQAFHlgKDKtyQlaQRLFSGKNLiqmvnqslmLVRlALKIIPlidgfsppgtqeigsypGpedwqmcFTHMPKiRgIQYLLVwiDTFTNWVEAFPCqTEKASEVIKVLINEIIPcFGLPKYLQSDNsPlFKaAVTQGVSKALGIQyHLHCAWRPQSSGKVEKtNnIIKrhLRKLSQEThlPWiTLLpIALLhvRNTPsKLGLSpFeMMYGWpFLtNDFLLDQetSDlIKHitSLAHFQQELKQLsEAQphElgplLFNPGdLvLvKalpSFSpslrPdleGlYtvLLSTpmaVKVTGIDsWIHYTrVKawEadgvtSvdPeeHpKyqcEEIgdLKLKiTKdksnnaf

**HUERSP3**

>huersp3_10930_100031_con_chaindnarm

tcaacttagacttggagttagctgacttaaaagtctaatccacattactggatttattgcctacatgactttgggtgtcagtttacttatccacacaatgaaaatgctgtgaggattaaaatgagatacaatggacataatgcagtcagcatgaaatctgacacaatgtcagtttccttttcattttcataataaaaaaaaataagttattaaaaaatttcttttctaaaaataagaaggctgaatattgatgtttttttcctttaaaaaaaatagaaccttgaacaatttaattctagaagaaaagcagcttgaaaggccagaacagcaagcattgatatgcaaatgccagcgattagaaaccaggtccatccaatatggtgattcccacaatcttattttgtcaacatgtgtgcctagcatcatggccacccccagaaaactccacgtatggagaacatcatggcggcctatatttgcatattaaAAggctagggtggGagggccagattTtttcatgggCTATGtaAATGaCAcACCTGgTCaAACCAATCtcttggGCCCTAtGtAAATCAgACACcaCCTCCTCaagagctcaacTATAAaagcCtgttcatttgctacaCgcagGgttttccattccagttttagagccccCtcCctctctgtagagAgaggtgattttCTTTtttcTTTCTTtcgcCTaaattattgcctattttAaacttCcgctCtTtaAAacTCACTCcatGTGTGTCcgTGTCcTtatttTtcatggcgtgagAaaaaaaaacCttGGgtaTttactCCAGaCAatagaGCCattTCATTTTgGGgGCcttGtCcaGGAtcCaaaGgtacaAcATTCAttggAaggGtggtaatcccAGTAtAGgAGcaaaCtccAAaaTCTgTtCTtTCATTtcgAGGCgcTctTgGCCTCcATTTTAaaatcaAAATCAAAtCAAacAaTgGGCatCctTcaGCCAgTTaAttAaAtAtgcTTAGtGTGGCtgcCagttTTacAAGACTcaGAtGacAGGCTTGCTGGGgGAGAACaTgGagAAtCCCCCctcagtaCCCacaGGttacTGGGAATgTTGGCcaaaccaatgTTTtGAACCAGtTTcttTgtTtcagGGaaaaCcTAGCCaTcaCAtGaGGCTGGaAaAaGTtCtGaaGcAACTGAggaTTtCTGGCcaGGGCtACaCCCTGGTGTTATcCaAAGGCTTCTGGACtgacCCCAGCCTCcaACaGCCcaaTcggGgttttGgaAcAGgATCtCCAaccaTttTgTatCAaAAcTTTCCTtCTTTtctttccgccgacCaCcaTgTCTCcTaTcctCTcTgTGTaTGcAATGcTGTGGGAAtTTTTACAGTTCAGGgAAgTAATCcTGTTagGCAAGtaaatggagatatgaTCAggaAATgttgccatAgTAAttttCcaggaATATAGggtCTCAagggattgctgtcgcTtTTTGTgATtttCTaGGAacaGAgaggGgtctcCctttccaggtctCtCccCCaCtctaaagtgaacatgcactttcaggcactctctgcccttggtCTgGtGAGCacATagcacttccaggtcTcTCTtctacccttggtctggagAGCAcATgGtATTTCaAaGcCaACAgcaCCACCTAGTGGAAaaTAgGaATCCTctccatgaggcacattgtcggttccTtTgCcgaAAcActcTAGtTTcCcAaTTcTccTcCcTTTTTgcaCCtCTCTACTAGAaAcCAGGCTtTATGCctCTTCTGTGAAcaaGGAAAAtTCTGccTtcAACAATtAGGAGtAAAATGTCCTCcaaAgCCAAATTTTAGTCTCaATACTGTCCCATCAGCAGGAAAAttGCCATTcgGTCCCTAtgTTCtTTTAAGGCACCTATTCTGcCTCCtggAATTAAaATaGTACTTAAtTAGTAAGGGGATTTTAaGTCtgGAAGTTAACCaGAACCAtTtTtCTAAGggTAAATGCTTTAGcacgGGCCATAATAGCAGgaTATAgAGcTCAAtCtAGcAcACTcCCTCCATTAAaGGGgcaagtgcaacagttgcccaaaTtcaaCTgTTACATAGTcTTTCCtgAGATCCATTTtTTttttttttttttcctaGGGAGcCAccttcaCAGGTCACAcAAGTCTAGGAAGTCAAggAGGGAAATCACacagagCAGAGGaACTAgaGtTgCaTGGGTaagtAGtGTGACTAatcCCAatcaCTTAGTTCCTCTGGTtCCATGGCTggGgGggTCAcaCCTgCAaCCATGGGcgGCACaTTTAAcagGgTGCcaGGgccCcaGGAACCAagGAGGGAAAAacCAGtTGggGGGgatgCCCCCACTGTcTTCctCTCCACCCTGgGtCActccaAaggAAAGGAAGGAGACTAAAgGGAcaCCTTTaTTCTCACTTCTCTTTCTAgAtGGgTaAcaGAtCATCTTcAGCcTGtACtCCtCTGGAcaattgTGcATTCTgAAgCACTGGGACTgCTTtgACCCTGAGACTTTGaAgAaAaAgccagcTCataTTCTTTTGCACAAGGGCATGGCCTTCTTAccatcttggagacagacaaacctggcctgctaggggaagccttgattttaatattatccaacaattagatcttttctgcagacagaagggcaaatggtctgaggtcccctatgtacaggctttctttgccctgtgagAcaacCCAGACCTTTGCAAGcaTTgcACAATtgACCCAGcTCTTTTAGCAGTCATAtCAGGCAgGCCCAaAGGGAATaaTTcCCCaAaActagaAAAGCAacTTCtaGaGGAaCaATCTGAGgCAGCTATTGAgTGTCCCAgCCCttcttgTtCCccTtatttggggcCCCCTCCAAtCaTACCATCAGCTCCTCCAGCTCCACCATCcTCCAAaAtTtCCCACTCtCCCaaCTTCACTCTTACCCCTACcAGGAAATGCCtaATGgGAaaTGaTGCCActAGGGTTCAAGTTCCCTTCTCATTaCAGGtaaatACCTTAgGCAAATAAAGGGAGACTTAGGCCAATTTTCTGATGACCCtatagaaacATAGGTATtttctatgaagtttttggaaacATAGAAGCTTTCCAAAATTTAACTCAGGTGTTTGACCTCACATGaGAGGGATGTTtATGCTGCTCCTAAGCCAAACCCTAACTGCAGCTGAAAAgaaCAGGCAGtTCTGCAGGCAGCAGAgAAaTttgGAGATGAGCAAtATGTCTCCTATAGCAtgcaGaCCAAAAAGGAAAAGAGAAaATAGGGAAGGTGAAGAAATAGtGGAAACACCATTCCCAATAGGAAGgaGAAGCAGTTCCTCcTTGACAACCCTgATTGgGAACCCCAATgACTCaACAGATGAATGGAAAAGgAAaataCACTTTcccccagccccccaccccctcggaaTTAATGTGCATATTaGAGGGCCTAcgAAGAACTAGGaaattgaGCCAAACCTCTTAATTACTCTAAACTGTCCATGATAGACCAAAAgCCAGATttaGAGAATCCCACAGCCTTTATGGAAAGGCTGAGAGAGGCACTAATAAAACACACCTCTTTATCCCCTGATTCAGTCaAGGGACAGCTCATCCTAAAGGACAAGTTTATTACACAGGCAGCTCCCAATATTAGAAGGAAACTACAGAAGCAgGCTATAGGACCAGATAGCACCTTaGAAAACCTCCTGAGGGTaGCCACCTCAGTCTTTTATAATAGGGAcCAGGAGGAGGCCCAAGAGAAtAGAGAGGAAAaCACAAGAGAAGGACAGAGGCTCTAGTAGCtGCTTTGCAGGCTTGCAAAGTCCAGGATCCCcGAGGTGCATCCACTAGTTGCTATCaGTGTGGCAAGTCAGGGCACTTTAAGAAGgGAaTGCtgcactCCAGGCAGCAAGAaGAAGCCACCTCGACCCTGTCCAGCCTGTGGCAGcaGACCACTGGAGATcaGACTGCCCCtaGAGATGGAGGTCACTGGGTTCAGAACCAGTCatcTCACAGATGGTCCAaCAGGACTGATGaGGTCCCGGGGgCTCAAACCCCTGGCTCCAGCtggaGCTCAAACTGCCATTACAGCACAGGAGCCCtGGGTGATTCTGGAAATTaaagGAAGGAAGGAAGGTAGATCTCCTTCTGGACACTGGAGCCAGTCTCTCTCTTCTCCTCTCTAATCCAGGCCTCCCCTCTTCCCATAGCATGACcgTaAGGGGtATCTCAGGAAAAAaCTCTAACCCAATATTTTTCTCAACCCCTTAGTTGCAGaTgcggggggggtGGGGGGACCTAtTATTTACACATGcCTTTTTAATCATGCCTGAAAGTCCCAaaagCTCCTTTATTAGGTAGAGAagtATTTTAGCTCaCATGGGGGcCtgCAGCATCCTTATAGCCCCAGGACAAACTCTTTGTCTCCCCCTGGTGGAAGCTAATATTAATCCAGAAGTGTGGGCAACTCAAGGAAGAATAGGTCaAGCTATAACCaCTAGGCCAGTCCAGATCcATCTTAAGGATCCCACTTCTTTTCCTAACCAGAgACAATATCCCCTAAAGCCAGAGGCTAGGAAAGGGCTAGAAGCCATTATTAATAACCTaAAGATGCAGGGCCTCCTCAAACCCTGTAAccAGCCCCTGCAACACCCCAATATTAGGAGTGCAAAAACCCAATGGaGGAATGGAGACTAGTTCAGGACCagtTCTGCCTCATTAATGgAGGCtATAGTcCCAATtCAaaTCCAGTGGTaCCTAATCCCTATACCTTGCTAACTCAAATACCTGAGGGAACttctTAAATGGTTtACAGTCCTAGATTTAAAGGATGCCTTTTTCTGtATACcATTACATCCTGACTCTCAATACCTGTTTGCCTTCaAAGATtCCCTCTGGCCAAACCgCCCAGTTtcctgAACATGGATGGTGCTGCCTCAGGGATTTTGAGATAGTCCTCAtTTGTTTGGACAGGCACTGTCAAAAGACCTCTCTGAGTTTTCtCATCCTCAGGTCAGcatcctcaagttagGGTCTTGCAATATGTaGATGATATtCTGCTCTGTctatGCCCCAACTGAGGAAGCTTCTCAGGAAGGCACTGAAGCTCTTCTTAATgcTTCTTAGCTAACAGAGGATATAAGGTTTCAAAATCcAAGGCCCAGCTTTGCAAAACCTCAGTGAAGTACacCTgGGTTTAGTGCTGTCTGAaGGGACCAGAGCATTAGGGGAAGAAAGGATTAAGCCtATTTCCTCCTTCCCCCTCCCCAAAACCCTCAAGCAACTAAGAGGATTTTgGGGCATTACAGGATTTTGCAGACTATGGATACCTGGGTAcGGTGAgATAGCTCaTCCaTTATATcACCTCATAAAAtGAAACTCAAGgAGCTAAAACTCATcTCCTAACCTGGGAACCTGAAGCTCAAAAGGCCTTTAAcCAGCTAAAgCAAGCCTTaCTTAAGGCACCAGCtCTCAGCCTTCCcgTAGGGAAGGCCTTCAATCTgTATGTATCAGAAAGGAAGGGAATGGCCCTGGGAGTTTTAACaCAGGCCCAAGGACCAGCTCAACAGCCAGTGGGTTACCTGAGTAAGGAAaCTTGATTTGGTGGCTAAAGGATGGCCAGCATGCCTCTgAGCCggATTGCCtCAGTGGCcCTACTGGTCCCAGAAGCCTaCCAAATTAACCCTGGGAAATGAcTTAACTGTTTATatcctgggaaatgatttaactgtttacaCCCCACATAATGTGGCAGGATTACTGTcCTCTAGGGGaAGCCTTTGGCTAACAGACAaGCTGaCTCCTTAAATATCAGGCTCagtgaaTGCTGTTAGAGGGTTCCACcATCCAATTAAAaACTTGTTCTCACCTAAAtCCAGCCACTTTCCTCCCCaAGGAAACTGGGGAACCTGAaCATGACTGTGAACAAGTtATaGTACAtGACCTATGCAGCCAGGGAAGATCTCAGGGAAACTCCcCTtgaaaatccagactgagaaaagaaaAGAAAATCCAGACTGGACCCTCTTCACgggtggatgtttcttttttttcatAGATGGAAGCTCCTTTGTAGAaCAAGGAgTCCATAAGGCAGGATATgCAGTAGTCACTCTaAATGAtGTtAtTGAAAGTGtaTCTctCTCcCtcCAGGCACAAGCaCTCAAtTAGCTGAaCTaATAGCTCTTACAAgctacaagatttcagcaAcTTGAATTAAGCAAgGGAAAGGTAGCTAACATTTACACTGAgtgcacttgagttaagcaagggaaaggtagctaacatttacactcaCTCCAAGTATGCTTTCcTaGTTCTCCATGCTCATGCTGCCATTTGGAAGGAAAGaCAttttttttTTTCTTACCACcAATGGATggacCtCCTATAAAATACCACCAGGAAATTAACAGGTTATTATCCTCAGTTTTCCTTCCATGAGAGaTAGCAGTGATGCATTGTAgtaAGGGACATCAGAAGGGAAcAGATtcaGAAATAGCCgAAGGAAACAAgTTAGCTGATCAGGCAGCCAAGTCAGCAGCAgtAGGAAGCCTCAatGAcATCAACACACTTcAAaCCCCTCTAATCTGGGAAGGCTCCATAAGAGAttccgaAATTAAaCCTCAGTACTCCCCTACAGAAATAGAATGGGCCACTTgtCTCGAGGGTAtACtTTTCAGCCCTCAGGATGGCTACAGTCAGAGGATGGCAAACTCCACTTGCCAGCCTCCAGCCAaTGGAAAGTCCTTAAAAaTCCTTCACtcCAAGCTTTTCACTTGGGAAAGGATAAAACTTATCAGTGTGCCCAGAGATTGTTTTCAGGAGAGAACTTACTAAAAACAGTCAAgCAaGTTGTTAATGCTTGTGAAGTCTGTCTTAAAAATAATCCCCTgAACAtGGCGGCTCCTTCCTCCTCAAACCCAAAGgATGGGAAGCTATCCAGGGGAGGACTGGCAGATAGACTTCACCCACATGCCAAAGATGAAGGGCATtCAATACCTCCTGGTATGGGTAGATACtTTCACTAACTaGGGTAGAAgaaGCATTTCCATGCCATACAGAAAAGGCCTCTGAGGTtaaaaaaaAATAAAAGTGTTAGTTAATGAAATAACTCCCTGCTTTGGTCTACCTAAGTACCTtCAAAGTGACAATGGcCCCTCATTTAAGGCAGCtgTCACACAgGGGGTCTCAAAaGCACTAGGCATAcAaTATCATCTCCATTGTGCTTGGAGACCCCAGTCCTCAGGAAAGGTAGAGAAAACaAATGATATTATCAAaAGACACCTCAGAAAACTaTCCCAAGAAACTCACCTTCCTTGGGTCACTCTTtCTTCCCATGGCtTTACTGcgaaTAAGAAATACCCCTTCAAAaTTAGGTCTtAGCCCTTTTGAGATGCTGTATGGATGGCCTTTCCTTACCAATGATTTTtCTATTAGAtCAGGAAgctgACCTCTGAatTgGTTAAGCATGTAACCTCTCTGGCTCACTTCCAACAGGAATTAACACAAtTAGCAaAAGCCCAACCCCAAGAAATAGGACCACCTtTtATTTAACCCAGGAGATTTGGTATTGGTAAAgGCTCTCCCTTCTCTCTCTCCTTCCCTAAGtCCCAAGCTGGGAAGGGCCTTACACTGTTCTTtCTTTCAACCCCCTCaGCAgTAAAAGTTACAGGAATCGActcaacTCCTGGATACATCACACTCaAGTCAAAGCCTGGAAAGCTGAGGGAGCtggaaAACCCCTGACAGCCCAGAGGAATGTCCTGgATATCAATGTGAAGAAATAGGtAGATCTTAAGCTGAAAATCAtAAAcAaAaaaTAAGTAAATGAGTgAGggCTACTCATCCTACTCAGTCtCACCCCTACCTcAcCAaATACTTTcaGTCATTTCTACcTtTCCTcTtgAGATTcgctgCCAgATATTAGAACTTCTttttgatgcatatttgcaagggaatttttaattatccatagagttacattataacttcatagagctccaaagagaaattgtatatcttggcaaataacaattttagatggaaattatttactacgccacccttgcaggaattgctatactcactctactatttgcagtaggactatatactgtagcaccctcagggtggaatatcagacagagaatctcaattactgtagcaTTTTGaTtcATaaTTatcctcatagCAGGaATAaTagttactaacaggaagtaaacatgagcgtTTtAcTATCacTgAGtcTgctaggactttttattggacTTagtAATtaacaCATcAcACCCtttagctcctacaatatctgccatggcccatttgtacaatAAaagtAAtTttTGtaTCTgtcctaagTGGTtaGctcAATTtaAtAacacTgAGAAttaTTtcaATtACcCcaaACTTgCcgtcttAGGAtTcccttTGTTAtaCTtACctTtaacccttaAagAtTTataaggcgtAaATgggaCAtggTAtgggagCagTttCAaCTGcataactaAaTtcTctcagGAaAAtatTcaAttgCtatgtgccaaagAAcAtTCTgCCtAAatATaAtCTTcAtAaCctcAGGctgggaaaagttgactaagTAATAGcaaatgcctccctctgctttaaaagtaACAGGGAAGAAcCAtaCatgggagatctcaaAaaTTgTAacATCACactTGaAaTTGCTgttAgTTcaaAgatttggggaggagagtgcCaCAagggtgatctaaaaggatcagaagccctagtgggggagtggggggctttcagaatctagccactCcttctggttggaaggatcccgatgggaatggcatgctctaagtaaccaccttgactataatgtaaagaataacacatgggcctttccaaacatgcccccactcatggaattccagacccTTgTATGatgttTgCTAACagTAGaGgcttacaAAtcTgTgGGCaAActggAgaCaTctggactgacagcccttgccacaaggATgatCaTcTAAgAtAttgGCcAggGtAtattatgtctctaatttttcaaaactccatttgtcaggtggacCcTtctcATtTGGcatgtcaaattccaaatggcaagGtAAAtgAgTATtatAAatatttgattatccttatcacaAggATGcCTctattaTATgtAAagAaggcaAactTCTagGATAtgagaaaaatctgctggtgtctctcacaagccacaacttagaaccaTggCtTcaaggaacaGtatTActattttctttgtggctcctggatacacttaatcctcccaaggcactagaaaGGAAcgtgtactaTAaTagcAGtagttcctgacttattatttttaaattctactaagatggcagcatcatctggggacatccctaatttaggctcttttctagaaAcTgcActatctcaaatacaccggacAAagagatctaTcatTtctAtgccctcatatggagatttaactgaAagagcagactggggagggcatgcAcATgacAaTccCAtcttagaaaaaccatggATGGgAaattctatagcaAgaGgaaTattctggtttatgggcatccctcctctccttgaaagatcagtaCTTAATAttcaTAccTTatgatgcaatgaggatggaaggcaactgtaggggccatagaggcacaatagcaatctatagactctttagcctcagtagtagcacaaaatagatgggccttagatgtccttacagctgaagtaggaggtacctgtgcacttttaaatgaaacatgctgcttctggattaacacctctagtaaagtagaggaaaatctacaggtacttaaagatcaaatcaaaattattgacaggctaagagaaaatgcaggcttcagccccgggtggctacaatccctctttaatgaattccagtcttctttatggaattggttagctcctttattaagccctctcttgcttacAagtCCTaTATTaAtacaatactataactcaatacttTggAacttgtataacTcAAtatTaTagtaAAAtTTaTcTCTTCTcaCCTAGAAGCaAaaTcAAACTCCAAATGGTGCTGgCAGAcgGAACCAcgCATGGACAtgCCaTTCTTCcGAGGACCCTTAGATcgACCCCAGGAGGAGCCCTAGCTGCTGTTgttCCCCACAtgAtgCCCCTTTtCAGCAGGAAGTAGCCAGAAAGAGTCaTcgtCCgAAcACCCCacccCTAAcAGCAGTTAGGGTtACcaCTCCTGAGgGGGGAaaATGATAcAGGAGtTAgaAAGAAATTATTTtAGGCAGATAGTgAGGGtAAaaGAGTCCTcaGcAagGcTTttcTTttaActaAAAagcaagctcccCcccaAAATCATTTCttTttcTAacaAAGAGcaGCCTGaAAAattaAGCTaGCAAACATAGATAaGCAAgctccagagaatagaagctaggtccaccctctaagacatagattatcaatatGgaAGcTtgCAtgGgtGaaTgcTgGcAgctgtaccaatagAAgagggctacCTaGGgaccAgaCATaTtCaagcATGgagGctccAtcTTcCctTttctttgattaacataTtaccAcgTgtacagtAaagaaagaacaggcaAcaTGgcgccggccaggaaGAgAatCCattTgcATaATAaaagaTtAGggTgggGgaaaCCAGattttcacaTgCaCtAtGCAaatggcAtAcCtgatcgtaAcCagatatataagccgtacataaatcagacaccagatccaaccaatctttattataaaatgcaattcatatactaCctgctcatcttttatatacaaaagactcctctatttctctaaagaacgagcaatttatctttctattctcctctgcctattaaaagtctgcacctaaactccttctgtgagttagtgacctaaattttccaagcataagataagaaaccctagggtctatacaccagagaacataagacaacaaatctaaggtatttaccccagacagtgaccttctatgaacatgggcatggctgcgaacccctcatcatcaggtgtatagcaagtgaaagcaggagttcacgacagtgaaaagttgagcgtcatttttcttacagtgtgccaagagttagatattagcattttcattgtattttcttacagtgtgccatcctgttagatatttgcatttttgttgatgaatgagacatagttaatgcatatttctgtttgtgtatccatgcacctacctcagaaaacaagtattgtcaggtattctctgcatagaacagcactaccctcctctctccccagatgtgactattgaggccagttctgggtgtttcagattttttttctctgcatttacacacacacacacacacacacacatacacacgtggaataccactataaatatctccatctgcttttccccatcgctaatgagtcctggtcaagcccccctcactctgtttcctgttcaacatgcactccctgctgcaaactcccctcatctgattcccctgtgttggtcactgccagttaataaacatttacaaacttaaaaaaacagataggacaaggggtccttggcaaggtttttttcttttaaagcagctccagaaactttattgcctagcagaaaaacagcttgaggggctgagccggcaagctttgatatgcaaataccagccatcagaaactgggtccactcaatacagcgattcctgcctcaacttcttgtcaccacaagtgcctagcatcatggacaccaccacatatccccatgtgtatagaacatcatagccccctgcatttgcatactcaaaggctagggtgggaggaccctttttttggcgggttaagtgaatgatatacctggtcaaaccaatcccctgggcctcaaatcagacactgcctcctccagcctcctaatataactggctcttttccacactcggagttccccctctca

>huersp3_10930_100031_con_gagputein

gglvlvvgalavagiagtvvlpfgllmleglqlpsgcglimvfvssgitfflfffvgsagipflsallmgnlqfclvllwyalgnsllvvvggtlcrvlglgmggiitvgptlevilsplgagspgyilkgpfiffllfplgnrlslgsgslgviskalgafvpvplsptvplslrlrtlpsspggrgqtwpaggslsFNviQqldllmglnqpglfagalilplsnillFirqkgKwsevpflpaayvqaFfaLzdnPgdLfksctidpsaailsaaiflgalqgptLLAvisGkrpkpggnSppleKQlpgEqSEaAIECPnPSSpPylgppPivPSApPaPpsPklPtpPtSLLPLQEMPngndAtRVQVPFSLQDLkQIKGDLGQFSDDPnRYIEieaFQNLTQVFDLTWRDVMLLLSQTLTaAEKqAvLQaAEkfrdeQyvSysrpKRKrgdrEGeEivETPFPigREAVpLDNPdWNpNnStDEWKRKHFLMCILEGLqkTRAKPLNYSKLSMIDQKPDENPtAFMERLREALIKHTSLSPDSVKGQLILKdKFITQAAPNIRRKLQKQAIGPDSTLENLLRVATSVFYNRDQEEaQEKERKhKRRTEALvAALQACKVQDPzGASTSCYqCGKSGHFKKECPGSKkKPPqPcqavgettgdrsapgdrghWvqncshrwsslgdgslglkpllqaarlplviltpgglgll

>huersp3_10930_100031_con_proputein

pgseiggaivgltdwvpgLkplAPaaQTAITAQePwVILEIEGRKVDLLLDTGASlSLlLSNPGLPSSHSMTimGISGKiLtqYFSQPLSCrwGnLLFTHdFLIMPESPTPLLGRDILAhMGASILIAPGQtlCLppveasinpevqgr

>huersp3_10930_100031_con_polputein

asillapgqslclplveaninpevwatqgrigqaittrpVqIhLKDPTSFPNQrQYPLkPEARKGLEAIINNLKMQGLLKPCNSPCNTPILGVQKPnGEwRLVQDLCLINEAiVPIHPVVPNPYTLLTQIPEGTKWFTVLDLKDAFFCIpLHPDSQYLFAFkDpSgQTaQLTWmVLPQGFZDSPHLFGQALSKDLSEFSHPQVRVLQYVDDILLCAPTEEASQEGTEALLNFLAnRGYKVSKSKAQLCkTSVKYLGLVLSEGTRALGEERIKPISSFPLPKTLKQLRgFwGItGFCRLWIPGYgEIAhPLYnLIKETQgAKTHlLTWEPEAQKAFNQLKQALLKAPALSLPvGkAFNLYVSERKGMALGVLTQAQGPAQQPVGYLSKELDLVAKGWPACLzAIAsVALLVPEASKLTLGNdLTVYTPHNvAgagLLssrGsLWlTdSwLLKYQALLLEGSTIQLKTCShLNPATFLPkeTGEPEHDCEQViVQTYAAREDlRETPLENPDWTLFTDGSSFVEQGihKAGYaVVTLNDViESasLpPGTStQLAELIALTrAlELSKGKVANIYTDSKYAFLVLHAHAAIWKERHFLTTNGSPIKYHQEINRLLSSVFLPzEvAVMHCKGHQKGTDEiAeGnkLADQAAKSAARKPQdINTLqaPLIWEGSIREIKPQYSPtEIEWatlrGytfsPqsGwlQsedgKlhlpasSQWKvLKiLHQAFHLGKDKTYQCAQRLFSGENLLKTVKqVVNACEVCLKNNPLNRrLLPPQTQRmGsYPGEDWQIDFTHMPKmKGIQYLLVWVDTFTNWVEAFPCHTEKASEViKVLVNEITPcFGLPKYLQSDNGPSFKAAvTQGVSKALGIqYHLHCAWRPQSSGKVEKTNDIIKRHLRKLSQETHLPWVTLLPMALLqvRNTPSKLgLSPFEMLYGWPFLTNDFLLDQETSELVKHVTSLAHFQQELaQLAeAQPQEiGPPLFNPGDLVLVKALPSLSPSLSPSWEGPYTVLLSTPSAVKVTGIDSWIHhTqVKAWKAEgAtPDSPeEcpeYQCeeigDLKLKIiKdkk

>huersp3_10930_100031_con_envputein

wtgygsgailvgnvpqatlgnnalpadklhilrlgkvdwvianaSlcfkssgegpylgdlkycNItLViadsskiwggacrkgdkkgrlsspvGGFqnLaSpsgwkSqlewHALSNhLDYnvkNNtwaFPNmpphgIpdpCmmfaNssgslqIcgqTGdIwTgspchkmdhlrYwPgNnmsLifqnsicqvDPshlacqipnsigNdYtkYgypyPKDApIicKegklLRYeknlkVSLTshnLplSlqgtglyFlcgllltLIlprhwkGtctIvavvpdLlllNstamgassGdiPNLgSFLETaLsaiHatKrSIISMPSyGDLTeReswgGhahdnpIlekpwmGnsiarGlfwftgipllersvlnisimvqkgwkgtvraieakpqsiDslasvgvAQNRwpalDVLtAEvGGtCallNetccFwINtpsfveENlQvlKDqiKiIdRLRenAGfSPrwLQsLFNEFqSsLwNWLaPLLSplLLiCLiLsaifgpcllntitqivssllsaikpngalnrttcmpailfrglllppcsppcsvptlrpfpeevggkvphpkppnss

**MER66**

>mer66_10730_5711_bre_chaindnarm

tgtcctaaccagacaggaaactggccagaaccagcaagtgattctgtaagcaatctctagttgccctcactgcccatcagcataagacactcccaccagtgccatgacagttaacaaatgccatggcaatacctggaagttaccacccctttccatggcaacaccaaaggttaccactcagtttttagagatttctgaataacctgtcccttaatttgcatgtaattaaaagtgggtataaagctagccaacagcccatacactgccactcttggtgcacagcctatgggcttgccatgctcaacaaggggcagccactgggctgtaacactgtcacttcagtaaagctgctttcttccattgctggtttgctcttgaaatctttcctgagtgaagcccataacctgccctgcatcatctggtgcctgatgtggggctagggaagatggtggcaatagcagaggtagctaggcagcaagatgcagagaggcagcgactggagagagaaggcagtgaggtagcaagccagtgagaggcaatgagatggcaaatggcaagagacagtgagacaatctgtgattgaagctgcaagaactgtaacacagaggttataaggctaaacagctgctaatgctgaagagctataacactagtctaaggctcttttcagagccatcatctttcctgacaggtggaggcagcagagctgtgtggacgggtcagtggctgcgcagtgctaccaccttgtgtgggaccaactgtcttggctggcaggtcactggtgtgtcagctggacccccgtaacagccgagcctgcccaagctggtggaatctggggagaacttcacccatgtcccacattggagactggtcagcaccatttaggcacttgaagatgggtgagtgtcctctctgcccctccttcaatatcaggtaaaacaagaaataaggcctctgtctaggtggtcaattcaatgtccgccatcatttgggtgcccggaagagaagcatgcctgaccacctacattcttgtcaccccttctcttgatcctttctcctctaatgctattttatttggggcatttggtgagggaatcctcaatgggtgttacttgggtgcattggggttttttgcactgggctgttgtctaccccctgaatgctctggcgtttttgacattggcattccctctaggattgtgggttatagcccctccccctgggagaaccttggtctttgatagggttttggtctgtgtccccatccgaatcttatgttgaattgtaatttctaatgttgggggagggaccttgtgggaggtgattggatcatgggggcagatttcccacttgctattcttgtgatagtgagtgagttctcatgagatctggttgtttgaaagtgtgtggcacttccccctttactctctctctctctctcatgctctgccatgataagacatgcttgcttccctttcaccttgtgccatgattgtaagcttcttgaggcctcccagccatgcttcctgtacagcctaaggaactgtgagtcaattaaaactcttttctttagaaattacccagtctcaggtagttctttatagcagtgtgagaatggactaatacagtcttgttttttctgcactgaagttggaaattatcattttctataacagccagttgtgggcctctccctgtgttctgtcttcctttctgtccaatagactgccttgctcaagcacatcttggactgttggttgaagggaccaggggttcccagacccctggcctgacagatgagtacccgcagcagtggatgagtggcctccttaacactttctttggtgtctctactgctgggtggattatccagtagctgaaggtctctgaggtctccctttgagccatgttgttcgcccatactcttcctccgttccacaatcactttctatttttttaaaatccatctttgcccaaactgaaatgctttcttcactctctttggaattcagacccatcattctactgcccactcatatctcatgatccacttttgtaatgctttgctagctatactaacaccttctctgcaggacgtgagaatttgaaagggaaagtaaggctctcactaaacttaggcaaacttgaaaacctcctgtacagattcttactggacaatgggaacaatggtgaccatcctggaagactcaacagtagggtgtcttttaggcaattggtgcaaattcaaattagaaactttaaaaagaaaaggaactcattttctattgcaacaccattggggtccaatataaattgggagaccaggaaatttggtctaaaaatggttctttatgttaaaatataatactattttacaattagacttattctgtaaaaaagaaggaaaatggggagaagttccttatgtgtaggcttttatatgccctctaccaggatcctgacctaagggatagctgtagaatgtatcagactcatgttacttccaggcaccaagaagctgtgccaggtatcataggtgaccccctcctagctgctcccactgggaggtccatgccccccttggagcctcctcagtcctgcagttctgagaggaattctgccagttctccagcacaggattccaccccaaggtcatcaggcacccctaccccttatccaactagtcctgttgtatacccctgttacctgagaatgtaagcccaaccagtactaccaggagcagggccccgtatcagcccctaaaagtgaaactgtgtccattgtgggtagctgatggagatgggggaacaatcagagtatatgtgccatttactgtatcaaatttggctttatgcaaggagaaatttggctggtttttggaggatccatggaagtttatagaggagtttgatacatttaccatgtcctctgaaataacttggcataacttgtgcttattattatccacttgctgtaccatagacatagaggagaaacaaaggattctaggtattacccatgaattttcagatggagtggccaataatatccaaggccatgccatttataatgtggggggagaggcagttccttatttgcaccctcaatgggattcccagaggggttcccaagatctcaaacacagaaattacatgctaacttgtttagtagaaggtatgaaaaagtgtgtggttaaaccagttagttgtgacaaggttagagaagtaactcagggaaaatatgaaaatgccactttatttcaaggccacttagtggaggccctcaggaaatatactaatgcagatcctgactctctggaaaggtgagctctcctggtcatgtattttattactcaaactatctctgacattaggaggaatttacataaggcagcaatagagcatcaaatccccatgagccaacttttaaatatggcctttggaatttacaataatagggacaaggtggagaaagaggtgaaaactaaaagtaatagctaaaaagtgctattattcgtggctgccttaagccccttactgccttagggttacaaatgtcaagaaagtgttacgagagtcgtggttgggatgcccagatgaaagcctctgacttcttggcccctgggccaaaatcaatgtgcctataagcaagaggaacactgaaggaaggactgccccaggcttgaaagagagtctgaaccactcagacccataatggctgagagaacagaggactgacagggcctgaggtctcttatggtttccaatggacaccttactatctccatagaggagctttgggtaactcttgatgtggcaggcaaaaataatgagtttttggtggacatgggagcagacttctcagtttcaacctatttctcagggccattgttttcccactcttgtaccataatgtagattgatggccagccaaaagttaggtgattcacccaccccctcagttacactgtggggaatcatgtgttttcccatatctttctgcttatgtctgagtgccctattcctttactgggaagagactaactttcccaattacaggccacagctcaatttggagagccttatgagaaggcaataggccagggaaagacacttcttctagctccaagtacatgccttaacacagataaataaaagacctctctttcactgcatcttacttctcaagtagacctctctgtttgggacatgaaagttcttggtagagatgttaatgtactcccagtccaggttattttgaaacccagagttaagtatccatacaaaaaacaacgtaccttgagacctgaagagcagagaggcattcaacccctaataaggaagttcctaaagtatggattactacagtcccgtcagtccctacgtaacacctccattttggttgtgaagaaactgaatggggaatacagatttgttcagaatctgagggcagttaatgaggcaatagtcccagtccactgaatagttactaattcatacacaatattgacccaagtccctgaagatgttaatagttcatggtattaggcttaaaggatgctttcttttgtatacctttacacgtggacacccagtatatctttgcttttgaatggactgatccagacattcatgctgcatctcatcttacgtgggctgttcttcctcaaggttttagggacagtccccatttctttgtcaatacattggcaaaagaattaaggaaactctagttaaataatgggtctctttagcaatatatggatgatctattaatttccagcccaaccagggaaggctgtgacaggaacacaatccagattcttaatttttttgagaagatgaggttattgggtattcctacaataaagcccaaatttctgcacaaaaggttaaatatttggagtatgtgttaactcctggggcaaacaccctggcccaggagtaaaaagagaccatcttggcactccagccccctcagactaagaaacaattaagaatctttctgggaatggccagattcttctggatttggagtcccaggtttgggctcatagcaaaaccactctatgaagctctaaaagagagtgatcatgagcctttgaattgggatggaacctgccaacaggcattcttaaccctaaaagaaaaattgggaacagcccctgctttgtgactcccaaacttagaaaaacctttcaccctatatgtggctgaaaaacaagggacagctttgggtgttctaactcaaagtctcgtgaatagccctataccagtggcttacttttctaaacagctagaccaagtggtggctgggtggccaggatgcttttgagctgtggccaccaccactctattggtagaagtcagtaagtttaccttgggacaacaattagatgtaatgacctgcccccatcaaatacaggggatcctagaggcaaaaggacaccaatgtctaacagtggttcaattacttaaatgtcaggttcttctatgtgacaccgcagatgttacttttaaaatatgttgttttaaatcttgctaccctgttgctggacttcacgtcccagtccccaactcattcactcctgtgtggaaactatggaacagagctcctctagcaggcctgaccttaaagatgagtccctgcctaaccccaatgttgagtggtttagagatggaagtagctttattcatgagggattaaaaaggcaagttatgctgtggctagtcaacaataagtcattgaggccaaagctcttcctccccagacctctgctcaaaaagtggaattgaatgctctaattagggccctccaactgggaaaagacttaagaatcaatatatttactgatccaaacatgggttcctggtgctccatgctcacaagccatatggaagaaaaggggactgttaacagccataggattcccccatacaacatcactctgagatcttggaacttttagatgctgtccaactcccaaaagagataacaattattcactgctgaggacaccaaaagggagacacctctattatcagaagaaatgtcctggtggatagagcagtcatggcctccgctaaaggaagatcagtactgctggctgctgcactaatacctgatgctccacccatgtcaacagtgccatattatacaccttagcaaattaaacgggcagaacagaaaggcttacaaaaggatccctcagagtgatttctagaaatcaataaactctttctccctgaggctgagcaatggaaaataattaagcatttccatgactcctcacatttaggatgggattctgtattcaaatttgtttccctaatattcttgggaaagggactattccagactataaaaagggtcaccaagacctgtgaactgtgtgctcataatgacccaggaagccaccccatactccaatccctactcaaacctgtacaacaccaaggaacataccctaggcaagactgcaaatagatttcacccagatgccaccttatagggactaaaatattttctactatttatggacactttcactgggtggatagaagctttccctacaaagacagaaaaagcattggaagtgtccaaattcttaaagaaaacatccccaggtttgcattaccaaaaagtttgcaaagtaataactgaccttcgtttacagctaaggtgactgagcatgttccctcagccttaggcattatctatcatcttcactccttctggtggcttcgatctttaggtaaggtagaaaaaagccaaccatgttctaaaaaggacattagcaaaactgcgtcagtagacctcagaggcctgggcttctcttctaccatagcccctttgtacataaggatggctccaaagggaaccgtaaaacttagtccatttgaaatgacctatgggaggcttttttttttccttcaggcctcctgcttaataaagagacacatagaatgctcacatgtattatcaacttaggccaggttcgaaaggcccttcaagaatacggaaatgaagtgttgcctcctcccacaagggaaagaattaactccatcaactccttcattcaacgaagagacttaatcttactaaaaacttggaaagaaggatcccctgaggatcaattacaacccaaatggaagggtccttatcaggtgttaagtacccttattgatatagtttggatatttgtccccatagcaatgcaagaatggcctaatatacccactgtgggtggcgagccacccaggtgccaaggcaagagaccgagggcacaagctgttccagtataataaagaaaatatatagaataagaatagttatactagaaatagattatagatatgattatatatgaatattattaatcattagtttgtagcatactctttattccaatattgtaataatctttgttctacaattataacctaggaaaaaccaggccatacagagataggagctgaagggacacagtgagaagtgaccagaagacaagtgtgagccctctgttatgcctggacagggccactagagggttccttggtctagcggtagtgccagtgcctgggaaggcacccgttacttagcagactgcgaaagggagtctccctttcccctgggggagttagagaagcctctgttctaccacctcttgtggaaggcctgatatcagtcaggcccgaccgcagccatccggaggcctaaccatctccctgtgatgctgtgcttcagtggtcatgctcctggtccactttcctgttccaccctgtacacctggctctgccttctagatagcagtagcagaattagtgaaagtactaaagtctttgaaatgcatagaagaaataatgacgtaagctgtcccctctctctctctccgcctcggctaccaaacagggaagggccccctgtctggtggacacgtgacttgcatgaccttacctatcattggagatggctcacactctttatgctgcccccttgccttgtatccaataaataatagtgcagccaggcattcgggggccactaccggtctccacgtcttggtggtagtggtcccctgggcccagctgtcttttcttctatctctttgtcttgtgtctttatttctatgatccctcgtctctgcacatgaggagaaaaacccacaggccttttagagctggaccctacaacccactgctgtgaaacttcaggacatcactagctgagtacacctatccaggatgaaacccatttcttatgagtccccacaaatgcaaaagaagaacaccatgacctatacctgtgaatctttggagtacctccactacctatttaaaagaatcaacactcagccagaagtggtaatgtgatgctgtgagtgggaataggaacattaatttttctcttcttcctgattgtatacttcttttctattgctttggccacttgcctcctcctgggaaatacctcttttgtccttgttgggtgtagaggccactctaaggtccaaccagacaccatgctgccactgttaatcctgtttgccatcctgattaacctaatccagtgtggtgggaataaaactctacagtaaatatttcaaaaatcataacatcaaggaatcatcttcatgattgctgcatttgtcatcaacatccccacgataaagagttccactttctgacacatctggaaaacctcatggccatctccctggacctcctaattaaccatagtgatcccttagtacccagatccttacttgttaggtggaactctttcccacttaatgaatttgacctaactgcccctgccactattacctggacctgggagattgggtacctgtacttcaggtgtcctaatgacactatgttttgcactcattgtgttaatgacacaaaagtggatactttcctgacatgctctgatccaatcctagttgccaaattcctgaggccacagacaggtaaatgggatcccacttgtaagtgagaaaacaacacatggtgcttcctcctcgatcagaacatgcaaggggaaaataactgtctaatctaggaaggtaagagtactggcttcttctggaaaacaaaggatgtttgactacccccattggagcaagggaaatatatctatagacacgactgggtaacaaaggatagacatcacatctcataggacttctatttgtgccccaactgggcttatttttgtctgtggccatgagtaagtagaagtcccacctcataaccactcctgactccctggggagctatctgttcttttaggagtagcttttccttgtatactaaaaactagaaatggggtgaatgtatactggccatcattgcccctctgggagtcaccatctataatcccataaggcccaggaatatcagaaataaatgagcaatagggttaattctggcaggaatcgagacagtaataggactagtggccccctggggtggctttgtctcccattagtcaatcctaaagaacttgactcaaaccctagaatcactggccaccaatataggccaggcattaaacagaattcaagagttcctagactctttgacaaatgttgtccttgacaacagactagcattggattattggattatttgctagctgaacaaggtggagtctgtgcagttattaatatgacctgctgcatgtacattaataactctggaaaatcgaggttaacattaggaaatctatgaacaagctacctggttagatagatgtaaccagggcactgaccccagctgtatctggtcacttatcaaaagtgccctcccaaatcttgcctggtttttacctttcctaggaacttgctagctatcctgttactactaatttttggcccttgcttgtttaaccacttagtaaagtttgtgtcttccagattacagtaaatccatgtaaagaatgctggcacaaggcttccaacccatcctgtattctgacctggagaatgaaaatatcctgcccttggttccgttagatcagatatccagaaatttttactcctccaatgctaggcagggcctatgcccataaaaacagcaggaaacagttacagaagatggatctctgacctgcagctcctttaagattaaggaggcatatctaatctccgagcaggggaatgaggtaggagaaaagtagcacttgttttctggaccagatagaaaaccagccagaaccagcaagtggcaccaaaagcaacctctggttgcccttgctgcccatcagcataagacactccccccaccggtgccgtgacagtttacaaactccatggcagcacccagaagttactgcccattttctagagatttctgaataacccaccccttaatttgcatgcaattaaaagtggatataaatacagttagcca

>mer66_10730_5711_bre_proputein

TLDVAGKnneFLVDmGAdfSVsTyfSGPLfshscTImrlMASQKlgdspTPsVTLwGimcfpISFLMsECPipLLGRDzLSqLQATaqFgEpyEkaigQGktLLLaPsTclnTdk

>mer66_10730_5711_bre_polputein

zkTsLsLhLTsQvDLSVwdmKVLGrDVnvLpVQViLkPRvkyPykKQrtlrPeEqRGIqPlIRKFLkyGlLqsrQSlrNTsiLvVKKlnGeYRfVQnLRaVNeaivpvHziVtNsYtiLTqvPedvnfmVLgLKDAFFCipLHvdtQyiFAFEWtDPdIhaaShLTWavLPQGFrdSPhfFvntLakeLrklzLNngsLzQYmDDLLissPTREgCdrnTiQiLnffEkmrLGYSynKAQIsaQkVKYLeYvLtpGantLaqezKETILalQPPqTkkQLRiFLGmARffWIwsPrFgLiAkPLYeaLKESdhepLNWdGTcQQAFltlKeKLgTAPALzLPnLeKPFTLYVaEKQGtAlGVLTQsLvnspiPVAYfSKqLDqVvAGWPgCfzAVAtttLLVevsKfTLGQqLdVmTcPHqiqgiLEAKghQcLTvvqllkcQvLLcDtADVtFkIccfkScyPvAgLhvPVPnSfTPVwkLEqssssRPDLkDesLPnPnveWfrDGSSFIhEGlKRqvmlwlVnnVIeAKALPPqTSAQKvELnALirALqLGKdlrINIfTDpnmgSwcsmlTsHmEKRGLLTaiGspIqhhsEILeLLdAvqLPKeitIIHCzGHQKGDtsiiRrNvLvDrAvmAsakgRSvLLaaaLIPdaPPmsTVpyyTPzQiKRaEQKgLqKdpsEzFLeinKLfLpeAEqwKiikhfhdSShLgwdsvfkfvsLIfLgkgLfQTikRVTkTCelCAhMTqeAtpySnpySnlYntkEhTlGKtAnRfHPDaTlzGLkYfLLFmDTFtGWIEAFPTKTEkALeVsKfLEnIPRFalPKsLqSnNzPsFtAKVtehVpsaLGIiyhLHsfwwlrSlGkVEksqpcSkKdisKTASvDLrgLgfsstiApLYiRmAPkGtvkLsPFEmtYGrLfFFlqaLlnKeTHrMlTciinLgQvrkALQeygnevLppptRErinsinsfIQrRdlilLktwKEgspEdQLqpkwkgpyqvlstlidivwifvpiamqEwpniPtvgGePprcqgkrPrAqaVpiIkkiyrIriviLeidyrydyiziLlIiSlzhtlySniViifvlqLnLgkTRpyRDrS

**MER52**

>mer52_10940_2310_bre_chaindnarm

ccatgatagaccagggatttgatgagaatccccactgccttcctggaaaggctaagagaggccttggtaaaatacatctctctgtctcctgatttcagttgagggacaactaatcctaaaggataaatttattattcaagcagtccctgatatcaggaggaatctgcagaaataggccctggggccagagagtactttagagaacctcctgaaagtggccacctcggtgttttataatatagatagggaggcccaagagagagagaggggaaacacaggaaagagatggaggctttaatggccaccatgcaagcacacaaaccccagaattcctgaggtgcacctgttaactgatacagatgtggtaagccagggcattttaggaaggattgcccaggcagcatgaggaagccacctcaatcctgtccaatctgcaacagggaccactggaagatggactgtctccagggacactgctcactgggcccagagccaatctcccaaatggtccagcagaactgatgggtcctggggcccctctcgccagctgcagtggtccagaccaccattaccatccaggagccccaagtaattctaaaaattgaaggaagaaaagtggacctccttctagacaccagggctggtctctcagttctcctcttctctcttagcacgaccataaggggtgtctcaggaaggcctttaacccaatattttccccaaattgtgatttgtagctggggaagcctcttgtttacccatgcctttctaaatatgcctgaaagcccaactcctctgttgggccgggatattctgacctatacgggaccaccaccctgatggccccaggacaaactatttttctccccctggggagattgcccagaagtttgggcaattcaaggggaaattggtcaggccacaactgctatactggtcaaggtccactttaaggatcccatctttcctagccagaaacaatatgcctccaaaccagaaattaggaaaggattggaagccatcattgacaacttaaggttgcaaggcctcctcaaaccttgcaatagcccctgtaatatcctgatattggggatacaaaatccaatggggaatggagactagttcaggacctctacctcattaatgaggctgtggttccaattcactcggtgagtcccaatttgtataccctgctagctcaaatacctgagggaatgaaatggttcacagtcctggacctaaaggatgccttctgtatactgttacaccccaactcccagtatttgtttgcatttgaggatccctctaaccaaaccacccagttaacctggacagtgttgcctcaggaattctgagacgcctccacttgtttgggaaggcattgtcaagggacctctccacattcctttatcctcaggttaaagttttacaatatgcagatgatagttttctttgtgctccaactgaagaaatctctcaggagagcagtaaaggtcttcttaattttctggttaacagaggatataaggtttcaaaatctgaagttcagctctgtcagacttcagtgaaatacctaggtttggtcttgtcagaggggaccagggcactgggcaaagaaaggattaagcatatctcctcctttcccctccccaaaaccctcaagcaactgaggggtttcataggcattacaggattctgcagactgtagatacctgggtatggtgaagtagctcgtcccttatatcacttaataaaggagactcaggcagctgaaactcactctgtaaattgggaactgaaggctagaaaggcctttgaccaactctcaactctaacaagccttacttaaagcaccagcccttagtttccccatagggaagatgttaaatctttatgtgtcagaaagaatgggaatggccctaggagttctaaccaaggcctgaggtccatcccaccagcctgtaggataccaaattaggagcttgatttggtaaccaaaggatgactggcctgcctctgtgcagttgcagtgataaccttgctggcaccaaaggctactaagttaccatgggcaataacttaaccatttatactctgcataatgtggcaggactatcttctaaggggagtatctggctaaaggacagccatctcctcaggtatcaagctctgttattagagggatcttcagtacaattaagaacctgcccctccctaaatccagccaccttcctcccagaggaagctggggagcttaaacatgactatgaacagatagtagtacaaacctatgtggccagagaagacctcaaggaaacccccttagagaacccagactggattctcttcatagaaagaagttcttttgtagaataagggatccataaagcagggtatacaatagttactctggatgatcctgttgagagcacctctctctccttgggcacaagtgctcaactaaccaagctaattgtcctcgcgagggcacttgaattaagcaaagggaaagcagttaacatttatactaactctaagtatgctttcctagtcctccatgaccctgccactaactggaaagaaagaacttccccacagctaacaggtcttccattaaatacctccaggaaattaatagactactattctcagttttcctcccacaggaagtggcagtaatacattgtaaaggccaccaaaagggatggatgaagtggccaagggaaataggttggcagaccaagcagctaaatcagcagtgagaaggttccagatttctggtccacttgaagcctgattaatctgggagggccccataagagaaatagaacctcaatattctcctgtggaaacagaatgggccacttcttagggatacatctttcagtcctcaggatggctacaatcagaggatgacaagcttcatctactggctgccaactaaaggaaagttcttaaaagacttcaccaggccttccacctaggtaaggagaaaacctatcaattgtcccagagattgttctcaggtacaaacctgatacaaatggttaaacaggttgttaatgcttgcgagacttgccttaaaaataataccctcaaccaatggcttcttccctcaggaatccaaaggacaggaggctacctggggaaagactagcaaatggatttcacccctatgccaaaggaaaggggcatccagtatctcctagtatggataggacaatttccatgtcagacagagaaagcctctgaggtgacaaaagtactaattaatgaggtaattcctcactttggacgccctaagtaactccagagcaataatggcccctcattcaaggtcattgtcatccaggaagtctcaaaaagcactaggcatacagtaccatcttcatcatgcttggagaccacagtcctcgggaaaggtagaaaagacaagtgatattatcaaaaggcatctcaggaaactgtctcaggagactcatctcccctagactacctgtctccccataaccctactacatagtagaaatatgccttcaaggctaggtttgagtccctttgaagtgatgtatggatagcattttcacaccaatgatttcttactagaccaagaaacctctgatttgattaaacatataacttctttggcctatttccaacaggaactgaaacaactgtcagatgcccaaccccatgaactacgaccatctctattcaacccaggagacctagtactggtaaaagtagtcccttccctttctccctctataagccagactgggagggaacttacactgtacttctttatactcctacggcaatgaaggccactggaatagattcttgaattcactatgcctgagtaaaggcctgggaaaccaacagaattacctctgtcaacccagaagagcagccaaaagtaccaatgggaagatattggggacctcaagctaaaaaatcacaaaagataataattcaccttccatgaatatcctctttatagtcttgcctatgcttgctgttcttaccttcattctgttctataccatgtggtacaatgttgttttcagaataattactatatttcatctcttagttctgtaatctttggcactagattctttccttttaacccctttttgtataatacacgtttgatccatgcatacctaaccttgtaaaacttgtttcttctcacctagaggccatcaaattctaaatggtcaggcaaccagagccttggacaatggctcccttttgccggggacccttaggtagacctgtgggaggaatctgactgctgttctccccaaaacaatgccccctgtcagcaggatgtagctaagactggtcatcatctctattctaatagcagttagatgtgcctcttcagaggtggggaaatgatagggacagagtgcagggaaattctgggcagaagagggcggatccctggcaagcgccccaccctcaagccaaaaagcctgataccacaaaccaaagtgagaatttcacatcactgttttccagctcgaatgtgtcttttccaaaaccactcatggcccgccccacccccatcctgcacccataaaaactccaggctcagctggcagagcgaggagaagcagctggacgttagaggctacagctggatgttggagagaagcagcttgatttcagagggacagcttgacaatgtaccttcagagaggagtccagccaaggacagccggactttagaggaagattgccttcccattctgtcccctttcagctccctttcctgctgagagccactttcatcggcaataaaatcccccacatttaccatcttcaattcattcatgcaacctaattcctcctggatgccaggcaacaactcaggtgccaagagtgtaggtacaaaaggctgtcacactgaccctccactgaggtgttaacacttaagccatccatggacagcaaagcaaaaagggcactg

>mer52_10940_2310_bre_gagputein

lGNGITSsrSLrnChTdFhhgztRLqSHhQckSVpIsPhPLQhlLfpEflmIAilTGVRWyLiVVliAFSYFfFiyKLfLnmFLKKhcNtHSgsSEVtVTccQmLLwgwPhSpLhkGLDKLzSaGRTGLASGRNHSfLcSkTFSvDVrANGLrPhMcrLSLPcrviqTfAnSvGLIqSSyLLSqKRlqGqSqgTKeTnpRGISSrEaSSLqPAfPgPlHPSyPVSLscLPPeiFTLQAPVSLLPLqQMPGeFgHskgqVsFSLqDLrQISrDLGKlSDDPeihRvfpEzpKYLnSPgktLLLLiQTLTNtEKQAALQAQRnlGmSFVSHsvrERvERyPiGREAVPaDaPkWDpNdEmGEWKRRHFQvYImEGLhRTRtnPlNYtKLSmIdQGfDENPTAFLERLREALvKYISLSPDSvEGQLILKDKFIiQAvPDIRRNLQKzaLGPESTLeNLLKVATSVfyniDREaQEReRKHRKeMEALmATmQAHKPQnSzGaPVNzyrCGKPGHfrKDCPGSmRKPPQScPIcNRDhwKmDcLQGHCSlGPePis

>mer52_10940_2310_bre_proputein

wvLGpLspaavvQtTitiqEPqViLKIeGrKVDlLlDTrAglSVLLfSLSTTIrGVSgRPlTQyfPQiVIcswGsllfTHaFLnMPEsPtPLLGRDiLTyTgpppzwpQdkLffsPwGdcpeVwaIqgeigqaTtailvkvhfkdpifpsqkqyaskpeirkgleaiidnlrlqglLkpcnspcn

>mer52_10940_2310_bre_polputein

PdigdtksngewrLVQDlYLinEavVPihSVSpnLyTLLaQipEGMkwfTVLDLKDAFCiLLHPNSQyLFAFEDPsnqTtQLTWTvLPQeFrrlhLFgkALsRDLstFLYPQVkVLQYaDDsfLcAPTEEisQEssKgLLnfLvNrGYkVSKsevQLCQtsVKYLGLvLsEGtRaLgKeRiKhIsSfPlPKTlkQLRgFiGitGFCRLzIPGygEvArPLYhLiKEtqaaeThsvNWElkarKAFDqlstlQALLkAPALsfPIgKmlnLYVsERmGmAlGVLTKazGPshqPVgYqirLDlVtkGzlaCLcAVAvItLLapkAtKTMGnNLTIyTlHnVagLssKgsiWLkdShllRYQALLLEgssVQlrTcpSLNPATfLPEEaGelKhdyeqivvqTyVaReDLkETPLEnPDwiLfierSSFvEzGihKAGytIVTlddpVeStSLslGTSAQltKLIvLarALeLsKGKavNIYTnSkYAFlVlHdpAtnwKeRtspqLTSsIKylqEInrLLfsvFLPqeVAvIHCkGHQKGDeVAKGNRLADQAAKsAvRRfqiSgPlEazLIwEGPiREiEpqySpvEqngPLlrdTsfsPqdGYnQRmTsfIywlPtKGKLKRLHQafHLGkEKTyQLsqRlfSGtNLiQmVKQvVnacETClKnypQpmASSlRnPKdRRlPGerlqmDFTpmPkeRGiqYLLVwIGqFPcqTEkAseVTKvLinEviPhFGrPKzLqSnNGPsFrSlSSrksQkaLGIqyhLhHAwRPQSSGkVEktsdiIKrhLrKLsqEThlpztTcLPitLLhsRNmPsRlGLsPFEvmYGzhfHtnDfLlDqeTSdLikHitsLaYfQqElkQLsdAQPheLRPslFnPGDlVLVkvvPSlSLykPdWeGtYTVLLyTPTAmKatGIflnSlcLsKglGnqqNyLcqPrrAakSTWEdigDLKLknhkRz

**LTR25**

>ltr25_10950_2482_bre_chaindnarm

tgggcttcagtgagtacattctttttctgttttggatgtactgagataaaataacttacacagggtgcttgcctaagacatgtttgcagctgcacagataagaagcattacacagaaccagacatgtctgcaagggaaaattccatctcccaaaacatgctcagtaagggaaataaaacaaaataaaacatgatcagtaagggaaataaaacaaaataaaacaaataaaaaaatagactcaggctaacggcctgtatgcacactggaggaacaaagtggagctgccaaaaatgtatgccttatgcaagtaagacacccagtcttaactatttttgtgtgtgtgtgccttataccaatgaagcatcctgcctcactagcttgttcataaaagcctttgtattcaactgtaaaatgacaatcctctttcgggctgtctctccacagtggagagctttcttctttcactcattaaacttttgctccaccctcgcccttggtgtctgcactccttaattttcttggtcatgagacaaagaactttgggtaatacctcagacaatgggactgctacatctgggaccattggagaggtgagtaagagcagacctccaacactttgctttaatttttgaggcttcttgtcctcagttttctttctctagatcaaacaatacactgggtccctgtcagtccaaaagtgggaaccacagctgccagccttacaagagtcaggagacaagcttgctggagaggagtttgtcaatcccccactgccctcaggtgttggaaatgttagctctgttatgatctagtttcctatcatggtggacctagccatcatatgggactggaaggaggtcctggtgcaactgaaggtttctggctgaggctagaaaccttcagtgttacctgaagtaccctggactaactttggctattgaccacctgtcaaggggtcagcaccaggactcccagtctttcatatttccttattttctttcccctgtggcaatcatgtctcttatccctttgttgtataccatgttgtgggtatttttacaacatggggatataatcttgttgggaaaagtcagaaacttctttagtaatcaggaatgtaactcaaagaattgctgtttttgttgtatgccatgttgtgggtatttttacaacctggggatataatcttgttgggtaaagtcagaaactgctttagtaatcaggaatgtaactcaaagaattgctgtttttgtaatttcctagaaacatgaggacttcaagaattcagtttaaattttcaccaagtaagagcctttttgtccccccagtgagagactttcatggcactatatagggggatattttaccccaagtagaagtgaataccttctgctccatttgttgttgttgttgttgtttcatgtaaaagctcagcactgctcaatgaatctgaacagttcatcatgagacaagttaattttcttctctcaggtgggatactatgatagcctattaagccccaaacctcccaccctttctttctttctttctttctttctttctttctttctttctttctttctttctttcttttctttctttttttctttctctctttctctcctgaaacctctctttctaacctctgcatggaaagaatttggagccatgatttttacctaatgcttaaggtcctactactccacctagtgaaatgggatttttctctgtggtgggccctataaaccttttgccataacctctaattccccaatttctttccctcctacatccctctatcagcaatcaggacctatcccctatttgtagacagaagaactccattttcaacagccagaagaagccatcctggcaagacagattctagtctcagtactgtccttgccaaagggaggacagccattcgacccatatgttattttgagacatttgttctgcatccagctacgttggtatttaaagaaaaagcgttttatgtttagaagtcaatcggtcccactatctggaaatccaatgctttgccagggccatagctagagaaggcaaggatagaattaatataccccccaattaaatggcttgcccaaatccaagttctgtacaatctctcctgggctcctgtggtatcttgggagccttttggatcaattggtctagcagaccaatagggaatccccagttgagagcaagagcatcgcacaggtattgaggactaggtaagcatgactactgccaactagctcctctggatccatgggtgaaggtcatgcttgcatccatggacagcacctatggcagttgctgggacccagagaatacagagagggaaggaggaaaagggggatgccctttctattttctctccaccttgggtcgctccaagaggaggaaggagacaaagggatgctttttctcccttctctttctagatgggtaacaaatcattttcagtctgcactcctctcaagtgcattctgaaaaactggaactcctttgaccctcacattctgaagaaaaagcacatcatattcttttacacttaggcatggctgacttaccaactgcaggatggggaggactggcctcctcagagaagtgttaatttcaatactctatgacaactagatattttctgcagacaggtcaaatggtccgaggttccttatgtgcaaggcttcattgccctgggagataatccagatctttgccaacattgtaaaattggcctggccctcttagctgttatatagggcaaacctgcagtagacaattccccaaaatcagagaaacaaccccctggggaaggctcacattaagctctctgattctccagcccttcctgtcctctctgtccaggaccctctttggccacatcaccagttcctccagttttacaacccccaaaatctttgacttcactattacccctaaaggaaatactgatggcctggtgccactggggtccaggttactttttccttgcaggatcttagacagataacaggggacctaggcaacttctctgatgaccccaatcaatatatagaggctttccaaaacctgaaccaagtgttttaccttacatggagagatgttatgttgctcctaaatgagaccctaagtgctgctgaaaatcaagcagctctgcaggcagcagaaaaattcgaggatgatcaatatgtctcctataaccaatcaagaagaaaataaaatgagaatgaaccataattccaataccctataggaagagaggcagtgccccttgtaagccctagttgggaccccagtgatcccttagatgaatggaaatgaaaacatttttaaatgtgcatattagagagcttatggaggaccaaagtcagatcattcaattactctatgcttttcatgataaatcaaaaactagactcaaattcctccaccattttggaaagtctaagagacttagtaaaacatacctctgtatctcctgactcaattgagggacagctaattttaaaggacaagtttattctccaggcagcccctgttattagaaggaagatacagaaagagggcataggtccagatagcaccttaggaaacctcagccttttacaatagggaacagaaaaagaaaagaaaacacaagaaaagggcagtggctctggtggccacgttgcaggtcaataaaatccacagtctttgagatgtacctgctaactgctaccattgcagcaagccaagacaatttaaaaaggacagccaaggcaccaaaagaaagttaccttgaccccgtcctgcctgtgctggtgaccactggatggcagactgcccccagaggcacaggcccaatgggtccaataccagtctcccacatggtccagcaggactgacgggtcccagggctcaattccctggctccaatggctcagactgccatcactatgcaggagcctgggtgattctggagtttgaaggaaggaaaatagacttccttctggacactggatctggtctctctgtactcctccaatctaggctttctctccttatgcagtacaactatcatggatgtctcaggaaagactttaacccaatgtttccctcaatcccttagttgtagctgtggagacctactgtttactcatctttttaatcattcctgaaagtcctactcatttactaggtggagatattctagcccatatggaagccatcatccttacagatccaaagcaaaccctttgcctccccttagtggaaaccaacattaatccagaggtttggacaacccaaggaagaactggccgagctataagtgttgcacctgtccaaatccacattaaggatcctatcttcttccctaaccagaagaaatatcccctaaaaccagaatctagaaaagggctagaagctattattaacaacttaagaacacaagacctctttagaccatgcaacagcccctgtaacaccccaatattaggggtgcaagaacccagtggggactggagactagttgaagacctctgcctcactaatgaagctgtggtttcaattcatccaatagttcctaatccctataccctgctaactgaaatacctgagggaacaaaatggttcaccattttggacatgaaggatgccttttcctgtataccactgcaccctgactcccaatatttgtttgtatttgaagatacctccaatcagaccatccagttaacctggacagtactgcttcagggcttctgagacagctaccacctgttcacacaggccctgtcaaaaaatcgatttgatgtctctcaccctcaggttgaaattttacaatatgtagatgacatcctcctctgtgccctaactgagaaagcctcttgggaaggcactaaggctcttcttaatttcctacctaacagaagatataagatttcaaagtttaaggctcagctctgtcagacttcattaaagtacctaggtctagtctttttcagtagggaccagagcattaggtgaggagaagattaagcccatttcccccttccccttccccaaatcctcaaagagctaagagtattattgggcattaaaagtttctgcaggttatggatacctgggtatgatgagatagcacttcccttatatcaccttataaaagatagttgggcagctaaaacttacttcttgatctggaaacttgaggttccaaaagcctttaaccagctaaaacaagccttacttaaagtaccagccctcagtcttcccatagggaagctatttattctttatgtcttggaaaggaaaaaaaaacagccttgggagttttaacccaggcttgagatacaactcaacagccagttggttacctaagaaaggaattcaacttggtggctaaaggatggccagctttcctccaaacagttgcaacagtagacttgatggagccagaagccactaatttaaccacggagaatgatttaactggatatacaccacataatgtggtaggactgttatcctctaagggaagtctctggctaacagataattgcctcctcaaatatcaaggttgggtgttagaaggatctgtagttcagttaaaaccctgtccctgcctgaacccagccactttcttccaggagaaagctggagaacctgaatataactgtgaacaggtagtggtgtaaacctatgcagccagggaggacctcaaactcccctggaaatcccagactggactttctttaccaatgggagctcttttgtacaacaggaaatccatgaagcaggatatgcagtagtcaccataaataacattattgagagtgcacctcagctcaactagctgagctaatcacttgagaagcacttgaattgagcaagggaaaagtagccaacatttatatggattccaagtatgccttcctatttctccatgcctatgcctctatctggaaggaaagacacttcctaacagctaatgggtctcccatcaaatatcatcagaaaatcaacaggctactattctctgttttcctcccacaggaagtggcagtaatacattgcagggggtatccaagggggatagaagcagaccagatggctaagtcagccacaagaacgccccaaagtcccaatacattttaagcccctctaatctgggagggctccataagaaaaataaagccttagtactcccctgcagaatggaccacctcttgaggatacatttttcagccctcgggatggttacaatcagaggacagcaaaccactttcaagcctcctgttaatggaagattcttaagctccttcaccaagcctttcacctaggaaaggataagacttcaatgcaccaagaaattgttctcaaaggaaaatctactaaaaatgtcaaacaggttgtcaatgagtatgaaatctgtcttaagaataatccctttaacaggcagctcctcctcccatgagctcaaagataggaagttatacaatggaagactggcaaatggacttcactcatatgccaaaagtaaaaggcatccaatatcttttagtatgagtagataccttcactaactgggtagaggcatttccatgccagacagaaaagacctctggggtaataaaagtaataattaatgaaataattccttgttttggattccccaattatgtccaatgtgacaatggcctctcatttaaagtggctattacacagggggtctcaaaagtgctaggcatacagaaccaacttcattgtacctggaaaccacaatcctcaggaaaggcagagaaaacaaatgatattatcaaaagacacctcagaaaatgttccaaagtaactcacctgccttgggttacccttcttcctatagcttcactaaggataaggaacacctgttcaaagttagttttaagcccattagaaattatgtatgggtggcctttcctcaccaataaccttttattagaccaagaaacttctgagttagttaaacatgtaacttgctggctcacttccaacaagaattaacacagccaacaaaagctcaaccccagaaaataggaccacctttatttaatccagaagacttagtactggtgaatgctctcccctcactttctccatctctaggccccaattgggagggactttacactgttctcctctccaccccctcagtggtaaaattgcaggaatcaactcctggattaatcacacttaagtcaaagcttggaaagcagagggagcaacccctaacagcccagaaaagcaacccagatatcaatgtggaaaaagtcgggggccttaaactgaaaattacaaaagataagtagatgaatgaggacaacttgtccagctcaatcccacatttacccaaccaaactctatcatctatttccatgtttctgttaaaaatctgccatcaagaattacaacttctttttgatgcatatttacagagagactttgattatccatgggaccaagtttgtaacttcgtagacccacagagggaaatctaaaactttaatggataaaacctcagatggaagtcattcactgtaccatacttacaggtattgttttgttcattctacttattgcagtagggttatccactgtgtagcaccttctgagtggagtaccagacaacgaaccttaattgctctaatattttgcttaggtattttcctcatagcagggatagtagttgccaataaaagacaaacatgaagattttgtcactgaagtactttttccccttcctaataagatggtttggtccactatgcaccattatacaacagctacaacacttggcctcagaaaccaacttaacctcttgctacatttgaactttggaatcactattggctgaagtactaccattatcattagatgatttagccaaagttcataccagggtcaccataaccatccaattagaagtaacatatcccaatccaactacagaaaagctacttcttacagtactatcacaagtcataagggatgtcccaacctaaattttatatgaaccccaaaccctgtgagggtttttcacaatgtaacttcaagaagccagttccctctccgcttcacatatcagtggaaaaaaggaattttcttggaaactttatccaacttttcatatgaagtttttcttagacacctcaagaaataccttgtaattcctcagaaaaaaaatcaatttatacctcaaaccagaaaaacaccaccaattccttctcattaaatatccaaacccaagaagacctcagtataccagcaatgagaccactttaaatatttccacaggctgctccacaatattaataaataactcatctttgcccttagctcaaatgttggctaggggttacaaaagatgtttttaaaattttaccctgactcagcttttcattccaacctccaggcacaactggctgcagatgggtctcttatatgggaaagactcactggaacctctgggagctcacaaaatccccctttggaactagcctcttcagggtatgaggtatctttagaccccaaacaagggcaatacttcatatgtggagactcaggatatatgagtctttgaagccaatggaagggaacttgtggcattgttgcaatacttccagaattgtcttatgtcaatgccattgcagcattcctgtttcttataaaacatatctatgccatgataaagacagcaaaccccacagtatttcttcctttagctgctgttgtgtctgaacttctggggaccactcttgggtccacctctcttcatctatcctcccaacaaactcaggtcctagctgaaataatggctgccattcacaaactacaacaacaattagattcacttgcaggtgttaccttacaaaattgtagaggattagatctccttacagcaaatcaaggaggaatatgtgtttttctcaaagaagaacattgtttttgcattaactcctctggcagggtacagcaacatctagtacaagcaaccaatattataactcatctacagaaatccaacccatcagaatggctggcagctaccaaacaaaccttactgtcatggctatggccaacaatacctccattaatagcgatgatcttaatactcatattcaggccctatgttctaaatcttcttacaaaacttacgtcttcctgcctagagactatgaaactccagatgcttctacaaatgaagcccaaaatggagacacctttcatccaaagactcttcaatcgatcccaggaggaaccctggctactttccctgcacaacatccctctccaacaggaagtagccagaaagattgtcacccaacctccctaacagcagtctaggtctccactcatgaagggggaaataagaggggagaaagtaagaaaccagttaggcagatgtttagagcaaagtgcttgacataattctttctaatgaaagagcagcctgacaactcaagctgcaagcatagataaggaagcaaggtccaacataaagcatttttgtctattgtaatcagcacatctcacatacacgcagtgggcttcagtgagtacatctctttccttttatggacatgctcagataaaagaacttacacatggggcttgcctaagacatgcctgcagctgcacagataagaagacttacacagaaccagacacgtgcacagtgaaaattttcctcttcca

>ltr25_10950_2482_bre_gagputein

MfrSQSvPLSgnPMLCqghLeKARiELiyPPikwLaqIqVlYNLSWaPVVlGAFWiNwsSrpigNPqlRAraSHryzglgkhdYCQlApLDPwVkVmLaSmdSTyGscwdpentereggkggcpfyFLSTLgrSKeeGDKGmLfLPSLSRwVTnHfqSALLSSaSeKLELlzPShSeeKAHHiLLhLGmAdLPtAGwGgLASSRSVNfnTLzqLdIfcrqVkwSevPyvqGfiALGdnPdlCQhcKiGGPLScyIGQTcsRqfPkireTTpWgrltlsslilQpflSSLsrtlfghitssssfttpkifdFtitPkGnTdGLVPLVqVtFSLqDLrQItgDLGnFSDDPnQYIEaFQnLnqvFYLTWRDvMLLLNeTLSaaEnQAALQAAekFeDdqYvSynqSRrkznEnEPzfQyPiGREAVPLVSPsWDpSDPlDEwKzKHFzmCILEsLwrTkvRsfNYSMLfmInQKlDSnSStILEsLRdLvKHTSvSPDSIEGQLILKDKFIlQAAPvIRRKiQKEGIGPDSTLKPQPfTIGNRKRKENTRKgQwLwwPRcRSIKSTVfEVPANCYHCSKPRQfkKDSQGTkrklP

>ltr25_10950_2482_bre_proputein

prpacagDhwmaDcpQrhrpnGSntsLphGpaglTgPraqfPgsngsdchhyagawViLEfeGrKIDFLlDTGSGLSVLlQSRLSlLmQYNYhGcLRkdFnpmfPsIp

>ltr25_10950_2482_bre_polputein

VVavETyCLLIfLiiPESPthllggDilahmEaIiltDPkqTLcLPlVETniNPeVwttQgRtGrAiSVApVQIHIKdPIffpnQKkypLkpesrkGleaiInnlrtqdLfrPCnSPcNTPiLgVqePsGDwRlVeDlcLtnEaVVsIHPiVPNPYtLLTeiPegTkWfTiLDmKDAFsCipLHPdSQyLFvFEDtsnqtIQLTWTvLlQGFzdSyhLFtqALSknrFdVsHPQVeILQYVDDiLLcAlTEKaswEGTKALLnfLpNrrYkiSKfKAQLCQtslKYLGLvffsRdqsiRzgEdzAhfplPlPqilkeLRvlLGiksFCRLWIPGydEiAlPLYhLiKdSwaakTyFlIWklEvpKAFnQlKQALLkvPALsLPIgKlFiLYVlERkktAlGVLTQazdttqqPVgYLrKefnlVAkGWPafLqtVAtVdlmepeAtnlttendltgytphnvVgLLssKGslwLTdNcLlkyqgwVLegSvVqLKPcPcLNpATffQEkaGePeyNcTgSGVnLCSqggpQTPLEiPDwTffTnGSSFvQqEiheAGyAVVTInniiEsTSAQlAELITzeALeLsKGKvaNIYmDSkYAFlflHayAsIwKeRhfLTAnGspIKyhqKiNrLLfsvFLPqeVAvIHCRGyPRGieADQmAKsATRTpkSqyILspsNLgGlhKKnkalvlPcrmdHllriHfSaLgmVTIrGQqTTfkPPVngRLKlLHQafHLGkdKTsmhQeivLkgkstknVNRlSmSmkSvLriIPLtgsssSHeLKDrklTmedWqmDFThmPkvkGiqYLLVzVDTFtnWVEAFPcqTEkTsgViKvIinEIiPcFGfPnyVqcDNGlsFkVAitQGVskvLGIqNqLHCtwkPQSSGkaEktNdiIKrhLrkcsKvThLpWVTLLPiAsLRiRNTcsklvlsPleimyGwPFLTnnLLLdqEtSELVkHNLlAHfQqELTQPtKAQPQkIgPPlFnPeDlVLVnalPslSpSLgPnWeGlYTVLLsTPsvVKlqestpGlItlkSkLGkQREqPltaqkSnPDinVEKvggLKLkITkDSr

>ltr25_10950_2482_bre_envputein

iALIFcLGIFLIAGiVLPiKDKHEdfvTEVLFPLPNkmVWSTmHhYTTATTLGlRNqLNLLlHlTLESLLAEVlPLSLDDLAKVHTRVTITiQLEVTYPnPTTeKLLLTvLSQHKGcPNLNfIztPNPVRVfHnVTsRSQfPLrFTYQwKKGiflETlSNFSYEVflRHLKKyLViPqKKNQfIPQTRKTppILLikYPnPRRpqytsnETTLNIsTGcsTiLiNNSSLPLAQmLARGYkRFLKFYPDSAFhSnLQaQLaadGsLIWeRlTGTSGSSqNPPLeLAsSgYEVSLDpKQGqYFICGdSGYMsLzSqWkGTCgIVaILPELsyvNaIaAfLFLIKhIyamIKTAnPtVFLPLaaVVSELLGTTLGSTsLHlSSQQTQVLAEImAAIhKLQQQLDSLAgVtLQNcRGLDLLTAnQGGICvFLKEEhCfCinssgrvqqhLvQAtNiitHlqksNirmaGsyQtnltVmamannTsinsddlnthiqalcsKsSyKTyvfLpRDyEtpDAstNEAQNGDTFhPkTLQSIPGGTLLLsLhnIPLQQeVARkIVTQpPzQQSrsPLmkgEiRgEkVRNqlGrclEqSAzhNSfz

**MER84**

>mer84_10530_3405_bre_chaindnarm

tatgagaaatcacccaggagccatgagaatcctgcccttttccaagctagactggtggaggctatgaataaataaacaaatttagacactgaaaaccctaagggccaatccattctggccatacattttataagtcaggcttcccgagacatcagacaaaaactcccaaaattagataaaggtccacaaactccctttcttacattgttaaatacagcctttaaggttttcagtaactgcgaggaaacatcaaaaataaaaaaggctcaattggaggaggaaaaatgctgttgtcatgctaattacatggtggcagcattggcgcattctttctccttagctaacaaccccaaggcttgtccctataatactaatagtatggaggcctgtcatcgctgcagaaatccaggacactggagtagagaatgtcccaaacctctggcttacaagctgctttcaggaccctgtcttcatttcaaacaagagggccattggaagagcgattgtccctctttccctcatgagatgggaccacctcttctttctgtgctgtcatagccacaaccttgccaacctacctgatgagaaattcctgcagaacgaggactagggcaagggcaaggacaagcacctctaacactattcctgggttatgctcaagactttgaaagtcatcctctagatgactgatggggctgtgaggctatccaggcccctgtcttttccacttctatggatgagcctcagataaatctgatcgtggctgaacaagaaataatgttcctcatagatacagggtccaactttaaacatttattgtaacccaatgtgccagtcctccatttccctcatgggtattgaaggaaaaccccaatgtagctgtttcacactgccactcccttgcaaaatggaaaactcatcctttaactactccttttagtcctgccaagctgccctgttccattattgggtcatgacttactcacaaaattacaagcgaatttacagttataacctcaccttctagctgtattaactcacacttcaccaaaagagccactgcagtcaatagaacctcacattctaaaacaaatgccatttgaggtttggaatacatctgctcctggccgctcaatatcagctgtttctgtcatcattcagcttaaaaatcccaatgagttcctcagaatcccccaatatccttgaaaccagaagcatgaaaatagttaaagcctgaaatgacaaaatttttagcccatggattactgagctcatgcaactcaccttgcaacactcctatttgagctgtaagtaaaccagatggctcctgccaactagtacaggaccttagaataattaatgaagctgttattcctattcatcctatcgtcccaaacccttacaccctttgtagataaattccatctaccacagcttggtttactgtacttgatcttaagaacaccttttcctgcattcctatatacccagatagctattttttttttttttttttttgcctttgaaaggcaaaacccagatactcaaataactcaacagttaacttggacagttctgtgccagggattcagagataacccccgcctttttggacaggccctagctaaagacctgtccaccctgcagcctctcccagatagcaatatactccagtatgtggatgacctactaatctgtagtcctaccaaggctgtttcagaccaaaatacagtatttgtaactaaacaaatttgctaactgtgggtacaaagaagtttcttccaaagtacaaatatctgcacaaagcattcaattttggggtcttagtttaacccccagtacaatgagcctttctagtgccagtaaagatcttaccttaaaatgacaacccctgtaactaaacaatagctttggtcctttttgggtatgtctgggttttgcagaatatggattccttccttcagaataataacaaaaccttcatatgaagccctcaagggaactgaggaacaacctctcttagggactaatgacatgaagcatgctctaaacactttaaaacaggctttaatctcagcccagccttagccctaaaagatctgactaagttcttgcttttgtatgtacatgaatgaaggggaattgctttgggtgtcttagcccaaaatctggggccctctaagtgccctataacatatttttcaaaattttagacttgatttcccagggatggccccctgctttgaagctttagcatcagtggccctcttagtccaaaaaagcctcaagctatattacttgcccctcctggaatcttacggttttgtagcaccaccaataattatttaatccatgttcaagctttggccctgtgccaccttgataattctcttcattatggtgattgtattttgggaacaatagctcccacccaaatcactgtcttgaacataactccaattcagaatttcattctagaaaaaaagggccctaggatttaatgtggccacagttgtgggaactgtctcactcttgccacttggaggttttacttaccatgaaatcacactacaagaacttaccgcctcccttgacatagccttagcaaaaactggcacaagtatatcagcgatagaaaagtctttagactcactaggaggaatggttttttgtaatggacaagctcaaaattacctcctagctgaacaaagaggaatctgtgctgtcatcaacaaaacttgttgcacctacattaatgtatctgaaaaaatggaaactgatacacacacttttccaaagtaaccaagactggaccaaaacctttattgaaatcacatggcttcttccattctttagacctgtattccttgtcattcttct

>mer84_10530_3405_bre_gagputein

MIVSLSiTLsSEcSPCtYTLFICSIPQstSTicNTLSSShThtgVayAtSSTesSENPKnilPlckVAngdLgtTQvhFSfPmSDLSQiQSKlSSFSQdPSKfiqKfQaltiafDLTwQdIfVLLTLkKCkSTTmRyHLtPVrmvIFKkSgnnrcwqgcrEiemLLHcWwecklvhPLwKtvwqllkdlepeipfdPaiPLLgiyPkdykSfYYkdtcTcmfiAALftiADLEPTQMPinDrLDkeNvahiHHGiicShkEECPlqGHgRSGnlhSQqTNTGTEnQTLhvLThKzElsNEnTWtQggehhTPGpVGGwgKrrgraLgqISNmQGLkPrzrvDRcSKppwhMyTYvknLHVLhMYpgTzSKikKkLinFzKkkRkRkimHmvLAQawaDEahacnLndnkAgQKLStTqnPaGSTRmpmpAQTRWgRLDYMiTCLLEGmKKAviKPVNfSKVeKSprShENPAlFqARLvEAmnKzTnLDTEnPkGQsILAiHFIsQASrDIRQKLPKLdKGPqTpfLTLLNTAFKVFSncEETSKIKKAQlEEEKcccHANyMVAALAhSFSLaNnPKAcPyNTnSmEAChrCrnPGHWsRECPKPlayKllSGPClhfKQegHWkSDCPSFPhemGppllSvls

>mer84_10530_3405_bre_proputein

PqpcqptzzeIPaeRglgqgQgqApltLflgyAQdfeshPLddzwGcEaIqaPvfsTSmdePQiNlIvAeqEiMfLiDTGsnfTfIvTqcaSPPfPswVLkenpnvaVShcHslaKWkTHPlTTPlVLPSCpVplLGhDlLtklQanLqLzPhLlAVLthtsPkepLqsiEpHiLKqmPfEvwNtsaPgRsiSavSviiQlkNPneflrIpQYp

>mer84_10530_3405_bre_polputein

ffffffAFERQNpDTqiTqqltWTVLcQGFrdNPrLFgQALAkdlstLQPlPdSnILQYvDDlLicSPTkavSdQnTvfvTkQIANcgykevSsKvQISaQsIqfwglSLTPStmSLssAskDLtlKtTPvTKqzLwSflgmsGfcRiwiPsfRIItkpsyealkgteeQplLgTndmkHALntLkQALisAqpaLkdlTkfLLlYVHezRGiaLgVlPKiwgPLsaLzHifqnfrLdfPGmapcfeALAsVaLLvQKsLklYyLPlLeSYfcsttnNyLihvQALalChLdNSlhygDcilgtiaPtQItvLnitPIqNfilekkGPrFnvATVvgTVsLLPlggFTyheItLqelTAsLdiaLaktGTSisAIeKsLDSLggmvfcNgqAQnyllAEqrGICLSStKlvaPtLmyLkKwKLiHTLFQSnQdwtktFieitwLLPffRPVfLVILLLIfcLcLfiALiKFisSRSqrPPImAmqyqyqPATaTsTymGSldGiwssPMneffIAPHsfhDkeqKreKcNLsLHFasvSsfSsTRyCrISyLLAkSqvLvTrPiLasQTLzRVRiVefiGqkGKNeKqgLsTQpKsLLVcfPPHRLnSRfhPgrGGarLhPtaKGTnFcdsTpvciPLsAQAGwSfSGdPfpPgcLKKKCLNymkdLyKni

**HERVFRD**

>hervfrd_10710_3633_bre_chaindnarm

actatcccctacgggggtctgattgagttccaagctggcacctacttttacctcacccccagtccagggcctctatcccctaaaggagatagcaaatgggtggggagaataattcatgttcacatccccttctccatggcagatctcagagtatgtaaagaaaagtttggccagttctcagaggaccccaataagttcagggaggaatttgtaaaactatgtctgagcttcaccctaacctgttaggatgtgatggtgattttagacaattgtgccaccccagatgaaaagatccagattcgtgaaagggcaagggcacatgctgactcatttgtataggccaatgcctcatatgctgttcatcaagctttggggaagacaatcgctgagcaggacccctggtgggaccttagtgatgaaatagacaaatgtaggatggagcattttattacctacgtggctgaagggatgagaaaatgcatgaagaagcctgtgaattatgacaaggttggggaagtagctcaggctgggctgatgaagaatctcctcagctgtgttacagaagccttcagaaagtatactaattaatatgttaatccagagttgccagaggggatagccttgttggctacgcactttgcattttatcacccagttcactcctgatattagaaggaagttacaaaagtcagagacagggccccaagcccctctgtcagtattggtagagaaggcttttaaggttttcaacaacagggacagggcaaagcaaaggaaaaagatatcaggctgatcaagaaaatacagcttttggctaccttgattagccctccacctcatggcaacccaaaaaggcagagaataccagagacaagagacaattgcccaatgtgggacctaaccaatgcacatattgccagaaggatgggcactggaaaagggattgtcctgaccacctcaaagcagggaggtcagttgactttccagacaggatcctcgtcttggaaaatctctcccatgagggagactgtgccactgactaatggcacccaagaatccacccagctcccacctccatcatcatcactccaggggagcctaggaaaactgaatttcttattgatactggagcagcctattctgtcctaagtctgtaagcctcctccccaatcatgcctgtctggtgataagaatagatggatgatttaaggtaagacaatatacttttcctcctgtctgtgaggctggttctgaagttataactcattcttccctacatattcctgaatgccccactttagtgttcggtaggaatctgctgagcacactgggggcttctatctcatcagaaaaagatagcttcaaggtgtcagtagacaaaggacaagggatcaattttttagcctcactacagctagtggatattaacatttcctttatccctgatgaaatttgaattcaggttaacacagtagtatgggacattacagttccagggttagctgtcagagtttcccctattgaagtaaagctaaaaccaggagtaaaatacgcttggaaaagacaatatcctctaagacctgaatccctaaagggaattcagccattacttaccaagttcctaaaacgtggattaataaaggcccatcaatgaccctgtaacattcccatacttcccatctaaaagccaaatggggaatattggtttgtgcagagttatgagctataaatgcagctgtggcttccattcatctgacagtactgcacttgtatacactgctcacccagattcctggtgatgcacaatacttcaccatgctggaccttaaagatatattcatctgcatacctctgcagcttaattcccaatatatttctgcccttgaatggagggatcccaatatcttagaaaccactcagcacacctggaaagtgctcccacagggcttctgggatgccttcacctatttgggaatgccttggcagccaaattgacagaactagatttcagagtaggagccatattgcaatatgcagatgacctgaggattgccaatctgactaagaaagactctgactgtaatatcattaagaccttaaatttcctggcagaacagggatacaaagtttccttggctaaggtccaaatctccctataaagggcccaaaatctagaatttgttctcactcccagtgcctgagccttggctatggatcagaaaagggcaattgatgtattgctgcccccataaaccaggaaacagctacaggatattattgatttgaccagtttctgctgaatatggattcctaatcatggcttaattgctaagcccctatacaaggtgctgacgaggggaaaagggtacccctgcaaagagataagaattgtcatcaaggttttgaggacttaaaaactgagttaagccaaatcccatcacttgggcttcctaacctggatgagcccttcaccttatatatatatatatgaaaagcagggaatggctttgggggttctaacacagaagctagggccaaaccagtggccagtggcttacaacagtattccattgcccagggttggctcagtggcagctactaccctcctggctaatgaggcctctaaacttaccttgggtcagtacctaaaagttgtaatcaagtccagactatactgggggtgaaggaacaccattgactcatcaagctcttttattagacactctggatgtttcacagagtgtcagattttgatccctgtcactttagtccctgttgcagacatgaaggcctaattcatcaatgtactgagacaacagaatagacctattccattaggcctgaactccaggatgagccccttgacaattcagaagtagaatgctttcctcatggaagtagctttatggaagaaggtgtccaaaaagtaggcttctcagcagaagctcttcccactctgacctcagttcaaaaggccaaattatttgttttaactagggttttgcagctgagaaaagcaaaaacaaactaaacatttttacaactaaaagtatgggtttcatgtcttatatgcccacactgccatctggaagaaaaggggtatgctaacagctaaaaatttcccaataaagcatagggatctagtcttagctcttctggaagctgtacaattccctggccaggtggaagtcattcactgcaaaggccatcggagggacagattgcttattagccaaggaaattagctgataggaccacaaaataagcagcttaagcacagaaagatgaccaggtaatggtactaataatcaaccctttgggacttcccagctaacctcagtactcccaacaagaacaggatgacactgagaagtgggaataggaaaagaggagtgccaggtggttggttaaggacagtaaagtccttatccccgcacttggcagtgaaaatttatttagtacctacatgaagttactcactataggagggatatcttttgggaattagtacaaaatacctcttctggaaaggggccaagaaagactgtaagtcaagtaaccatggcctgcaaattacgtattcaaaacaatccccagactcaactggttcctcttgccctagtcaagccagtccaacactagggaacctaccctgtggaagactagcaaagaaatattacccaggtgccctctcttttgggatagaaataccttctcattttcatggacacttttactggctgggtcaaagcatatccaacccagtctgaaaaggccactgatacttgcgaaagtctaaaagaagtaattcctcaattcggtcttccaaagtcactccagggaaacaatagaccctgtttcatagctaaaatcactcaaagctttttggaagtcctgggaatagattataaatacatgactgttggcatccccaatcctgaggaaaggtagaaaggatgaatcatacccttaaaagaactttggcaaagttatttcaagaaacatagcaaacctggataaggctatttcctattgcactctttcagattcacactgccccaaagagcaacttaaaacccagtccatttgaaatggattacaggaggctcctcctcactactgacattttactggactgagaaatgagtcaaaacatatggtattatcaatgtggatcagattcagaaagcaatattacattttgggaataaagtaccatcaacttctataaaagagggacaatctcctctaaaggtcaacccaggagaccaagtccttcttaagatttggaaagaaggctccccagaggatcaatggttgcctaagtgaaagggccctaggactgggtgatcctggctactccaactgcagtcaagctaccagggatctccagcctctcccaattaaaaggctttcaccagaatgtttctagcctcatccagaaagccccctttcctcccgtgaatgagtggaaggcctccactacaggtttacaagaaccgatagccagatgcaaacacataatgctgtgagtaagcataggaaaactaatttttctcttcaccctaattaccgttttcttgttctaccatctggacagcccctcccactggtaaggaccttttctttctgtccttgctaggtttaaggcaggatcacaatgagaaataacttcctcttcctccttctccacatactcgttgctactagggcccaatgggaagaaaactcactcatcaatttttccaggattatcacctcaaggaacaacctaactgttagatttgccaccacacctacaggtgggaggaactaagctccaatttgtccccactaagaccaatttgacattaaatcttacctctgactatgttgaaaagccatcacctatgactacacaaattgaagtaacagaaagacttgaatgtgtgtgcctcacagacatctataaccaatccatttggaaggagatctaaatgcttaattgtccatccttgacagccccctgttgtccttgtagaaccagtccttgggcaaatataatatatgtagacagttggcgtccaccctcaatttacccaatggcattcaatatccctgaacaatgtcaaactaatactacctattggtaccgcgcctctcatttactaaaacatcctgactggttccaccccatcatctccacagggatgacgtacagattggacaggtatggagagtataacatggaggcccttttgtctacttcctaagggagaaagggcattccctgggtgcaaagacccagaactcagtgggaagacctctttcaccccaatgcagcagcaacattccttcctgagaacctagttcgaataaatggctcaactcctctataccccccatcaggcaggcccagcccctggatatgtattcctttgtagcagtcaaggtcctacaagggagaaacagaccccatcttcccattcaacttacaggccttagcctatccttgtttggacacccccagttccaaggaaaagttaccgtaagacagttgggctccaatagagtaacagtccataaccttacttccaaaattagaatataagagaccaactgagctaatcctggcagaaattatggcagctatgggaagggtagctccctgaggggcttcacctaacatgaaataacttttaaaaaccttacacagattacagaaaagttgaccacaaatataagagaaggcttataaggtctccaggcttccctagaccaaatggtggtgttgtcaacaattggctaggattagaccatgttctagctgaccagggagggtgtgtgtgcagttatcaacaaaactggttgcacttacgtcaatagctctggaaaggttgagaccaatatacaaaaaatatccaaacaagctacatggtaacatagatctaaccagggaactgacccaaattatgtctgatcaatcaccaaaagtgccttcccaagtctcacctggtttctgcccttcttagaaccttaagtagctgtattattattactaatcttcagcccttgtctacttaatcttttagtaaagtttgtgtcttccagattgcaacagtttcagataaaattgatgctggccgaagggtaccagcctgagccactgcaggaaggagcaagtccctataaatcattagattggatagcaagagatttctatgcctctggagcaggtagggatgacagaccctgctcagcatgaagcagttatagaagacaagattttcattcctttctccccctaagagtaatgaggttgataaggaggttaaaacctcttaggggggattgagacattatagagaaaatacttggtccccacagagatatgacaccaaggccccgatgggcctttcatatctgggtgtatctctcccctacctttttgcctcccttaacaagtcatgtaggaggaagaggcgc

>hervfrd_10710_3633_bre_gagputein

MASIIPSLLPGVEKQVLELPIHpLLQSLLIFLTLGLihSNPPaLFQVQGLLwPLNLhhTwirPIRTRgFKArALSTiPYGGLIEFQAGTyfYLTPSPGPLSPKGDSKwVGRIiHVHIPFSmADLrvCKekfGqFSEDPnKfreEfVKLcLSFtLTczDvMVILdncaTPDEKiqIRerARahaDSFVzANAsYavhQaLGKTiaeqDPwWDLSDEiDKCRmeHFiTYVaEGmRKcmKKPVNYDKVgEVaQAGlMKnlLscvtEAFRKYTnzYvNPeLPEGIAlLArtLHFITQftPDIRRKLQKSEtGpQApLSVLvEKaFKFQQQGQGKAKEKDiRLIKKIQLLATlISppphGNPKRQRIPETRDNPNVGPNQCtYCQKdGHWKRDCPdhLKagRSVdfPDRiLVLENLSHeGDCatd

>hervfrd_10710_3633_bre_proputein

TSiiiTPgEPRkTEfLIDTGAaySVLsvSLLpNhAcLVIridGzfKvRqyTfPpvCeAGsEVItHSsLHiPECPtLvfGRnLLsTLgASIssekdSFkvsvdkGQGinfLasLqLvdinisfIpdei

>hervfrd_10710_3633_bre_polputein

ziQVNTVVWDiTVpGLAVRVsPIeVkLKpGVkyAwkRQYPlrPEsLkGIqPlltKFLkrGliKPindPvtfPyfPsKsqmgNiglcRVmsINaaVasIHlTVlhlYtLLTqiPgdaQyfTmLDLKDiFiCipLqLNSQyiSAlEWRDPnILetTQhTWkvLPQGFgclhLFgnALaaklteLDFRVgAILQYaDDLriAnlTkkdsDCniiKTLnfLaeqGYkVSlAKvQIsLzRaqnLefvLtpsazaLamdqKrAIdvllPpzTrkQLqdiidltsFCziWIPnhgliAkPLYkvLTRkrvPlQrdkncHQgFEdlKteLSqiPsLGLPnLdePFTLYIiyEKQGmAlGVLTQKLGPnqWPVAYnSipLPrVGsVAAttLLaneAsKLTLGQyLkVVIkSrLywgzRntiDsssSfiRHSgcfTEcQILiPvTLvPVadEGLiHqCtEtTEzTysiRPeLqDePLDnseVecfPHGSSFmEEGVqkVGfSaEALPtlTSvQKAkLfVLTrvLqLrKaKKLNIfTtkYgFhvlyaHtAIwKkRGmLTAKnfpIKhrDlvLALLEAvqFPgqVevIHCkGHrRDRllisqGNzLigpqnKQLkhRkmTrmvLIiNplGlpszPqYsqqeqDDtEKwEzekrSARWlVkdsKvLiPalGsEnLfSLHevTHyrrdifweLVqNtSSGkgPrktvsqNhgLQiTysKQspdsTgSSCpsQAsptlgnlpCgrlAkkyypgAlSwdrnTfSfswtFTGwVkayPTqsEKATdTcEsLKEvIPqFGlPKsLqGnNrPcFIAKitQSfLevLGIDyKydcwhPQSzGkVERMNhTlKrTLaKLfqETzqtWirLfPiALfqihTaPksnLkpsPFEmdYGGsssLLtFywTeKzVkTygiiNVdQiqKAiLhfgnkVpstsikEgQsplkVnpgDQvLlkiwKEGsPeIngclseRalglGDpgysncsQaTrdLqPsqlKGfHqnVssLiqkaPfppVnewKasttGlqepiArckHiAvSkHrKtnfslhpNyrfLvlPSgqPlplvRtfSfsLLgLRqdhnEk

**PRIMA4**

>prima4_10882_100034_con_chaindnarm

aagaGaaTtaAtagaTaTTTttttttTtttgTTAtaTaAAAATgTtTGTTttAATATtatTTTTTTaAATTTGGAGCCCTCAAAATCATCTaCAAAGAAAGGCATAGACCTGTCTCCTGGGTGTGTCCTTAACTTTGGCAAATAAAtCTtCTAAAATGATTGAGATTTGTCTCGTCATTTTCTTCGACTGACATCTGGTAACCACGGAGGGATCCTGAGTGAAAGTGACCTGGCCTGtGGCAATTTGCCTCTCAGTGCTTGGTACTGGCTTGGGCACCTTATAGCCCAAACCAACAGGAaAATTTGCCGAAGTCTGGGACCTaTTTCCTCCAGGGATCCCTGATTTCCAATATTTTTCAGTTGGGGGTCTGAGGTTTATTtGCTGTTAAAAAAAAACtCCTTTTTTGTGGGAGTTTTTCCACTTGCTTCCATCAAGGAAGGTGAGCTCATCTGCTTCTGCATCGGCAGAGAGTGGTCTCCAGCTTGGGCCCCATCACTAGGTAAGAAACTGGTTTGGGATTCTGTCTTGCAAATTCTTTAAATGACTAAAATTAGCATTAACAACCAGCTGGTGTTAATTTCCTGCTTACACTTAGAGTGCTCAGAAATaTAtATTTTGTGTGATCATTGTTAGTTTTGCTTAACTGTTTTGTTGTTTGTTTCTGtTTTTGTGTGTGTGTATATGTGTATGTTTCAGTCCTTTCCCCTATCAGATTTGACCAACTCTGAACCCTCTAGCTCATGAGTGTGGAATCTTCCACTCTGAAGAAATAGAGCACCTTGCTCCCCTCAGCCTTTCGGGGCATTCTCAGGCAACTGAGAATCACaTGAGGGTGTCTGGGAGAAACGCTCCCTAAGACGTGCAGAGTCTAAATAGGTTTCCCTTTCAGAAGAACATATTTAGGGTCTAATCTCAGCCAGCAGGTGCATATAAGGAACCGACCCCTCCTGCACCTTGAGCCCCTGACACAtTTGTGCCAGGTAGCCATGACACAGGTGGACCAAACCGGTTCAGGGGGTAACGGCCCTGAAAAGCTAGGTCTGCAAGCAGCACATTTTGGGTCCGACACATGTCCCGaCTTGGTCAAATCTGAAGAGGAGCTCTAAATTATGGGGAACAAGGCCTCTAAGACCCCAGCAGCTGCAGAACATAAAGTTtCCCCTTTAGAAACTCTGGCTGGGTATATGCAAAATACTTATGGCAAATtATCATGCAAATATTTAACCAAGTGGACCACTATAACTAAAGCAGATTCTAAGTTACAATGGCCTAAATGGGGATCTTTTGAGATGCCCAAATTAGTGTACCTGTGAACCAGGATGGAaAATGCAGGCACAAAAACTAAGCAACCAGAATGGGAGAGTTATTTTCAGTGGTATCTGGAGAGTAGCAAAAGGGGtGAAGACaACCTCATTTAtAGGAGGtAAACAAGCAACTTAGAAATGCTAATCAAGAACTCTCTAATaTTTTATCTCTCTTAAAGAAATCCTTGGAATCCCTTAaTTCCCaCTTGGtACCtttaTCCACCTACACTCCCACTCTACCCTGACCTCTCTGAACTTCCCAGACCAGATCTGTCCaaTCTTCTCCTGCATGTTTAACACTTGCTCAACAGAAGGCAGTCGGGAATCTGACTTCAAAGACCCCAtCTGCTGAtGATTCCCTGACAGCCCCAATGGCAGGTCTCATCTGGAAGATGTGGAAAAGGGGGATCCCACAGGGATTTCTCTCATGATTaCCCCATTTTGGGAACAACCAGTAACAGGTAGGGGAACCCCAGCGATTGTCTACCAACCCTGGTCAAAGGCTGAATTATGAGGCATAGTTAAAGAATTTCCTGACCCCATAAAGATCCAATTGGGTTTGCCCGAGAATTTGAGCTtAtTATCAGAACCTATGACCCAGGTCATTCAGACCTTTATCAGCTAGTCCACATGTTGGTCTCAGAAGCTAAAGCTAAGGAATGGCTGGAAAAAGCACAATGGTCAGACCCTATAGCAGATTTGACTCCTGAAGGCCCAGTAGAGCCACAACAACCAGCaCCCCCAAATCCAGAAGACAGGTGTAAAGATGCATAGGAACAAGCAACCACTTGTTAAATATTATTCCTTCAGTGTTCCAAAGGGTTGTGGATTGGAATAAAATCCAACAATGCTGCCAGAACCCAAATGAATCAGTTTTAGATTATTTCACAtaTTTTGATAaAACTTTAAGACAATATTGCGGGATGTCAGCTGATTGCTTTGAAAACAaAAAATGATACATTATTAAATGCAAATTTCTTAAACGGACTAGATGATGATTTAGCCACCCTTGTAAAATGCCACATGACAAACTGGGCCACAGCCAGAACTAATGAACTAGTTAACTTAGCTGACCAATTATCCCGCACTACGATAAAAAAGAAAAAAAGAAGATTGCTtGAGTTATGCATTTACAGCTAAAGCAATTAACTTCTAAACCTaTCAGCCCCAGAaAGATTTTAAGCTCCCTCGGTCTGAGAACCCTTCCCTCCCAGTCTGTTACTACTGTAAAAGACCGGGACACCTTAAAAGGGATTGCCTTAGGCTGAAACAAAAGAAAAGGCAGGAGAATGCAACTCAGGAAGACTAGGGATGCTCTGAGGAAGTACAGGGGTTTCACCTCTCCAAATATTCTACCCTGACAAACAAACTGGGAGAGATTAATATAATAATAAACCATGAGCTTACAACTGCCTTaATTGACACAGGCATGACTATATCTCTGATAAATCCCACCTTATTTAGAAACCCATTTGGAGTAATAaAAaAaTTAAATGGTtGGTaTGTTAATAAAAAATTTAtGTTTTAAGTaaAAATaTAtTTataTTTATtaGGTTAGtAAAGaTTAAGTGTGAatGTAGtAGGTAtATgTtTTTATATGtTttGGtTGTAATtTTTTGGGATGATTTAAaATATAATGgAtaTaTTTTTTATAAAAGGTGAATTTTaTaAGAATTtGAAAGaGGAAAAAATAAAATTaAAAAAtGTTGAAaTGTaAAaTTTAGtAtAGTAATGTTAAAAATaATTTGTGAAGGAAAATaAAaTGGAGAgAGAGGAGGAAATaTTAGGGGAGAAtAaaAGAatTGGAATAAAgAatAGaAAAtaATAAAATTtTTAGTTAATTTTTaTTaAAAGAAGtaGaAtaTTGTAAGGATGTgtTATTAtGGTTAGTAAATAAGATATAGGaAAAATATTTAgAtTagaTAAAGGTAGAGATAAAaAAGAAACCCCTACCCAACCTTAAACAATAaCtTCTATGAtAGGAAGCCATAGATGGAATTGCCCCTATCATACAAGATTATCTGAAAAAGaaaaaGGCTCATTATTCCCTGCACAAGCCCCTGCAACAGCCCTATATTCCCTGTAAAAAAACCAAGCGGGAGAGGATGGAGATTTGTGCAGGACTTGAGaGCAATAAACAATATCGTAATACCCAGGCACCCAGTAGTtCCCAAACCACATACCCTTTATCAGCTATACCCACTACCAGCCAGTATTTCTCAGTTGTGGATCTCTGCGGTGCCTTTTTTAGTATTCCTGTAGATCCAGACAGCCAGTATTTGTTTGCCTTTACTTGGAAAGAATGGCAATATATGTGGACTGTAATGCCCCAAGGGTATACAGAAAGTCCCACTTACTTTTCCAAATATTAAAAGCTGATTTAGAGGATTTAATTTTTCCCCAGGGCTCAACAaTCATTCAGTACATGGATGACCTTCTCCTTTGTTCAGGCACACTATCTTCCTCCCAGGAAGATAGTCTATATTTACTCAAACAGCCACCAAAGGACACAAaGTGTCCAAAGACAAACTTCAGCTATGCTTACCaCAAGTTAAGTATTTGGGGCATATTATCTCAGTCAAAGGACTGAGTATTAACCCTGACAGAGTGAGAGGAATTTTAGCTTTCCCAGTGCCCGTCACTAaaAAACAACTTAGAGGATTTTTGGGCCTGGCAGGCTATTGTAGAAACTGGATACCAAATTTCTCCCTTATGGCTCAACCTCTGTATGCATACCTAAAAAATGAACAAaCTGATCCCATCATGTGGACTCCAGAGGGACAATCAGCTGTACAACAAATAAAGGAAATTCTAACTAATGCCCCAGCCTTAGGGCACCCAAACTAAAATTGCCTTTCTCCCTTTTTGTACACAAAACTGGAGGTACTGCATCCAGGGTACTGATCCAGAAACATGGTGATCATCAGAGACCTATAGGCTATTATAGCCAACACCTGGACCCTGTGGCTTGAGGGCTGCCTCCTTGTGTGAGAGCAATAGCAGCCATGGCCCTTCTGTACAAGTCTGTTGAAGAAATAATTATGGGTTCCCCCCTTACCATTTTTGTGCCACATTCTCTTGAGACCCTTCTAAACTCTCATCATACTAACATgTGTCTGTCAACCaGTTAGCCTCTTATGAAATTTTGCTTTTATCATCTTCCAATATTACTATTTCCaGTGTAATAATCTTAATCTGGCCACTCTCTTGaCAGGCCCTaCCGACAAAACCCCTCATGACTGTGTTCTGATGACTGACaGACTTCTCACCtCCAGGACAGACCTACAAGAGACGCCACTGGATAATGCTGAGATAGAATGGTATACAGATGGGTCTTATTTAAGAGGAGAGGATGGAAATTTTAGAGCAGGATATGCTGTGGTTTCaTTACCAGAGGTAATTAAAGCCAGTCCTCTTCCCCAAGCCAGATCAGCTCAAGTGGCCGAATTGATTGCCCTGACCCGAGCTTGTCAATTGGCAAAAGACAAGGCTGCAAACATTTACACTGACAGCCACTATGTTtTGGGTTGCTCATGACTTTGGGATGCTATGGAAAGAGAGAGGATATTTAACCTCCTCAGGGCAACCCATAAAAAATGGACAACAAGTATCAGAGCTGTTAGAAGCTATTCTAAAACCAAAATGTTTGGCAATTATAAAAATCCCAGGTCACTCAAAATTAGACACCACAGAAAGTAGGTAACCAATTGGCTGATGCCACTGCTAAAAGAGCAGCATCCAAGCCACCAGCCCCAATCCAAGAAATGGCCATAAAACCCGAAACACTTAGAAACATGTTGAAAGAAACCCAGAaAtAGCTCCTACAAAAGAGAAATCTACTTGGAAACAGGCAGGGAGATACTTGTCTCCCGAAACTGAAATATGGTGTGGACCTAATAATAAACCATTATTCCAATGGGATGTCAGGTGCCCCTTATGGAATATGTCCATAATCTAAaCCATTGGAATCCAGATAAAATGATATCCTGGTGTAAACAATATTACTGGAAACCATCCTTCACAGTGGCACAAAAAGTTTACTCTTGATGTGTTATTTGTCCCAAATATAAAGGAAAGCCTATGGGGCCCAGGGTCATTTTTCCCTTCTGGCTGGACCTTTTGAGGTATGGCAGCTTGATTTTATCCAGCTGCCATCATCTCAAGTTTACAAGTATGTTTTAGTAATGCTCTACATGTTTTCCCATTGGGTTGAACCTTTTCCCTGCAGGCAAGCAACAGCCATGGCAGTTGGAAAAATCCTACTAGAAAAAAaTATCCCACTGTGGGGAGTCCCCTGTGAACTTCACAGTGACAGGGGAACTCACTTTACTGGCCAGGTTATTCAAAATATTTGTAAAATTTGGCCCATATTTCAACATTTCCATTGTGCCTACCATCCCCAATCCTCAGGCCTGGTGGAGAGGAAaTGGAATAATTAAaACACAATTGGCTAAGTTCACAGAGGCATTTTACCTCCCCTaGCCCAAAGCACTCCCCCTAGTGCTGCTTACACTCCAATCCACCCCTTTTGGAAAACaTAACTGTCCCCTTATGAAATAATAACGGGAAGGCCCATGTGTATGGGAACAAACATAACCAACCAACTTTTCTCAAGGGAGATATATTGCAATATTGTGAGGGACTTATTTATCACCTTAAAAAAAGCCAAGATTTGGTAAAGAATTTTTCATAGTGTGCTCCCTGAAGATAAGGTACCTGGTCACAATCTGCAACCCAGAAATTTTGTCTATTGGAAAAGACATCTAATAAAGAATTCCCTTCAACCCCGATGGAAGGGCCCATACCAGGTACTATTGACTAATCCATGTGCCACAAAATTAGAGGGTATAGACTCATGGATTCACATCTCTCATCTTAaAAAGGCACAACCTCCTGAGTGGACTGTAACTCCCACCAAAGACCTTTGCCTCCAGTTCACTAAACATCGACCTTCAACCCAGGATTAGAAGCAGATGACAGCTGTTGTGGACTGCTTAAACCCAAGACACAGGACCAGGCCTGTATACAAAGAAATGCCTATGTTTATTGTACAATAACCATTACAATTATTGTCCTAGATATACTGTCAACTGCTGTCTaATAAAGAACAGGGCACTTGCtTTGTCTGATTTAACACCCTTTAGTAACTAAGATGAATTTCACAACCTTGCTATTATTAATCATGTATCCCTACACCTTCTTGCCAgTGtAACTGATGCCCATGAAACAAACCTGTTTCTACAATGGGCTCAGGATTATGCAGACAGATTACAAAAGGACACCTGCTaGATATGCGGACTCATGCCTCTTTCCAGTGGCTCCaGCCTaCAATGGTGAGTATCCTTCTTAAGGTCAGGACTGGATAGAATACCAAAAATtTATTACATAAGAAAaGTCTGGTATTCTTAGTGCTGGCATAACAAAAGACAATATATATAGTTGGCCCATTAAAAACACTCTTAAGAACAAGGGACATGGGAAAAGTTTTTCAATGGAAAGGACCAGCTCATTAGCTCTCACTTTAGCATTCCCCCAACTAAAACAGAAGGTGGTAACCATGCCCCAAACAATGGCCCATTTTCAaAATGGAATAATGCAAATTTGGGATGGATTTATCTGGCTCACCCCTTCGTTTGGCCAACTCAGtAAAATGtTTTTGTGTGGGAtAAAGAAATAAaAGGATaTGgCaAAACAGTAtGAGAGATATGGGGTGGATACCTGGAGAACATTGTGACCACATTATCATATTATAAGACACTGACTGGCATGCCAaCAATTGGGTGCAGTGACCAGaTATTTATTGGCTAGCTCCAAATGGGACATATTGGCTATGTGGCACTAACTTATGGCTGTGGTTACCTCCAGGGTGGTTAGGATGATGTTCCCTAGGTTATGCTTGGGCACAAGGACAAGTAATTCAGACCCTGCCAAAACCAGCAAACCTTTTTCATTTACAATCTTGTTGGACACGTTCAGTATTCCAATGGTATGATCATTTAGCTTCAATCTTTGTACCACAGATAGGTATTGAAGATGTTATATGGCATATAGAGGCCTTAACGAATTACACCCAAAAGGCCCTGAATGATGGCTACATGAtTATCTCTTTGCTAAACGAaGAGGTCATGCTTATGAGGAAAGCTGTGCTGCAAAATCATATGGCTTTAGAaATACTAATGGCAGCACAAGGGGGGACTTGCGCCATATAAaAACTGAATGTTGTGTGTATATTCCAGATGAATCAAAGAACATAACCCAACTTATGACTGATATGAAAACCCAGATAACCAACCTGTCAGATCCAAAACCCTCACTGATGATTGGTTGAGCAGTTGGTTTGGATCCTGGGGAACTTGGTGGCAGAAACTATTGCTTATAATAttAATAATAATAATAATTTGTGTTCTGTCCTGTTTTTGCTTACAATGTTGTTATGGTATGTGCTTGCAAATAAGTCaATGTGCAACTGAAAGGGtAGGGTAATaAtTGCCCAGAGAATTGCTCTAATTGAGGAGGCAGTAATATAGCCTaACCCAGCTTCCAGGTTTGCTTTCCTTTTGTTGCTATAAATCTGGCCTAGGTCCCTATATATTTTCtTTTaTaTCTaTaTaTaTgTaTtTaTaGTaTatatatATaTatatatatttaaatatAtataTatATaTaTTTTTTaCCTTCATGGGAAAGACTTCCTAGGAATAAGCTTCCTAGCAAtGTGGGACATGAaCTCCTAGGAATGAGCCTTCCTAGTGACGTGGGACCTGAACTTCTAGGAATAAACCATCCCAGCAATGAGAAACCAGCAAaaaAAAAAAGAAAGAAGAAGCAACCTGAGGCCAGGAACtCATATTCTTTTTAAAATGCTTTTCTCCAAAAGATTTTAAAGAAAAAAAaGGGGGGAAATGTGAAAGGAAATTAAATTTTGGGACCCCAAACTCATTTAGCCAAAGGGAAAAGTCAAGCTGGGAACTGGGTCACGCAAACCTGCCTCCCCCTTTTGGTTCCTAAATAAGATGGCTACAAGATGAAAAGCTACATGCCTCCCCCATATTTTGCCCACAAGGAAATTCCTGGTGAGCTGTTTAAAACTTCACCAtGGCAATGCAAATTGATAGCTTATCTTTACAGGTGCAGTCACCCTAGCCatAAATggTATCTGATTGTTCCCTACCCCATTTTGTCTGtGTTATCTTATGTAAAATGCAGATTCCCCACATTTTTCCTCTGCCCCTTTTGTTTATGAAAaCTCTGTTCTCAATAaTCCaCCCTTTCCCCTTTAAATCTGGGGCaCTCAAAATCATTTTTaaAGAAAGGCATAGACCTGTCTCCaAGGTGTGTCCTTAACTTTGGAAATAAATTa

>prima4_10882_100034_con_gagputein

mankaskipaaakhkvpplgtlarymqntygkzsckyltewttvtkadsklqwpkwesfdaqisvpvnqmgNAGTKTKQpEWesYFQWYLESskngedalvslQegNKQLgNANqELSNilSLLKKSlgSltfpLgPlPPPPiLaLYpDLSELlgpDLfpPSpaCLTLAQQeAVGNLTSKTPpadnSLTvPMAvfigsvgKGgptgislmiaflilgpvsnRzGtPvsvlpplvKgelmgivKaFaDPiKiplGlaeelsLlstpmtpgtsilislvtlLVlEAKAKEWLEKAqWSDPIADLTPEGpvEPpttSAPlniqdggidawenttpLlnIlflvfskgvwigiisqcatnpstsvlyiisCfDkTLRqsCGMSaDCFEntKnyiLlNANFLngLdDDlATLvKcnMTsWATARTNelVNLaDillrimiKeKtedwpslalqaKaltfsTaQsQiDFkLPqSENpfLPVCYYCKRlGHLKtDglRlKQKKRpENaTqED

>prima4_10882_100034_con_polputein

PfHLwSQSNTDIGKIFSaipvKVEInPKETLvQLZTIvSMtgAvDGIaPIItDYLKKglLIIPCTSPCNSPIFPVKKPSGRGWRFVQDLRAINNIVIPRHPVVPpPlTLLSAIPTTSQYFSVVDLCGAFFSIPVDPDSQYLFAFTWKEgQYMWTVMPQgYTqSlTYFSQILKADLEDLIFPQGSTlIQYMgDLLLCSGTLSSSQEDSLYLLsQPPgDTvCKtKFQvCLPQvKiLGHIISVKGLSINPDRVRGIvAFPVPVTmKQLRGFLGLAGYCRNWIPNFSLMAQPLsAYLKNEQpDPIMWTlEGQSAVQQIKEILTNAlALGHPNYKLPFSLFVHKTGGTASRVLIQKHGDHQRPIGYYSQHLDPVAZGLPPCVRAIAAMALLYKSVEEIIMGSPLTIFVPHSLETLLtLivlsaSVNlLASYEILLLSSSNITIScCNNLNLATLLlGPaDKTPHDCVLMTDQLLTPRTDLQETPLDNAEIEWYTDGSYLRGEdGNFgAGYAVVSLlEVIKASPLPQARSAQVAELIALTRACQLAKDKAANIYTDSHYAvGAHDFGMLWKEgGYLaSSGapIKNGQQVSELLEAILKPKCLAIIKIPGHSKLDHRKSGNQLADATAKRAASKPPAPIQEMAIKPETLRNMLKETpslAPaKENLLGNRQGnTfLlKlIwSGPNNKPIIlvGCQVPLMEYVHNLsHWNPDKMISWCKQYYWKPSFTVAQKVYSmCVICPKYNPgnPLHGAQGHFSLLAGPFEVWqLDFIQLPSSQVYKYVLVMLYMFSHWVEPFPCRQaTAMAVGKILLEKiIPLWGVPgElHvDRGTHFTGQVIQNIvKiWPIFsHFHCAYHPQSSGLVERTNGIIKTQLAKFTEgFSLPlaKALPLVLLtLqSTPFGKnQLCPlEIIvGeAHVmGTNIiNPTFLKGDILQYCEGLIasLKKSQDLVKNSFHSVLlEDKaPGHNLQPRnFVYWKRHLIKNSLQPqWKGPYQVLLTNPCAaKLEGIDSWIHISHLiKAQPPEWaVTPTKDLCLQFTKHRLlTQi

**PRIMA41**

>prima41_10720_100035_con_chaindnarm

TGaaCTCATTaaTCCTGaTAtCTTCAGAAAaaTaTCCTACCCTGTTTAaGaAGAAGTTGTCCTTTaACaATACAATCTaAATaaTTTTGAACTTTTaTTTCAACATCACaGACTaaCaaTAaaTTATCCCTaATACTGGTtCCAGAGCACTTTCCaCTAaGATaTaTCCTTGTAtACaAAaAAaTGTTaaAaATAAAaGGGGTtAAGaaGagtTAAaTTaAaGAAaAACCTTCTCTATaTAaTAAtTGGCTGaCCTTAGTAagCTaaGTGtATAAACTAaatAAGaAATGGtaTTCtTTTGGAGGCaCAGCCTGaAaTAaTTAtAGTATATaATaAAAtAAttaataTTTGAGGTCaTaaTataTAATAaAaTaTAATAATaTaaGaCTAATaTTatAtTAaAGTaCaAaCTAAtaGACTTaGTGAGTtAGAGGAAACAttaAaTGAAATACCAaTCGGCCaAAAaCAGGTTTGttGCTGCAGTCTATaTTATACCaaGTAAaaaGCaTaaTCaTGTTTGATGCAaGAAaaGGAAGaATaGGAGCTatACCTAaTACACAGTCaCTTaAAGaAaaCTCTtagaaaagAGCAAACACaTTTTTatGGAtTTGAACTTACTtCTCTCaCTATGaGGGTTCaGGTtTtTAGtaCATTgTGCaTTAGAaCCACTTGGAAatttgTaAaAatCATTAATaGCTTTGTCTTTACAaaTGTTTATTaCtATTaaaTATttAaCAATCCCTGAGCAACaTaGCTataTGGATaaaatttGTGGTTGaGtCTtTTGTTaCattgaAatttaAataTATGTCAaCTAAGTGaGaTtAaGAAGtACtAGGATGGAAATCAGGGGAaTaTtCTTGCTGTTTTGTTTCAgTTTatACACTGaaaAAAAAAAAGCTTCTTTCTTTTCTTGGATTCAGGCAAATTGGCTTTGTTTGTCCAATTCACaCTGCCACTATTGCCCAGAACCTGCaTaCTTGGTCATCCCCATCTAAATCCTgTtaCATTTCCTTTGCCTTATTtGaACATCTTTGaTCAACTCaaATaagATaTTGATTCATGGCCTAGCtaTaATTTATATTATtTGGaaAATACAAATAAGGGAATTCAAATTTtGACAGGaATTCCaTCATaAATGCAGCGGGCCTTAGGAAGgCTCTCATCCTTTTTaaTGGAACTTGGCCAGTGaCAATACAaTCaCACAGGTTTGGAAtTTTTCTTTCAACATTATCtGCCTCCTTCATGGGAAAtACAGCATCGATTCCAaAGaACTCACCACTAGGATGTATTCTTGAACACTGGCACaAGTTTAAACTaAATGGACTTAAGAAAAGAAAaTTGGaGGTTCTATATAATACTGTCTGGCCTTGATATtTATTTGGAAAAACAAGAGAAAaGGTCaaTTACTAGAACTATGGCCTTTAATATTATACTTCaACTtaATTTGTTTTGTAAGTaaGAGGGAAAATGGAATaAAATGCAATATGTTCAGGCATTTTTATTGCtTAGTCAGaATAAAACCCTGCAAaAGGTATGTGCATGTTTAATGAaAGGAAAAGAAGAAAAAGAGaTAGACATAATTGATGATCCaTTAATGCAAGACCACTCCaGTTCAaTaGGCACTaTCAAaCGGAGAAAAtGTaCTTCTGTCTGaAATAAAtGGTTCaGATGATatCGGTCCATTATaaaaCaCTCaaAtaCACTCagAAAGTCaTATTGAGAACCCTTTATCCCCTCTCaTTACCaATCTAaTCCTAtTCTATACCaTATACTCCCTGAGGAACCTAGCCCAGCaAGTACTACTTATAGTGGAGCCTCaTATCAACCTCCAAAGGGAAATTTTTGTCaGCTTAGAGAaGTGGCAAATGGGGAAGAAGGCACTGTaAGAGCaTATGTTCCCTTTTCTATGTaTCATTTGACTATGTAaAGAGAAAGTTGGTtATTTCTCTGAAGATCCAGGGAAATTCATAGATGAGTTTaAGAAATTAACTCTGACtTATAaTTTAACTCGGaAGGATCTGaATGTTTTGTTGTCTGTaTGTTtTACAGTGGAAGaGAAACAATGCATTTTGGGAACaGCTAAGACCCaTGaAAATGAGGTACTGGCTTGaAACCCAAACCATTATATaTATatAaTgaTATCAGGCAGTAGGTGTAGCAGTaCCTGATCaAGATCCAGaaTGGAACTATCAAAGGGGCAGTaAGGACTTGGGGAaGAGAGATCCTAGGGTCATTTaTTTtTTGGAAGAGTTaAAGTAAtgTATGAAAAATCCTGTTAATTATGAAAAGGTTAAGaAAatTTCTCAttGaAAAGATGAGAATCCAGCTGGCGTTTAGTTAAGGCAATCAGGaAATATGaTAACACTGATCCTGCCTCAAGAGAATaACAAACTCTTTaGGGAGTACATttTTTTATAACTCAATCTGCCCCTGATATCCATAGGAAACTaCAAAAAGCAGCTaTGGGTCCGCAGACTCCTATGaAtCAGCTTTTGGATATGGCATTTTTAGTTTTTAATAACAGGGACAAAACAAAGGAAGCAGAAAGAGCAAAAAGGACCTtCCACAAGGTGCAGCTaTTGGCTGCAGCCTTAAGtTCACCTCCaACATGGGATTGCCCTCCTGGCTCTTAGCCTGAACAAGGGAAGCTAAAAGGTGGAAAGCCCAAAACTGGGCATCCAAGTCACtATGCCTTGGGCATAAATCAGTGTGCACATTGTAAGAAAACTGGCCATTGGAAGAGGGAATGCCCAGTGTTCtAAAGGGAGCCATCAGCACCTGAACCAATGATGGCTAAAATAGCCAAGCAAGCCCAAGAGTGATGGGaTgAGACCTTCTGCCACAGCTCCCATTGGACAACTAACCATATCTCCAGAGGAGCCTCAGGTAACCCTTGAAGTGGCAGATAAGAATATTAACTTTTTTTTGGATATGGGGCTGCTTACTCTGTTTTGACCCATTATAATGGGCCTCTGTCACCCCAAAACTGTATGGTCATGGGGATAGATGGACAAGCCCATAGATGCCATTTTACCTATCCTTTAAGCTGCTCTTCAGGGACTTTAGTTTTCTCACATGCCTTTCTTATCATGCCTGAATGCCCCACCCCTTTGTTGGGAAGGGATTTGTTGACTCAGCTGCAAACAGTGGTATCTTTTGGAAATCACAAGGCAGATGAGGGATTGCTCCTTCTCCTTTCCTGTGATAAGGGAGGAAAGTCAATAGGaGACTTATCTACTTTaTAtTGAAGTAAtTCCCAAGTAAATCCTATAGTATGGGACACTGAGGTTCCAGGCAAAGTGTTAAATGTTCCCCTGaTTTGCATCCAACTTAAGCCTGATGTCCTGTACCCCTGGAAGAGACAATACCCCTTAAAGCCAGAGGCACAAAGAGGGATCCAACCATTAATAGCTAAGTTTTTGCAATTTGGGTTGTTAAGaCCCTGTGAGTCTCCTTGTAATACaCCAATCTTGCCAGTTAAAAAGCCAAATGGAGACTATAGATTTGTTCAAGATCTTTGAGCTGTCAATGGGGCTGTCATTCCCATACATCCTATAGTGCCCAATCCCTACATGCTGTTAGCCCAGGTCCaTGGGGATGCCAATTGGTTTACAGTCTTAGATCTTAAGGATGtTTTTTtTTTTTTTTTTTTTTtTTTGCATTtCAGTACACCCTGATTCACAATTCATCTTTGCTTTTGAATGGACTGACCCTGATAGTCATTTGGTTTATCAATTAACTTGGACAGTTCTTCCCCAGGaGTTTAGGGGCAGCatATtTaTTTGGAAATaATTGGCTAGAGAATTAAAGATGTTACAATTAAATAGGGGCACTATTATTCAATATGTGGATGATtTGTTGGTTGCTAGCCCAACCAAAAGAGACTCAGATGAAAATGCCATTAAGTTGCTAAATTTTCTGGGAGCTAATGGGTATAGGGTCTCACaGCATAAGGCCCAGATTTCAACTCAAGAGGTTAAATACTTAAGATATGTCCTAACCCCTGGCACCCAGGCAATAGCaCCAGAAaAAAAGGAAGCTATCTTGGGCATTCCAGAACCCCAAACTAGAAAGCAGCTGTGGGCTTTCCTAGGGATGGCAGGaTTTGttaTTaaTGaGTGTGGATTTGGGCATATGGCCAAGCCTTTATATaAGGCTCTGAAAGGAGCAaATGTAGATCTTTTTGAATGGAATAGCAATTGTAAACAAGCTTTTAATGCTCTCAAGGAGAAATTGGGATCAGCTCCAGCCCTAGGGATCCCTAATCTTGATAAGCCATTTTTCCTTTATaTGGCTGAAAAACAAGGAACaaCtCTGGGTGTCCTTGTCCAGAAACTGGGAGATATCCCCGAACCAGTGGCATATTTTTCTAAGCAATTAGACCATGTCGCTTTGGGATGGCCTGGATGCCTCAGGGCAGTTGCAGCAACTGCTCTTTTGGTAGATGAAGCCAATAAACTGGCTTTAGGACAAATCTGGAGGTTTTAACCCCACACCAAGTACAAGGAGTCCTAGAAGCTAAAGGACACCAGTGGATGACAGGGGGACACTTATTGAAATATCAGGCTTTGTTGCTAGACACTCCTGATGTAACCCTTAAAGTATGCCAGACTTTAAATCCAGCTACCTATCTGCCTGAACCCACAGGCACCCTAGATCATTCTCGTATACAAGTaATGgAgAAGTTTATTCCAGCTGGCTGGATTTAAAGGATGAGCCTCTAGATAATCCTGAaGTAGAATGGTTTACAGATGGAAGTAGCTTTGTGCACCAGGGAAACAGAAAAGCTGGGTATGCTGTTGTCAGTCAACACAAGGTAATTGAATCTCAGGCCTtACCAGCTTCTACCTCAGCTCAAAAGGCaGAATTAaTGGCTCTTATTAaAGCCCTGCAATTGGGAAAGGACTTAAGAATTAGCATTTaCACTGATTCTAAGTATGCCTTTCTGGTGCTTCATGCTCATGCTGCTATTGGAAGGAATGAGGACTCCTAATTGCTAAGGGTTCCCCTATAAAACATCACTTAGAAATTCTAAATCTATTAGATGCTGTTTTGCTGCCCAAGGAAGTAGCTATAATCCATTGCAGAGGGCATCaAAAAGGAGACTCTAGTGTGGCTAAGGGAAACTCCTTTGCAGATGCAGCAGCTAAGGCAACTGCATTAAAGGAGCCAGTTGGACTTGTAGGTATGTTAGTGCCCTCTGCCCCGGTAATGACAGAACCaAGATATACTAAAGAGGAAaAAGAATGGGCTAAAGGTCATGGTTTAATTCAAGATCCTTCTGGCTGGCTTATCAATGACAACAAACTATTGATACCAGATGCTAATCAGTGGAAAATAGTTAAGCATTTGCATGACTCTACTCATTTGGGAAGAGATTCCTTGTTTCAATTAATGTTTCATCTTTTTATAGGAAAAGGCTTACTTAAAACAGTAAAGCAGGTAACTCGGGCCTGTGAACTaTGTGCCTGGAATAAaCCAAATAACCAAtCTTTACCTTCTCCTTTAGTAAGGCCTGTTCAGCATAGGGGAACGTATCCCAGTGAAGATTGGCAAATAGACTATACTCAGATGCCCCCATGTAAAGTGTTTaAATATTTATTAGTATTCATTGACACCTTTACTAGTTGGATTGAGGCTTTTCCTACCTGGTCTGAAAAGGCAATtaAGGTTTCTAAACTCCTATGAAAGGaAATAATTCCTAGATTTGGGCTGCCTAAaAGCTTGCAGAaTAATAATGGCCCATCTTTCACAGCGACAATTACCCAAAACATATCTTCaGCCCTAGGAATTCAGTACTGCCTTCATTCAGCATGGAGGCCAGTCTTCAGAGAAAGTAGAAAGAGCTAATCAAACTTAAAAAGaACTgTTGTAAAtTATGCCAGGAAACATCAGAAACCTGGCTGTCTaTATTGCCTGTAGCCTTGTTATGGGTTTaAGCTGCCCCCAAAGGAAAtCTACAGTTCAGCCCTTTaaAAATaATGTATGGAAGGCCTTTCTTAACTACAGACTTCCTAATAGACATAGATACTTTCAAGTACAAAATTATGTAATCAACTTAGGACAAGTGCAAAAaGCACTCCTTGAATATGGAAATCAAAGACTTCCTTCCCCTACTAAGGAAGAGAATaTTGTTATAACCCAGCCaGGAGACTGGGTCCTATTAAAAATTGGAAGGAAGGATCCCCAGCAGATCAACTTTCCCAAAATGGAAGGGATCCTATCAAGTTCTCCTTAGTACCCCAACTGCAGTTAAACTTCTAGGAATAAACAGCTGGGTCCACTTATCTCGAATTAAACCTGTCTCTTATGAAGTCCCACAGGCCaACAGAACACAAGAGACTGATATTATTCCTGTGAGCCAACCAGTGACCTCTGGCTCCTGTTCAGAAGAAACAAAAGGGATGGGTAACATAAAGATATGGATTGGCATTCTACTTTTGGGTATAAGTTGGAATCACACAGAGAGTAACTTATTTGCTAAGTGGGCAAGACTTTAGCCTCTCTACATAATCACACaAAaTGTTGGGTATGTGGAGAATTGCCACTTTCCTCCACTTCCAGGTTGCCCTGGCATATTCAACCAGCCAACCTAAGTTTATGGGGATTTTATTATGATTGGGAAACTGAACATTATAAACATAGaCCCTCTTTTCCCATGTACCATAGCCACACAGGCCTTAGCCCCTTCCTCTCaTATAGAGAGACAAGAAGGCACTTTTTTaATCTAATTAGGAAACAGCTAAATTCCACCCCAACTTTAGGTTATACTGTACAtAATGGACTTGGGTGGATGACAatTGTTAAGgaAGGTATCAGGCAAAGCACCTCTATGTTTTGAAAGGCACAATAATAGTCACCACCAGACTGAAACCCAtGATATGGGATGGTTGCCACCTCAACAATGTAATCAGACCCTTCTTtTAACAGACCAAATGTGGaTAGGATGGCAACATAATTTGCCAAAAATAGGTGCCTACCCTTCTCCTTGGGGATGGTTATGGGCTTGTAGAACTCATGGCTGGCCATACTTACCTTATAATTGGACTGGAAGGTGTatGTGGGGTTGTCCTTATCTCCTAGGACaTAaCCTCACCAAATTGGACTCTCTGCCATCTAACTGGGAAATTGTAAAGGCTCGCCATAGGCGACAAAAACAGGCATCTTGGTGGTTCTATCTGATGGCTGTATTTTCCCCACAGGCAGCCATAATCAATAtgaAGTTAAAGTTGAAGaCaGCCAAGCACACAGCTGCAGCTTTCAATAATACACACCATGCCCTTACCCTCCTAACTGAGGAAACTTCTCAGATTAGGCAGGTAGCCTTACAAAACCATATGaCTTTGGACATTTTAACAGCaGCCCAAGGGGGAACTTGTGCTTTGATCAAAACCAAATGTTGTGTGTATGTTCCAGACTATTCACATAATATTACCCAGGCTATGAAAGCTTTAGACACTCATATCTCTGCCACTGATGCACTGTCAGTTGACCCaATATTGGCTTGGTTCCAACAACTGCCTAGTTCTTGGAAAGCCTTCCTGTTTAGTTTACCTGGAATaATTTTACTTATTTTGCTTTGCTGTTGTGGAATATATTGCAGTTGTACTCTTTGTGTAGGAATGCAAGACAAGCTTACTCAATGCTTTCTTAAATTGGATACTTATTAATCTTCCAGATTTCACCTTTTGTCGGGACTaGAGTTATGAAaGACCCTCACCATACCAATGCTTTCTGACTGAGCTCCTCCaTACCCTGAATACAAGAGACCCTAATAATTAGGCAGGAATATCATCGCCCCTGTTCAGCCTAAAGAAGTTACAGAaGAttGATCTTtaTtaTCTGCAACCCTTAGGATTAAGGGTCCTCTTGTAAAaGGAGGGGGGAAATATGTCAGAGGTATTCAAACaAGAGCAACTCCATCTTGAGTGAGGGTTAGGAAAAATaAGGCTGAGACtTGCTGGGCTGCATTCCCAGAAAGTTAGGCATTCCTAGCCTCTAGATGTTTATGGTTAAGGGAACAGATTtATAACaTaTACTaAACAGACCCAGATTaGGAGTaTCCTGATATCCCAATATCTaGAGAACAAAAGCATTCCTAATTTaAGAATAaGGTAaGtaaGAAAGaTAATCCTTtgTACAAACTCTTGTAGCAGAtCATATCTCCCCAAGAaCTATTTTTaTtTATAATAaAAAGATTaTATAGGGTGaAtaGTttTTTTATTTAGGAAaTTATgTGTTATGGAGTAGTGTtTTTAATTTATtTTTAAtAAATTaTTTtaTTttA

>prima41_10720_100035_con_gagputein

mgntawipedsplgcilehwdkfkpnglkkrklvvsyntvwpqyytwknkkngqlpelwplilyfhlicfvsvrangikpirssifiaqskznsaigmcmfnerkgrkrirhtrzsldarppmSvlpPsSSpgsFvwnmsalmlqvywplllaslepvetPlSPPlYlSsPsLYllLPEdlSPaSTTcSGASYQlPKGNlClLRaVANGEEGTVRAyVpFslsilwLCkEsfgyFSEDPGKFvDEFqKLTLTYsLTwqDLyVLLSvCfTVEgKQcILGmAqalanEVLAqppplyiyiiYQAgGaAVPDqDPgWNYQRGSeDlGkRDpmViclLEglKkcMKnPVNYEKaKefiqgKDEnPglclVeaIRqYvtlilasqggQTLlGVHfFITlSAPDinRKLQKAAlGPqTPMnQlLgMAllVFNNkDKaeEAERAkwTfHKVqLLaagLgSPPaWvalpgiwltqgeakggKalsgalsslALGiNQCAHsentshWksdcpvfqgiPpVlegailt

>prima41_10720_100035_con_proputein

wdlrpsatalivtLavageninvwldvadkNINFlLDtGaAYSVLTHYsGllSPQNCMVmGIDgqAHtCHFTYPLSCSpgTLVfSHaFLiMPECpllemplvgnapcwetPLLGRDLlaQLQTVVSlgnhkadeglflll

>prima41_10720_100035_con_polputein

GnHkAdeGLlLLLfCDKgGKSIGtlSLPiEVTSQVNPIVWDTEVPGKVLNVlLvCIQLKPDgLYSWKRQiPLKaEAtRGIpPLiAKFLQFGLLRPfESPCNTPILPVKKPNGDYRFVQDLZAVNgAVIPIHPIVPNPYMLLAQVlGDANWFTVLDLKDaFlFFFFFCIaVlPDSQFIFAFEWTDPDSHLVYQLTWTVLPQgFRgSPyLFGNaLARELKMLqLNRGTIIQYVDDLLVASPTKRgSDENaIKLLNFLGANGYRVSlHKAQvSTQEVKYLRYVLTPGTQAIaPEqKEAILGIPgPQTRKQLWAFLgMggFCsfwVPGFGHMAKPLYdALKGAnVilLEWvSNCKQAFNALKEKLGSAPALGIPNLDKPFFLYgaeKQGTaLGVLVQKLGDIPqPVAYFSKQLDHVALGWPgCLRAVAATALLVDEANKLALGQHLEVLTPHQVQGVLEAKgHQvMTGGHLLKYQALLLDTPDaTLKVCQTLNPATYLPEPTGTLDHSRIQVMpQVYSsWLyLKDEPLDNPEVEwFTDGSSFVHQGNRKAGYAVVSQHKVIiSQaLPASTSAQKAELvALInALQLGKDLRISIsTDSKYAFLVLHAHAAIWKEZgLLIAKGSPIKHHLEILNLLDAVLLPKEVAIIHCRGHlKGDSSVAKGNSFADAAAKATALKqPVGLVGMLVPSAlVMTEPRYTKEEqEWAKGyGLIQDPSGWLINDNKLLIPDANQWKIVKHLHDSTHLGRDSLFQLMvfLfIGKGLLKTVKQVTqACELCAwNNPsNQaLPSPLVRlVQHRGTYPSEDWQIDYTQMPPCKVFeYLLVFIDTFTSWIEAFPTWSEKAIfVaKlLlnEIIPRFGLPKSLQsNNGPSFTATITQNISSALGIQYCLHSaWaPSSEKVERANqTLKRTvAKLCQEaSEaWLtvLvMALLwVCvAlkGlivSPFkimvWKAFLNYRlPidiRYFQaQNYVINLGQVQKALLEYGNQRLPSPTKEENIVlTQPGnwVlLniggKgpPaiQLpPKgKGSYQVLLSTPTAVKlLGINS

>prima41_10720_100035_con_envputein

lgitsgstylelnlslmkshrptehkrlipvsqpvtsdccseeikgmgniKIWiGILLLGISwNHadsNLFaaWAQTLASLHNHanCWVCgeLPLpplpglpgiinwatlvlwglimigelniintaPlFPmiialcgLaPFPlIqRqegalfffvklgNstpalgsaLrlylggiTwVqgqvsgKalpglegtitvttqtgpmtwdglslnnviqpllltpnvvmgmqtnlpKmsalPfalGmvmglrtngWlaiLileLdgvvgVgLisqdtlSPnmtLtkLtgllsnwlavsaksghlsgaiwWlYlmavvlpsaavidlelaKlTaaaFsNTHHALTLLTgETSQvRQaALQNcMaLDILTaAQGgTCALIqTeCCvYVpDYsHtITQaiKaLdiHISATDvlwvtPilaWlqslpslvlgflfsLVYLElFYLfClAVVEYiaVVLFVzEiqiTLlpaFlnlilinlpdftllsdlgvmndlrpsigqCfLTELlrTLnaRDPNszagissplfslkklqdpatlrikgplvsggskicqrcsnqsnsilseg

**PABL**

>pabl_10750_100033_con_chaindnarm

tgtggaggaggtttattagtaatCAGaAgagtAGttTtCaaTaaTCtTtTaCtaTAAttttGAtgAatgtatAAgtaAAATAaTAGaaACtaggaaaatTaCattCttAGTaCaAGGaCtgAaATgTaAaAaCAAaaTCAAGataaCAgatGgagtTttTtaaACttaAaaattaaTagAaaGattAAGGCAttTtTCtatataagtTGTaAatGagATgAAGatAtaaaTAaaCACaTCTtGTACCAgaGtagaCttaAGattaaGaAatTagTggaGggCTtTaAAtaAaatTTttaggtCttAgaataTAGTTaAAgAtaAaATTaaaTaaAAtAgaCTTatGGtAaaTGCaaaattGCATGTAGaCAtATAACTtgAAtgTATATAAGCACtgAAaAAAaattTaaaACTtTgAaTTGaTCTGaTGaAaTTatCtCtaaCCTTCTCtCTGTatCtagTTACAGaAATAAAtTCtCTTaTTTCCAaGTataTCTGCaTCTCATTAaTTGGaCCataAGAaaAaaCAGCCAgAtCtaagTTttaTCCaGGaACAAAAatTttgtgaGgCAGCtaggaggCCaCtaGGAtaGtaGCTGCCtTtaataGCTaGaaGtTTGtaAtgaGACaGTCtTtAGGAaACTCCCAGCAGCTGCTaGGTAAGaTTGTCCTGaGGACtCTaCTGAGaaCTtTCCCTtGGaAaAaaACCACaCATCCttTTgaTaaCTGGGaaAGAACaGaaGtTaGGaaaaaGaAGatGTGattAAAaattGAtgAaAAgCAGagaaTaTATTaTTTCCTTATCtGGGCTtataAAGCCaTTtaTTttaGTACCaTTAaGaAGAtAATAGGtCTaaTTTGTACatCtatTTtgtaTTTGgTTTGaAatataTTTaCAttTaGgtgattttCTGTGagaATCTGaatatTtTtTGTGTGTCTaTtgaaatatttgTaTCTGTataTAagtTTtgaGaAtTtAAGATGGGaAAtaCTGtCTCtgTTCCtgCagataGCCCtCTaGGaAaaATtCTGttaGATTGGAAACAaTATGGtTAtCCaCCCATtACTAAAAAGAAAaTaATttatTAtTGTAAtAtaGtTTaGCCAAtaTATGTTTTaGaaTCTGaaGAAaGGTGGCCAaTTTtTtGaaCtTTaAaCTattatACtATCTtaaTtAGAACaaTTtTGtCAatGttaGGGAAaAtaGGgaGAAaaGCCaTATGTaCaaGCaTttATGTTACTGtATaATtAaGAtGTtAAgaaagaAGGaAATAAGtTAATGGTaCAatACaCaGatAAGGTTTGTCttaAgTCCtAaGGaGAGGaAGAAAaGGAaTtAGAaACtCAACAatTaaTAAATGtaCTTAACCCagtaggtgggaCaaaaGtAGCtAaTGCtCCagtaCTaagtCAaaaaaaGGAAGaGttattaCCCCtaGAATAtaAGGAaGCtGCAaAaaTAGTgTCTCCCTCtCaaAaTAGaCAAGGtACTAATTTtGatgGGaaGaCACTGAgCCTGGAGCAGGGCAATTTtCaCTataACAATAtCCaGTaGGaGTTaATtAGCAAGGAGCTCtGGCaGGATATTATTGGGCaTAtAaTCCTTTTtCtAtaTCtaAtTTatTaAATTGGAAAAAttaATtCTTCaTAtAGagaGGATCCCCAGAAAATGACtGAATTaTaTACTaCTATaTTTGCTACtCATtGtattACATGGGCaGaTGTGtAAGCCCTCCTaAAtATtATGCTCACtGCAGATGAGaGaaaGCTAGTtTTAGAaaatAGtaaaAGAGGAaGtatAataCCTTtATGATGAaaaCCtaaATGATAgtCCAGAtttTaAaaaGGCAAttCtCTgaAttGACCtAAaTTGGtAtCCTAATGAGGCaaaTGgaGCTGGgATGaaTCAttTGGAACACTAtAGaAaaTaCATatTAAAaGGCATtaaATCAaGatgatattggaaTGCCaAaACCTaAaAGttTaAaCAAaGTAtAGgaaCTttAatAaAaACCTAaTGAGGAaCCCTCaGAaTTtATGGAACAtATTGTCAGataTatAGAAAaTATACAGAgttagaCCtACAaGAtCCTGAAAAtaTtAaaATGGTtAATATGACtTTtAtAGGGCAAAGTaCCCCaGAtATtAGGAAaAAatTaCAAAAaaTaGAaGgGGCttTTGGaAtGAAtGCtTCCCAATTaATtaAtATTGCATTaAAGGTaTATAAtAGCAGaGAaaCCAaGGAAattAAaaaaCTttaaCAGGCAaCaaTACTtCTaGCaaCaGCAGGAGGAAAtCCaAaAaaAAAGaGAttCCaAAaataGAaAGGaAAAATAGAAAAGGAtCAaTatGCTTaCTGttaGGAAAtAGGaCAtTGGaAgAgAGatTGtCCaAAaTTAAgtCAGAatGAACCaAaaTCACTtATGGgaGTtAAGCCtaaAAATGAATttGAGGAAGATTGAaGGTGCCCAAGaCtCCCAaCAGCTCCAACtCTAaCTGAtATtAAAATtTCCCCAaAGGAGCCTtGGtTAAAaTTGAtAgtaaagaaaaaaaagtggatttttagtaatTGGtaCTagTTATTCAGTaGTTAAtACaCCAaTAACTGaaCTTTCTaATACTTtCTGTtAaTaTaGTtaGgaTAAGtaGagAAtTaAaATCaGAAtaGTTCCTGtaaCCCCTtTCaTGTAAAGTGGGCAAtaAaTTaATAatTCataAATTCgTTTATGTGCCAGAgTtCCtaaTaCCTTTACTTaGCAGAGAttTaCTATGtAagTtagagattAtaTaAtttTgtAatCaaGAGAAACatCAGaTGTGTCTtCAAaTGCCTCtaGAaCATGGatTtCAGCTGCAaGCaCTgCTaataaaCaCaGAGGCttCaaACtCTaAaatAGaGaCaATTCCaCAaGAAaTCtTTGACAAGGTaAagtCAGAGaTtTGGGCaTgAgAtgaaCCtaaGAaGGaaATtAaTGTGaatCCAaTaAAAAatAAAaTtAAGGAAGGagCCtAaCCaaTCCaGAAaAAAgCAATACaCtTTAAAaAaaGAAGtatTaGAAGttATtCAaCCAGtaTTaaTCtaGTTtTTGtAaTATGGCtTAATAatACCttaTCAtTCttCaTACaAtACTCCaaTttTGCtatTaAAaAAGCCTCAtTCaCAtAAaTaTaaaTTTGTaCAAGATtaaAGaGCaATTAATGAtAtTGTaGAAGAtaTaCAtCCCAttGTaGCTAACCCATatACTaTGTTTatTTCaCTaCCTaaaGATCatGAATGGTTTACAGTatTaGAtTTAAAGGATGCtTTCTTTTGCATACCaGTAgaaATAGAAAGtCAAtTattGTtTGttTTtGAaTGGACAGAtCCTGAaaCaGCaaCaCAGTTTCAaTaTTatTGGAtTGTGtTCCCaCAaGGGTTTAAAAACTCtCCAAgTATATTtGGAGAaGttTTGatTCAAGACTtaAGAaGtTTACAATTaGAAAATGGGGTGtTatTagAATAtaTGGATGATTTAtTAATaTCtAGCaCCTgaaAAtaaGaATGTCAAaAaAAtACtaTaAAaACtCtAAAtCACtTaGCAaCttaTGaGTAtAAaGTTTCAAGTAAaAAGGCtCAAATATGtAaaaAAACTaTaGaaTACTTAGGGTTTtTgTTaCAaaAGggAaCtAGAGCCttGAtGGTGGAAAgattAAaTGCAATTGCCTCCATCatgAtaCCCaCaACtAGaAaaCAaCTaAaaGGaTTtCTaGGaATGGCAGGaTTTTGTaaaATtTGGAtTCCtAAtTATGGatTaatAGtAAAGCaaCTATATgagtgtTaAAAGGaGCaGACaATGatttaatgGGAAaTgaaaCaAACACCAAaataCATTtaAaCAACTaAAgtaTAAaTTAATaTCTGCaCtaGttttaGGaCTtCCAAAtCCaCACAAaCTtTTtCAACttTAtAatCATGAaAGACTaGGTCTaGCACTCaGGGTCtTaaCaCAAAAatTaGGAGAAATAtTACAGCCaGTaGCtTAtTTTTCaAAGCAGCTggatACTGTGGCtAaaGGCTGaCCCCCTTGTtTaAGGGCAGTatCtaCgACCaGCtTGtTGtTaAAGGAAGCTGAGAAGCTaACTtTaGGaCAGCCtatCAtaaTtTaTGTGCCataCtAAGTgtTGGtGttatTaGAACAAAAGGtaGaCTATTGGCTaACAGCaGgCAaaTTaGGCaAATatCAGGCtATttTgTaaATGACCCtaCAGTGAAAtTaCAaACCACtaGAaCCtTaAACCCaaCtACTTTaCTtCtaCCTACtaaaGAAtCAaAagAACtaATaCATaAtTGtttAtAAGTTATTGATCAAGTaTTTTCtAGtCatCtaGATTTGAAGaAtACAGCCatgttataTGtAaAtTGGACATTtTTCatAaAtaGGAaaAGttTaGTaAttAATGGAAGAAaGAAtGCTGtaTATGCTaTaGTGAgtttTaAGAGGtAatAGAGGCAAGaACTtTaCaataGGaACtTCTGCACAGAAaGCagAGtaAATCaCCCTTAtaAGAGCCTtGCAatTGTCCCAAaGTAAaAataCCAACATCTAtaCTGATTCtaattAAATATGCaTTCATaATAGTCCATGCaCAtGGtGatATtTGGAAaGAaAGGGGatTACTGAAGGCTGaCAATACTGAAATtAAATAtGCaAaaCAAGTatTaGAatTACtAGAaGCAATAAAGGCtCtAaaaGaaaTaGCTGTaATGCaTTGtCtaGGCCATCAataCAaCAATTttaAAaTAGCaAaGGGAAaTGCtTTtttAGAttaCACaGCCaGGCACtTAGCCAGCtaataTTGAAtTCCaGataCCTtTAATTCCtCAAATAGATTTaaCAGgCTTtAaaCCtaaaTAtAGTCtTtaGaAtGAaAAaagTaCaaAaaaCAAaGGatTTatTtaaaATaAaaAAtGaCTtGAAAtTaAATaACAAaGGCCTAATtTGGaTgtagagataaaatttttttattattttttgtttaatagtattgttttgatatgtaattttaaatgggaaggatatgagaagtagatgatttgttaggttgtggtgttattttagaagtggtgggttatatgaagggaaagattggagaatttaaagttttggatttaaaaatggACCGGCTCAaaatgatgggagtgataaggaaAttTGTCagaatggtgaaatttaatgaaggaatgggttttttCAtCCAATatTAAAaTATATtCATGATaGCACAaatTaTGGGtaAGAttgtTCATTAaCtTTCATatAGaaGTATtagagAgGaAAAGGaaTaAaaGCtaATTTaGAAAaTAtAaTCCaatGaTGttAttTtTGtaCTAaAAATGAaCttAACAAtCAtAaTagAGGACAaCCtaGACAtCAAaagAGAGGaAAAtAaCCACTaGAaAATTGGCAAATaGACTTTaCTCAAATGCtACCttCCCgaGGAGGaTACAAATAtCTCtTAGTTCTaGTaGACACCTTCTCaGGCTGGGTAGAGGCatACaatgtgttGAaTGaGCAAttGAaGTAaTTAAaGTatTgtTAAAGGAAATCATaCCTCaaTATGGGtTTCttGACATaATtCAaAGtGAtAATGGaCCtTCATTCACATatGAaATaaCtCAAtAAGTAAaaAAaGtaCTaGGaATAAAATGGAAaCTACAtaCaGtgTGGAGaCCTCAaTCtTCCaGACAaAttGAAAGAATaAATCaaACatTaAAAACAAtCaTtGCCAAaCTaTGttaGGAAACaCaGtTAAAGtGaaTTCAGtTaCTtGGgATTgCaCTGCTCCaGGTAAGaaTAaCtCCCAgAAGTGGGATtAAaTgaaGTCtCTataAAATtATaTTtaGGAGaCCtTTTGtaGCtAAtttaTCtCaGGTtaCTaaaatatCtCtaaACAaGGAatTAaCTATTAAAAatTaTGttACTCACTTGGaACAAACTCTTAAtCttTTGCATAAATTTttttAACAGaAaCaTtaTaaACTCtGaaGAAgtgtaCCACaCaTTCCAGtCtGGaGATCAAGTGCTataaAAAGAATGGAAaGAAaCaGatCCTGCttaaCAaCTaCAaGAGAAATAgAAAGaGCCCTATGATGtGCTGtTaAgtACCaatTCAGCACTGAAACTaGtaGaCATCAAaCCaTGGaTtCAttAtAtatGatTaAAaAAaTTCCaGtaagaaaAaAaaTCCACgACAGAaaaaaCtaaaGCCACtgAATGGGAGataGAaCCCCTaGAaGaCCTAAaaTTTCTaTTCAaaAAAtGAtAAGAaTTTCttTTATaaTATTtTTtCTtTtTataCCTaaTaTTGTTaatGCatatACttCTAACgTtTTtCTACAATGGGCACAgaatTATGCAGAtaGCtTaCAaCAaGgagAttCtTGtTGGGTCTGTGGtttGTTACCCgTtaCTAaCACCAtaGagtTACttTGGTGGGTCTCaCCtaTaCAAGGGAAAtAtTaGatataTTTaCAAatCTTtataggGGATtTaAAACAaTGGACaGGGtCACAaATGAttGGaGTaACTAGaaaAAAtaTtTCAGAATGGCCtATAAAtAaaACTTTAAATGAgCCtttaaAtGaCATGAaAAaCCATTCTtaaTaAAtaaaACAAGaGAtaAAGTaATAGCatTaGCtAttCCCTTGTTaGAaaCaAAaGTGtttaTCCAaACTTttAaACCtCAaAAtatTtaATAtaaaAaTGGgTTTCTCCAaATaTGGGatGGGtTCATaTGGtTaACaaCCaCTAatGGaCACTTaAGtCAaaTAGCtCCCTTaTGtTGGGAaCAAaGAAAtCACTCCCTTGAtaACTGaCCaAAtaCaACatGtGTTATGGGATGGATTCtaCCaGaaCaGTGCCaACATAtTATAgtatTaCAaCAaAGGGAtgTATTTGCCACAGAtTGGTCTCAaCaaCCtGGCtTaAAgattgggagaatggttaGTATGCTCCCAACaGaACtCAaTGGCTtTGtaGCaCaAAtTTaTGGCttTGGCTtCCtttaGGtTGGtTAGGAtGCTGgACTCTAGGtatTCCCataGCACAAGGatGCTGGGTAAAAAaaAaaaAAaaaaaaaaaaTCtAtCCTtaTttTCCACATaTGaTTAAtaaatGGACTAGGtCaaTttTTCAtaaGaATGATCAtCTAgCtaCAATaTTtATGCCCTCAGTAGGttTAGGAACTGTaATAtaGCACATAGAaGCtCTAGCtAAtTTTACCaAAaGGGCtCTaAATGACAGCCTCCAaAGtATtTCTCTaATaAATaCTGAAaTGTaTtAtATGCagaAGGatATttTAtAAAACCGAATGGCCtTaGAtATttTaACTGCaGCTgAAGGaGGAACtTGTGCCCTCATCAAAACTGAATGtTGTGTGTATATTCCaaATAACTCTaGaAACATtTCCtTGGCaTTAaAaGATatatatCaGCAAATtCAaGtCATCTCCAGCttaGaaCTGTCACTaaATGAtTtgaTaGCATCataGTTTaGTGGAAGaCCtTCtTGGTGGCAGAAAATtCTgATttTCtTaGCCattTCttaaGCaTAGGaaTaaCatTaTGtTGTGGAaTGTATTtTTatgGtaTGtTtTttCAaaACATTCCttaaACTCATtCaaTTATaTTTCAACAGGaaCTaCCCtTaAGaCtCCgAAaTgAaaAatAtTaCaaaaaaCAaataGACaTCTTCCACTCtAACgCCaagttaagaaCCCTGAtaACGACCCtatTCAGCAGGAAGTAGCCAGAaatAttACaaaatCCAtTaCtCtataatCTgCTaTGttAtAgtttaAgtaTtatAgaAATCaTtaAtaAATTaAtatTaaaaAaagTaaCaGaCaaaaTatgaaTgAtaaaCAaAtaGaaaaGaCTGTataGaCaAtgaCaCaGatAGgCaagCatAaCAgTtaatTTaCACaaACtaagTaCtatAaatCTGattaAtataTaaAttAAAtAtTaaaAgtTaGaGAAAttaGTGCCtaaAtAtaAaaGCTaaAAattaAAgatAaagtCAtTAaGAgCCttaCaTGGGtTTtttCatAaCtTAaAgCaaaatAAAATAAaGAaGaCAtTtTTaCAtataCTaGTaCaAGGaCCCAttTaaGaTtaAgtaatttttaAaaTtttAaAtaAattTCaAGaCatgAGAggCaagTtaaaGAaTAaATTgAtTGaAaCaCCttaAaGGCAGaTgtaCAgaaCAaGtaGtaAtatAGaTaaaATatAaaTaAtgaCTGaaGaAaaACatGTAattttGAgaGGtCTggTGAAgtTaCttGaCCtTtTtgTaatCaGatatAGaAataAatttTgTtttTtAtCatttaGTCgAaataaaATTAaTTAgaCaATaaGattAaGCAaaCaGatatatTttaTgtAGGtAa

>pabl_10750_100033_con_gagputein

igvsVpvaalnlrlGmlLaspwepsGypPvgletvivpsniawpmyvLlzyglanicfgvwGmvanfliFkllsyLsElFCQclGkwgEmPYVlAfMLLynqdVKkeGNKLMVntllkvvLtpqGegeKgLetptVlnvLtPvggdpegAtAPvLaQggEgllPlEyeEAAelVSPSrtdqgliLggesLsLEQGNfpmtiilwgfiakslgwillGliSFfmiqLvnlensnpSYRvDPQKMTELfTaIFATHclTWAgVqALLNIMLTADEwqLVLdlalEEaqcLyDEslnDsPDldeAilsmDlsWyPNEAggAGMvHLEHYRkcILKGigSgmisgvPkPeSLsnVqalnqtlienPlsfmnilsgvykiYtpLdpQDPENvnMVNMTFiGQSaPDIRKKlQKvEgAvGmnapnlitlalkviiagepRevknlsqalillalaggtpKgKgfpkwkGKIEKDQcAyClgigtlgqgCPKLsQsEPkSLMgVKPgNEfEED

>pabl_10750_100033_con_proputein

rcprpptaptlsdiqifPqgagVKvtvkkqkvdflvhiGapYSVVNTPvTgLSnTfVsvVgvSgqLkSEwFLcPLSCKVGNnLIlhqFvYVPdflvPLLgRDLLCtlyvslif

>pabl_10750_100033_con_polputein

tpentqlvlQvPlEHGlQLQALLvssEApnpevgaIPQEvlDKVspEvWAsdgPekaIsVsPvKiKlKEGaqPvqKKaipLKegaLggIQPalvwFLqYGLIvPcqSpYnTPiLlvKKPHSHKfrFVQDlRAINDiVEDvHPiVANPcTvFaSLPgDrEWFTVLDLKDAFFCIPVeIESQLlfaFEWTDPEaAaQFQccWiVlPqGFKNSPsIFGEaLaQDlRgLQLENGVLLqYvDDLLISSpsewgCQnNTvKTlNHLAacgYKVSSKKAQICkqTvgYLGFLLQegtRAlmVEtlsAIASIvmPsTRkQLkGFLGMAGFCcIWiPNYGlpaaalsellkgadndpfpWEveqtiTfiQtlecKLISAlalGLPNPHKLFQlYmHERLGLALwVLsQKLGEILQPVAYFSKQLvTVAkGwPPpCLRAVaaTsLLLKEAEKLTLGQPvmvsVPlsvgVvletKGgYWLTAgslgayaailLnDPaVKLQTTgaLNPaTLLlPTgEpeqliHnCleVIDQVFSSplDLKnTAllcanWTlFangsSLViNGRkNAaYAvVsSzEgaEARTLptGTSAQKAqsIaLtraLQLSQgKsaNIYaDSnsKYAFiIVHAqGaIWKERGLLKAvNTEIKYAtQVLELlEAIKAlqvvAVMrClGHQcsNsqvakGNAFaDcTAwHLASsnvElqvPLIPQIDLagfnlgtvLntKrlaesKGvalmKnglKvNnKGLIWvpeikfllsysfclhlvlflhplltsfynghrtmqtassmipvrsvacypflappwwvspmqgkdwrhlqvfldlkhwtgsqmmgvtkanvsewlhnfqdpghgslvHPiLKYIHDgTtfGzecmlaflswysigKGlkAnLEsivlcCylCakNElNNHstGQPgqQgRGKyPLENWQIDFpQMlPapGGYKYLLVLVDTFSGWVEAypccsemAmEVvKVlLKEIIPqYGllDIIQSDNGPSFTsdIaQqVnKaLGIKWKLHsaWRPQSSgQmERiNlTLKTilAKLCwETqLKglQvLGIaLLqVRvaPkvGlszslceIIFgRPFaANlSqVaeaplnkELaIKtcaTHLgQTLNlLHKFssnRsvvnSvEacHpFQpGDQVLlKEWKEagPAstlqEiykgpydvllstssALklvdikpwihytrlkkflqeets

>pabl_10750_100033_con_envputein

mmaakhqfstetgghqamdplhtiekvparrnihdrghqghrmgdgtpgrpkisvqqmtrfsliifflsapfvvnastsnvflqwahsyadglqqgdpcwvcgsvgvTyamEllwwvspLpGkdwvffqsfigdlKtlTGaQMmGVTRaNvSEWPINnflnpfgzHdKPFlvNiTRDeVIAlAiPLLdpKVfvQTfkpkcliPqwlspnlGfaqivngliWalastgplvLgtiaPLpwpltncslgygmnspRvvgwippgpakgtIllqqrdLvaTpglqigglgwvapngtsmalwpnlvavaPsGlagtltlgiPiAQGcWVKtmevypilPvvHvvNqgTRavvHryDHLaaIFMPSVGLGTVIwHIEALANFTqRALNDSLQSISLiNaEvcyMreaILqNRMALDILTAAqGGTCALIKTECCVYIPnNSgNISLALeDvcqQIQaISSlaLSlhdflASwFgGRPSWWQKILivLAflwsvgiallwgvllcclFsQnIPwTHaviFQQvLPLslpsqeyfqsQvDlFHSNpkfkaPZqRPlSAGSS

**MER65**

>mer65_10850_1024_bre_chaindnarm

tgagataacttgctgcaactctaggactaatgctgcccactgatatcacccactattgaagcttgccagctccccaaagctttactaatgccaatgaattttttctcaagaccacacataacattcttcttttttataaaacccccaactttctctttgttcttaggatatactaaagaccatctaatctacatacatgccctgaattacaattcttgcttcccaaataaaacgttaagtttagagattcaactctacattttaattttgactttgaaatacatggtgtcagaagtgggctctaaagctgattgaccttgaggagaatcactggcccttgggattatggtgtgagttacctataatggagatctttgagtgccccttttccccctgatgagtctttcttggacagagctcctgaatttggtttgagttccattttatttgggatttggttgagagaggtctttctcctccctgtttgaagagacgtctttctctctcttgtctgggatatcttttgttgaggagggttatttcctcgtgttgacctcagctgtgaggtttcgacctccaggttcagatgaggggattttgacttctctaatttagtttgtctcaggaataaactagaaagcaaaggctctaaaactaagcaatctgaatgaaatgtgtaccttaattggtacactgaggcatctaaatgcaattgggaatcaaaatttttcttgctccaaaatattaattaacaaagacaaataataaattaaaagaagaaaaaaagactttaaaagttcaaaatccttctgtctctcaggtgccatctactgcctctcctctcaatctttcactttccaaactttcttcctttttaatacatcttcctgtgctaacaccccatctcctgcttttcagtctcaacctagaaaacttgctcctttaagatcaggctctctaacaatcctgaggttccctagatttcttatgacccttggactaaatttgaacctcatgctatagccaaggaatttcctagttactgaagactcccattatttttccatgaattttacattacagttcaggcttaaaagagcttggcttctctggcctttactgacttctttgtatgcttgtagaaagccaggtccatcaccaaatgcaaaagcagattgtaaacatctccaaaaggatttagtgagacctgttcctacttcctcagcatatggtctgcaatactgggaacagactcagcaacttgcaagaaatttacatggggccattgtcaaagcatttcccaaagccacagactgcaataaaattcaagcttgtactcaatagccaaaagaataggttcacaactattatatcaggttccaagctatacttagagaaaatcctagttttcccatggatgttgactccactagagtcacctttaattctgtgtttgctaatggtctctcataagagcttcctcaactaataaaaagagcctgcctaaaataggagaccatatcaactactaatcttgttaatttggcaaattaatggcctgaaaccttagacaataccaataaaaagaaagccactaaaattttaaatgtccaactacacagatgaatgcttccagaaaaaacaattgagcaaacaaacaaaaaacagacgaacaaaaactccataattgagaccctaaatggtacaacttgaatccacaagggctatttcctggtaactgtcactattgtaaatagccaaaccactggaataagaactatcctaagcttaaatgaattagacatccttcaaaagaacttccattatctcccccacaatgtaggggatgtgaggactcagtgttttcttcaatcctccctcttaatcactgtgaggagattactcttcagattggcaataagattctaactgttctcagagacacaggaacaaccctatcagttcaaccccatctgcccctacctcagagtaataaaacatccaaagggtggcagtctctaataagccattgatagcatttctgtcccaatctattactttctgcttgggatttttacaggatactcatcttttcttgctggtggagtctgctccagctcatttactgggataagattttttaaagagaaaaaccatgccggcatctccttctcccaaaagagggaaataattctagaatttgatgaccccaatagttcactgaactgtcaaaaaaaggaaaacctaaaaaaaaatatgtttttaaagtgactcaagattgcttcagtttggacatatgagtaataaaaactaggcctataaactaaagttaatctctcactcacaaggatgaggtctgtcaaatatttaactgacatgtactaattacaacctttcctatcagataaagcacaaagacaggataaaatcaggtatctctctccagaccttaagacaactatatactagttaacattttactctgtatatgttaaccacctatttgctttgtcttatatataaggtcactgagcactaatcagaattgtaagaatgtcacctttgcttcactgtctcccttctttcattttttagataacaaaatgtataaattttgagctcatgtagcccactctaggacatattcttggtttgtgctaattctgcatttctggattgcaatcctcaaatttggctcaaaataaaattaagtttatattgactcttattttagcctctagttgacaggactctaccaatgatgtctcagttatttgttatgctgtctcatgtgccaaagacatctctgttgccttccaaaattccttattggaacagacacctgtgtctttatgggccatatcatcagcagagtacactcctaataaaaatccaaattgacccccttgcctaatatcgaagagtatctcttcagtcaatttgcctgacaacgcataaaacccacaactgaaaaatagaagcctcgaggtttaataattctttgtactagtctctgtaatactcccattctcccaataagaaaacctaacagccaaggatgaagatgtgttcaagataaataatatcatcataccccatcatcccatagttccaaattttcacactttgctgacatgaataccaactgatagtaaatttttcactgtaattaaactctgcagttcctcctttagcttcttgttgattaaggccatcaatacgtatctgccttcacctgggtaaaactgtaatatacctgggtggttatgccttagggatatactgaaaatcctttttacgtctcccaaattctgaatgctgatttaggagatgtggctttttcttaaaacttgtctttattacattagtttgttcttcctcataggcaagactgcaaggaagacagtgtacatttattaaaactcctagccaccaaaggtcatatggtttctaaagaaaaactacaactagttaagtcccaagttaaatatctgggtcatctgatttcagaagcagagctgcatttggatcctgaacaactacaaggtgttctacaattctgtaagctcaaaataaaatgacaactttgagggctttttggattcactggatgggatactgccattaactggataccaaacttctctttgactgtctgatccctgcatgtcctacttaaaaacctctaaacctgataccattatatggaaaaaaatatgtagttactggttttttaaacttaaatgaaggacttttaaatactcttgccctagacaccctaattatcagcttcttttcctctttttgtttatgaatagaaaggaaatgccttggacatcctaacccagaaacatagatttcagcaccaccccattggagattacagccagctactagagccaatagtagcttgagaattaccctcttgtttgtaagcaattccagctaccaccctattaattaacttccactgaagaaattatcatggaatctcccttaagcatttttgttccccacactgaggaatctcttctaaattcacatcacacctagcatttctctaccagtcaccttccctcttatgaactccttttacaagcttctcctcacaccactctttttcaatgtaataatctcaacccggctacccttttgcccccttcttttggtgagaacccccaacccctaccacacgactattggaaactctctgactcctcagaaagatttagaagaaactccactagataattctgaactattatggtttacagatgaacgatatttaaaaaagaacagtggtaaatactgctcaggttatgtagttactacttcctttgaagtcattgacgatgcccccttacctatggctacttctgcccaacagaccaaactttttgctcttgctcagctttgcctccttgctaaaagaaaatgtgcatatgcatgtgtgtgtatgtgtatataaatgtgtgtatatatatatacacacacacacacacatatatattttacacatacacatttaattgagtcatctacttcctttcacttctgattggaagcacagtgagatattgtttctgaatgtcttggtacttagctaacccacagagaaagcagaatagtgcctgatgctttttttctgggaatgttaaataaaataatgaaggttcatcattatatctcccagatccacattgttcttgttgactctcctgagagtgttatcgagagaagggatttttggtttattgtacaaggctgttaatatgctgccccatatatacgtatatatgtgtatatatacgtatatatatgtgtgtgtgtgtgtgtgtgtgtgtgtgtatatatatatatatacagacagcagataggcctttggtgttgctcaggattgtggaatgctctggaaacaaaggggtttcctcacttccacaagagatcaaattaaaaatggaggattctatgtacaggacttcctgaataccatctagctaccagctgcctgggttgttaccagaactccagacattccagggtagactctatggaagctagaagaaacaaccttgctaataatgctgcaaagaatgatgctattaaaggttcgcttaatcaaatctctactgtggccccactaaaagagccctcaaaagatgactgaaaaataattaccaaagactcacaaaatgggcaccagaagctgaaaaggaaagatcatagttgctggtttgataacaaaaaagaactatggtttgaacttgatagcaaattagttttcccaaatttagtaaaatattcattgttaaccaaagaacataatttgactcactgggcaattaacaaattaatttcttttaaaaaacaatattggtggggtgacatttaaaaagctgcaaaacatgcctatttggcttgtaccacctgccctaagtttaatctagaaaaacccatatgtaccaatcctggacattttagttgacctaatgggcctttcaaggtttggcaaatggatttcacccaactacctcttcccatgggtatatatttatatgcgctagtaatggcttgcatgttctcccattgggaagaggctttttcctgtagataacccagtgcttcaggtatggccaaaattcctccagaacaaattatcccacccagggaactcctcttgaaataaaaagtgattgagaatcccattttcctggccatgtaataaaacaagtatgttcagtttggacaacttcatagtatttccattgtgcctactatcctcagtgttctggcctagtaggacgaacaaatggaactctacaaacccagttggaaagatttatggaaactctacacctcacttggcctaaagttctttctttggtattattaaatctaaaggccacttttttggggggctcataaactttcacctttttcaactgtcacaggccatcttatgcacttggctccttctgcttttgacactcaatgaaaggagatatacttcaatattgtaaaggcattactaaagtaatgaatggcaatcatactttggtagaacatctttccacagcacagtctcaggaaacaaagacctaaaacatcatgatctgcagcctggcaacttggtttactggaaaagacacctacataagaattctctccagcctcaccgaaaggcccataaccagtccttctcaccaatccctgtgctgccaaactaaagggcattgactcctggatccacatctctcattttaaaagatttctacttctcactgaaccagcacccaaacagggaatttaaaactaaattcccttcatcgatgagataaagcagataatatctgtagtggactgtggactaggtcagtataccatttaaaaaaaaatttcagtctcttcttacatgcctacattttcctggaaagataatgccctagtgcacattttctagtctaatgccaaaggtggaaacataactgttggatctgtcactcaaaactctgatctgttcatgatgcaagcgatctttcagtttgccacataacagatttcactcatgttactaatgtaactgtttgttcaagttgtgcatctggccctttttataaagtcaaactttcagactcacatgttcagattccctgtttcaccctaacagagctgacaatgtgtagaaaatactttgaaaatagatttaaaaattataaaaatcagaacttaaaaacttgtaaaaatatttatgatacaaattctttaattgttgatggtttccaacaatgtaataacatgatagtaagaccttgaataaatattgctgacacttccctatgaataactattggtacataaggtactctacatgggattacctgttgtgccccaataggatacatttttatttatgggggatatggtaatcaatcatatgtataggcaacatcacacctcaataaatggaaaggaaaaggccaatgcggtttaggatgtttctggattcaaattaagttaacaaactttgaacactgttgacgcctattaccttatgctttaatattaacagagataccctcctagaaggcatacatccagccagatgggtctcagttgccagagcttttctgccttgtatcagtcacttagtaacaatggttggaaacctatgcttaacaattggagctgactctagagctaaggtcattgtgacccaacaaatttccccagattttcttgctaaagtagtattagctaacagaattggtctagattacttactagctaagctagtgtttgtgcaatagctaacttaatgaagtagaaacacaattacacaagataaaaaaaatggcttcaacaattttcaccagatacttctggactttttgatctatttagttggttactttcaaagttagataatggtttagaactattattcaaaccagatttattatactgttgctgattttttttgtattatgatttttaaagctttgtccttattgtctgtctaatctttgtaaagccaactcttctaacaggataatgttagctcaatatctgaagatgattgctgatgcctatgggactgataagatagagcttgataccagactccaaaaagacctttcctgaggacatatttcagtctggcttttttgttactcaacgtggcctgtgtctctgacatagatccctattctttttcactggtgtgggacagagaaaactgggacaggtccatctaggcatggagggacagttaagcttaattgcaaaataattgattagtgatatctgcaaagaaatatcttgaacaaaagggagaaatgggggaagagaaatgtgaaagtttgaccccaatcggaaggaacaggagaaatagcactcaggtacataacattgctctagaaatataattctcctcaagtgctatgcaagtctggctgctgaaactacctgttgtaaccttagaccagtttgatctaatagctgctgagataacttgctgcaactctagaactagttttgcccgctgccatcacccacccagcagatcttgccagctccccaaaactttaataatgcaaaagaaccttctcttagcaccacacataacattcttcttttgtataaactccccaaccttctctttgtacttcagacatactgaagacca

>mer65_10850_1024_bre_proputein

iTLQiGnKILTVlrdtgttlsvqphlpLpqsnkTSkgwQsLisidSISVpiyYfLlgIftGyssflaggvcsssftgirffkeknhagisfsqkreiilefddpnsSlncqkkENlkknmflKzlkIAsvwTyEzzkLgLztkvnLsLtRmrsvkyLtTctnynLSyqikhKdriksGislqTLrqlyts

>mer65_10850_1024_bre_polputein

nillciczpPicfvlyiRsLsTnQnCknvTfasLspffhfLdnkmYkfzaHvahSrTySwfVlIlhfWLQssNlaQnkikfiltliLAsszqdstNdvsviCyaVscAkdislPskipywnrhLcLygpyhqQstlLiKiqidPlAzIeeYlfSQfAzQrIKPtteKzkprGliilCtSlcNTPiLPirKPnsKDedVfkINniiiphHPiVPNfhtLlTziPtdskffTViKlCssSfSfLLikAintylPspGzNcnipgwLclGytenPfyvsqiLnaDLgDVafsznLsLLhzfVLPhRqDCKEdsvhLLklLatKGhmVSKeKlQLvKsQVKYLGhLisEaelhLdPeqlqgVLqfcklKikttLRaFwihwmGYChzLdtkllFdcLiPacPTzKPlnLiplyGkkyvvtgFlNlnegLLntlALdtLLsasFpLFVyEzKGnAldiLTQKhrfqhhPigdySqlLePivAzelPsCLzAipAttLLLtsteeiiMesPLsIfvPHteESLlnshhtzhfstshlPsyelLlqaSphttlfqcNnlnpAtllppllvRTpnpYhttignSLtpqkdLeetpLdNsellwfTdERylkknsgkycsgyvVttSFeviddAPlPmATLpNrPnfLLLlSfAsLlkenVhmHvcvcvykCvyIyihThThiyiLhihizLshllPfTsdwkhSdIvsEclGtzlthrESRiVpdAfflGmlnkimKvhhyisqihIvLvdsLRvLsrEGifgllyKavNmLpIYTyicvYiriymcvcVcvcvciyIYiQtadRplvLLrivEcsGnKgvsslpqEIkLkmedsmygLPeyhLAtsClGcyQnsRhSRvdsmEARrNnLAnnAAKndAikgSLnqisTVAPlKEPskDDzkiItkDsQnGHQKlKRKdhsCWfdnKkELwfelDsKlvFpnlvKysLLtKeHnLTHwAinKLisfkkqYwwGdIzkAaKhaylaCttCpkfNleKpIctnPGhfszpnGPfkvWqmDFTqlPlpMGiYlYaLVmacmFShWeEAFscrzpsAsgmAKippEqIiPqGtPleikSDzeshFpghVikqVcsvwttszyfHCAYyPQcSGlVgRtNgTlqTqLerfmeTLhlTwpKVLsLVLLnlkaTflGGszTFtffnchrPSyaLGSFcFzhSMkgdiLQyckgItkvEwQsyfGRTsfHstVsGnkdLkhHdlQPgNLvyWKrHLhKnslqpHrKAhNQSfSPiPvLQTKGh

>mer65_10850_1024_bre_envputein

IPFKKKFqSLLMPTFSWKDnALVHIFzSNaKGgNITVGSVTQNSDLfDASDLSVCHITDFTHvTNVTVCSSCaSGpFYKVKLSDsHVQIPCfTLTELTmCRKYFeNrfKNYKnQNLKTCKnIyDTNSlIVdGFQQcNNMiVRPzINiADTSLzITigTzgTLHGITcCaPiGyIfIYgGyGNQSyVzATShLNkwKGKgQCGlGcFwIqiKLTNFELLTPITLcfNiNRdTLLEGIHPARWVSVarAFLPciSHLVtmVGNLCLTIgAdSRaKViVTQQISPDFLAKvVLaNRiGLDYLLAKLvFvqzLTzzSRNTITQDKKNGFNNFhQiLLdFLIyLVGyFQSzimVzNyySNQIYYTVaDffCimiFKalSLLSv

**HEPSI1**

>hepsi1_10810_3172_bre_chaindnarm

tggacaaagccatccagacaccctccacccacctgtcagcccaggccacctccagattccagtttcacacagaacctgtcctgctgctgggcaggacacctcctggtcaccaggctgacctggcctctgtggactactcactgccttaggtgccttttgctccccaggatccaaaggagtagctgactttttccaaggagcactgcccatggggacggagccctgacaccaaagggacggctgccctctgtgcgcttccctcacactttcccagatatgagtcacagccacctgtgccaacgagcacccttagaacacccattcacccagcaggtgtgaccgctgcaaagctgaaccttccatctggccaccaggcccccagcatggagcctgggaccagcagcagcccctactccaaggctggaaccagctcagatgcaaccctctcaatccaaggagtctgggactccacattctgggactccagctaattttatgcataaaaattatgggcccagaacttgtgcatttttagaaaaatgggttaacctaactaaagaaaacttagaattaagatggctgcaatggggaagttttaatttggataaaattgtttatttatgaggcatattagaaaagggggaatgaaaaaccccataaaacagtgggatgtgtattcttttattggtatgcagaagcccctaaaagagtaaatgaatcaaaaattgactccttaaaagattctttgcaaaaagcaaacgaaaagcttaagcaacaaactaacgacatgatgaaagaggactgtactgtgactgacctcatcctgactgttccttctctttatccttctctacctacatactctgagtccactaacctttttgctaaattaccctttcataaaaaataaaaaatatgtatatattaccctttcaccctgaagacgattaaaaaaataataataagttagacaggcaccttacaaagtgagactttctgatcagtcatgcctgcctgctgtaactactttcactccatggtctaaaactgagcttagagccattgtgagggacttgcctgatccaagagaaaatcctcaaaaatttactgaggaatttagaatcctcataggagcttatgatccaggacttcctgaccttcactaatgtattcccgtgatattggggcctggtaaagctcggaaatggacggcagcagcagaatggggcaaacctgaggaggatgttaaagacccctccaaaagctcctcacaagaaggaccaaaagtcgctggaaaaattgctgaaaaccttttaaattcaattcctaaaatttttccgcaaaaaatggactggtccatcatacaatcttgtaaacataacaaaaaaaaagatttcagtttcagattacagaactcacttagaaatgctgtttgtgaaacattctgggatccaagtacaacaagaagtatttcctgcaaggactgaaacggcattaagtgctctatttgtaaatggactccatcccgaactcagcaatttaattaaaatacatgagttgggataggaagttacagatatgactaaattgttggccttagctggacattttgagaggactctagagcaagaaaaaactcaaaaggctaacaagcttatggcccttcagctataacagttacagggaccgggacccaagggattaatcattttatgtatccttctcattttaaatcacaatcaggaggtcctagaacaagaagttctttgccccaagatgtctgcctgtattgcaaacaaccagggcactggaaaagggattgtccgcttttctatcagtccaccaatgagcctccctttcggccaaactgtttcaccactagagggagcccaagagaccttaggcctcctgataataatcaaccttgatgaggttccaagggattctccagtagattgctctccgtgcctttaaatcaacgtggagaaacaggttaaaataaatggggagttgtgtacagtcctcggggatatgggggctactctatctaccataaaccccactttaataagccaacaaatcccttggagtaaaaaggtcatttctgtggtgggggtttcaaatcaagtttaagaggttcccgtatctgaaccaatccaattagctttgggccccttttcagaaaaacacttttttactatgttatactgctccagtaaacttgttagggcgagatttgctttcaaagctaaaagggcacataaaatttttctcaggagaaataaccttagagtttcctgattctcctggactagaattgttatgctgtctacagacagaaatctataagattgaaactcaggcctgtaatacccctgatctttcaaaaatatctaaatgtttttgggcctcttccccaactgatatggtaagaattaaaagtttggaacctataaaagtacaaagagatcgttctaaacctttgcctaaattaccccaatatcctctaaaacctgaagcaattcaagggctgtcaccaattgtagaggatttcattaaacaaggactcataatcccgtgcaccagcccttgtaacagtccagtactaccagttaaaaaaccaaatggacaaggctggagatttgtttaagatttacgggcatttaataaaattgtaataccaagattcctgtagttccaaatcctgatactttattatctaatgtaccaattagttccaagtggtccacagtaatagatctctgcttagccttctttagcattccagttcataaagtcaatacttgtttgcctttacttggaaaaataggcaatacacctgggctgtaaagccactagggtttaccaaagccacttcatatttttcccaggcattgcatcaggacttaataacactacaatttcctcaaaattgtactctcattcagtatgtagatgacttattgttctcccactaaggagtgctctgaaattgactcagtttaccttttacaacaacttgcatataaagatcacaaggcttaaatggaaaaacttcagttttcaaaagaaaaattccactatttgggacatgacttgactgccaaagggatttcctctcacctgggaggataaaaactgttcaaagttttccttgacctgaaaccaaaagacaattaagaggatttcttggacttacaggatattgcagatcctgggttccaaatttttccttaataggttcgccattgtatgaactcactaaaaatgctgtaccagagcctttaccttgggaagatagtcatgagcaggcttttagccaaatgaagttggccttacaacagcccccagctttaggacttccaaattaccctaagcctttcaccttgtttgttcatgagtgtgacaatcaggcattaggagtccttacacaagaacgtggtgctaaacataggtccattgcatacgatagcctgcaattaggcccagactctaaggcatattctaactgtttaaaagcagtagcagcagctgccaagctggtagaagcttcatcagatctggttttaggaagcgaacttaatttgcaaacctcacatgctgtggaaagtctgttaaattccaaccaaacccagcatttttcagtaagtaaactaacattttatgaattactgctatctcctaatcttcatttaaaatgctgtaatctacttaaccctgctactctgttacctttgcctgacgatggtgaagaacacaactgtgtaaatgtagtgccagaaatagtggcccctcatgttgatttacaagatactccagtggataatcctggatttatactttttgttgataggtcctatcctaaaatctcagaaggaaaataccaggcagaatatgctgttaccatccaaaatgagtgaatagagaaggaaacgcttcctcaatttaagtcagcccgaactgcagagctttttgtccttacccaagtttgtcatatagctgaggacaagtcagtaaatatttatacagctagtagatatgctttcggagtagtacatgattttggcgtgatatggaaactatgagggtttctcacctctagtgggacctccatcaaaaatagaccccaagtaaatgagcccctttctgctatcctgttaccattgtagactgttgttactaagattgaagctcatacttgtagaactgaacccaaatatcagggaaatgctttagcaaatttttatgctaaatcagctagtgctgaaactgttaagaaatgcaatctgaatgaactccattggattaagccgagcccacttccttatgatgacctgcttagtaaacagtgcagtgcacctgatttggaaaagcaaaattggtatctaaaaggatgtaaatttaatgtgatgcacagactcacggagggcccagacggccgcctggtccttcctgagtctttgaagcttccattgttgaaaacgctgcactccaccgctcatcatggaacagacaaaatgatccaaattatgaaaaaatactggtcgggtgactgttccaaaattgctaaaatggtttataatcaatgtttgacttgtcaaactcataatcctggaaaaacaatcaaaacttcaggtcatatatttccaccacctgatgaacaatttgagcatttacagatggacttaatttagttgtgacgcgcaatggggtatcagtatgttcttgtaatagtttgcatgtttttcaaaaaggggccccttcagagtcctggaactgtacacctgttggagactttaaagtaaagctgaccaaggaaatctctcttcagaagcagacagcatcctagttttggacagctttcccaagatcatggaccaagacttctctatcatcaggaaagccttatgtgtttttctgttttcctcatcttctgccctaatcttgtccttttccctacaggcaaacccatggcaccataatcagtggatggctttagctcaagtttatgctctagcacaaaaccagagtaattgttgggtttgtgggctaatgccaaaaaaaaatcaggaaatgattccactggtgccagtgcccctccgcgttcccagcgagagctacctgaaactaagggaagaacagaaagttattcttgatatgctaaacatcactgctacttgcttcctacactcactaaaaacaacactctaactttttcaattgataagtcgatcattaccaaatataaaaaaacaatccaatccaagtgatgccagcaaaagatacattgtgcttccagacatcacgcactcaagatttgggaattacttatgttgatacaagtgattgcttgtataatgtaactggattaaatccagtagggtctcttttttactagatatggttatatacccttacaacatgctataaaggggccacagaaaagtaaatttcccactggacgttgttcaggaggtatgcagatttgtggtcgaacaaacctgactgatctctgctcaaatgcaactaggtggccctcttttccaatccccaagggtctatactgggtctgtgaagagtctgcgtatcctgttctgcctcctcattggttgggttcttgttatgtggcctggcttgctcctgcctttcaaatagcttcccctgaaaattctcatgatggcccttatgattggaggccaaaatgatcaataactgaaattagcactagccttgaaatatttgaggataagctcgtttctactgaggaaagattccagtgggattcctgggggtgcactcttggtggtagtgagatagcagttgtatggaatttaaagctaatttgtaaattgggaaaaatcttggattttgtaatcaatcagacctcccagggtttcagatgggtagaagctgctctcagaaagatagatgacagtagatgacaacacgtgaattcagcaaaaacacttactggaacatcgtgcaacctcagatctttttgctcaagctggaagcctaggtttggtgttaaacaaaactgaatgttgtactacttatctcttacctgattttgttattacaggaagcttaatttaaaaggtgctgatactgctgtttccttagacactgccaccaaatacattaaagacatctctcaagagaaaggagcccatgacatgtttacaataggcaactaacggtttgcaggcatcctaagggtggatggcaagcttgggttttccaagggcttctaatccttataattcttctagtaggtttccaagttattatgacttgtattaccaatgtaacaatgaaaatgagtaactgtttaagtcaggccactttacagcaaaccatggtccttaatcgctatcatactctaaacgaggataatgaccaattggaccctaacactgttgaactgcctatattgtctgaaccttcacttggctaatttggttcggttcatttcataagaactctcattaaggagtacgccttggcattttgatagtatcctcttgatagtcataataatcgtcaccctgatacactgtatcctctcaagtcttaaatgttttgtatgcagccatccattgagagttgaatggtctcattccaaccaggtcaaaaagaacataaagaatcatttagctggtacaaagctgcaacttgtgaattccatgttaacaccaaagaagatgtgtgagactccatactgaaactaaagaagacttgtgaattccacactgagaccaaataagtctttaggatggtgacagagagtggcatcaatgcctaaagcttcggtcaatctctctaaattgagaggctgaccaaaagaggggaattgttaaagcaaactaaatatggcctaagaagggactccctacctctctatttgagtccttgtggatgaaccataagctggcttaataggcagacaagatggaaaacccaacttagtagtatgcacctataacaatggctgagtgttggccaatcccagcggccatacttcaaccactcatagactgctgagtgtttaaactgtgttcaactaaggcaaacgccgagatgcaaccaatctctgtttctgtacttcatttccaattcctgtacatcactttacttttttgtctataaatttgttctgaccacgaggaacccccagagtctctgaatctgctgtgattctggaggctgcccgatttgtgaatcatttcttttttatttttttccaattaaactccatcaaatttaacttgtctgaagttttaacagctggtaacaccagcaccatagcttcctgatgcattcactcattcagcaaatattctctggtcctgctgtgtgcccagctgtgtgcctcagtgagcgtgatccgatgaggtcactccccacgtggagctcacgcccactctgtgacgtgcagggacggtccatggggttagcagggatgatgagcaccgggaattggccattttattaaaagagagtcctgtatttgaaagaaataatggagaaaaggcctacttggcacttgatcttgaaaaatgaaataaaaatatgaaaaataaatgtagccttttttgtttgcttgaaatgtatatttaacaaaatgtcccccctcccccagaaaaggaagctataatagcaagtatacactgagttttagaaacaacaatgaataaataagtccacagtagattaggtacgtgtgacacaatttttacgacaaaattatagcgtatcagcagaaatcttctagggcctcattaattttcctaagggttcgtaaatcttctagtgcttaaattgttctctttgaaaccgtctgtcacattatcttttttgtcagttgtctcttcttccattgctaagtgatctgactccgccagggtctcccttggcagtggcttgctgtgtctggacaggctgccagcagcccttcctcagccaggcgagctccggtttcttcccacgatttgcatttccctttgcaattgattggcattttgactgtgcatcactttgcatgtgcacatgccgtgtctcctttagtgacttcatgagttaggacttttgtccccattttacagattaggacactcaagtccagaaggtagagaccacctgctcaagtcctgcagacagtgtggaaccctggcagagtctgggtccctgaaagcagacaaggacttggggacatgtcatttgttgcagatgatcccaggacacctgagtgaggacatgggaaggtgagcccggaggaggggagccggagagtggccctgatgagcgagctctgtggtgggtgcctgggctcagtctcggctcagcctcccggcgtcccacctggatgggctccatctctgtggtaaagggtcgtccctggggcattaatgctcttgctctgctggctggtgctggcatgcaaacgaggacgctcccatggggtttgaagtacatgatgctatcccacctgggagcaggggtcttcaccgtggatccctgttctgttctaaacaggcacagagcctgaagcgaatgcaccaggagctttgtaagtagaaaaaccccagcacagccaggggctgcatcctctctgcctccctggggtctccgcacgcatccccccttgtgagaggctggagtaagtagcctcctcgggccaggaaagctcagccgggagggcacctggtgcctgagaggtaaccgtagtgcctggtcaagctcggagcagcgggctgggtgagggagctgcctagtacagccaggccagacgcaggtgtagtcatctaccagctgccatcagtgtgtgccgggggctggagcgcaaaccgtgtcatctgcaaccaagcctctgaggtggagaacgtggttcccccatgctctaagtgggaaatggaagcacctcctggccagtaagtagcagtgctggggatttagccaggtctgtctggctcaaagacaaggctccccactctcctgggaacggggagccctgagcaaggatgtcagaggccaggaggcggcctctcctgtacaggtgtcctgggctaccccaaccccctcccacacctgccacacctggcctgtcccccatctagaacacttcatgtctgacggtgtccacacgcacacacctgagacca

>hepsi1_10810_3172_bre_gagputein

MQpSQSKesgTPhSGTpANFmhKNYGPRTcafleKWVnLTkEnLELRWLqwGSFNLDkIvyLEAyzkRGnEKPHKtVgCVFFywyAEAPkrVnESkiDSLKDSLQKANekLKQQTnDmmKedcTVTDlILTVPSLYPSLPTYSEStNLFAkLPfhkKzKICIyyPFTLkTikkIIISRqApYKVRlSdQScLPAVtTFtPWskTeLrAIvrDLPdPrENPQKfTeEFRILIgaYDPglpDLHzcIpViLgPgKaRKwTAAaEwGKPEEDvkDpSkSssQEgPKVAgkIAenLLNSiPKIFPqkmDWSSYNLVnITkKKIsVSDyRThLEmlfvKhSGiQvqqEvFPARteTALSaLFVnGlhPELSnLiKIheLGzEvtDMTKLLALagHFERTLEQEKTQKaNKLmPsAITVTGtGTQGInhFmypShfKSQsGGPRtRSSLpQDvClYCKQPGHWKRDCPLfyqStNEPPfRPnCfTTrGSprDLrPPDnnqP

>hepsi1_10810_3172_bre_proputein

invEkQvkingElcTVLGDMgaTLsTinptLisQQIPwSKKvIsVvGvSnQvzEvPVsePiqlaLGpfsEkHFftMlyCPVnLLGRDLLSKLKGHiKffsGeITLef

>hepsi1_10810_3172_bre_polputein

PdsPglELLcCLqTeIYkiETqacnTPDlSkISKcFWAsspTdMVRiksLepIkVQRdrSkPlpKlpQYPlkPEAiQGlsPIvedFikQGliiPCtSPcNsPvLPVKKPngqgwRfVzDLRafNKiVtkIPvVPNPdtLLSnvPiSskWsTViDLclAFFsipvHSQyLFAFtWKNrQyTWavkPlGFtKaTsyFsqALHqDLitlQfPQnctLiQYVDDLLsPTkECsEidsvyLLQqLayKdhkAzmeKlQfskekfhYLGhdLtaKdfLsPgRikTVqSfPzPeTkRQLRgFLGlTGYCRsWvPnFSLigsPLYeLTKnavPeplPWEDsHeQAFsQmKlALqqpPALGLPnypKPFTLFVhEcdNQAlGVLTQerGaKhRsiAYdSlqLgPdskAySnCLKAVAAAakLVeAssDLvLGselNLqTSHAVESLlNSNqTqHfSvskLTfyElllSPnlhLkccNllnPATLLPLPddgEEHnCvnvvPEIvApHvdlQdTPvDnPGfiLfvDrSypkIsEGKyqAeyAVTiqnEzIeKetLPqfkSArtAELfVLTQvchiAEdKsvNIYTaSRYAFgvvHdfGvIwKlzGfLTSsGtsIKNrpqvnepLsAIlLPlztVvtkIeaHtcRtEpKyqGNaLAnfyAKsAsAETvkkcnLnELhwIkPsPlPYDDLLskQcsaPdLEkQnwyLkGcKfnVmhrlTegpdGrlVlpEslklPllKTlhsTAhhgTdKmiqImkkYwsGdcskIaKmVynqcLtcqThNPgKTikTsGHifpPpdeQfEhlqglnlvvTrngvsvcScnsLhVfqkgApseswNcTPvGdFkvkLTkEIsLeAdsiLVldSFPkiMdqDFsiiRkaLcVfLFssssALiLsfsLQanpwHhnQWmaLAqVYalaQnQsncwVcglmPkKnQemipLVpvplRvPSeSyLklReeqkViLdmLnitatcflHSLKTTltfSIdkSiiTKykKTIqSk

**HEPSI2**

>hepsi2_10820_100001_con_chaindnarm

tgagattatgagagaAaaGGCAagaaTGaaTGTaaaaCCatACCtTCtaCttaaAaGAaAaatatataTTTatgAAtatCaatTgggTTTCtgtTTaAgAtaTataaagtCTCaaaATgAaatATatttaAAaaAaTaGaCaTATgTaaCatGtGaGtAtCCtaaAtTataGtaaatAAAattaAaaaataaTTGAaATtttCCTaGtTtAtattCaTGaTtaAttAgaaAaaaAttgAaaaAAaaaGGCtCTaaaaTaaAatAGatCaatTattaaaattAtaTTgaaCagtaTaTaaaagaaaCCAAtAAGAgaaTtTGTTaAaGttTgggattTCtTCaCaGgAaGAAaataaaaaaaaatTgTgatGaatAATTTgataTTTaAAAAAaAaaaaaaAAgaCCTCtTttattaTTttTaGgatTtTTTatttgtTattATCaGaatAagttaaGCataTCTaCtTttTCaTCAttgtAgaaCaGttgtTCaGgtTgaaTtTCAtaAatAtgTaatGaggtGtAGatGaGaAttTGtCTTGCaaaTTCTCaaTaATaattAAAGaTAgaGtTaAaAAggaCaAttaaGaCTTaatTtatCCTtAtaattAgAGtACTCAatATTGtAtAAaaTatGaaaTtaaTaTTtGagtTTtTGaTaataTtTtTTatTtAtTTaTaTCaattattTtAgaTGTTatTTTagaaaGTAaaGCTaCAGatttaCTaggtaTatTGGATTtGaCCAACtttttggAAAaCtTttAGaTaaTaaTaagaaAtaTtCCTaTaAaGaaataAaAGCTttaCtTtaTaagatttTtTTAGtCaattaAaAAaTAgaTGAtGaaaTataaAaaaAAtAaTgtttAaaAaaTaCAagtGagTCaGaGatttAtaaTTTgagaTtaGAAGAAaaTatTTAaagaatAaTCaggTaAGatAGtaatagtAAatAagaAaaTGATattCtatgCaaTtagaattTgAtAaAttGTtagAtGaAGaaataAtACCAGTaaTaAaaaaatAACagtCtaGaaaaaaTatGTCtCaAAgtagCAtaaTTTGGaTgaAataaaCCCatCgAtTttgTCAaAtCTtAAgtAAAaaagaAAaaaAtGaGaAatGAaaCaaatAAtatGGCTaAaaCAaAGtTaTaaaatAaaAatTaaaACaatTAtAaACtCaatCCAGGtaTataaAAaaCACtTAtggtgAAatgTCAtaAaatAttTAAtCaatTGgattaCaaTaATtaaaGgAaAttttAaCaCTatAaAaataGATtGgaaaaaaCTaAaaTGtgaaaAaTAGtataCataTGAAgaAaaAaatAAAAgGaAGaaaaaaaAtTaaaCAAggAgAaTtGaAGaGaTatTTTCaaTGttaCCTAgaGAgaatCaaataaaataaAaATtAttTtAaaTgttaAAtAAgtCAtACAAACAaCTTataaATatTaaagaaagAtTaTCTaaAaTaaaatCtatCTTAatAgAAatCCCtAtaatttgtTataaaCaaataGttCAagTggCgAgtttCACTaAtACtCCtataTtagtgCatAaaTCTCaGaattTggCttatCAaATatGTaaCtTaCaagagattCATGTTtTaaTtTaAgaTCtaaCagTCCttgtaaCCAttCAtttaAtAGaaagtatTAGaaataTCtaAtatCCAaaattttattTCCtaAaAatttGaaAaTGtaatTaGaAttataaCAtataaAAaAatTaaAAAaattaGACaCtaaAgatttAaaaCTgatGAtCattCTaatTTagaAAtaaCCgtTaAtataTagaGaAAggttagTGAaTTaTtaAgtAAtGaaGaattaCAaAaGCTGtAaaaGCAGtaCaTAAagAATTattGtaaaGtCCtCCAtaaaaattaAaaaAaTTatCttaagaatTtgAATTaAaaatCAaAaaCaaTGAtCaAaGCtaTTCaGAaCttTAAgaaaTagCACatAaGaTtaaGGtCTtGGAAgTAAAgTAAgaaATAtCattatAaAtTAtAgTaaaCAGAgaCTGtagagACTTAatCCtTGaAAGtCCaaaTAaaaCTaCAATaaCtAgCCgtgAGAAatCaAaAaaAtAGAgagtAaAGaTtTGTataGaATgaaaaAataaTtaataAaATAgtaaTTCtTtaaaATTCaaaAGtGttaaagattGagAtAaaaTtggCaGCaATgCCaaCaGAAggtaAATtaaTtAaTaTTAgAgatTTATaTtATataTTtTtaatgAtaTaataTGaTaaAAAtAaTtAattCtTatatTCttTCaCtTGGaaataCaGACaAtAAataCtaATGattAtGtttTGatGAtagACgAAgatCtCAatTtatttaagaCTTACAAGaaaTGaaAaaaGAtaattttaAtaTaGAaTttTAaAaAaATggattTTAaTaaAaaatATGGatGATttAaaTTTtaGatCAGaAtATaatgaaGtCTCTaTAatAGAtGtaaTTgAagtTGtTaCaatattTgAatttAAAtgaatagAAGGtgtCAAAttaAaaaCttTaatTtaAaaTTtagGTCaattAatAaaAaAtAAaGatgtaaaCATaaatttAgAtataatAAaCATTaatGAaatttTCatGatttaaaAaTaaGAAAgaAtaAGAGGATaTtTaaggttgtCAaaatAAtAaAaAAAaTGGAttattaAAaaAAaaCTCAGAgCTaTtAtAaAtTaTTtTaAAatAAaACaattgAgaGttttaaaAATaGataaatgaAaATtaGatAaCACTAaAAGtaaatAaAAaaaATTTgTATaGaTaCagCTGaaaaaagaaCATTCAAattAtaAtAttgaaTaCAaaaaaaTtaTtaTAAaaCCtGAaatAtTtaaAaaCaaaaagaaGGaaAtCtAtAaTAtAtCtaCAagAaAatAtAaaTgCAgTTGGAtAataGataGaGGAaTAtCaTgTtTCaTGAaAgTaAaAaAatgaAttGaCtTaaTAaTAAAgCAattAaataAATaGtaattgAaattCCCCTtAatGaaTtTGTTaaaaATtttAtAgatGaAaTaCataTAAaattATAtCatAgTgaaaAtaATttaatTaGAaaCtatCCTCtaaaGaaGtataAaaGgTTTaTtCaCattaTatTATaaattCTaAGtaTaATCaaCtaAAttgtatatttGCtCCTaCaTtAaGatgAaaaaCaataTgatttTgtAaCttGAgCTTaTgAatTattGtatCTTaAtaTaaaCCtaAGCaaaaaTCCtCTtAatattACtaATGTtTtAtTaAtaGattGaaTGTttTtaaAagaTGaaTTtaaaaTtTttCaTGtAGtttAaGCaAtAGtGaTgttAGtTGaAaAAaTttattagaAaAAAtTatTtaaCTttgaGaAGtCAataTtaaCTtaACatgatAGaAaAAtTgATatTatTaaTtAgAGtTatTCAaaataaaaaaGaAATaattGCtaATATTtaaACAgataaCaaaTaTGCCTatgaagtCtaGTgattataGaatGCtaaaaaaACaAATaGaaTaaTTAAaaCataaTttGgTaAGTTCATAAaaaaaataCACCTtatTTgaCtaaAataaTtagaaGgtataTtaTAaCCaaaattCCAgaTtattAaAaATCAaaTAttCCgtaAaGAAAatAaAACAaaAAaaGaaCAagtataTGataAgCAAAaaAattaaCtCtaAAtaTaTCTaggCAAGAaAaAtAaatTatAtTAatTTaaAAGaaACtCaTaagTtaaCaTaAaAaaAGCtaAGaTTtGagtTAAAagaGAAtaagaaaaaTAGaaaACaaaatGaaGAaaAtaTTCCtTaattATaAtaTaaaGaaTGaagaaTtTGaCTagtTatAaaatatAtTtAATaAaaGtAtTattTTtaACttaaaTatAaGaattaAtaCaaaAtAgTatTaAtAAAaCgaTttgtTGtaaAAAttAGaatCAtTatAAACTtaTgtaTTaAtACCaCtaatTaAaAaAgaCAgaaaCTgCtaaaTattCTgaAAaTattAtCAAGGgagAaCTTtACaTagatCTCAatAACAtTtaaCTTCAattgAaGaCtagtagCAaagtATaGCaaTTatatatTattaAatACCtaaGaCAtaaGaattCAGGtaTGTataaaaaAtaAtatgTATGaaaCCtCAatagaCAGaAaCAttttTgTATACAaAAtAatAtaTAgTtaGTatTaaaTGATAtttatttggaaAaAAaaATTaTtCTaGAgaTACtGagaaCttCTAgaattTtgTagaGAaaaaGatATTTaCtTtatCaGAtaTaaTaTCCatTtAaTAttTAAaATGtggAATTttaCAACaTTtttatTaTTtATATgaCtCTaAAaCaTCTtGgttAgTataAtgCAtaaaTGaaATAAgaaAAACttaaTTCatAaAATaaaCTaAGGaTTaTaaAaatataTtaTaAAAaGaTgttTgatTGaTaTtGCaGAatCatagaaaTtaaGTatCTCCAaCtaagGtaATaaaGGTaGatATTTgaaaCtaTtACaAGGaaAatAttAatCtAtAATaCaAAAaagaAtaTATTACATtACaGaAATGGaTTtaTAaaataAgTaattaaAaAatAaAAtattaaatAaAaATAaaTattttATtaaAAAtaCTgaaaAgCAAaaAaAtGtaAAaAaGaatTTCAaggaaAAGaattaGCttACtaGaaaggataTCTtataaaaGaaTttCCtCAACtAAAAtAGAAaGGTggCaAagAtGtaCttaAAgAAtAattgattTtCAAaattaGAaaatatAAAtTaTGGAaTgagatttTtATCTaaagaaGGtTgAtCCtCtTgAaTtaaCtaACTtAgtgaAAgATGaTCtTTtAtttaaGtaaaaaGaaCaAaaAAAaGACaaCaAaGataaATaGttttCCAtaAtACtAgtCaAGagaTaTGttGGTaaATAatgCtTatAgaaATttaaTaGTGACtatTatAaaaATTGtTttaaaAAtCTtgttaATTGtTattAaTaataATaATAaaaAaAATTTgTttCatatCtttTaTTtgCTTaCaATttTAtTaTGtTaataCaAtaTTaGTtaTaCAACTtaAataGgTAGaaTaATGaTTGttCCaaaGagTtaaTgTaaataAtGAtataaTaaCAtTaGatTaAaCtAttttaaAGGatTGCTattgaggaaaCttaTtTGaTGgtTatATCTGttaCTaGATtCCTAtATTTgTTaATtaCCaaTaTAtgTtaTTTgtaataCCTaTtatTAATtTTtaaGatGtaCgaaaTGttAatAattAttCataCTAatCaaggTtgaAgCTGAaTTTttaaGaatAAAttATtCaaGaAAttAAtGAatagtTCaAaAaaaaaAatAAAAatAttgtAaaAAataaaCTgAGAagaaAtatagattTtgCtTttaaaATtCTaTttCtaAaTGAaTTaAaAaAaaaAaTGgtGaAAAtaaTGAAaaTAaattTTGtgAaCttAaaCttattAattCAaaaaaaaaaatCAaGttCTtGgAAaTatGtaAaaAAGgaaatCTCTggatggaCATaaTGgTttCTAaatAagaTGttaataAGatgAatAAtgaACATACCttCtaAaaGaAaTTttTTGTGgTCtCCCAAGaTaTttAttttaAaGtgATaTCaataaaaaTtgattAttCAAattTGaataGatAaCtTTgtgtCTttAtAGCTGgtaaatAaCaTAtGtttttAgaGaAtTtaaaaaCatCCttaTaCatACATaaaaataTaaaataaCTGCttTatTgTCTaCATTaAaagtTAaaTAAAAataCaaatTtAgaaaGatAtgatgataACtaAGGtAaaAAtattTaTtaTaTaCTaatCCTaagtgCTgaaATtaAtATtatagaGaTATtatTCAAaAtCtatCaGgCatTatttgTttaAataTTGAAGaCattaaAAaaatCaTtagAGaaaaaCAaAaAtCTtTgaatTCCttaGgTTatGTGTtatTAaaTtaaCAaATaaaCCTgaaatatggagaaaaggaagagttg

>hepsi2_10820_100001_con_polputein

phflfpkgemylelehrkklkildtkhlkldpsklqialhvsrkvtellsneelqkllkavlnqlwsksstyiekiilaapikiqihzsklllnlkqyhlrskalezithiildyinrdzlipctspcntpilpvqpvtnRGzrfvzdlrtinnivipcnsvvpnppvlvaaiwsegElfgItnlwavifslSvpKNsqfLfSlswpmtQaIqmvtpWgttwSptlfLqvLdKdflnvllkkstliqymddllfcsedaslyvqWsslVatltleGiigLqgktlvlnlghpisnkdlfinvdretlmklscsktkkqlrgflglakzzrnwiPapkssdyiyslvwdkpgplewteenqltleaikkdfidalalghsnynalallNTigililkvgDwnkitgcyslqldpvagglisFmevIcataLlvkqpnkvmetpltvfvphsievllssyhtqhysvsrlpsyevzllsaphiinsecdhlnpaTllPgPsDEtPHDcVlmayQlLtlgiDLsemPLtsanvewfTDGlsLkDgfGifragYAvVfLaEvvEagpLPqAtlAqgAKLIaLiqACqLaKgiaANIptdllsafgvlglgmlvKgigfLTSSGQfIkNgqptlravsnllqaiLlpvvqIignsiLdttKsgitnlllllaKKfalsistQsKqmIltfetlpNmlKlaqfgAltaEqstwKttGgtlSletnIlyGLcnlPllcwdvqsllftvviiltdgipiKvvasgNnvtgnlplnativYsvtAIspqniikglLyialgnffLpagPfqgmaldFiQappvmriqvcgsmlvmVsmlgrwvfafpcyiAlavavilLleKIIllwGVplELpviedliFlvkviqiivKmgPilnhisCaYHPQSSgLaEcTNgIiKTqlaKfinglklvglkwSpgaSlvLvnLgknqvSPtevITGRplclgpgitnpimlKRdILqYcnGlIcqLtKsynLVensifsglpwdpvpGktygLQPEDFaYWKgyLlKdSlQPqwKvPyqilltNpCAAkleginswihtshlkklsllneltYwlMildLSshkakkkttkvdsssmipvqpvsgk

>hepsi2_10820_1356_bre_chaindnarm

atgggcaactgtgcatgtaaaaccacaccttccaccttaaagacagacccacctttaggaaccccaactgggtttctgtttaacacctatggtgtctcttcatgaaaatattttaaaaattggtcatatgtaacttgcgaggaccccagattacagtggccaaaatgagtttcatttgaaatgcctagtttagacatgctcaattagaaaaaaaaaaaaaaaaggctctaaaataaaatagagcaattgggaaagttatcttcaacagcatttgaaagcttccaaaagagtttctgttaaagctttttcttcacaggaagaaaacaaaaaaaaattgtcttgaacaatttctgatttgaaaaagactgctgaagtcctcctccaccattcttagctccttctttatcccttattatctgaactaccttgtgcagatctaccttctcctcatcctactcctgctgctctggctccatttcacaaacatctggtgtccggtagaggaggacttgccttgctttctcaatgatggtcaaagatagagcttaaaaggcacaattaagacttccctgaccctcatcaggacagtactcattattgcacaaaatacgaactgaatatttgagtttgtgatactgtttttttctgatttatatcaactagttcacatgttagtttcagaaagtaaagctacagattgactaggtatggtggattggaccaacctcttggaaaactttcagatactgatgacagacatgcctatgatgttgctaaagctttacttgataccatccttttagtctttcaaaaaatagttgatgaaataaaatacaataatgttgacaaaatacaggtgagtcagtgattccatattttgagatttgaagaaaatctttaaacaacagtcaggtaagagagttatactaatcatcaaaatgatactctccccaattccaatttcataaatggttagatgaagaattaacaccagtaataaaaagacaacactctagttggaatgtgtctcaaactcatgatttggtgaattttgcccatccattgtctcataccttaactaaagaagaaagaaaggagaagtgaaacaaagaatagggctaacaaagttataagttaacagttaaaacaactatatacccaatccaggcttccagaatgcacctacaaacctcacaatggacctaaccctttgctttgcagttattgcaggaaacctgatcactttaaaaaggattgcagaaaactaaagtgtgaggaatagcaccagatgaagaaaaaaataaaaggaagaatagggattctccaaggaacttgaagtgatcttttctttgtctcctactgacactctgggaaaatagaaattattttaaatgaagaactcatacaaacacttgttcatattggagctacattatctgtaataaatcccaccttattacaaggccctatttcttggtgtaaacaaacagttcaagtggcgagtgtcactaatactcctgtatcggtgcataaatctcagcctgtggcttttcaaataggtaacttacaagagactcatgttttcctcttagttccaacagtccccatccatgtgatagggacttttagaattatccgatacccacattttcttttcccaaaaggggaaatgtatttagaattagaacacagaaaaaaattaaaaatattagacaccaaacatttaaaactggacccttctaagttacaaattgcccttcatgtttccagaaaggttactgaattattaagtaatgaagaattacagaagctgctaaaagcagtacttaatcaattatggtcaaagtcctccacttatattgaaaaaattatcttggctgctccaattaaaatccaaatacactgatcaaagcttcttctgaaccttaagcaataccacctaagatctaaggccttggaataaataacacatatcattttagattatataaacagagactgacttattccctgcacaagtccatgtaatactccaattctacccgtgagaaagcaaaacaatagagggtagaggtttgtataggatctaagaacaattaacaatatagtcattccttgcaattcagtagtgccaaaccccgacacattgttggctgccatcccgactgaaggtgaattctttactattacagatttatatgatgtgttctttagcatttctgttgataaaaacagtcagttcctcttttccttcacttggaaagacagacaataaatacgaatggtcacgccttggggatacacgaagagcccaacttatttcttacaagtattgaaaaaagacttcttgaatgttgacttttaaaaaaatccactttaatacaatatatggatgatttacttttctgttcagaagataagcaagcctctataagagatgggattcacttgttacaacatttgactttaaagggatacaaggtctcaaaggaaaaactttagttttaaatttaggtcacccaatatcaaataaagaccttttcattaatgtagatagaagaaacattaatgaaactttcctgttccaaaactaagaaacaactaagaggatttctggggctggcaaaataataaagaaattggattccattaaaaaaaaactcagactatatatattcttttaaaataagacaagccagggcctctggaatggacagaggaaaatcagttaacactagaagcaattaaaaaggattttatagatgccctggccttaggacattcaaattataatattgtttacatttttttaatacccgaggtattctgattctaaaacttggggatcataatagatctacaggatactatagtctgcagttggatccagtggctggaggactatcatctttcatgagagtgataaaagcaactgccctgctagtaaagcaaccaaataaatagtgatggaaactcccctcactgtctttgttccacattctatagaagtacttctaagttcataccacactcagcattattcagttagcaggctgccctcttatgaagtctgactgctttctgcacctcatattataaactctgagtgtgatcatctaaatcctgctactattctgcccctaccttaagatgaaaaaccacatgattgtgtaacctgagcttatcaattcctgtgtcttaagatagacctaagcaaaactccccttactagcactaatgtttgatttacagatggattgtccttaaaagatgaatttggaatctgtcatgtagtttatgctatagtgttcttagctgaagaaatagaaagtgcttatcttccagaagcaacattggctcaacaagcaaaattgatagtattaattagagcttgtcagctggaaaaaggaataactgctaatattcatacagacaacagatatgcctttggagtcttgtgactttggaatgctatggaaacaaatagtgttcttaacctcttctggtcagttcataagaaatagacaccttatttcacaattattagaagccatattataaccaaaatcccagatcattacaaatcagatactccggaaagaaaagaaaacaaaaaacagaacaaccagctgattagcaaagaagtttgctctaaatatatctaggcaagaaaaacaatctatattaacttttaaagaaacccctaacttgacataaaattagctcagtttggagctctaaaagcagaacaagaaaattagaaaacaaaagggggaacatattcccttaaggataacataaggaatgggctatgtgacttgcctatactttgtactgaattacagtcattattttttacttatgtatatgatttaactcatgagagtcctgataaaacggttgcttgtggaaatcagtatcattggaaactttctcttaataccaccataagatacacagtcactgccatatcttctcaaaatatcatcaagggagaactttacatagttctcaagaacactttccttcacctgaagccccctcaaggtatggcaattagattttatcaagctacccccgtcatgaggattcaggtatgttctagtgatgatctgtatgtttcctcattgggcagaaacattttttatacagaatattatatagccttagtggtaagtgatattctcctggaaaaaaaaattattctgacttacggagtccctctagaacttcctagtgacagagatatttacttcaccagacatgttatccagtcaatatgtaagatgtggccaattcttcaacattttcattgttcatatcaccctcaatcatctgggttagtagaatgcacaaatgaaataactaaaactgaattcacaaaataaactaaggcttttaaaattttgtggtcaaaagctctttcattggttttgctgaacctcagaaatgaggtatctccaactaaggtaataacaggtagacatttgaaactatcaccaggaaactatgaatccataatgcaaaaaagacacatattacattactgtaatggatttataaaacaactaactaaaaattacaatttggttgaaaaatatatattttatggtaaatcctgggaaccaaaaaaaggtaaaacccacggacttcagccagaagattttgcctactggaaaggatatcttctaaaggagtctcctcaaccaaaatagaaaggtccctaagaggtactttaagaataattgtgctgcaaggctagaaggcataaactcatggattcacacttctcatctaaagaaggctgagcctcctgaatgaactgacttactggaagatgatctttgacttaagtcaacacaaggccaagaaaaagacaactaaggtagatagttcttccatgataccagtccaggcctgtgtctggtaaataatgcttatacttatctactagtgactattataatcattgttttagaaatcttgttaattgttattattttgtatgatatagaaaatttcttccccagctcttattcccttgcccattctagtctgttgatacaatttttgttttacaactttagtaggtagttcttatggttgttcctttgtctcaatgtgtagaatgatatgatgacactttgattaaactattccaaaggactgctactgcaggaaacttatctgatggttggatctgtcacttgattcctagatttgttcatgaccaatatatgtcatttgtaatacctgtcactaatttttcagatgttcctaatgtcattacctattcagactaatctcccttcaagctgacttttcaagtccaaattattcatgaaactaatgaatgcttctatatgttgactaaaattatctgctatcaaatctactcagaaggaatttggccctggccttcttcattctattccttattgaattaagaaatggattgctgaaaacaatgaatctatgctttgtcaacttattcctctcatttcatgtaatgtgagcaggttcttgcaaatatgcaaaaaaggaaatctctggatggacataatgctctctagccatcttgttagtgaggccattaatgaacataccctcagaggagttttttgtgctctcccaagttatgtatttgcatgtgatatcaagattgattggctactcaaggttgggcaggcaacgttgctgcctctatagctggtaaatagcttatgctttttagggcattttgtaactccctcatacttacataaaaatataaaataactgcttttctctctccattacagagctaaataaaaggactatgttagaaggatatgatgataacttaggtaaaaacatttttgatatactgatcctaagtgctggaatttatatcactagagatatgattcaaaacctatcaggcactattggtcggacggttgaagacactgcaaaaggcattgcagctcaacaaaaatctttgaactccttagcttaggtgttactagataacagatttgcctgagactactggcagaaaaggaagagtctg

>hepsi2_10820_1356_bre_gagputein

MGNcAcKTTPsTLKTDPpLGPQLGFCLTPmVSLHENILKigHmzlARTPdYSgqnEFHLkcLVzTcSirKKkKKgSKiKzSNWeSyLqQHLkASkRVsVKAFSSQEEnKKKLSzTiSdLkKTASPPPPLSSffIPYyLnylVqIyLLLilLLLLwLhFTnIwcPVEEdlpcfLndGQRzSLkgtIktSLtLIrTvLiiaQNTNziFEfVILFfSDLYQLvhmLvSESKARLTRYGGLDQPLGKLSdTDDrHaYdVAkAllDtILLVFQkIVdeIKYnNVDKiQVVSDSIfzDLKkiFKqqSGkrvIliikmiSPQfQfhKwLdeeltPvIKRqhSSWNvSQTHDLVNfAhPLShTlTKEERKEKETKnraNKvISzQLkQLYTQsRLPEcTyKPHNgPNPLLCsYCrKPdHfKKDCrKlKcEEzhqmKKKikgriGilqgTZsDLSLSPTDtlGk

>hepsi2_10820_1356_bre_polputein

PhflfPkGeMyLeLEhRkkLkildtkHlkldPsklqialhvsrkvtEllSneElQkllKaVLnqlWsKssTyiEkiiLAAPikIQihzSklllnlKQYhlrskALezIthiIldyinrdzLiPCtSPcNTPiLPVrKqnnrgzRfVzDLRtINniVipcnsvVPNPdtLLaaiPtegeffTitDLyDvFFsisvdkNSQfLFsFtWKDrqzIrmvTPwGytkSPTyFlqvLkkDflnVLlkkstLiQYmDDLLfcsedASLyKrwdsLvtTfDfKGiqglKgKtLvLnLGhpiSnKDlfinvdRETlMKlscsKTkkQLRgFLGlAkzzRnWIPlkknsdyiYsLkzdKPGPleWTEEnQLtlEaIKKdfidAlALGhsnynclhFfNtRGiLilKLGdhnRstgYySlqLDPVAgGlssfmRvikAtALLVKqpnKvMetPLTVfvPHSiEvLlssyhtqhySvsRlpsYevzLLsAphiinsecdhLNPATiLPLPzdEKPHDCvtzAyqflclkiDLsktPLtstnvzfTDGlSLkdEfGIchVVyAivflAeeieSayLPeATlAqQaKLiVLirAcqLeKGitaNIhTDnRYAFgvldfGmlwKQivfLTSsGqfIrNrhlIsqLLEAILzPKsqIItnQilRkEkKtknRttszlAKKfALniSrqekqsILTfKETpnDIkLaQfGAlKAEQEnzKTKggTySLKdNiRnGLcdLpILcteLQSlffTyVyDlThESpdKtvACgnQyhwklslNtTIrYtVTaissQniikGelYiVlkntFlhlKpPqGmaiRfYqatpVmriqVcssDdlyvSslGrnIFyTEyyiALVVsdILlEkkIiLtYGvPleLpSDrdIyftrhViQSIckmWpiLqhfHCsYhPQSSGlVEctNeitKtEfTkzTKAfkilwSKaLsLVLLnlRNevsPtkvitGRHLklsPgnYEsimqkrhILhYcnGfikqltknynLVEkyiFyGkswEPkkGKTHgLqPedFAywKgYLlkEspQPKrKvPKRyfknncaaRleGinSWiHTShLkKLsLLnelTywKmIfdlSqhKAkkkTTkvDSSSmiPVQPvSGk

>hepsi2_10820_4152_bre_chaindnarm

tgcagattcactgagccagacaaaggcatgaatgactgttttcccctaccctcttctcacatgaaaattatgtatttctcaatatccttccctttcccctttagatattgaaaccctcaaaatcatctatggggaaaggaatagacctatctcccaggtgtgtatccttaactttggcaaataaacctcaaaaaatgattgagattttcctgggtcattttccttgattgaccacggagggatcctgagtgaaggtggccctggcctgcagcagctctcctattggtaccagattgggctctttatagcccaaaccaataagacaatttgttgaggtctgggacctcctccctgcaggaagttggggctctgaggtttaatttgatgtttaaaaaaaaaaaaaaaacacctctttttttttttctgggagtttttgcttgcttccatcagggaaggcaagcttgtctgcttcttcatcagtggagaacagtcttcagcttgagtctcatcactaggtaaggaggtgagttgggaatctgtcttgcaaattctctttaataactaaaggtactgttaacaaccagctggtcttaatttctccttacatttagagcactcaaattgtataatttgtgtgattgctgttcgttttgcttagatgtcttatttacttgtttctgtcttgttaggtgtttttttgtatgcttcagtccttctcttattggatttggccaactctaaaccctctagcttatagtatggaattttcctctccaaagaaataagagctcgcccttctcagcctttcttaggcaactgagaattacatgagggtgtctggaagaaacactccctaagatgtgcaacggctctgagtaggatttcccctcagaagaacgtacttagggtgtaatctcagctagcaggtgcaaataaggagctgatctcccatgccttgagcccctgacacactgtgccaggtagccacgataccagttcagcgagtaacggccctgaaaagctaggtccccaagcagcacactttggatccaacaaaccctccgacttggtcaaatctgaaggaaaactctaaattatgtggaaggagccctttaagttggctgaaacccccacagctgtggaacacaaagttccacccttagaaactctggccaggtatatgcaaaaacacttatggtgaattgtcatgcaaatatttaatcaagtggaccactataatcaaggcagattctaacctacagaggcctagatggggatcttctgagatgcccaaattagtgtacctgtgaactaggatgcaaaacgcaggcacaaaactaaacaaccagaatgggagagctactttcaatggtacctagagagtagcaaatggggggaagatcacctcatgtccttacaataagccaacaaacaacttagaaatgctaaccaaggactctctaaagttctatctctcttaaagaaatccccagaatcccttacttccctgtggcacctccccacctccacttacacccccactttacccctgacctctctgaacttcccggaccagatctgttccctccttctcctgcatgttctaatttaacatcttcctctccttgtacaccactcactcaacagaaggcagtagggagtctaacttccaagaccctacctcctgacaattccctgacagtgccagtggcagcctctcatctggaagatgtggaaaagggggaccctgcagggccatctctcatgatcaccctgttttgggaacaaccggtaacacataggggaaccccggtgattgtctaccaacgctggtcaaaggctgagttgcagggcataaagaatttctgggcccccataagatccaattgagtttgcccaagaatttgaattcattatcagaacctatgacccaggccattcagacctctaacagctggcacacatgttggtctcggaagctaaagctaaggaatagctggacaaagtacagtggtcagaccctgtagcagacttaacccttgaaggcccaatagagctacaataaccagcccccagaagtcgagaacacagacacaaagatgtgtgggaatgagcaactgctctgttaaataccatttcttcaatattccaaagggttgtggattgggataaaatccagcaatgccaccagaaccgaaatgaatcaattttagattattttatacattttgactgagataatatgctccaaataattaacttacactctctcgcctggtccttctgacgaaacccctcatgactatgttctgatgactgaccaacttctcacttctgggacagacttacaagagatgccactggataatgctgagatagaatggtatacagatgggtcttatttaagaggatggggatggaaattttagagcaggatatgctgtggtctctttactagaggtagttgaagctggtcctcttcctcaagccaaatcagctgaaggggccaaattgattgccctgactcaagcttgtcgattagcaaaagacaaggctgcaaacataccctgacacctgctaagcatttggagttgctcaggacttcggaatgtgaaaggagagaggatatttaacctcctcagggcaacccataaaaaatggacaaccaactctcagagctgttagaaactattctcaaccaaaacttttgacagttgtaaaaatcccaggtcactctacattggacaccactaaaagtggagataacaaatttgctattgctacagctgaaaaagcagcattcaagccaccagacccaatccaaaaaaatgatcataaagcctgaaacacttaaaaacatgttgaaggaaacctagagtatagccccaacaaaagagaaatccacttggaaacagacaggggaatacctgtctcctgaaactaaaatattgtatggacttaataataaaccattattccaatgggatgtcaggtgccccttaaggaatgtgttaataatctgacacgatggaatccagataaaagtatatcctagtgtaaacaatgttactggaaacgatcctccacagtggcacaaaaggtttattcccagtgtgctatttgtcctaaggataatccacggaagctcctccatgaggcccagggtcattttccccttccagctggaccttttgaggtatggcagcttgattttatccagccgccatcctctcaaggttacatgtcttagtaatggtctgcatgttttcccattgggttgaagcttttccctgcaggcaagcaacagcgatggcagttggaaaactttattagaaaaaattattccactgtggggagtccactgtgaactttacgtgatagaagaactcattttcctggtcaagttattcaaaatatttgtgaaatttggcccatatttcaacatttccattgtgcctaccatccccagtcctcagagctggcggaaaggaccaatggaataattaaaacacaattggctaagttcataaatggacttcacctctgttggcctaaagcactcccctggtgcttcttaccctctggtccaccttgggaaacatcaactatccccttatgaaattataacaggaaggcccatgtgtatgggaaccaaaataaccaacccaacttttctcaagagagatatattgcagtattataagggactcatttgtcatctttacaaaagccaagatttggtaaagaattcctttcatagtccactccctgaagacaaggttcctggttatgatctgcagcctgaagactttgtctattggaaaagacatttaataaaggactcccttcaaccccaatggaaggagccataccaaatactattaacaaatccatgtgctgcaaaaactagacagtataaactcatggatttacacctctcatcttaaaaaggcacaacctcctgagtaggctgtaactcctaccaaggatcttcacctgcagctcattaacattgaacttcaatccaggactaggagcagaccatagctgttgtggactgcttaaacctaagacacaggaccaggcctgtatacaaaagaacacctatgaaacccaagacacaggaccaggcctgtatacaaaagaacacctatgtgtattgtatgatagctattacaattattgtcctagagatactggcaactgctatcttgtgcagaacagggcatttgccttgtctgatttaatgtccttttagtagttaaaatgaatttcacaaccttgctcctatttaccctatgtctctaaaacctcttgccactgtcacccattgttgcccataagacaaacctgtttctacaatgggctcaggattatgcagacagattataaaaggatgcctgtgtgatatgcagactcatgcctctttccagtggctccagcctgccatggtgggtatttcccttacaaggtcaagactagctagaataccaaaatttattacatcacagaaatggtttcgtatccttagtgctggcataacaaaagactaaatatatataatttccccattaaaaacactcaagagcaaggacatgggaaaagattttcaagggaaaggaccagctcaccagctctcactttagcattcccccaactaaaagagaaggtggcaaccatgccccaaacaacagcccattttcaaaatgggataatgcaaatttggaatgggtttatctggttcatcccttcatttggccaactcagccaaaatgctcctttatgcttggaacgaagaaacgaccccaaggaccaatggccaaacactacaagagatatggggtggatacctggagaaattgtattgtgacctggagaaattggtggcagaagctattgcttccaataataataataataataatttgtgtcatgtcctgtttttgcttacaatgttattatggtatgtccagattagtcatcaactgaaagggctagaataatgattgcccaaagagttgctctaattgaggaggcagtagcatagcctgacccagcttccaggtttgctttcccttttgctgtatatctggtctagatccctatatttttaatttcctcttaccttctttcctccccctattttaatcttcatggggcacgacctgctaagaatgagccttcctagcaaggtgggacctgaatttctgggaataaaccatcctagcaatgaaggaccagctcaaaaaaaaaaagaaaaagaaaaaagcaagctgagaccagagacacattttccttctaaaatgctttctccaaatgattttaaagaaaaaatgggggaaatgtgaaaataaatcttgggaccccaaactcactaagccaaaaggaaaagtcaagctgggaactgggttatgcaagcctgcctctcattttggttcctaaataagatggctacaagatgaaaagctacatacctccctcacaaggaaattccttgtggtccccaagatcttcaccctaaagcatttctgttaaagtgcatcatgcaaatgtgaattgatagcttttcttcacagctgcggggaacataggacagaactcaaagtcatccctctgcacacattgtttcctctgccctattgtctacattaatcttatgtaaaaatgcagattcactgagccagaccaaggcaggaatggctattttctcctacccctctcacatgaacattgggtatttctcaatatcctgccctttcctctttaaatattgaagccctcaaaatcatcttcagagaaaggcatagacctgtttcccggttgtgtgtccttaactctggcaaataaacctcca

>hepsi2_10820_4152_bre_polputein

eQpVThRGTpViVyQRwSKaELqgIKNFWApiRQLslPknLnSlSePmtqAIQtSnswhtcwsRKLkLrnLdkvQWsdPVadlTlegPieLqzpaprsrEhrHkdVwezataLLnTisSifQrVvDwDkiqqChqnrnESiLdyFihfDEIIcskzLTyTLspGPsDEtPHDyVLmtDqLLTSGTdLQEmpLdnAeIewyTdGsyLrDGdgnfrAgyAVvSlleVvEAGpLPqAkSAegAkLiaLTqacrLakDKAaNIpzhLLSIwscSGlRnVKGRgYLTsSGqPIKnGQPTLrAVrnySQPKlLTvvkipgHStLDTtkSGITNLLlLqlKkQHSSHQTQSKKmIikpETlKnmLkETzSIApTKEKsTwkqTGEYLSpEtKILygLnnKpLFqwdvrcPlRnVlIIzHdGiQiKvypSvNnVTGNdppQAqKVySQcaIcPkdNPrKLLhEaqGHfplPAGPfEvWqlDFIqPpssqgYmLVmVcmFShWVEAFPcrqaTAmaVKtLLEkIiPlwGvhcelyvieelIflvklfKifVkfGPyFnIScAYhPQSSelaERtNgiIKTqLaKfinGLhLVgLkhsPGasyPlvhLGkhqLSPyEiiTGrPmcmgTkiTNPTFLkRdiLqyYkgLicHlyksQDLvKnsfhsPLpeDkVPgydlQPeDfVyWkRHliKdSLqPqWKePYqILLTnPcAAKTRqyKLmDLhLSS

**HEPSI3**

>hepsi3_10830_4269_bre_chaindnarm

tgaaattgccctgcaaagtgtcttgtgggaaaaatccacatttcccttttgttttccttttctttccagatcctggagataatcaactaagagccaggcacccttttaggtctgataagaaacattttacaacctgctttctctctgaactctgctatctgagagattcctctgcactctgcataataaaacctggtctctatgatcctttttctctatgatcctttttcttaacttgaacattcctttgcattaaccccaggtcttcaggtgaactcaaccaattgtcaaccagaaaatgtttaaatttacttatagcctggaaacccccgctttgaggtgtcccacctttcggaaccaaaccaatgtatttcttaaatgtatttgattgatgtctcatgcctccctaaaatatataaaaccgagctgtaccccgaccacctgggtcatatgttctcaggacctcctaagggctgtgttataggccatggtaacttatatatggctcagaataaatctcttcaaatattttacagggtttgactcttttcaccgacaataatctggtgcccaacattgggcctcggagaagactcaggaccctgaaggagttgcccaaaactggagctaaggtaccagcaggagcccattgaagcctcaccgaattcgagcttctccttcggtggaactgggaagttctcctgagccccaacctcctgttttggttgatggtccttgatttattctaagctgattttttcctctctgtcttttctcctaggaagttggttaaaatcttaattttagtttcagaagtgcattcaaaaggatctttctccattgccttttctctcaaaattaatctcaattggcttgtctgtgcacactttgcgtgaggaactgaactgttgtttttcataagtaagtgagagactgagttcctcagctctgaagtgaaagggcattttgctcctcccagctgaaaggcacctctgggtgaccgggggcctcgtgggagtgtctgcggagggcggggggcggggggggttgatcccctgtaatgtgcagcggccctataggaaactctccaacaaatattaatttaaaaaggctcatccaggaaatgcacataagggctgatcacccagcgtcttgagccctctcagaggtgatagacctctgaagagagaaattgagacatgtaagagtgtagaaataactcagtggtgacacactgtggagtgctactgacaggcagcacatatcgatccaccacacaaaaaccttagtccgcaggtcagttccctaaaaaaaaaaaaaaacaaaaaagataggaaataaatcctccaaaaaagaggaaaggcaaggagagtggccccctgtgggcactccaactgagtttatgtttaataattgtggtgcctttacttgcaattatttatgtaaatggaaagatcttactaaagatgacctaggtttaaggtttccaaaatagggaactttgatattcctaaattggctttcctgcatgctagattagacaaattaggctcccaaatcaaagaaaatgaatggcaatcgtactttagctggcatctagaatcatcaaaaagagggaaagataagcttgccttcctccaggaagttaatttaaaaatttttgaaactgtttcagaatttaaaaaaaccagtaaaagtcacttctgaaatcaggaaggctccagaaactgactccccttctgctcctcctgaccctctactctctgaacttccttatccagacaatttcatttttcctcctcctttccctcctcccttgcctcaaaccacagggggaccagaaatagctgcagaaaataatatagtagttgctccttttagagtaaaacccataagcagaggggattctaacataatatatgccccctggaccaaatcatagttaagtccctggtatctggattcctcaatccgattgaagatccctttgggtttgcttaggaattccagttaagtcttaagatttatgacccagggttctctgacctatatcagctgattcagttactggtctccaaaaacaaagctgaagaatggtttagagtagcagattggaaacagcctttagaagattttgacaaaatagatgcaactggaagagagaaatgcagagagctctgcaatcacctctgtgacactgtaccacagatattccccaaggttatagattgatagattgagcaaaggtgcaacaatgtaaaatacacccaaacgagatcatgcctgacttttatatcaggtttgaaaagacattcaaagagttttcaggaatacctcctgaaaactttgagcacggtaaaagtgacaacttactcaattctgagtttattcaaggtttagataaagagttagcaaccctaataaagaagaataatgtgacttgggcttcctttcccactagccacctagtcaccctagctgaccagttgtcacaagcaatcattaagaaagaaaaggaaatagtctctaaaattataagtttacaactaaaacaacttagtaaccaagttgggagtttgcaagggtcccatggtctccagagatctaagcaaccccaagaggaagcaatttgccactattgcaaaaagaaaggacattttttaaaataatgtaaaaaacttaaatgggtgcttgctcaacatgggcaaaggaccccaaaggaaggcaccagaacaactcaaaaatcagaataaggatgctctgagggaatagagagggttttccccctacttctaactaatgctttaggagaagtagaaatatccataaatggggaaaatatcaaagcccttgtcgacatcagcactacactgtcagtcttcaaccctaccttgattaaaaaccctctcccttggagtaaagaaaaagtacacatggtaggggtttcaaactctcctattacagcctttaaatcaaatccccttcaatttcaactaggagaactagtgagggatcacatttttcttatagtagatagtgtcacacacacacctgctggaacgggatttcctagagacccataatgctcatatctcattttcacaaaagggagaaattattctcaacttgggagacttacaaaaacccacatgtagtattgtgcttgggaaagtcaatcaagattctggacaggaagaattagagcctttcttatctaaagtaccagactccttatgggcaagctcttccacaaatattgggagaattaagtcagcagtccctatagagataaccataaataaatccaaacctctgccaaatgtgaggcagtatctccttagacctgaagccttcctaggaatcaaaccaatcattcaggactatttagacaaagaactcatagtaccctgcaccagcccctgtagtacaccagtcctcccagttaaaaagccaaatgagaaaggctggagatttgttcaagacctaagagctataaataaaataatagttcctagacaccctgttgttcctaactcccacaccttactgtccaatattcctatcactgcagccacctctctgttattgattaatgtagtgcatttttcagcatacctgtagagcaaaatagccagtatcttttcccttccacttgggacaatcaccaatacacatagacagtcttaccccagggctactctgaaagtcctacttatttttctcaaatactaaaggcagatttcgatgacattgagtttccaaatgaatgtactctaactcagtatgtggatgatttattgttcgtcctccctaccaaaatgcagagaagatacagtccttaaacagcttgcttttaagggacacaaagtgccaaaagataaattacaattttgcctcccccaagttaaatatttgggacatatgatttccccaagggggcttttaataaaccctaagagaatctctgctgttatgacatttcccctgcccaaaactaaaaaacaattaagaggcttctcacagttaacagtctattacaggagctggattccgtactactgcttaattgctcaacccctatataaaaaactaaaacagacccagccagacccaatccactgggaagagagggaaaaaacaacacatagaagatctaaaacaggctctcacccaagcacttgctataggccaccccaattacagtcttcctttttccctctttgtacatgaaataaatgggaatgctctaggagtattaactcaaaaacatggtgacaatcacagaccaattggttattttaggcaacagttagattgagttgcaagggggcaccctccttgcatgagggctatatcagctgcagccacattatgcaaaaacattgaagaatttgtcatagggtcccctctgaccatctatgtcccccactcatgaaatctcttctaagctgtcactacacttaacaatattctgttagccaccttgcctcttatgaggtactcctgctttctgccttcaacatcactttgaagtgctgtaacactcttaaccctgctaccctggtctcccttccaggtggacaacaaaaggaagaggaacatgactgcactctactaactaatatcttactctcctctaaggaagatttataggaaaactcccattgagaatgctgaattaatttggttcacagatggttgttatttaagggatattaccaggcaggatacaccatcacttccataacagacataatcgaaagtgcccaactccaaggagtcaaatcagcccagatggctgaattaatagcattaactagacaccaactgaaggattccttacaacccctccagaaaggacctttgcaggtgcttttaaccaactcttgctctgcacgttaaagagagtcgcttcttggattcacattttacacctaaaatgagcacctgaccacattcctgactgcccctgaacttccgcagcagtctctgacacccacctgcaaatcaagagaacactggaggctcagccaatctctagggctacaacaaagtcaacatacttccagagatgctggaccaggctagtcctcatgaaccctaaaataaccatcatttttactttccttgctatagaatattgctaaaacacaaatgttttaactgagacccccaagaagctgtttggaatctatgcaatcttctttggtataactttctaaatggatgtggctacacattcccttgctaaagccaggaaaatgttttacttgttcattctcattcccacaatctcctctgtctacctaccccacactcctctttccaacctgaaaatttctcccagtcccctgtaaacctcagcagtcttcctgcaggcagagcccacatcatttggtatgatatagaccaggaggaaatgaagactgttatatggaagagcaccattctaaacattccccaaggtccccactttttaggtacccctattatagggctgcaaccaaatatgacgccctgcaggaatactttttctaacaatgcctcaggatgatcactgagggggtctaaccaggtagaacgttgcataacaatagatattggggatgatcacttgagacaactgccttgttacaatgtctcctgtgtaaactgccatagcatggcctgcccaaataactaccagccctgtgcttgtggctggatgctcctatccagtaccaaccacactgacactgcccttaaataactatttatggaagtcaattggtggacagaagtccctgacagcacaaattccaccctaaaaccagggcgccactgggaagccttatggaatacccagacaagagcatacttaggtgtaacctccactaacctaacaattgtttgggatacccagcagttctgcattgactccatttatccaaattgtacaaatggcatcagtatacaatggcaacagcattcttgatggatacacgaaaattggccacccaaaaaactaaagcatgacctcactggaggagtcagagctgggcaaggcatactggggcaagctgagttcatggctaatgaagagagacattctttagaaaaaaaaatccctatcaaaaataggccacatagaaggaatgctattccaggaagagggaaaaggatgggaagctgctttaaaagataatgcagcactcatcaaatggatagaagaaaccagacaaaccatgaaaacatactcccaaatccaaagatgggaacaagcatctactcttacctgtaggggctcatgtctattttattacaaatggaatcagaggtggatatgaagcatggtctacagtcctcagtactgaggcacaatccaaacacctgtggaaaatatgtggtcctccaaacttatggagatttttggacgttaagtgtgatcttaaacattgtattctcagagaacaggcaccgaagttaaatgaggctcaaaggtactacccctgggaaataggtgggctattttagctgataataacacatggtattccatctccctttcagattatgaaaagagaaaaggagaatggctatggcctcaaaccacttggaaccctgattttcctaaacttggcctctaatatccacccccatggaacatagcatctggtatataaggaagggccatttctgttgggagggacaacaaaatgacactgtagaattacatgacttctcctgtaacaaaacctcctttctctcccaaataccagtatatggtgcaatacaaaggtggtgggaaaaatagatttgtccactgttaataagtcctataacaatttagacattgagggccaggcattccttgcatttgatttataccctcctcaggatgttatacccatggagataaattggccggaagagagactaaatttattagattctgatttagtgtcagtcctacaatcctcaaataagatttatcataaggttcaaatgacccttgataaggagggtaaacacattgtgagtctgatcaaagaatatgatgatgcatgcagtggttttttttgctggttaggttgtttactgccctccgactctacctgtaaccttcttggatgcctaatctttgctatcttgggtattttggtattcgtctcggtatttatcttggtatttgtcactgtattagcattatatatctcttgtaaatgttatgccagttacagcaaaatgaacaaggcacaattaaagacccagatcatgatagctcacaaaatagatatgatctgggatttttttatactaaaccctaggtctgactccatctcaccccttaaactactggttattacaccaggtcagaccatttcctcccgccatgatccaaattgcaaatacttaaaattattatcacccaaccagactactctaggaatgagcgttcctagcactgtgggacctgttgctgtttgttggcctgcatatgcattttgtggaatgctttttggccaagaagggggatggaggactaagctctgtttttcattttgcccaaattcctacctaaggggtccagggagtcatgccctacaaaccataaattctcatcagatgggttttatctgaccctgtatatcacgacttacttttcaatctgactctggcataatattatgagacaaggaaaaaatacttaatcccaaaatatatttccttgccataccttgaaattgccctgcaaagtgtcttgtggaaaaaatccacattctatagagaatcccctttcccttttgttttccttccttcctttgcagatccaggagataatcagctaagagccaggcacccttttaggtccaataagaaacattttaca

>hepsi3_10830_4269_bre_gagputein

iGNSPTnInLkRLIqEmhIraDhSVLsPLRgDrPLKREiEtcKSvEITQgDTLwsATDRqHISihhTkTLVrRSVPzKKKKTKKIGNkSSkKEerQgewPPVgTPTefmFnncgAftcNyLcKwKDLTKDDLfKVSkigNfdiPKLAfLhArLDKLGSqIKeNEwqsyFSwhlESSkRGkdkLAfLQEvNlkifETVSELKKPVkVtSEirkAPeTDsPsAPpdpLlselPyPdnFIfPpPFpPpLpQtTGGPeiaaENniVvApFRVkpISRgDSNIIYaPWtKIVKSLVSGfLnpiEDPFGfAzeFQlSlKIYDPGfSDLYQliQLLVSKNkAeewfRvAdwkQpLeDfDKIdaTGrEKcrELCnHLcdtvPQIfPkViDzzIEQRcNNvKyTQtrScLTFISGLKTFKEFSgiPPeNFEhGKSdnlLNsEfiQglDKElaTliKKNnvtwasfptshlvtladqlsQaiiKKEKEiVSKIISLQLKQlSnQvGSlQGShGlqRSKQPqEeAIChYCKKKGHfLKNVKNLNGCLLnmGk

>hepsi3_10830_4269_bre_polputein

ptliknpLpwSKEkVhMVGVSnSpiTAfkSnPLqfQLGELVrdHIFLivDSvTTHLLErDfLETHNahISfSQKgEIiLnlgdlQKptcsivlgkvnqdsgqeeLepfLSKvPdSLWAssSTNIGRIKsAVPIEITiNKSKPLPnVRqyLLrPEAfLGIKPIIQDyLdKELIVPCTSPCsTPVLPVKKPneKGWRFVQDLRAINKIIVPrHPVVPNShTlLSNIPITAHLsVIDzcSAFFSIPVEqnSQYLFpsTWDNHQYTzTVLPQGyseSPTYFSQILKaDFddIeFPnECTLtQYVDDLLFVLPTKMQRRYSLKQLAFKGHKVPKDKLQFCLPQVKYLGHMISPRGLLINPKRISAVMTfPLPKTKKQLRGFSQLTVYYRSWIPYYCLIAqPLyKKLkQTQPDpIHWEEreKTTHRRSKTGLTQALAIGHPNYSlPFSLfVHEiNGNALGVLtQKHGDnHRPIGyFRQQLdzVARGhPPCMRAISAAATLcKNIEEfVIGSPLTIYVPHSKSLlScHYTzqySvShLAsyEVLLLSAFNITLKCcNTLnPATlVSlPgGQQKEEEhDCTLlTNILLSSRKIYRKtPiEnAENLVHRWLLFKGYYqAGYTITSITDIIESAQLQGVKSAQmAELIAlTrhQLKDSlQPLQKGPLQVLLTNSCSALKRVASWIHILhLKzAPDHIPDcPzTSAAVSDTHLQIKRTLEAQPISRATTKSTyFQRCWTRLVLMNPKITIIFTFLAIELLKHKCFn

**HEPSI4**

>hepsi4_10840_5359_bre_chaindnarm

tgcttggctatataaaagggtgagatttcctgctacctttgcaatctcattagcagattgcctgtgatgcaaatcagtgggcttaatgcttattcaataataaaactgttttctttcttccctacatttgtagtcaggatttcctgtgttgccagattttacttttaattttcccctagcaatctgctgatgaggatgggatggcctggaagttcctgaatcgagcagaccagctgaaggtgaattataaaatgtctcccttctagctctgtgctattttcaaatactagccagacaaatcatcttttccaaattccagattttatgattgagaagcatgtgtaatgcagaacctataactccatcaggatcaccactagctttgatggtagaaaaatatgggaaagcctgcagggctttctcttaaagggctaacatatctgtacataacatttggaatgaaatccatctgtaatgagaatccagtcttatagtgtgacctgaggatgattccttttatttgggtgaattaacttatttgtggtgattcttagaaggacctcattgcaagcaaatgcattactcatgtttatgggatgaatgtcattctaagaagaaagctaaaaattatttggaaggattgaggaaaacagaagagcaaatgggagaatgaaaggtactttctctttcaccaaaccctcctgctcctccttgctctgctgtccccattcactctgttccccttgcatcgacctctttatctctacccacccccacccccgtggcctcaaattgattcctggtaagaagaaagccataatctttgagatgggtggttcccttagggggtttccctgcaaagagggacaaaagcccaactgttttcaccagcaatgaattatctgccctgtgtgaggtttgtgtgccctgattccatttcatgcttagcctagattgcacctccaacagtcctttattatccacataccatatagctagggaagcacaggtaataaagcaacatcatcagggaataactgagatacaagactgtgtccccatatgcaggtgacaagatggacagcagttgcagtagctacttggaaaagacaaacaaaaaaggtgtgttaggctgatatataccaatatggaaaaggaatcccaagtgctatctaattctacatctttcttgactcaatgagaggacagcagcccttcctatctgcatcaccaataaggctgatgccatcacaaagctaccccagacctacacaaggggaacctgaaatggtaagaatctatatgccttcaactcagccagtattactcacacaaggacttcctttcctgtgcccaggcaagagccacagaagtttgcacaagaacttggctgtgtgtttccagtttacaaccctgaatatggggatgtaccctggctcctgcatggaattctgcaaccagcaggctgtgcttatttgatggaacaggcatgttagggattggaagatgacctgtcacaataggaggaatagtggccaaaagactgctactatctgccatcccccatggccatggaagaacaaggactttagggacccattaaaaagggttgctaatgcccatacttgaacccctttctgctaaaagtgagctggcagaaattacagcagtgaacctagaagatgagtgtgttccttgttgactcctctcagcagtttgttgaggctttctcaaaatttaccagtgcaaatccagggacagatcaaaaccactctctggtcctgtctgcctttgtctcagccaaggacggattattactcaaatcttagcagcagctacttagttttgggacaagttagagaaagatgaagaagagaagcaattaaaacccactgtaacaaaacaaacaaacaaacaaactccactgcgttaactgttcagttacaactattttccaaaggaatcacgctaccctggacaatatacaaagggagccaaggacaaagagaaaactgtcattagagtaagaagccaggacattggaagagggattatagagaccccagaggatgcagaaattgcaggaacagagagacatcagaaaagccccggagaaaaccctccactaaggaacaactggcaattggctcaaaactggcagattgagggagtttcaggggtggaaactagtgaacagcaagtgtcaataactttccctcaggctaacctatgtttgccttttctggtagatactggtgctacttattctacaactgcctcaaatttctcacttgccgtctggtgacaggttaatacaggctgctgacatttcaggtcagccttctacttgtttctttccctccacagttcccataaagataggtcctctgataacgaacatgctttcttactttctcctgatcctcctgtaaagctattgaagagagacttccttgcagatttgctgtgcaaaataaaggctaccatattctctactccagatggagtgcttgtggagatgcctagaggaaaaggcacctgatctggttactaggtgcctttagaaagaaaaggggatggcagagggtagcaatctcacaccgtgctggaaaagtaccccaaactgaaacaaatcaatgggccccacagaataatgatactggtttaattcaagatatcgaatcaataaaaatagcttaccaaaaagataagctatggccatgtataacacaataacctttgttgagagctcaatcagaaggaataaaaccatgtataaaataattagaaaaaccaggaataattttaaaagggcactcaccctgtaatagtcccatacttctagtcaagaaaggtacatttgatgaagacggacaaccactatatagatttatacaagaccttagggaagtaaatacatttgttgctccattaaccccattagttcctaagcctgcaactactctgacttcagtgccattgtctgaaaatttttcctgtgtagtttatctgtgttctgcattttttttctgttcctctagctaatgagtctaaattctagtttggttttacttatgatgaagttcaatatttatggcaaatggtctctcaagggtttccggactctcctactctttttctcgagccactcacaggaaaacttagggaaatctgtctcactggagggatctgtaattattcaatatgtagattatttgctggtagcctctaaaactgaagaacaatgcaaaactgatactttggctttgttacaattccttgcttggctgggacatatggcctcactaaacaaactctaatactgccaaactgaagtgaaatatttagggaatctattgtcaggtaaaggctgcaattttgcagatgaaagggcctactacaataaaacaactgagaccattttgggaactgctaggggactatagagcaggggattccagcttctgcaaagagcagaaaacctcggatggagcaactgcatggtaattctccaaatgttctgatttggtccctagaatccgaagaggcttttgaataactaagattcgctatggtctctcccctagctttggagattccaaaatttttaaaacctttccacttgttttatcatgaaaataaaagtgctgctagtagaattttaactcacaaggcggggttaggctatagaccgacagctcatttcccagttcttctggatgctgttgggaatgtctggatgcccccgagcagcagcaatagctgccacacttagtgaaaaggctcaaacccttaccttgggtcacctgactcttctgcatacccttcatgctatgacaactatcttacaagtgcataagacccagcatttatcggtgcactatcttattaattatgaatcagcattattacccaatcctaatcttgtgcttggaagatgcaatcttcaaaaaccagctactcttttaccagatcctggtcaaatagatgatcatgagtgtacaacagtaatagaagatactaggcagcaacctgatttttctgatattcccgttcaaaatgcatatttgataaggtttactgaggggcatatacctgggatgagaagggccaccttcagggttcttatgctgtggtgactgctcaggaaatcttgaacgcttatgttttgcctggaattaaatcagcacaagcagacaagctaatagcagctactagggatctacaactcttggttcaggacttaaagtgaatctatatactgacaataaacatgtgtttggtgtctgtcatataataggggaaatttgaaaaaatggattcttgacttcatgggaactaaaatatctcatggaaaattaatatccgagttatgagaggcttcacaactgctgagtaagataaaataatccactgtagagaccacatagtgcagaaaaacaaaatttctaaaggcaatgattctgctgatagggctgtaaaaactacggttaacacacggctgacacccagctcagaagccccaattctaatacaagaaactttcatttattttcaaaaatatgcattccaaaagaaaatgggcaaatggattaaggaaggggggctgtactatgggaattagctaacaatacaattcctactccttggtctttatttacttggttagtattgtgtcagcaacaaaatcagctacataaaagatggattttaaaggtttcagactctcactatgcctaccaaaaagatgaatttattcaaaacatagtaaagagatgtgctatttgtcagcaaaagtcttcacagaccactcccaaggtaagcacaggaagttttccccaatcaacataccaggaacctggtggcagctggatttcatagaatcaatgcccgcagaaggtaagagatgtttgtctagttatggtgtgtatcaatgccctgaagttttgccctcttcaaaagtcattgctcaggcagtggtaaagttttccttaataaacataattctcactttggggatacaaaagcagattgaatcagagtaagaccttttattttctgttgttcaggaattatgcaaatgtttgcagaatcctataaactattatatgctctatcaatcattttcagtcctctggacaagtggagcaaatgaatcaaagtctaaaaaaactcattggccaagattcactgactttctgaattaaaatggccaatggctttaccactagccttgttaaaaattagagcaacccctgctagcaagcatggtatcactccctttgaacttatgtttggcagacctatgatgtgggtctgagaccatgtcctgtgcctaacttaattgaatcttcttataatcatattcaatatttagaagagttcctctcttccttaggaaccctaagatataaagtcataaagatctggatggacctgctggaagaagtctgccacctttttcaaccaggagatttcatctacatgaaagtgttcctgggaaaagacaggccgtcgctcaccttttcaagtgctgtgatcacccactctgctatcaaagtaaaagaaaaatgcagttggggaaaacaaaacaaaacaaaacaaaataaaaaaaaacaaaaaatacagttggatccatgctttccttgtgaagccagcactgacaaaagacttgaaacttaagttttccaaagtaacttacactggtattgagattcgactgaaaagaggtattctggaaaatgtattactactatcctcagagactgccttgctctcctgttcttaataggcttattttgattacttaggaatctcattttactagaaaacaaatctgttatttgttatgctgcttttttttgtactcccagtaatggtctccacaattacacagaccatgtcctgtgcccacgccatgtcctgtttttcctgatgaactccgtattatatcctcagaatacacatgatctggacttttaagtccaacagtcaaattattggcagagtaaaggcagagccatcttcactagtaatgtttgataatactaaagtaacttatatgcatccagatactcaagccctaaatttgatacaaaaatgttttgttaactggtttgactggaaggatccttagagggaaaaaaggaatctgttaactatatgacaatcaaaatacctataggagtaagccttgtgatttattctctcacctttactatggtagaggtttggttcaataaaactactgatagttgtaaagacatcgatggtaaattacatccatactggtgggaatcctttcaacagactcctcaagatgtatataagagagatcccccccacccccccgccactatgtttggaacatggcaatgccctgagcctattttaatagcccccaggattataggtagatggtttttcttgaactgtctgaggctgctgtcagtcttcacagctagacatgtatcccttgagccttcccaatataaaatttattaacaggcctgttcggggacagccaagggcaaaaggagacttattagaatacagtcctgggaggaatagggacaggtacagatatacctaactcttttgagattgagactttgaataataaacatagtgctttgggacaactacaaaagcacataatacaatcacaaactggatgaatcacatcatgtaagatattttaaagcaaggggagctcaataggagtggaaatggaaaacagtatagaattgaactatttggagtaatttaatgacggaaccaaaaatcaccacgaaggtctcagaggttcatttgcacagtccaaggcaattggttataaaatagtcccagataccatcactagttcagaatcacagggctgttcatctttaaggaaatagataggcattagactatgactataccagaagagacagaagaatagtataaaaaaaagcatctacttaatagacaaacaaatctggcagtcacagatagagactgaagaaaacaggtttaatgtcattggattaatttaggatgcagattctctccagtcccagagataaactgggacagataaacacaggtggatgcctgttactcatggggactgacatgcctggtgttactattgatttagactaatgcaagcaatgacccacagattggggttttcctcaagacttaaaacaattagagccaagcacattaagcactggaagttcaatctgtacccttcaaagaatgcctcataataataaactcgataatagccaggggtatctttgtgttctgcatcttggacatattatttgagaagatggaacaagtactacagggctacgttttataaatgtggtgttacttattgattgacaggaactcaggagaaatttattgggtgttataaaactctaaattaaaaattagaatatcaggtccctggaaagctactccttagacttggattgggtaagacttcctaaagaactacaaaataatacttaaacaatcatcttaacaagcaatacggacaacaccagcaatctgtaaaagtaatatatcaggaaatgagaatttctgcacgttgctaatcaagaatgtgctttaaccattcaccactggtaagacatacttaaaggattttcccacaatgcaaatgttgcactgacattttaaattcatcctatggtaattttcataattgcttgctctgcattttactatggatttgctggatctttagatgtctaactgcccaaagaaaggtagaaatggtgagtgacagagaaaattagaaaaccgtagtgttattataaaagaaggggaaatcataaggagaaaaaacatttaaattttccttattaccaaaaggaaaagagaccttcctccctcttcccttttcttagagcatttcctgaaaaaacttgtatttgtaaatctttcctctgctcttttgatatgtgtatgaatttttaaaagctaaacaagtcttctgcctgtattacaatccaagaatgtttattctgattctcttagcctccaccaagaatgtttttcttaagagctcaggagccatctctttgatatataaacatcaaggatcctatctccctgtcaccctgggagtttagcctaggtgctatctccctgtcaccctgggagtctagcctaggtgcctggctctagtctgtaactacctgcatgtcatatatagaagtttgatttttgcctttggataagggcaattaactagcacagatggccactccaattaccaggtaaacttaggatgaattatgaaagaatatacagcaaatggtgctatcagttcttcttacatgagacaagttatttatcttgagaacatgaatccgcccggctacatacaagggtgagatttccttctgtctttgcaatttcattagcagatttcttgtgatgcacactgcagtgtggtttagtgcatattcaataataaaactcttttctttctattctatatttgtggagaggatttcctgggttgccaggaattttatttttaatcttttctccaaca

>hepsi4_10840_5359_bre_proputein

qkspgenpplrnnwqLaqnwQiEGvsGvETSEqqVSiTfpQanlclpFLVDTGAtySTtASNfslAVGdrlIQAaDiSGQpsTcFfPstVPikigpLiTnmLSyfLLIlL

>hepsi4_10840_5359_bre_polputein

AieeRLPcrfaVQnkgYhiLysrwSacgDazrkrhlIWLlgaFrkkrgwqRvaishrAgkVpQtetnQwAPqnndtgLIqdIEsiKiAyqkdklwpcItQzPllraqsEGIKPcIKzLekpGIiLKghSPcNsPiLLVKKGTfdeDgqplYRfiQDLREVNtfVaPlTplVPkPattLtSvPlSenfscVvyLcsAFFsvpLanESkfzFgFtyDEvQylWqmvsQGFpdSPTLFlEHSqenLgksVsLegsVIiQYVDyLLVAskTEEqCKtdTlALLQfLawLGhmASlnKLzyCQtEVKYLGnLLQvKAAILqmkgPTTikQLRpFwelLGdyrAGdssFCkeqktSdgALhgnsPnvLIWslEseeAFEzlRfAmvSPlALeiPkfLKPFhLFyhENkSaAsRiLThKagLGyRPtAhfpvlLDaVgnvWCpRAAAIAatLseKAqtLTLGhLTlLhTlHAmttIlqVhKtQhLSvHYLiNyESAllPnPnlVLgrcNlqkPATLLPdPGQiDDhEcTTviedTRqqPDfsDipvQnayLirftGAyTWDekghLQGsyAVVTaqEIlnAyvLPGikSAQAdkLIAaTrdLqLgsGlKvNlYTDnkhvFgvcHIiGEIzKnGfLTSwelnIswKinIrvmrgFttaezdkIIHCrdHivQKNkisKGNdsADrAvKttVnTRlTPSSeapILIqEtfIyfqkyAfqKKmgkWikeggLLwELAnnTipTPwsLFTwLvLcqqqnQLHkRwiLKVSdShYaYqkdefIqNIvkrCAiCqQksSqTTpKVsTGsfpqstPGTwWqlDFiEsmPaeGkRclssYGvYqcpeVlpsSkViAqAVVKfsLiniILtlGIqKqieSezdllFSVvQeLckcLqnpINyymLYqsfQSSGQVEqMNqSlKkligqdsLtselKWpmaLPLALLkiRaTPaskHGiTPFElmfGrPMVglrPcPVpnLieSsynHiQyLEefLsslgtlrykVikiwmdllEEVchlfQpgDfIymKVflgKDRPsltFsSaVitHsaiKvkEkcsWGkqnkTkqnkKkqkIsWIHAfLvKPAlTkDLklkfSKVtyTgieIRLkRGi

**MER89**

>mer89_10881_1069_bre_chaindnarm

aaccacaaaatgccctgctcctacttagcctgcctccagcttccaacagcaagatccagtcagagcatacctgaaaccttccccttttataactatgaagttttctcactccccttcctgcctttgagtctctactaagcagaagtgatggtggtcaactcccttgctagagcaagctctgaataattagcctctgtttgttttcatttgtgtgagcttcacttgtttctgtagttttcttgaagtttccacagagatactgtctgcactgcatgtcaccatggatcccagttctctgccaggtgcagtacccaccaaggcttcttgtgcctcactgcacacagttgagtctgcattgacacattgggtcagcactggttctgaactctgctctttgattttgagttctttggctatttagtctcactttggtacctaggatttgttatcacttcccatttgattggcatctttggtggagtcaggctattccttttcatctctgttttctctcttttctgcttctgttttgcattgtgcaatctggaagtgttgtcatagagtagaacacaggcataggccctataagcctttaatttgagctgtcctcccagaccaatgtagttttcatttgactggcattcatttggacaaactttgctgtgggttaccaataaaactagataagattatcctctcatctagatttttgttctgagaatttggttttgatccagaaagagcattctctctggtgttatacttgcctggggagacacattgtcaggtgtgcatttagaggcaactcatcctcaaggttgggggctcaagatacaaagtacacaagctttacttttaacttttgccagctcttatgggaccatttaagtcagatttttccactcctacaggaaaaactcctttttcctatatatatatacttattctaattctaattctagtgcccacctctctaaatagcataatttcaccaaagattatttgggctttcaatggccatgctgggaaacactggacttaaacaattatctgagaagcaccttagaaaagaaagagaaaatgatttatcagatgtaatggataattttctaattggtatgcaaaagtctacaaataaaactcagttctaaaattgcttcactataagattctttggttaaagtgaatgagcaatctgatgacttaaacaacaacaaagtagaattatttccttggtaaattctactcctttttggtttccagttatcactctatgttcagttttccttcttcgtctttatttttctgatatttctttttctgcatctccttccttgccttcctctcatgcagaccaccttcttccccaacagtcctcttaaaactcaaaatgccatttgcttctaaggatgcatcccttccatgagctaggtatgtaggcaactgtagaatttaaaccttgaacccaagatgaattaagagctatcactgaagatttccctaaacccaaaaagaagagcaaatagttgtagaataatttggaatttttttgggtgcctatgacccaggagtacctgacctatatcaatttgtgcatatgttggttgaaacatcagatattggatagcaaagactgattgaactgatgcagaaagagacctgcaggatttctctctctacaataaacctgagagtcaaaaaatcccaaaacaaaagacaaaaaccagtaaagtagatcaaagtttcaaaaaagacatatgtgaaacatttccaaaaaaagttgattggaccttaattaaatcatgttaacaaagaaacaaacaagatatgtaactaaatgaggtagttggagttgggggtagactactagaaaataccatttttaaaaattttttgtagaaaacaccttctgacaacactagtctttctacattaaacctgccatctttgctcaacttcttccttggacctaacatagcacacaagtggtaggtgtttcaagtaacccacacatttttttttcctatctctctcacttaaccataaccttggacccttgcctgaaaaatattctctcctgctttgtgataccactgctacaagcttaataggtagagctatgctttgcctatgcaattgtaatattaaatgcataccagaggaaagatttcttgaggtttcagaggattctatttacaatcaagttgtatctggtctatatatacctttgccttctccactgttcttgttgtatacctcagatgaagatcttgaggctgtacctgacactctatgggctaaaatttccactgacagaaaaagtaattggagcagaatctattaaaattcagatacatccttccaaattgctacccaaactttcccaatatctcttaaatccaggagcaaaggaagcctcaaacttataactgaaggcattatattcaaaggcctttttacaacctgcactaccccttgtaatattcctgccctgccagtaaagaaaccaaatggacagggatattgacttgttcaagaactcagagacacaaataaaattgttgtctttacttttccttggtgcccaacccaaatactatcttgtcatcatttcctccccaaaccacttgcttcagtgtagtggataattgctctgccttttcactgttaaattacaccagaacagtcaatacctttgtcttcatctgagaggacaacactacatacataaacagttaggtgccaggggtttactgaaacccattcctatttttcctatgcccttaattaagaactaaaagatccaaatttctctggtgatccagttctgatataacatgtagatgatctcctacttgtttagagaagctggtaaaaagattccactcacttgctctcagccttagcagaaaagggacataaagttttaaaacacagagttttgccaaaacatagttcatttagggcaggaggagaacattctctcctaacagactaagtattatccagacttatcctagatccccagaaaactacagttgaaatgatttctaggtctaatgggatattcttgttaatgggtgccaaattttcctgagattgtgacacctctttatgaaatggctaagtcctcagtaacattccctctcttctgggagcccaagtattgaaaaagcctcctataatctgaagacagctcttcaacagcctccttccgtaggcatcctgaactaaaacttttgtctatttgtgcatgagtgttctggacaaacctcagtattttaattcaacttcataggaattattgaaaacccatcacttactatagccttgccctcaatccagttgcaaatccaagattgtcttaggatattaccaccactatagaactggtcaaatctgctgaattagttctaggcttcctgcttgacctgatgtttcctcatgcagtacagactttaccacccattgaggcacaattaacctcctaagagttccagctgcactctccatctcacattactattcactgctgcatcctgccatcctcctgaacttaccaaaagaaggggaacctcatgaatgtcttaccttagttagagaactttaaatactttgctcagatgtattagtaactctcattgaaaatcctgaccttttcctgaccttatgttatttgttgatgaatcatacctctgataatccttggaactcaagatggccttttggtggccccaagtgatttcaaatgagtattagctgaatttctccatgaagctactcattatgttaaagacaaattgattactatcttaaatcaacattggtgagaaaactttaaaaagacagctgagattgttttggggtcatgtgttacttgtgaacaacataatcctggaaaaattataaaggtgaggcatggccaagaaccaaagcctcaaagaccctttgaacactttcagatgggatttgtctattttcagaatgggttgaataattttcttgctgaaaatctccagccttttcagtctctaaaaagcttcttgattttatgtttccaactagggtatacaaacttttatgtcaactgactgaggcacacaatttataggaaccattataaaacaacttaataaggtgataccccttctcaaaaactatactgcctttaccacccacaaacttctgtaaaggttgaaagaaccaatggcatccttaaattcaaattaagaaaactttcataaatccttgcacttccctgctaaaggtaatcacctgagctcttacggccatataatccattattttggggtcatattgattatgaactgctaccaggaagacccaagcatttagaaatttcatcctgatgctagattcttccatgctgcatccagatatgggaaaatattgcaagggactcatgtactatacccagtcctatcaccaataaattaaggccaccttgctacaacatcttcttacattggctcttcaggagcatctatcaggaaatttcatctattgaaatagacatcggataaaaaactgctcttgaacctcaatggaaggaaccttatcaggtactgttaacaacaaatacagcagtgaaactgcagcagaccaatcctcaactttatgtttcaccactaaagaaacaagccagaattccacacttttggaagactagtccataggatacctcatgctgagaattctcagagatcctcaaaaacagcctatctttggcaatagacatttgacccaagacctttcaaacaagcagatgtcctcttaatggacagcttctacctaacacattggacaaagacccctgtataattcctcttttgtttttgacctgcttattgataatcattcttctcgttaactatggaccactggttattttctaccataacgatcccttttttcttttggtaccctctttgttgacaggtcagaatatacttattctaacacaattctcagattatgtgaaacagtagccactgccatcaatcttactgactgttggatatgtcatccatcattggacccccaagataaaaacgtgtgggtaattccagtctcatcaaattagaccacagggacttatagcctatggcagtttcacttgcacctactggaaaaattaacccataaccttgctagtttcttttctcccagaaaaataatgaagtattaaaatctgtttgtcagatcccaggtaattctaaatttgaattcaacaacttttgaaggttgatgagccaaatacctgtcacctgaaaaagactaatagatatccaaatatgtaacccagcaaacgtaaaggccttggttctgacttcatgtcatgcacatctttttcttgcatttccatgtgtctctaacgctattacttcctttgcacccaggacacatgatcctctttacctcagactaacacttcatgcactttaataatggtattccaagacttgtctgtatacagagactctaattctataaaccaccaaggatgaaaattttgactcagatgcaaactccaaaaggaggccaccttgattattcaagtactctacctgaaggaattactgactcactgtttatatgaacaatcagagctgttttctcaatgttgcgaatcatacaaatagaaagagactgtaagaaacttattattaactctggcagaagttattagtgatatcacctccgccctggaggaaatacagaccgatgccaattcattggcatgagcagtgatggacaatttcattgttcttgatttcttgtttgctagtcatggttgtgtctgtgccattgttatatatacttcttgccatgtgaaacaaacaaggtaagacaggctaaacaacatctgaacaaaagagcttattggctctgtaagattgatcctcatggccttcaggaattactctcttggcctgggttaggcagctggggttcctgtttctgaagcatcttacagggactgttaattattgttgtttatgtcaatagtcattatgatgtaggttctttgcattctatctgaagttctatatatttctgtgcagctgctctttcatcacgtggtcatgatcatgacacaacaaaaaggtcaagatcttgcaatatggttgactatagagataattggagaatccccaagtggtaaagatatggatggatatttagccttctctgaattggtcaatctcttggaagtgaaagaatgaccaaaagaaagggctgagaaagggctgagagactgaatattcaaaggacaatcaaagaacagttggacaaacctctgcagcagttagcccagaatggtcaggatttggtcatgactgccagtttctctacttttttttccccccagagccaaccagagattcatatgattttatcccagtttaggcatcttaaattatagttgtactcaagatgggttttcccaatattaaagaaaacccaacaatatagtaaaactacacacacacacacacacacatacacacacacagacacacagaggaaataaatgaagatcactcaaatgagaataaacaaaggccatttattcagagtctgatatacatagtaagggatttagcatttggcagagactcaaagacaggaaagggagtgggaaaggtttatagtgaaataaagggcaactttcaggaatgctctgattaagggtatggggaatctggatgtggattacctagtagcaggacatcttattctttggggagcatatttggatcatatttggagcaacaagaaaaagccaaataaatagctttctagaggtataactgacatccaattttatatatttaaagcataccatttaatgagttttgacacacacatccctgcacaaagtcacatgcaagtcacctatgttatatacacacatacagctgtggaaccatcaccacagtcaacaaaataaatatccatcaccctccaaagttttctcactcttttaagtctatctttcctgcccctccctgttccctttcca

>mer89_10881_1069_bre_polputein

nQVVsGlyiPLPspLflLyTsdEDLEaVPDtLGLKFPlTEkviGAesikIqIhPsKATqTfPISlKsrSKgslkLiteGIIFkGLfttCttPcNiPaLPVKKPnGqgYzlVQeLRdtNKiVlyfslVPNPNtiLSSfPPqtTcfSVvDncsAFsLkLHqNSQyLclhlRgqhyIhTvRcQGFtethsyFsyALnzeLkDpnfsgDpVLizhVDDLLLvzrSwzKdsThLLsaLaeKGhkVlKhrvLpKhssfraGgEhslLTdzvlsrlILdPqKTtVemFLGlmGYsczWvPnFpEivtPLYemaKsSvTfPlfWsPsieKAsyNlKtALqqpPsvGiLnznFcLFVhEcSGqtsVLiQLhrnyzkPitYySlaLnPVAnPrLszditttIeLVKsAeLvLGflLdlmfPHAVqtLPPieAqlTSrvpAalSishyySLlhPAiLLnLPkEGePHeCLTlvReLziLcsDVLvTlIeNPDlfLTLcyLLMNhTSiIlGtQdGLLvApsDfKzvLAEFLheAtHyvKDkliTiLnQhwzEnfkkTaEIVlgSCvtCeQhNpGKIiKVrhGQepKpQRpfeHfqmGfVYFqnGLNnfLaenlqpFqslKsfLIlCfqLgytnFyvnzLGTqfigtiIkQlnkVipLLKLyClYhPQtSvkVERtNgILKfkLrklSKsLhfPAkgNhlssyGhiIhYfGvilImNcyqeDpSizKFHpdarFfHAiQIwEnIaRdsctipsPitnKLRPpcynIflHwlFRsiyqeiSsieidigzkTaLEPqWKePYqVLLTTnTAVKlqqtnpqlyvSpLKkQaRIPhfWKtSpgyLmLRIlRDpq

**HERV24**

>herv24_10872_4820_bre_chaindnarm

tgtcttttttttttctgtaaataattaagactaaatggcaccagagatgagactccttcagaccatttttctcccctcacagagtaataaagtaatctccttggaatgtagcaatctgtaaccaatcaaatcaccatggcttacacactggtcttttatggaagatgtaatgctgctaaaatttctgtctctgcccatataaatgaagccgtaacttctccacttaggaacactggctccattcatttggagtctgtgtttctggctggccatcctcaagctttgcactagaataaactctacaatgaaccacattttctgagtcttgttatttaaggttgacattttggcaaccatgaagggatctgaatcagccctccagcaacccccgtcacttcactgacaactgaagccttggtaccaggatgaccaacctttgttcatctgacttcaccagagtcaaaacaaatctctggtaaggccctctcaggtgtgaattctcctggccttggttgaagatacagactttatttgagctacacacatttctaacttagtggggttgaaattgaagttctactgcaaagtagaagcttcattttttattctctaaaaagtctgctaattgtggatttgtaactttcacttttctctgaaattaaaattttgtttgaaattactccttagagtttttcaaccatttctctcattccaaatcatagtcagggaaaaaacagtttctctttgaaaagtagaaatgaatctttgtgacttaagcaaaaaaaaaaatttaatctttaaaactggccagattctaaagcttggttcaattgacaaatttttgagtgatcaaaatagtaaaaggctatgagcctagaagataagagacactttctcctgaccaaaaggcaccttaggtgactaaaatctcctggaactatgggagtttattgctttcaatgtaaaatgttctcacggagaatcccttaggaagaatatgcagggaaccttgcttaatccagtgggcacacaaaaaggttgagcacctggtgccttaagcacccaagtctcttggaaccaccaagacatgtaaaaggaaagaactgactcaaggatggccccttgaggagctattctcacaagcatcacactcgattcaagacatacccccgaggtcattttggtaattcaaaagaagagtctaaattatgagcaattatgcttctaaaagcatgtcttcttcctccagaaaagatccacctgtggaaacaccagctagatttgtgtttcatacatgcaaatatatttttaaatggtctcacataattcatgatgaccccaaattacaatgattaaaagggggtacctttgaatacctaatttacctttacactcaaatagaaaaggccagctctaaaatgaaacaaaataacttggagagctattttatttttatttatttttttcttgtcctgcttcgtaggtgattactggaagagaaaactatttaaatgggacttggaaacttctaaaagaggttctgataaagtcatttcttcaaacaaacaaacaaaaaacagaactcatttctttgcaagaagaacatgaaagattgtctaaaactgtttctgaatttaaaaagactgcctaaaatctctccccttcttcagctcctcttgtcttttccttgtccagacttaccttccctcctccttttcttccttctgctgttctggctgtatttcaggaatactctgtgtcgagtagggagaatctgccttggtttaccaaccactgtcaaaagtagaacttaaaggcatattttgtcgttgttgttttagacttgggggcacatgtgcaggtttgttgcatgggtatgttgcatgatgctaaggcttgggcttctattgatcccatcagcaaaatagtgagcatagtacccaataggtagctttttagcccttaccccctccttcactgcccacttttggtgtccccagtgtctgtcgttcccatctttatgtctgtgtgtatccagtgtttaactcccacttaaacgtgagaacatgaggtatttggttttctgtttctgcattaattcacttgggataatggcctccagctccatctatgttgctacaaaggacataattttattctttttgtggctgcatagtattccattgtgtatatgtaccacattttctttatccagtccactgataatgagcactcaggttgattccatgtcttcgctgttgtgaatagtgctgcaataagcataagagcacagctgtctttttggttgaatggtttatttttctttggatatatactccaccatgggattgtttcatcaaatggtacttctttaagaaatctctaaactgctttccacagtggctgaactaatttacattcccaccaacagtatatatatgttcccttttctccacaaccttgccaacatctgttgttttttgacttgttaacaatagccattccgactgatgtgaaatggtatctcattgtggttttgatttgcatctctctgatgatgagtgatgttgcaggattttttatatgtttattggccatttgaatgtcttcttttgagaaatgtctgttgatgttctttgcctactttttaatggtttgttgttgttgttgttgttgttaaaggaataattaaagaattctcagattctcttccagaccctaaggaattttctggagaatgtgaattaaacacttgaacttatggccccagattctctgatttatatcaattggttcacatgttagtgtcataaagtaaagccaaggactggatagcaaaggaaaattggaggaatcctttagaggacttatgtgaattttctgaagcaggcagtaaatgcacctgcaaggctgccaaaacttcactacacattatccccttgacccccttatgcctagtgttccattattggaacgttagggatgtgggagttttttatatcctattgctcaaggtcattgccaaggtctgatttctcactcatgcaaacatttgaaaaattgcaatctccagcataatgggttaattttccaaagagtagttgattggaacaaaacacaacaatgttgacaaaatcttgatgagtcagtaatatcatactagaaaatatttgaaaacaacgtttaaacaatatttgggtctatcagaagaaatttatgctaaccatcaaaatgatgcactcataaatggattacatgaagaattggccactataatagagacatcactcattgggctacttctcaaactcatgatctagttaattttgctgatcaaataccctgtaccctaagtaaggaaggaaagaaaggggaagttaaacagaaagaagaagcaaataagatcatgagtttatgactaaaacaataatccaacaaatttttaacccctaaacagcctaataagttccaaagctgacctaatccctcattttccaaatactgaaaaaaaaagcctgaacatttcaaaaggattatagaaattttaaatagaacagcaacaggtcaaaggaagagaagaataggaatgatccaaggaaatcaaagggactttctcttttatcctcagtaataccatgggaaaattgaaattaatttaaatgaagaactaacatcagctctttttaatactgaagctacattatcagtgataaatcccaccttatacaaggtcccattccttggagtgaaaaagccatacaagtggtaggtttaagaaatacccctatatctacacaaaaatctcaaactatagtttttcagttagatctcctaaacatatgtttccctcttggtttcctcattccccatccacttgatagatgaagacttttaagaattttacaaataaatacccacatctctctctttcaaaaggggggaaatgtatccagaattaaaagataaaatagaattattaggttcaaaaaaattttgaaatctgattcttccaattcacaaattgcaattcatattgccatggaggaaactaaattgttttagaagaatgacaagggctgttaaaaatagtacctgatagactccaattaaaatgtaaataagtccatcggaacctctttcaaaccttaagcaataccctctcagaggtcttggaagggaaaaaacataattttgaattacataaaaggaggcttgattattcattgtacaaacccatgtaacacttccattctcaccataagaaagccaaatagtggaaggtggagatttgtacagaacctgagagcaagtaataatgtagccattcctcaaaatccagtggtaccaaacctttatatgttgttgactgccatcctaactgaaggtgaattctttgctgtgatagacctagatagtgcattcttcagcactcctgtggataaagatggtcaatttctctttgctttcacttgaaaagacagacactgcacatggaagttatgcctcagggatatagtgagagcccaacttattttttacaaatattaaaagcagacctatcagatatcaactttcctaagaaatccaccttgatacagtatgtagataatttacttctcaggggataggaaagcctccatagaggttggaattacaacaattagctttaaagtgatgcaagtttcaaaggaaaaactaaagttctatcaaaaacaagtaaaatatttatgtcacctaatatcagaggaagccctttttattaatatgggtagaataaaaggaatattggtcttctctaccctaaaaactaagaagcaagtgagaaagtttttggtgttggcagaatacggcagaaattggattctaaacttctccttaaaaacttaacctttataaactcttttaaaacaagacaagccagacgccctggaatggacagaaaaaaatcagatgacatcaaaagaaatttttaaaaagtcttatagatgccccagccttaggacatccaaattagaatatttcattttcatcgttcatgaaaaatcagggaaataccttaggcattttgactcaaaaacacagagatcaaaatagaactgtagctatagtcaacaactgaagctgtggctaaagaattgccactttgcatgaaagcaataattgttactttatgaataaaagcaactaaagaaatagtgataagatatcccctcactgtctatgttccacattctatggaagtgcttctaaatttacaccacactcaacactattcacttagtatactggcttctcatgatgttcttctttctgctctccgtaatactgcctccagatgtaataatctaaatattgccacacttctactcctgctttcagacgaaatgttacacgattgcatagtcttaaccaaccagcttctctcttctaggactgacctgtaagaatcctgccttactgatattatttggtttacagatggctctcacttaaaggatgaatctggaatttttcacgcaggctatgctatagcatttttgactgaagaaatagaaagcgcttatctttcaggaacaaacttgactcaacaagcagaatcaatataattagagcctgtcgattggcaaaagaaataactgctaatatttatacagatagcagacgcgcttttggagttgcttgtgactttggaatgctatgaaaacaaggagtattttagccttttccaatcagttcattaaaaatggacaccttgtttcagaaatattggaagccatactgttgccaaaatcattagtcattgtcaaacttccaggccattcaaagtaaagcatcccagaaagccaaaaagcaatggaaatcaattggcagatagtataagagaaagggctgccctaaatgtatctgaataagaaaatcaacccattttagcttttaaggaagcacttgattttttaataaacaattagctcaatccaaagctccaaaaacagaacaagaaaattgggcaaatgtactcctctgagactgggctatggtaaagaccaaatggtttacccatactcacagccaagttactgtcatctttcctagcttatgtgcatgggttaattcataggagccccgataagatgattgcaatgttgtttattggaaatattattgttgattgaaacaatattattggaaatcttcaactgttgctcataaggtacatgaatcgctgccacatctgcctaaaacataatttagggaaacctttacatagttcttaaggtcatcctcctttgtaagaaggcccttttgaggtatgacaactagatattattcagctatcaccctcacaagaatacaagtacagtctagtgattgcctgcatgttttctctttgagtagaagctatccatgcagaatagcaaaaaccttagcagtaagtcaaattctctaagaaaaaaatgttacaacttggggacttaacctagaacttcaaactagcagagatactcactttactggataaacaatcaaaatctggcccattctgtaacatttccattgtgcatatcacccccagtcatatgggttagtggaacacaaaaaatggaacaatcaaaattcagttggcaaaattaactgaggtcttaacaatttttggccaaagacccttttattggttttgcttaacctaagagtgaccctttttataaacaccagttgtatccatttgaaattataacaggcagacctataaaattgtctctaggaaattgcgaatctatgatactcaaaggaggtatgttttactattgtagaggctttataaggcagctaactaaaaactataattaagtaaaagactctttccacagtgagctcctgggagaccaaaaactcaagggccacggacttcaaccaggagattttgtcttttgggaacaatgtctttcaaaggacttcatccaattaaggtgaaagggactttatgagttgctcctaaccaaaccttttgtcattaagctaaaaggtatcaattcctggatgcccatcatccatttaaagagagctgcctttaaattctaggagctttttggaggagtctttagggttttctaggtatacaataatatcatcagcaaacagcaacagtttgacttcctctttaccaatttggatggcctttatttttttctcttgtctcattgctctcagcaacctggttgtaacaggagactactattctaagtgaagtaactcagaaatggaaaaccaaataccatatgttcttactcataagtgggagctaagctatgaggatgtgaagtcgtaagtccaatttggacttcatctcaaacaggtgaactaaaattaaaattctacagaagcagcaaggataagaagacaacatctgagctagtcagcgttcccaaggtaccagatcacaccagcatctaaactattgcttaatattaatacttgtcaatagatggagaaagtcttcctgtgcaagttcccactaaatatttttcttttcatccctaattctcatattttgttgctgctattaccagctgttgcccattaagcaaacctctactggcaacaggctcacgactgtgctaataaattaccgaaaaaaaaaaaaagcactttttttaatattaataaaacaaaaatttttttaaagtcacattttgaatattcaacctcatgccccttcttagtggctctggcctgctatgtgtgtatctcttttccaaagacaagataggataaaatatcagaaatatacctgtgtttctcaagaagagtcaccaatgcttagcactagtatgactaaggatggtgtgcatcactgccctattgataactctttacaaagcaaatggcgtgggaagagccttttggtaaaggagacccagtttgttagctttaatgctggcatcgctccaacttagagataagacacttcaatttggggatggtataacacaaattgataatataacacaaatggctgtatctggcttaccccctcttttggtcagcttagtcagctctcctttttatggtagagaccaaagaactacaccaaaaacacatggcccaacagcactagggacatggagtagttacccaaggaacagtatgttcacacaccattatattacacagtgctaattggcatgtccctaaatgggcacaataaccacgtacttattggcttgctccaaatgaaatgtcttgggtatgtggtaccaatctatggccatgattactcccaggatgtaaggaaggtattcttttgagttttgcctggacacaaagtcaaacagttcataccctcttaaagcctgcaaatcttccatatttacaatttcagtgggtttgttatgtatttcattgagatgatcatttagcttccatcttctagccacaactagcataagaggccccaaccaattacacaaaaaagaccctaaataatatctacgtgggtctctcattatctcattattaaataatgaagttgctcttatgagaaaggtcgtcttacagaactgtatcactttagacatcctcaatgcagccgtaaggaagcctgcactgtcataaagactgagtctggcatctatattctagatgaatcagatagcatcactaaattaatggctgatacagaagcccaaataactaacctttcagatccaacaactaccctaaataattggctaagaagctcatttggatcttgtgaaacttggtggcactagctgttacttactatcagaattataattatgtgtgtgttttgtcctgcttttgtctttattgctgctgtggcacttgttcacaatgaagtcaacacacaacacacaaatgaacaaactaaaataatggtcactcaaataattgcattaatgaaagatgcagatcacaaagtcacttccttcctgttactttaaatttgatctgtatcttgtcagctttgcagcctgaaaacttccttctcccccagccccctactctgaaacatgacactgggaatgagcattcctgaagacacggactaagctcctgaacataatacgagccaaaaccatttgagttcatctaaaatcctttctttgaaagatctcaatttaaaggggaaaatgttttttctagttatgtgaagaatgacggtggtattttgatgggaattgcgttgaatttgtagattgcttttggcagtatggtcatttttacaatattgattctacccatccatgagcatgggatatgtttccatttgtttgtgccacctatgacttatttcagcagtgttttgtagttttccgtgtagaggtctttcatatccttggctgggtatattcccaagtattttactttttttgcagctattgtaaaaggggttgagttcttgatttgatcctcaacttggtcactgttggtgtatagaagagctacttatttgtgtacattaattttgtatcctgaaactttgctgaattcctttgttcattctaggagctttttggaggagtctttagggttttctaggtatacaataatatcatcaacaaacagcaacagtttgacttcctgtttaccaatttggatggcctttatttctttctcttgtctcatttctctcagcaacctggctgtaactggagactactattctaagtgaagtaactcaggaatggaaaaccaaataccatatgttcttactcataagtgggagctaagctatgaggatgtgaagtcataagaatgatacaatggactttggagactcaagggaaagggtgggaagggggtgagggataaaaagactacacattgggttcattgtatattgctcagatgctgggtgcaccaaaatctcacaaattgtcactaaaaaacttactcatgtaaccaaataccacctgttcctcaaaaacccataaaaataaaacattaaaaagggggaaatgtgaaattaaatattcaaacttaaagctgttggaacttaaatcattccaagccttgagaggaatatagctatgcagcttgagtcacatggcatatggctgcaacctctgccttttttatttcctgtaaacaattaagattaaatggcaccagagaaaagagacctcctcagatcattgcccctcctcatggagtaataaactgatcttccttggaacatagcaatctataatcaatcgaatcaccatagcatacacactgatcttgtatagaaaatgtaatcctgaaagaaagaaagaaaaagaagaaagaaattgtaatccttctggaacttctctgtctctgcctatgtaagtaaaactttaacttttccactttggaatgctgaccccatttattcggaattggtgtttctgtggaggccattctcaatctttgtgctcaaataaactccatacttaatcatattttctgaatctcgttatttaaggttgacaggtgtcctgttgggcaatgttaca

>herv24_10872_4820_bre_polputein

AkVGGsLEqELETSKqSKLLFlQkikiVPinVnKSigTSfKpzaIPsQrswkGKniIlnyikGgLiihCtnPcNTsiLtirKPnsGrwRfVQnLRasNnVaIpqnPvVPNlYmLLTaiLtegeffaViDLDsAFFstpvdkdgQfLFAFtkRqtLhmevmPQGyseSPTyFlqiLkaDLsDInfPkkstLiQYVDnLLLRDrKasievgitTiSfKvmqVSKeKLkfyQKQVKYLchLisEEAlfinmgRiKgILvfSTlKTkkQVRkFLvlAeYgRnWIlnFSLktzPLztlLkqdKPdaLeWTEknQmtskEIfKKSyrcPsLrtskLeyFiFiVhEKSGkYlRiLTQKhrdqnRtVAiStteaVAkelPlCmKAiiVtLziKAtKeiviryPLTVyvPHSmEvLlNLhhTqHYSLsiLAshDVllSAlrnTASrcnnLNiATlLLLlSdEmLHDCivLtnqllssRtDLzEscLtDiiWfTDGShLkdEsGifhAGyAiAflTEEIeSAyLsGTnltQqAEsIirAcRLAKeitaNIYTDSRrAFgvAcdfGAmKtRsiLafsnqfIKNghlvseiLEAIlLPKsLvIvklPGHkvKhpRkPksnGNqLADsiRErAALnvSEzenqPILAfKEaldflinnzLnpKLqkQnKKiGqmYsseTgLwzrPnglpilTaKLLSsFLayvHgLiHrSPdKMiAmLfigNiivdznniIgNLQLlLirymnrChiClkhNlGKplhsszGhpplzegPfevzqlDiiqlsPsqeYKYsLVilhvFSlsrsYPcriaKTLaVsqILzEknvttwGlnleLqTsrdthFtgzTikiwpIlzhfHCAyhPqSygLvnTkNgTikiqLAkLTEVLtifgQRPfywfcLPKSdPfyKHqLyPfeiItgrpIklSLGNceSmIlkggmfyycRgfIRqLTkTiik

**MER34**

>mer34_10860_2836_bre_chaindnarm
[truncated: 306,554 more chars]
